# Visualization of pseudogenes in intracellular bacteria reveals the different tracks to gene destruction

**DOI:** 10.1186/gb-2008-9-2-r42

**Published:** 2008-02-26

**Authors:** Hans-Henrik Fuxelius, Alistair C Darby, Nam-Huyk Cho, Siv GE Andersson

**Affiliations:** 1 Department of Molecular Evolution, Evolutionary Biology Center, Uppsala University, Norbyvägen 18C, S-752 36 Uppsala, Sweden; 2 Department of Microbiology and Immunology, College of Medicine and Institute of Endemic Diseases, Seoul National University Medical Research Center and Bundang hospital, 28 Yongon-Dong, Chongno-Gu, Seoul 110-799, Republic of Korea; 3 Vector Group, Liverpool School of Tropical Medicine, Pembroke Place, Liverpool L3 5QA, UK

## Abstract

**Background:**

Pseudogenes reveal ancestral gene functions. Some obligate intracellular bacteria, such as *Mycobacterium leprae *and *Rickettsia *spp., carry substantial fractions of pseudogenes. Until recently, horizontal gene transfers were considered to be rare events in obligate host-associated bacteria.

**Results:**

We present a visualization tool that displays the relationships and positions of degraded and partially overlapping gene sequences in multiple genomes. With this tool we explore the origin and deterioration patterns of the *Rickettsia *pseudogenes and find that variably present genes and pseudogenes tend to have been acquired more recently, are more divergent in sequence, and exhibit a different functional profile compared with genes conserved across all species. Overall, the origin of only one-quarter of the variable genes and pseudogenes can be traced back to the common ancestor of *Rickettsia *and the outgroup genera *Orientia *and *Wolbachia*. These sequences contain only a few disruptive mutations and show a broad functional distribution profile, much like the core genes. The remaining genes and pseudogenes are extensively degraded or solely present in a single species. Their functional profile was heavily biased toward the mobile gene pool and genes for components of the cell wall and the lipopolysaccharide.

**Conclusion:**

Reductive evolution of the vertically inherited genomic core accounts for 25% of the predicted genes in the variable segments of the *Rickettsia *genomes, whereas 75% stems from the flux of the mobile gene pool along with genes for cell surface structures. Thus, most of the variably present genes and pseudogenes in *Rickettsia *have arisen from recent acquisitions.

## Abstract 2

Variably present genes and pseudogenes in Rickettsia species tend to have been acquired more recently and to be more divergent from the genes conserved across all species

## Background

Pseudogenes represent a heterogeneous collection of sequences, ranging from genes with an internal stop codon or frameshift mutation to extensively degraded genes. Pseudogenes and noncoding DNA were originally considered to be rare in bacteria. However, a recent genomic survey identified 7,000 pseudogenes in 64 bacterial genomes, a large fraction of which had arisen from 'failed' horizontal gene transfers [1]. Recently evolved pathogens in particular have many pseudogenes [2], and the genomes of intracellular bacteria such as *Rickettsia *and *Mycobacteria *have exceptionally high fractions of noncoding DNA and pseudogenes (>25%) [3,4]. This has been accounted for by reductive genome evolution and small effective population sizes [5,6]. Also, increased exploitation of host metabolites and reduced selective pressure for rapid growth in the nutritionally rich eukaryotic cytoplasm may allow mutations to accumulate in essential bacterial genes. Furthermore, it was suggested that the reduced threat of genetic parasites in the protected intracellular environment has lowered the genomic deletion rate, making pseudogene elimination a slower process [7]. However, this model was based on the assumption that horizontal gene transfers are rare in intracellular bacterial populations.

As more and more genomes are sequenced, it is becoming increasingly clear that obligate host-associated bacteria are not immune to the spread of genetic parasites. All kinds of mobile elements, plasmids, integrated conjugative elements, prophages and transposons have been identified in one or another species of intracellular bacteria [6,8]. In fact, the most highly repeated bacterial genome identified to date is that of an obligate intracellular pathogen, namely *Orientia tsutsugamushi *[9]. This genome contains about 37% repetitive sequences (>200 bp), most of which represent clusters of deteriorating genes for conjugative transfer systems and eukaryotic-like proteins putatively involved in host cell adaptation processes [9]. The intracellular arena hypothesis posits that the transfer of mobile genetic elements occurs in these populations but is restricted to intracellular bacterial communities that infect the same hosts [8]. One expectation from this hypothesis is that the circulating pool of mobile elements may be different for free-living and intracellular bacterial populations. Another prediction is that the recent evolutionary history of mobile elements in intracellular bacteria follows host specialization patterns rather than the phylogeny of the bacterial core genes.

With the growing realization that mobile genetic elements are circulating among obligate host-associated bacteria, it is time to revisit the source of the many pseudogenes in these bacterial populations. The genus *Rickettsia *represents an excellent model system for such studies; genome sizes are small while pseudogene contents are high. Furthermore, the availability of genomic data from multiple *Rickettsia *spp. [4,10-13], now also including the closely related outgroup species *O. tsutsugamushi *[9], and the more distantly related outgroup *Wolbachia pipientis *from *Drosophila melanogaster *[14] and *Brugia malayi *[15], provides all of the raw material needed for such a study. Because we wished to study the deterioration process over time, we placed the analyses within a phylogenetic context, with the underlying species tree essentially as outlined previously [16,17] with three main groups: the spotted fever group (SFG: *Rickettsia conorii*, *Rickettsia sibirica*, and *Rickettsia rickettsii*), the transitional group (*Rickettsia akari *and *Rickettsia felis*), and the typhus group (TG: *Rickettsia prowazekii *and *Rickettsia typhi*). *Rickettsia bellii *is the earliest diverging lineage in the genus and is a member of the ancestral group *Rickettsia*.

Previous studies of pseudogenes have either traced the degradation pathway of a few individual genes [18-21] or identified pseudogenes *en masse*, ignoring the various stages of the degradation process [1]. With the aid of our recently developed software for visualization and comparison of closely related genomes, we have performed a large-scale analysis of the deterioration process, in order to investigate the source and the ancestral function of the variable segments in *Rickettsia*. This study was implemented to provide a general model for the evolution of host-adapted bacterial genomes, including lineage-specific expansion and deterioration of host-interaction genes. We also sought to understand better the connection between the load of pseudogenes and the spread of selfish genetic elements.

The results suggest that the variability among *Rickettsia *genomes is due to a slow and steady accumulation of mutations in essential genes, as well as to a more rapid degradation of genes acquired by horizontal gene transfer at the base of the *Rickettsia *lineage. In addition, the circulation of genetic parasites across the modern species continues to generate variability.

## Results

### Identification of positional orthologs

We developed GenComp for the visualization of gene order structures and pseudogene relationships across multiple closely related genomes. The program was applied to a comparative analysis of eight *Rickettsia *genomes and a closely related outgroup, *O. tsutsugamushi*, plus the more distantly related outgroup *W. pipientis*. We first predicted open reading frames (ORFs) with the aid of Glimmer [22] using similar settings for all genomes. Homolog identification across the seven most closely related *Rickettsia *genomes (excluding *R. bellii*) was accomplished by basic local alignment search tool (BLAST) searches [23] followed by clustering with the aid of Tribe-MCL [24]. A total of 9,450 Glimmer-predicted ORFs were clustered into 2,940 homologous gene families when applying the length ratio criteria 0.80 for homologous groups, with up to 59 genes in each family, including 359 single-gene families.

Information about gene location was considered with the aid of the visualization component of GenComp to identify the final set of positional homologs. This resolved many of the clusters with large numbers of homologs into groups of true orthologs that are conserved in two to six species or, in the full set, seven species. The conserved orthologs were fused into 86 metaclusters (segments with conserved gene order structures). These represent from 84% (*R. felis*) to 93% (*R. prowazekii*) of the *Rickettsia *genomes. Present in the metaclusters were 665 gene families with a cluster size of seven, representing single-copy genes that are conserved in sequence across all seven taxa, using 80% as the length cut-off value. We refer to the positional orthologs present in all seven species as the 'R7 core genes'. In total, this set comprises 688 genes, which accounts for 62% of the TG genomes and 47% to 56% of the SFG genomes. The mean size of the R7 core genes in *Rickettsia *was 1,006 to 1,010 bp (median size 850 to 860 bp) per species (typical of bacterial genes; Table 1).

**Table 1:** Gene sizes of the R7 core genes and R2 to R6 strain-variable ORFs located in the variable segments

RNumber	Number of ORFs	Mean size (bp)	Median size (bp)
R7	4,816	1,009	856
R6	366	976	744
R5	225	1006	579
R4	304	567	330
R3	558	404	237
R2	696	332	219

'Rnumber' refers to the number of species carrying open reading frame (ORF) homologs. bp, base pairs.

### Identification of *Rickettsia *variable segments

**Figure 1:**
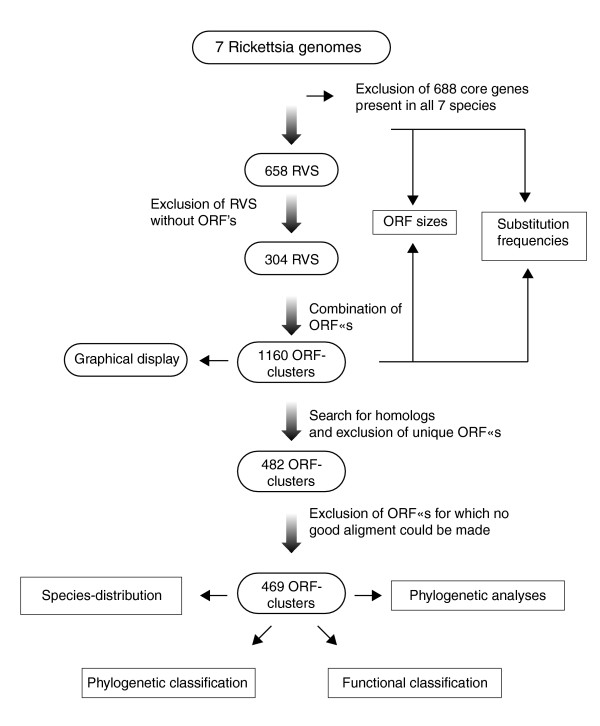
Identification of strain-variable ORFs. Presented is a schematic illustration of the process whereby strain-variable open reading frames ORFs located in variable segments of the *Rickettsia *genomes were identified and analyzed. RVS, *Rickettsia *variable segment.

We identified a combined total of 658 *Rickettsia *intergenic segments, defined as sequences flanked by orthologs in the R7 core gene set. These segments represent from 30% of the TG genomes to 38% of the *R. felis *genome. Glimmer-predicted ORFs located inside *Rickettsia *variable segments (RVSs) and present in one to six species, or present in all seven species but differing by more than 20% in size, are here referred to as the 'strain-variable ORFs', irrespectively of their origin, size, and functional status. An ORF-cluster was defined as a set of positional homologs conserved across two or more species. Paralogs located in different genomic regions were manually sorted into separate ORF-clusters. In total, 1,160 ORF-clusters were predicted in 304 of the 658 RVSs; these were used as the starting point for all subsequent analyses, as schematically outlined in Figure 1.

The ORF-containing segments ranged in size from a mean of 510 bp to 854 bp per species (median sizes: 143 bp to 186 bp) and contained on average 3.82 ORF-clusters per RVS over the seven species. The longest RVS (reg_id 546) was 19,665 kilobases and contained 29 ORF-clusters in *R. felis*. The sizes of the strain-variable ORFs were found to be roughly proportional to the prevalence of the ORF across the various species. Thus, strain-variable ORFs identified in six members exhibited a mean gene size of 976 bp (median 744 bp), whereas the mean size of strain-variable ORFs solely present in two species was only 332 bp (median 219 bp), which is only 30% of the R7 core gene size (Table 1).

### Rapid sequence evolution of strain-variable ORFs

**Figure 2:**
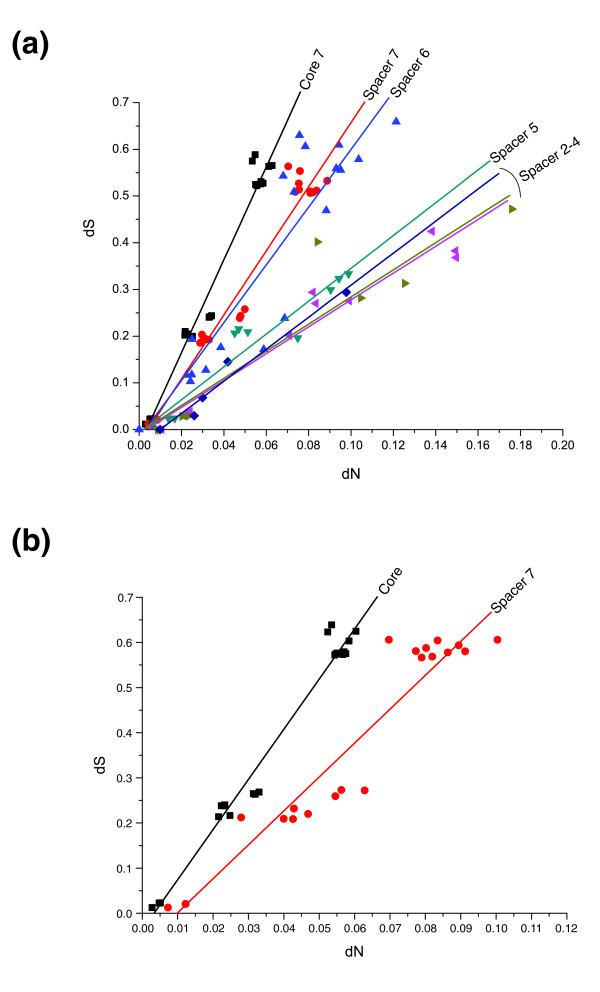
Substitution frequency at nonsynonymous and synonymous sites plotted by genes present in different numbers of species. The nonsynonymous substitution frequency (dN) values were plotted against the synonymous substitution frequency (dS) values for **(a) **R7 core genes and strain-variable open reading frames (ORFs) present in two to seven species and **(b) **core genes and strain-variable ORFs present in seven species that are longer than one kilobase in size.

The nonsynonymous substitution frequency (dN) ranged from 0.5 to 6.3 × 10^-2 ^substitutions per site for the R7 core genes (Figure 2a). The synonymous substitution frequency (dS) values were more than tenfold higher than the dN values in all pair-wise comparisons, which is indicative of purifying selection. In comparison, the dN values for the strain-variable ORFs were much more variable, ranging from 0.5 to 18 × 10^-2 ^nonsynymous substitutions per site (Figure 2a). Median dN values were inversely related to the prevalence of the ORF across the seven species and approached the dS values in some pair-wise comparisons that included only two or three species. A difference between the R7 core genes and the strain-variable ORFs in the seven-ortholog clusters was observed even if only ORFs that are more than 1 kilobase in size were included (Figure 2b). The smaller size and higher substitution frequency suggests that many strain-variable ORFs, particularly those present in a limited set of species, have evolved as pseudogenes.

### Graphical display of the gene fragmentation process

**Figure 3:**
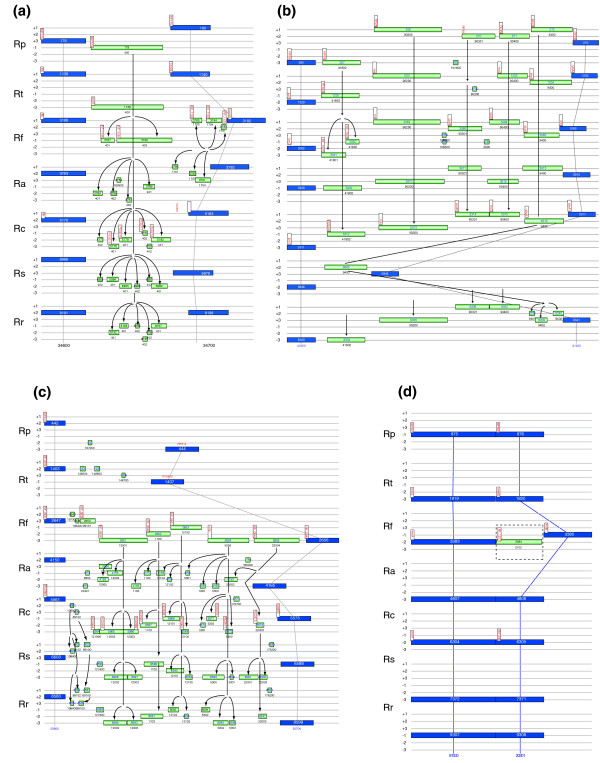
Visualization of variable segments using the GenComp visualization tool. Segments are shown that contain variable open reading frames (ORFs) that are present in **(a) **all seven species, **(b) **in most but not all species, **(c) **in members of the spotted fever group (SFG) *Rickettsia*, and **(d) **in a single species. The visualization tool displays (in blue) the location of positional orthologs that are conserved across all seven *Rickettsia *spp. and differ by less than 20% in size. Interspersed among these are segments with strain-variable ORFs shown (in green) that differ in sizes and are normally present in only a subset of the *Rickettsia *spp. Vertical lines show positional orthologs and horizontal lines indicate the six frames (+1, +2, +3, -1, -2, and -3, in that order). Each set of six lines represents a species, with *R. prowazekii *(Rp), *R. typhi *(Rt), *R. felis *(Rf), *R. akari *(Ra), *R. conorii *(Rc), *R. sibirica *(Rs), and *R. rickettsii *(Rr) shown from the top to the bottom, in accordance with their phylogenetic relationships. Numbers inside boxes show ORF numbers, and designations above boxes show gene annotations. The first digits in the numbers below the boxes indicate homologous strain-variable ORFs that are members of the same ORF-cluster, and the last two digits indicate ORFs in the ORF-clusters that differ by less than 80% in size. Arrows illustrate the fragmentation process for sequences that are similar across species.

With the aid of the visualization component of GenComp, we produced graphical images of the positions and relative sizes of all strain-variable ORFs in the 304 ORF-containing RVSs (Figure 3; see Additional date file 1 for graphical images of all RVSs). Note that many of the individual strain-variable ORFs represent fragments of the same pseudogene, broken up by indels and stop codons into multiple short ORFs. To follow the gene deterioration process in detail, we implemented a digital code to track sequence similarity across the strain-variable ORFs, with the first digits being identical for homologs within and across genomes and the latter two numbers representing a size index such that ORFs that are more than 80% similar in length are given the same number. A total of 482 of the 1,160 ORF-clusters produced significant hits (E < e^-10^) to genes in the National Center for Biotechnology Information (NCBI) database other than of the seven *Rickettsia *genomes used to identify the variable segments. We selected all *Rickettsia *ORFs for which multiple gene alignments with homologs in other species could be created for an in-depth analysis; this amounted to 469 of the 482 strain-variable ORF-clusters and 681 of the 688 R7 core genes.

To illustrate the fragmentation patterns, we sorted the strain-variable ORFs into four sets depending on the different species distribution profiles (Table 2). One set of 75 ORF-clusters contained homologs across all seven species, as exemplified in Figure 3a. Many strain-variable ORFs in this set were only weakly mutated (slightly shorter with only a few indels or internal termination codons compared with their full-length homologs in other species), suggesting that they may encode functional or semifunctional gene products in some species. Another set encompassed 31 strain-variable ORFs in 18 segments (Figure 3b), including 27 ORF-clusters present in the TG plus some but not all members of the SFG, as well as four ORF-clusters uniquely present in the TG. A third set of clusters included strain-variable ORFs that are present in members of the SFG but not in the TG (Figure 3c). This was the largest set, including 215 ORF-clusters, 76 of which have homologs in all members of the SFG. Another 28 strain-variable ORFs were solely present in *R. felis *and *R. akari*, and 26 ORFs only in *R. conori, R. sibirica*, and *R. rickettsii*. Visual inspection of the erosion patterns in this set provides many examples of how a long ORF in one species, typically *R. felis*, has been disrupted into numerous short sequence fragments in the other species. The final set included 148 strain-variable ORFs identified in only a single *Rickettsia *sp., 115 of which were solely present in *R. felis*; many of these encoded transposons and other mobile elements (Figure 3d).

**Table 2:** Classification of strain-variableORFs into species sets and phylogroups

Species profile	Phylogroup				
	
	ROW*	RO	R8	R7	Total
Set 1: all species					
1, 2, 3, 4, 5, 6, 7	29	15	30	1	75
Total	29	15	30	1	75
Set 2: TG + SFG					
1, 2	0	0	1	3	4
1, 2, 3	1	0	2	0	3
1, 2, 3, 4, 5	0	0	1	0	1
1, 2, 3, 4, 6	0	0	1	0	1
1, 2, 3, 4, 5, 6	0	0	1	0	1
1, 2, 3, 4, 5, 7	3	0	1	0	4
1, 2, 3, 5, 6, 7	0	1	1	0	2
1, 2, 3, 6, 7	0	0	1	0	1
1, 2, 4	0	1	0	0	1
1, 2, 4, 5, 6	0	0	1	0	1
1, 2, 4, 5, 6, 7	0	0	2	0	2
1, 3, 4, 5, 6, 7	4	1	1	0	6
1, 3, 5, 6, 7	0	0	1	0	1
1, 4, 5, 6, 7	0	0	1	0	1
2, 3, 4, 5, 6, 7	0	1	0	0	1
2, 3, 5, 6, 7	0	0	1	0	1
Total	8	4	16	3	31
Set 3: SFG only					
3, 4	2	4	19	3	28
3, 4, 5	0	0	3	0	3
3, 4, 5, 6, 7	16	6	53	1	76
3, 4, 5, 6	0	0	6	0	6
3, 4, 6	0	0	1	0	1
3, 4, 6, 7	0	0	1	0	1
3, 4, 7	0	0	1	0	1
3, 5	0	1	5	0	6
3, 5, 6	0	0	4	1	5
3, 5, 6, 7	4	2	17	2	25
3, 5, 7	0	0	3	0	3
3, 6	0	1	3	0	4
3, 6, 7	0	0	1	0	1
3, 7	0	0	2	2	4
4, 5	0	0	1	0	1
4, 5, 6	1	0	0	0	1
4, 5, 6, 7	1	1	3	0	5
5, 6	0	0	9	1	10
5, 6, 7	5	0	16	5	26
5, 7	0	0	3	2	5
6, 7	0	0	1	2	3
Total	29	15	152	19	215
Set 4: single species					
1	1	4	2	2	9
3	21	5	69	20	115
4	1	0	11	1	13
5	0	0	3	1	4
6	0	0	2	1	3
7	0	1	3	0	4
Total	23	10	90	25	148
Grand total	89	44	288	48	469

Number abbreviations for species are as follows: 1, *R. prowazekii*; 2, *R. typhi*; 3, *R. felis*; 4, *R. akari*; 5, *R. conorii*; 6, *R. sibirica*; and 7, *R. rickettsii*. The phylogroup ROW* is a summary of ROW, RW, W, and OW. ORF, open reading frame; SFG, spotted fever group; TG, typhus group.

### Strain-variable ORFs and core genes are associated with different phylogroups

**Figure 4:**
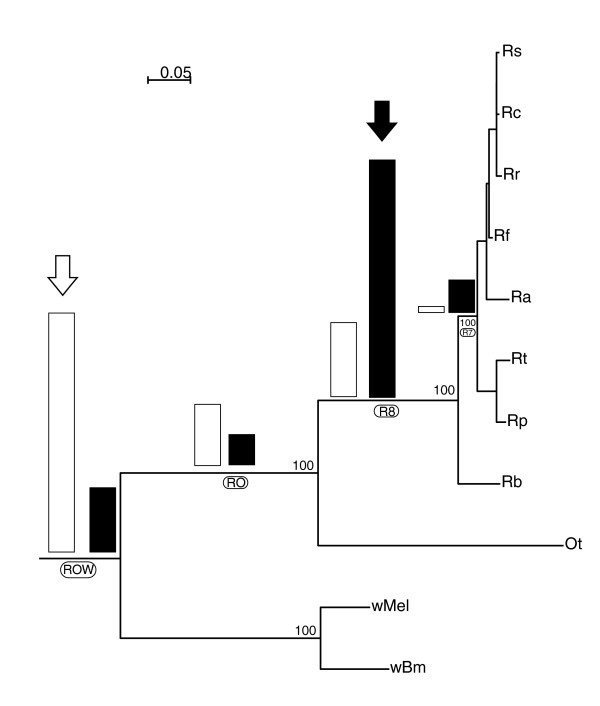
Number of R7 core genes and strain-variable ORFs placed at different nodes of the species tree. The relative proportions of R7 core genes (open boxes) and strain-variable open reading frames (ORFs; black boxes) are indicated at each node of the tree. Arrows show the nodes that contain the majority of core genes (ROW*) and strain-variable ORFs (R8). Species abbreviations are as in Figure 3, plus Ot (*O. tsutsugamushi*), wMel (*W. pipientis *[*Drosophila melanogaster*]), and wBm (*W. pipientis *[*Brugia malayia*]). The underlying phylogenetic tree was constructed using the maximum likelihood method from a concatenated alignment of adenylate kinase, SecY, and ribosomal proteins S3, S8, S10, S11, S13, S14, S19 and L2, L3, L4, L5, L6, L14, L16, L18, L22, L23, L24 and L29.

The strain-variable ORFs were then mapped onto the species phylogenetic tree to estimate the node at which it had been vertically inherited (Figure 4). To this end, core genes and strain-variable ORFs were separately grouped into different phylogroups. The R7 class contained ORFs without homologs in *R. bellii*, *O. tsutsugamushi*, or *W. pipientis*; the R8 class had homologs in *R. bellii *only; the RO class also in *O. tsutsugamushi*; and the ROW* class in *O. tsutsugamushi *and/or *W. pipientis *(E < e^-10^; see Additional data file 2). The results of this categorization revealed a dramatic difference between strain-variable ORFs and core genes, as summarized in Figure 4. Thus, 72% of the strain-variable ORFs were placed in the R7 and R8 classes, 9% in the RO class, and only 19% belonged to the ROW* class. The converse pattern was observed for the core genes; only 21% belonged to the R7 and R8 classes, and 62% traced back to the ROW* ancestor.

### Strain-variable ORFs in the R7 and R8 phylogroups are present in fewer *Rickettsia *spp. and are more degraded than strain-variable ORFs in the ROW* phylogroup

The extent of degradation was different for strain-variable ORFs placed at different nodes in the tree (Table 2). In brief, strain-variable ORFs in the ROW* class were often weakly mutated and normally present in all or most *Rickettsia *spp., whereas strain-variable ORFs in the R7 and R8 classes tended to be heavily degraded or only present in a single *Rickettsia *sp. For example, 32% of the 89 strain-variable ORFs in the ROW* class were present in all seven *Rickettsia *spp., whereas only one strain-variable ORF in the R7 phylogroup had homologs in all species. The RO class also contained a high proportion, 34%, of strain-variable ORFs with homologs in all *Rickettsia *spp., whereas strain-variable ORFs placed in the R8 phylogroup exhibited a more scattered species distribution pattern. Taken together, 71% of the strain-variable ORFs solely present in the SFG belonged to the R8 class, although they represent no more than 46% of the ORFs overall. The R7 class was even more biased in that 52% of the 48 strain-variable ORFs in the R7 phylogroup were members of a single species and another 40% were present solely in the SFG.

### Strain-variable ORFs in the R7 and R8 phylogroups are associated with different functional categories than strain-variable ORFs in the ROW* phylogroup

With the aid of a classification scheme based on clusters of orthologous groups of proteins (COGs)-based classification scheme, we analyzed the distribution of functional categories for genes and pseudogenes belonging to different phylogroups (Tables 3 and 4). The 89 strain-variable ORFs in the ROW* class exhibited a broad functional distribution profile according to COG classification (Table 3), much like the core genes inherited from the ROW* ancestor (Table 4). Both strain-variable ORFs and core genes in the ROW* phylogroup exhibited a relatively high abundance of genes in categories such as translation, replication, and energy production, as observed previously [17].

**Table 3:** Classification of strain-variableORFs into functional categories and phylogroups

Category	Phylogroup				
	
	ROW*	RO	R8	R7	Total
Information					
J Translation	6	3	2	1	12
K Transcription	2	5	5	2	14
L Replication	12	3	13	13	41
Total	20	11	20	16	67
Cellular processess					
D Cell cycle	0	0	3	1	4
V Defense mechanisms	2	2	16	3	23
T Signal transduction	0	0	3	0	3
M Cell wall/membrane	7	0	10	3	20
U Intracellular trafficking	2	1	7	0	10
O Post-translational modification	6	0	2	0	8
Total	17	3	41	7	68
Metabolism					
C Energy production	4	0	6	0	10
G Carbohydrate transport	0	0	4	1	5
E Amino acid transport	2	2	2	0	6
F Nucleotide transport	1	0	1	0	2
H Coenzyme transport	4	0	0	2	6
I Lipid transport	0	0	11	1	12
P Inorganic ion transport	5	1	3	0	9
Q Secondary metabolites	0	0	2	0	2
Total	16	3	29	4	52
Poorly characterized					
R General function	13	3	29	7	52
S Function unknown	11	4	30	6	51
Unclassified	12	20	139	8	179
Total	36	27	198	21	282
Grand total	89	44	288	48	469

The phylogroup ROW* is a summary of ROW, RW, W and OW. ORF, open reading frame

**Table 4:** Classification of core genes into functional categories and phylogroups

Category	Phylogroup				
	
	ROW*	RO	R8	R7	Total
Information					
J Translation	68	9	8	0	85
K Transcription	13	2	4	0	19
L Replication	38	2	2	0	42
Total	119	13	14	0	146
Cellular processess					
D Cell cycle	9	3	0	0	12
V Defense mechanisms	4	1	1	0	6
T Signal transduction	5	1	3	0	9
M Cell wall/membrane	21	6	32	0	59
N Cell motility	0	0	1	0	1
U Intracellular trafficking	26	4	0	0	30
O Post-translational modification	32	7	3	0	42
Total	97	22	40	0	159
Metabolism					
C Energy production	45	9	5	0	59
G Carbohydrate transport	6	1	0	0	7
E Amino acid transport	13	6	1	0	20
F Nucleotide transport	9	0	1	0	10
H Coenzyme transport	14	2	2	0	18
I Lipid transport	18	1	3	0	22
P Inorganic ion transport	8	4	2	0	14
Q Secondary metabolites	3	1	2	0	6
Total	116	24	16	0	156
Poorly characterized					
R General function	24	8	10	0	42
S Function unknown	12	6	13	1	32
Unclassified	60	31	53	2	146
Total	96	45	76	3	220
Grand total	428	104	146	3	681

The phylogroup ROW* is a summary of ROW, RW, W and OW.

The R7 and R8 phylogroups showed strong functional bias. Interestingly, the large majority of the strain-variable ORFs (69%) and the core genes (54%) in the R8 phylogroup represented unknown or poorly characterized genes. Although these hypothetical ORFs may include gene prediction errors, a similar fraction of unknowns was observed in the core genes, suggesting that they represent current or ancestral genes of unknown function. Three functional categories - 'cell wall biosynthesis', 'replication', and 'defense mechanisms' - dominated among the rest of ORFs in these phylogroups. For example, 32 of the 146 core genes in the R8 class represent components of the cell wall that were possibly shed from *Orientia *and *Wolbachia *[17]. A total of 20 strain-variable ORFs belonged to this category, ten of which were placed in the R8 and three in the R7 phylogroup. These include genes for lipopolysaccharide biosynthesis, which may have been acquired at the base of the *Rickettsia *lineage.

### Strain-variable ORFs in the R7 and R8 phylogroups contain an over-representation of mobile genetic elements

The large majority of strain-variable ORFs in the R7 and R8 classes represents mobile genetic elements and their associated genes, many of which were classified into the COG categories 'replication and repair' or 'defence mechanisms'. Altogether, these two categories contained 44 strain-variable ORFs, which account for 30% to 50% of strain-variable ORFs with a COG-based functional assignment in the R7 and R8 classes, respectively. These genes include transposases, phage genes, plasmid genes and genes for DNA helicases, RNA helicases, and different types of DNA restriction-modification enzymes. Many more mobile genes were identified in the *Rickettsia *genomes, but were either unclassified or represented poorly characterized genes, or were categorized in the RO or ROW* classes because of the presence of distant homologs in *O. tsutsugamushi *and *W. pipientis*. The latter include numerous strain-variable ORFs for ankyrin repeat and TPR repeat proteins that are normally associated with conjugative transfer elements in *O. tsutsugamushi *[8] and phage genes in *W. pipientis *[14].

To search systematically for remnants of mobile genetic elements, we extracted already identified phage and conjugative transfer genes in the individual genomes and BLASTed these against all of the other genomes. Genes for conjugative transfer encoded by the *tra *operon have been identified on the *R. felis *plasmid as well as on the chromosomes of *R. felis*, *R. bellii*, and *O. tsutsugamushi*. Using the *tra *operon as the query, we analyzed each genome for remnants of such genes. No evidence of conjugative transfer genes or remnants of such genes was observed in any of the other *Rickettsia *genomes nor in *W. pipientis*. The explanation may be that the *tra *gene cluster has been horizontally transmitted into or across *R. felis*, *R. belli*, and *O. tsutsugamushi*. Indeed, a phylogenetic analysis of the *tra *genes present in *R. bellii*, *R. felis*, and *O. tsutsugamushi *showed that the order of divergence was different from the species divergence pattern (data not shown), as was also observed in a recent survey of the *Rickettsia massiliae *genome [25].

We also observed phage-related genes of the HK97 family in a few RVS with multiple strain-variable ORFs. For example, flanking a gene encoding a HK97 phage portal protein in RVS-308 were five duplicated genes putatively encoding cell surface antigens in *R. felis*. In RVS-552 we identified a long stretch of 13 ORFs in *R. felis*, all of which have homologs in *R. bellii *and six of which (including a copy of the gene for the HK97 phage major capsid protein) have homologs also in *O. tsutsugamushi *and *W. pipientis*. Interestingly, not only the sequence but also the order of genes was preserved. None of these six genes, or remnants thereof, was present in any of the other *Rickettsia *spp. Although no intact prophage was found, the identification of phage genes in the variable *Rickettsia *segments suggests that bacteriophages are circulating in the *Rickettsia *population.

Finally, we examined in greater detail the 48 strain-variable ORFs identified in the R7 class, which represents putative horizontal gene transfers into individual *Rickettsia *spp. and clades, although it cannot be excluded that they were acquired at the base of the *Rickettsia *lineage followed by species-specific losses. These include a long stretch of genes in RVS-422 that were solely identified in *R. prowazekii *and encode resolvase-like proteins, transposases, and ankyrin-repeat proteins. Also present in the TG were multiple genes encoding proteins with glycosyltransferase domains in RVS-626, one of which represents the 5' half of a longer gene with two such domains in the other *Rickettsia *spp. None of these have a close homolog in the Rickettsiales, but were related to glycosyltransferases in distantly related species such as *Geobacter *spp. and *Vibrio cholerae*. The genes RP336/RT0325 and RP337/RT0326 have adjacent homologs in *V. cholerae*.

The SFG and transitional group clades shared gene remnants for aminoglucoside phosphotransferase and acyltransferases. In addition, these clades had unique strain-variable ORFs associated with mobile elements, which encoded products such as lyase, DNA-damage inducible proteins, type I restriction-modification enzyme, transposase, and plasmid stabilization proteins. Twenty strain-variable ORFs in the R7 group were only present in *R. felis*; these included an exochitinase, a biotin synthase gene *bioB*, and mobile elements such as a mutator-type transposase (similarity to *Psychrobacter*), which was located in six different RVS fragments. The only strain-variable ORF with remnants in all species was the *metK *gene, which encodes S-adenosylmethionine synthetase.

### Strain-variable ORFs and core genes in the R7 and R8 phylogroups have different proportions of closest proteobacterial relatives

To investigate how the differences in functionality relate to the putative source of the sequences in the R7 and R8 phylogroups, we inferred the closest relatives from the taxonomic classification of the most similar sequences, excluding *Rickettsia*, *Orientia*, and *Wolbachia *(Table 5). The R7 class was dominated by proteobacteria-like sequences, with 17 out of 35 ORFs showing similarity to γ-proteobacteria. Overall, the proteobacteria-like sequences accounted for 73% of strain-variable ORFs in the R7 class, and 11% to 12% of strain-variable ORFs and core genes in the R8 phylogroups. The lower proportion of proteobacteria-like sequences in the R8 classes is due to the high numbers of strain-variable ORFs (60%) and core genes (38%) that are specific to *Rickettsia *with no identifiable homolog outside the genus. A total of 80 strain-variable ORFs and 80 core genes in the R8 class exhibited sequence similarities to proteobacteria. However, the ratio of ORFs with the highest sequence similarity of α-proteobacteria versus γ-proteobacteria was strikingly different: only 0.6 for the strain-variable ORFs versus 3.2 for the core genes.

**Table 5:** Sequence similarity to bacterial subdivisions

	R7	R8	
	
	Strain variable	Strain variable	Core
R8-specific	-	173	56
Proteobacteria			
α	7	21	52
β	7	14	6
δ	3	10	4
ε	1	1	2
γ	17	34	16
Actinobacteria	1	2	1
Aquificae	0	0	3
Bacilli	3	5	0
Bacteroidetes	1	8	1
Chlamydia	0	2	0
Clostridia	1	2	1
Cyanobacteria	2	6	1
Deinococcus	1	1	0
Fibrobacter	1	1	1
Fusobacteria	0	0	1
Mollicutes	1	3	0
Spirochaetes	2	1	1
Euryarchaeota	0	4	0
Total	48	288	146

The α-proteobacteria do not include *Rickettsia*, *Orientia*, and *Wolbachia *spp.

## Discussion

To study the pattern of gene degradation, we developed a program for the identification and visualization of positional pseudogenes in multiple, related species. Previously developed software for the identification of strain-variable regions such as tRNAcc [26] and Islander [27] are based on high-throughput systematic interrogation of tRNA and transfer mRNA genes, which act as hotspots for insertions of temperate phages and pathogenicity islands [28]. The tRNAcc approach identified 49 genomic islands in the vicinity of 18 tRNA genes in *Escherichia coli *and *Shigella*, representing as much as 1.7 megabases of these genomes [26]. Islander was applied to the analysis of 106 bacterial genomes of different phylogenetic affiliation, with 95% of the islands identified in Firmicutes, and α- and γ-proteobacteria [27]. However, Islander failed to identify strain-variable regions in about half of the genomes examined, including all obligate pathogens and endosymbionts.

Yet another program for the identification of variable segments is IslandPath, which searches for horizontally acquired DNA by profiling GC contents and dinucleotide composition patterns of individual genes and clusters of genes [29]. However, because variability may not only be caused by gene acquisitions, but also by species-specific gene degradation processes, strain-variable segments will go unrecognized if nucleotide and codon usage statistics of the gene remnants temporarily appear normal. Thus, programs such as tRNAcc [26], Islander [27], and IslandPath [29] are not useful for the analysis of the genomes of intracellular bacteria in which the deteriorating ORFs are neither flanked by integrases or tRNAs nor exhibit atypical GC-content statistics.

The application of our program, GenComp, to a visualization study of eight complete genome sequences of *Rickettsia *has unveiled a mosaic of conserved core genes for basic cellular functions interspersed with segments containing complete and deteriorating genes that are variably present across species. The novelty of the findings reported here is that as much as 75% of the variable genes and pseudogenes in *Rickettsia *have no homologs in either *O. tsutsugamushi *or *W. pipientis*. Many of these are extensively degraded or represent mobile genetic elements and their associated genes. Although some genes such as those for cell wall biosynthesis may have been lost from the outgroup species, we believe that a majority of the variably present and deteriorating genes entered at the base of the *Rickettsia *lineage. A smaller subset seems to be circulating across some of the modern *Rickettsia *spp. in a manner that is inconsistent with vertical inheritance. Collectively, the results suggest that a substantial fraction of the variability in gene content and extent of deterioration is accounted for by horizontal gene transfers into the genus *Rickettsia*.

In total, we identified 688 R7 core genes and 1,160 ORF-clusters, with 469 of the ORF-clusters containing easily recognizable homologs in species outside the R7-class. Although the aim of this analysis was not to quantify the total number of ancestral genes in *Rickettsia*, our minimal and maximum estimates of 1,157 and 1,848 ORF-clusters in the rickettsial ancestor, respectively, are consistent with previous inferences of 1,252 to 1,650 ancestral genes [30]. Our visualization analysis has shown that the fragmentation process often involves segments with multiple pseudogenes, in accordance with the suggestion that lost genes are clustered more frequently than expected by chance [30]. With only a few exceptions, we found no tendency for genes with similar functions to be clustered in the same segment. Rather, the deletions appear to cover blocks of genes in some species whereas in others they are best explained by independent small deletions in neighbouring genes, putatively acquired by horizontal gene transfer.

Another observation is that the rate of evolution is related to the species distribution patterns such that genes present in fewer *Rickettsia *spp. tend to accumulate more substitutions than those present in more species. One explanation for a faster rate of evolution for horizontally transferred genes is positive selection and adaptation [31]. However, because we observed a correlation between limited species distribution patterns, high substitution frequencies, and small ORF sizes, we believe that most of the enhancement in the rate of evolution is due to degenerative processes.

For many of the heavily deteriorating genes, remnants were identified in the SFG *Rickettsia *but not in the TG *Rickettsia*. The presence of homologs in *R. bellii *suggests that these genes were acquired at the base of the *Rickettsia*, with subsequent loss in the TG *Rickettsia*. Hence, it is tempting to speculate that some of the gene acquisitions at the early stage of rickettsial evolution conferred functions that facilitated invasion and spread into novel arthropod hosts, or into multiple tissues of an already infected host. Upon subsequent host switches and/or niche adaptation, this early set of acquired genes may have become superfluous, leading to gene degradation and elimination. This is consistent with the observation that species with a restricted host range, such as *R. prowazekii*, exhibit extensive gene loss. Among the few genes present in the variable segments of the TG but not the SFG *Rickettsia *are duplicated genes for glucosyltransferases and enzymes involved in lipopolysaccharide biosynthesis.

Some of the variably present genes, mostly mobile elements such as transposons, conjugative transfer elements, and their associated genes, may represent recent gene transfers into individual lineages. The insertion of these at unique locations in the genome with no indications of remnants in any of the other species supports a recent integration rather than acquisition at the base of the lineage and loss in all other species.

The bias for loss of recently gained genes in *Rickettsia *is consistent with computational inferences of insertion/deletion rates based on gene presence/absence data in other bacterial species [32,33]. For example, a study of 13 completely sequenced genomes from *Bacillus *showed that there are more genes coming in and going out at the tips of the phylogeny than at the deeper nodes, suggesting that most of the laterally transferred genes are lost shortly after their insertion [33]. In our study, genes of unknown or general function prediction was found to be lost more frequently than expected by chance alone. A trivial explanation for the apparent high turnover rate of genes of unknown function at the tips of the tree may be false gene predictions. This is almost certainly one aspect of the problem; the visualization profiles of the *Rickettsia *genomes confirm that many short ORFs located in immediate proximity to each other (previously annotated as different genes) represent short fragments of one and the same gene. For example, the annotated *R. conorii *genes RC0215, RC0216, RC0217, and RC0218 are short fragments of the longer positional homolog RP174 in *R. prowazekii *(see Additional data file 1; Reg_id 5). However, false gene predictions cannot be the sole explanation because a more rapid deterioration of genes acquired at the base of the *Rickettsia *lineage was observed even if counting ORF-clusters with homologs to species outside the R7 class instead of individual ORFs. Thus, our results from *Rickettsia *suggest a low residence time for horizontally transferred genes of yet unknown function.

Gene acquisition depends on the availability of mobile elements that can mediate the transfers, whereas the probability for retention is determined by the mutation bias along with selection and drift. Therefore, the transfer-deterioration process is expected be particularly high in bacterial species that contain plasmids or are exposed to bacteriophages. Recent gene acquisitions in *Rickettsia *appear to have been mediated by plasmids, discovered in *R. felis*, *R. monocensis *[34], and several additional *Rickettsia *spp. isolated from ticks [35]. Species that lack plasmids (for example, *R. prowazekii*) exhibit a much lower incidence of recent gene acquisitions than the plasmid-bearing species *R. felis*.

Previous studies of the *R. felis *plasmid genes revealed that 38 of the 68 pRF plasmid genes have no chromosomal homologs and are not present in the SFG *Rickettsia*, although 18 of these show homology to other bacterial proteins [16]. Plasmid genes with chromosomal homologs show mostly an evolutionary relationship with earlier diverging species, such as *R. bellii*, although the chromosomal homolog may support the expected relationships with other *Rickettsia *spp. [16]. Our phylogenetic analysis of the *tra *genes is fully consistent with this pattern, by showing that the conjugative system on the *R. felis *plasmid diverged earlier than the chromosomally encoded *tra *genes in *R. bellii *and *O. tsutsugamushi*. This was also observed in a recent phylogenetic analysis of the *tra *cluster genes [25]. Traces of the *tra *genes could not be identified in the other five *Rickettsia *spp. And neither in *W. pipientis*.

Although some species of *Wolbachia *and *Rickettsia *infect the same arthropod host, these two genera do not share the same mobile gene pool; plasmids are the vehicle of choice in *Rickettsia *whereas bacteriophages dominate in *Wolbachia*. Thus, in contrast to free-living micro-organisms such as *Escherichia coli *[36], species-specific ORFs are not derived from bacteriophages. However, the co-transferred genes encode proteins in the same broad families, such as for example ankyrin and TPR repeat proteins. The taxonomic distribution of species containing the most similar sequences outside the Rickettsiales was different for core genes and strain-variable ORFs, with a higher fraction of non-α-proteobacterial relatives for strain-variable ORFs. However, the phylogenetic analyses indicated fairly distant evolutionary relationship (data not shown), which might suggest transfers via yet unsequenced bacteria and their plasmids.

Taken together, the findings of our analysis suggest that the likelihood for a rickettsial gene that does not have homologs in *Wolbachia *or *Orientia *to be degraded is at least three times as high as that for a gene that has been vertically inherited since the divergence of the three genera. Our estimate of deteriorating horizontal transfers in *Rickettsia *corresponds well with a global 'failed horizontal transfer index' of 2.3, which means that 'pseudogenes' are 2.3 times more likely to arise from horizontal transfer than vertically inherited core genes [1]. However, at the detailed level there are large inconsistencies between the two studies. Whereas we identified hundreds of pseudogenes and gene fragments in the *R. conorii *genome using GenComp, only nine were detected by the prokaryotic pseudogene pipeline, none of which was inferred to have been acquired by horizontal gene transfer [1]. The discrepancy in gene numbers and origin of acquisition suggests that comparative genomics methods are superior for the identification and analyses of pseudogenes and gene fragments.

The analysis presented here also explains the puzzling observation that the median size of *R. conorii *proteins is only 173 amino acids, thereby the shortest of bacterial proteins, as compared with a median size of 267 amino acids estimated from 191,541 bacterial proteins [37]. We have estimated the median size of the R7 core proteins in *R. conorii *to be 284 amino acids, which is equal to the median size of *R. prowazekii *proteins and slightly higher than the global bacterial protein size estimate [37]. Thus, the previously estimated short size of the *R. conorii *proteins is that it was markedly influenced by the short sizes of the many pseudogenes present in this genome. The overall variability in protein size among bacterial species has been attributed to different adaptations to stress, temperature, protection, and other environmental factors [37]. A simpler explanation is different proportions of pseudogenes and gene fragments (of different sizes) in the genome annotation lists. Because 2,300 of an estimated 6,895 candidate pseudogenes in prokaryotic genomes overlap with more than 2,600 annotated hypothetical ORFs [1], attempts to determine mean and median bacterial protein sizes from genome sequence data are likely to yield underestimates, unless the annotated gene lists have first been decontaminated for pseudogenes.

## Conclusion

We have presented a visualization tool for the comparison of pseudogenes in multiple closely related species. The tool assigns a number to each individual ORF to indicate its relative size and the ORF-cluster to which it belongs. The application of this tool to the analyses of the variably present genes and pseudogenes in *Rickettsia *has implications for our understanding of genome reduction in intracellular bacteria. Variably present ORFs account for 30% to 40% of the rickettsial genomes and a majority of these represents mobile genetic elements and associated genes acquired by horizontal gene transfers. This explains the paradox of the high pseudogene content despite the small sizes of the *Rickettsia *genomes.

Our visualization of the rickettsial pseudogenes has highlighted one of the major hurdles in gene prediction, namely how to automate pseudogene identification and distinguish genes from spurious ORFs [38-43]. Over the next ten years, thousands of bacterial pathogens will have their genome sequenced and hundreds or thousands of isolates from these species will routinely be examined. The number of species-specific genes and pseudogenes will increase with every genome added, perhaps even exponentially for populations with open pan-genome structures. Understanding their origin, function, and survival rate is an important task for future research. This creates a need for better ways to compare and visualize the genomic data from closely related species. We are convinced that the GenComp software and other similar programs will demonstrate their utility in future pan-genomic surveys.

## Materials and methods

### Genome sequence data

Genome sequence data were downloaded from NCBI, including genome data for *R. prowazekii *[4], *R. typhi *[10], *R. conorii *[11], *R. felis *[12], *R. belli *[13], *R. sibirica *(AC = NZ_AABW00000000), *R. akari *(AC = NC_00981), and *R. rickettsii *(AC = NC_00982).

### Positional matching of genes and strain-variable ORFs

Gene prediction of the *Rickettsia *genomes was accomplished using Glimmer2 [22]. The ORF sequences were extracted, translated, and BLASTed against each other in all possible combinations using BLASTP [23]. The resulting BLAST file output, which contains true orthologs, paralogs and false hits, was fed into Tribe-MCL to cluster all homologs into gene families [24]. These were resolved semi-automatically into orthologous gene clusters by a series of steps, as outlined below.

We first applied a simple ratio-rating test to the Tribe-MCL gene families, in which ORFs were partitioned by size. The ORF length ratio was set to 80% in pair-wise genome comparisons. In analyses that included multiple genomes, we measured the length ratios in all-against-all comparisons within ORF families and produced a ratio-rating matrix. The gene families were sorted into subgroups by partitioning the matrix recursively according to length ratio similarities. Next, the neighbor-joining method was applied to the dataset to resolve the gene families further. It is important to recall that both operations retain the same total number of ORFs, but that these are resolved into an increasing number of gene families, each of which contains ORFs of increasingly similar gene sizes and sequences. Finally, we manually inspected the obtained gene order information using the graphical viewer (see below) to distinguish true orthologs from in-paralogs. The gene sequences in the metaclusters as well as the sequences in the intervening segments were automatically extracted for analysis of substitution frequencies and other statistics.

### Sequence and phylogenetic analysis

Pair-wise sequence alignments of positional orthologs and positional pseudogenes were done using ClustalW [44] from protein sequences that were reconverted to in-frame DNA alignments. Substitution frequency estimates were obtained by the method of Yang and Nielsen in PAML [45]. The sequences of the intervening segments, including strain-variable ORFs, were searched for sequence similarity using tblastx, version 2.2.8 [23] against a nonredundant bacterial genome database (March 2005) downloaded from the NCBI bacterial genome ftp site [46]. The best hit (BeTs; E < 10^-10^) was recorded for each segment and in segments with multiple ORFs for each ORF individually. A reference set of sequences that yielded top hits to species other than the α-proteobacteria were aligned with all of their homologous genes (E < 10^-10^) using ClustalW [44] (gap-opening penalty: 10; gap extension penality: 0.05) and protein distance estimates were recorded. Phylogenetic trees were computed using the maximum likelihood method implemented in PHYML [47].

#### Implementation

The GenComp software was crafted to enable systematic comparisons of multiple genomes on a genome scale from an evolutionary point of view. The software was based on a MySQL database server and the main scripting language was Python and Perl with biopython and bioperl libraries. The interface was implemented in Tkinter, which uses Python as a gluing language to Tkinter and to the database. The main virtue of having all information in a DBMS (database management system) is the ease of access to all data and the ability for crosstalk across various sources of data. Annotated genomes in the form of sequences and annotations from NCBI or in multifasta format were stored in the database and used for reference to other scripts for computing. GenComp uses a number of different scripts and common programs such as Glimmer, BLAST, Tribe-MCL, ClustalW, PAML YN00, PHYLIP, and scripts developed in-house to process the data. The entire genomes under study are fed into the system and then semi-automatically processed, guided by the user. Data and tables are redrawn from the system by scripts written in SQL and Python, sometimes interchangeably to call the database guided by previous analysis and result. The final result of an analysis session is a table exported to Excel for further processing or graphing. Sometimes external graphing software is used, such as Origin Pro or Gnuplot.

### Graphical viewer

We developed a graphical viewer to visualize the order, sizes, and relationships of ORFs in the intervening segments. Positional orthologs were indicated with lines that span across all ORFs in each orthologous cluster. The gene names for orthologous clusters and for ORFs in the intervening segments were shown in the graphical viewer if annotations were available in the annotation file from NCBI. ORFs in the intervening segments were indexed to indicate gene family according to Tribe-MCL in cases in which ORFs were located at the homologous position but varied in size as expected, for example positional pseudogenes. The index is split into two parts, in which the first part indicates gene family and the second part length ratio. The scripts developed in-house to process and visualize the data are available from the GenComp website [48].

## Abbreviations

bp, base pairs; BLAST, basic local alignment search tool; COG, clusters of orthologous groups of proteins; dN, nonsynonymous substitution frequency; dS, synonymous substitution frequency; ORF, open reading frame; RVS, *Rickettsia *variable segment; SFG, spotted fever group; TG, typhus group.

## Authors' contributions

H-HF developed GenComp and performed all the analyses presented in the paper, including the graphical display of the gene fragmentation process, estimates of substitution frequencies, phylogroup assignments, functional classification, and database searches. ACD supervised the phylogenetic study of the *tra *genes and the search for mobile genetic elements. N-HC provided the *O. tsutsugamushi *genome sequence before publication and helped in interpreting the status of genes for outer membrane proteins. SGEA designed the study, analyzed and interpreted the data, and wrote the paper with contributions from H-HF.

## Additional data files

The following additional data are available with the online version of the paper. Additional data file 1 presents the visualization of all variable segments containing ORFs in the seven *Rickettsia *genomes using GenComp. Additional data file 2 presents the distribution of individual strain-variable ORFs into species sets, phylogroups, and functional categories, and shows the most similar sequence outside the Rickettsiales.

## Supplementary Material

Additional data file 1 Presented is the visualization of all variable segments containing ORFs in the seven *Rickettsia *genomes using GenComp. Click here for file

Additional data file 2 Presented is the distribution of individual strain-variable ORFs into species sets, phylogroups, and functional categories. Also shown is the most similar sequence outside the Rickettsiales. Click here for file
