# Supplementary material for: Visualization of pseudogenes in intracellular bacteria reveals the different tracks to gene destruction
Source: Genome Biol. 2008 Feb 26;9(2):R42. doi: 10.1186/gb-2008-9-2-r42 (PMC2374718; doi:10.1186/gb-2008-9-2-r42)

1 Rickettsia prowazekii str. Madrid E, complete genome
2 Rickettsia typhi str. wilmington, complete genome
3 Rickettsia felis URRWXCal2, complete genome
4 Rickettsia akari str. Hartford chromosome, whole genome shotgun sequence
5 Rickettsia conorii str. Malish 7, complete genome
6 Rickettsia sibirica 246 rsib_agncrt, whole genome shotgun sequence
7 Rickettsia rickettsii chromosome, whole genome shotgun sequence

Reg_id: 4

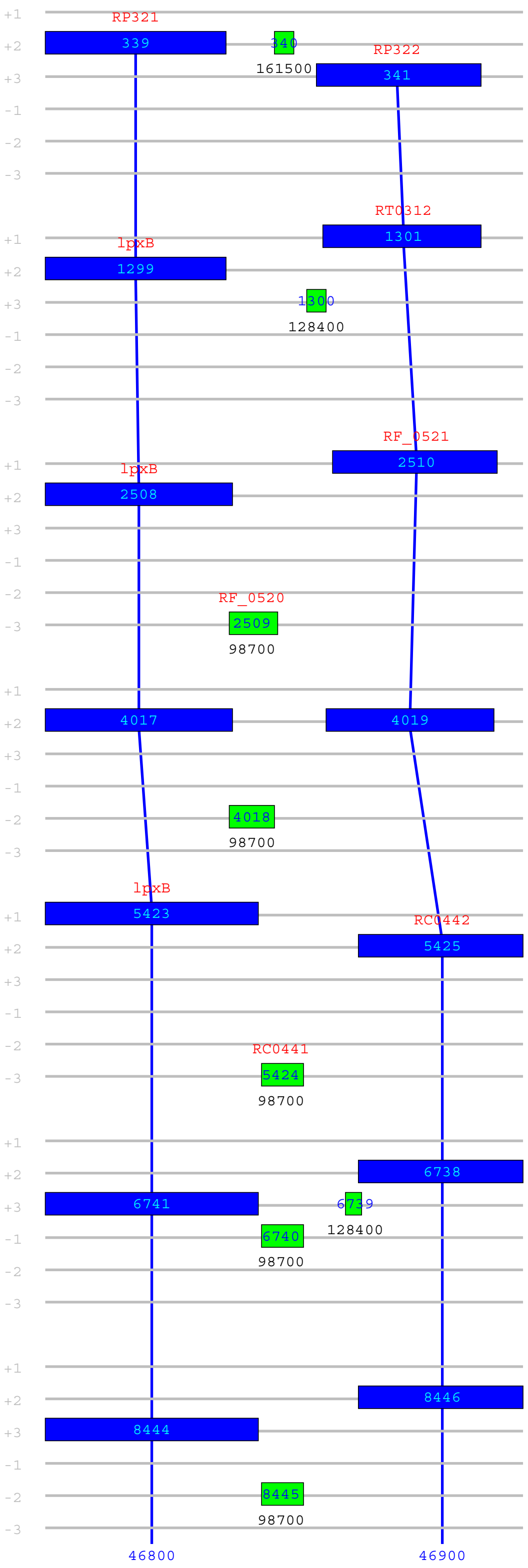

1 Rickettsia prowazekii str. Madrid E, complete genome
2 Rickettsia typhi str. wilmington, complete genome
3 Rickettsia felis URRWXCal2, complete genome
4 Rickettsia akari str. Hartford chromosome, whole genome shotgun sequence
5 Rickettsia conorii str. Malish 7, complete genome
6 Rickettsia sibirica 246 rsib_agncrt, whole genome shotgun sequence
7 Rickettsia rickettsii chromosome, whole genome shotgun sequence

Reg_id: 5

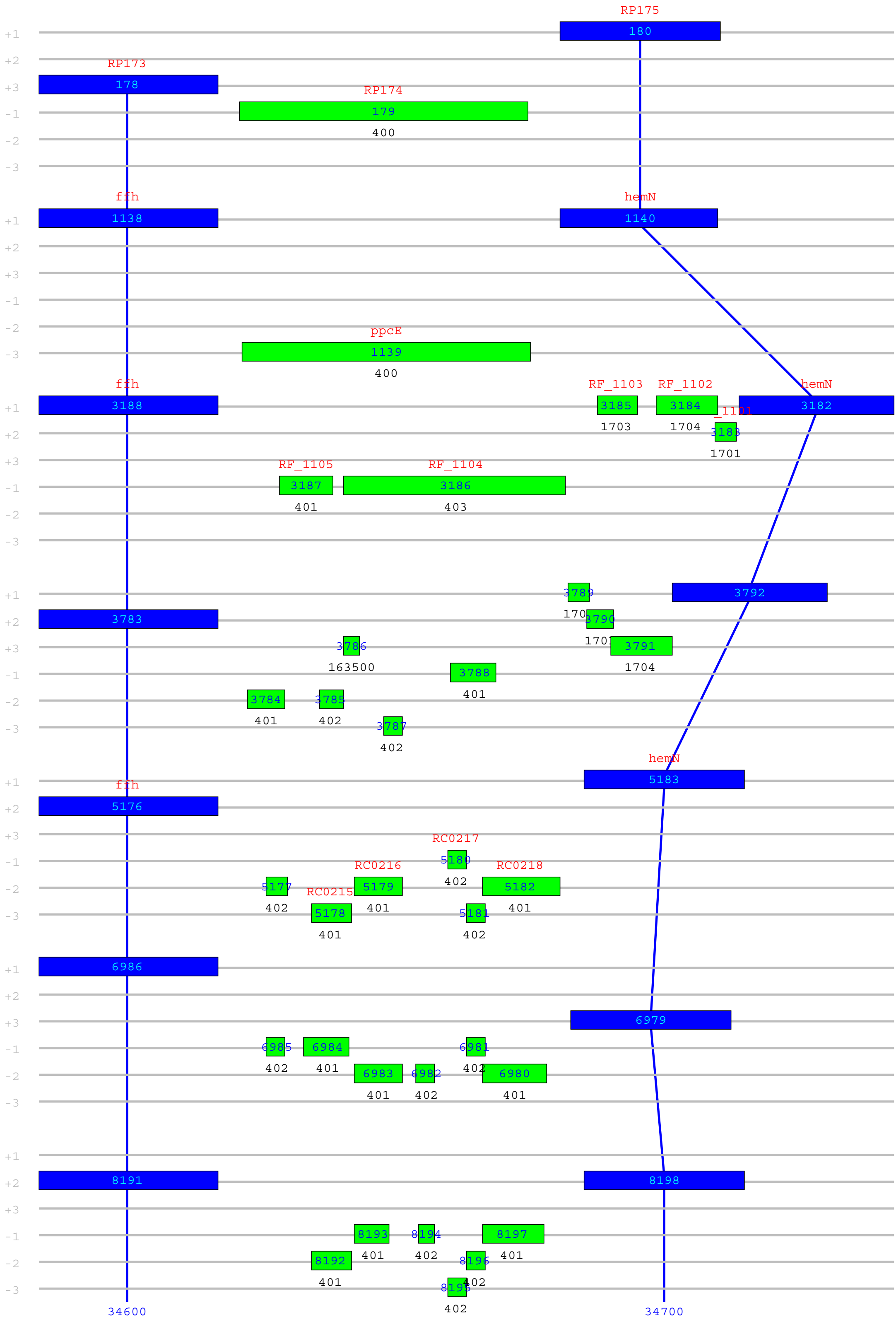

1 Rickettsia prowazekii str. Madrid E, complete genome
2 Rickettsia typhi str. wilmington, complete genome
3 Rickettsia felis URRWXCal2, complete genome
4 Rickettsia akari str. Hartford chromosome, whole genome shotgun sequence
5 Rickettsia conorii str. Malish 7, complete genome
6 Rickettsia sibirica 246 rsib_agncrt, whole genome shotgun sequence
7 Rickettsia rickettsii chromosome, whole genome shotgun sequence

Reg_id: 6

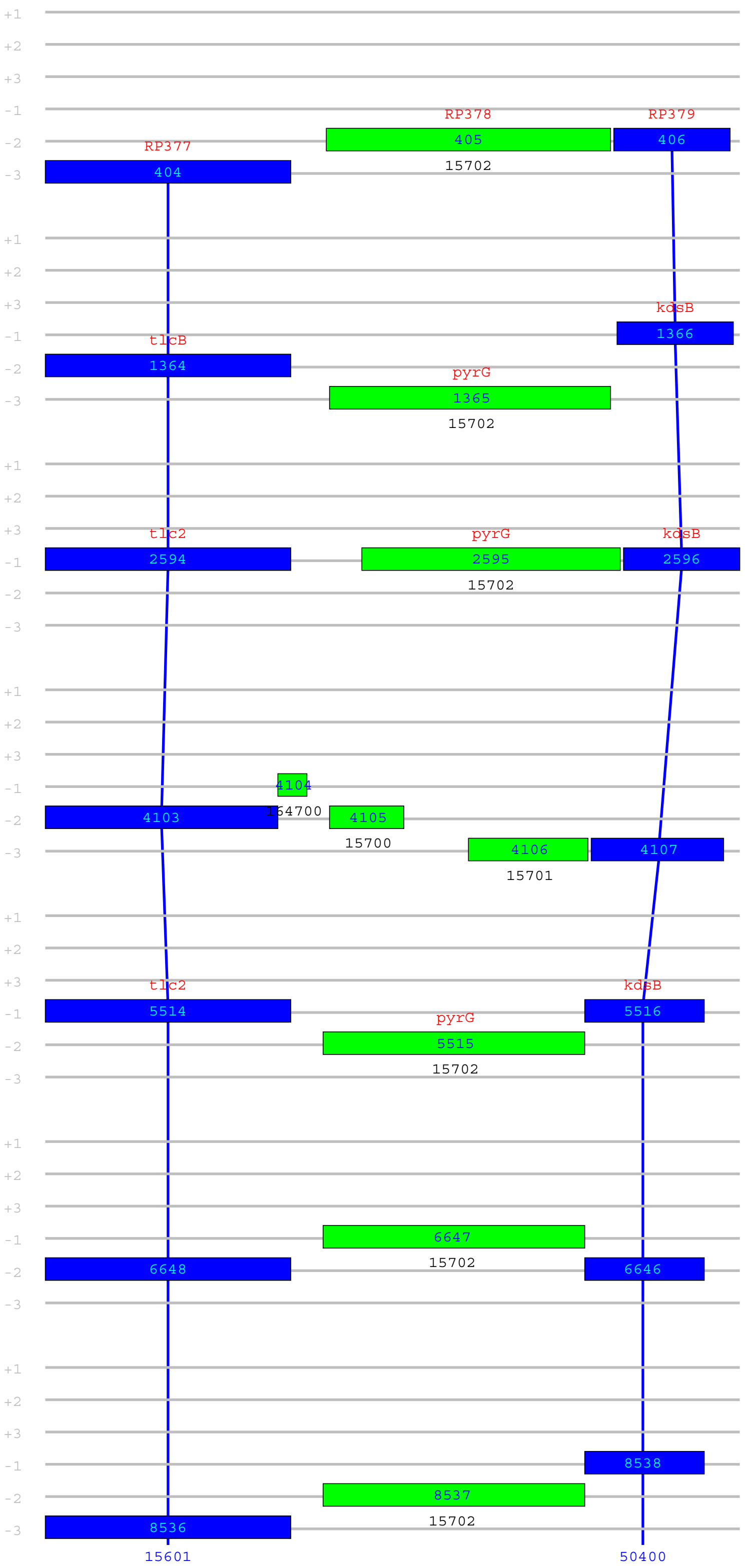

1 Rickettsia prowazekii str. Madrid E, complete genome
2 Rickettsia typhi str. wilmington, complete genome
3 Rickettsia felis URRWXCal2, complete genome
4 Rickettsia akari str. Hartford chromosome, whole genome shotgun sequence
5 Rickettsia conorii str. Malish 7, complete genome
6 Rickettsia sibirica 246 rsib_agncrt, whole genome shotgun sequence
7 Rickettsia rickettsii chromosome, whole genome shotgun sequence

Reg_id: 7

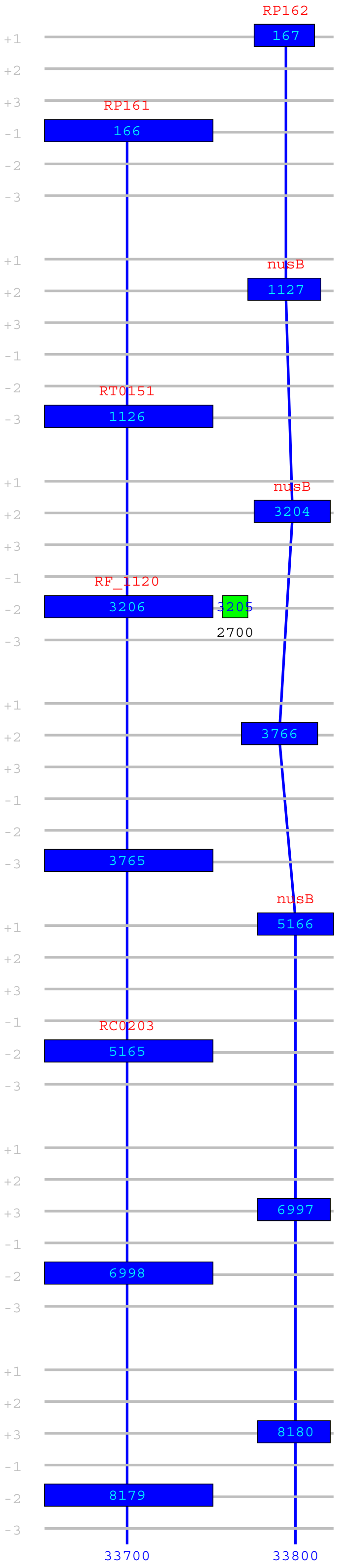

1 Rickettsia prowazekii str. Madrid E, complete genome
2 Rickettsia typhi str. wilmington, complete genome
3 Rickettsia felis URRWXCal2, complete genome
4 Rickettsia akari str. Hartford chromosome, whole genome shotgun sequence
5 Rickettsia conorii str. Malish 7, complete genome
6 Rickettsia sibirica 246 rsib_agncrt, whole genome shotgun sequence
7 Rickettsia rickettsii chromosome, whole genome shotgun sequence

Reg_id: 9

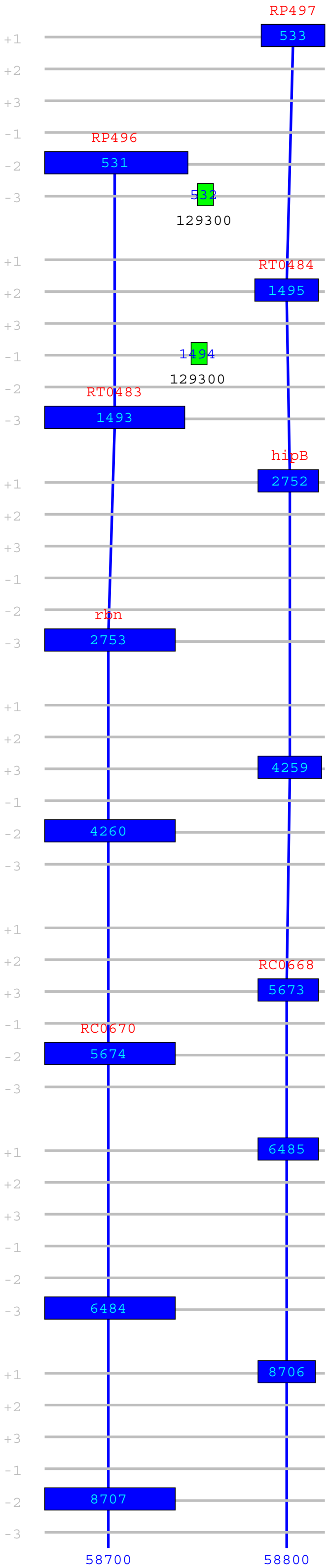

1 Rickettsia prowazekii str. Madrid E, complete genome
2 Rickettsia typhi str. wilmington, complete genome
3 Rickettsia felis URRWXCal2, complete genome
4 Rickettsia akari str. Hartford chromosome, whole genome shotgun sequence
5 Rickettsia conorii str. Malish 7, complete genome
6 Rickettsia sibirica 246 rsib_agncrt, whole genome shotgun sequence
7 Rickettsia rickettsii chromosome, whole genome shotgun sequence

Reg_id: 11

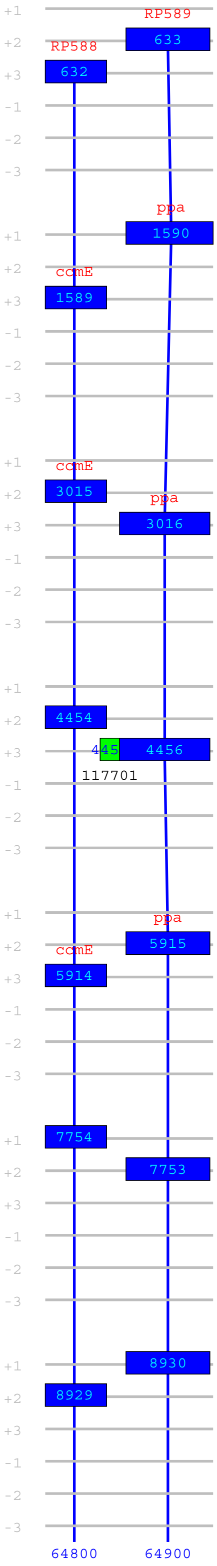

1 Rickettsia prowazekii str. Madrid E, complete genome
2 Rickettsia typhi str. wilmington, complete genome
3 Rickettsia felis URRWXCal2, complete genome
4 Rickettsia akari str. Hartford chromosome, whole genome shotgun sequence
5 Rickettsia conorii str. Malish 7, complete genome
6 Rickettsia sibirica 246 rsib_agncrt, whole genome shotgun sequence
7 Rickettsia rickettsii chromosome, whole genome shotgun sequence

Reg_id: 12

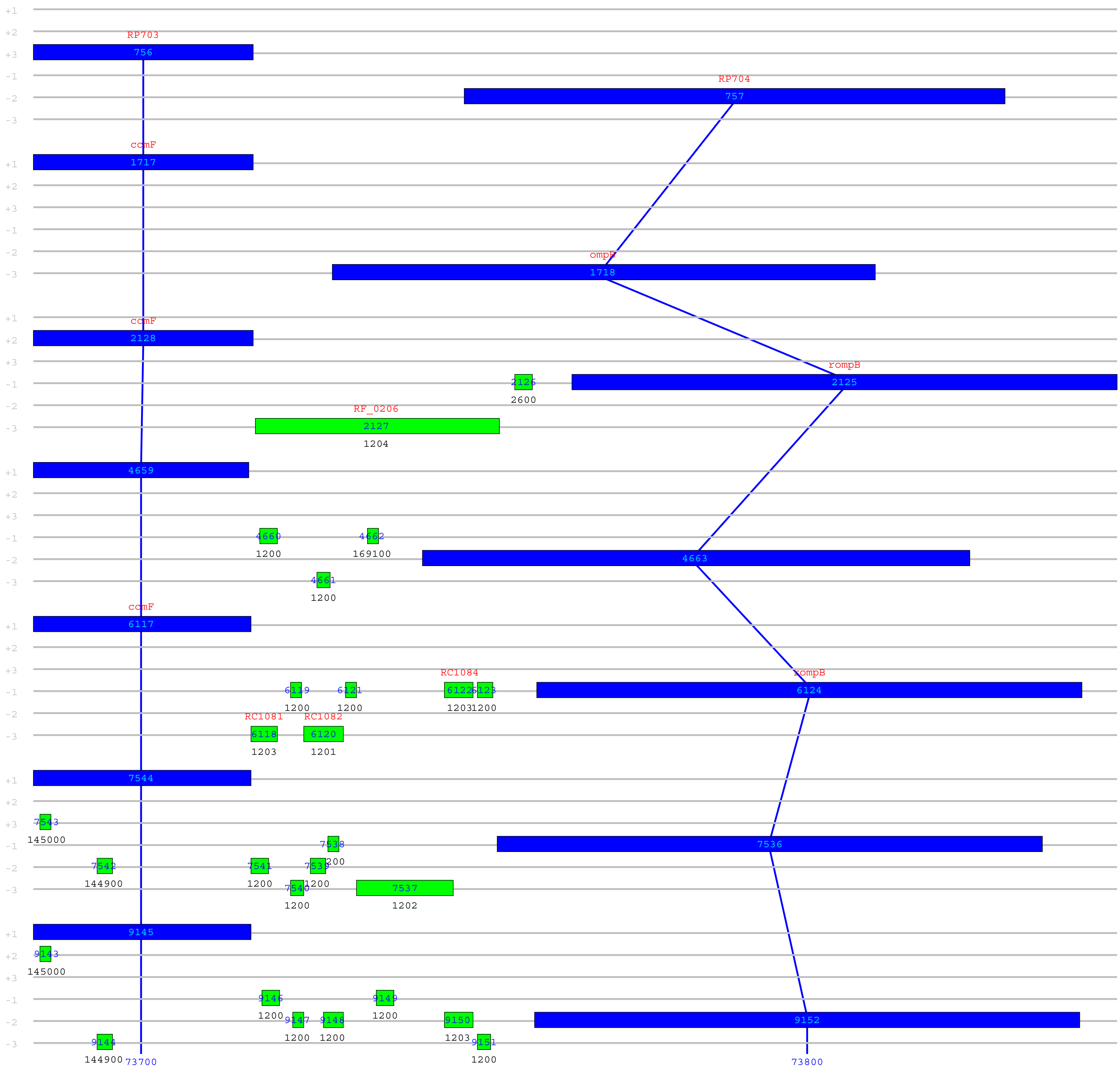

1 Rickettsia prowazekii str. Madrid E, complete genome
2 Rickettsia typhi str. wilmington, complete genome
3 Rickettsia felis URRWXCal2, complete genome
4 Rickettsia akari str. Hartford chromosome, whole genome shotgun sequence
5 Rickettsia conorii str. Malish 7, complete genome
6 Rickettsia sibirica 246 rsib_agncrt, whole genome shotgun sequence
7 Rickettsia rickettsii chromosome, whole genome shotgun sequence

Reg_id: 13

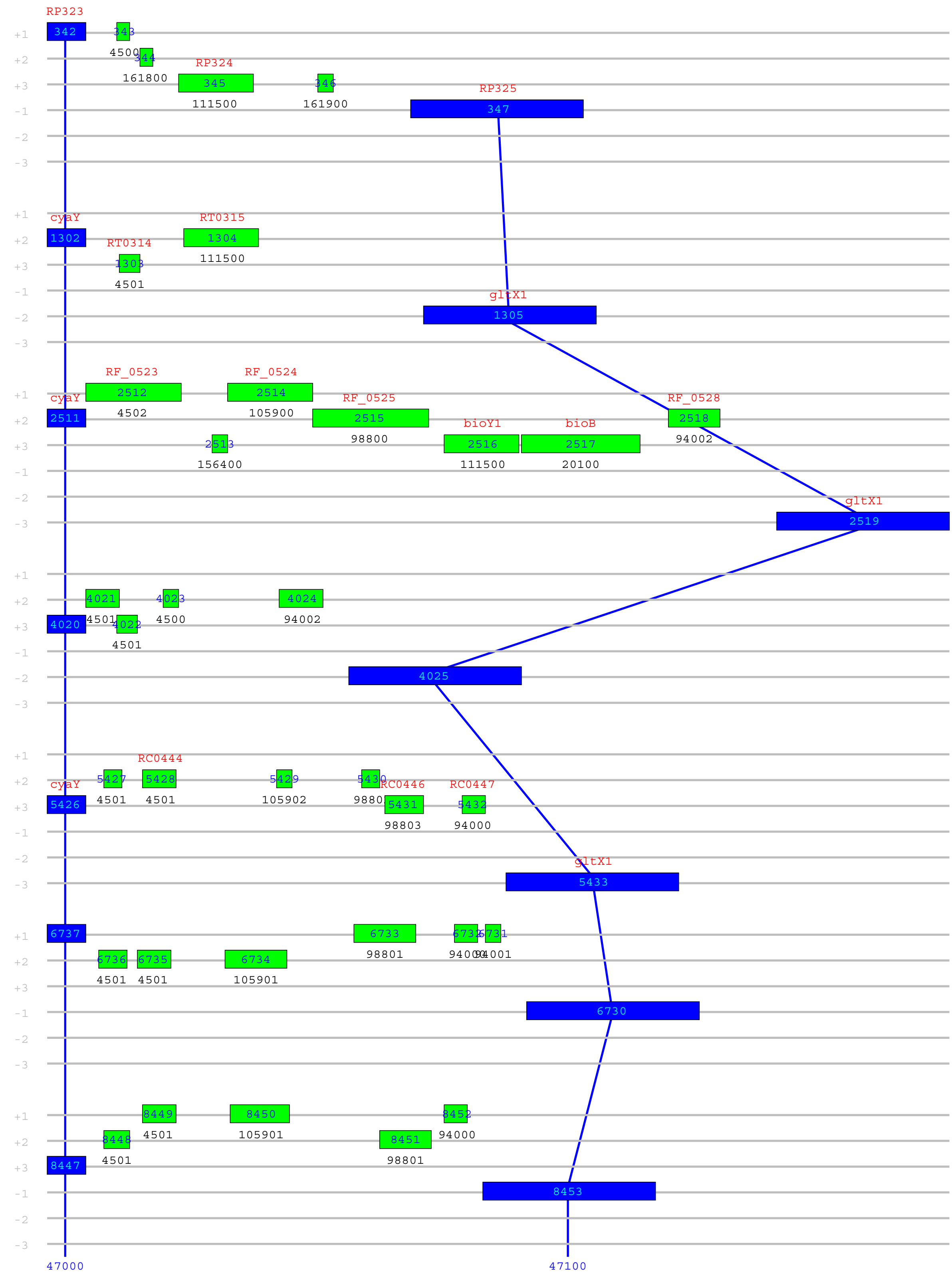

1 Rickettsia prowazekii str. Madrid E, complete genome
2 Rickettsia typhi str. wilmington, complete genome
3 Rickettsia felis URRWXCal2, complete genome
4 Rickettsia akari str. Hartford chromosome, whole genome shotgun sequence
5 Rickettsia conorii str. Malish 7, complete genome
6 Rickettsia sibirica 246 rsib_agncrt, whole genome shotgun sequence
7 Rickettsia rickettsii chromosome, whole genome shotgun sequence

Reg_id: 14

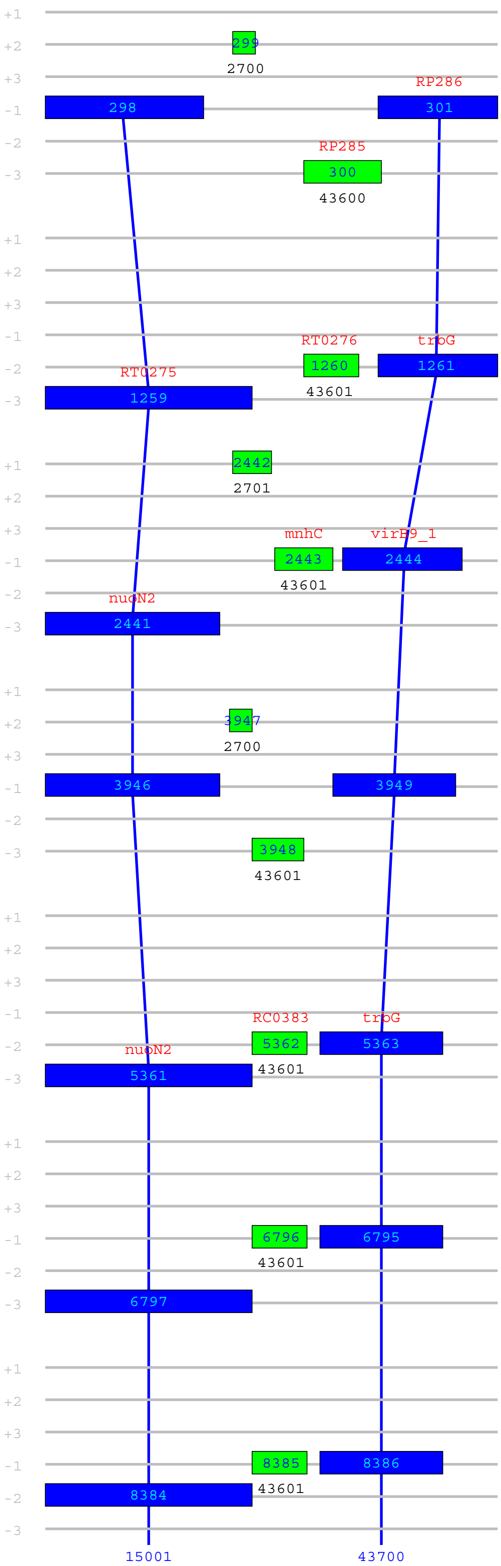

1 Rickettsia prowazekii str. Madrid E, complete genome
2 Rickettsia typhi str. wilmington, complete genome
3 Rickettsia felis URRWXCal2, complete genome
4 Rickettsia akari str. Hartford chromosome, whole genome shotgun sequence
5 Rickettsia conorii str. Malish 7, complete genome
6 Rickettsia sibirica 246 rsib_agncrt, whole genome shotgun sequence
7 Rickettsia rickettsii chromosome, whole genome shotgun sequence

Reg_id: 17

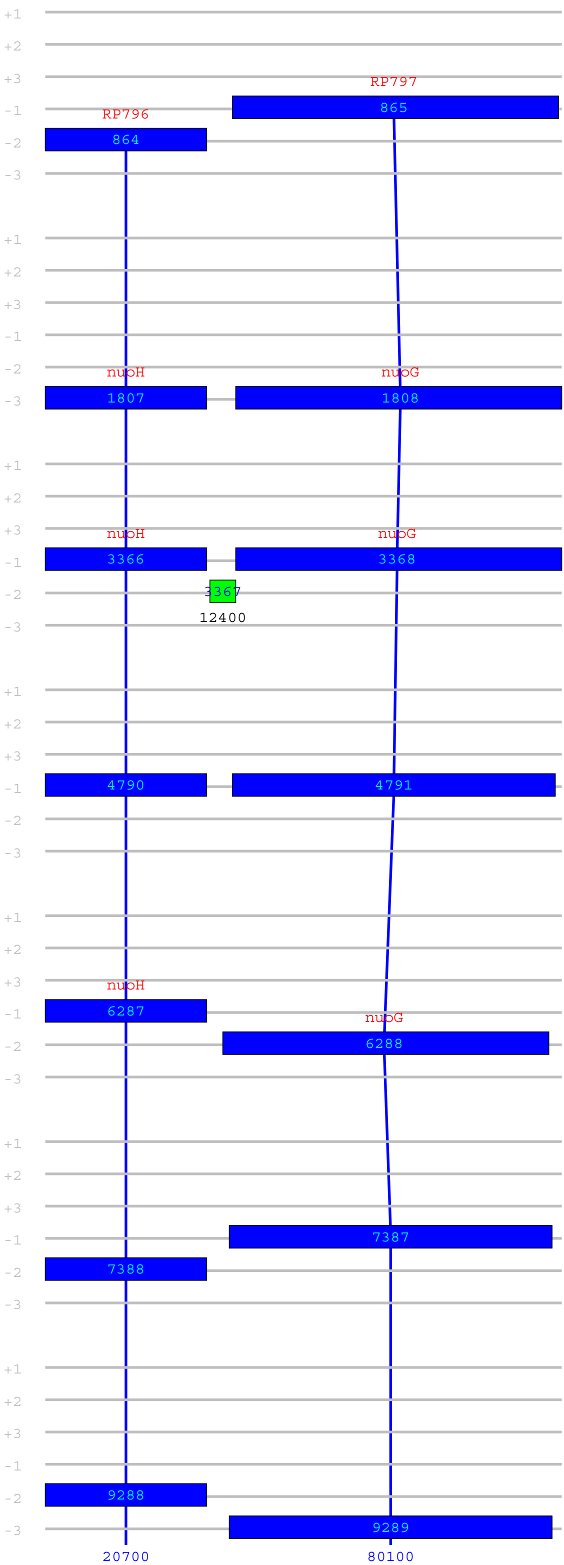

1 Rickettsia prowazekii str. Madrid E, complete genome
2 Rickettsia typhi str. wilmington, complete genome
3 Rickettsia felis URRWXCal2, complete genome
4 Rickettsia akari str. Hartford chromosome, whole genome shotgun sequence
5 Rickettsia conorii str. Malish 7, complete genome
6 Rickettsia sibirica 246 rsib_agncrt, whole genome shotgun sequence
7 Rickettsia rickettsii chromosome, whole genome shotgun sequence

Reg_id: 18

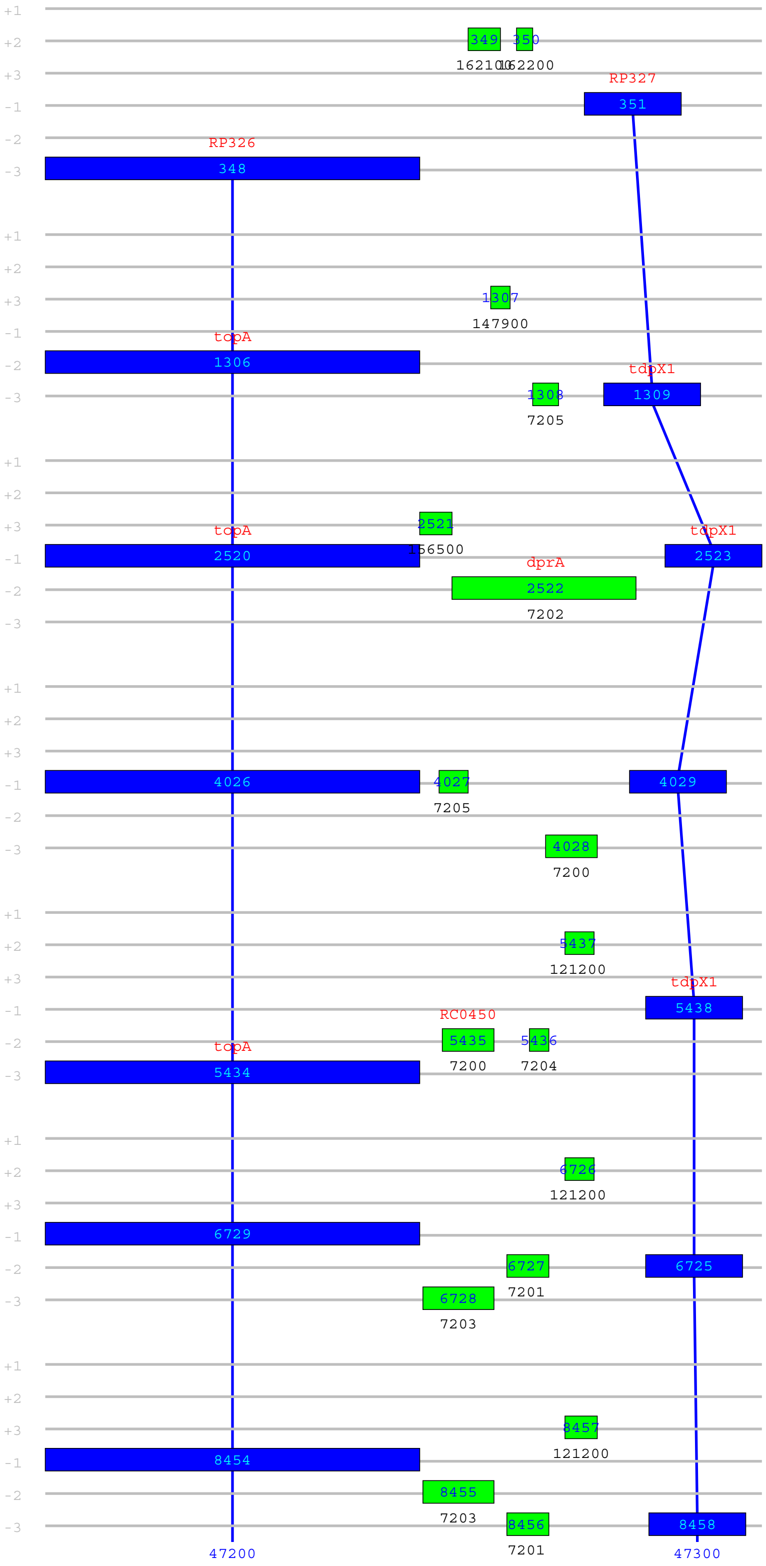

1 Rickettsia prowazekii str. Madrid E, complete genome
2 Rickettsia typhi str. wilmington, complete genome
3 Rickettsia felis URRWXCal2, complete genome
4 Rickettsia akari str. Hartford chromosome, whole genome shotgun sequence
5 Rickettsia conorii str. Malish 7, complete genome
6 Rickettsia sibirica 246 rsib_agncrt, whole genome shotgun sequence
7 Rickettsia rickettsii chromosome, whole genome shotgun sequence

Reg_id: 24

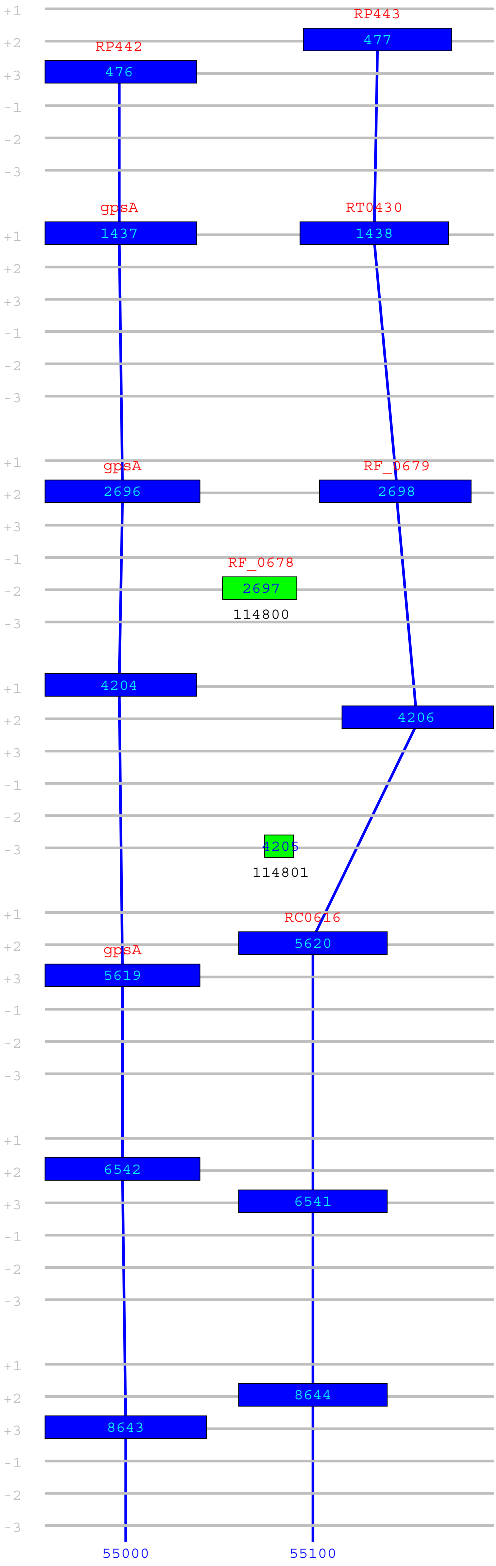

1 Rickettsia prowazekii str. Madrid E, complete genome
2 Rickettsia typhi str. wilmington, complete genome
3 Rickettsia felis URRWXCal2, complete genome
4 Rickettsia akari str. Hartford chromosome, whole genome shotgun sequence
5 Rickettsia conorii str. Malish 7, complete genome
6 Rickettsia sibirica 246 rsib_agncrt, whole genome shotgun sequence
7 Rickettsia rickettsii chromosome, whole genome shotgun sequence

Reg_id: 25

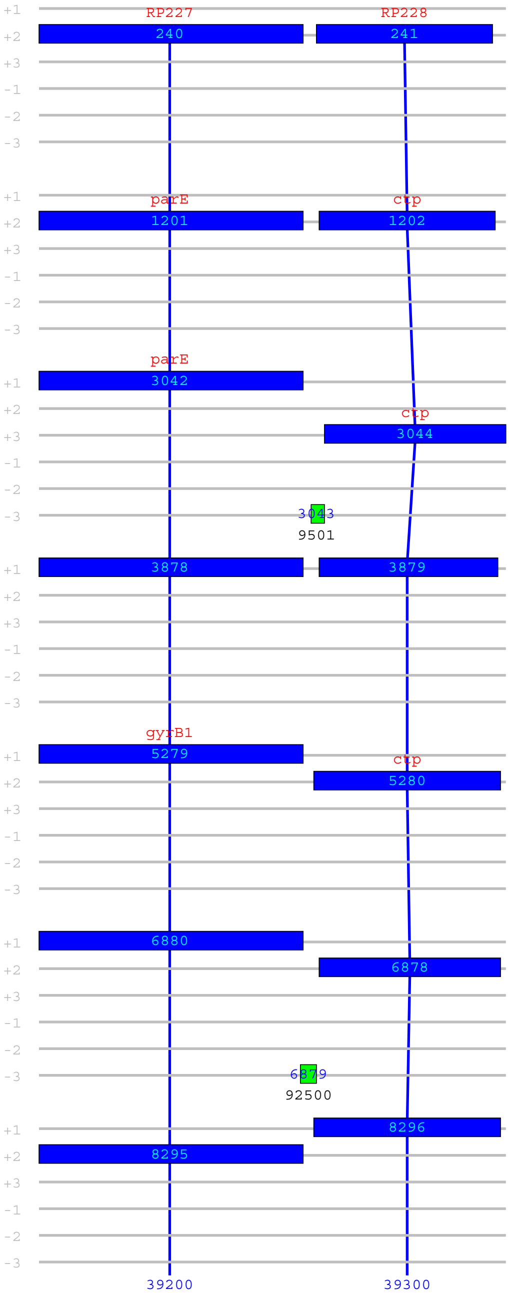

1 Rickettsia prowazekii str. Madrid E, complete genome
2 Rickettsia typhi str. wilmington, complete genome
3 Rickettsia felis URRWXCal2, complete genome
4 Rickettsia akari str. Hartford chromosome, whole genome shotgun sequence
5 Rickettsia conorii str. Malish 7, complete genome
6 Rickettsia sibirica 246 rsib_agncrt, whole genome shotgun sequence
7 Rickettsia rickettsii chromosome, whole genome shotgun sequence

Reg_id: 29

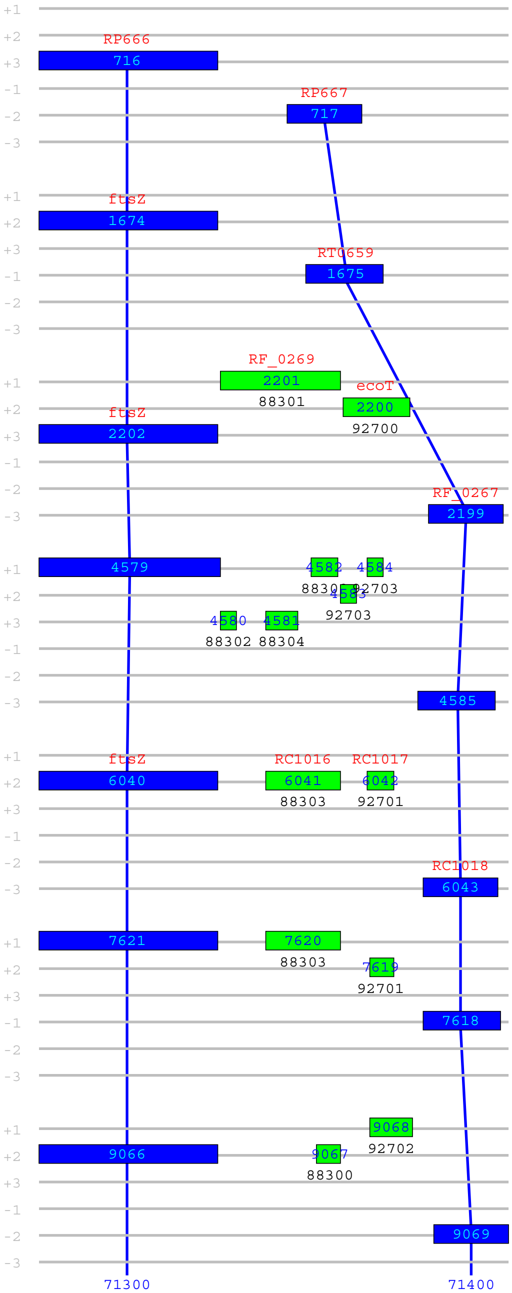

1 Rickettsia prowazekii str. Madrid E, complete genome
2 Rickettsia typhi str. wilmington, complete genome
3 Rickettsia felis URRWXCal2, complete genome
4 Rickettsia akari str. Hartford chromosome, whole genome shotgun sequence
5 Rickettsia conorii str. Malish 7, complete genome
6 Rickettsia sibirica 246 rsib_agncrt, whole genome shotgun sequence
7 Rickettsia rickettsii chromosome, whole genome shotgun sequence

Reg_id: 31

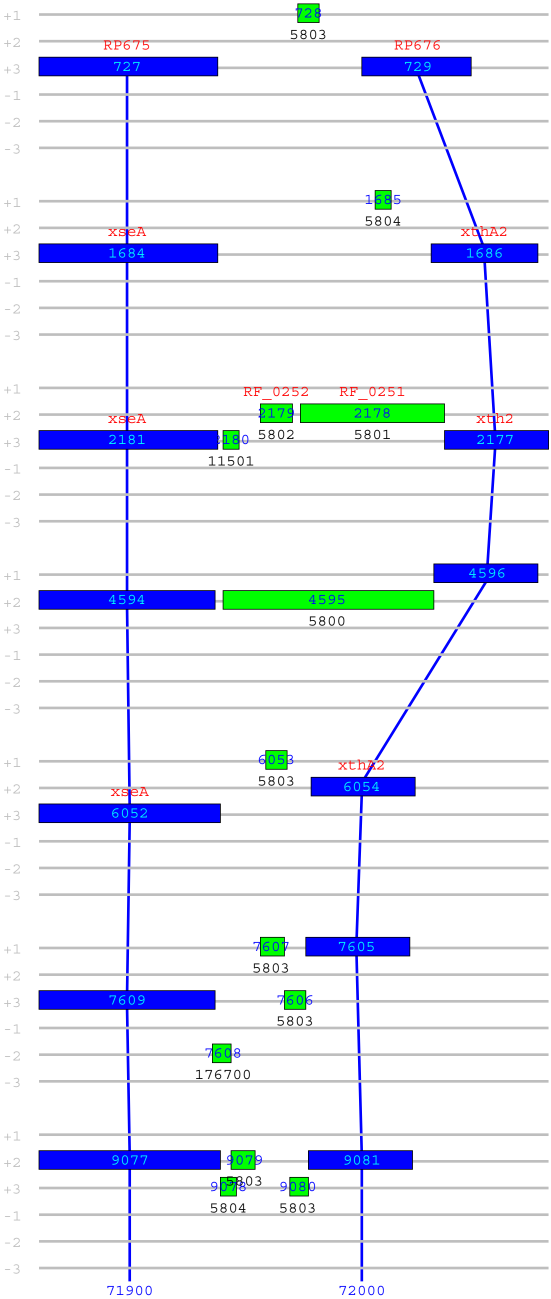

1 Rickettsia prowazekii str. Madrid E, complete genome
2 Rickettsia typhi str. wilmington, complete genome
3 Rickettsia felis URRWXCal2, complete genome
4 Rickettsia akari str. Hartford chromosome, whole genome shotgun sequence
5 Rickettsia conorii str. Malish 7, complete genome
6 Rickettsia sibirica 246 rsib_agncrt, whole genome shotgun sequence
7 Rickettsia rickettsii chromosome, whole genome shotgun sequence

Reg_id: 32

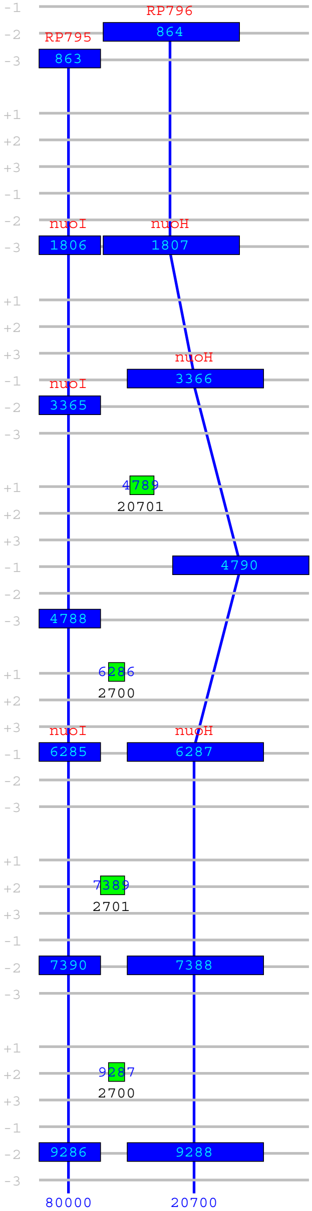

1 Rickettsia prowazekii str. Madrid E, complete genome
2 Rickettsia typhi str. wilmington, complete genome
3 Rickettsia felis URRWXCal2, complete genome
4 Rickettsia akari str. Hartford chromosome, whole genome shotgun sequence
5 Rickettsia conorii str. Malish 7, complete genome
6 Rickettsia sibirica 246 rsib_agncrt, whole genome shotgun sequence
7 Rickettsia rickettsii chromosome, whole genome shotgun sequence

Reg_id: 33

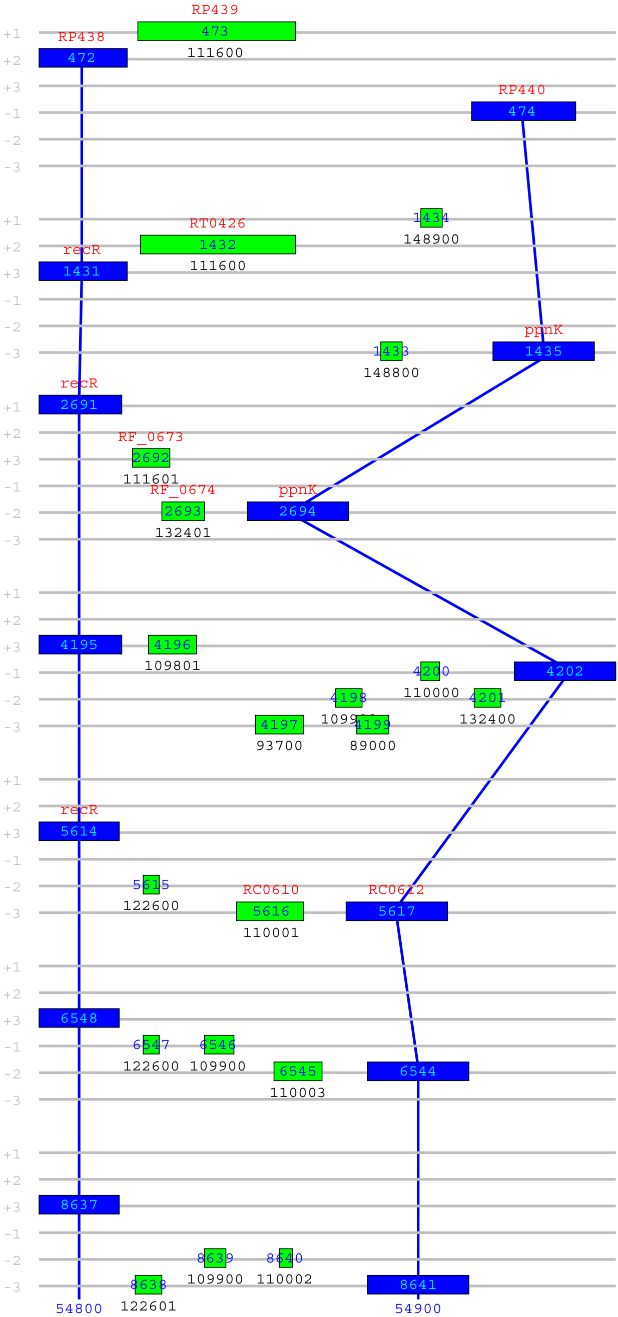

1 Rickettsia prowazekii str. Madrid E, complete genome
2 Rickettsia typhi str. wilmington, complete genome
3 Rickettsia felis URRWXCal2, complete genome
4 Rickettsia akari str. Hartford chromosome, whole genome shotgun sequence
5 Rickettsia conorii str. Malish 7, complete genome
6 Rickettsia sibirica 246 rsib_agncrt, whole genome shotgun sequence
7 Rickettsia rickettsii chromosome, whole genome shotgun sequence

Reg_id: 34

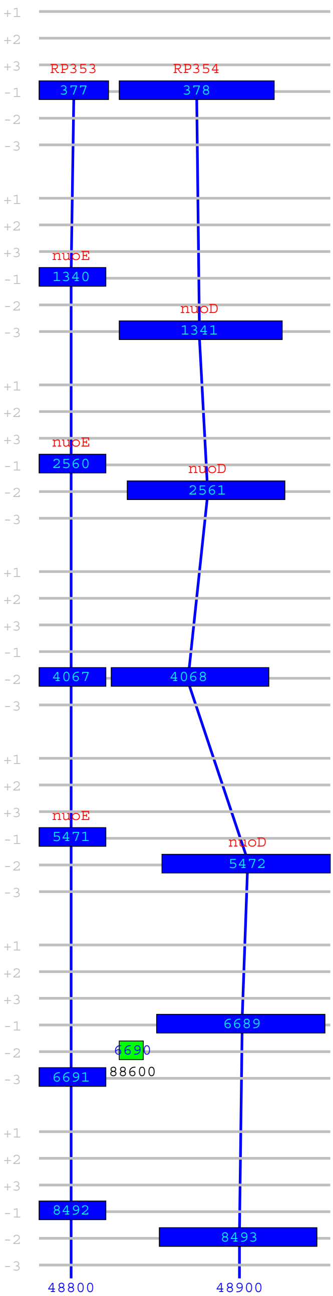

1 Rickettsia prowazekii str. Madrid E, complete genome
2 Rickettsia typhi str. wilmington, complete genome
3 Rickettsia felis URRWXCal2, complete genome
4 Rickettsia akari str. Hartford chromosome, whole genome shotgun sequence
5 Rickettsia conorii str. Malish 7, complete genome
6 Rickettsia sibirica 246 rsib_agncrt, whole genome shotgun sequence
7 Rickettsia rickettsii chromosome, whole genome shotgun sequence

Reg_id: 36

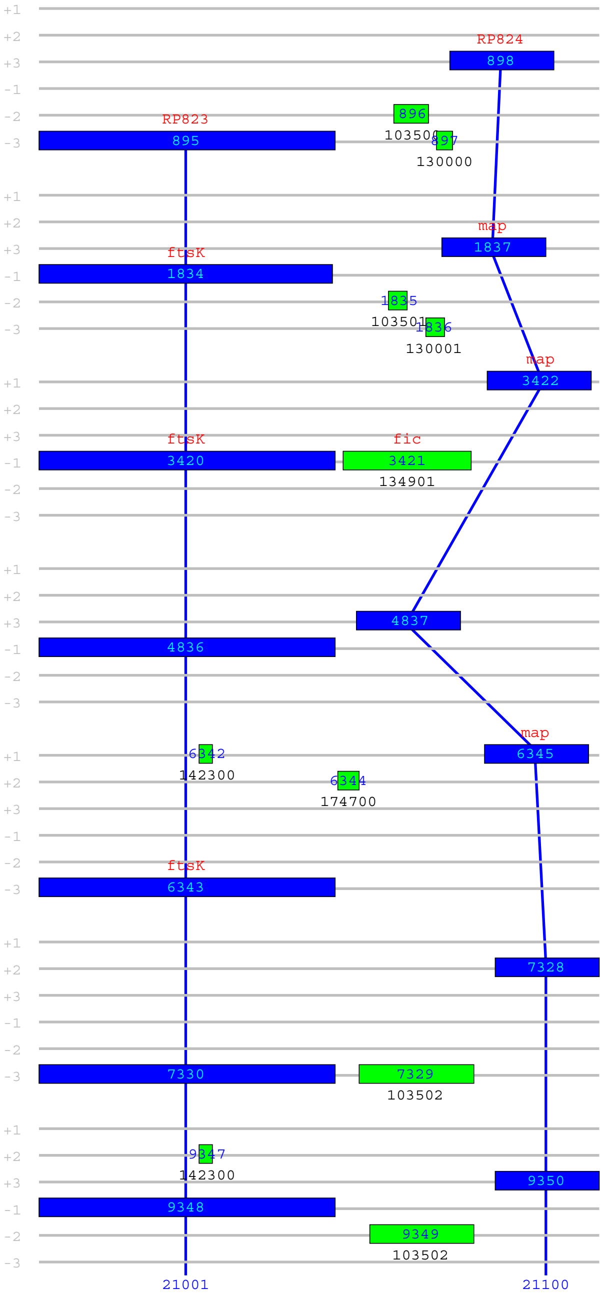

1 Rickettsia prowazekii str. Madrid E, complete genome
2 Rickettsia typhi str. wilmington, complete genome
3 Rickettsia felis URRWXCal2, complete genome
4 Rickettsia akari str. Hartford chromosome, whole genome shotgun sequence
5 Rickettsia conorii str. Malish 7, complete genome
6 Rickettsia sibirica 246 rsib_agncrt, whole genome shotgun sequence
7 Rickettsia rickettsii chromosome, whole genome shotgun sequence

Reg_id: 38

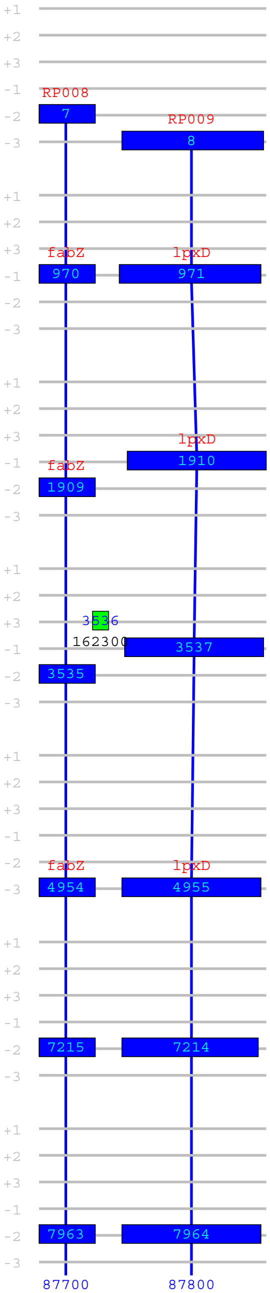

1 Rickettsia prowazekii str. Madrid E, complete genome
2 Rickettsia typhi str. wilmington, complete genome
3 Rickettsia felis URRWXCal2, complete genome
4 Rickettsia akari str. Hartford chromosome, whole genome shotgun sequence
5 Rickettsia conorii str. Malish 7, complete genome
6 Rickettsia sibirica 246 rsib_agncrt, whole genome shotgun sequence
7 Rickettsia rickettsii chromosome, whole genome shotgun sequence

Reg_id: 46

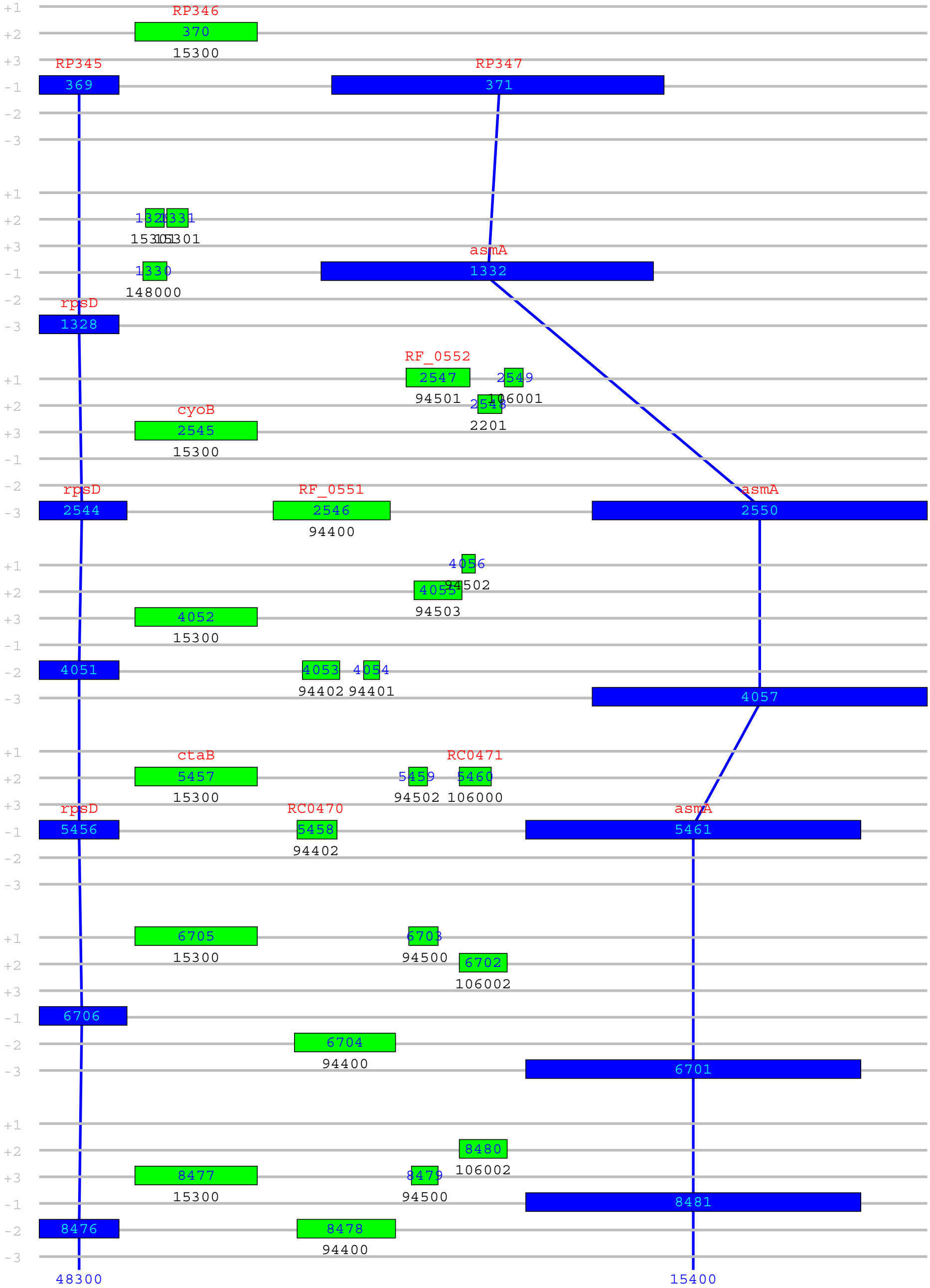

1 Rickettsia prowazekii str. Madrid E, complete genome
2 Rickettsia typhi str. wilmington, complete genome
3 Rickettsia felis URRWXCal2, complete genome
4 Rickettsia akari str. Hartford chromosome, whole genome shotgun sequence
5 Rickettsia conorii str. Malish 7, complete genome
6 Rickettsia sibirica 246 rsib_agncrt, whole genome shotgun sequence
7 Rickettsia rickettsii chromosome, whole genome shotgun sequence

Reg_id: 48

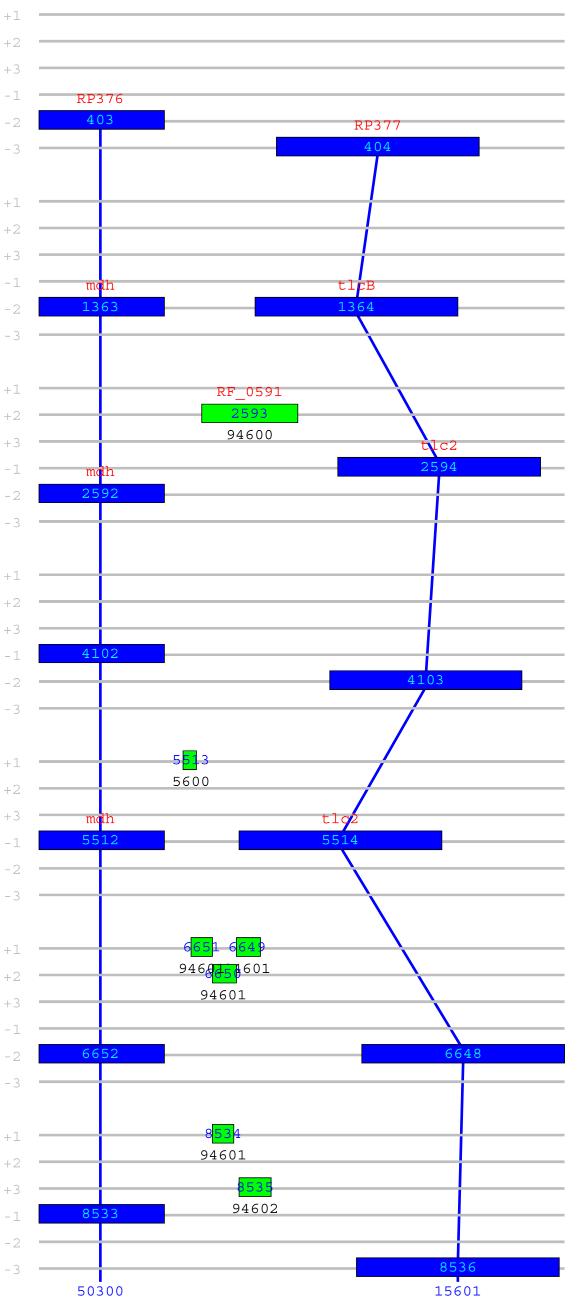

1 Rickettsia prowazekii str. Madrid E, complete genome
2 Rickettsia typhi str. wilmington, complete genome
3 Rickettsia felis URRWXCal2, complete genome
4 Rickettsia akari str. Hartford chromosome, whole genome shotgun sequence
5 Rickettsia conorii str. Malish 7, complete genome
6 Rickettsia sibirica 246 rsib_agncrt, whole genome shotgun sequence
7 Rickettsia rickettsii chromosome, whole genome shotgun sequence

Reg_id: 49

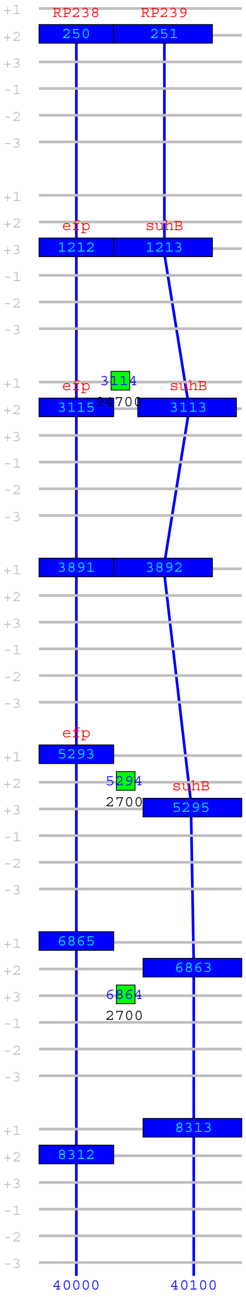

1 Rickettsia prowazekii str. Madrid E, complete genome
2 Rickettsia typhi str. wilmington, complete genome
3 Rickettsia felis URRWXCal2, complete genome
4 Rickettsia akari str. Hartford chromosome, whole genome shotgun sequence
5 Rickettsia conorii str. Malish 7, complete genome
6 Rickettsia sibirica 246 rsib_agncrt, whole genome shotgun sequence
7 Rickettsia rickettsii chromosome, whole genome shotgun sequence

Reg_id: 50

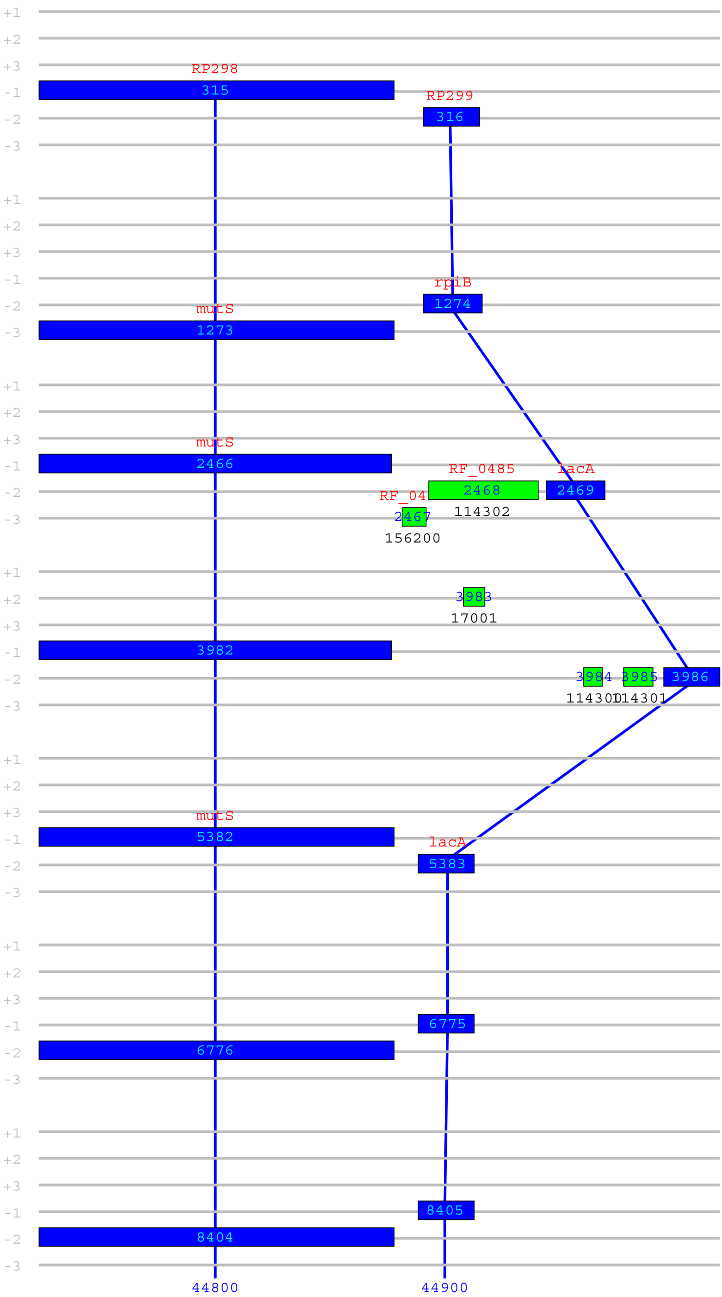

1 Rickettsia prowazekii str. Madrid E, complete genome
2 Rickettsia typhi str. wilmington, complete genome
3 Rickettsia felis URRWXCal2, complete genome
4 Rickettsia akari str. Hartford chromosome, whole genome shotgun sequence
5 Rickettsia conorii str. Malish 7, complete genome
6 Rickettsia sibirica 246 rsib_agncrt, whole genome shotgun sequence
7 Rickettsia rickettsii chromosome, whole genome shotgun sequence

Reg_id: 51

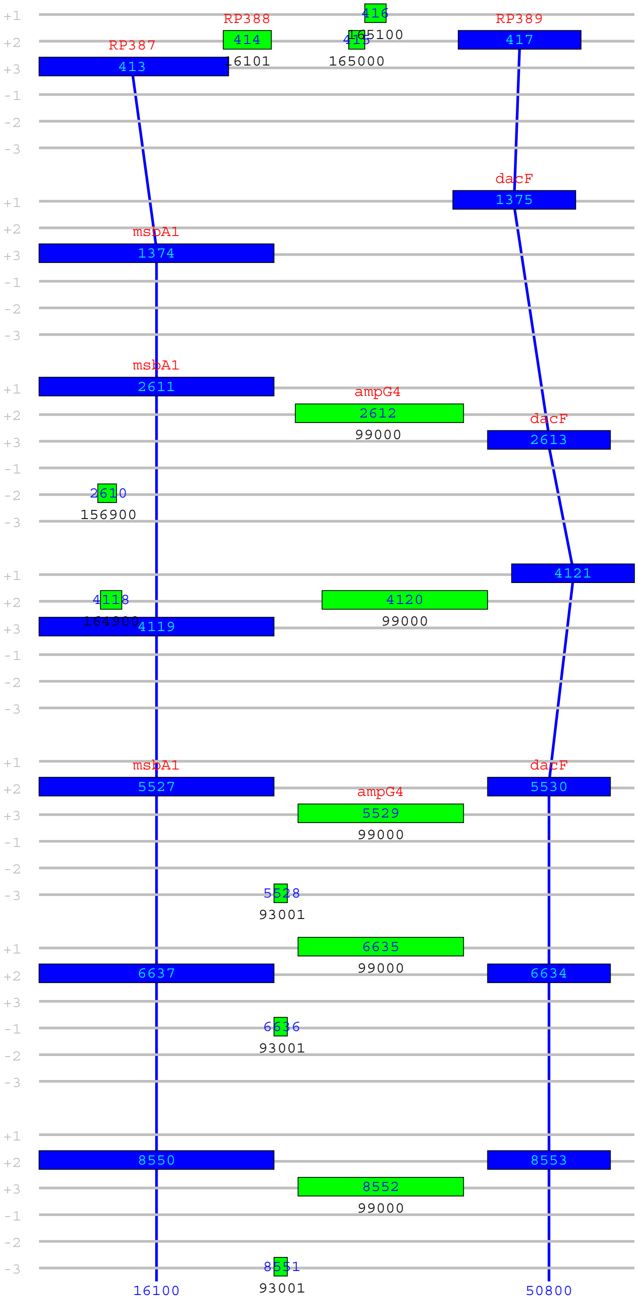

1 Rickettsia prowazekii str. Madrid E, complete genome
2 Rickettsia typhi str. wilmington, complete genome
3 Rickettsia felis URRWXCal2, complete genome
4 Rickettsia akari str. Hartford chromosome, whole genome shotgun sequence
5 Rickettsia conorii str. Malish 7, complete genome
6 Rickettsia sibirica 246 rsib_agncrt, whole genome shotgun sequence
7 Rickettsia rickettsii chromosome, whole genome shotgun sequence

Reg_id: 53

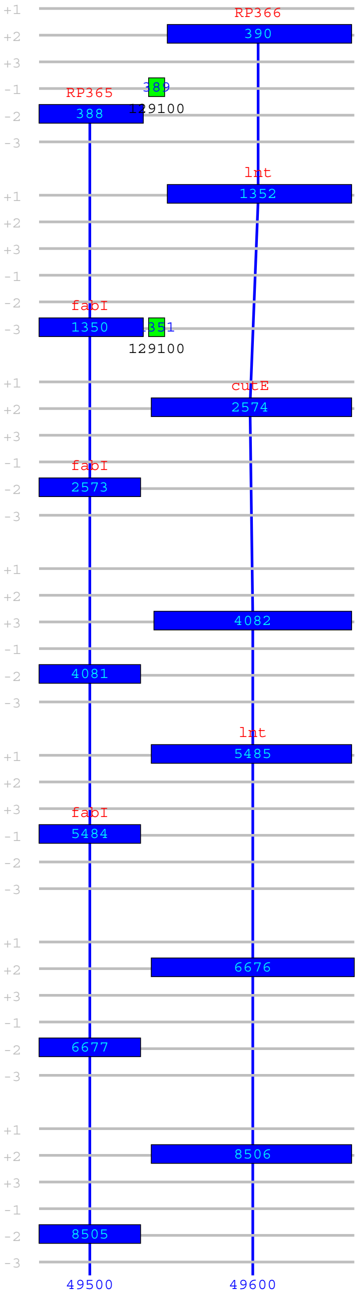

1 Rickettsia prowazekii str. Madrid E, complete genome
2 Rickettsia typhi str. wilmington, complete genome
3 Rickettsia felis URRWXCal2, complete genome
4 Rickettsia akari str. Hartford chromosome, whole genome shotgun sequence
5 Rickettsia conorii str. Malish 7, complete genome
6 Rickettsia sibirica 246 rsib_agncrt, whole genome shotgun sequence
7 Rickettsia rickettsii chromosome, whole genome shotgun sequence

Reg_id: 55

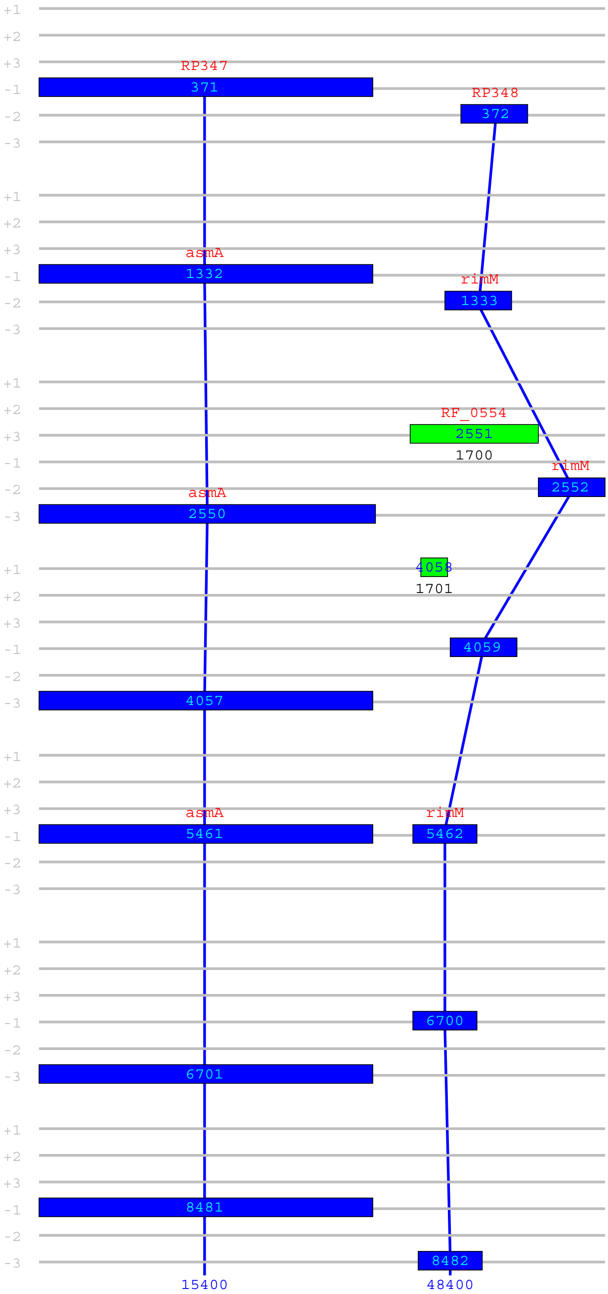

1 Rickettsia prowazekii str. Madrid E, complete genome
2 Rickettsia typhi str. wilmington, complete genome
3 Rickettsia felis URRWXCal2, complete genome
4 Rickettsia akari str. Hartford chromosome, whole genome shotgun sequence
5 Rickettsia conorii str. Malish 7, complete genome
6 Rickettsia sibirica 246 rsib_agncrt, whole genome shotgun sequence
7 Rickettsia rickettsii chromosome, whole genome shotgun sequence

Reg_id: 56

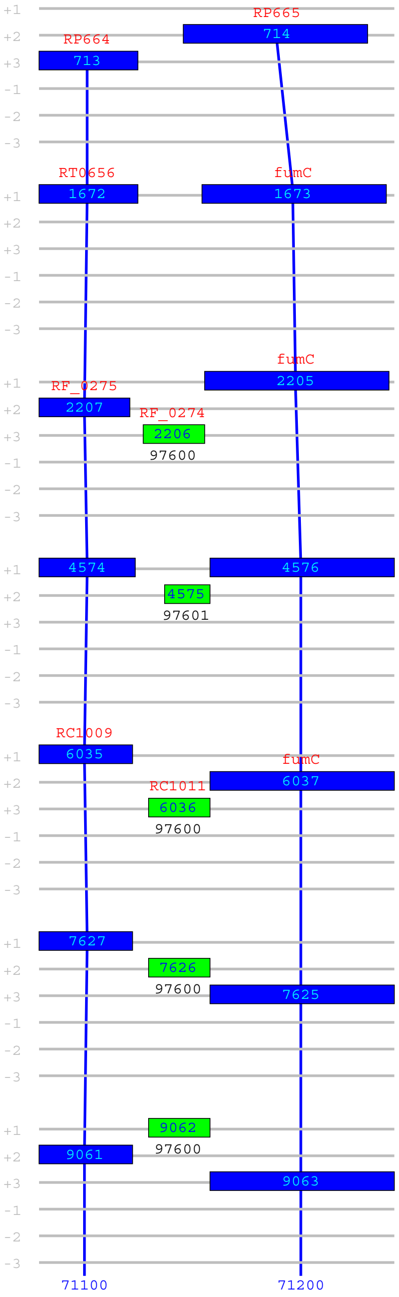

1 Rickettsia prowazekii str. Madrid E, complete genome
2 Rickettsia typhi str. wilmington, complete genome
3 Rickettsia felis URRWXCal2, complete genome
4 Rickettsia akari str. Hartford chromosome, whole genome shotgun sequence
5 Rickettsia conorii str. Malish 7, complete genome
6 Rickettsia sibirica 246 rsib_agncrt, whole genome shotgun sequence
7 Rickettsia rickettsii chromosome, whole genome shotgun sequence

Reg_id: 57

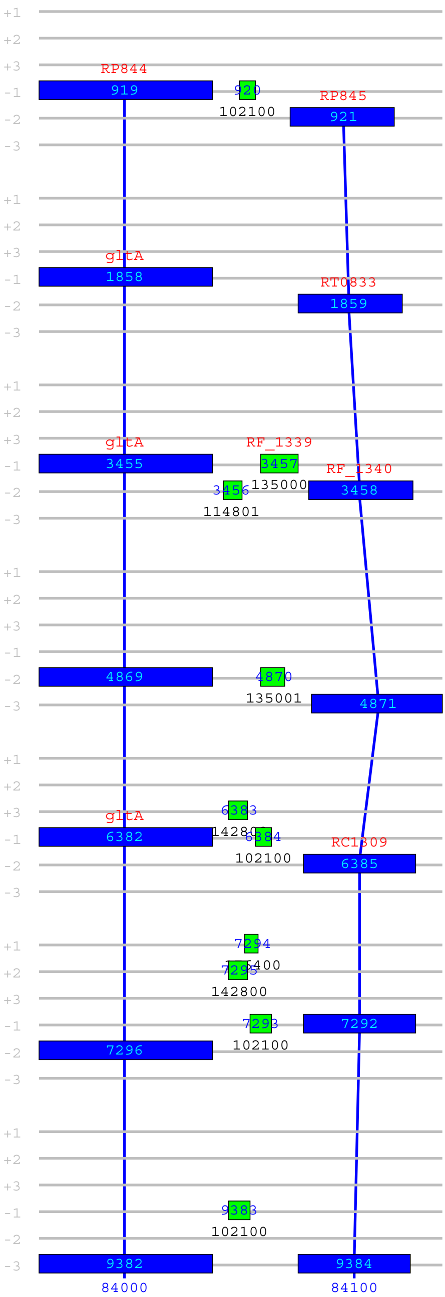

1 Rickettsia prowazekii str. Madrid E, complete genome
2 Rickettsia typhi str. wilmington, complete genome
3 Rickettsia felis URRWXCal2, complete genome
4 Rickettsia akari str. Hartford chromosome, whole genome shotgun sequence
5 Rickettsia conorii str. Malish 7, complete genome
6 Rickettsia sibirica 246 rsib_agncrt, whole genome shotgun sequence
7 Rickettsia rickettsii chromosome, whole genome shotgun sequence

Reg_id: 60

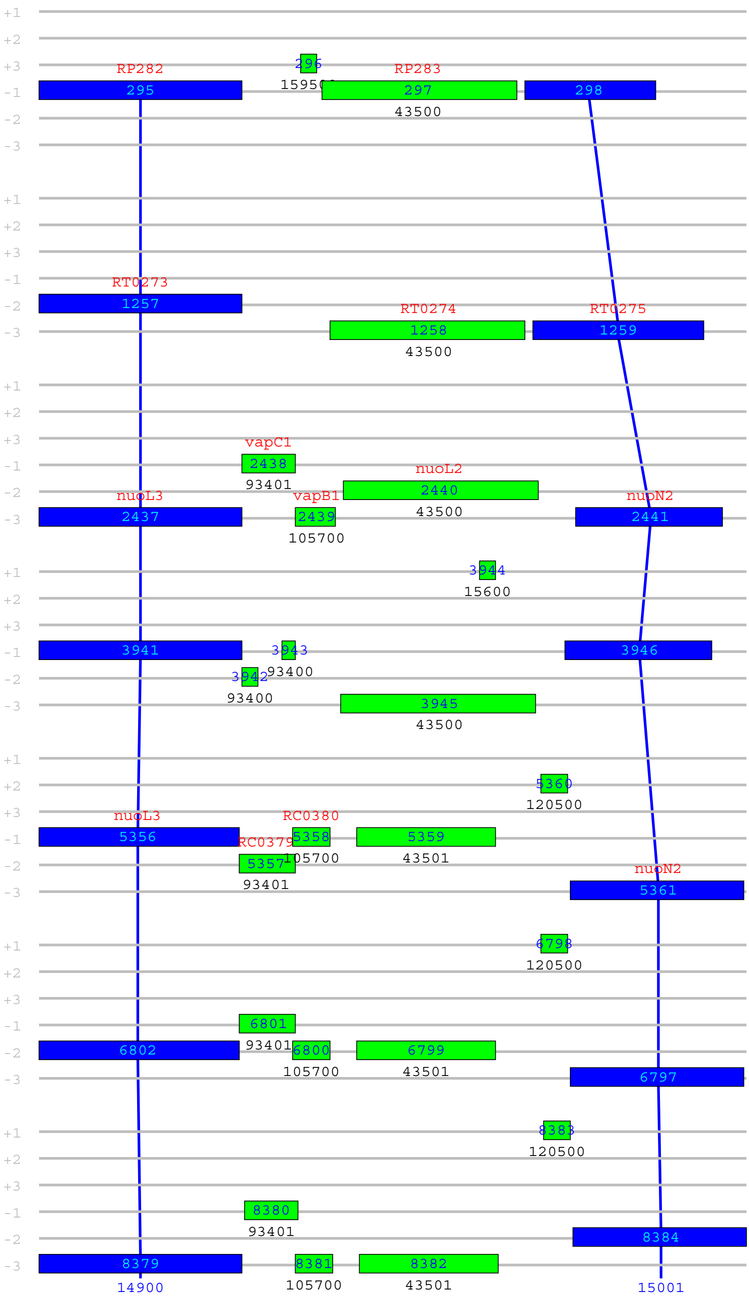

1 Rickettsia prowazekii str. Madrid E, complete genome
2 Rickettsia typhi str. wilmington, complete genome
3 Rickettsia felis URRWXCal2, complete genome
4 Rickettsia akari str. Hartford chromosome, whole genome shotgun sequence
5 Rickettsia conorii str. Malish 7, complete genome
6 Rickettsia sibirica 246 rsib_agncrt, whole genome shotgun sequence
7 Rickettsia rickettsii chromosome, whole genome shotgun sequence

Reg_id: 62

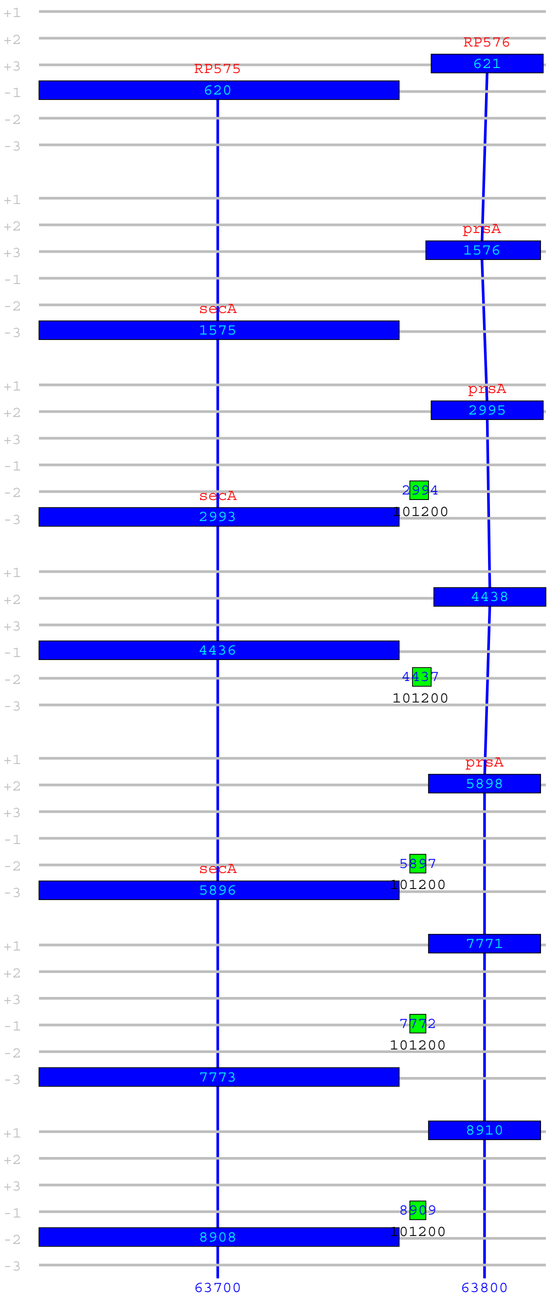

1 Rickettsia prowazekii str. Madrid E, complete genome
2 Rickettsia typhi str. wilmington, complete genome
3 Rickettsia felis URRWXCal2, complete genome
4 Rickettsia akari str. Hartford chromosome, whole genome shotgun sequence
5 Rickettsia conorii str. Malish 7, complete genome
6 Rickettsia sibirica 246 rsib_agncrt, whole genome shotgun sequence
7 Rickettsia rickettsii chromosome, whole genome shotgun sequence

Reg_id: 65

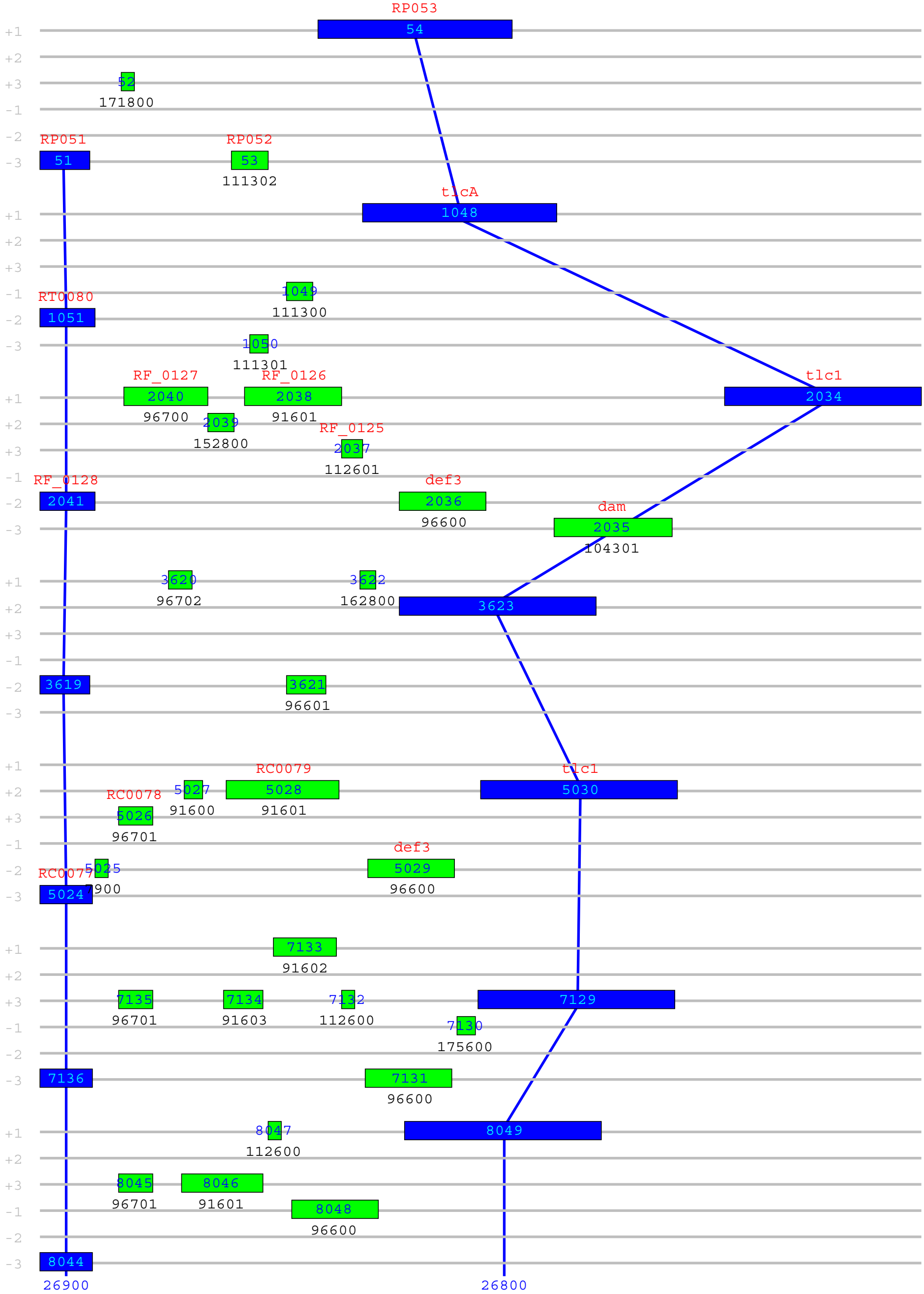

1 Rickettsia prowazekii str. Madrid E, complete genome
2 Rickettsia typhi str. wilmington, complete genome
3 Rickettsia felis URRWXCal2, complete genome
4 Rickettsia akari str. Hartford chromosome, whole genome shotgun sequence
5 Rickettsia conorii str. Malish 7, complete genome
6 Rickettsia sibirica 246 rsib_agncrt, whole genome shotgun sequence
7 Rickettsia rickettsii chromosome, whole genome shotgun sequence

Reg_id: 67

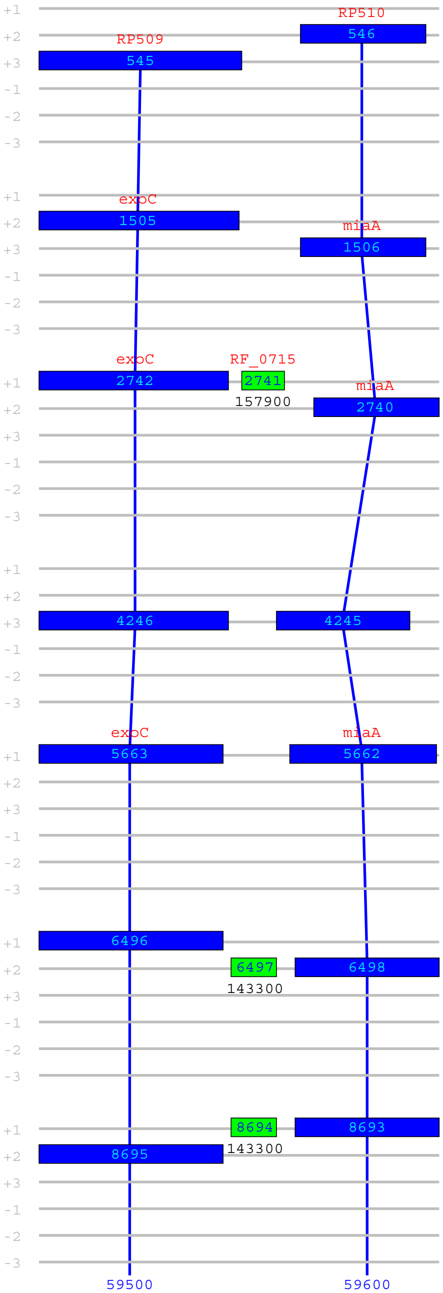

1 Rickettsia prowazekii str. Madrid E, complete genome
2 Rickettsia typhi str. wilmington, complete genome
3 Rickettsia felis URRWXCal2, complete genome
4 Rickettsia akari str. Hartford chromosome, whole genome shotgun sequence
5 Rickettsia conorii str. Malish 7, complete genome
6 Rickettsia sibirica 246 rsib_agncrt, whole genome shotgun sequence
7 Rickettsia rickettsii chromosome, whole genome shotgun sequence

Reg_id: 71

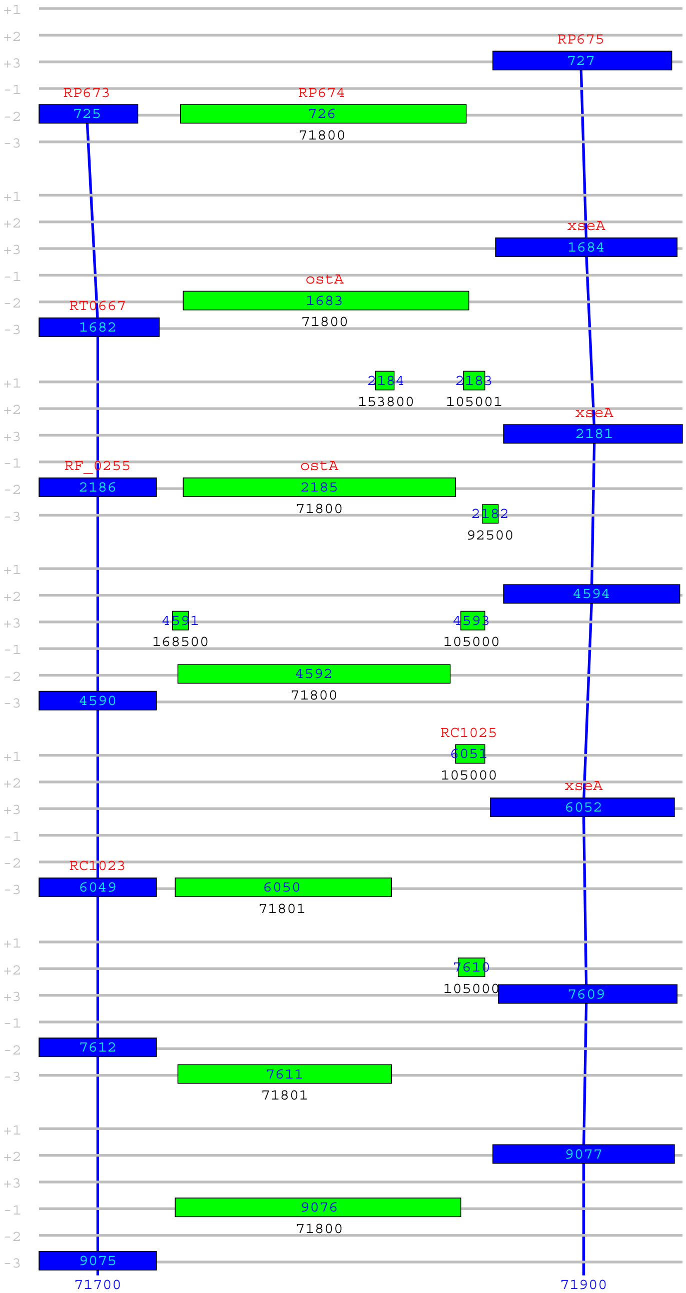

1 Rickettsia prowazekii str. Madrid E, complete genome
2 Rickettsia typhi str. wilmington, complete genome
3 Rickettsia felis URRWXCal2, complete genome
4 Rickettsia akari str. Hartford chromosome, whole genome shotgun sequence
5 Rickettsia conorii str. Malish 7, complete genome
6 Rickettsia sibirica 246 rsib_agncrt, whole genome shotgun sequence
7 Rickettsia rickettsii chromosome, whole genome shotgun sequence

Reg_id: 72

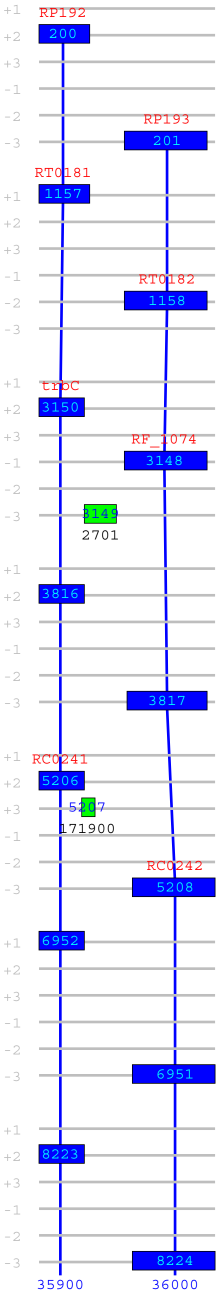

1 Rickettsia prowazekii str. Madrid E, complete genome
2 Rickettsia typhi str. wilmington, complete genome
3 Rickettsia felis URRWXCal2, complete genome
4 Rickettsia akari str. Hartford chromosome, whole genome shotgun sequence
5 Rickettsia conorii str. Malish 7, complete genome
6 Rickettsia sibirica 246 rsib_agncrt, whole genome shotgun sequence
7 Rickettsia rickettsii chromosome, whole genome shotgun sequence

Reg_id: 73

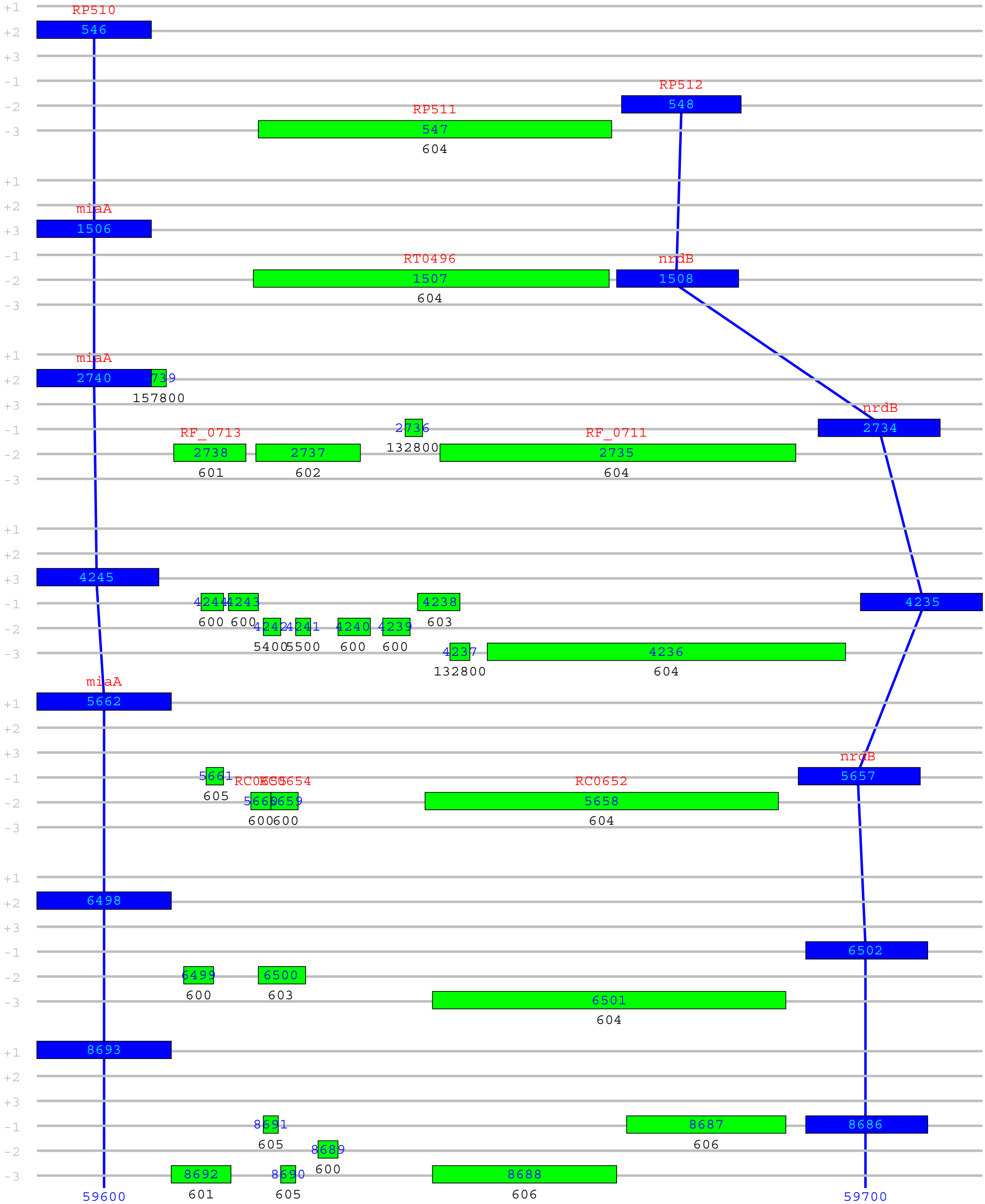

1 Rickettsia prowazekii str. Madrid E, complete genome
2 Rickettsia typhi str. wilmington, complete genome
3 Rickettsia felis URRWXCal2, complete genome
4 Rickettsia akari str. Hartford chromosome, whole genome shotgun sequence
5 Rickettsia conorii str. Malish 7, complete genome
6 Rickettsia sibirica 246 rsib_agncrt, whole genome shotgun sequence
7 Rickettsia rickettsii chromosome, whole genome shotgun sequence

Reg_id: 75

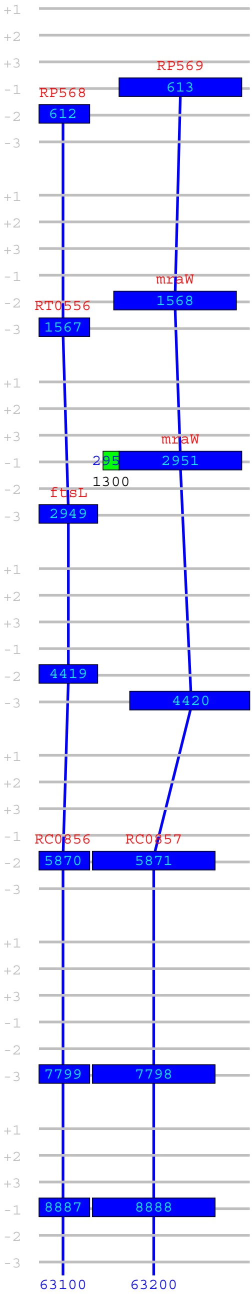

1 Rickettsia prowazekii str. Madrid E, complete genome
2 Rickettsia typhi str. wilmington, complete genome
3 Rickettsia felis URRWXCal2, complete genome
4 Rickettsia akari str. Hartford chromosome, whole genome shotgun sequence
5 Rickettsia conorii str. Malish 7, complete genome
6 Rickettsia sibirica 246 rsib_agncrt, whole genome shotgun sequence
7 Rickettsia rickettsii chromosome, whole genome shotgun sequence

Reg_id: 76

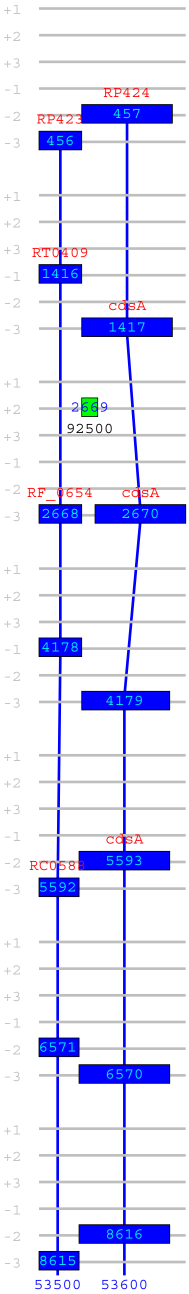

1 Rickettsia prowazekii str. Madrid E, complete genome
2 Rickettsia typhi str. wilmington, complete genome
3 Rickettsia felis URRWXCal2, complete genome
4 Rickettsia akari str. Hartford chromosome, whole genome shotgun sequence
5 Rickettsia conorii str. Malish 7, complete genome
6 Rickettsia sibirica 246 rsib_agncrt, whole genome shotgun sequence
7 Rickettsia rickettsii chromosome, whole genome shotgun sequence

Reg_id: 79

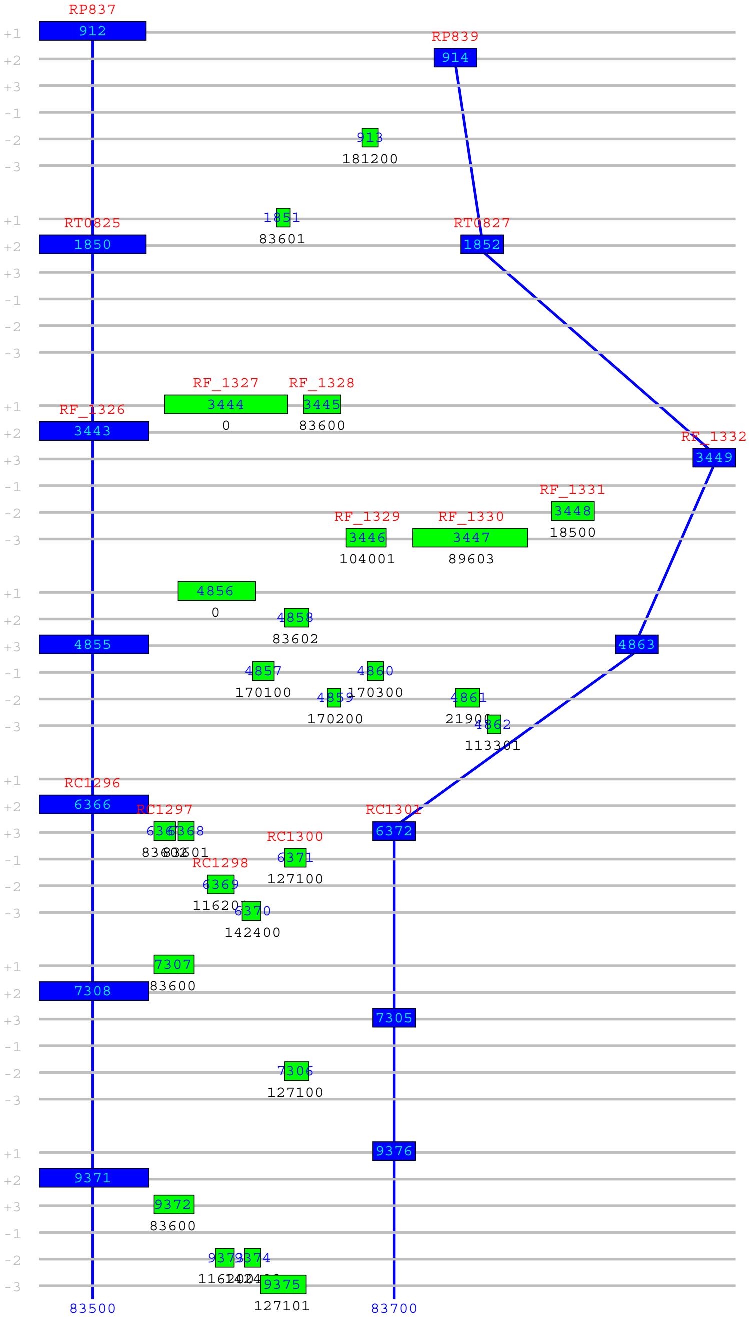

1 Rickettsia prowazekii str. Madrid E, complete genome
2 Rickettsia typhi str. wilmington, complete genome
3 Rickettsia felis URRWXCal2, complete genome
4 Rickettsia akari str. Hartford chromosome, whole genome shotgun sequence
5 Rickettsia conorii str. Malish 7, complete genome
6 Rickettsia sibirica 246 rsib_agncrt, whole genome shotgun sequence
7 Rickettsia rickettsii chromosome, whole genome shotgun sequence

Reg_id: 80

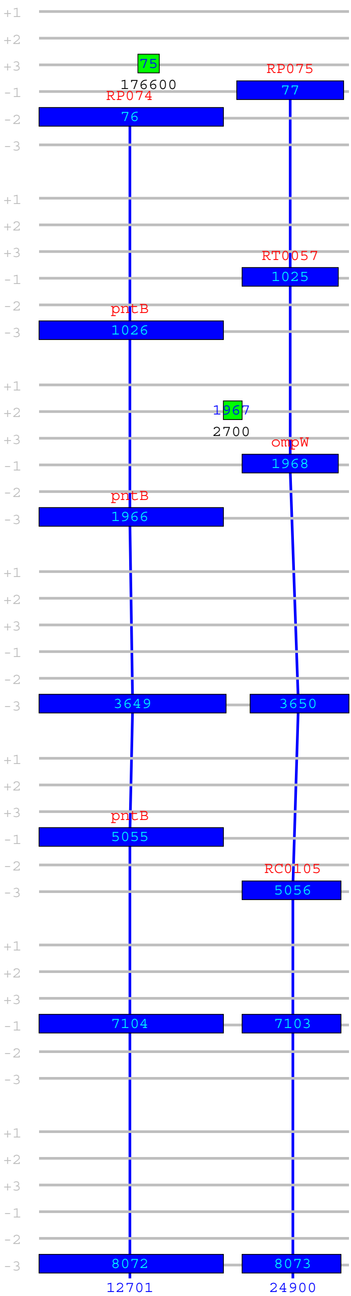

1 Rickettsia prowazekii str. Madrid E, complete genome
2 Rickettsia typhi str. wilmington, complete genome
3 Rickettsia felis URRWXCal2, complete genome
4 Rickettsia akari str. Hartford chromosome, whole genome shotgun sequence
5 Rickettsia conorii str. Malish 7, complete genome
6 Rickettsia sibirica 246 rsib_agncrt, whole genome shotgun sequence
7 Rickettsia rickettsii chromosome, whole genome shotgun sequence

Reg_id: 87

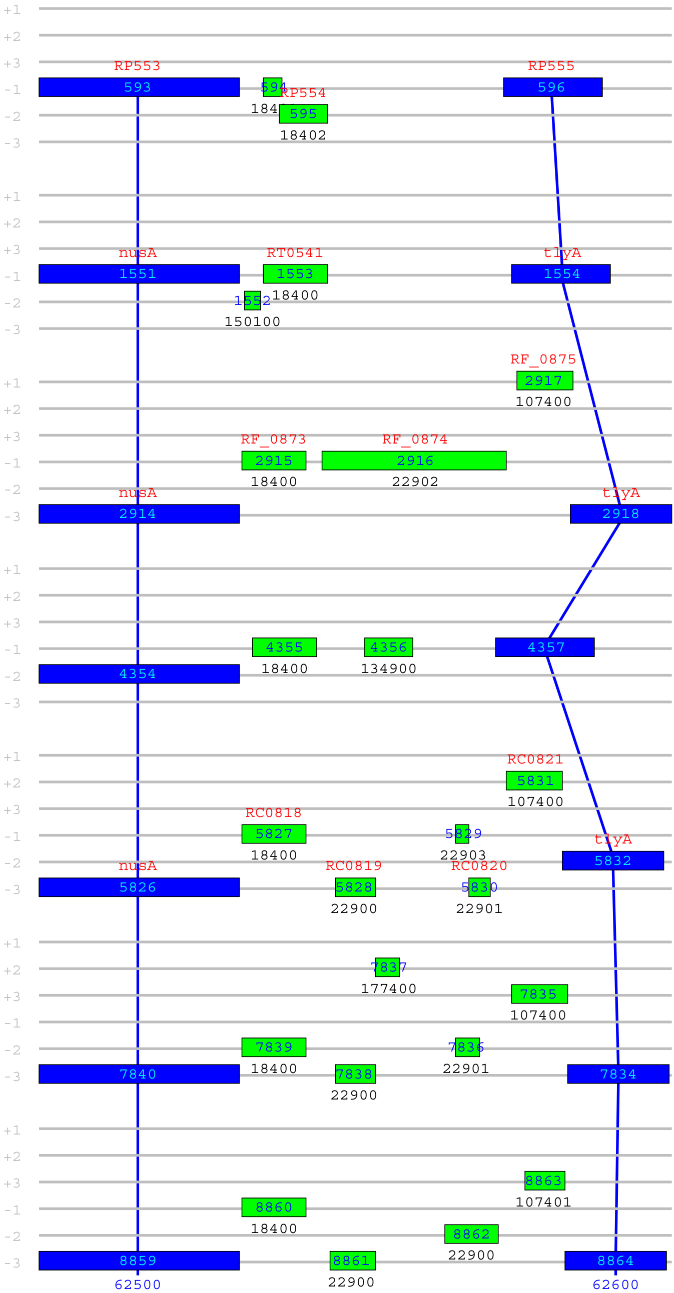

1 Rickettsia prowazekii str. Madrid E, complete genome
2 Rickettsia typhi str. wilmington, complete genome
3 Rickettsia felis URRWXCal2, complete genome
4 Rickettsia akari str. Hartford chromosome, whole genome shotgun sequence
5 Rickettsia conorii str. Malish 7, complete genome
6 Rickettsia sibirica 246 rsib_agncrt, whole genome shotgun sequence
7 Rickettsia rickettsii chromosome, whole genome shotgun sequence

Reg_id: 91

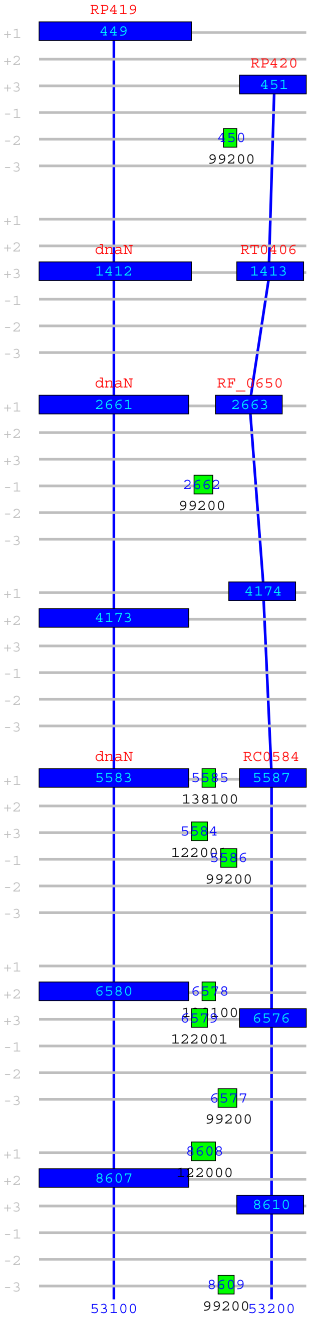

1 Rickettsia prowazekii str. Madrid E, complete genome
2 Rickettsia typhi str. wilmington, complete genome
3 Rickettsia felis URRWXCal2, complete genome
4 Rickettsia akari str. Hartford chromosome, whole genome shotgun sequence
5 Rickettsia conorii str. Malish 7, complete genome
6 Rickettsia sibirica 246 rsib_agncrt, whole genome shotgun sequence
7 Rickettsia rickettsii chromosome, whole genome shotgun sequence

Reg_id: 92

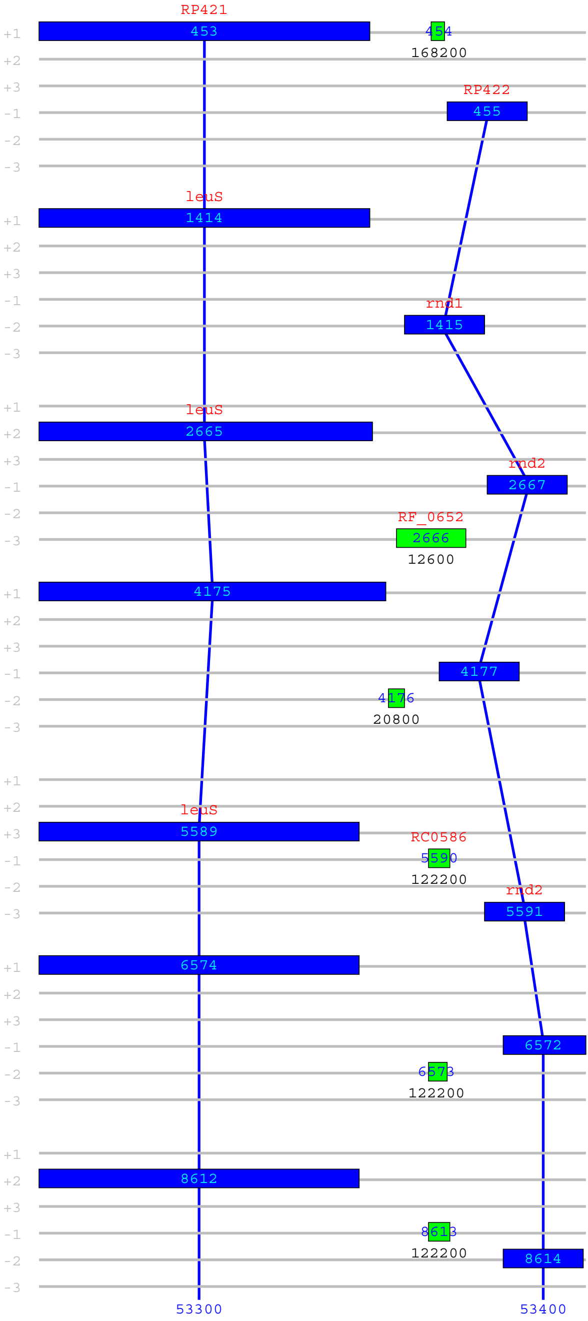

1 Rickettsia prowazekii str. Madrid E, complete genome
2 Rickettsia typhi str. wilmington, complete genome
3 Rickettsia felis URRWXCal2, complete genome
4 Rickettsia akari str. Hartford chromosome, whole genome shotgun sequence
5 Rickettsia conorii str. Malish 7, complete genome
6 Rickettsia sibirica 246 rsib_agncrt, whole genome shotgun sequence
7 Rickettsia rickettsii chromosome, whole genome shotgun sequence

Reg_id: 93

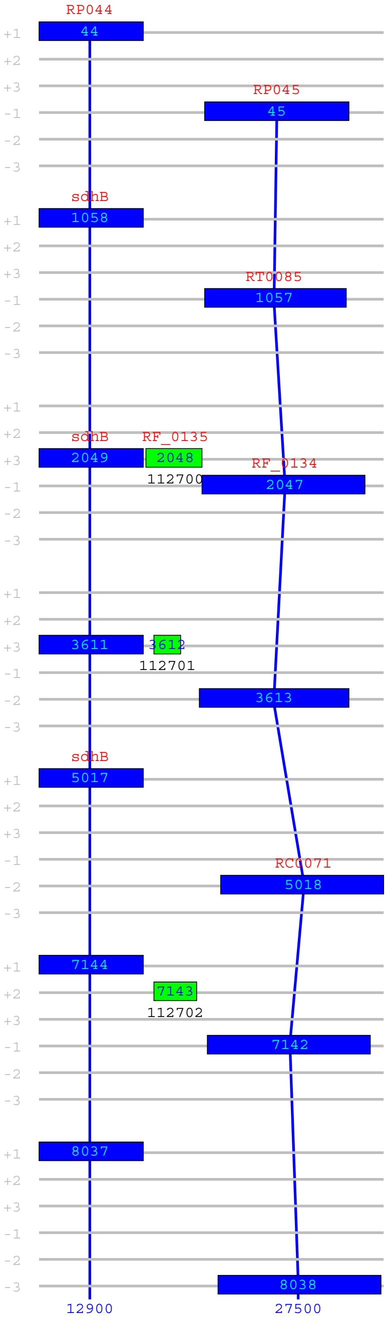

1 Rickettsia prowazekii str. Madrid E, complete genome
2 Rickettsia typhi str. wilmington, complete genome
3 Rickettsia felis URRWXCal2, complete genome
4 Rickettsia akari str. Hartford chromosome, whole genome shotgun sequence
5 Rickettsia conorii str. Malish 7, complete genome
6 Rickettsia sibirica 246 rsib_agncrt, whole genome shotgun sequence
7 Rickettsia rickettsii chromosome, whole genome shotgun sequence

Reg_id: 95

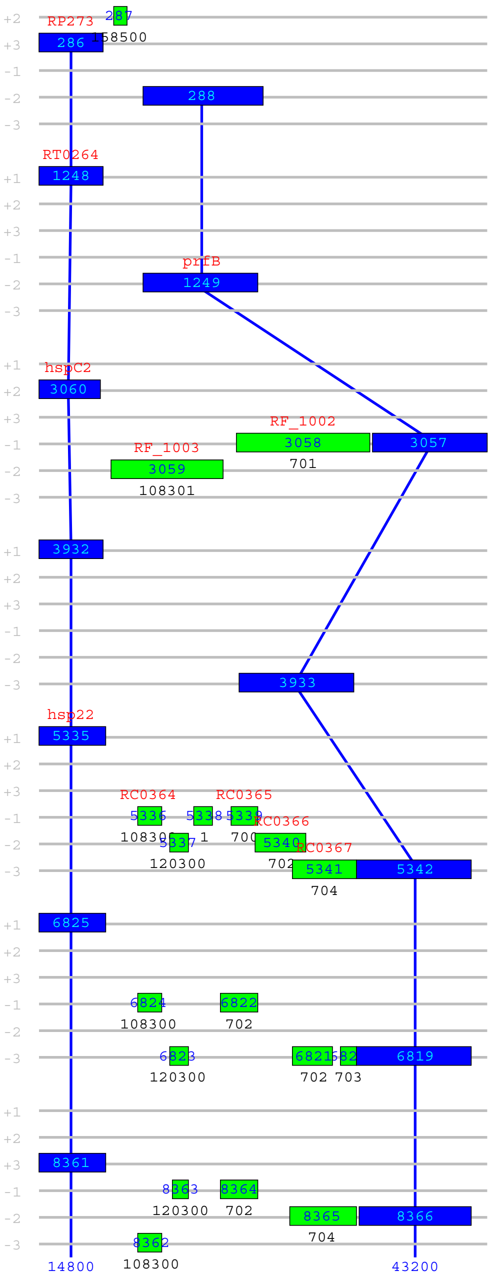

1 Rickettsia prowazekii str. Madrid E, complete genome
2 Rickettsia typhi str. wilmington, complete genome
3 Rickettsia felis URRWXCal2, complete genome
4 Rickettsia akari str. Hartford chromosome, whole genome shotgun sequence
5 Rickettsia conorii str. Malish 7, complete genome
6 Rickettsia sibirica 246 rsib_agncrt, whole genome shotgun sequence
7 Rickettsia rickettsii chromosome, whole genome shotgun sequence

Reg_id: 98

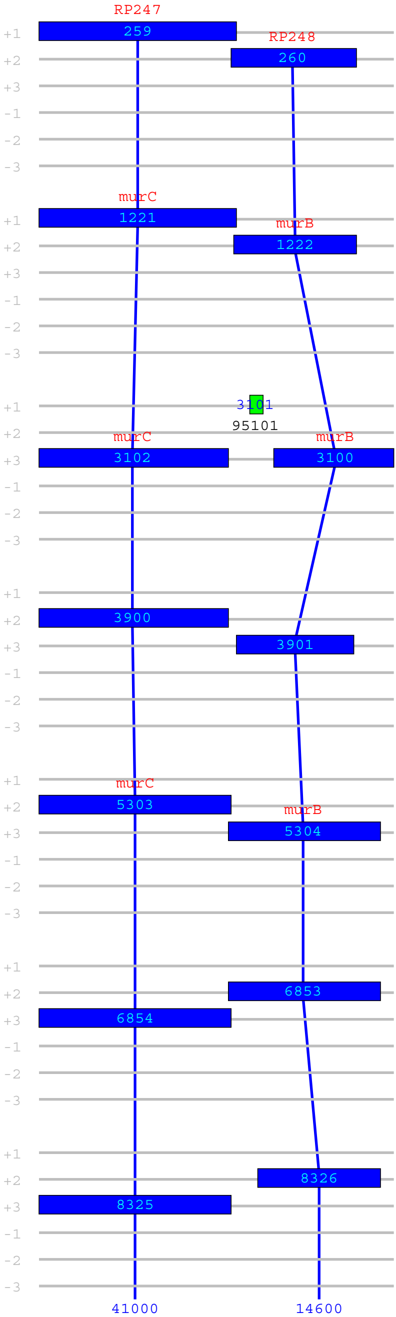

1 Rickettsia prowazekii str. Madrid E, complete genome
2 Rickettsia typhi str. wilmington, complete genome
3 Rickettsia felis URRWXCal2, complete genome
4 Rickettsia akari str. Hartford chromosome, whole genome shotgun sequence
5 Rickettsia conorii str. Malish 7, complete genome
6 Rickettsia sibirica 246 rsib_agncrt, whole genome shotgun sequence
7 Rickettsia rickettsii chromosome, whole genome shotgun sequence

Reg_id: 100

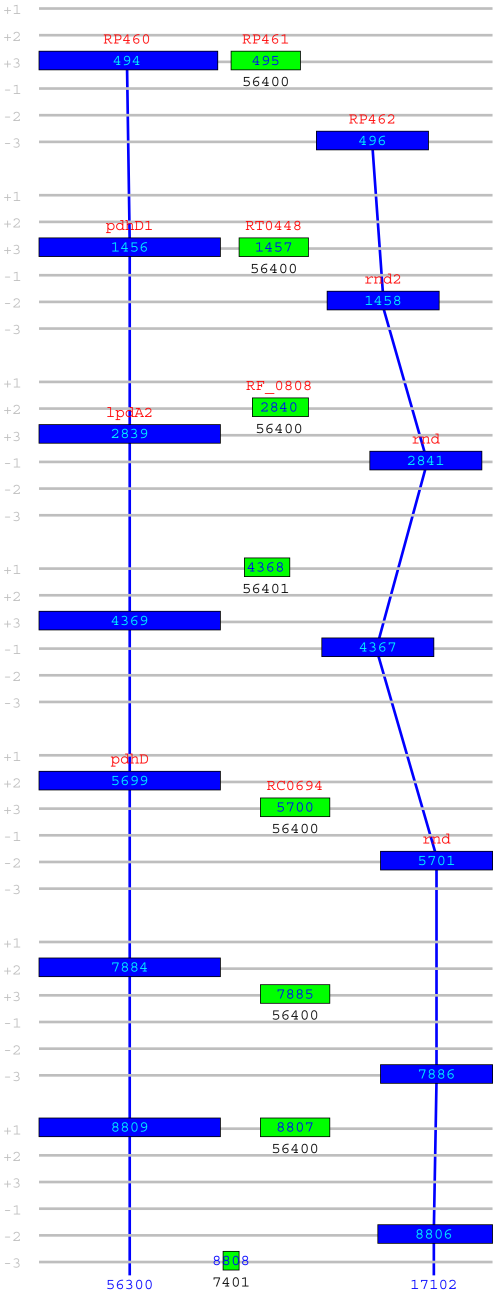

1 Rickettsia prowazekii str. Madrid E, complete genome
2 Rickettsia typhi str. wilmington, complete genome
3 Rickettsia felis URRWXCal2, complete genome
4 Rickettsia akari str. Hartford chromosome, whole genome shotgun sequence
5 Rickettsia conorii str. Malish 7, complete genome
6 Rickettsia sibirica 246 rsib_agncrt, whole genome shotgun sequence
7 Rickettsia rickettsii chromosome, whole genome shotgun sequence

Reg_id: 102

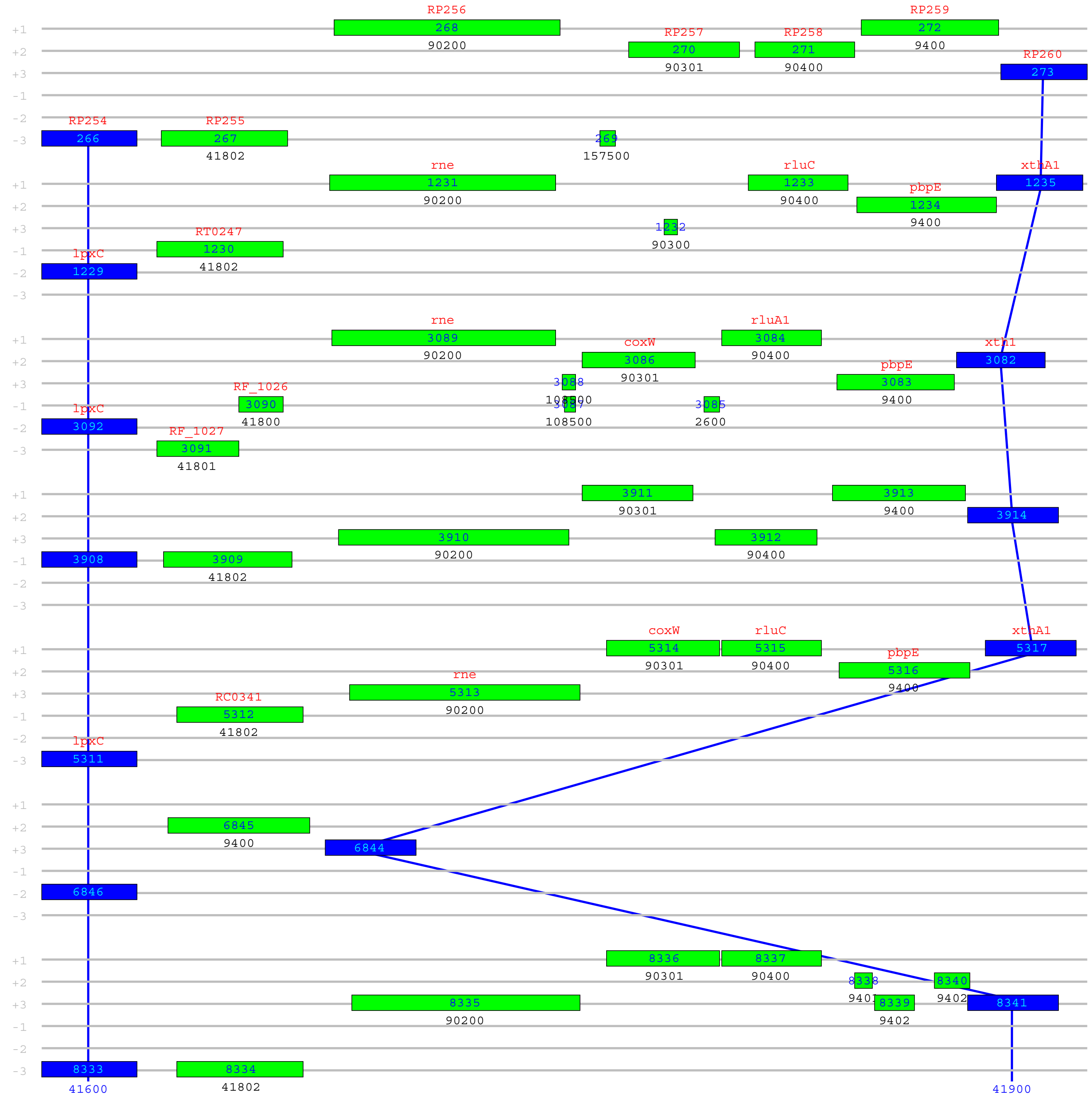

1 Rickettsia prowazekii str. Madrid E, complete genome
2 Rickettsia typhi str. wilmington, complete genome
3 Rickettsia felis URRWXCal2, complete genome
4 Rickettsia akari str. Hartford chromosome, whole genome shotgun sequence
5 Rickettsia conorii str. Malish 7, complete genome
6 Rickettsia sibirica 246 rsib_agncrt, whole genome shotgun sequence
7 Rickettsia rickettsii chromosome, whole genome shotgun sequence

Reg_id: 103

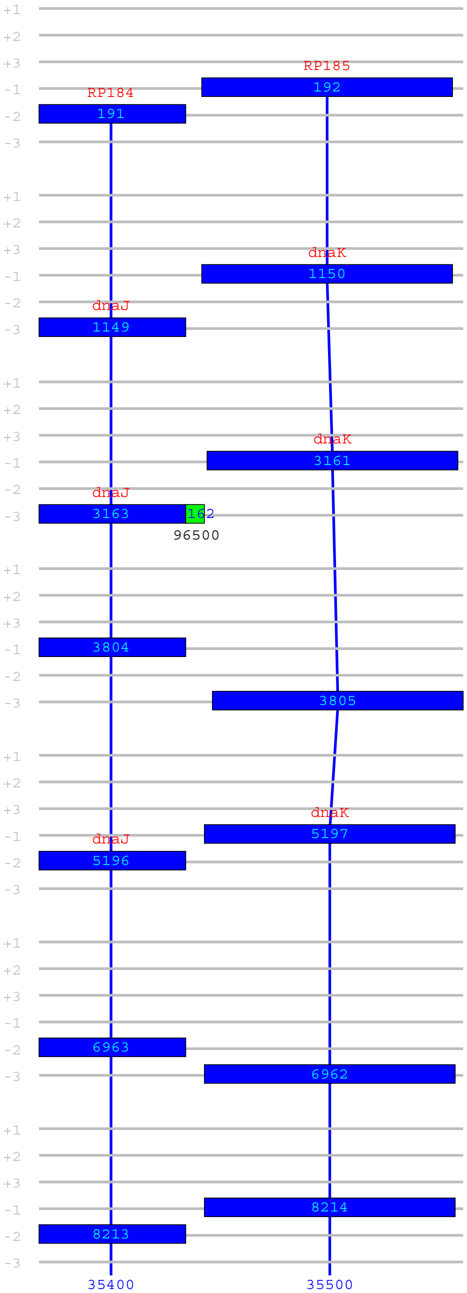

1 Rickettsia prowazekii str. Madrid E, complete genome
2 Rickettsia typhi str. wilmington, complete genome
3 Rickettsia felis URRWXCal2, complete genome
4 Rickettsia akari str. Hartford chromosome, whole genome shotgun sequence
5 Rickettsia conorii str. Malish 7, complete genome
6 Rickettsia sibirica 246 rsib_agncrt, whole genome shotgun sequence
7 Rickettsia rickettsii chromosome, whole genome shotgun sequence

Reg_id: 104

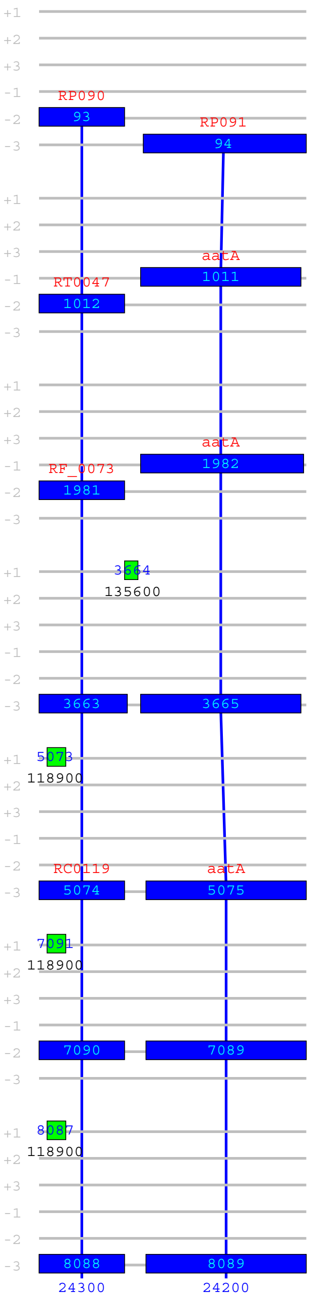

1 Rickettsia prowazekii str. Madrid E, complete genome
2 Rickettsia typhi str. wilmington, complete genome
3 Rickettsia felis URRWXCal2, complete genome
4 Rickettsia akari str. Hartford chromosome, whole genome shotgun sequence
5 Rickettsia conorii str. Malish 7, complete genome
6 Rickettsia sibirica 246 rsib_agncrt, whole genome shotgun sequence
7 Rickettsia rickettsii chromosome, whole genome shotgun sequence

Reg_id: 105

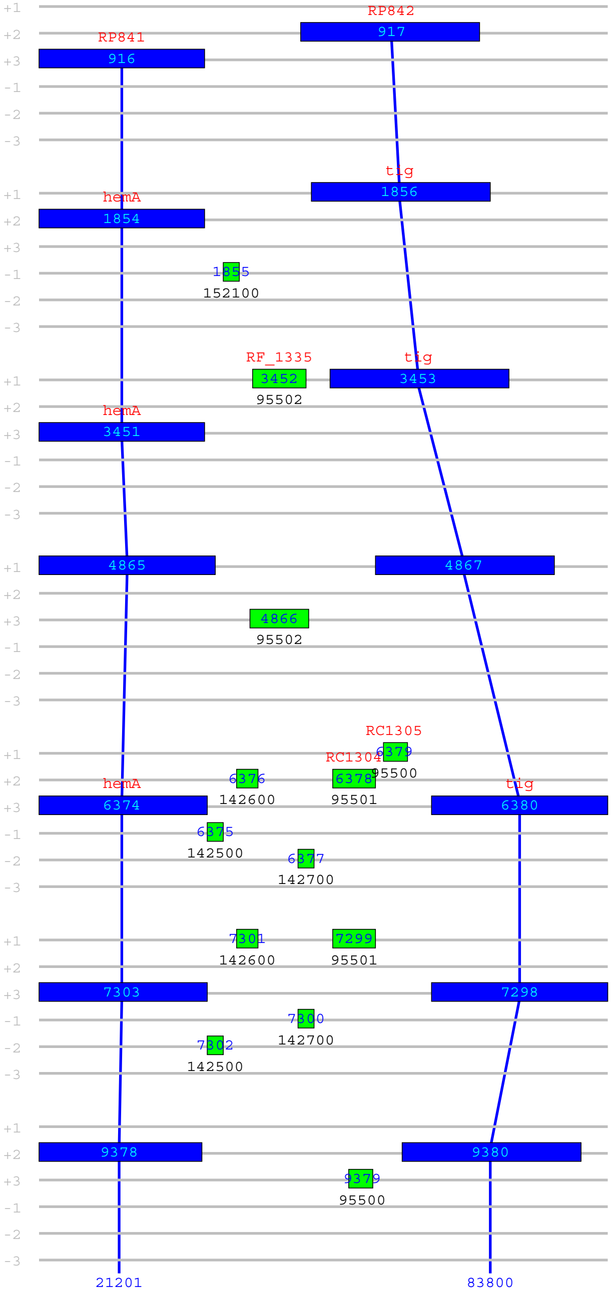

1 Rickettsia prowazekii str. Madrid E, complete genome
2 Rickettsia typhi str. wilmington, complete genome
3 Rickettsia felis URRWXCal2, complete genome
4 Rickettsia akari str. Hartford chromosome, whole genome shotgun sequence
5 Rickettsia conorii str. Malish 7, complete genome
6 Rickettsia sibirica 246 rsib_agncrt, whole genome shotgun sequence
7 Rickettsia rickettsii chromosome, whole genome shotgun sequence

Reg_id: 106

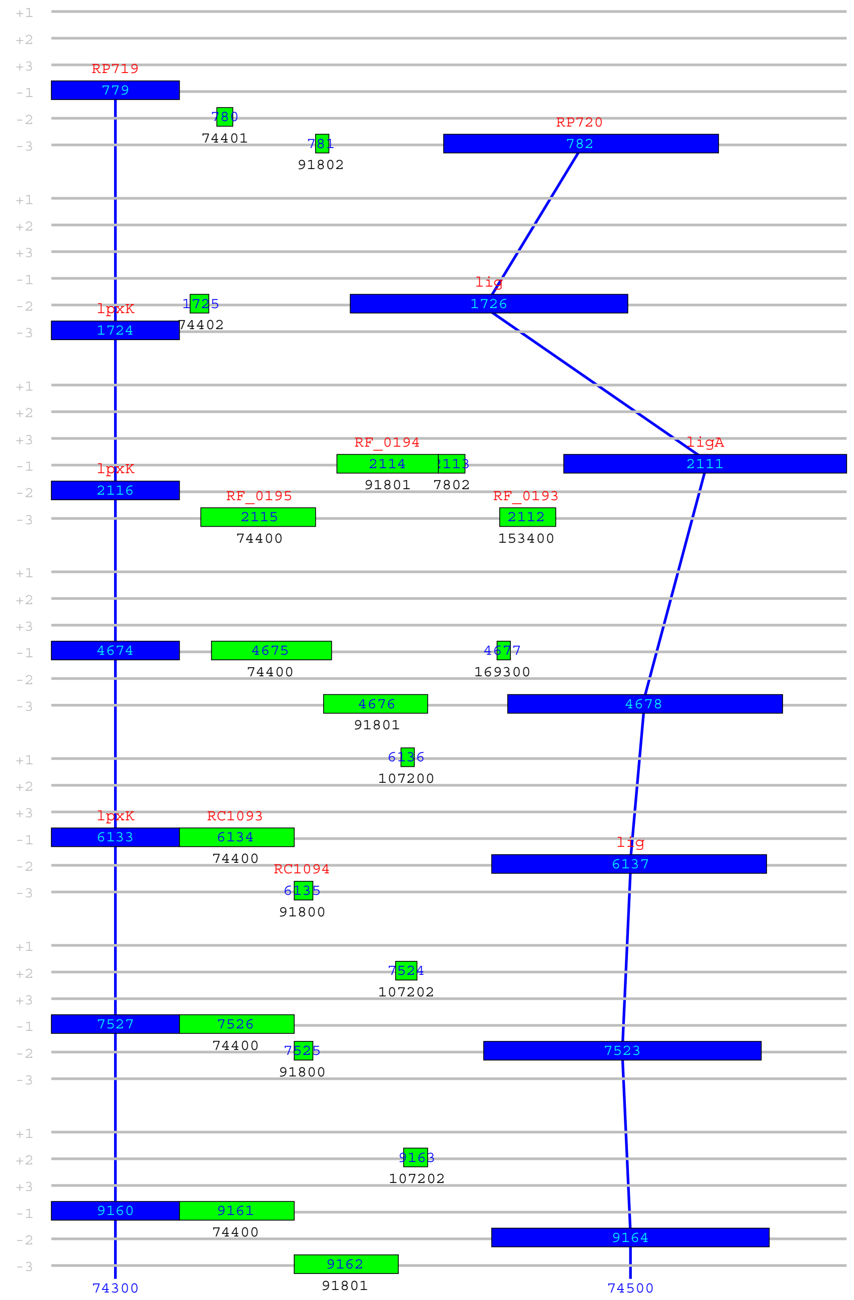

1 Rickettsia prowazekii str. Madrid E, complete genome
2 Rickettsia typhi str. wilmington, complete genome
3 Rickettsia felis URRWXCal2, complete genome
4 Rickettsia akari str. Hartford chromosome, whole genome shotgun sequence
5 Rickettsia conorii str. Malish 7, complete genome
6 Rickettsia sibirica 246 rsib_agncrt, whole genome shotgun sequence
7 Rickettsia rickettsii chromosome, whole genome shotgun sequence

Reg_id: 108

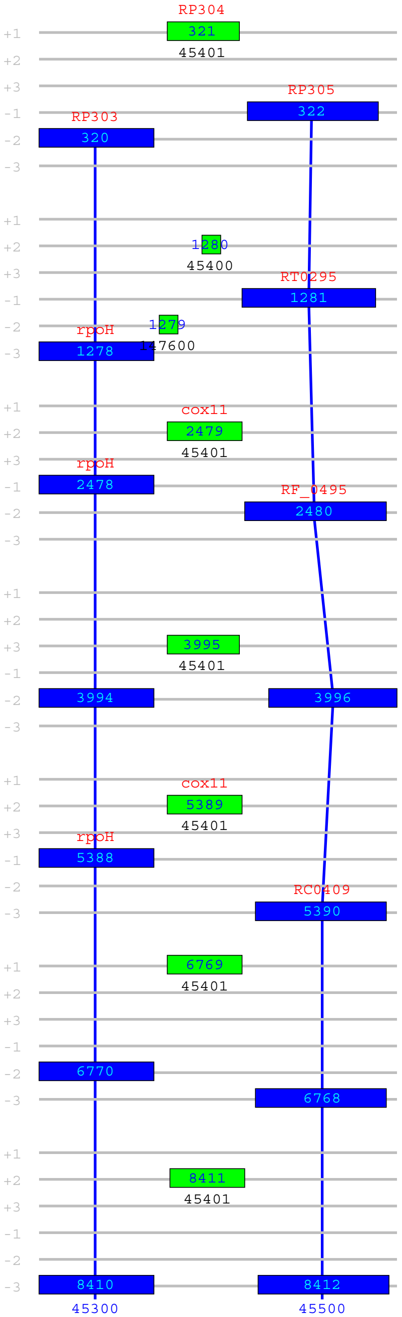

1 Rickettsia prowazekii str. Madrid E, complete genome
2 Rickettsia typhi str. wilmington, complete genome
3 Rickettsia felis URRWXCal2, complete genome
4 Rickettsia akari str. Hartford chromosome, whole genome shotgun sequence
5 Rickettsia conorii str. Malish 7, complete genome
6 Rickettsia sibirica 246 rsib_agncrt, whole genome shotgun sequence
7 Rickettsia rickettsii chromosome, whole genome shotgun sequence

Reg_id: 110

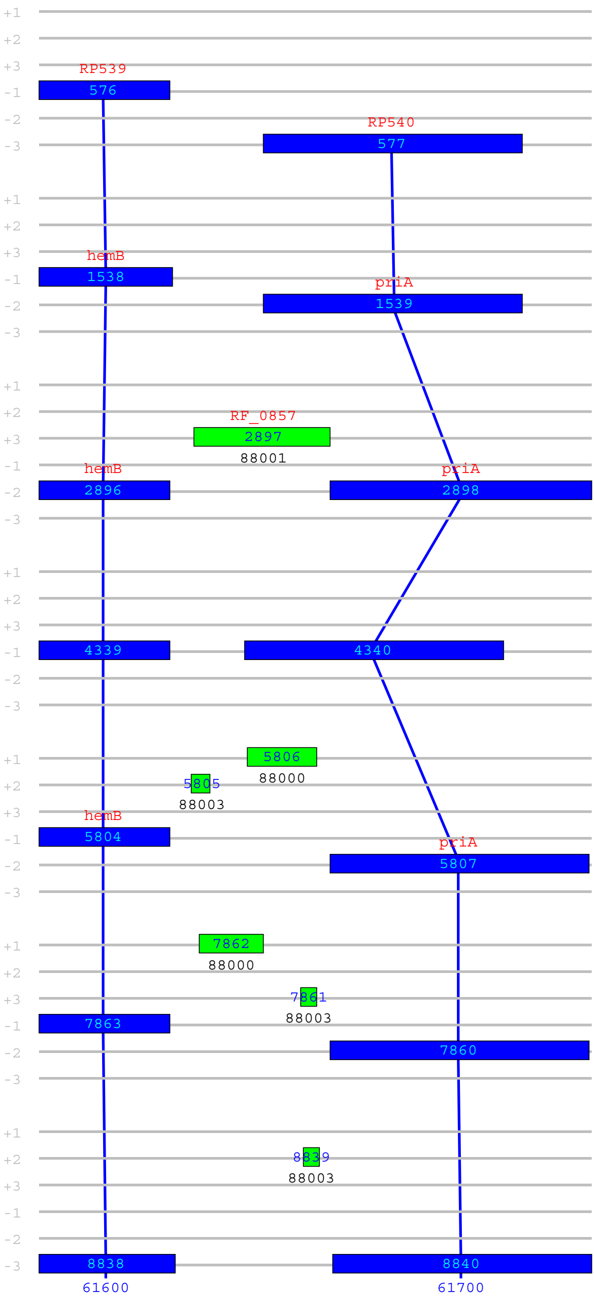

1 Rickettsia prowazekii str. Madrid E, complete genome
2 Rickettsia typhi str. wilmington, complete genome
3 Rickettsia felis URRWXCal2, complete genome
4 Rickettsia akari str. Hartford chromosome, whole genome shotgun sequence
5 Rickettsia conorii str. Malish 7, complete genome
6 Rickettsia sibirica 246 rsib_agncrt, whole genome shotgun sequence
7 Rickettsia rickettsii chromosome, whole genome shotgun sequence

Reg_id: 113

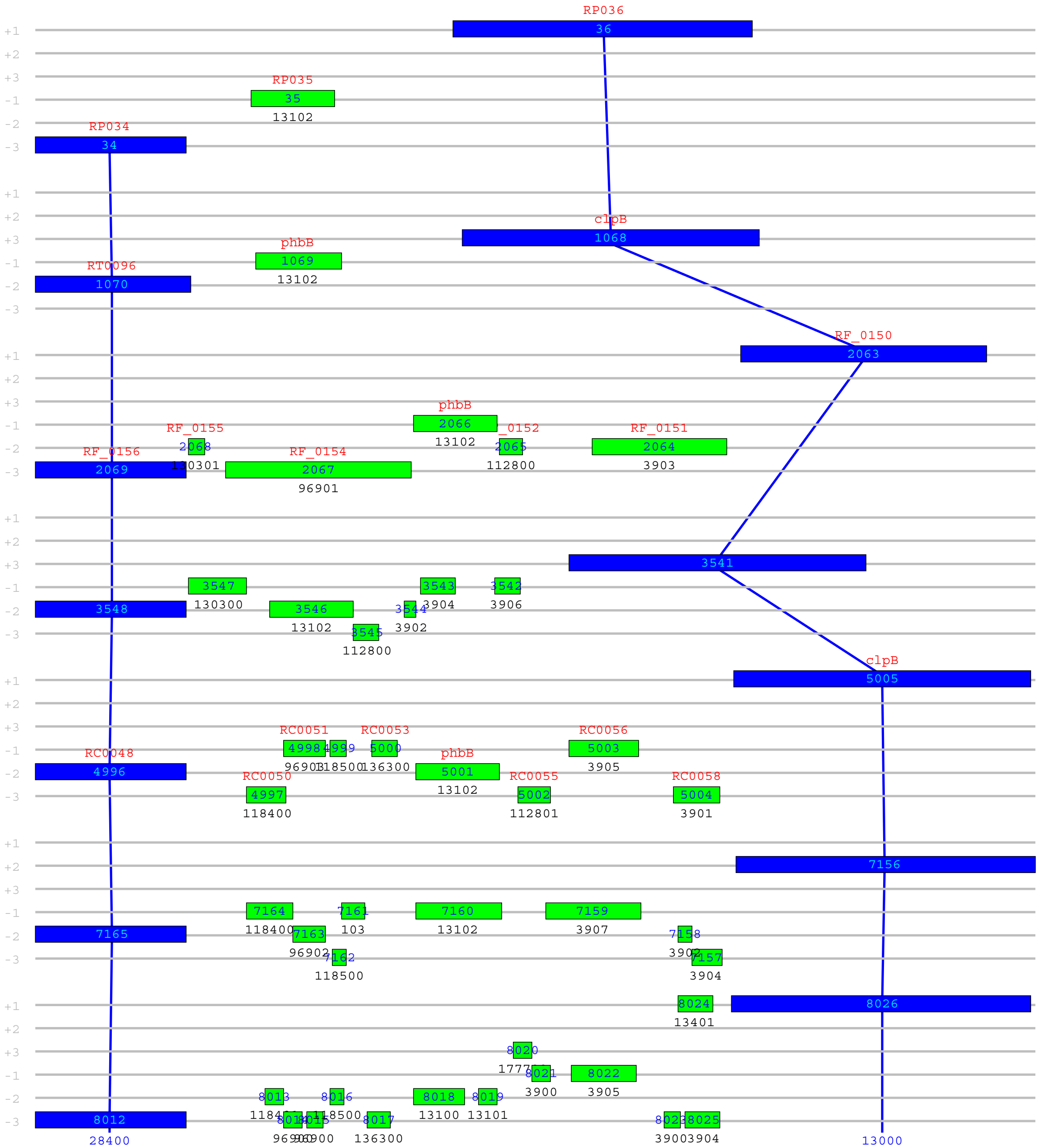

1 Rickettsia prowazekii str. Madrid E, complete genome
2 Rickettsia typhi str. wilmington, complete genome
3 Rickettsia felis URRWXCal2, complete genome
4 Rickettsia akari str. Hartford chromosome, whole genome shotgun sequence
5 Rickettsia conorii str. Malish 7, complete genome
6 Rickettsia sibirica 246 rsib_agncrt, whole genome shotgun sequence
7 Rickettsia rickettsii chromosome, whole genome shotgun sequence

Reg_id: 115

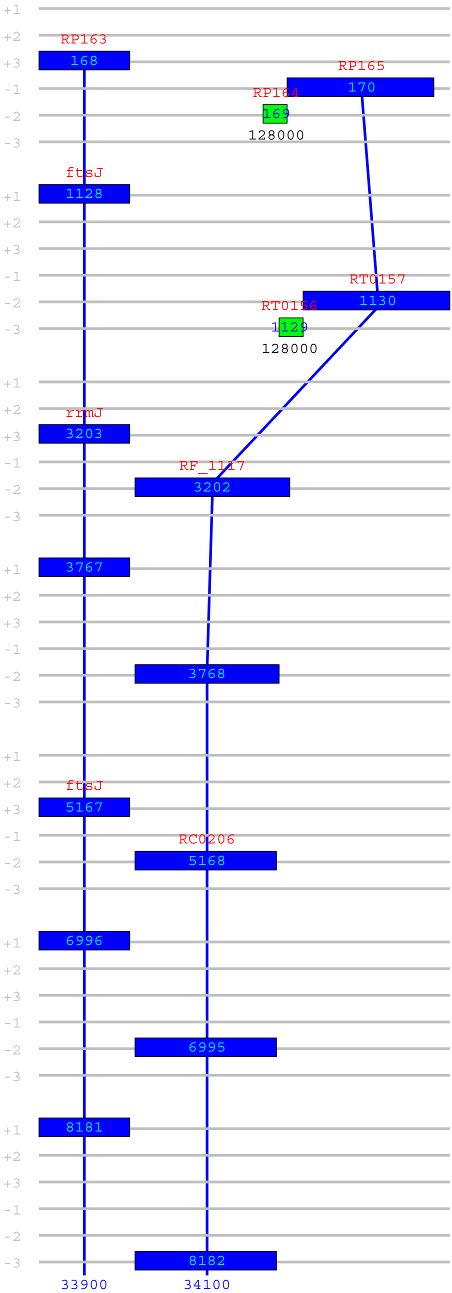

1 Rickettsia prowazekii str. Madrid E, complete genome
2 Rickettsia typhi str. wilmington, complete genome
3 Rickettsia felis URRWXCal2, complete genome
4 Rickettsia akari str. Hartford chromosome, whole genome shotgun sequence
5 Rickettsia conorii str. Malish 7, complete genome
6 Rickettsia sibirica 246 rsib_agncrt, whole genome shotgun sequence
7 Rickettsia rickettsii chromosome, whole genome shotgun sequence

Reg_id: 116

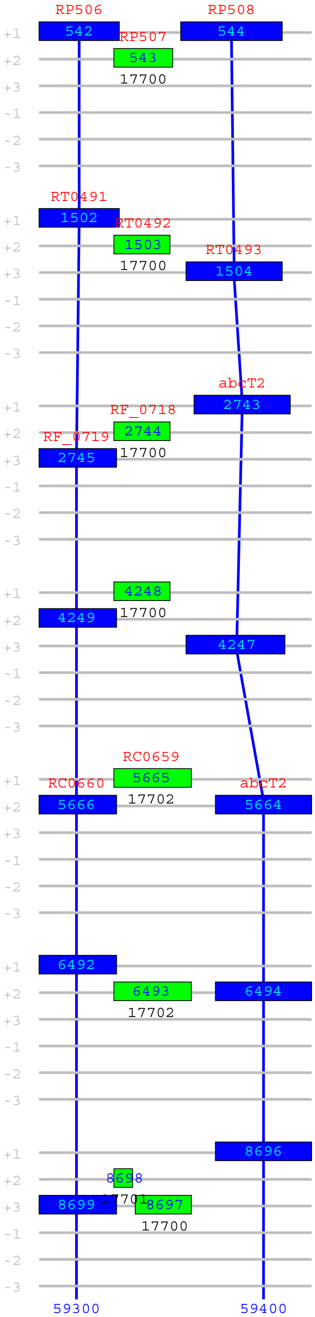

1 Rickettsia prowazekii str. Madrid E, complete genome
2 Rickettsia typhi str. wilmington, complete genome
3 Rickettsia felis URRWXCal2, complete genome
4 Rickettsia akari str. Hartford chromosome, whole genome shotgun sequence
5 Rickettsia conorii str. Malish 7, complete genome
6 Rickettsia sibirica 246 rsib_agncrt, whole genome shotgun sequence
7 Rickettsia rickettsii chromosome, whole genome shotgun sequence

Reg_id: 118

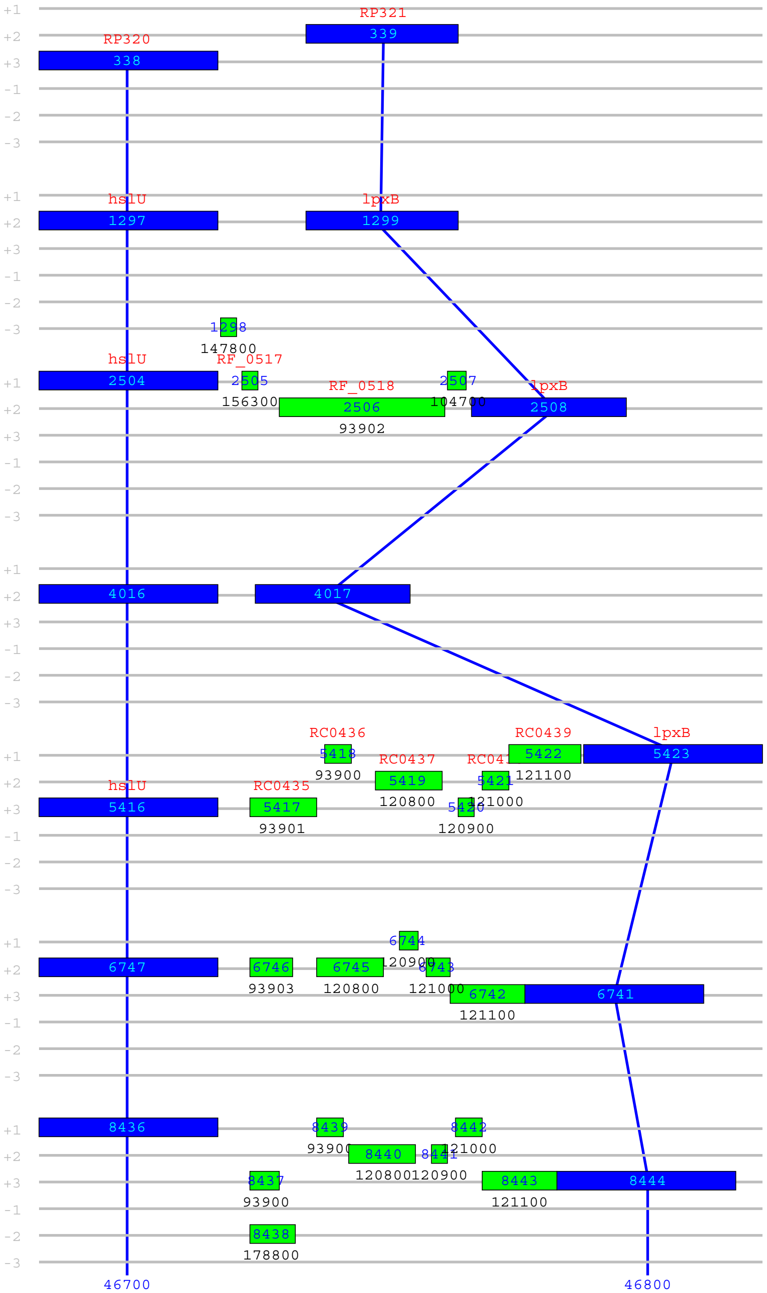

1 Rickettsia prowazekii str. Madrid E, complete genome
2 Rickettsia typhi str. wilmington, complete genome
3 Rickettsia felis URRWXCal2, complete genome
4 Rickettsia akari str. Hartford chromosome, whole genome shotgun sequence
5 Rickettsia conorii str. Malish 7, complete genome
6 Rickettsia sibirica 246 rsib_agncrt, whole genome shotgun sequence
7 Rickettsia rickettsii chromosome, whole genome shotgun sequence

Reg_id: 119

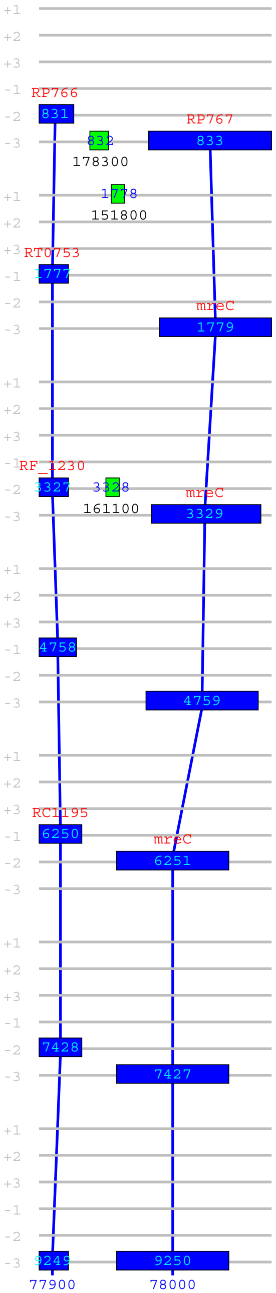

1 Rickettsia prowazekii str. Madrid E, complete genome
2 Rickettsia typhi str. wilmington, complete genome
3 Rickettsia felis URRWXCal2, complete genome
4 Rickettsia akari str. Hartford chromosome, whole genome shotgun sequence
5 Rickettsia conorii str. Malish 7, complete genome
6 Rickettsia sibirica 246 rsib_agncrt, whole genome shotgun sequence
7 Rickettsia rickettsii chromosome, whole genome shotgun sequence

Reg_id: 125

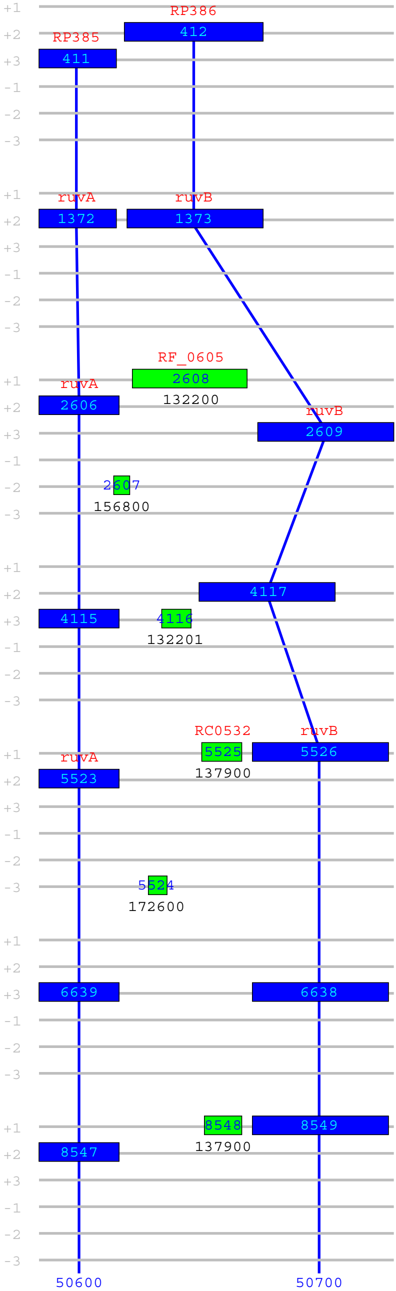

1 Rickettsia prowazekii str. Madrid E, complete genome
2 Rickettsia typhi str. wilmington, complete genome
3 Rickettsia felis URRWXCal2, complete genome
4 Rickettsia akari str. Hartford chromosome, whole genome shotgun sequence
5 Rickettsia conorii str. Malish 7, complete genome
6 Rickettsia sibirica 246 rsib_agncrt, whole genome shotgun sequence
7 Rickettsia rickettsii chromosome, whole genome shotgun sequence

Reg_id: 126

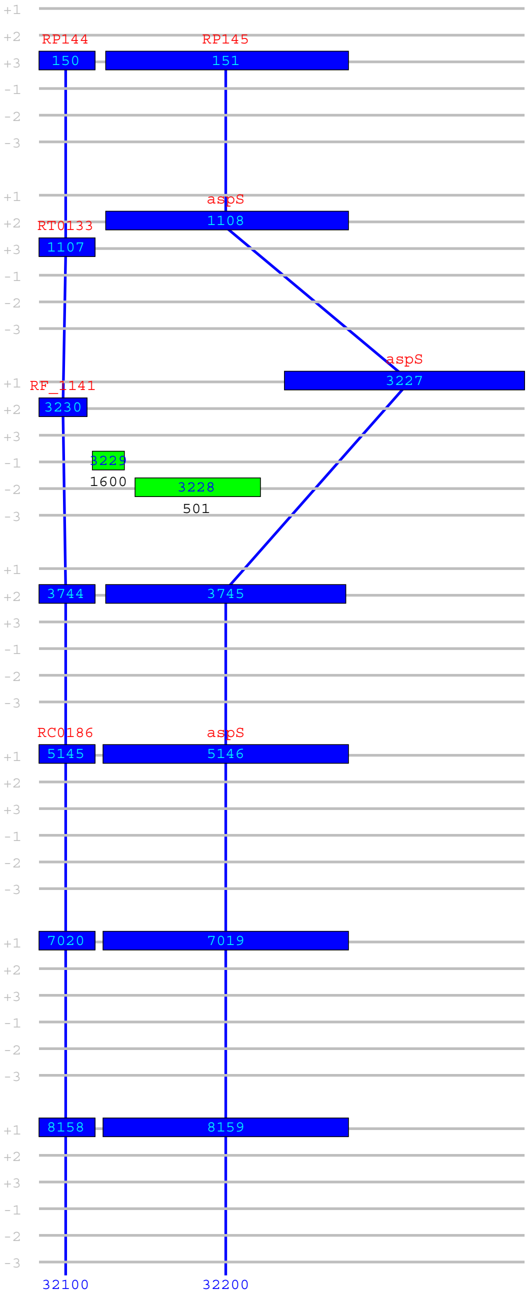

1 Rickettsia prowazekii str. Madrid E, complete genome
2 Rickettsia typhi str. wilmington, complete genome
3 Rickettsia felis URRWXCal2, complete genome
4 Rickettsia akari str. Hartford chromosome, whole genome shotgun sequence
5 Rickettsia conorii str. Malish 7, complete genome
6 Rickettsia sibirica 246 rsib_agncrt, whole genome shotgun sequence
7 Rickettsia rickettsii chromosome, whole genome shotgun sequence

Reg_id: 127

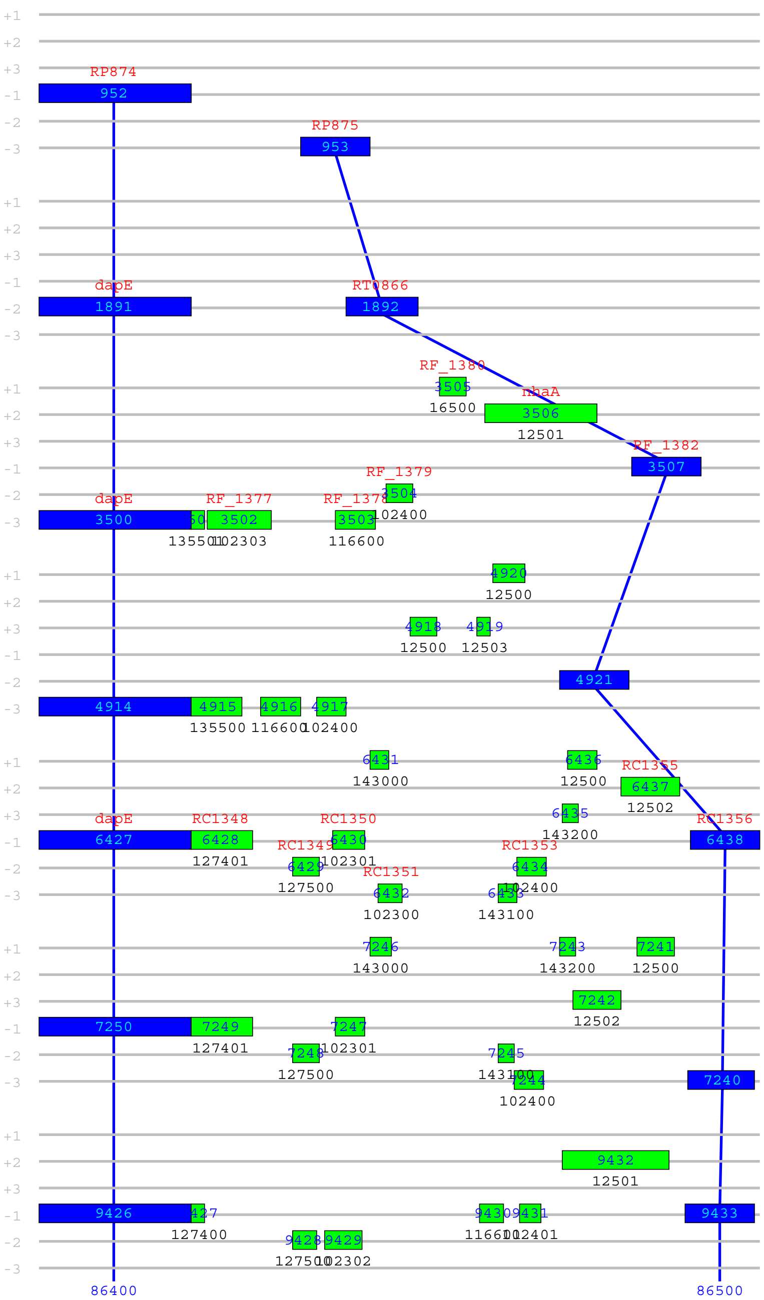

1 Rickettsia prowazekii str. Madrid E, complete genome
2 Rickettsia typhi str. wilmington, complete genome
3 Rickettsia felis URRWXCal2, complete genome
4 Rickettsia akari str. Hartford chromosome, whole genome shotgun sequence
5 Rickettsia conorii str. Malish 7, complete genome
6 Rickettsia sibirica 246 rsib_agncrt, whole genome shotgun sequence
7 Rickettsia rickettsii chromosome, whole genome shotgun sequence

Reg_id: 128

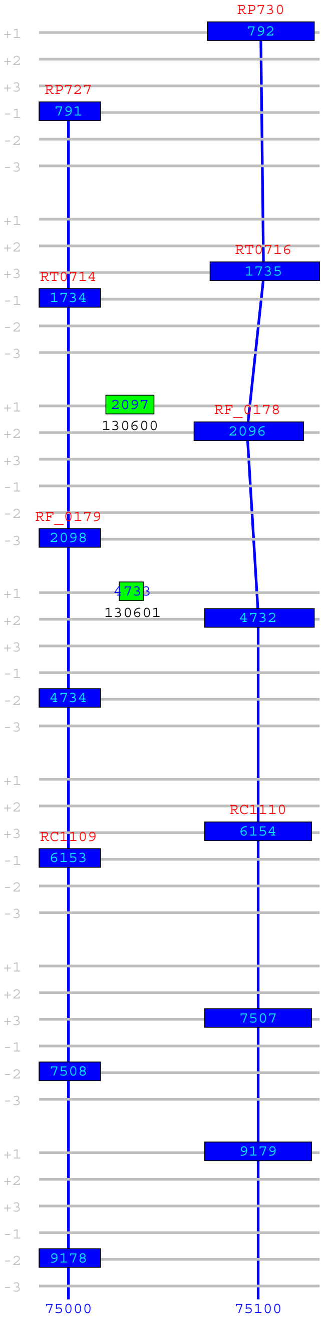

1 Rickettsia prowazekii str. Madrid E, complete genome
2 Rickettsia typhi str. wilmington, complete genome
3 Rickettsia felis URRWXCal2, complete genome
4 Rickettsia akari str. Hartford chromosome, whole genome shotgun sequence
5 Rickettsia conorii str. Malish 7, complete genome
6 Rickettsia sibirica 246 rsib_agncrt, whole genome shotgun sequence
7 Rickettsia rickettsii chromosome, whole genome shotgun sequence

Reg_id: 129

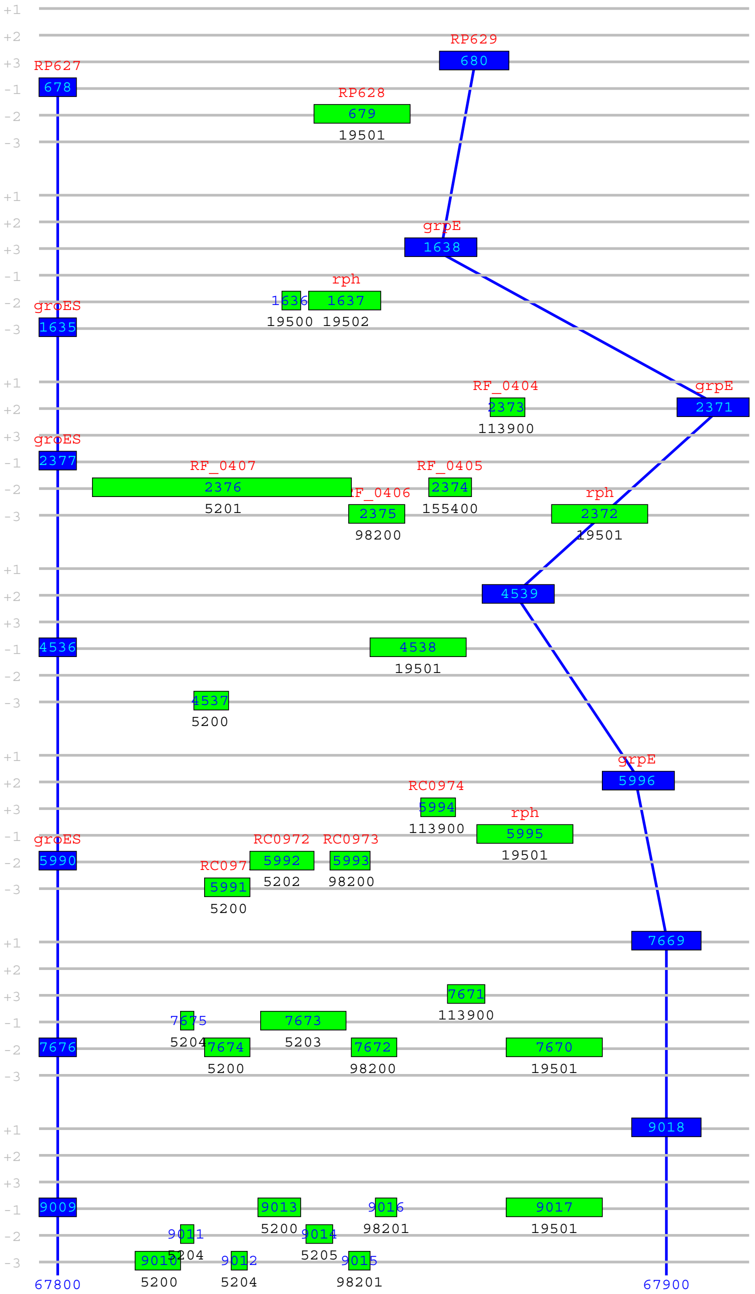

1 Rickettsia prowazekii str. Madrid E, complete genome
2 Rickettsia typhi str. wilmington, complete genome
3 Rickettsia felis URRWXCal2, complete genome
4 Rickettsia akari str. Hartford chromosome, whole genome shotgun sequence
5 Rickettsia conorii str. Malish 7, complete genome
6 Rickettsia sibirica 246 rsib_agncrt, whole genome shotgun sequence
7 Rickettsia rickettsii chromosome, whole genome shotgun sequence

Reg_id: 130

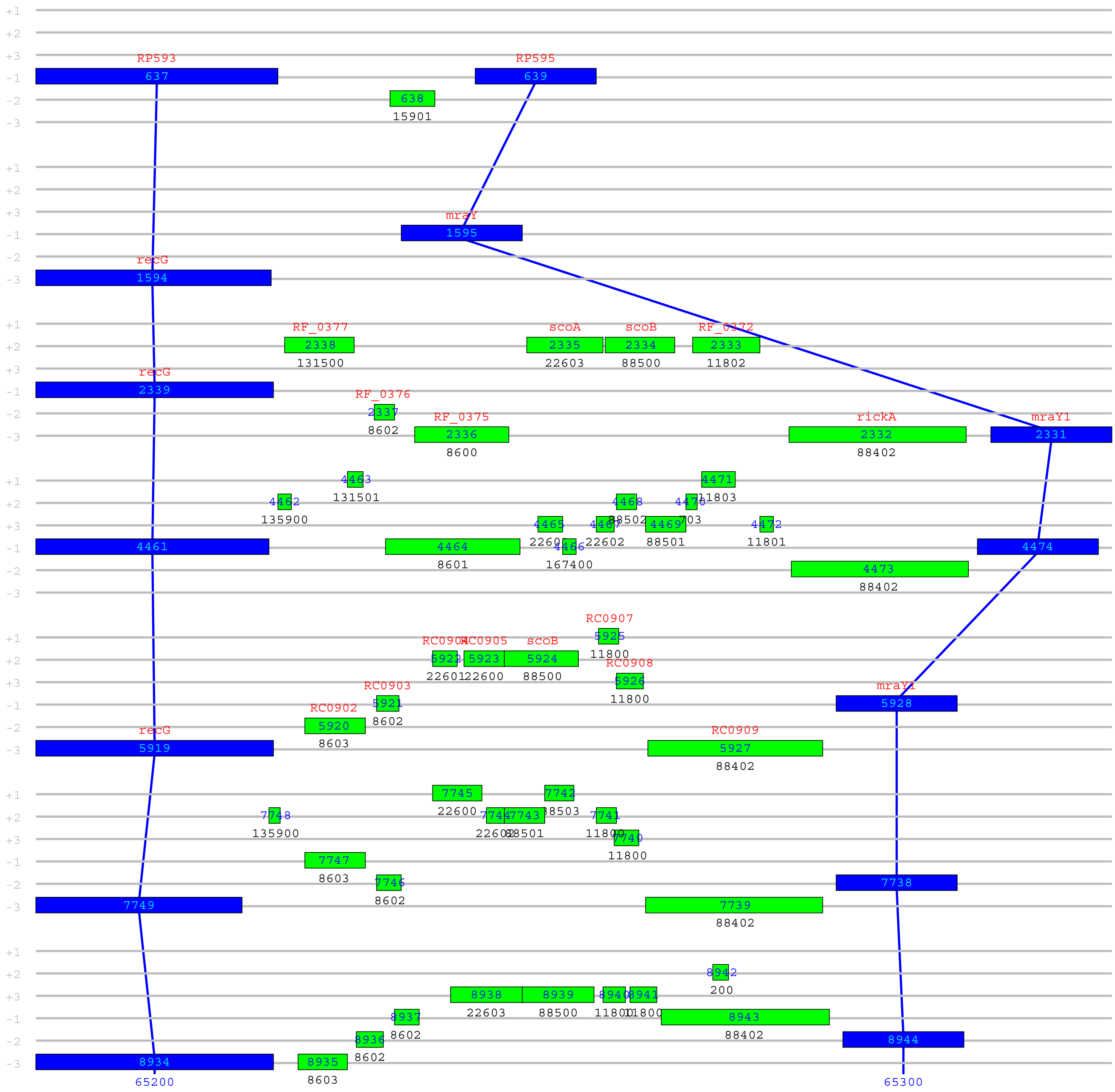

1 Rickettsia prowazekii str. Madrid E, complete genome
2 Rickettsia typhi str. wilmington, complete genome
3 Rickettsia felis URRWXCal2, complete genome
4 Rickettsia akari str. Hartford chromosome, whole genome shotgun sequence
5 Rickettsia conorii str. Malish 7, complete genome
6 Rickettsia sibirica 246 rsib_agncrt, whole genome shotgun sequence
7 Rickettsia rickettsii chromosome, whole genome shotgun sequence

Reg_id: 131

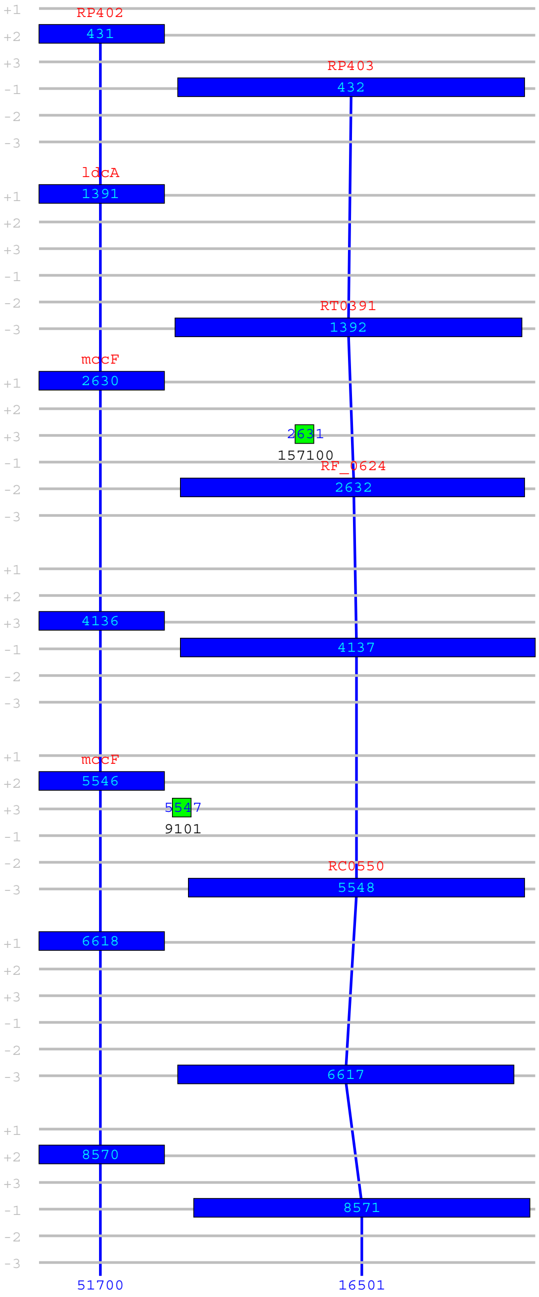

1 Rickettsia prowazekii str. Madrid E, complete genome
2 Rickettsia typhi str. wilmington, complete genome
3 Rickettsia felis URRWXCal2, complete genome
4 Rickettsia akari str. Hartford chromosome, whole genome shotgun sequence
5 Rickettsia conorii str. Malish 7, complete genome
6 Rickettsia sibirica 246 rsib_agncrt, whole genome shotgun sequence
7 Rickettsia rickettsii chromosome, whole genome shotgun sequence

Reg_id: 134

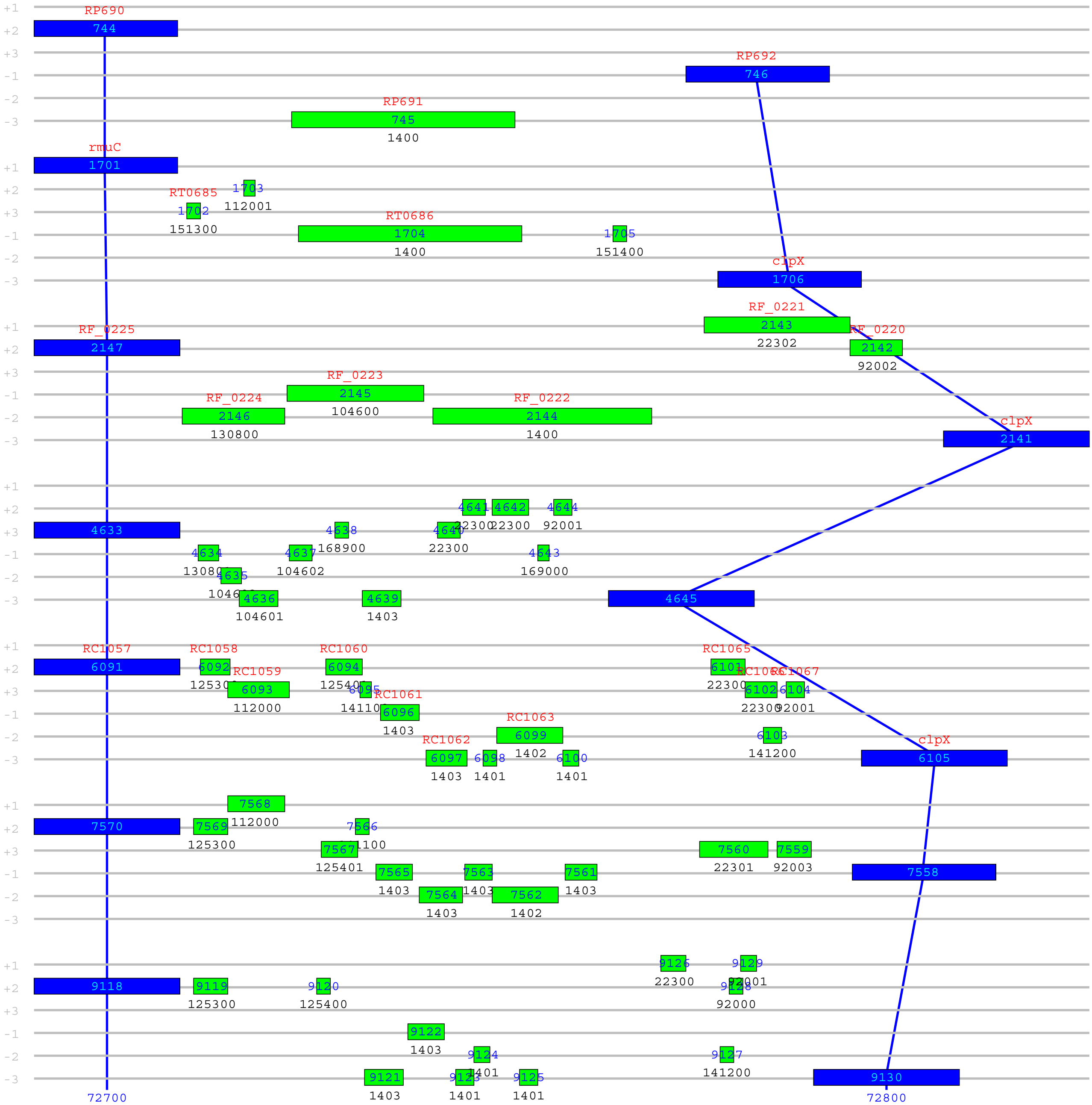

1 Rickettsia prowazekii str. Madrid E, complete genome
2 Rickettsia typhi str. wilmington, complete genome
3 Rickettsia felis URRWXCal2, complete genome
4 Rickettsia akari str. Hartford chromosome, whole genome shotgun sequence
5 Rickettsia conorii str. Malish 7, complete genome
6 Rickettsia sibirica 246 rsib_agncrt, whole genome shotgun sequence
7 Rickettsia rickettsii chromosome, whole genome shotgun sequence

Reg_id: 136

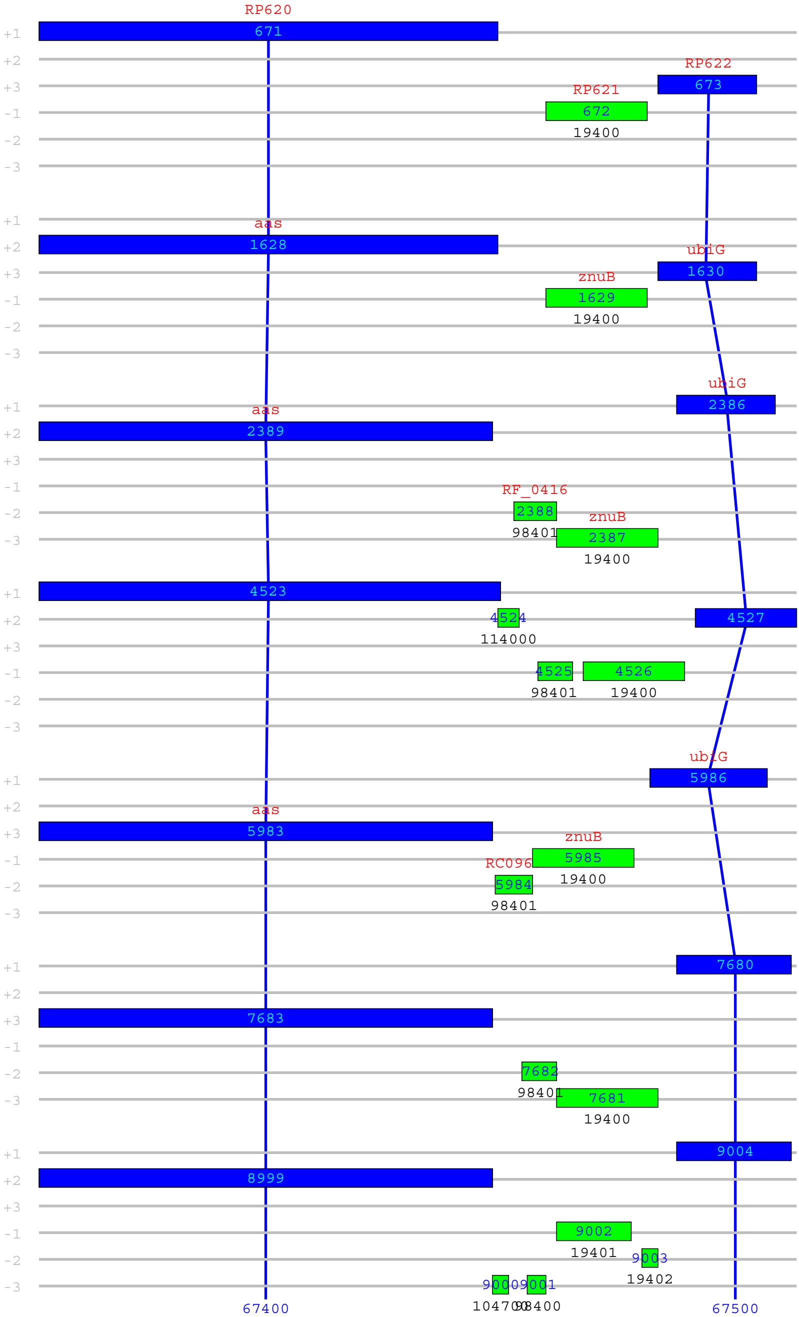

1 Rickettsia prowazekii str. Madrid E, complete genome
2 Rickettsia typhi str. wilmington, complete genome
3 Rickettsia felis URRWXCal2, complete genome
4 Rickettsia akari str. Hartford chromosome, whole genome shotgun sequence
5 Rickettsia conorii str. Malish 7, complete genome
6 Rickettsia sibirica 246 rsib_agncrt, whole genome shotgun sequence
7 Rickettsia rickettsii chromosome, whole genome shotgun sequence

Reg_id: 137

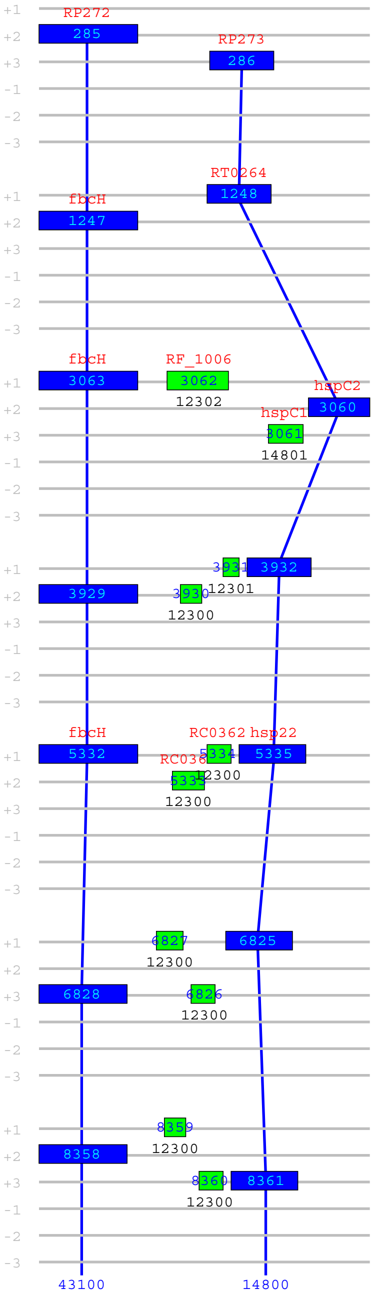

1 Rickettsia prowazekii str. Madrid E, complete genome
2 Rickettsia typhi str. wilmington, complete genome
3 Rickettsia felis URRWXCal2, complete genome
4 Rickettsia akari str. Hartford chromosome, whole genome shotgun sequence
5 Rickettsia conorii str. Malish 7, complete genome
6 Rickettsia sibirica 246 rsib_agncrt, whole genome shotgun sequence
7 Rickettsia rickettsii chromosome, whole genome shotgun sequence

Reg_id: 138

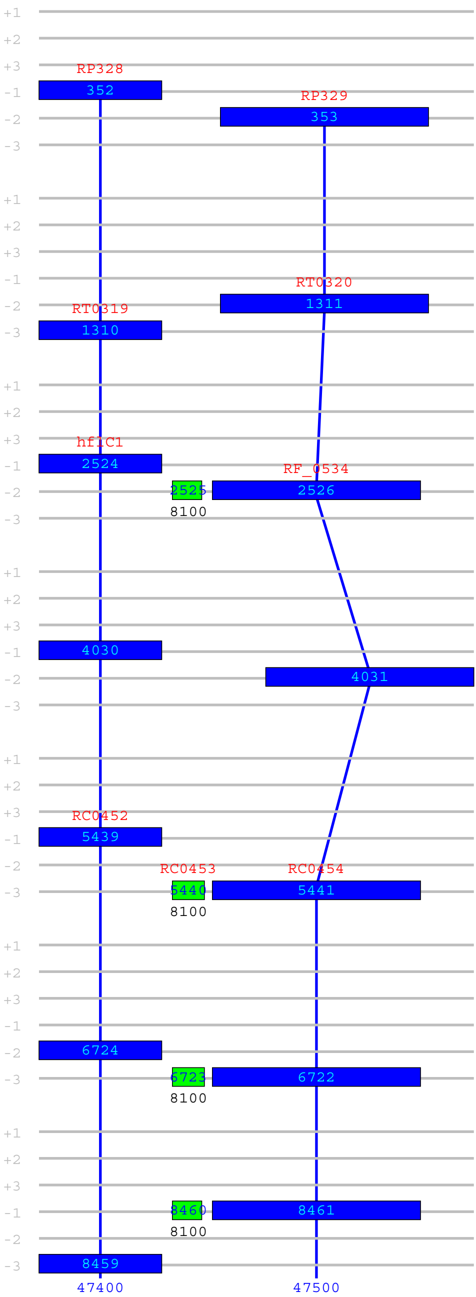

1 Rickettsia prowazekii str. Madrid E, complete genome
2 Rickettsia typhi str. wilmington, complete genome
3 Rickettsia felis URRWXCal2, complete genome
4 Rickettsia akari str. Hartford chromosome, whole genome shotgun sequence
5 Rickettsia conorii str. Malish 7, complete genome
6 Rickettsia sibirica 246 rsib_agncrt, whole genome shotgun sequence
7 Rickettsia rickettsii chromosome, whole genome shotgun sequence

Reg_id: 139

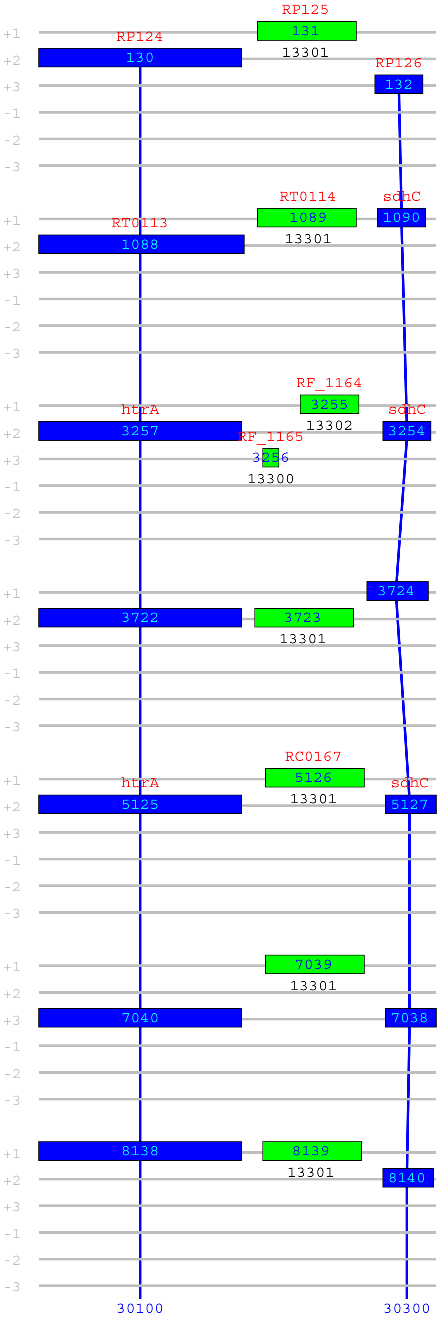

1 Rickettsia prowazekii str. Madrid E, complete genome
2 Rickettsia typhi str. wilmington, complete genome
3 Rickettsia felis URRWXCal2, complete genome
4 Rickettsia akari str. Hartford chromosome, whole genome shotgun sequence
5 Rickettsia conorii str. Malish 7, complete genome
6 Rickettsia sibirica 246 rsib_agncrt, whole genome shotgun sequence
7 Rickettsia rickettsii chromosome, whole genome shotgun sequence

Reg_id: 143

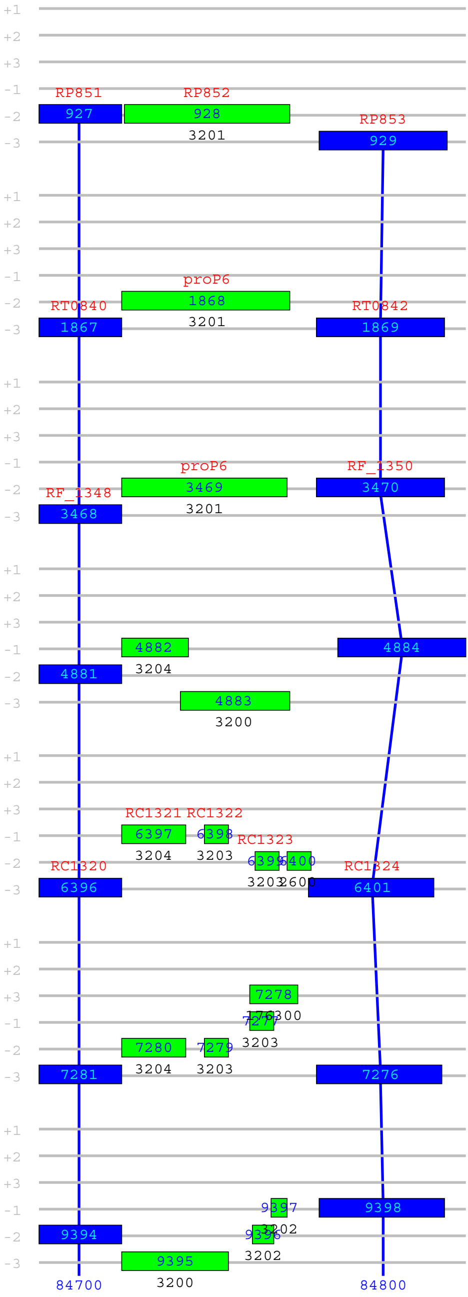

1 Rickettsia prowazekii str. Madrid E, complete genome
2 Rickettsia typhi str. wilmington, complete genome
3 Rickettsia felis URRWXCal2, complete genome
4 Rickettsia akari str. Hartford chromosome, whole genome shotgun sequence
5 Rickettsia conorii str. Malish 7, complete genome
6 Rickettsia sibirica 246 rsib_agncrt, whole genome shotgun sequence
7 Rickettsia rickettsii chromosome, whole genome shotgun sequence

Reg_id: 144

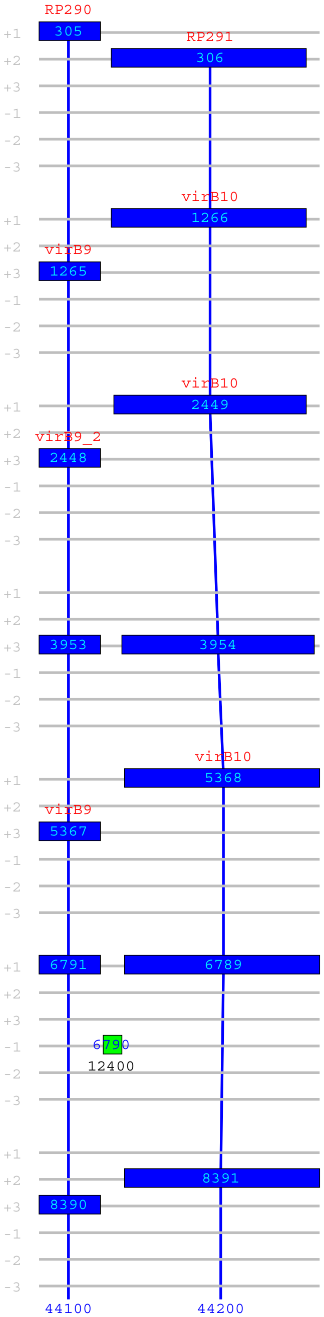

1 Rickettsia prowazekii str. Madrid E, complete genome
2 Rickettsia typhi str. wilmington, complete genome
3 Rickettsia felis URRWXCal2, complete genome
4 Rickettsia akari str. Hartford chromosome, whole genome shotgun sequence
5 Rickettsia conorii str. Malish 7, complete genome
6 Rickettsia sibirica 246 rsib_agncrt, whole genome shotgun sequence
7 Rickettsia rickettsii chromosome, whole genome shotgun sequence

Reg_id: 149

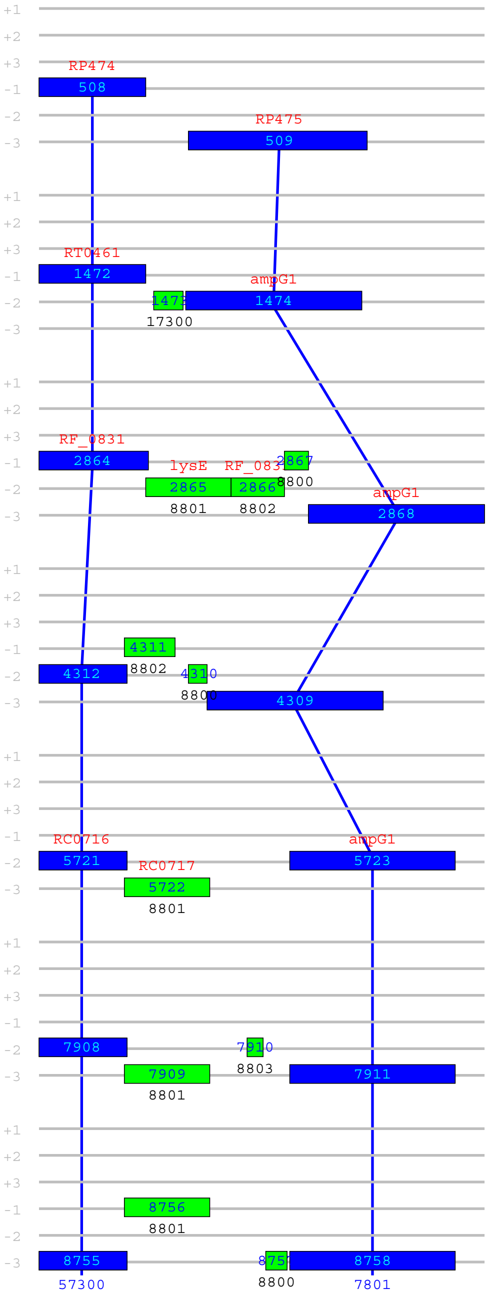

1 Rickettsia prowazekii str. Madrid E, complete genome
2 Rickettsia typhi str. wilmington, complete genome
3 Rickettsia felis URRWXCal2, complete genome
4 Rickettsia akari str. Hartford chromosome, whole genome shotgun sequence
5 Rickettsia conorii str. Malish 7, complete genome
6 Rickettsia sibirica 246 rsib_agncrt, whole genome shotgun sequence
7 Rickettsia rickettsii chromosome, whole genome shotgun sequence

Reg_id: 151

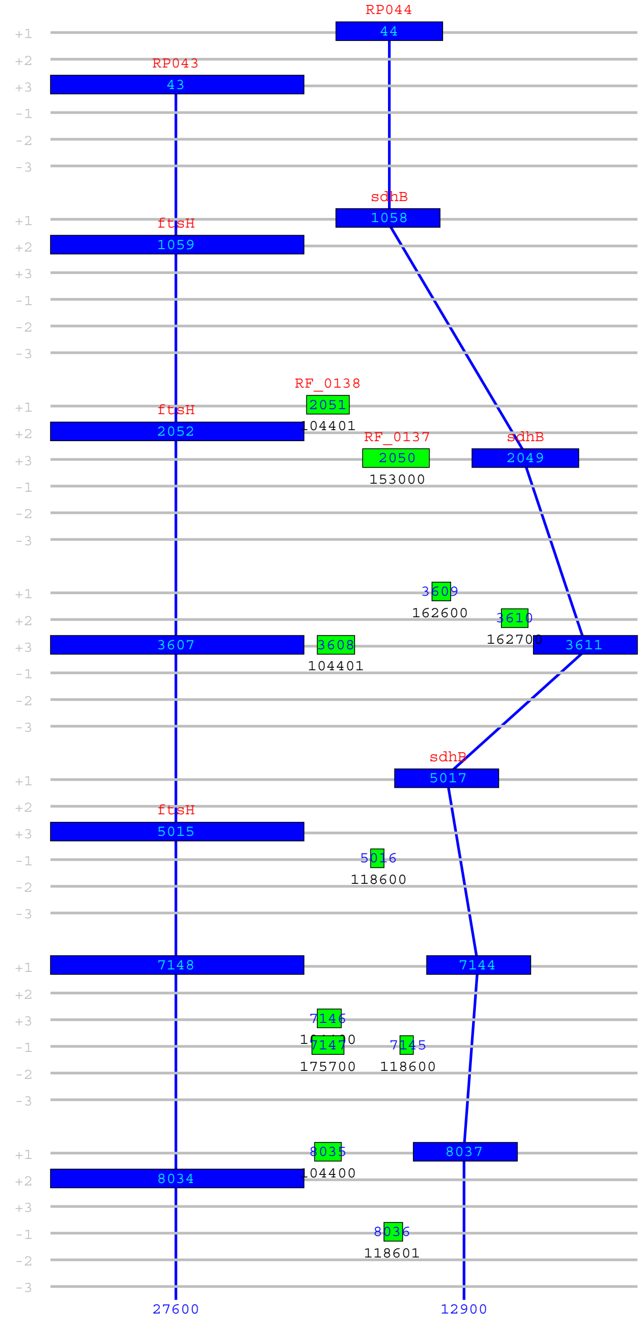

1 Rickettsia prowazekii str. Madrid E, complete genome
2 Rickettsia typhi str. wilmington, complete genome
3 Rickettsia felis URRWXCal2, complete genome
4 Rickettsia akari str. Hartford chromosome, whole genome shotgun sequence
5 Rickettsia conorii str. Malish 7, complete genome
6 Rickettsia sibirica 246 rsib_agncrt, whole genome shotgun sequence
7 Rickettsia rickettsii chromosome, whole genome shotgun sequence

Reg_id: 152

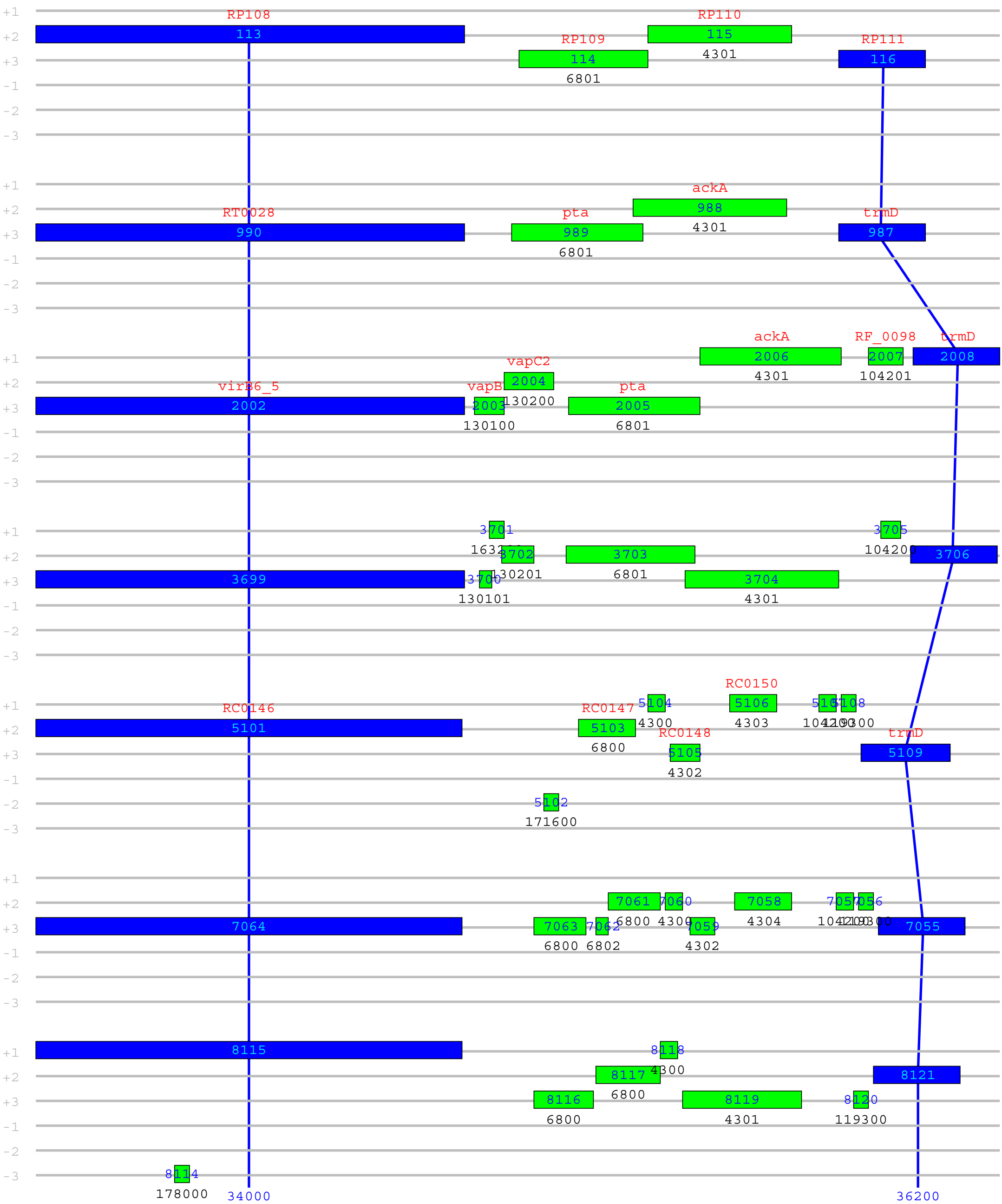

1 Rickettsia prowazekii str. Madrid E, complete genome
2 Rickettsia typhi str. wilmington, complete genome
3 Rickettsia felis URRWXCal2, complete genome
4 Rickettsia akari str. Hartford chromosome, whole genome shotgun sequence
5 Rickettsia conorii str. Malish 7, complete genome
6 Rickettsia sibirica 246 rsib_agncrt, whole genome shotgun sequence
7 Rickettsia rickettsii chromosome, whole genome shotgun sequence

Reg_id: 156

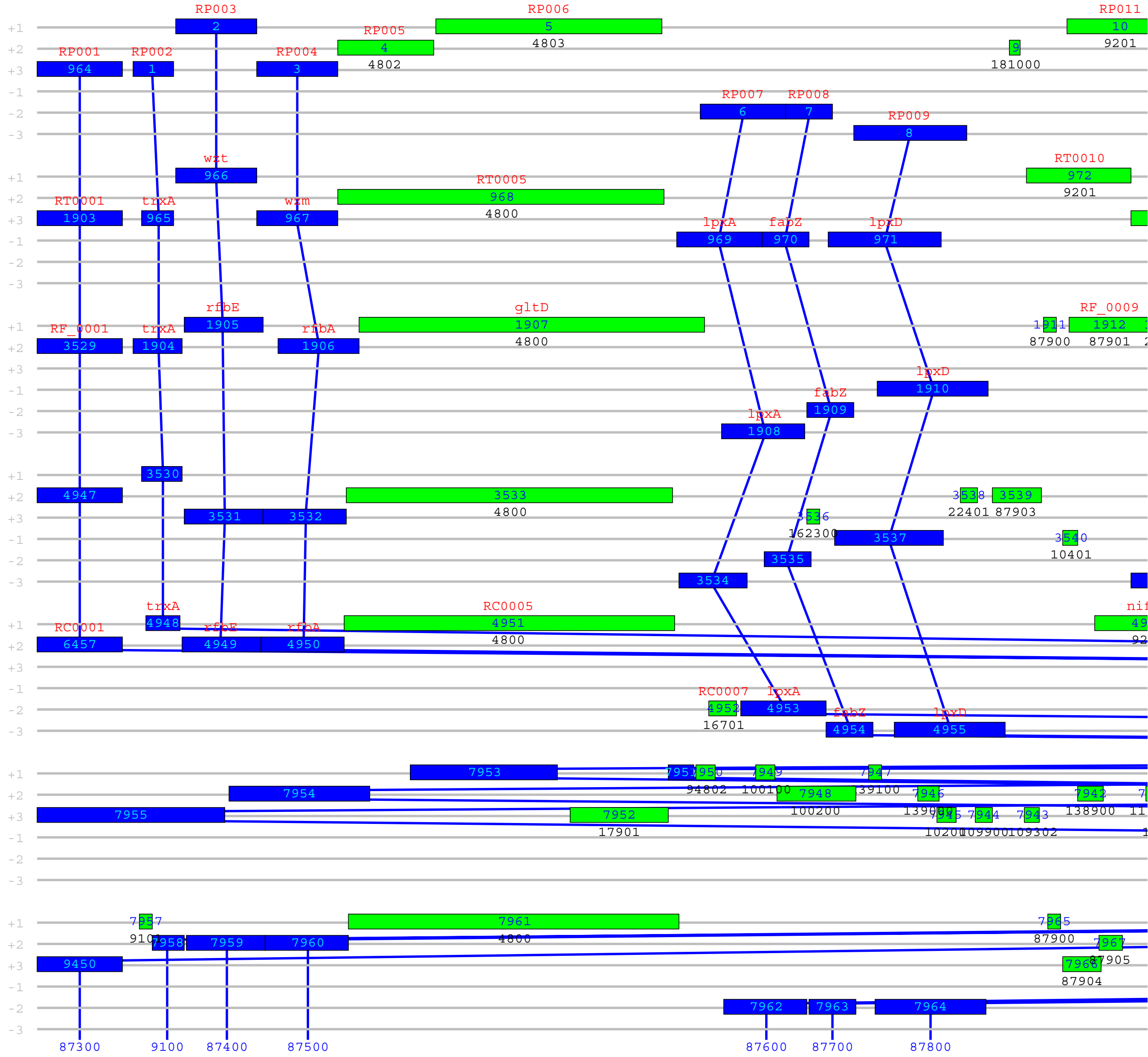

1 Rickettsia prowazekii str. Madrid E, complete genome
2 Rickettsia typhi str. wilmington, complete genome
3 Rickettsia felis URRWXCal2, complete genome
4 Rickettsia akari str. Hartford chromosome, whole genome shotgun sequence
5 Rickettsia conorii str. Malish 7, complete genome
6 Rickettsia sibirica 246 rsib_agncrt, whole genome shotgun sequence
7 Rickettsia rickettsii chromosome, whole genome shotgun sequence

Reg_id: 157

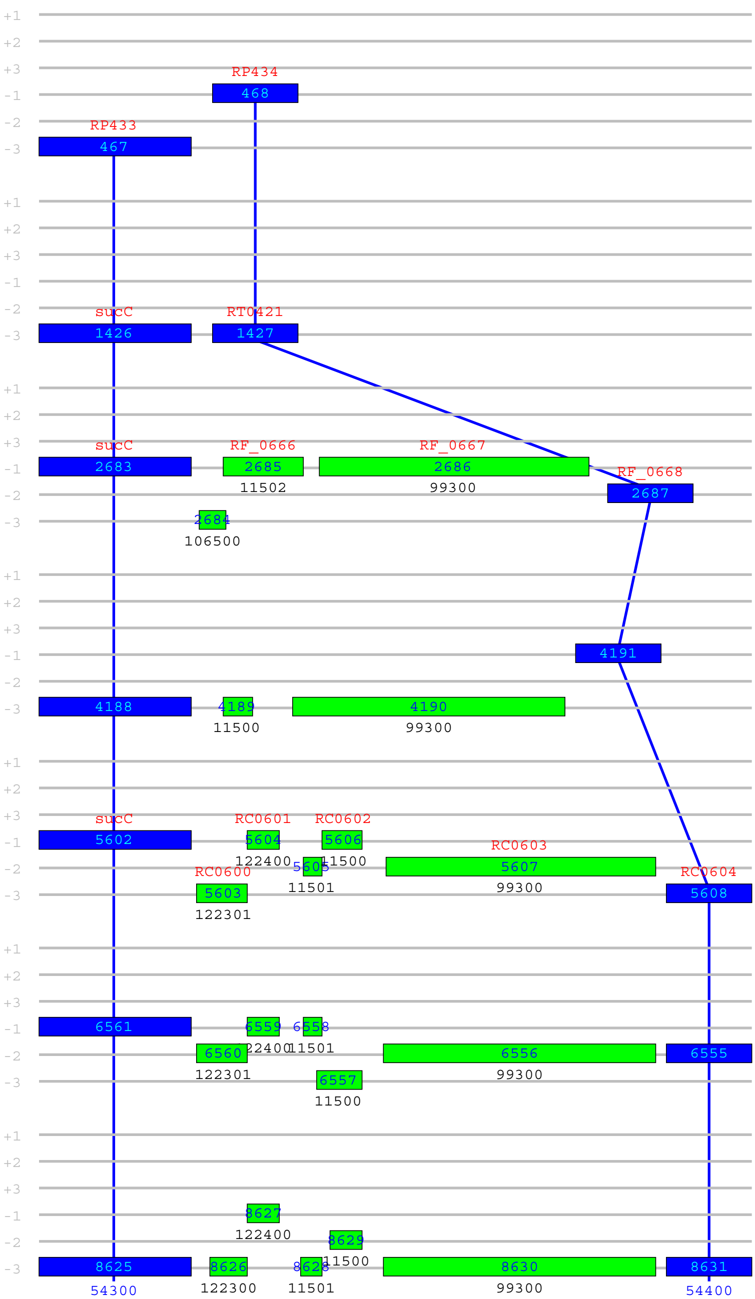

1 Rickettsia prowazekii str. Madrid E, complete genome
2 Rickettsia typhi str. wilmington, complete genome
3 Rickettsia felis URRWXCal2, complete genome
4 Rickettsia akari str. Hartford chromosome, whole genome shotgun sequence
5 Rickettsia conorii str. Malish 7, complete genome
6 Rickettsia sibirica 246 rsib_agncrt, whole genome shotgun sequence
7 Rickettsia rickettsii chromosome, whole genome shotgun sequence

Reg_id: 160

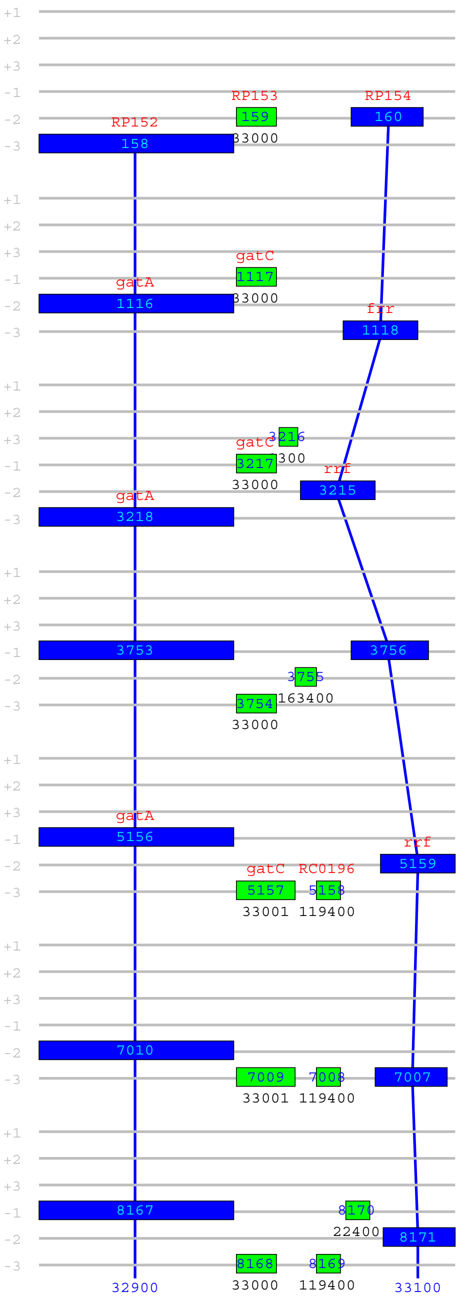

1 Rickettsia prowazekii str. Madrid E, complete genome
2 Rickettsia typhi str. wilmington, complete genome
3 Rickettsia felis URRWXCal2, complete genome
4 Rickettsia akari str. Hartford chromosome, whole genome shotgun sequence
5 Rickettsia conorii str. Malish 7, complete genome
6 Rickettsia sibirica 246 rsib_agncrt, whole genome shotgun sequence
7 Rickettsia rickettsii chromosome, whole genome shotgun sequence

Reg_id: 161

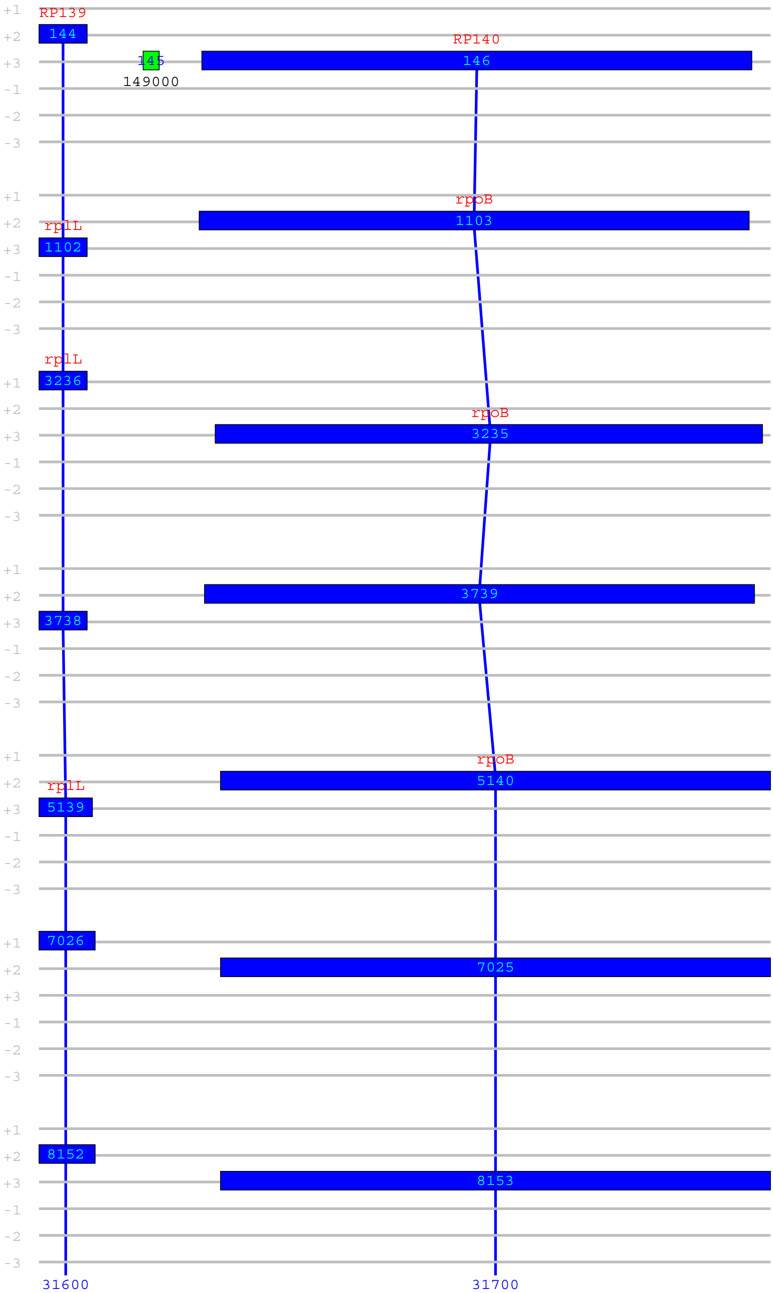

1 Rickettsia prowazekii str. Madrid E, complete genome
2 Rickettsia typhi str. wilmington, complete genome
3 Rickettsia felis URRWXCal2, complete genome
4 Rickettsia akari str. Hartford chromosome, whole genome shotgun sequence
5 Rickettsia conorii str. Malish 7, complete genome
6 Rickettsia sibirica 246 rsib_agncrt, whole genome shotgun sequence
7 Rickettsia rickettsii chromosome, whole genome shotgun sequence

Reg_id: 162

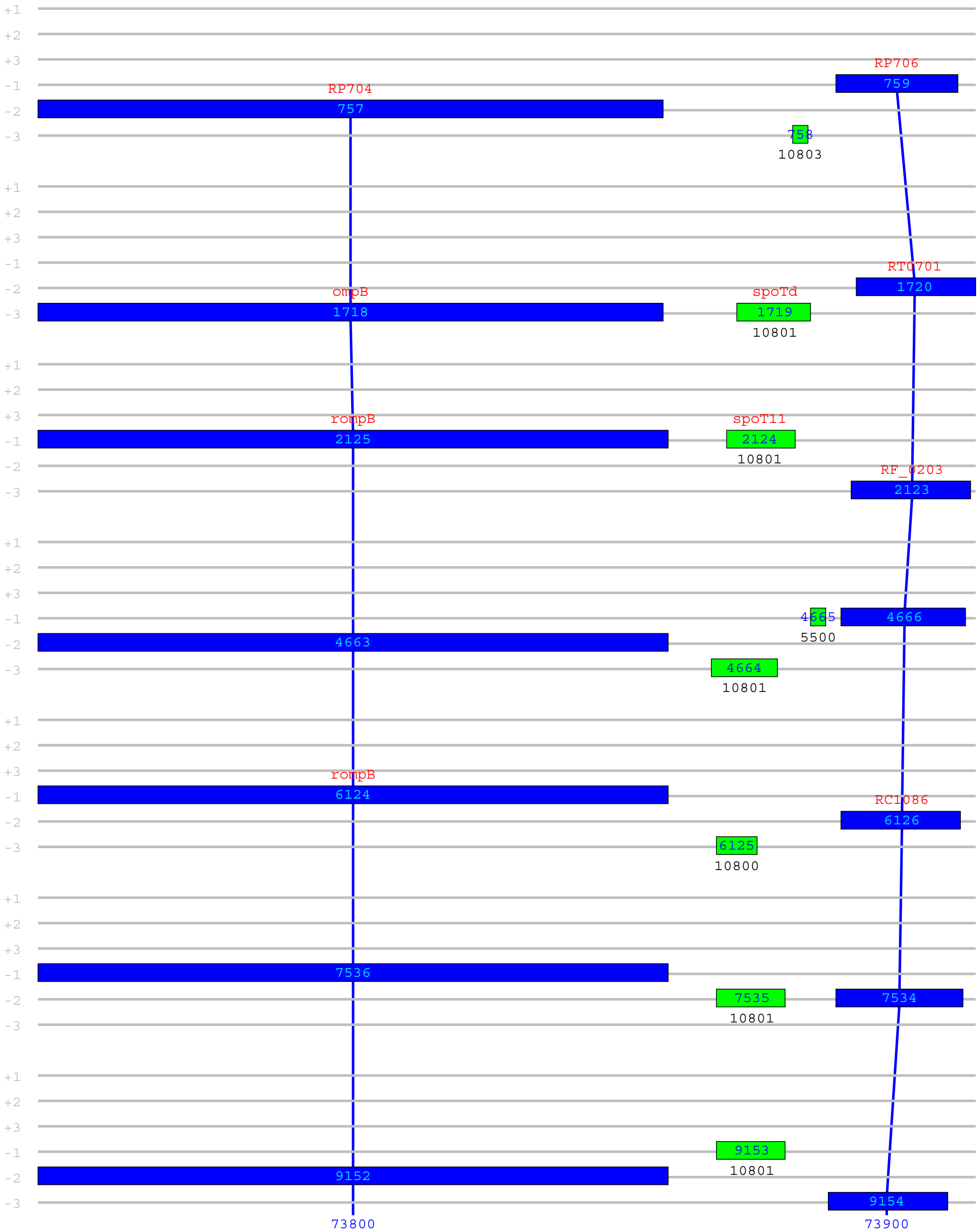

1 Rickettsia prowazekii str. Madrid E, complete genome
2 Rickettsia typhi str. wilmington, complete genome
3 Rickettsia felis URRWXCal2, complete genome
4 Rickettsia akari str. Hartford chromosome, whole genome shotgun sequence
5 Rickettsia conorii str. Malish 7, complete genome
6 Rickettsia sibirica 246 rsib_agncrt, whole genome shotgun sequence
7 Rickettsia rickettsii chromosome, whole genome shotgun sequence

Reg_id: 163

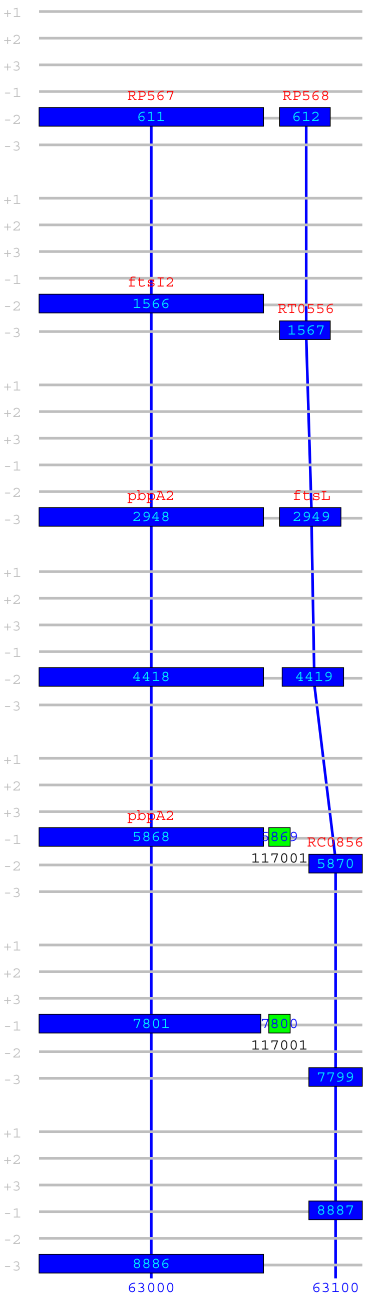

1 Rickettsia prowazekii str. Madrid E, complete genome
2 Rickettsia typhi str. wilmington, complete genome
3 Rickettsia felis URRWXCal2, complete genome
4 Rickettsia akari str. Hartford chromosome, whole genome shotgun sequence
5 Rickettsia conorii str. Malish 7, complete genome
6 Rickettsia sibirica 246 rsib_agncrt, whole genome shotgun sequence
7 Rickettsia rickettsii chromosome, whole genome shotgun sequence

Reg_id: 164

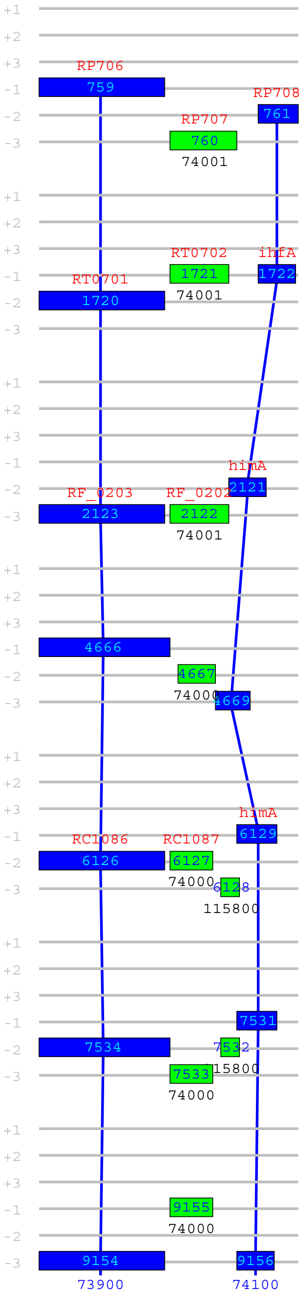

1 Rickettsia prowazekii str. Madrid E, complete genome
2 Rickettsia typhi str. wilmington, complete genome
3 Rickettsia felis URRWXCal2, complete genome
4 Rickettsia akari str. Hartford chromosome, whole genome shotgun sequence
5 Rickettsia conorii str. Malish 7, complete genome
6 Rickettsia sibirica 246 rsib_agncrt, whole genome shotgun sequence
7 Rickettsia rickettsii chromosome, whole genome shotgun sequence

Reg_id: 166

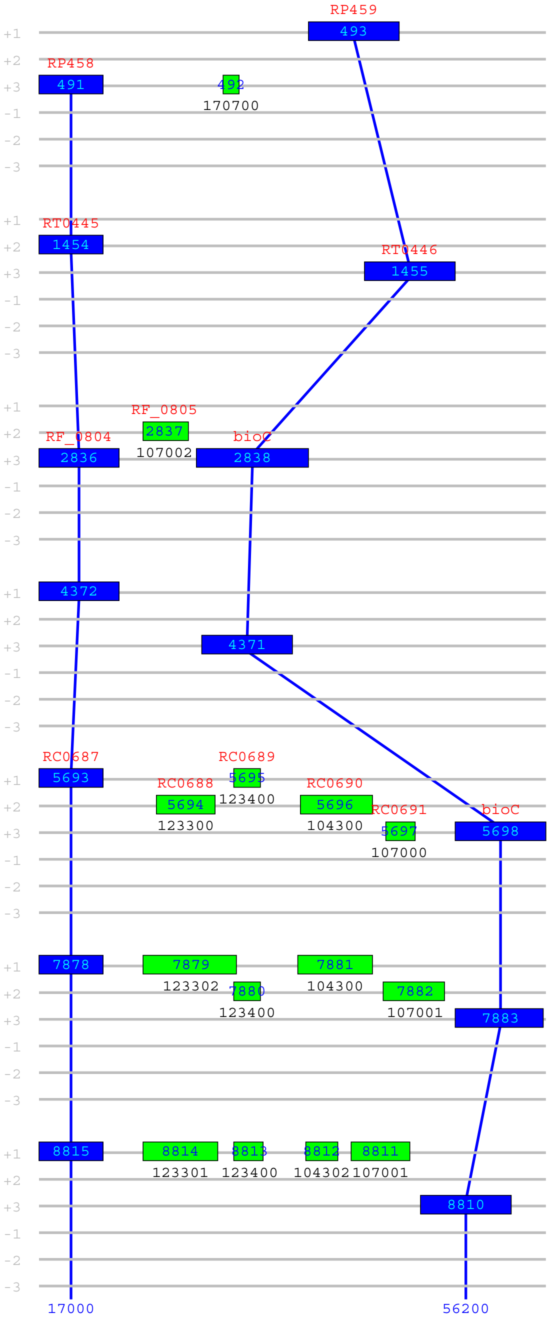

1 Rickettsia prowazekii str. Madrid E, complete genome
2 Rickettsia typhi str. wilmington, complete genome
3 Rickettsia felis URRWXCal2, complete genome
4 Rickettsia akari str. Hartford chromosome, whole genome shotgun sequence
5 Rickettsia conorii str. Malish 7, complete genome
6 Rickettsia sibirica 246 rsib_agncrt, whole genome shotgun sequence
7 Rickettsia rickettsii chromosome, whole genome shotgun sequence

Reg_id: 168

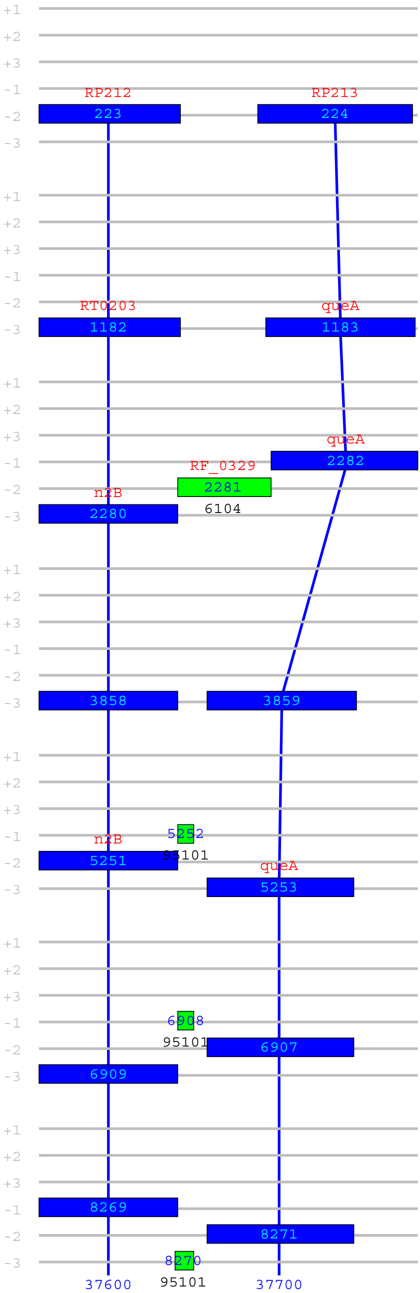

1 Rickettsia prowazekii str. Madrid E, complete genome
2 Rickettsia typhi str. wilmington, complete genome
3 Rickettsia felis URRWXCal2, complete genome
4 Rickettsia akari str. Hartford chromosome, whole genome shotgun sequence
5 Rickettsia conorii str. Malish 7, complete genome
6 Rickettsia sibirica 246 rsib_agncrt, whole genome shotgun sequence
7 Rickettsia rickettsii chromosome, whole genome shotgun sequence

Reg_id: 169

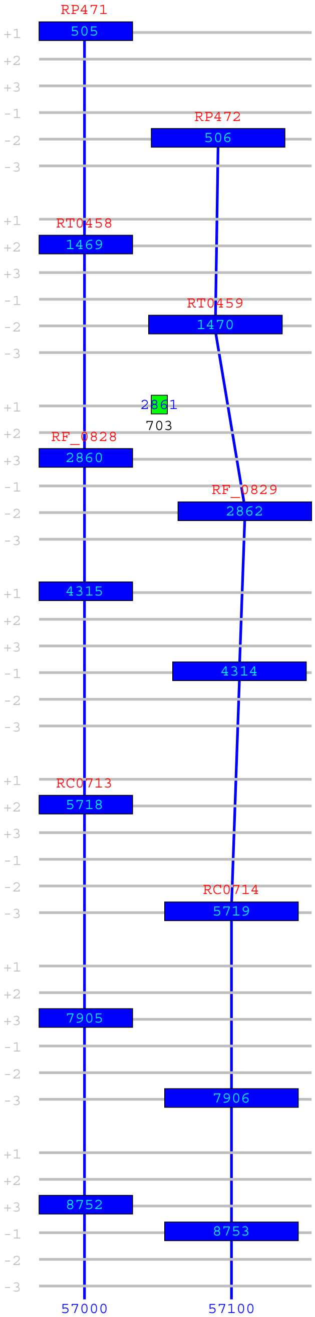

1 Rickettsia prowazekii str. Madrid E, complete genome
2 Rickettsia typhi str. wilmington, complete genome
3 Rickettsia felis URRWXCal2, complete genome
4 Rickettsia akari str. Hartford chromosome, whole genome shotgun sequence
5 Rickettsia conorii str. Malish 7, complete genome
6 Rickettsia sibirica 246 rsib_agncrt, whole genome shotgun sequence
7 Rickettsia rickettsii chromosome, whole genome shotgun sequence

Reg_id: 171

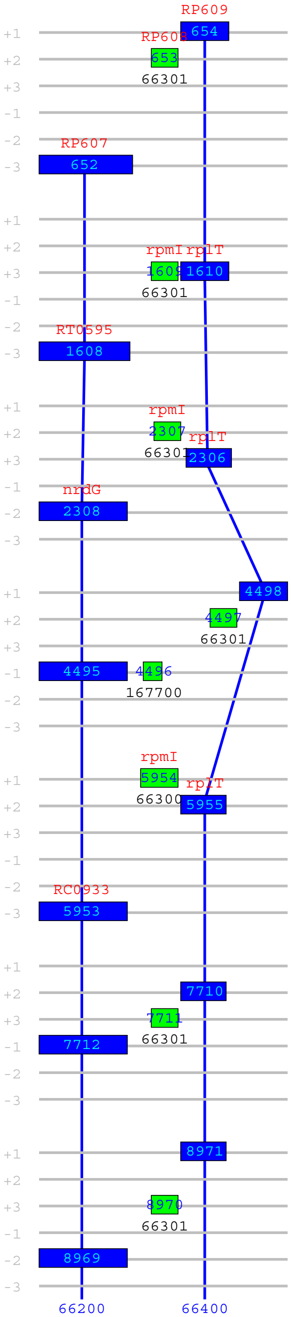

1 Rickettsia prowazekii str. Madrid E, complete genome
2 Rickettsia typhi str. wilmington, complete genome
3 Rickettsia felis URRWXCal2, complete genome
4 Rickettsia akari str. Hartford chromosome, whole genome shotgun sequence
5 Rickettsia conorii str. Malish 7, complete genome
6 Rickettsia sibirica 246 rsib_agncrt, whole genome shotgun sequence
7 Rickettsia rickettsii chromosome, whole genome shotgun sequence

Reg_id: 177

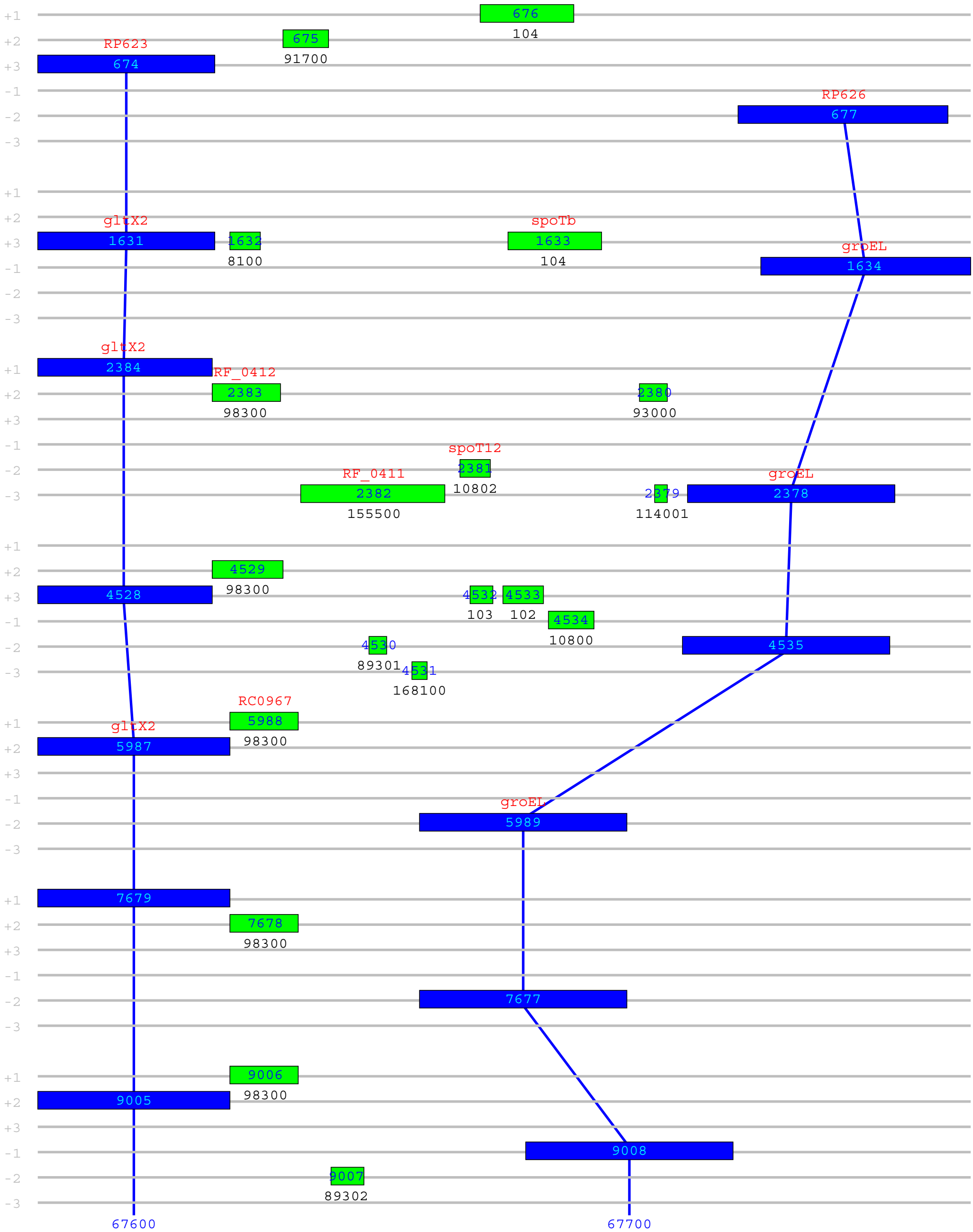

1 Rickettsia prowazekii str. Madrid E, complete genome
2 Rickettsia typhi str. wilmington, complete genome
3 Rickettsia felis URRWXCal2, complete genome
4 Rickettsia akari str. Hartford chromosome, whole genome shotgun sequence
5 Rickettsia conorii str. Malish 7, complete genome
6 Rickettsia sibirica 246 rsib_agncrt, whole genome shotgun sequence
7 Rickettsia rickettsii chromosome, whole genome shotgun sequence

Reg_id: 181

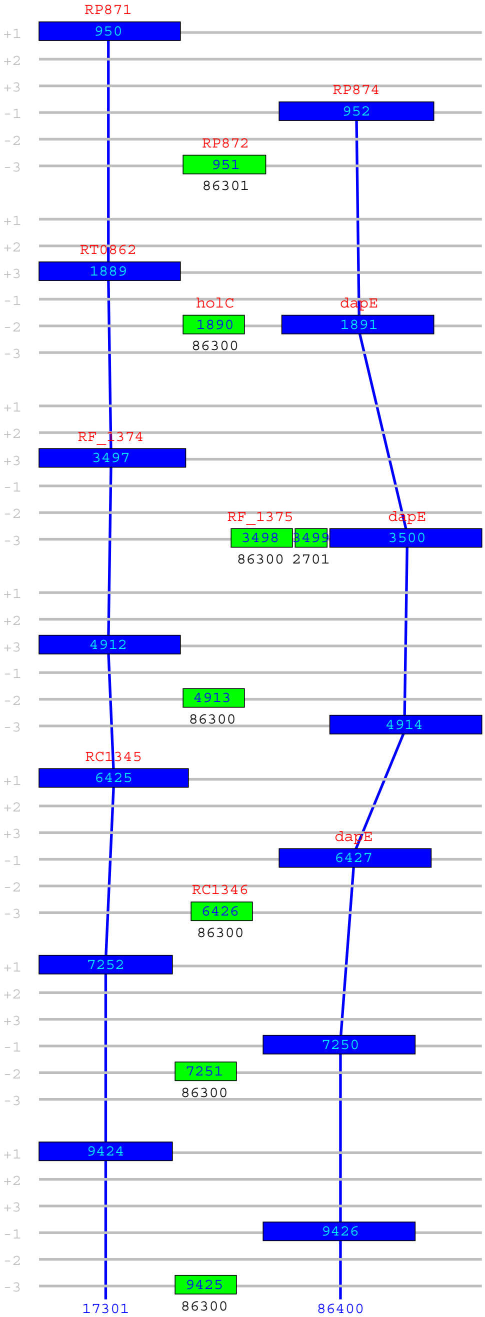

1 Rickettsia prowazekii str. Madrid E, complete genome
2 Rickettsia typhi str. wilmington, complete genome
3 Rickettsia felis URRWXCal2, complete genome
4 Rickettsia akari str. Hartford chromosome, whole genome shotgun sequence
5 Rickettsia conorii str. Malish 7, complete genome
6 Rickettsia sibirica 246 rsib_agncrt, whole genome shotgun sequence
7 Rickettsia rickettsii chromosome, whole genome shotgun sequence

Reg_id: 182

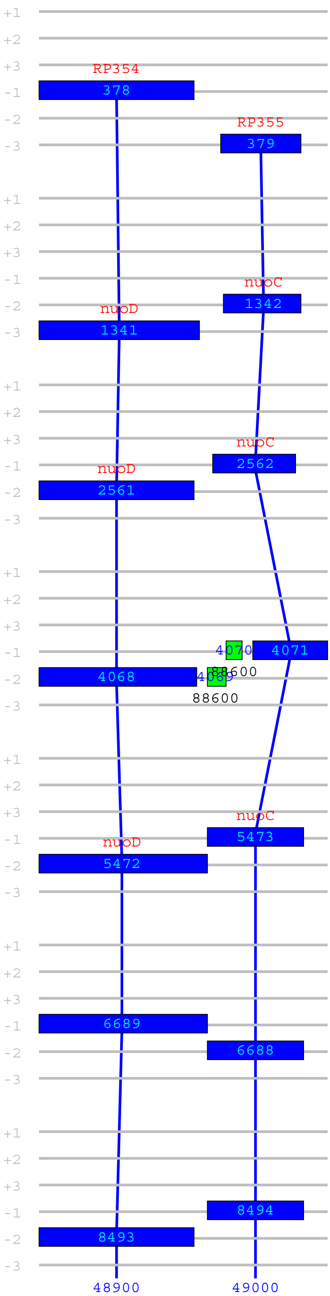

1 Rickettsia prowazekii str. Madrid E, complete genome
2 Rickettsia typhi str. wilmington, complete genome
3 Rickettsia felis URRWXCal2, complete genome
4 Rickettsia akari str. Hartford chromosome, whole genome shotgun sequence
5 Rickettsia conorii str. Malish 7, complete genome
6 Rickettsia sibirica 246 rsib_agncrt, whole genome shotgun sequence
7 Rickettsia rickettsii chromosome, whole genome shotgun sequence

Reg_id: 184

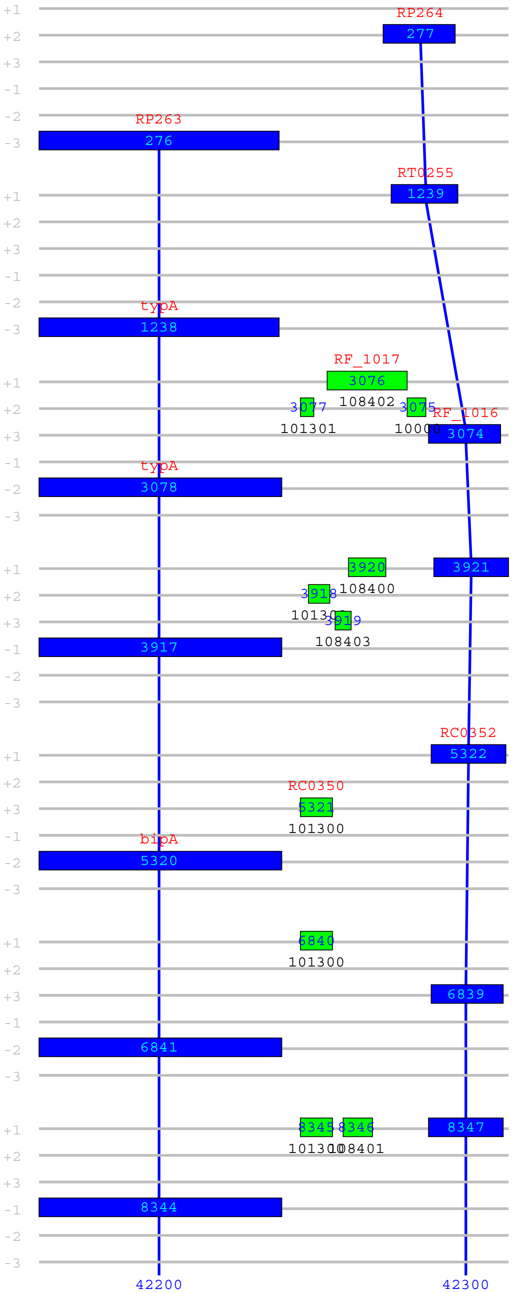

1 Rickettsia prowazekii str. Madrid E, complete genome
2 Rickettsia typhi str. wilmington, complete genome
3 Rickettsia felis URRWXCal2, complete genome
4 Rickettsia akari str. Hartford chromosome, whole genome shotgun sequence
5 Rickettsia conorii str. Malish 7, complete genome
6 Rickettsia sibirica 246 rsib_agncrt, whole genome shotgun sequence
7 Rickettsia rickettsii chromosome, whole genome shotgun sequence

Reg_id: 186

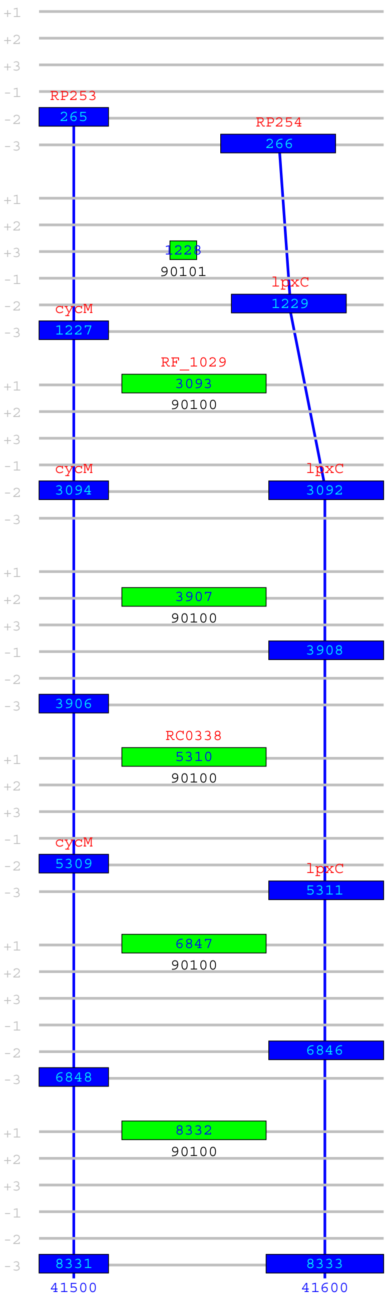

1 Rickettsia prowazekii str. Madrid E, complete genome
2 Rickettsia typhi str. wilmington, complete genome
3 Rickettsia felis URRWXCal2, complete genome
4 Rickettsia akari str. Hartford chromosome, whole genome shotgun sequence
5 Rickettsia conorii str. Malish 7, complete genome
6 Rickettsia sibirica 246 rsib_agncrt, whole genome shotgun sequence
7 Rickettsia rickettsii chromosome, whole genome shotgun sequence

Reg_id: 187

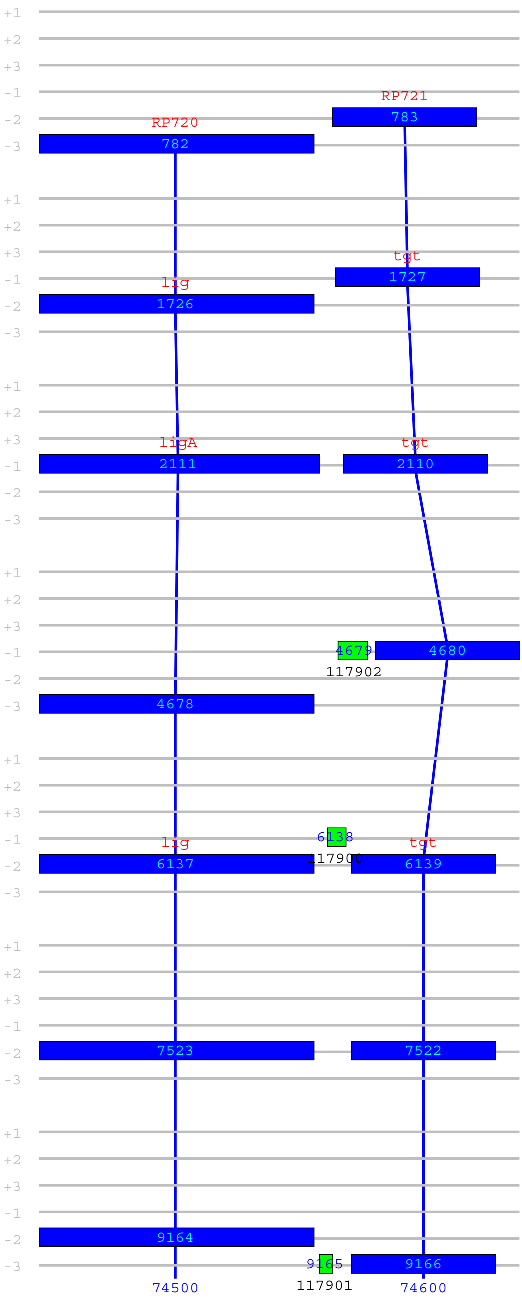

1 Rickettsia prowazekii str. Madrid E, complete genome
2 Rickettsia typhi str. wilmington, complete genome
3 Rickettsia felis URRWXCal2, complete genome
4 Rickettsia akari str. Hartford chromosome, whole genome shotgun sequence
5 Rickettsia conorii str. Malish 7, complete genome
6 Rickettsia sibirica 246 rsib_agncrt, whole genome shotgun sequence
7 Rickettsia rickettsii chromosome, whole genome shotgun sequence

Reg_id: 191

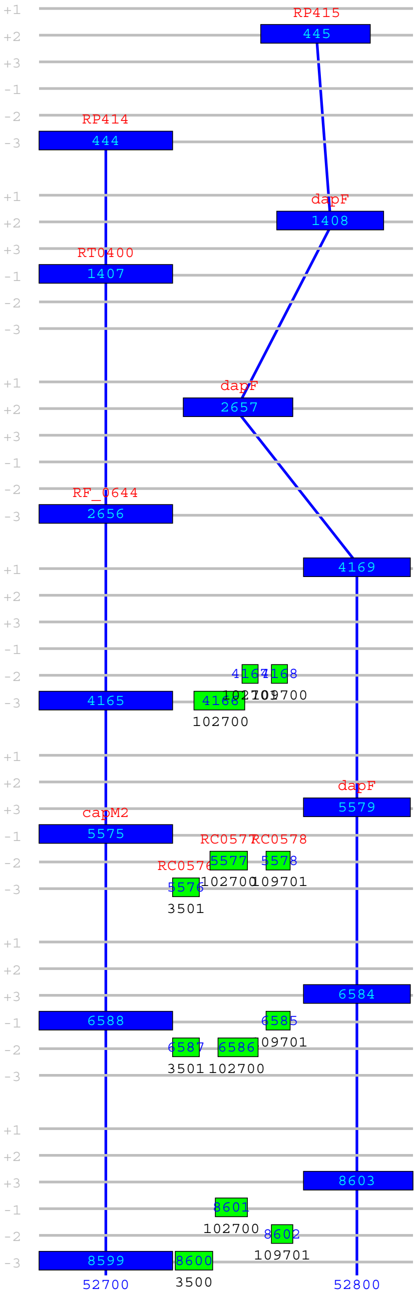

1 Rickettsia prowazekii str. Madrid E, complete genome
2 Rickettsia typhi str. wilmington, complete genome
3 Rickettsia felis URRWXCal2, complete genome
4 Rickettsia akari str. Hartford chromosome, whole genome shotgun sequence
5 Rickettsia conorii str. Malish 7, complete genome
6 Rickettsia sibirica 246 rsib_agncrt, whole genome shotgun sequence
7 Rickettsia rickettsii chromosome, whole genome shotgun sequence

Reg_id: 193

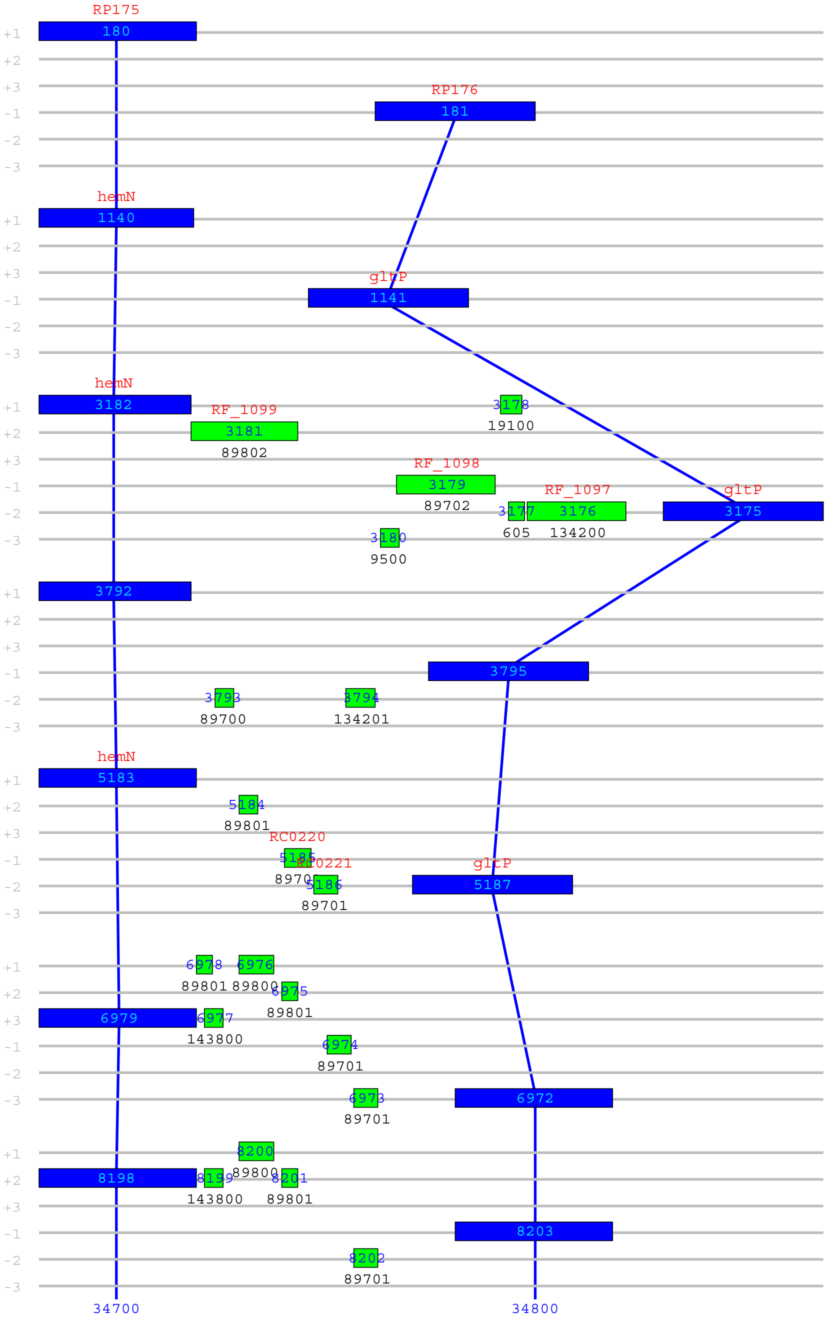

1 Rickettsia prowazekii str. Madrid E, complete genome
2 Rickettsia typhi str. wilmington, complete genome
3 Rickettsia felis URRWXCal2, complete genome
4 Rickettsia akari str. Hartford chromosome, whole genome shotgun sequence
5 Rickettsia conorii str. Malish 7, complete genome
6 Rickettsia sibirica 246 rsib_agncrt, whole genome shotgun sequence
7 Rickettsia rickettsii chromosome, whole genome shotgun sequence

Reg_id: 196

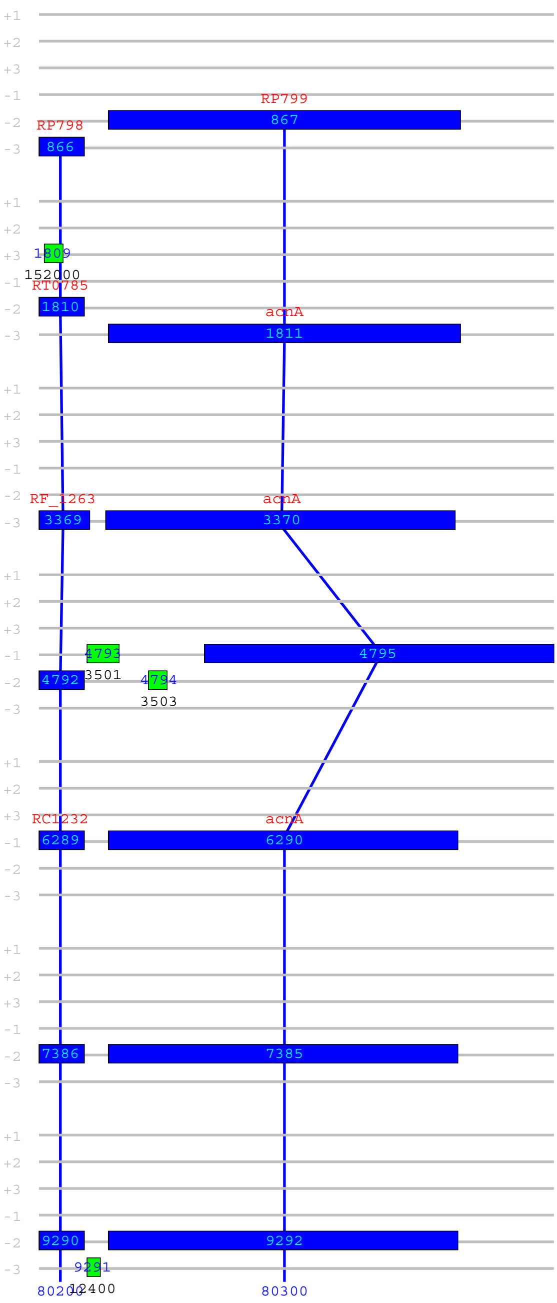

1 Rickettsia prowazekii str. Madrid E, complete genome
2 Rickettsia typhi str. wilmington, complete genome
3 Rickettsia felis URRWXCal2, complete genome
4 Rickettsia akari str. Hartford chromosome, whole genome shotgun sequence
5 Rickettsia conorii str. Malish 7, complete genome
6 Rickettsia sibirica 246 rsib_agncrt, whole genome shotgun sequence
7 Rickettsia rickettsii chromosome, whole genome shotgun sequence

Reg_id: 197

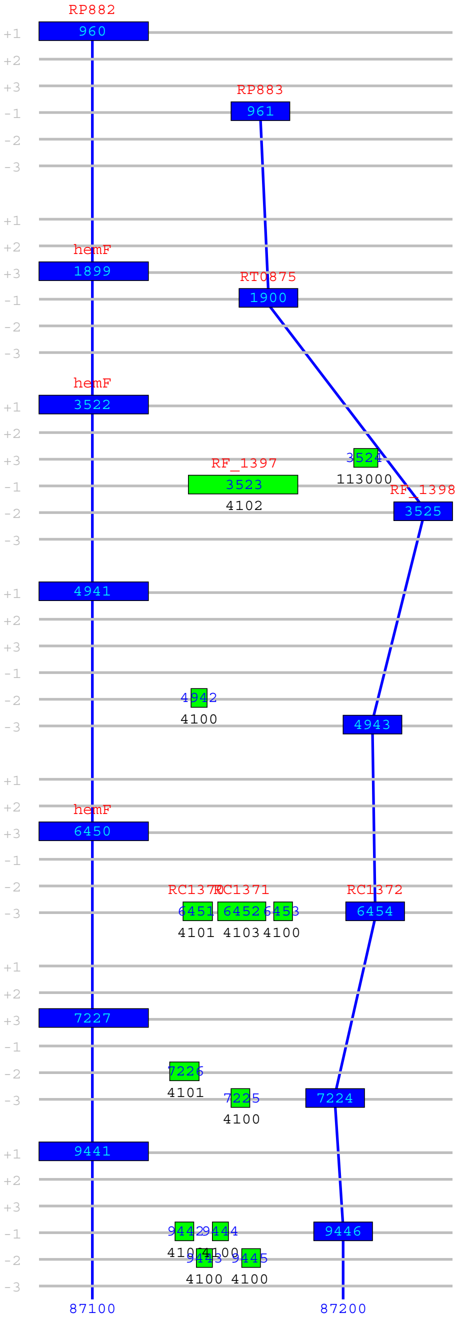

1 Rickettsia prowazekii str. Madrid E, complete genome
2 Rickettsia typhi str. wilmington, complete genome
3 Rickettsia felis URRWXCal2, complete genome
4 Rickettsia akari str. Hartford chromosome, whole genome shotgun sequence
5 Rickettsia conorii str. Malish 7, complete genome
6 Rickettsia sibirica 246 rsib_agncrt, whole genome shotgun sequence
7 Rickettsia rickettsii chromosome, whole genome shotgun sequence

Reg_id: 199

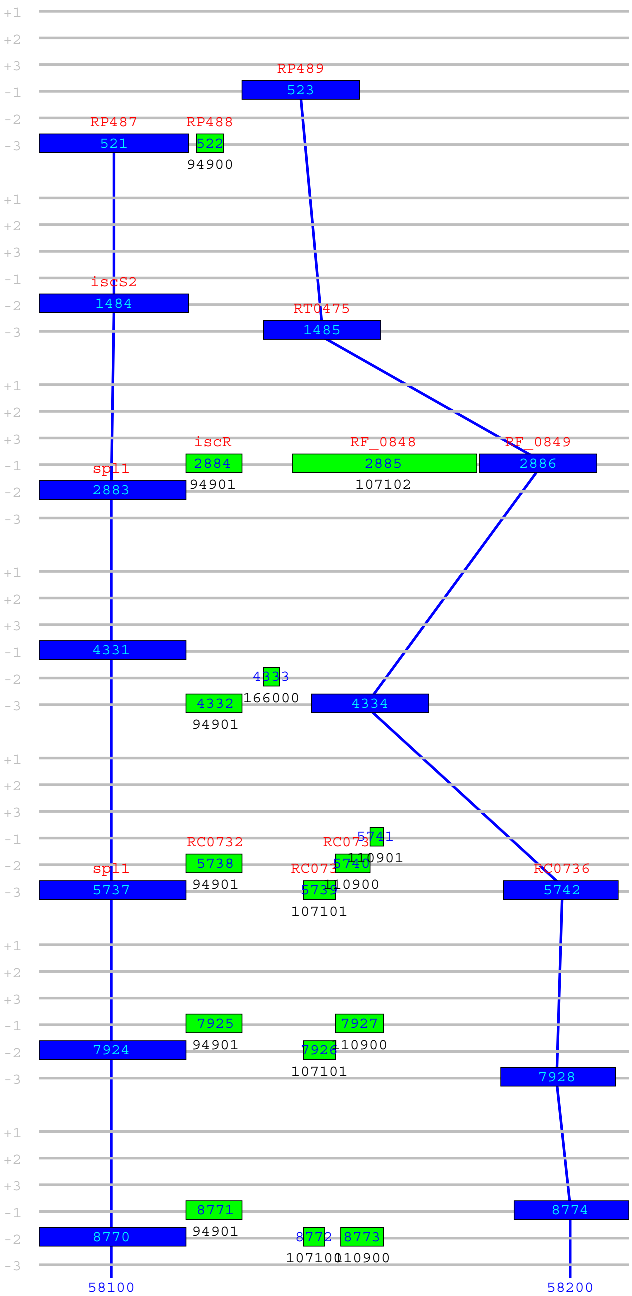

1 Rickettsia prowazekii str. Madrid E, complete genome
2 Rickettsia typhi str. wilmington, complete genome
3 Rickettsia felis URRWXCal2, complete genome
4 Rickettsia akari str. Hartford chromosome, whole genome shotgun sequence
5 Rickettsia conorii str. Malish 7, complete genome
6 Rickettsia sibirica 246 rsib_agncrt, whole genome shotgun sequence
7 Rickettsia rickettsii chromosome, whole genome shotgun sequence

Reg_id: 200

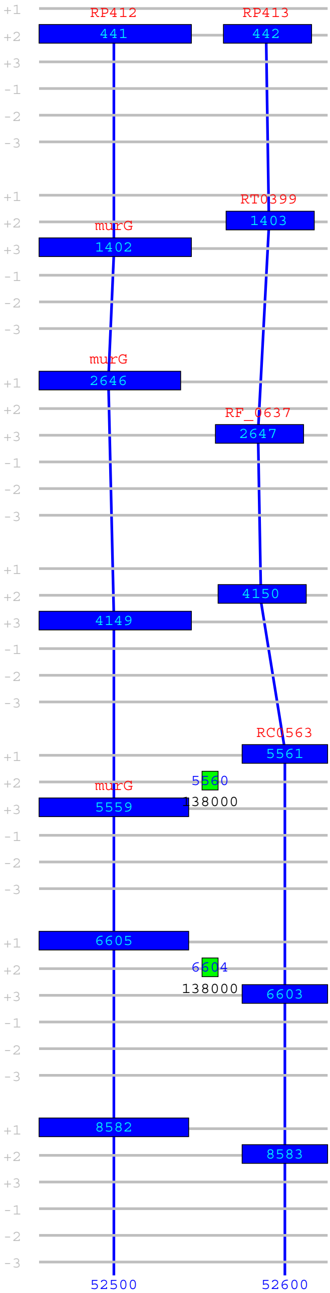

1 Rickettsia prowazekii str. Madrid E, complete genome
2 Rickettsia typhi str. wilmington, complete genome
3 Rickettsia felis URRWXCal2, complete genome
4 Rickettsia akari str. Hartford chromosome, whole genome shotgun sequence
5 Rickettsia conorii str. Malish 7, complete genome
6 Rickettsia sibirica 246 rsib_agncrt, whole genome shotgun sequence
7 Rickettsia rickettsii chromosome, whole genome shotgun sequence

Reg_id: 204

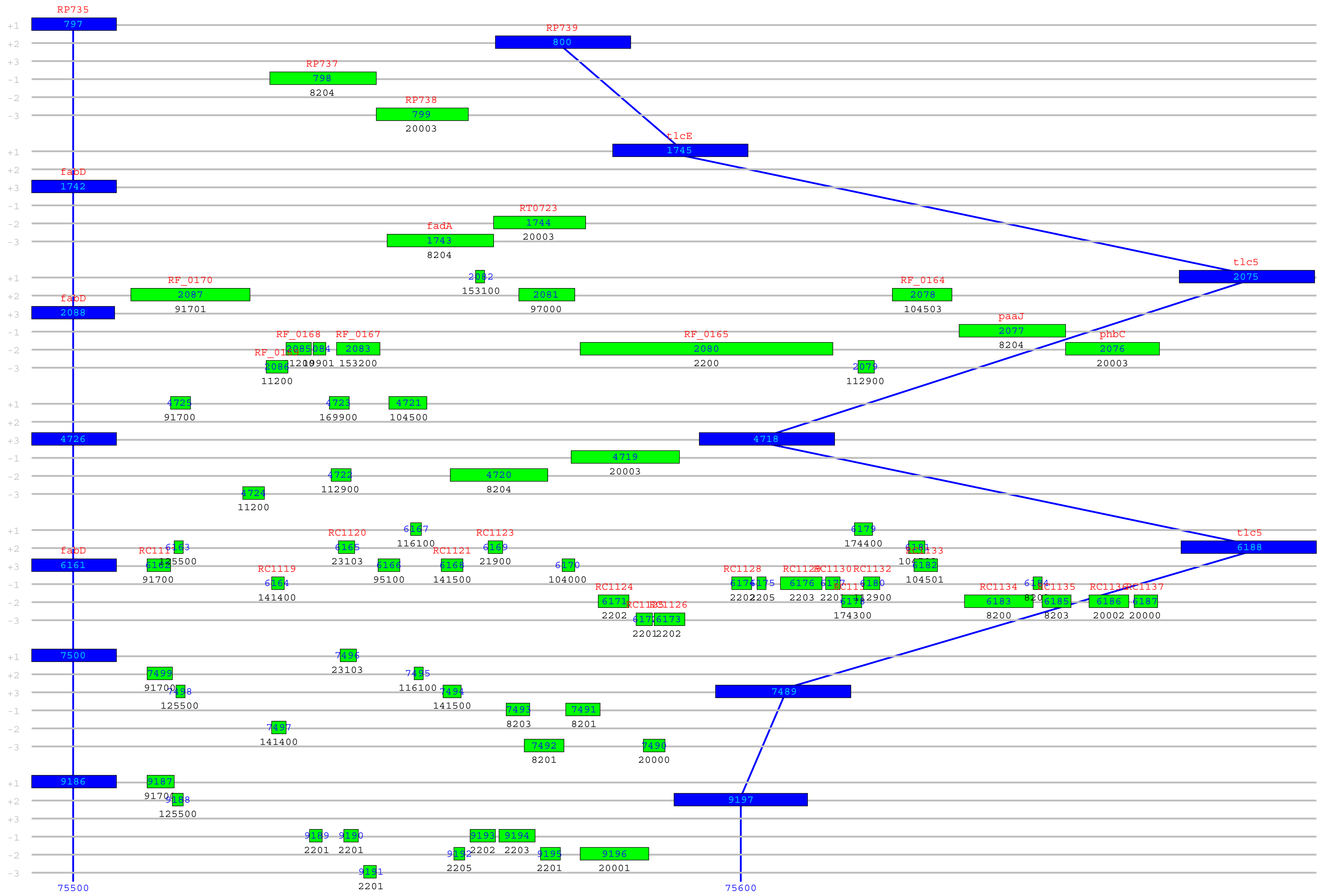

1 Rickettsia prowazekii str. Madrid E, complete genome
2 Rickettsia typhi str. wilmington, complete genome
3 Rickettsia felis URRWXCal2, complete genome
4 Rickettsia akari str. Hartford chromosome, whole genome shotgun sequence
5 Rickettsia conorii str. Malish 7, complete genome
6 Rickettsia sibirica 246 rsib_agncrt, whole genome shotgun sequence
7 Rickettsia rickettsii chromosome, whole genome shotgun sequence

Reg_id: 205

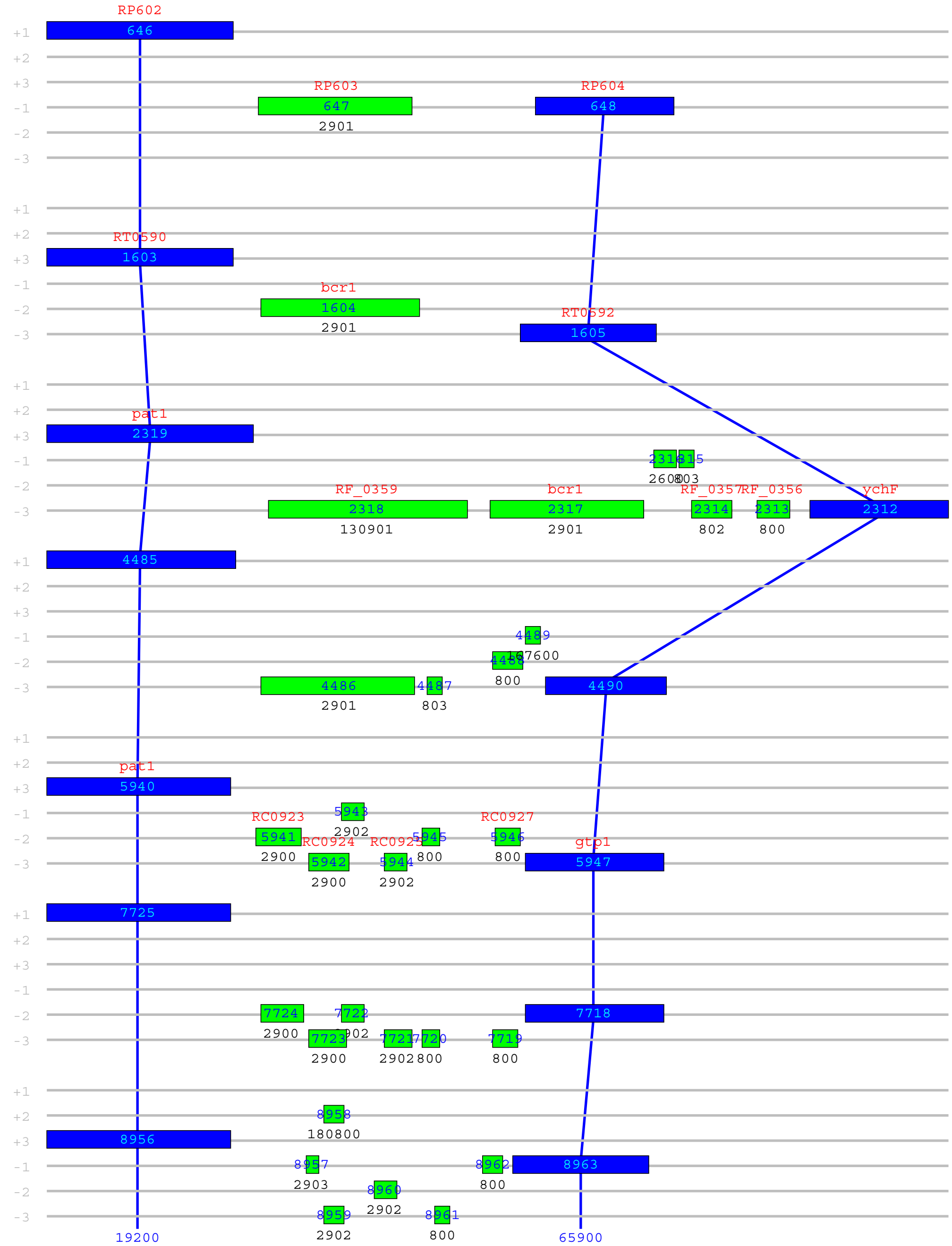

1 Rickettsia prowazekii str. Madrid E, complete genome
2 Rickettsia typhi str. wilmington, complete genome
3 Rickettsia felis URRWXCal2, complete genome
4 Rickettsia akari str. Hartford chromosome, whole genome shotgun sequence
5 Rickettsia conorii str. Malish 7, complete genome
6 Rickettsia sibirica 246 rsib_agncrt, whole genome shotgun sequence
7 Rickettsia rickettsii chromosome, whole genome shotgun sequence

Reg_id: 208

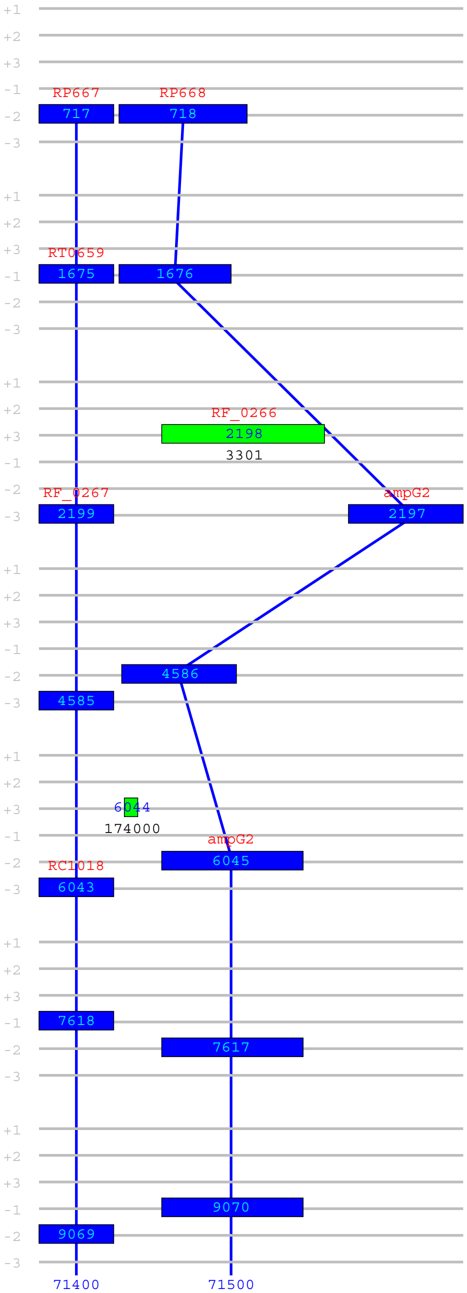

1 Rickettsia prowazekii str. Madrid E, complete genome
2 Rickettsia typhi str. wilmington, complete genome
3 Rickettsia felis URRWXCal2, complete genome
4 Rickettsia akari str. Hartford chromosome, whole genome shotgun sequence
5 Rickettsia conorii str. Malish 7, complete genome
6 Rickettsia sibirica 246 rsib_agncrt, whole genome shotgun sequence
7 Rickettsia rickettsii chromosome, whole genome shotgun sequence

Reg_id: 209

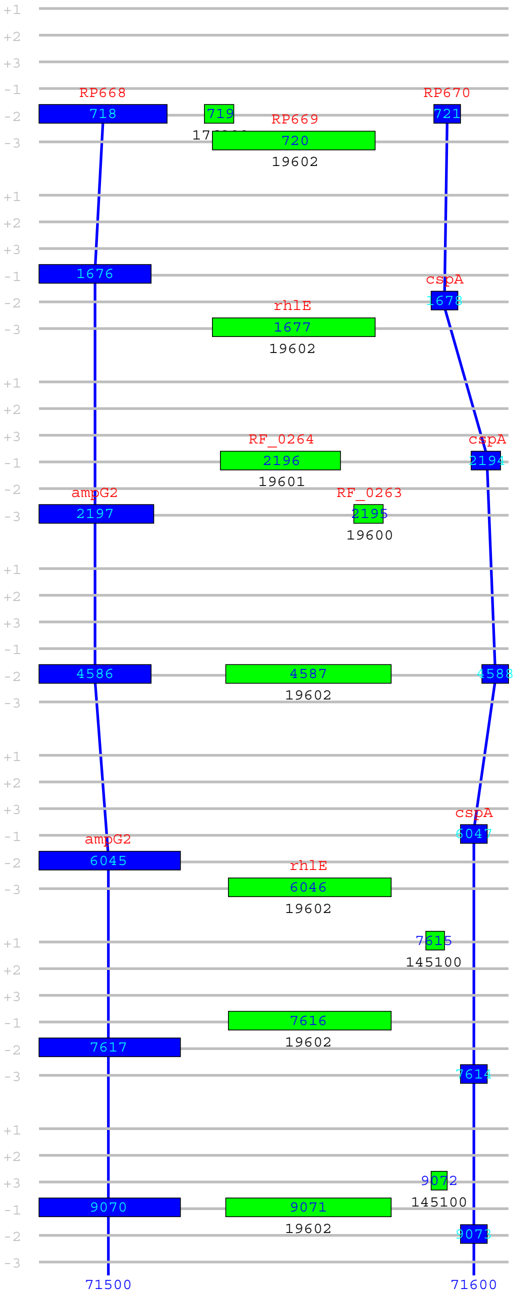

1 Rickettsia prowazekii str. Madrid E, complete genome
2 Rickettsia typhi str. wilmington, complete genome
3 Rickettsia felis URRWXCal2, complete genome
4 Rickettsia akari str. Hartford chromosome, whole genome shotgun sequence
5 Rickettsia conorii str. Malish 7, complete genome
6 Rickettsia sibirica 246 rsib_agncrt, whole genome shotgun sequence
7 Rickettsia rickettsii chromosome, whole genome shotgun sequence

Reg_id: 212

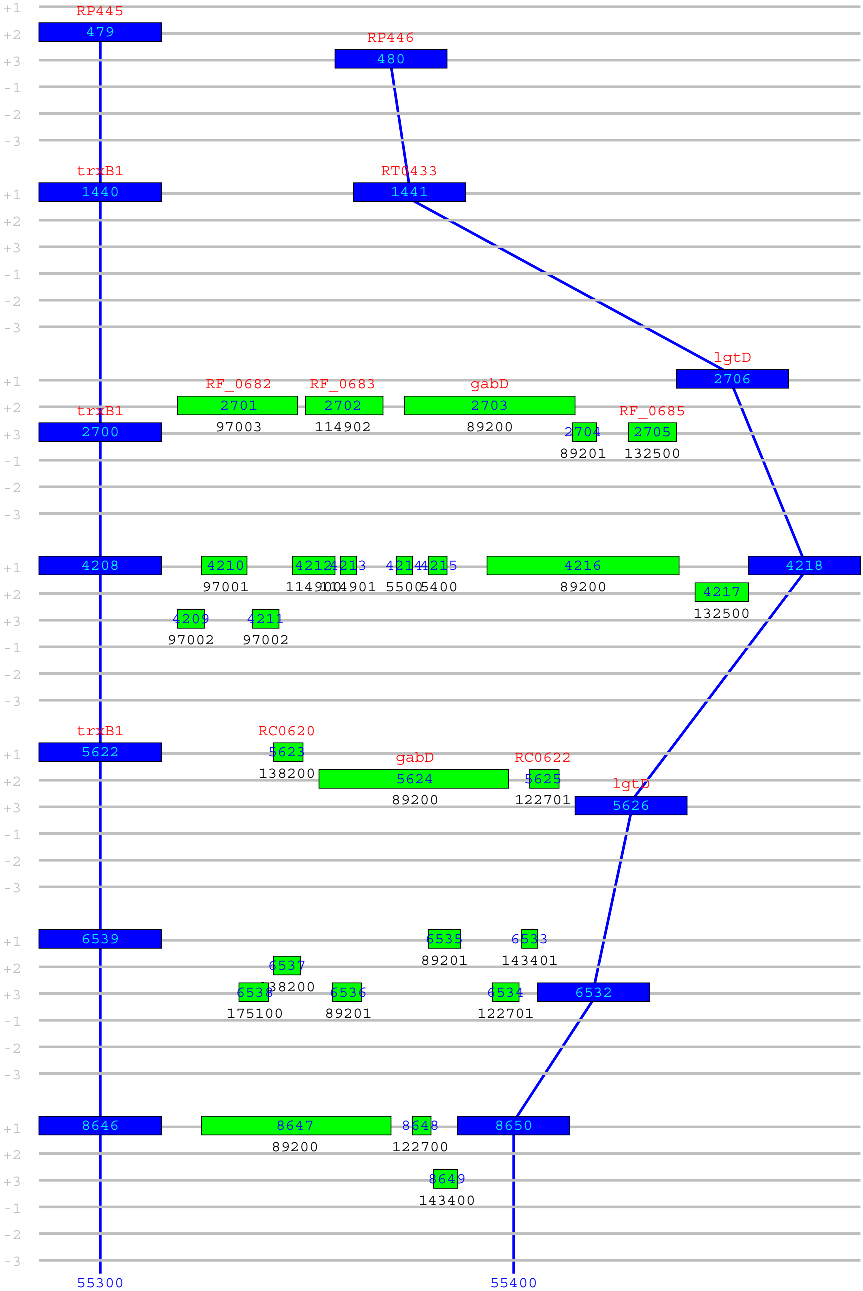

1 Rickettsia prowazekii str. Madrid E, complete genome
2 Rickettsia typhi str. wilmington, complete genome
3 Rickettsia felis URRWXCal2, complete genome
4 Rickettsia akari str. Hartford chromosome, whole genome shotgun sequence
5 Rickettsia conorii str. Malish 7, complete genome
6 Rickettsia sibirica 246 rsib_agncrt, whole genome shotgun sequence
7 Rickettsia rickettsii chromosome, whole genome shotgun sequence

Reg_id: 215

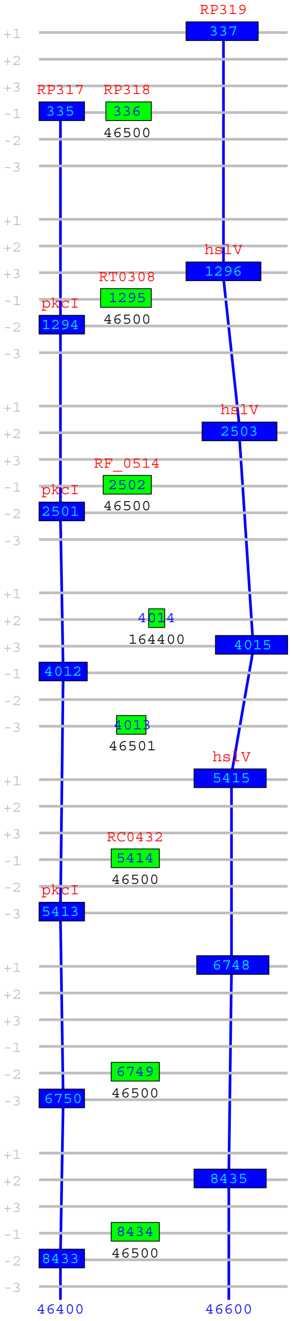

1 Rickettsia prowazekii str. Madrid E, complete genome
2 Rickettsia typhi str. wilmington, complete genome
3 Rickettsia felis URRWXCal2, complete genome
4 Rickettsia akari str. Hartford chromosome, whole genome shotgun sequence
5 Rickettsia conorii str. Malish 7, complete genome
6 Rickettsia sibirica 246 rsib_agncrt, whole genome shotgun sequence
7 Rickettsia rickettsii chromosome, whole genome shotgun sequence

Reg_id: 217

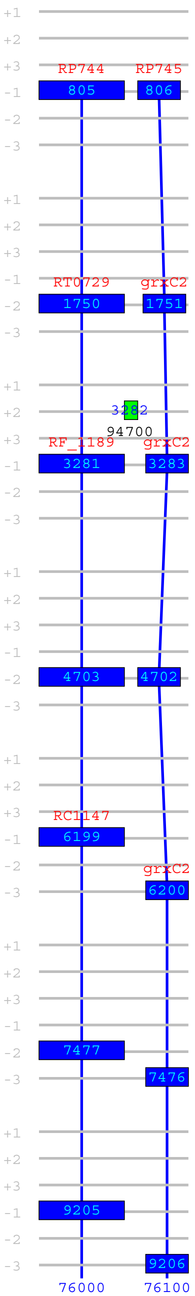

1 Rickettsia prowazekii str. Madrid E, complete genome
2 Rickettsia typhi str. wilmington, complete genome
3 Rickettsia felis URRWXCal2, complete genome
4 Rickettsia akari str. Hartford chromosome, whole genome shotgun sequence
5 Rickettsia conorii str. Malish 7, complete genome
6 Rickettsia sibirica 246 rsib_agncrt, whole genome shotgun sequence
7 Rickettsia rickettsii chromosome, whole genome shotgun sequence

Reg_id: 222

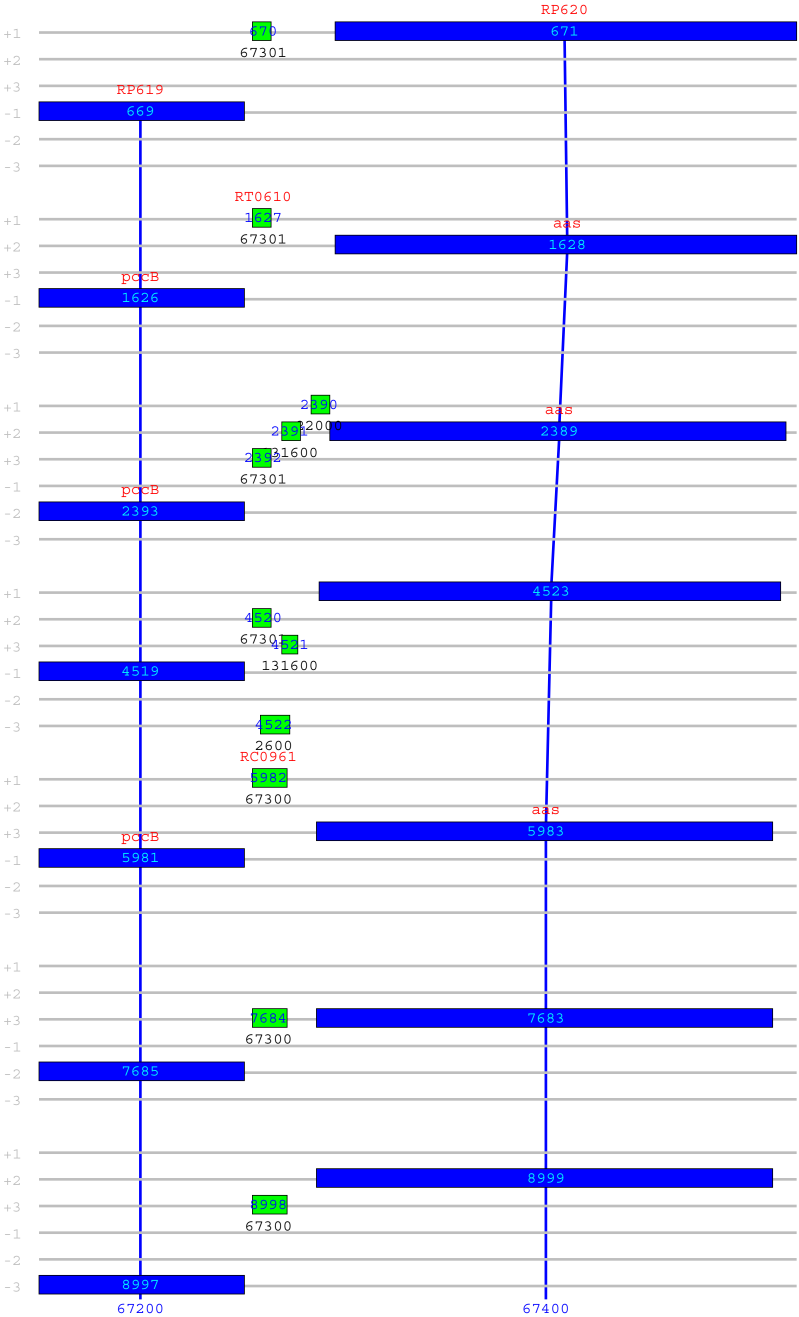

1 Rickettsia prowazekii str. Madrid E, complete genome
2 Rickettsia typhi str. wilmington, complete genome
3 Rickettsia felis URRWXCal2, complete genome
4 Rickettsia akari str. Hartford chromosome, whole genome shotgun sequence
5 Rickettsia conorii str. Malish 7, complete genome
6 Rickettsia sibirica 246 rsib_agncrt, whole genome shotgun sequence
7 Rickettsia rickettsii chromosome, whole genome shotgun sequence

Reg_id: 224

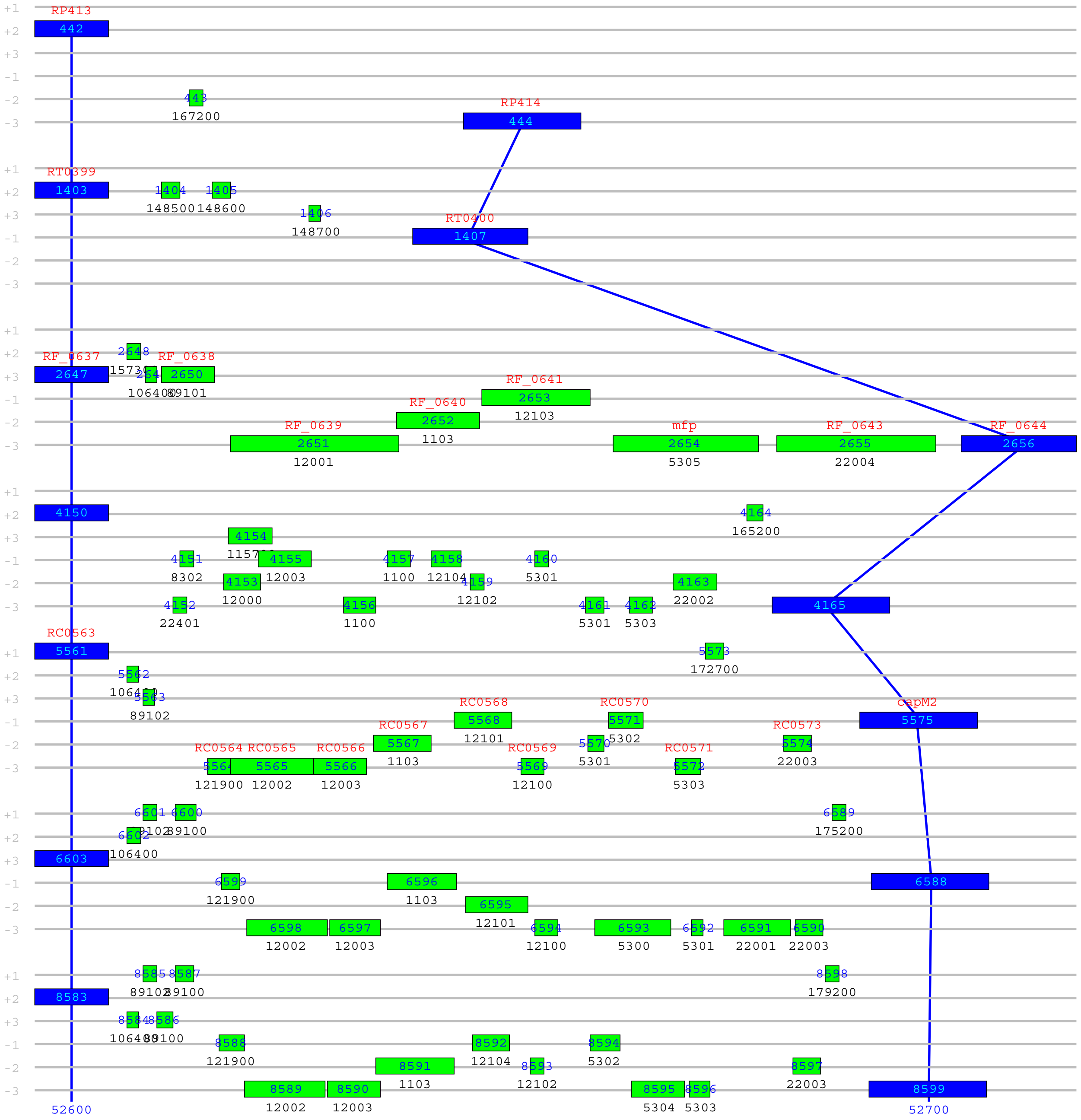

1 Rickettsia prowazekii str. Madrid E, complete genome
2 Rickettsia typhi str. wilmington, complete genome
3 Rickettsia felis URRWXCal2, complete genome
4 Rickettsia akari str. Hartford chromosome, whole genome shotgun sequence
5 Rickettsia conorii str. Malish 7, complete genome
6 Rickettsia sibirica 246 rsib_agncrt, whole genome shotgun sequence
7 Rickettsia rickettsii chromosome, whole genome shotgun sequence

Reg_id: 229

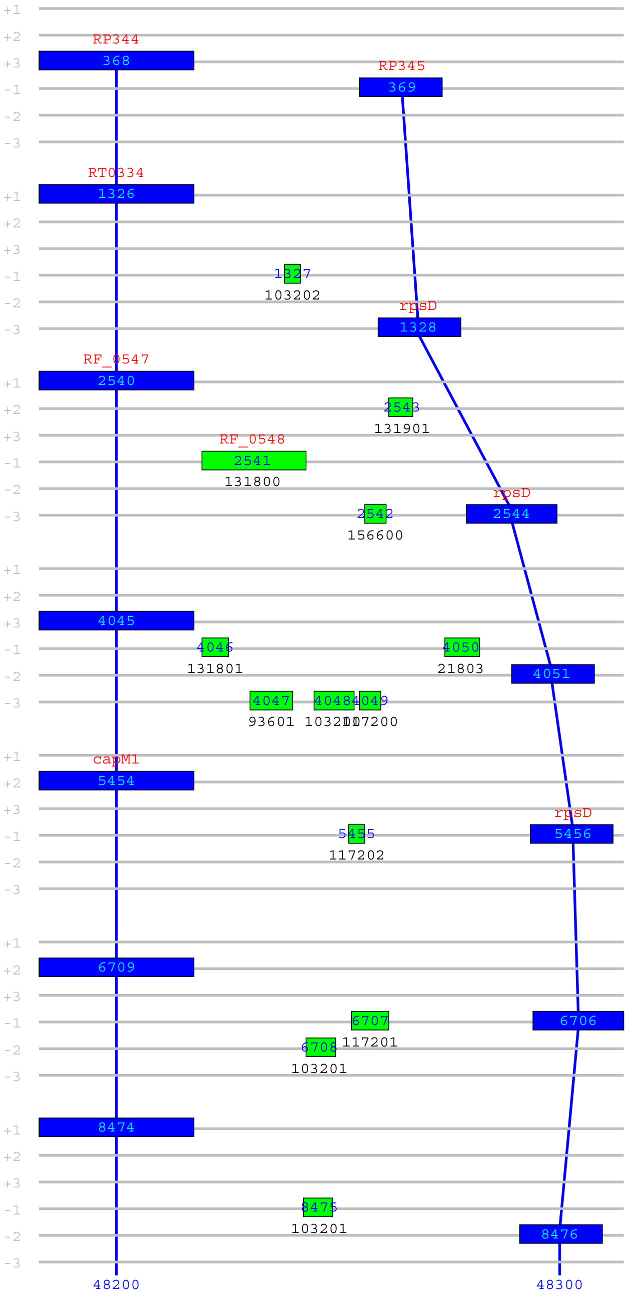

1 Rickettsia prowazekii str. Madrid E, complete genome
2 Rickettsia typhi str. wilmington, complete genome
3 Rickettsia felis URRWXCal2, complete genome
4 Rickettsia akari str. Hartford chromosome, whole genome shotgun sequence
5 Rickettsia conorii str. Malish 7, complete genome
6 Rickettsia sibirica 246 rsib_agncrt, whole genome shotgun sequence
7 Rickettsia rickettsii chromosome, whole genome shotgun sequence

Reg_id: 230

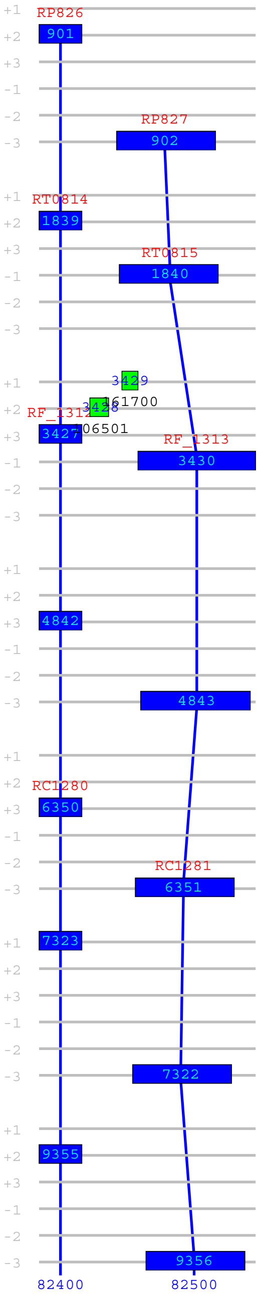

1 Rickettsia prowazekii str. Madrid E, complete genome
2 Rickettsia typhi str. wilmington, complete genome
3 Rickettsia felis URRWXCal2, complete genome
4 Rickettsia akari str. Hartford chromosome, whole genome shotgun sequence
5 Rickettsia conorii str. Malish 7, complete genome
6 Rickettsia sibirica 246 rsib_agncrt, whole genome shotgun sequence
7 Rickettsia rickettsii chromosome, whole genome shotgun sequence

Reg_id: 231

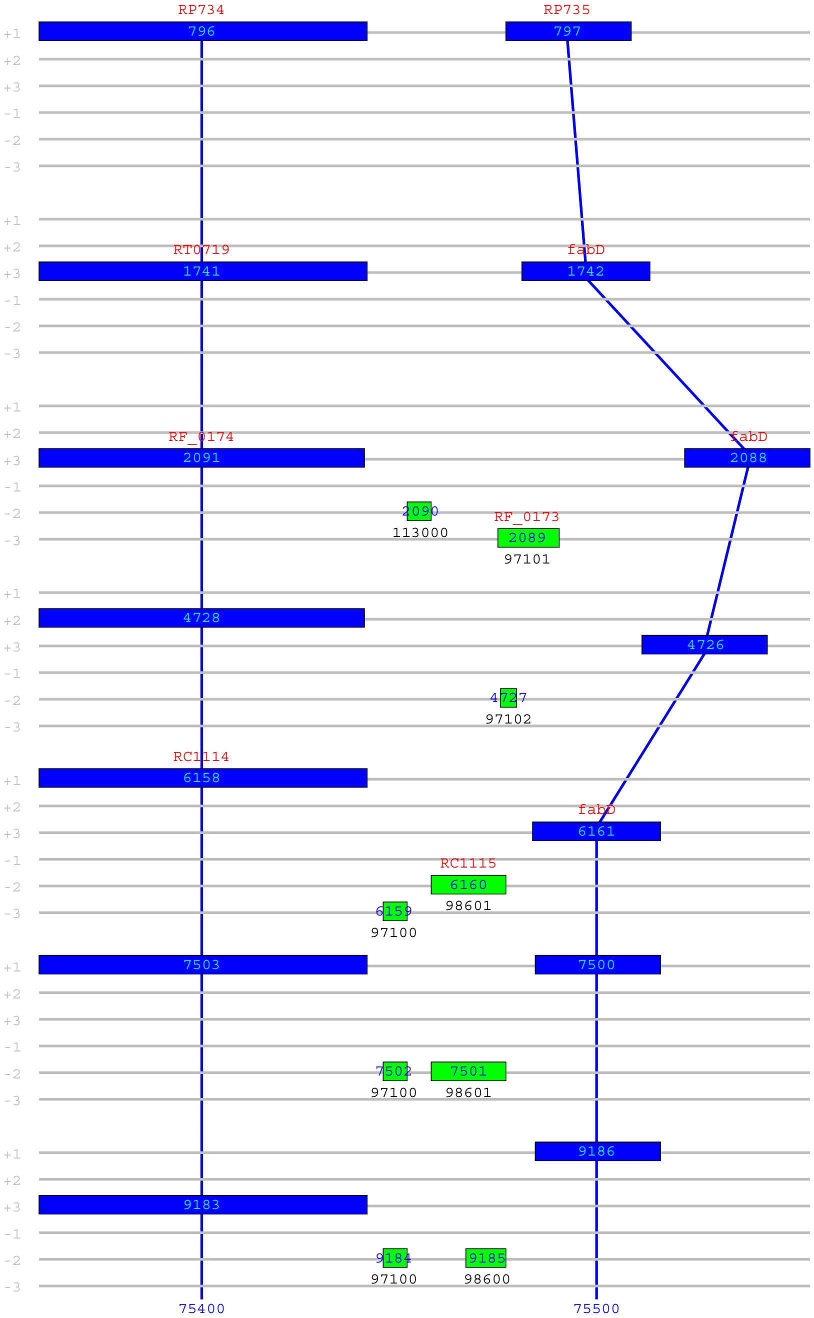

1 Rickettsia prowazekii str. Madrid E, complete genome
2 Rickettsia typhi str. wilmington, complete genome
3 Rickettsia felis URRWXCal2, complete genome
4 Rickettsia akari str. Hartford chromosome, whole genome shotgun sequence
5 Rickettsia conorii str. Malish 7, complete genome
6 Rickettsia sibirica 246 rsib_agncrt, whole genome shotgun sequence
7 Rickettsia rickettsii chromosome, whole genome shotgun sequence

Reg_id: 234

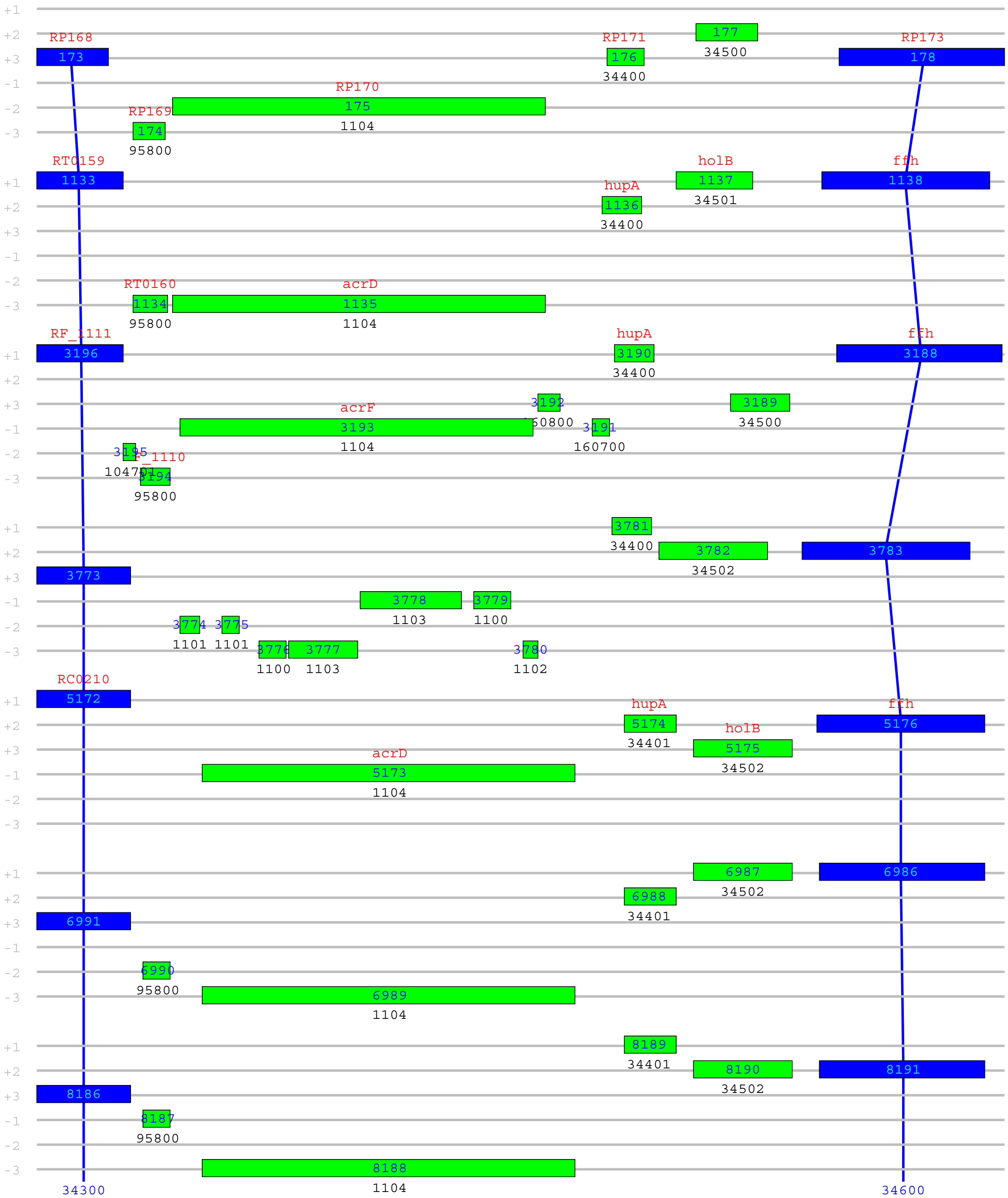

1 Rickettsia prowazekii str. Madrid E, complete genome
2 Rickettsia typhi str. wilmington, complete genome
3 Rickettsia felis URRWXCal2, complete genome
4 Rickettsia akari str. Hartford chromosome, whole genome shotgun sequence
5 Rickettsia conorii str. Malish 7, complete genome
6 Rickettsia sibirica 246 rsib_agncrt, whole genome shotgun sequence
7 Rickettsia rickettsii chromosome, whole genome shotgun sequence

Reg_id: 235

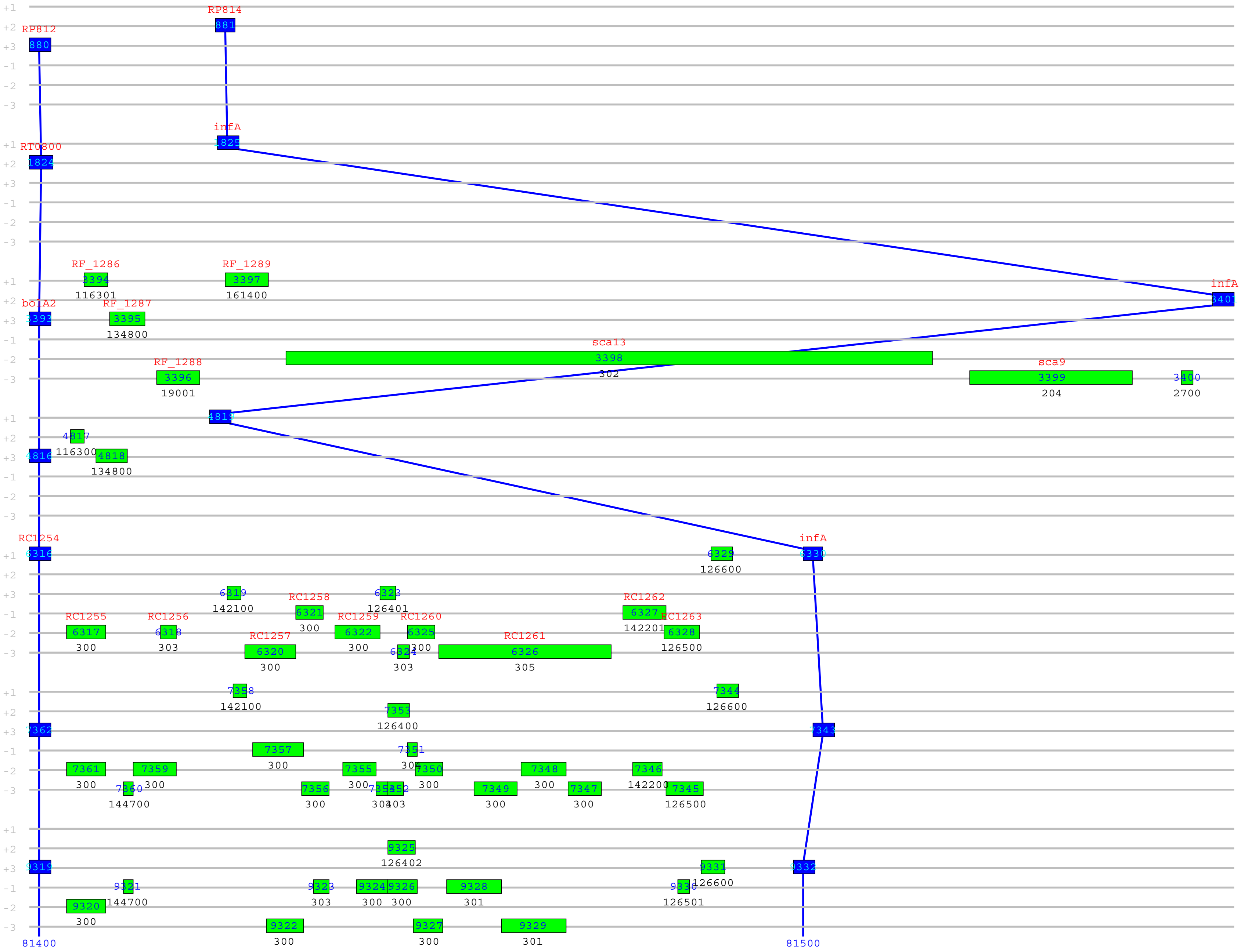

1 Rickettsia prowazekii str. Madrid E, complete genome
2 Rickettsia typhi str. wilmington, complete genome
3 Rickettsia felis URRWXCal2, complete genome
4 Rickettsia akari str. Hartford chromosome, whole genome shotgun sequence
5 Rickettsia conorii str. Malish 7, complete genome
6 Rickettsia sibirica 246 rsib_agncrt, whole genome shotgun sequence
7 Rickettsia rickettsii chromosome, whole genome shotgun sequence

Reg_id: 240

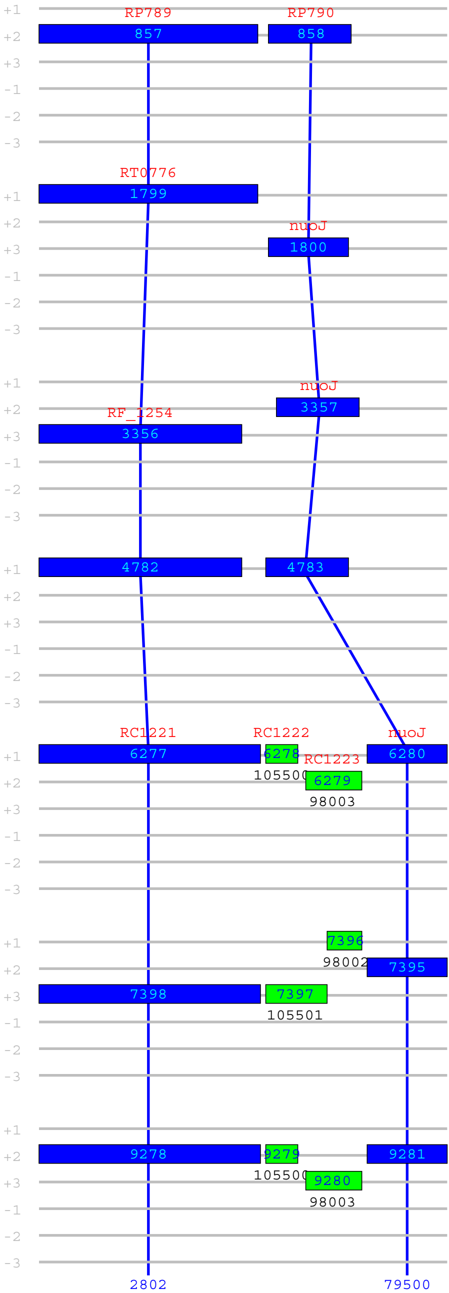

1 Rickettsia prowazekii str. Madrid E, complete genome
2 Rickettsia typhi str. wilmington, complete genome
3 Rickettsia felis URRWXCal2, complete genome
4 Rickettsia akari str. Hartford chromosome, whole genome shotgun sequence
5 Rickettsia conorii str. Malish 7, complete genome
6 Rickettsia sibirica 246 rsib_agncrt, whole genome shotgun sequence
7 Rickettsia rickettsii chromosome, whole genome shotgun sequence

Reg_id: 244

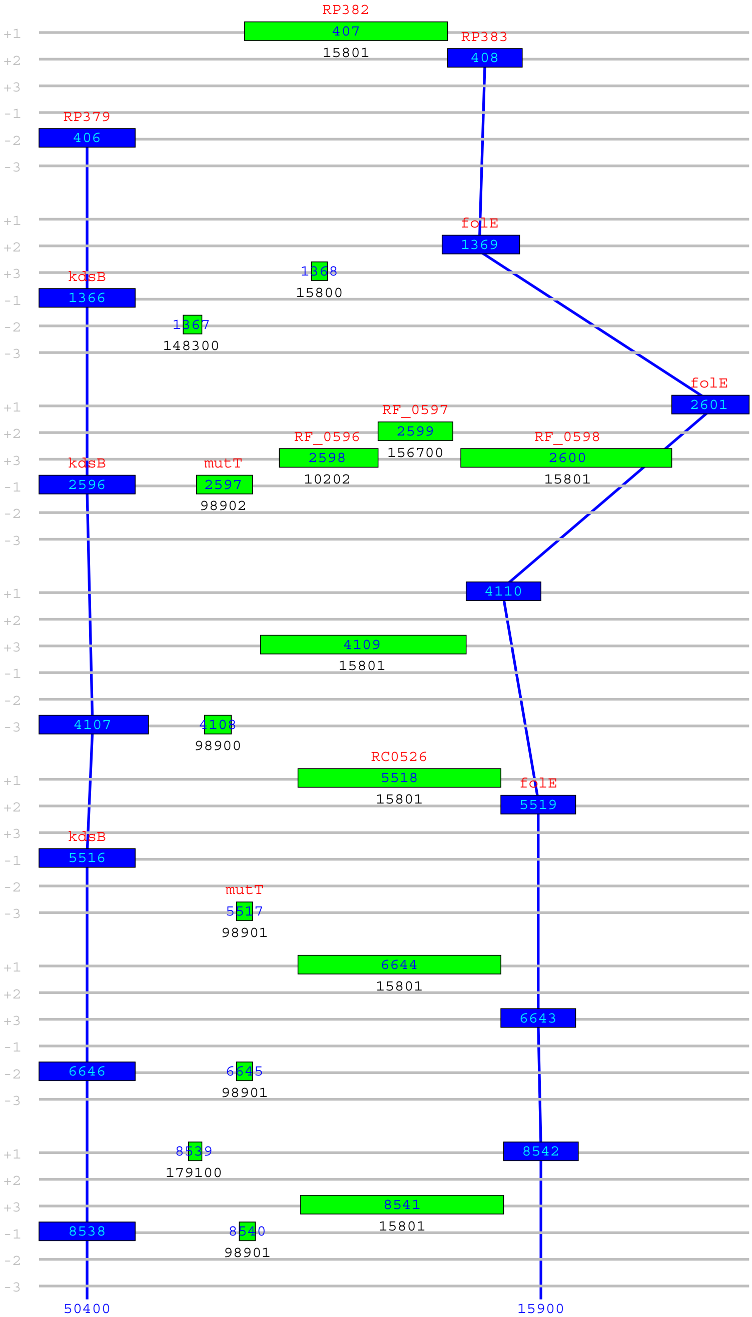

1 Rickettsia prowazekii str. Madrid E, complete genome
2 Rickettsia typhi str. wilmington, complete genome
3 Rickettsia felis URRWXCal2, complete genome
4 Rickettsia akari str. Hartford chromosome, whole genome shotgun sequence
5 Rickettsia conorii str. Malish 7, complete genome
6 Rickettsia sibirica 246 rsib_agncrt, whole genome shotgun sequence
7 Rickettsia rickettsii chromosome, whole genome shotgun sequence

Reg_id: 245

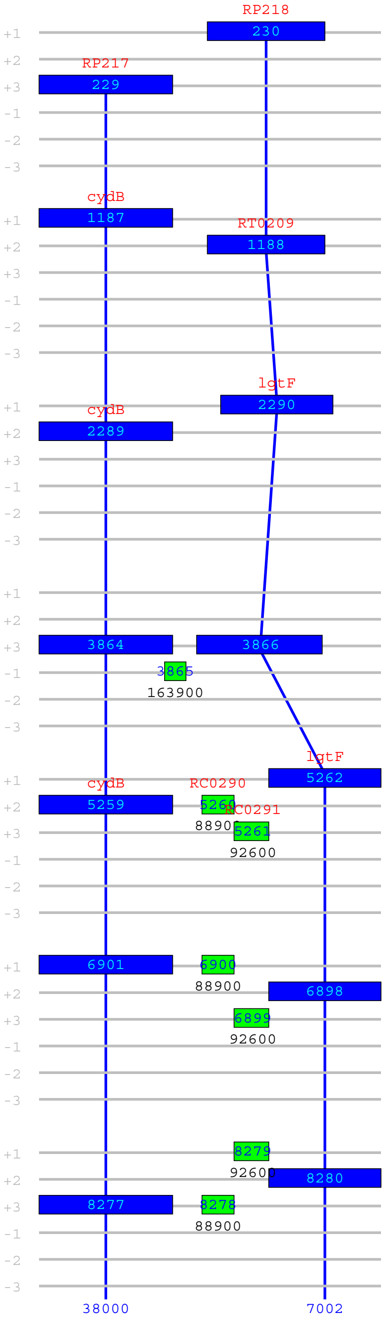

1 Rickettsia prowazekii str. Madrid E, complete genome
2 Rickettsia typhi str. wilmington, complete genome
3 Rickettsia felis URRWXCal2, complete genome
4 Rickettsia akari str. Hartford chromosome, whole genome shotgun sequence
5 Rickettsia conorii str. Malish 7, complete genome
6 Rickettsia sibirica 246 rsib_agncrt, whole genome shotgun sequence
7 Rickettsia rickettsii chromosome, whole genome shotgun sequence

Reg_id: 246

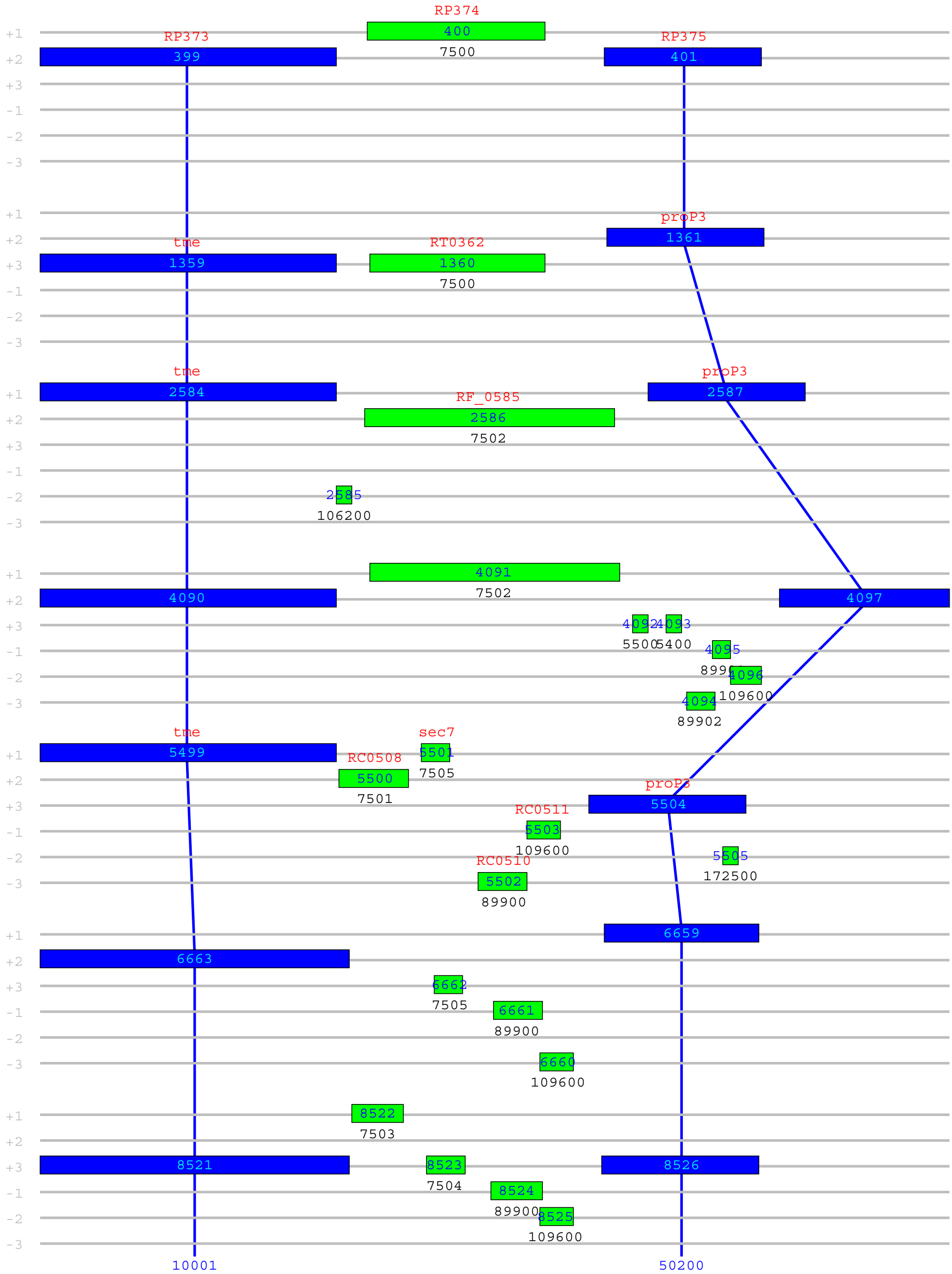

1 Rickettsia prowazekii str. Madrid E, complete genome
2 Rickettsia typhi str. wilmington, complete genome
3 Rickettsia felis URRWXCal2, complete genome
4 Rickettsia akari str. Hartford chromosome, whole genome shotgun sequence
5 Rickettsia conorii str. Malish 7, complete genome
6 Rickettsia sibirica 246 rsib_agncrt, whole genome shotgun sequence
7 Rickettsia rickettsii chromosome, whole genome shotgun sequence

Reg_id: 247

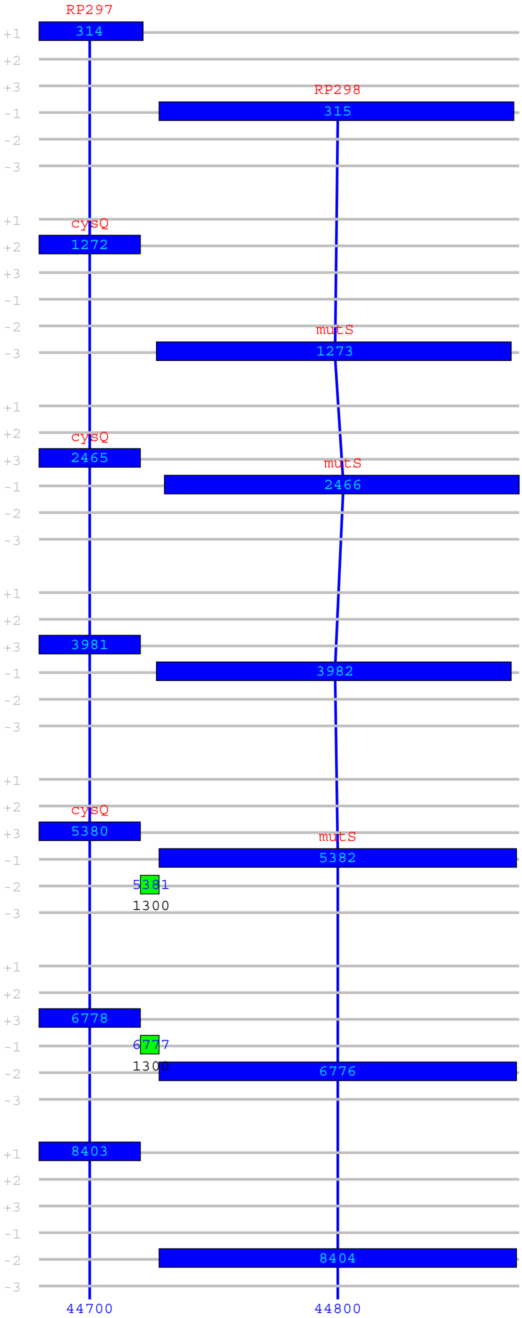

1 Rickettsia prowazekii str. Madrid E, complete genome
2 Rickettsia typhi str. wilmington, complete genome
3 Rickettsia felis URRWXCal2, complete genome
4 Rickettsia akari str. Hartford chromosome, whole genome shotgun sequence
5 Rickettsia conorii str. Malish 7, complete genome
6 Rickettsia sibirica 246 rsib_agncrt, whole genome shotgun sequence
7 Rickettsia rickettsii chromosome, whole genome shotgun sequence

Reg_id: 248

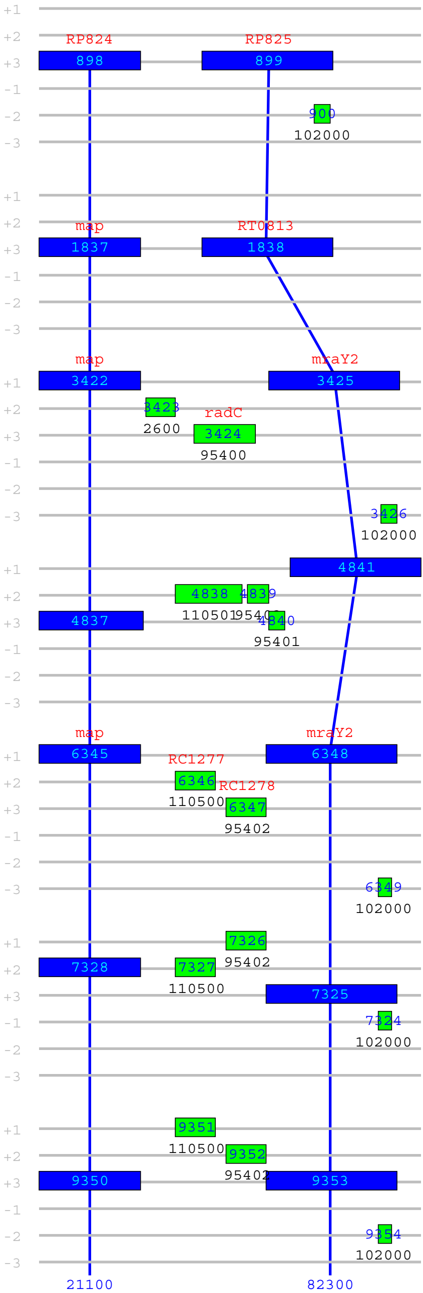

1 Rickettsia prowazekii str. Madrid E, complete genome
2 Rickettsia typhi str. wilmington, complete genome
3 Rickettsia felis URRWXCal2, complete genome
4 Rickettsia akari str. Hartford chromosome, whole genome shotgun sequence
5 Rickettsia conorii str. Malish 7, complete genome
6 Rickettsia sibirica 246 rsib_agncrt, whole genome shotgun sequence
7 Rickettsia rickettsii chromosome, whole genome shotgun sequence

Reg_id: 249

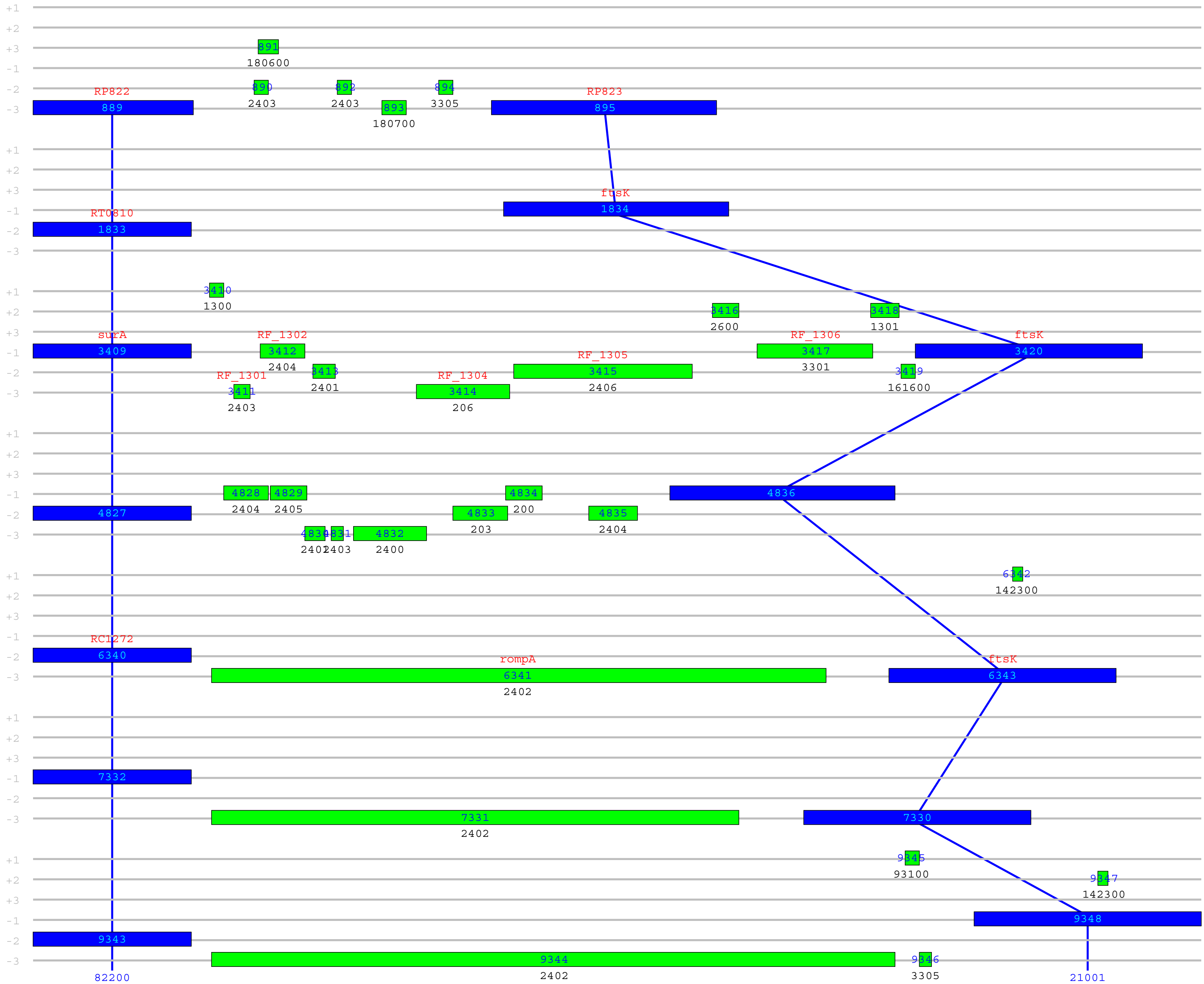

1 Rickettsia prowazekii str. Madrid E, complete genome
2 Rickettsia typhi str. wilmington, complete genome
3 Rickettsia felis URRWXCal2, complete genome
4 Rickettsia akari str. Hartford chromosome, whole genome shotgun sequence
5 Rickettsia conorii str. Malish 7, complete genome
6 Rickettsia sibirica 246 rsib_agncrt, whole genome shotgun sequence
7 Rickettsia rickettsii chromosome, whole genome shotgun sequence

Reg_id: 250

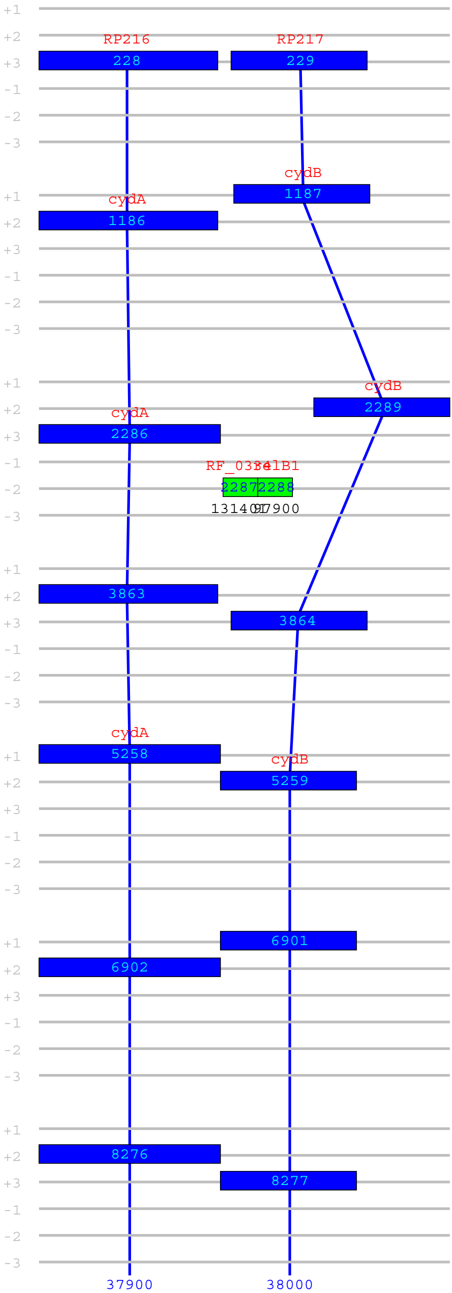

1 Rickettsia prowazekii str. Madrid E, complete genome
2 Rickettsia typhi str. wilmington, complete genome
3 Rickettsia felis URRWXCal2, complete genome
4 Rickettsia akari str. Hartford chromosome, whole genome shotgun sequence
5 Rickettsia conorii str. Malish 7, complete genome
6 Rickettsia sibirica 246 rsib_agncrt, whole genome shotgun sequence
7 Rickettsia rickettsii chromosome, whole genome shotgun sequence

Reg_id: 251

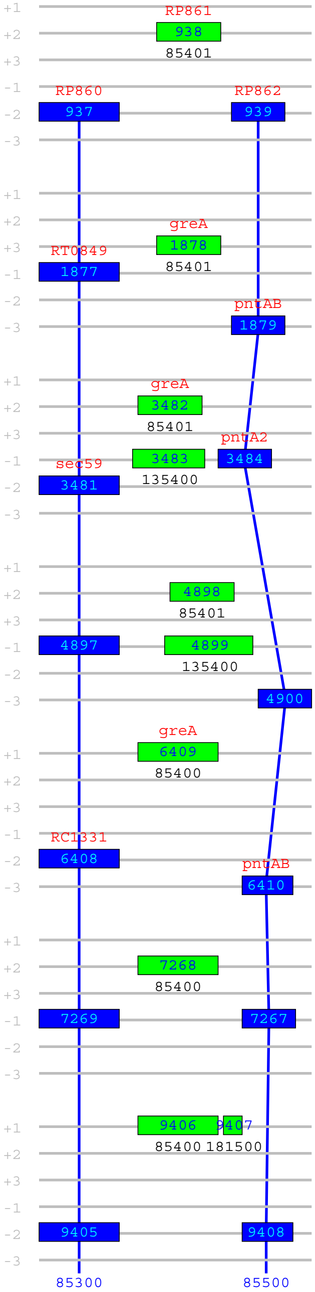

1 Rickettsia prowazekii str. Madrid E, complete genome
2 Rickettsia typhi str. wilmington, complete genome
3 Rickettsia felis URRWXCal2, complete genome
4 Rickettsia akari str. Hartford chromosome, whole genome shotgun sequence
5 Rickettsia conorii str. Malish 7, complete genome
6 Rickettsia sibirica 246 rsib_agncrt, whole genome shotgun sequence
7 Rickettsia rickettsii chromosome, whole genome shotgun sequence

Reg_id: 252

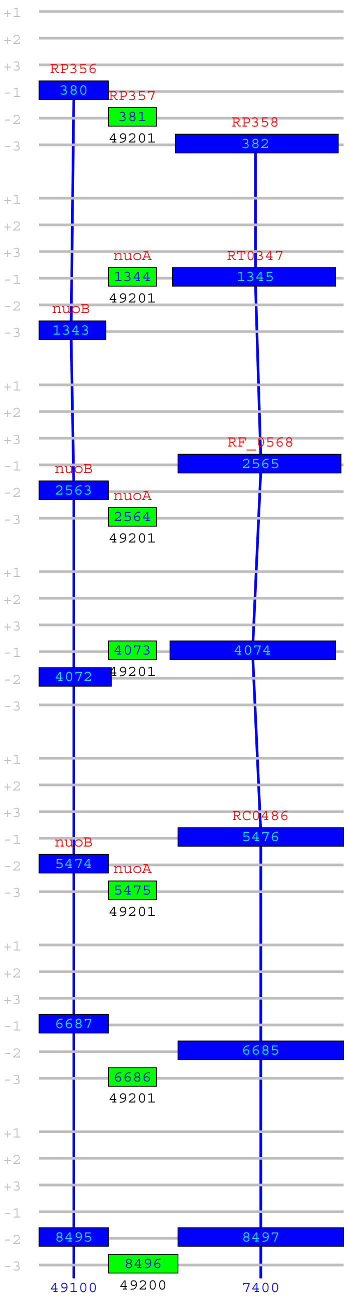

1 Rickettsia prowazekii str. Madrid E, complete genome
2 Rickettsia typhi str. wilmington, complete genome
3 Rickettsia felis URRWXCal2, complete genome
4 Rickettsia akari str. Hartford chromosome, whole genome shotgun sequence
5 Rickettsia conorii str. Malish 7, complete genome
6 Rickettsia sibirica 246 rsib_agncrt, whole genome shotgun sequence
7 Rickettsia rickettsii chromosome, whole genome shotgun sequence

Reg_id: 256

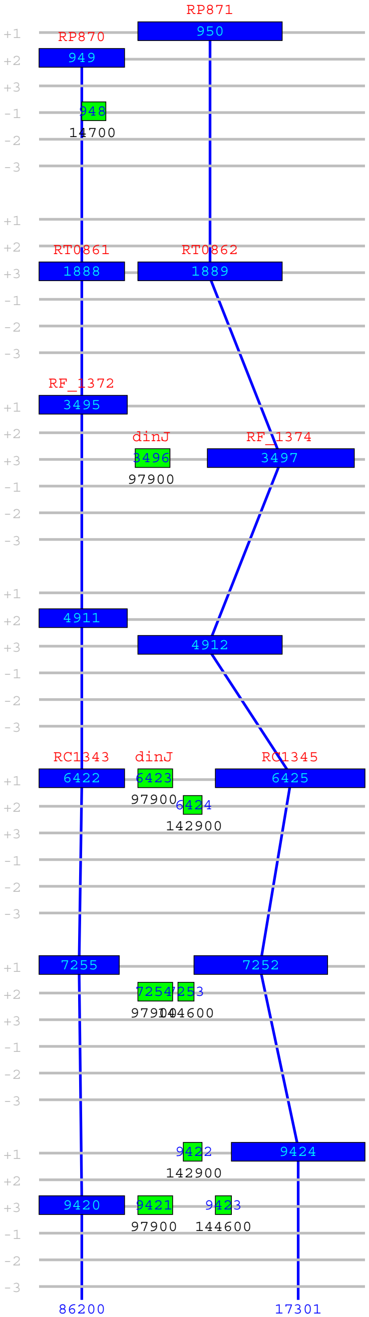

1 Rickettsia prowazekii str. Madrid E, complete genome
2 Rickettsia typhi str. wilmington, complete genome
3 Rickettsia felis URRWXCal2, complete genome
4 Rickettsia akari str. Hartford chromosome, whole genome shotgun sequence
5 Rickettsia conorii str. Malish 7, complete genome
6 Rickettsia sibirica 246 rsib_agncrt, whole genome shotgun sequence
7 Rickettsia rickettsii chromosome, whole genome shotgun sequence

Reg_id: 257

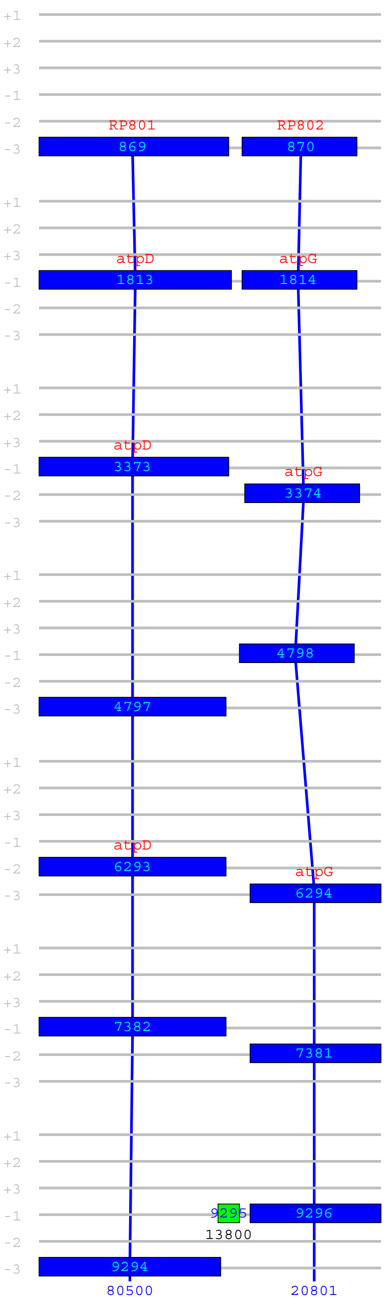

1 Rickettsia prowazekii str. Madrid E, complete genome
2 Rickettsia typhi str. wilmington, complete genome
3 Rickettsia felis URRWXCal2, complete genome
4 Rickettsia akari str. Hartford chromosome, whole genome shotgun sequence
5 Rickettsia conorii str. Malish 7, complete genome
6 Rickettsia sibirica 246 rsib_agncrt, whole genome shotgun sequence
7 Rickettsia rickettsii chromosome, whole genome shotgun sequence

Reg_id: 258

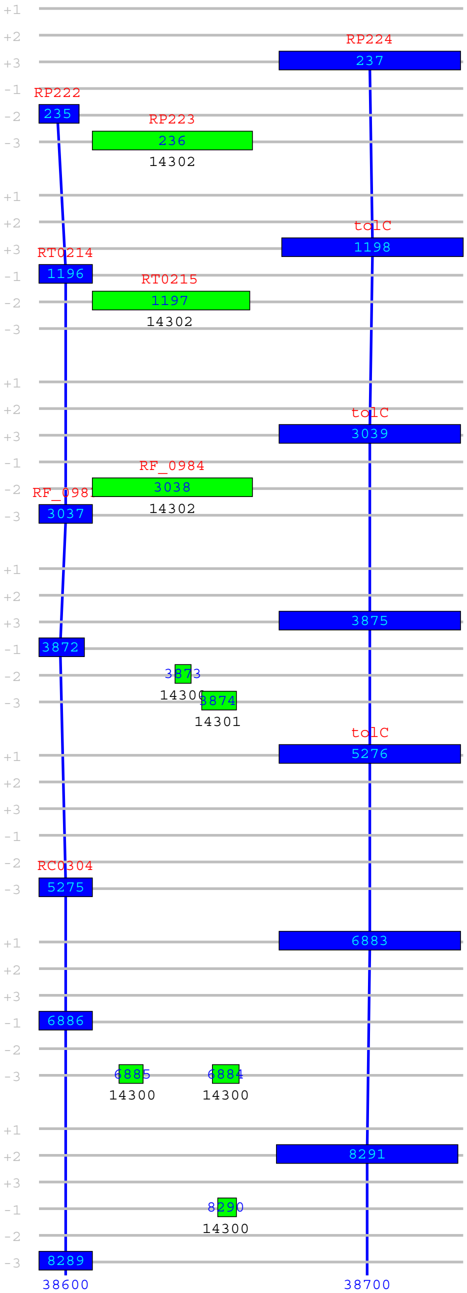

1 Rickettsia prowazekii str. Madrid E, complete genome
2 Rickettsia typhi str. wilmington, complete genome
3 Rickettsia felis URRWXCal2, complete genome
4 Rickettsia akari str. Hartford chromosome, whole genome shotgun sequence
5 Rickettsia conorii str. Malish 7, complete genome
6 Rickettsia sibirica 246 rsib_agncrt, whole genome shotgun sequence
7 Rickettsia rickettsii chromosome, whole genome shotgun sequence

Reg_id: 262

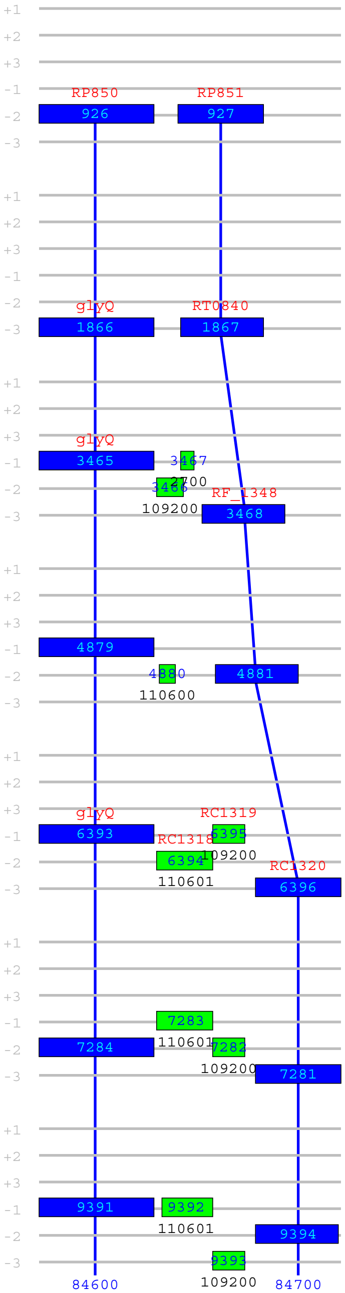

1 Rickettsia prowazekii str. Madrid E, complete genome
2 Rickettsia typhi str. wilmington, complete genome
3 Rickettsia felis URRWXCal2, complete genome
4 Rickettsia akari str. Hartford chromosome, whole genome shotgun sequence
5 Rickettsia conorii str. Malish 7, complete genome
6 Rickettsia sibirica 246 rsib_agncrt, whole genome shotgun sequence
7 Rickettsia rickettsii chromosome, whole genome shotgun sequence

Reg_id: 263

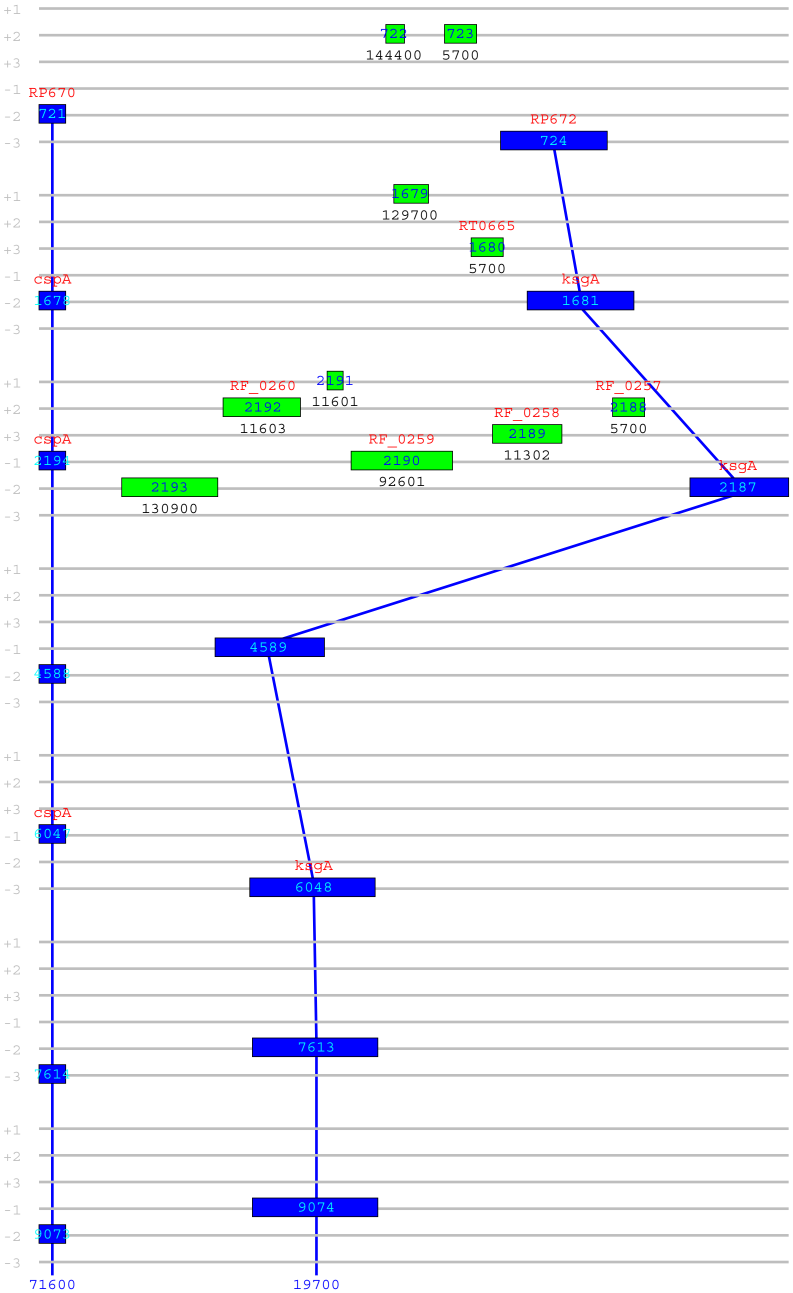

1 Rickettsia prowazekii str. Madrid E, complete genome
2 Rickettsia typhi str. wilmington, complete genome
3 Rickettsia felis URRWXCal2, complete genome
4 Rickettsia akari str. Hartford chromosome, whole genome shotgun sequence
5 Rickettsia conorii str. Malish 7, complete genome
6 Rickettsia sibirica 246 rsib_agncrt, whole genome shotgun sequence
7 Rickettsia rickettsii chromosome, whole genome shotgun sequence

Reg_id: 264

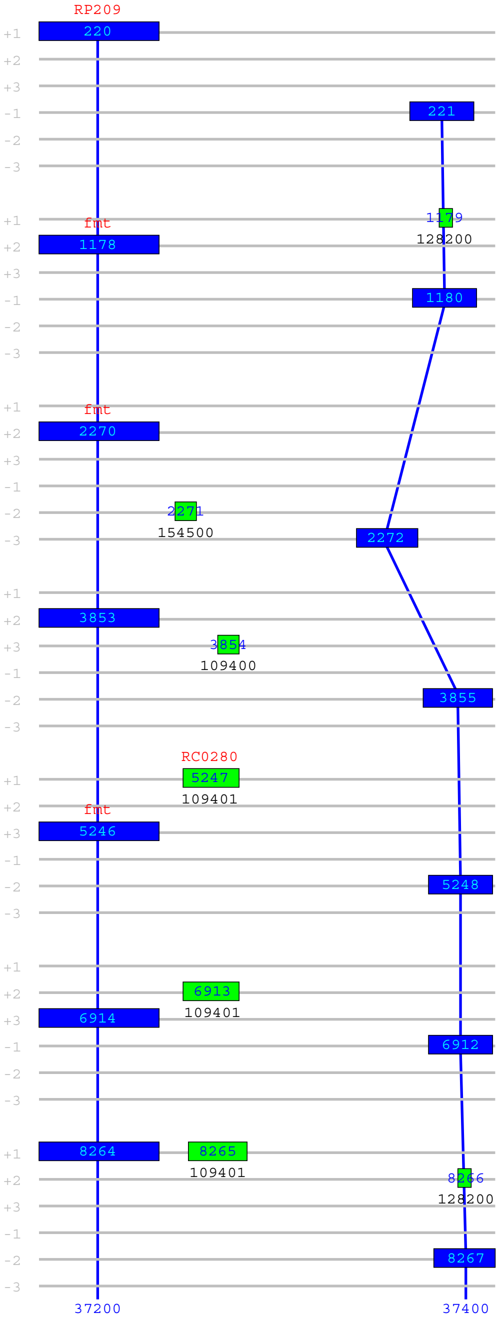

1 Rickettsia prowazekii str. Madrid E, complete genome
2 Rickettsia typhi str. wilmington, complete genome
3 Rickettsia felis URRWXCal2, complete genome
4 Rickettsia akari str. Hartford chromosome, whole genome shotgun sequence
5 Rickettsia conorii str. Malish 7, complete genome
6 Rickettsia sibirica 246 rsib_agncrt, whole genome shotgun sequence
7 Rickettsia rickettsii chromosome, whole genome shotgun sequence

Reg_id: 265

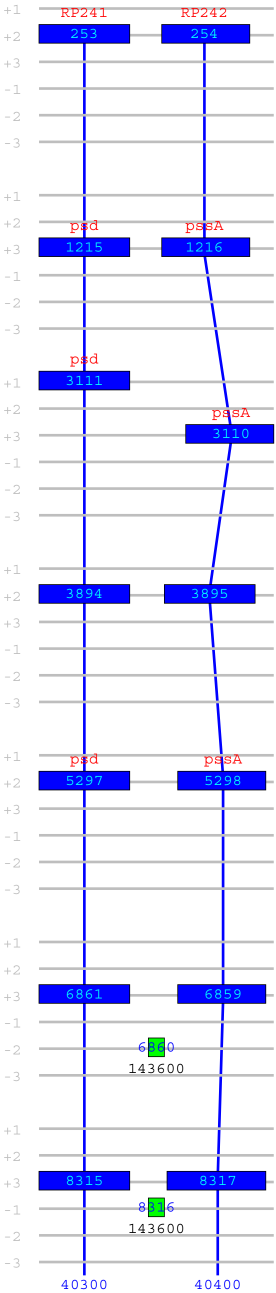

1 Rickettsia prowazekii str. Madrid E, complete genome
2 Rickettsia typhi str. wilmington, complete genome
3 Rickettsia felis URRWXCal2, complete genome
4 Rickettsia akari str. Hartford chromosome, whole genome shotgun sequence
5 Rickettsia conorii str. Malish 7, complete genome
6 Rickettsia sibirica 246 rsib_agncrt, whole genome shotgun sequence
7 Rickettsia rickettsii chromosome, whole genome shotgun sequence

Reg_id: 266

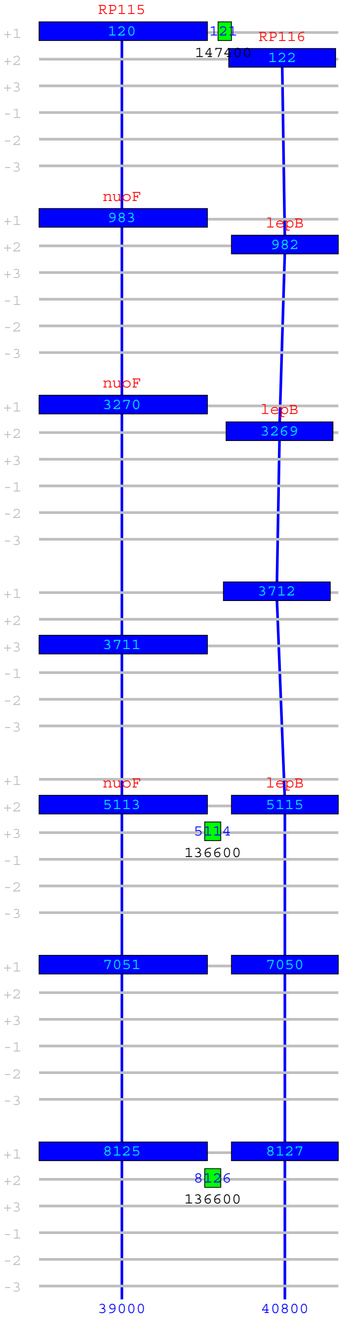

1 Rickettsia prowazekii str. Madrid E, complete genome
2 Rickettsia typhi str. wilmington, complete genome
3 Rickettsia felis URRWXCal2, complete genome
4 Rickettsia akari str. Hartford chromosome, whole genome shotgun sequence
5 Rickettsia conorii str. Malish 7, complete genome
6 Rickettsia sibirica 246 rsib_agncrt, whole genome shotgun sequence
7 Rickettsia rickettsii chromosome, whole genome shotgun sequence

Reg_id: 267

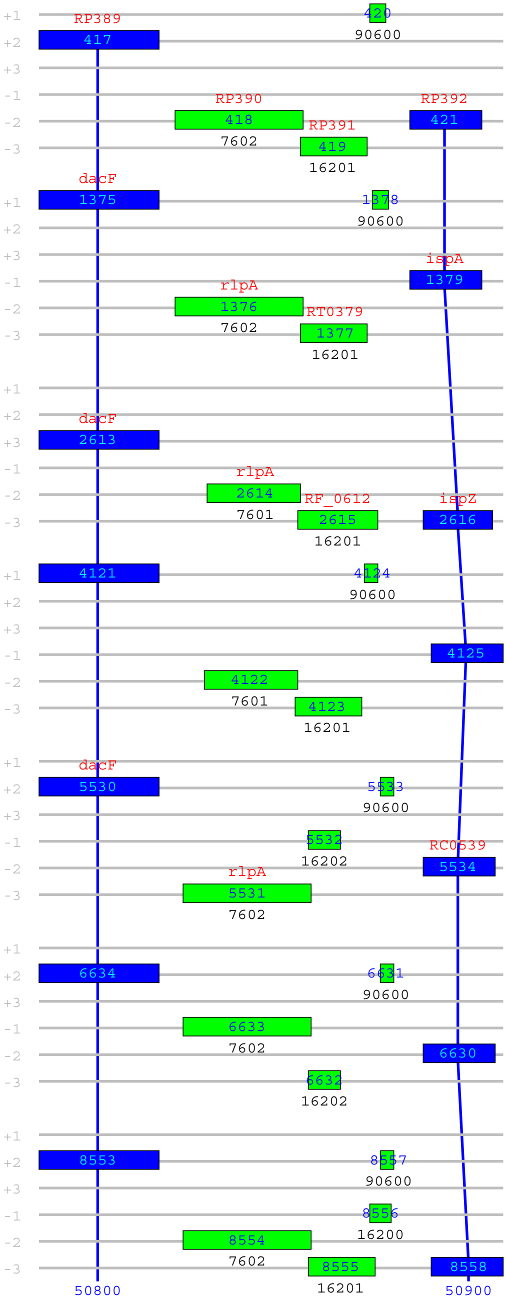

1 Rickettsia prowazekii str. Madrid E, complete genome
2 Rickettsia typhi str. wilmington, complete genome
3 Rickettsia felis URRWXCal2, complete genome
4 Rickettsia akari str. Hartford chromosome, whole genome shotgun sequence
5 Rickettsia conorii str. Malish 7, complete genome
6 Rickettsia sibirica 246 rsib_agncrt, whole genome shotgun sequence
7 Rickettsia rickettsii chromosome, whole genome shotgun sequence

Reg_id: 268

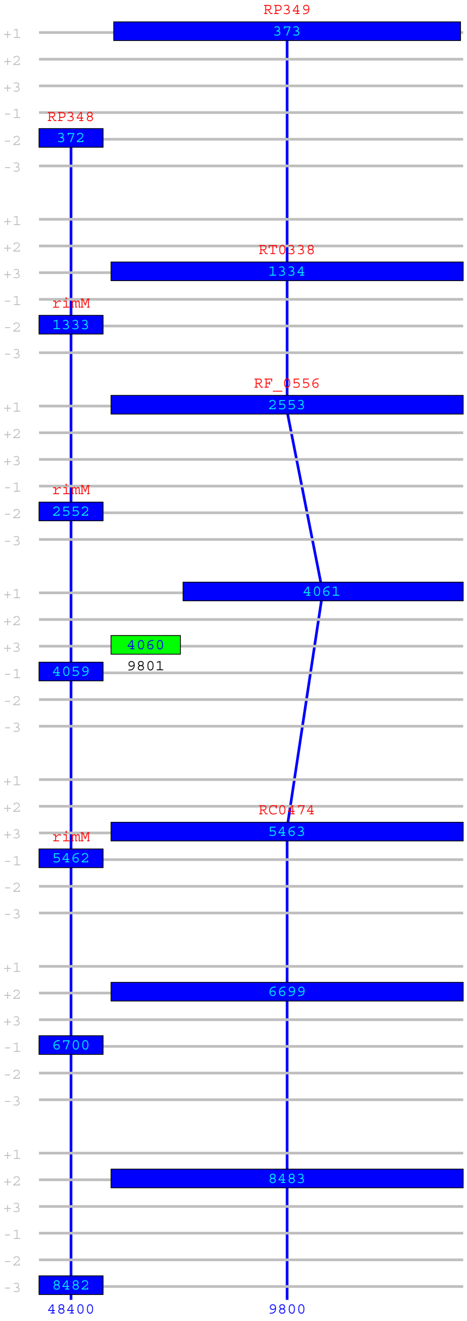

1 Rickettsia prowazekii str. Madrid E, complete genome
2 Rickettsia typhi str. wilmington, complete genome
3 Rickettsia felis URRWXCal2, complete genome
4 Rickettsia akari str. Hartford chromosome, whole genome shotgun sequence
5 Rickettsia conorii str. Malish 7, complete genome
6 Rickettsia sibirica 246 rsib_agncrt, whole genome shotgun sequence
7 Rickettsia rickettsii chromosome, whole genome shotgun sequence

Reg_id: 274

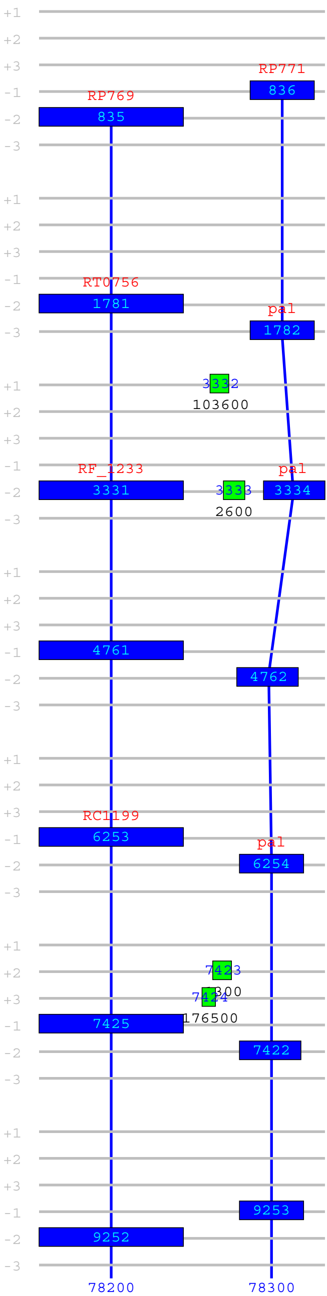

1 Rickettsia prowazekii str. Madrid E, complete genome
2 Rickettsia typhi str. wilmington, complete genome
3 Rickettsia felis URRWXCal2, complete genome
4 Rickettsia akari str. Hartford chromosome, whole genome shotgun sequence
5 Rickettsia conorii str. Malish 7, complete genome
6 Rickettsia sibirica 246 rsib_agncrt, whole genome shotgun sequence
7 Rickettsia rickettsii chromosome, whole genome shotgun sequence

Reg_id: 276

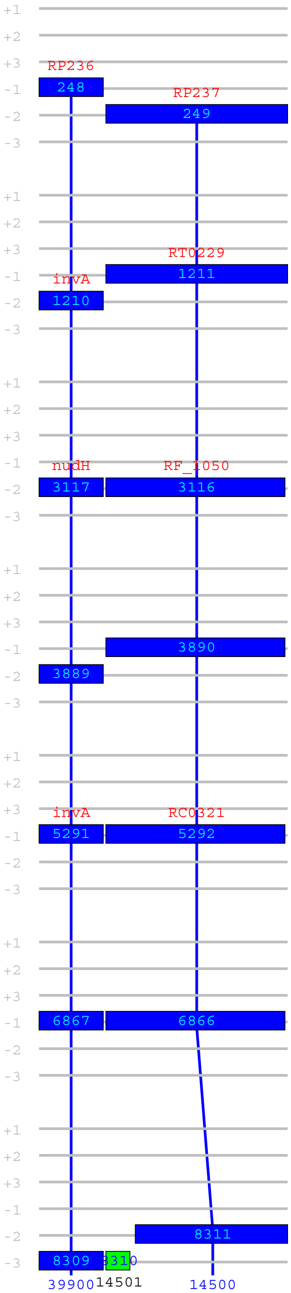

1 Rickettsia prowazekii str. Madrid E, complete genome
2 Rickettsia typhi str. wilmington, complete genome
3 Rickettsia felis URRWXCal2, complete genome
4 Rickettsia akari str. Hartford chromosome, whole genome shotgun sequence
5 Rickettsia conorii str. Malish 7, complete genome
6 Rickettsia sibirica 246 rsib_agncrt, whole genome shotgun sequence
7 Rickettsia rickettsii chromosome, whole genome shotgun sequence

Reg_id: 278

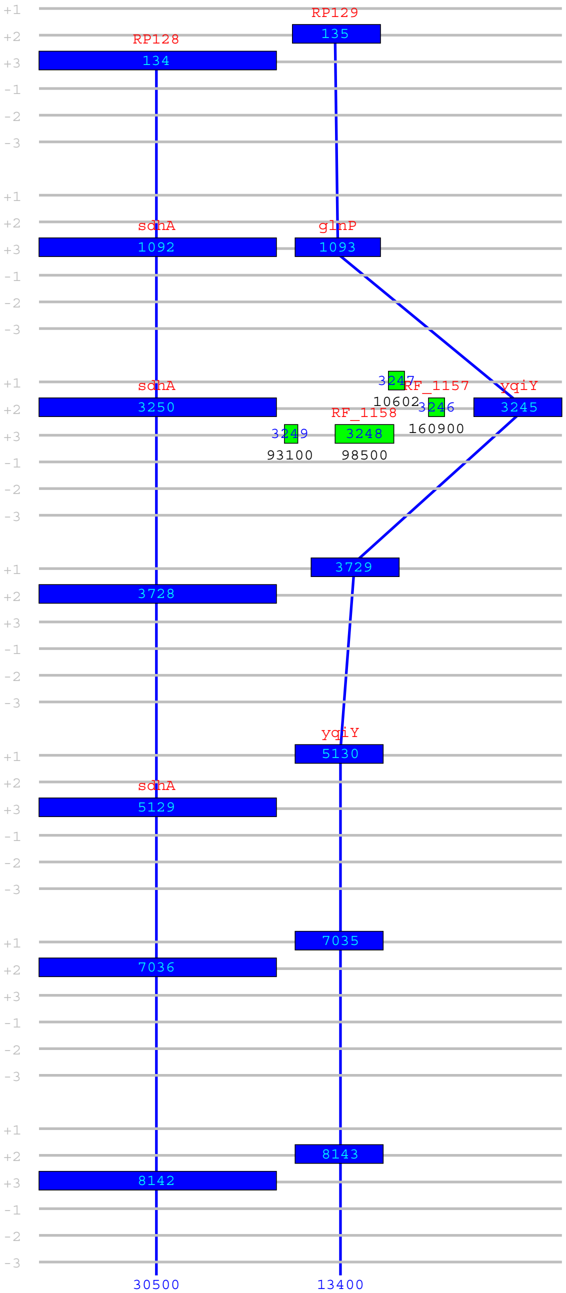

1 Rickettsia prowazekii str. Madrid E, complete genome
2 Rickettsia typhi str. wilmington, complete genome
3 Rickettsia felis URRWXCal2, complete genome
4 Rickettsia akari str. Hartford chromosome, whole genome shotgun sequence
5 Rickettsia conorii str. Malish 7, complete genome
6 Rickettsia sibirica 246 rsib_agncrt, whole genome shotgun sequence
7 Rickettsia rickettsii chromosome, whole genome shotgun sequence

Reg_id: 280

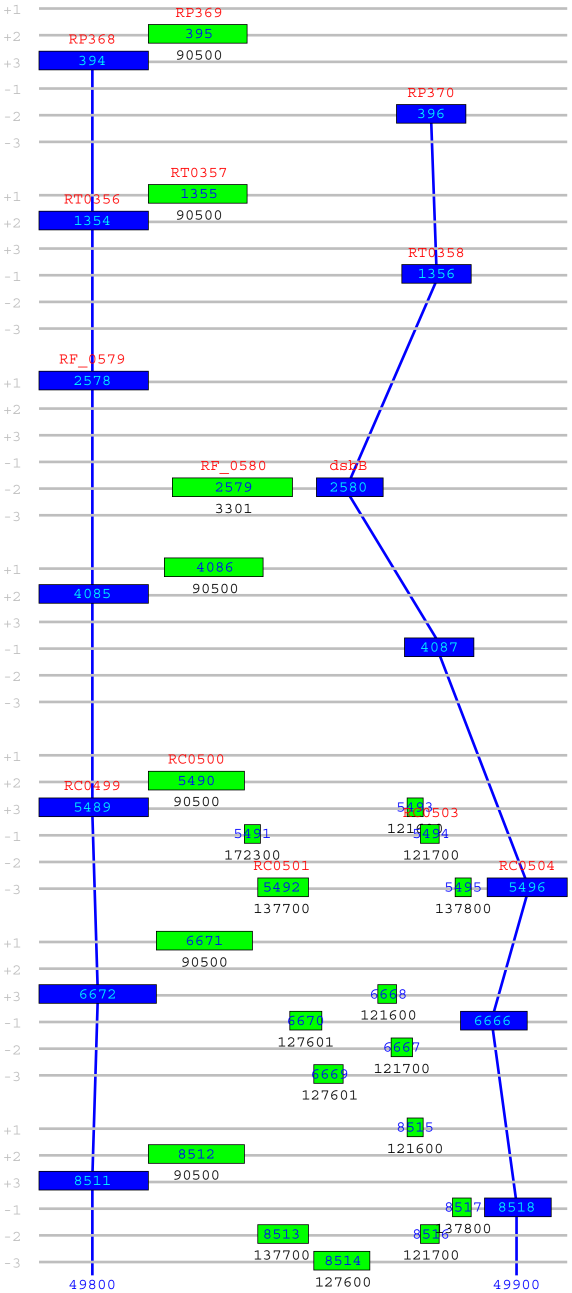

1 Rickettsia prowazekii str. Madrid E, complete genome
2 Rickettsia typhi str. wilmington, complete genome
3 Rickettsia felis URRWXCal2, complete genome
4 Rickettsia akari str. Hartford chromosome, whole genome shotgun sequence
5 Rickettsia conorii str. Malish 7, complete genome
6 Rickettsia sibirica 246 rsib_agncrt, whole genome shotgun sequence
7 Rickettsia rickettsii chromosome, whole genome shotgun sequence

Reg_id: 283

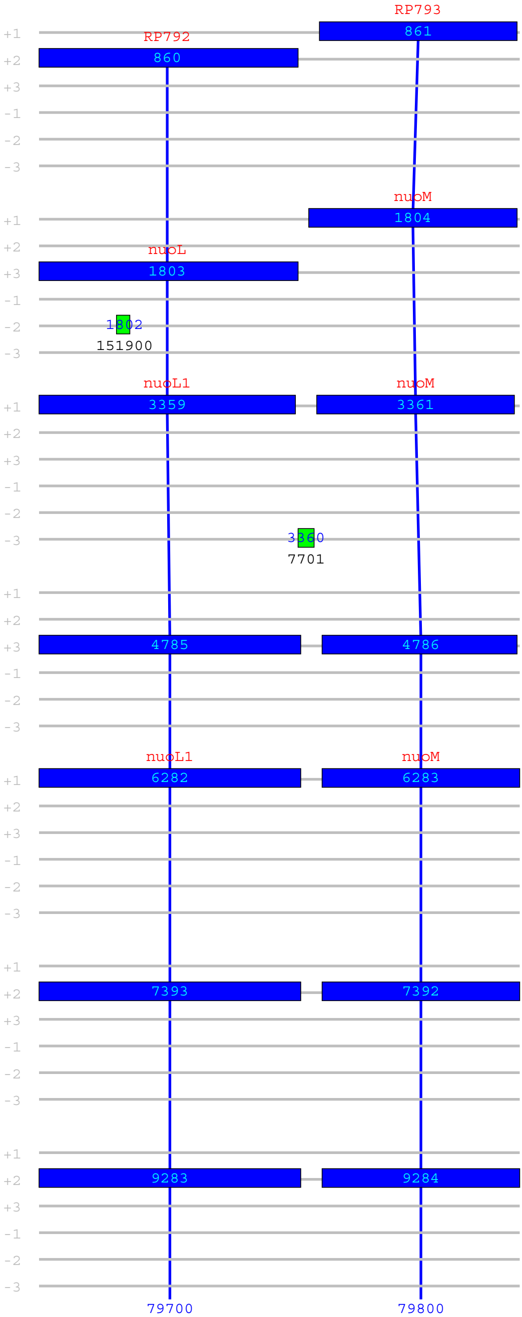

1 Rickettsia prowazekii str. Madrid E, complete genome
2 Rickettsia typhi str. wilmington, complete genome
3 Rickettsia felis URRWXCal2, complete genome
4 Rickettsia akari str. Hartford chromosome, whole genome shotgun sequence
5 Rickettsia conorii str. Malish 7, complete genome
6 Rickettsia sibirica 246 rsib_agncrt, whole genome shotgun sequence
7 Rickettsia rickettsii chromosome, whole genome shotgun sequence

Reg_id: 287

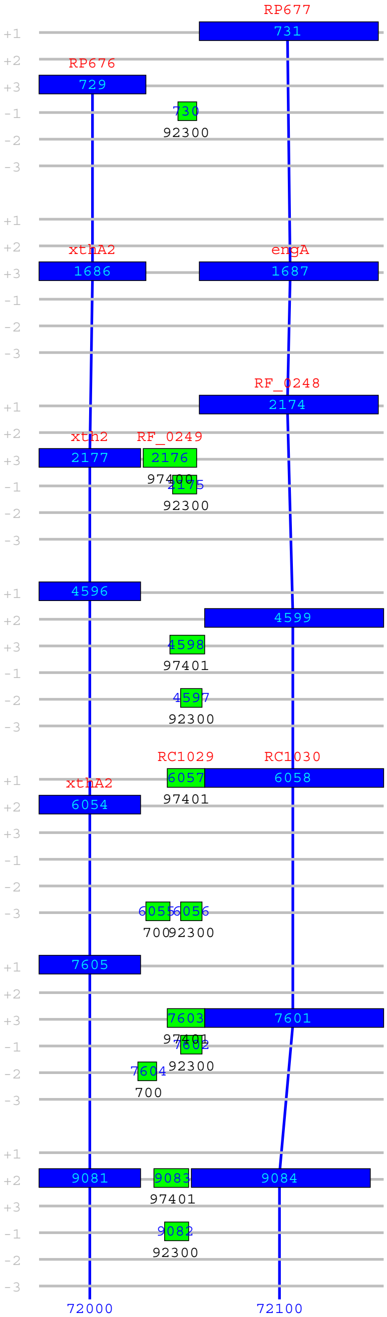

1 Rickettsia prowazekii str. Madrid E, complete genome
2 Rickettsia typhi str. wilmington, complete genome
3 Rickettsia felis URRWXCal2, complete genome
4 Rickettsia akari str. Hartford chromosome, whole genome shotgun sequence
5 Rickettsia conorii str. Malish 7, complete genome
6 Rickettsia sibirica 246 rsib_agncrt, whole genome shotgun sequence
7 Rickettsia rickettsii chromosome, whole genome shotgun sequence

Reg_id: 289

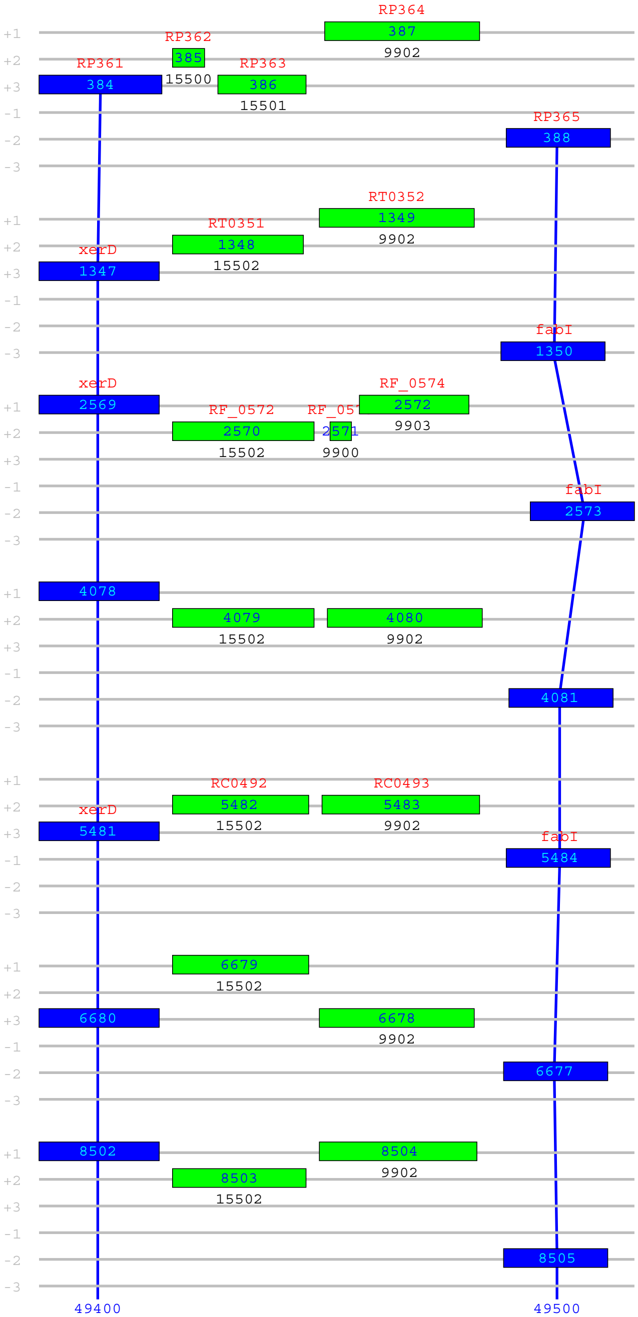

1 Rickettsia prowazekii str. Madrid E, complete genome
2 Rickettsia typhi str. wilmington, complete genome
3 Rickettsia felis URRWXCal2, complete genome
4 Rickettsia akari str. Hartford chromosome, whole genome shotgun sequence
5 Rickettsia conorii str. Malish 7, complete genome
6 Rickettsia sibirica 246 rsib_agncrt, whole genome shotgun sequence
7 Rickettsia rickettsii chromosome, whole genome shotgun sequence

Reg_id: 291

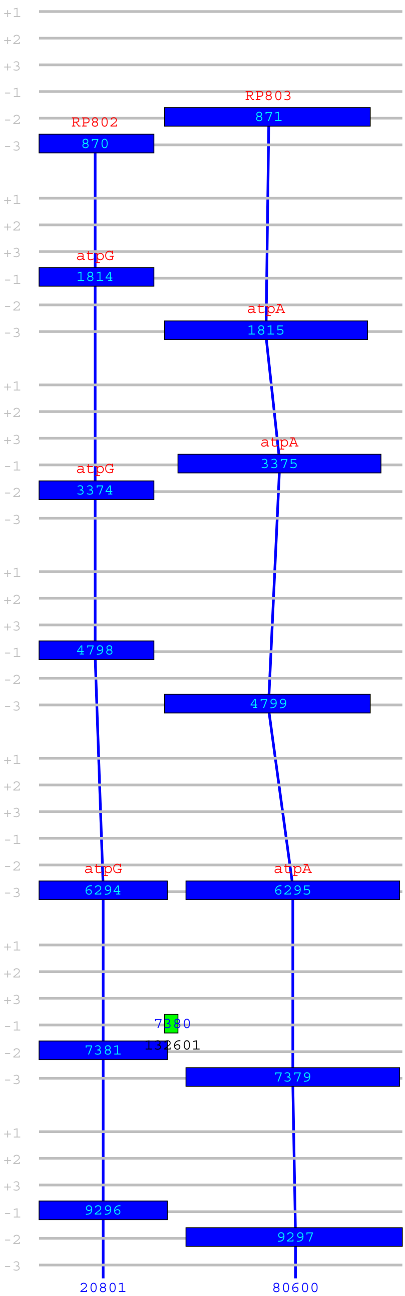

1 Rickettsia prowazekii str. Madrid E, complete genome
2 Rickettsia typhi str. wilmington, complete genome
3 Rickettsia felis URRWXCal2, complete genome
4 Rickettsia akari str. Hartford chromosome, whole genome shotgun sequence
5 Rickettsia conorii str. Malish 7, complete genome
6 Rickettsia sibirica 246 rsib_agncrt, whole genome shotgun sequence
7 Rickettsia rickettsii chromosome, whole genome shotgun sequence

Reg_id: 292

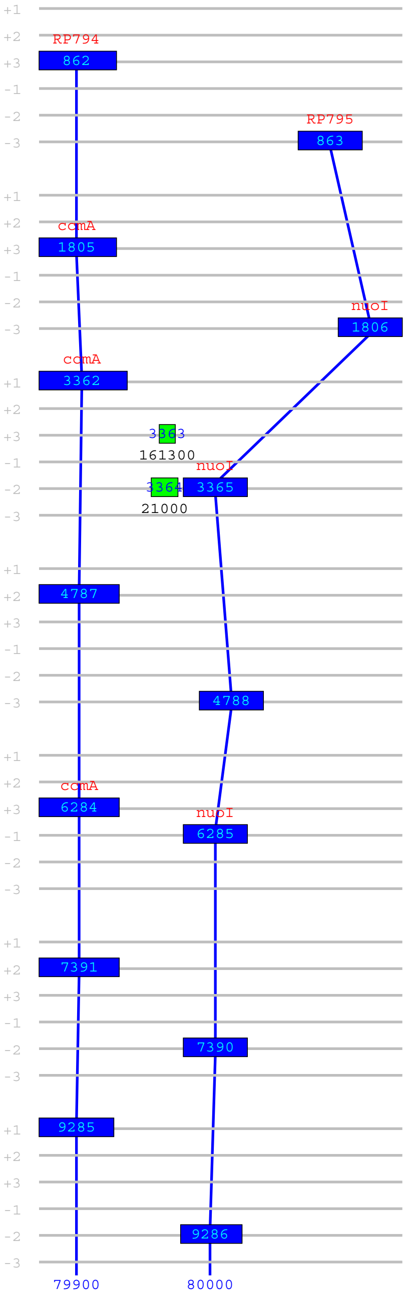

1 Rickettsia prowazekii str. Madrid E, complete genome
2 Rickettsia typhi str. wilmington, complete genome
3 Rickettsia felis URRWXCal2, complete genome
4 Rickettsia akari str. Hartford chromosome, whole genome shotgun sequence
5 Rickettsia conorii str. Malish 7, complete genome
6 Rickettsia sibirica 246 rsib_agncrt, whole genome shotgun sequence
7 Rickettsia rickettsii chromosome, whole genome shotgun sequence

Reg_id: 294

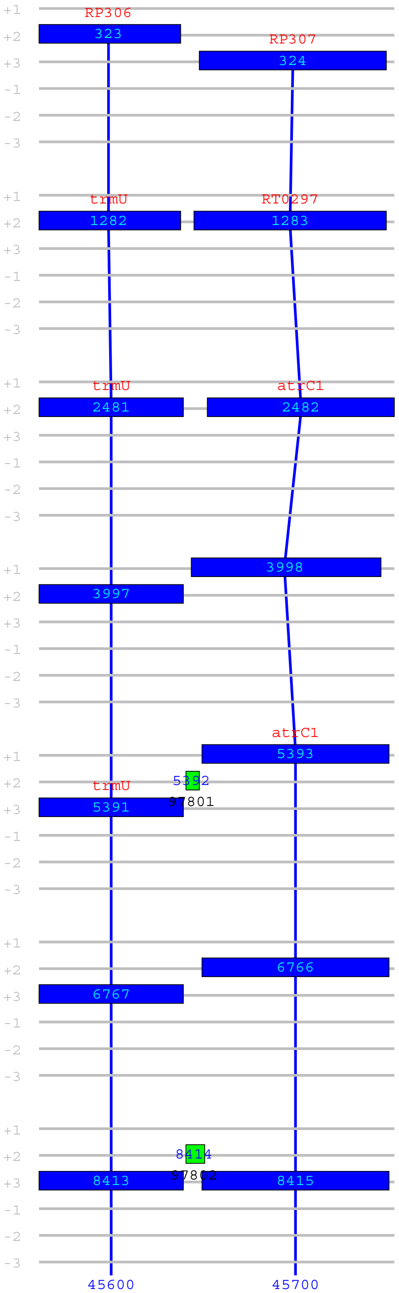

1 Rickettsia prowazekii str. Madrid E, complete genome
2 Rickettsia typhi str. wilmington, complete genome
3 Rickettsia felis URRWXCal2, complete genome
4 Rickettsia akari str. Hartford chromosome, whole genome shotgun sequence
5 Rickettsia conorii str. Malish 7, complete genome
6 Rickettsia sibirica 246 rsib_agncrt, whole genome shotgun sequence
7 Rickettsia rickettsii chromosome, whole genome shotgun sequence

Reg_id: 299

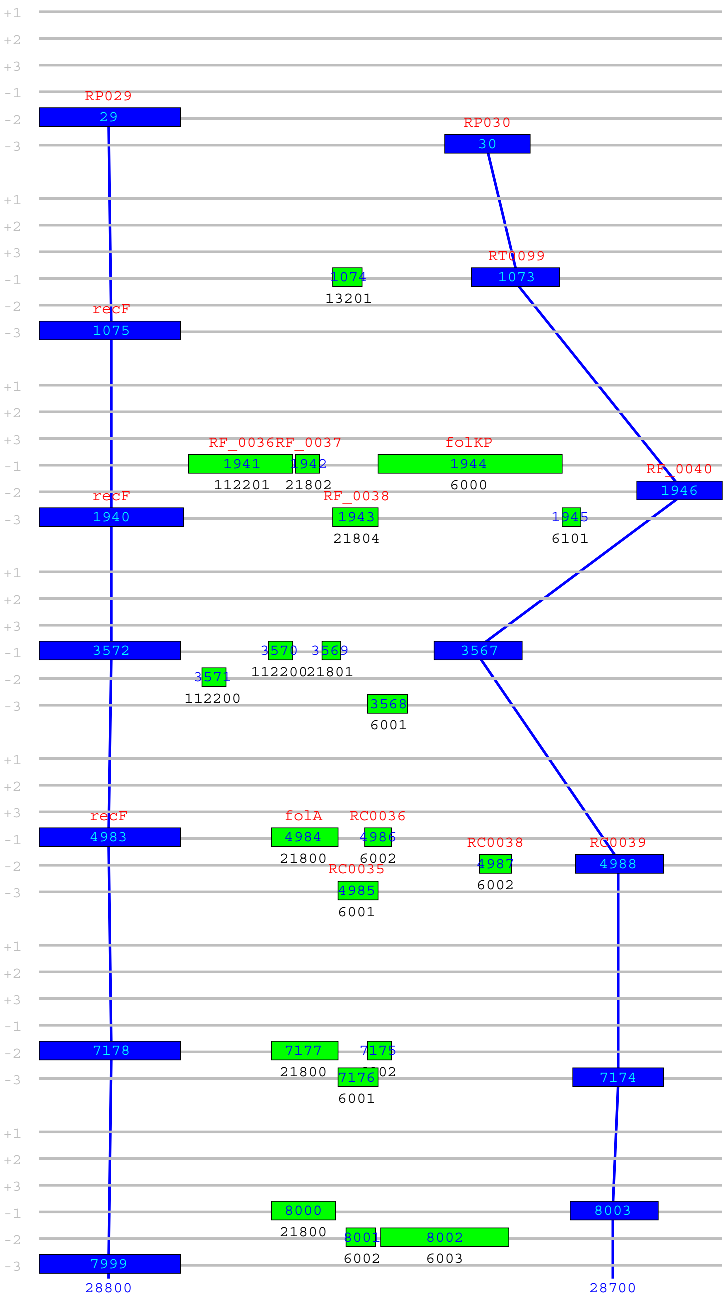

1 Rickettsia prowazekii str. Madrid E, complete genome
2 Rickettsia typhi str. wilmington, complete genome
3 Rickettsia felis URRWXCal2, complete genome
4 Rickettsia akari str. Hartford chromosome, whole genome shotgun sequence
5 Rickettsia conorii str. Malish 7, complete genome
6 Rickettsia sibirica 246 rsib_agncrt, whole genome shotgun sequence
7 Rickettsia rickettsii chromosome, whole genome shotgun sequence

Reg_id: 301

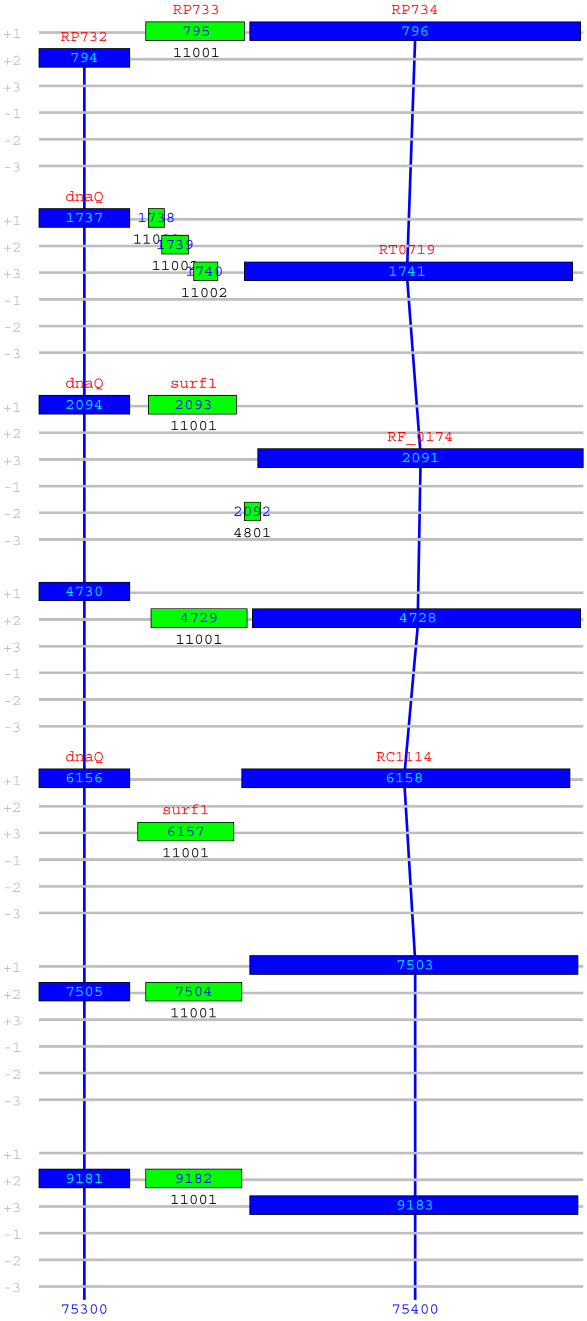

1 Rickettsia prowazekii str. Madrid E, complete genome
2 Rickettsia typhi str. wilmington, complete genome
3 Rickettsia felis URRWXCal2, complete genome
4 Rickettsia akari str. Hartford chromosome, whole genome shotgun sequence
5 Rickettsia conorii str. Malish 7, complete genome
6 Rickettsia sibirica 246 rsib_agncrt, whole genome shotgun sequence
7 Rickettsia rickettsii chromosome, whole genome shotgun sequence

Reg_id: 302

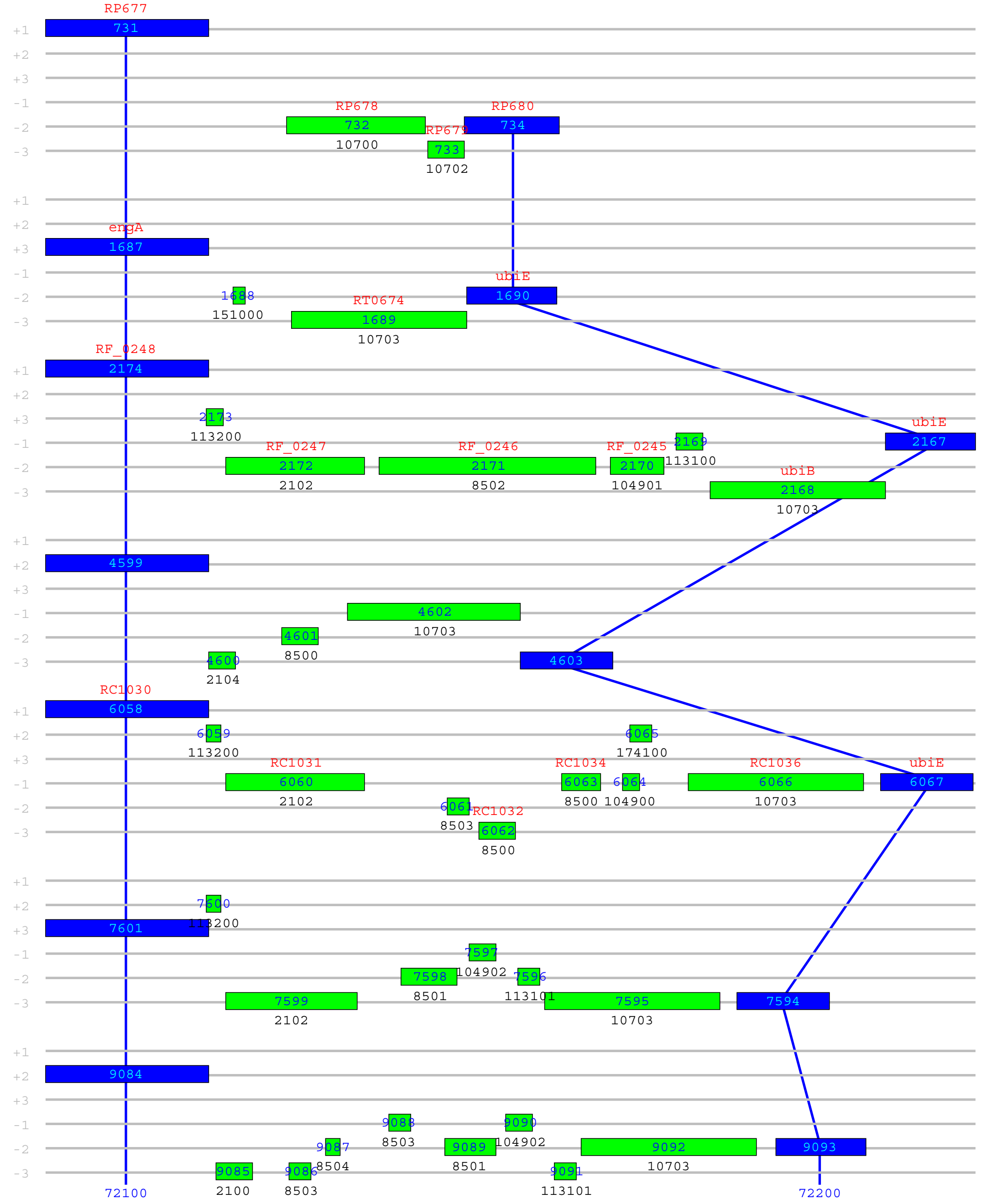

1 Rickettsia prowazekii str. Madrid E, complete genome
2 Rickettsia typhi str. wilmington, complete genome
3 Rickettsia felis URRWXCal2, complete genome
4 Rickettsia akari str. Hartford chromosome, whole genome shotgun sequence
5 Rickettsia conorii str. Malish 7, complete genome
6 Rickettsia sibirica 246 rsib_agncrt, whole genome shotgun sequence
7 Rickettsia rickettsii chromosome, whole genome shotgun sequence

Reg_id: 303

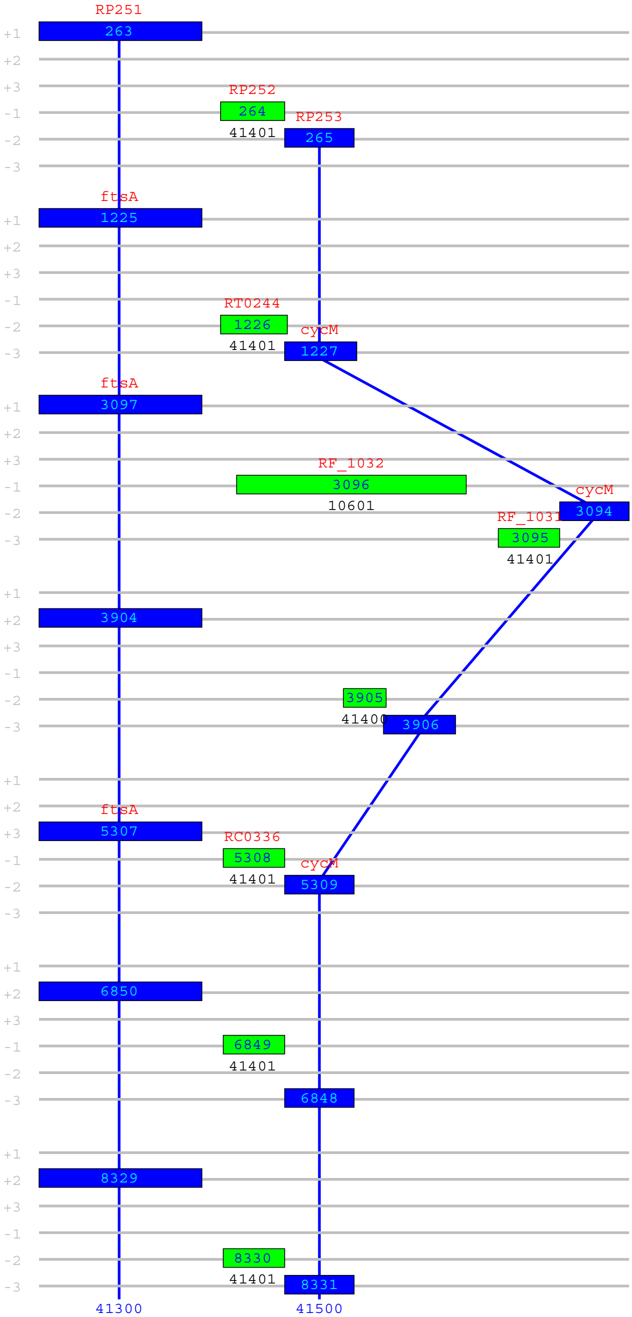

1 Rickettsia prowazekii str. Madrid E, complete genome
2 Rickettsia typhi str. wilmington, complete genome
3 Rickettsia felis URRWXCal2, complete genome
4 Rickettsia akari str. Hartford chromosome, whole genome shotgun sequence
5 Rickettsia conorii str. Malish 7, complete genome
6 Rickettsia sibirica 246 rsib_agncrt, whole genome shotgun sequence
7 Rickettsia rickettsii chromosome, whole genome shotgun sequence

Reg_id: 304

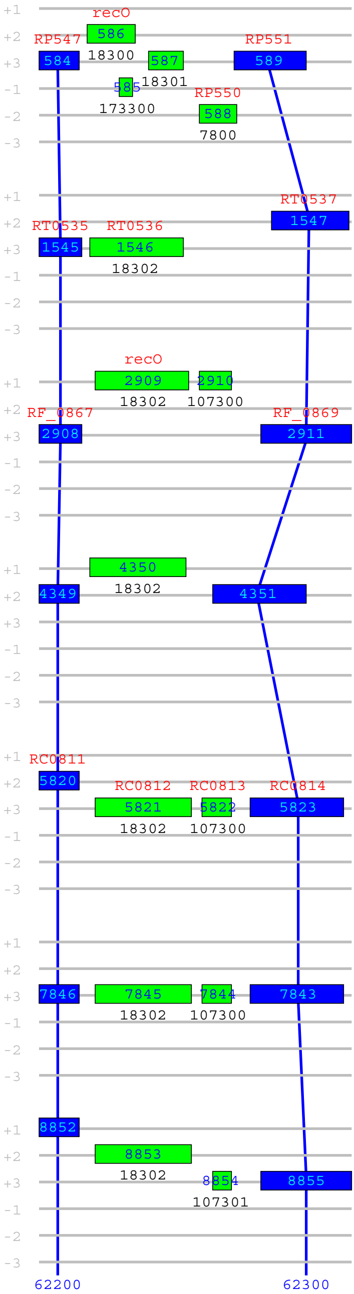

1 Rickettsia prowazekii str. Madrid E, complete genome
2 Rickettsia typhi str. wilmington, complete genome
3 Rickettsia felis URRWXCal2, complete genome
4 Rickettsia akari str. Hartford chromosome, whole genome shotgun sequence
5 Rickettsia conorii str. Malish 7, complete genome
6 Rickettsia sibirica 246 rsib_agncrt, whole genome shotgun sequence
7 Rickettsia rickettsii chromosome, whole genome shotgun sequence

Reg_id: 307

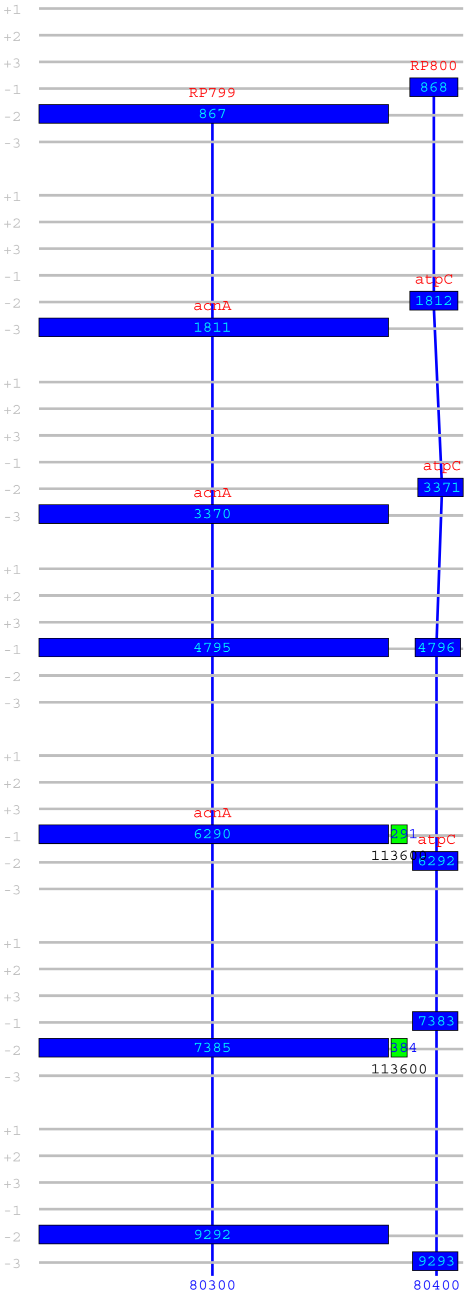

1 Rickettsia prowazekii str. Madrid E, complete genome
2 Rickettsia typhi str. wilmington, complete genome
3 Rickettsia felis URRWXCal2, complete genome
4 Rickettsia akari str. Hartford chromosome, whole genome shotgun sequence
5 Rickettsia conorii str. Malish 7, complete genome
6 Rickettsia sibirica 246 rsib_agncrt, whole genome shotgun sequence
7 Rickettsia rickettsii chromosome, whole genome shotgun sequence

Reg_id: 308

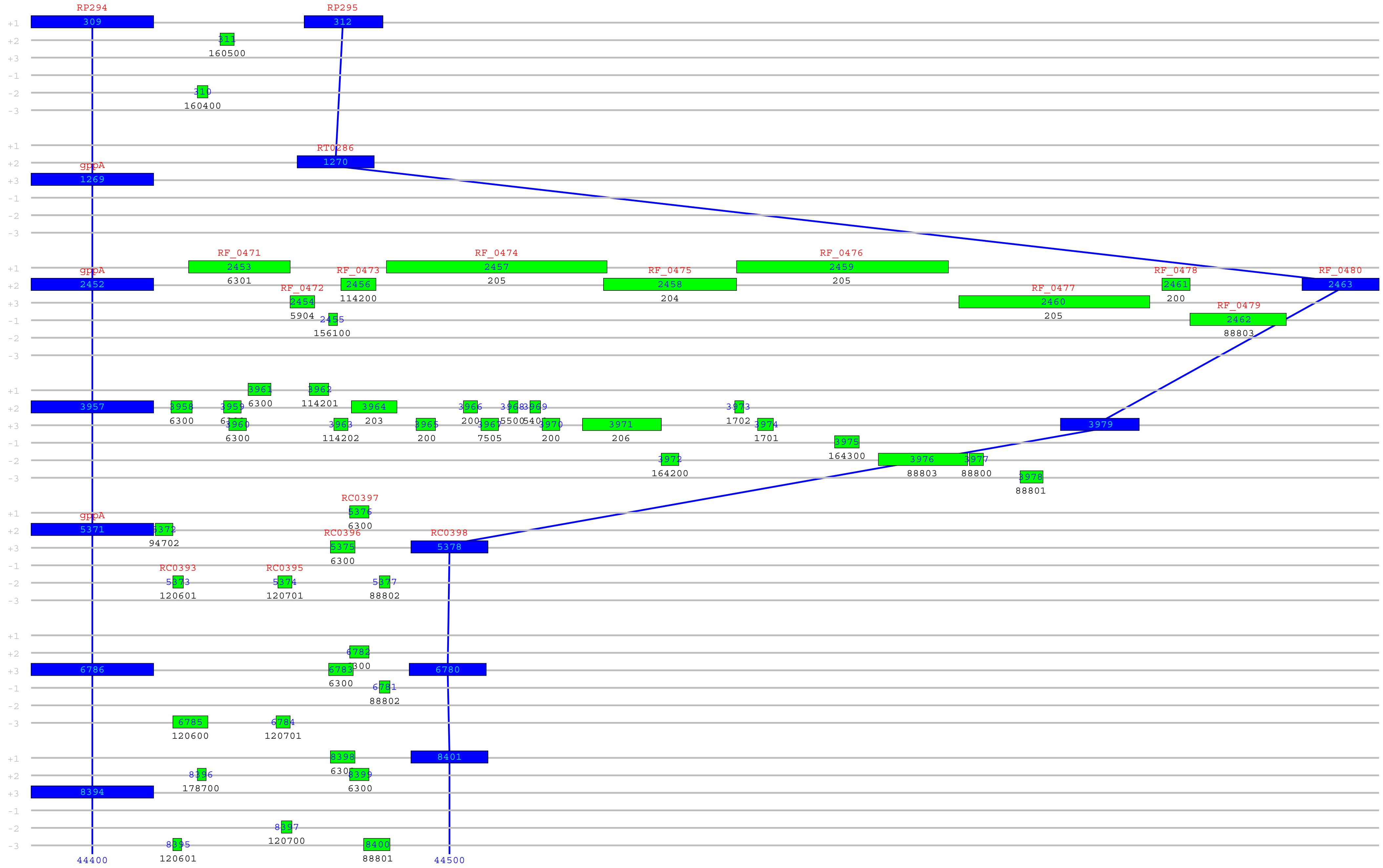

1 Rickettsia prowazekii str. Madrid E, complete genome
2 Rickettsia typhi str. wilmington, complete genome
3 Rickettsia felis URRWXCal2, complete genome
4 Rickettsia akari str. Hartford chromosome, whole genome shotgun sequence
5 Rickettsia conorii str. Malish 7, complete genome
6 Rickettsia sibirica 246 rsib_agncrt, whole genome shotgun sequence
7 Rickettsia rickettsii chromosome, whole genome shotgun sequence

Reg_id: 310

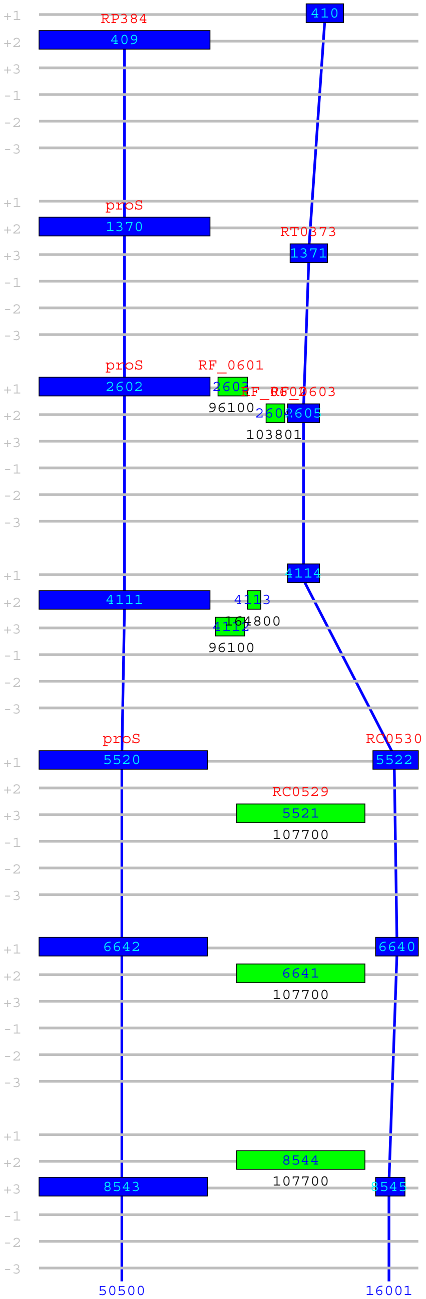

1 Rickettsia prowazekii str. Madrid E, complete genome
2 Rickettsia typhi str. wilmington, complete genome
3 Rickettsia felis URRWXCal2, complete genome
4 Rickettsia akari str. Hartford chromosome, whole genome shotgun sequence
5 Rickettsia conorii str. Malish 7, complete genome
6 Rickettsia sibirica 246 rsib_agncrt, whole genome shotgun sequence
7 Rickettsia rickettsii chromosome, whole genome shotgun sequence

Reg_id: 314

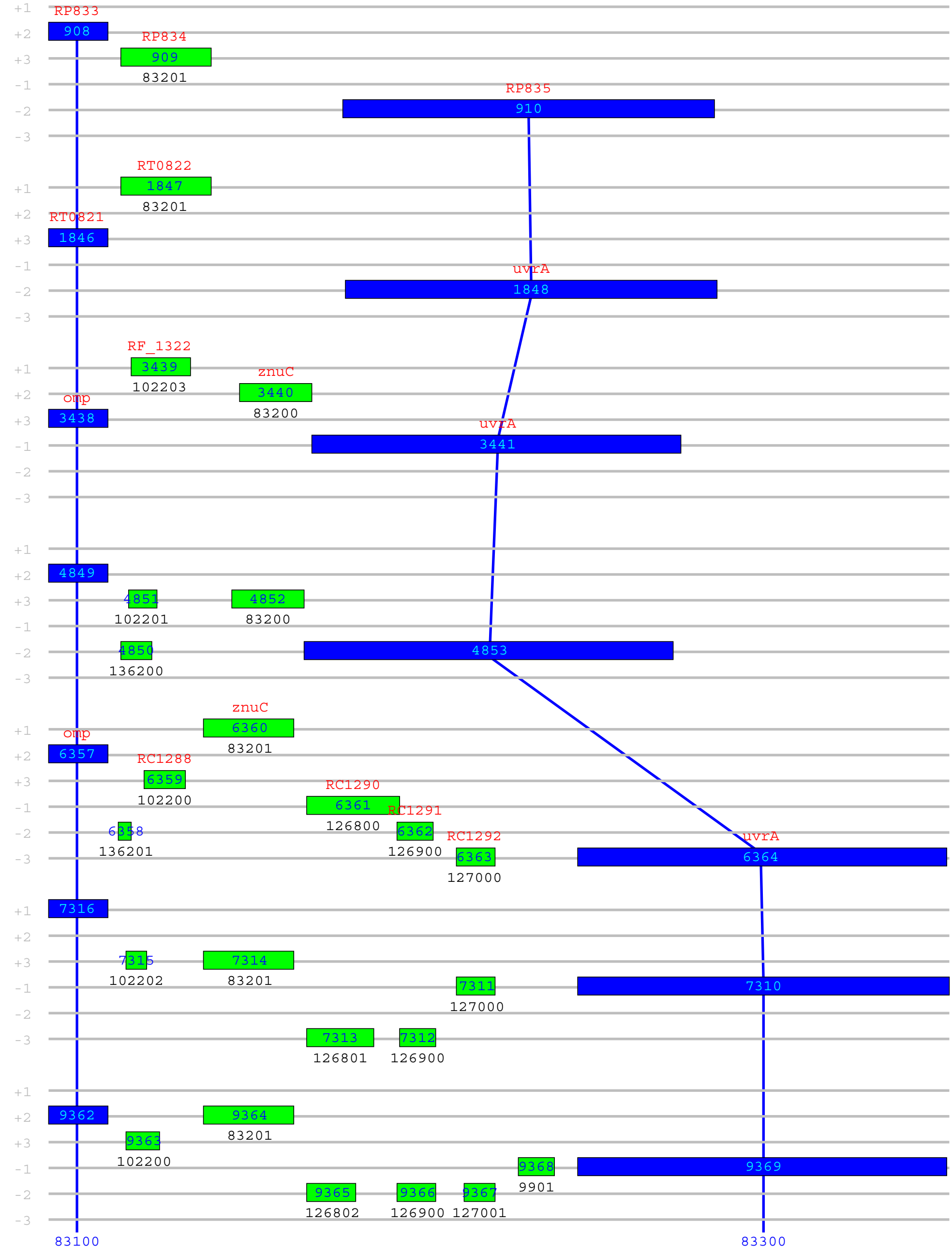

1 Rickettsia prowazekii str. Madrid E, complete genome
2 Rickettsia typhi str. wilmington, complete genome
3 Rickettsia felis URRWXCal2, complete genome
4 Rickettsia akari str. Hartford chromosome, whole genome shotgun sequence
5 Rickettsia conorii str. Malish 7, complete genome
6 Rickettsia sibirica 246 rsib_agncrt, whole genome shotgun sequence
7 Rickettsia rickettsii chromosome, whole genome shotgun sequence

Reg_id: 320

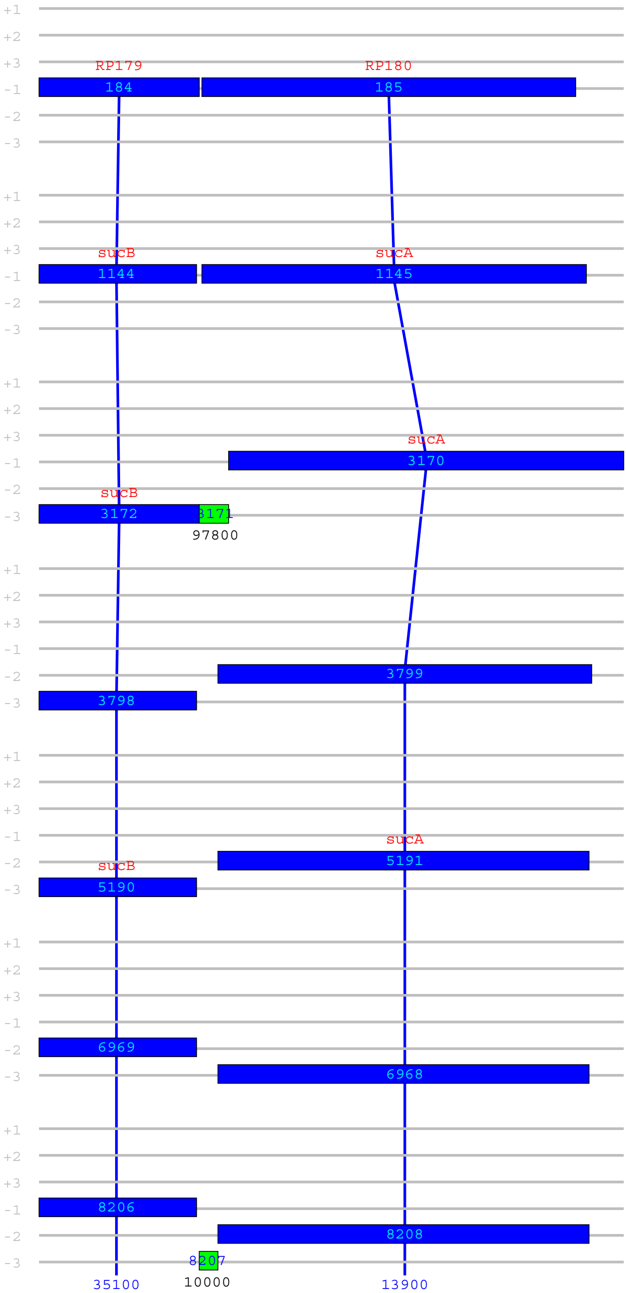

1 Rickettsia prowazekii str. Madrid E, complete genome
2 Rickettsia typhi str. wilmington, complete genome
3 Rickettsia felis URRWXCal2, complete genome
4 Rickettsia akari str. Hartford chromosome, whole genome shotgun sequence
5 Rickettsia conorii str. Malish 7, complete genome
6 Rickettsia sibirica 246 rsib_agncrt, whole genome shotgun sequence
7 Rickettsia rickettsii chromosome, whole genome shotgun sequence

Reg_id: 322

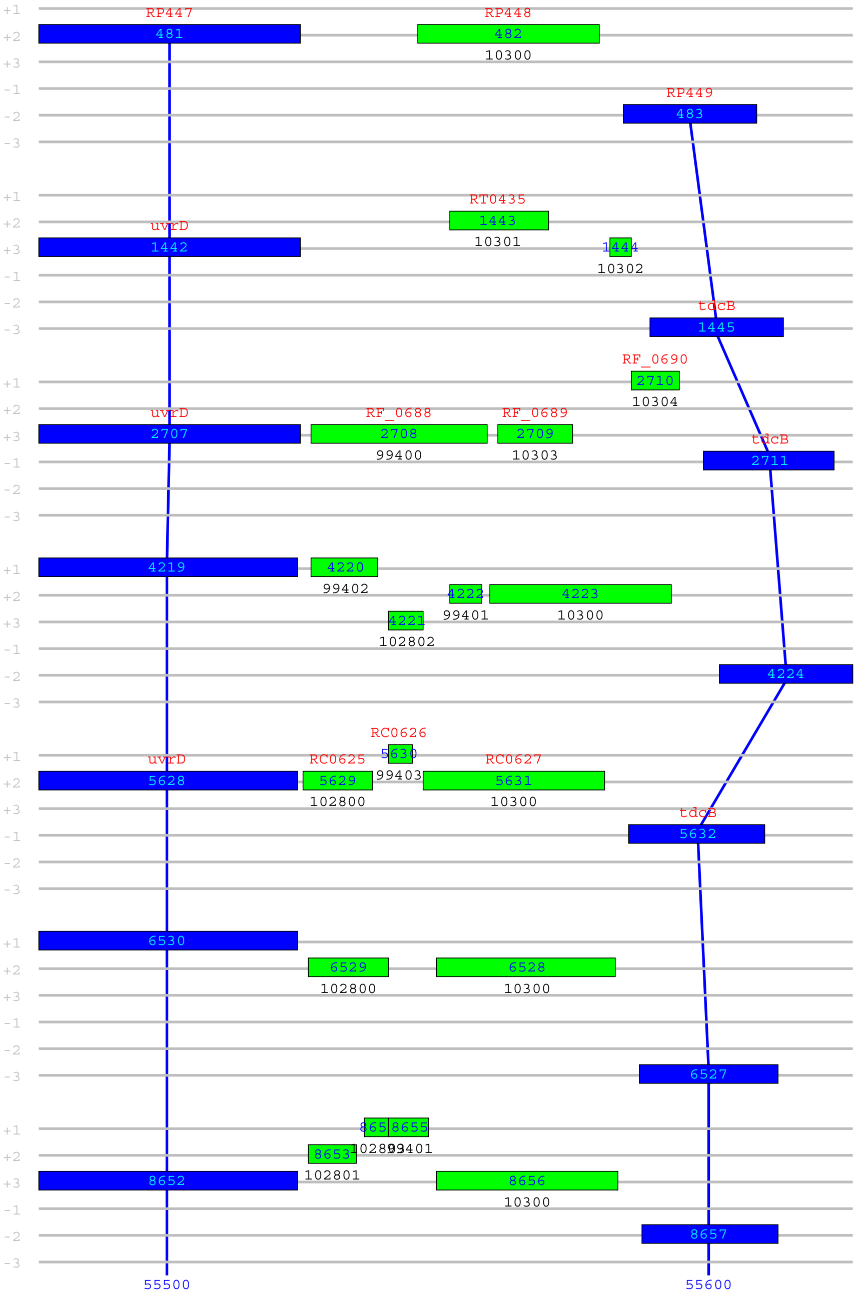

1 Rickettsia prowazekii str. Madrid E, complete genome
2 Rickettsia typhi str. wilmington, complete genome
3 Rickettsia felis URRWXCal2, complete genome
4 Rickettsia akari str. Hartford chromosome, whole genome shotgun sequence
5 Rickettsia conorii str. Malish 7, complete genome
6 Rickettsia sibirica 246 rsib_agncrt, whole genome shotgun sequence
7 Rickettsia rickettsii chromosome, whole genome shotgun sequence

Reg_id: 323

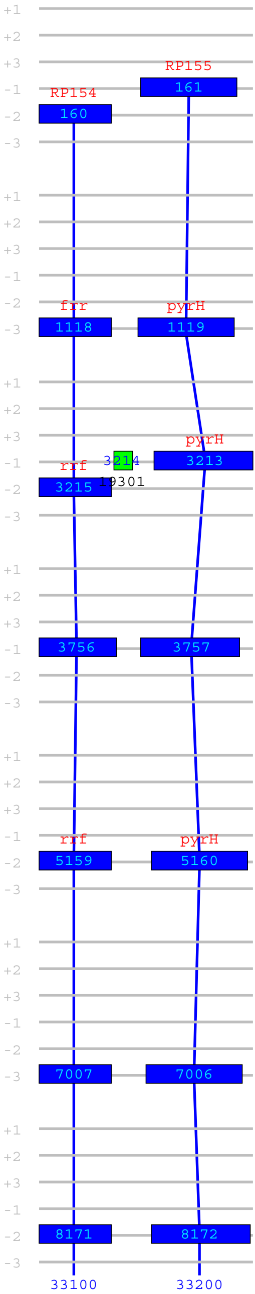

1 Rickettsia prowazekii str. Madrid E, complete genome
2 Rickettsia typhi str. wilmington, complete genome
3 Rickettsia felis URRWXCal2, complete genome
4 Rickettsia akari str. Hartford chromosome, whole genome shotgun sequence
5 Rickettsia conorii str. Malish 7, complete genome
6 Rickettsia sibirica 246 rsib_agncrt, whole genome shotgun sequence
7 Rickettsia rickettsii chromosome, whole genome shotgun sequence

Reg_id: 324

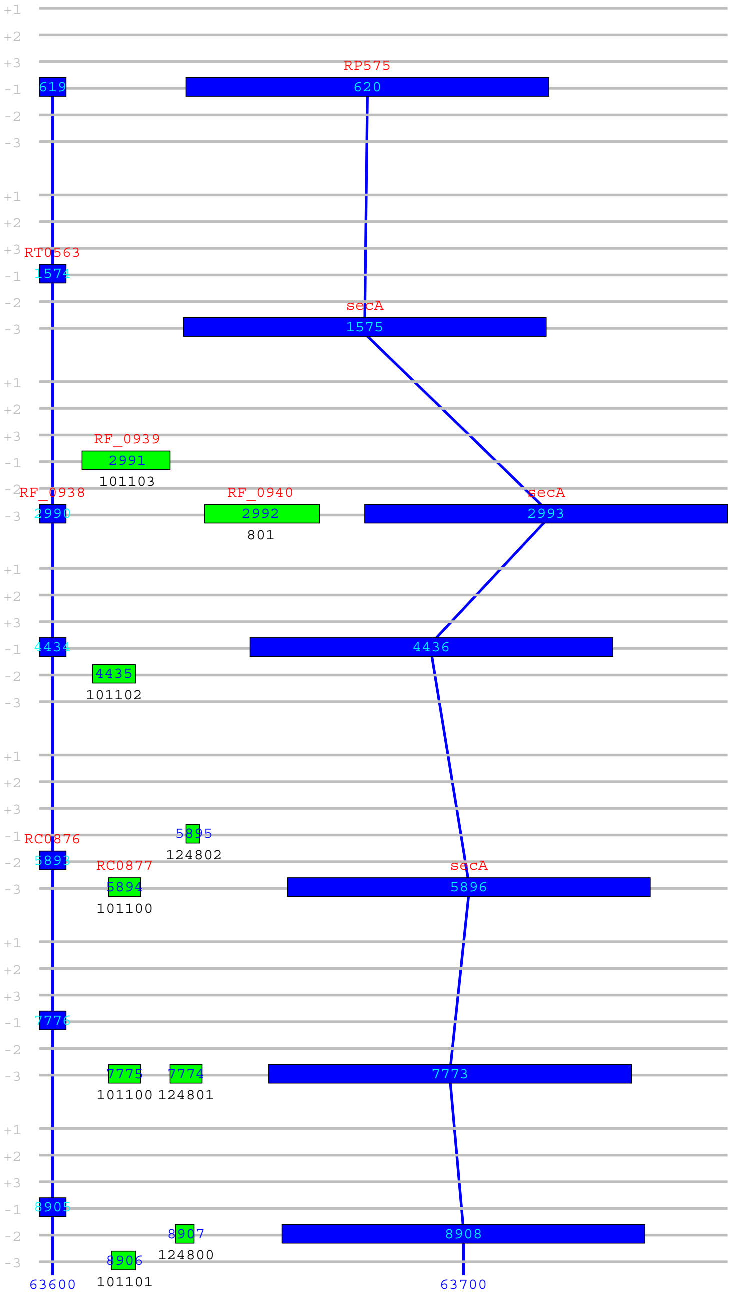

1 Rickettsia prowazekii str. Madrid E, complete genome
2 Rickettsia typhi str. wilmington, complete genome
3 Rickettsia felis URRWXCal2, complete genome
4 Rickettsia akari str. Hartford chromosome, whole genome shotgun sequence
5 Rickettsia conorii str. Malish 7, complete genome
6 Rickettsia sibirica 246 rsib_agncrt, whole genome shotgun sequence
7 Rickettsia rickettsii chromosome, whole genome shotgun sequence

Reg_id: 328

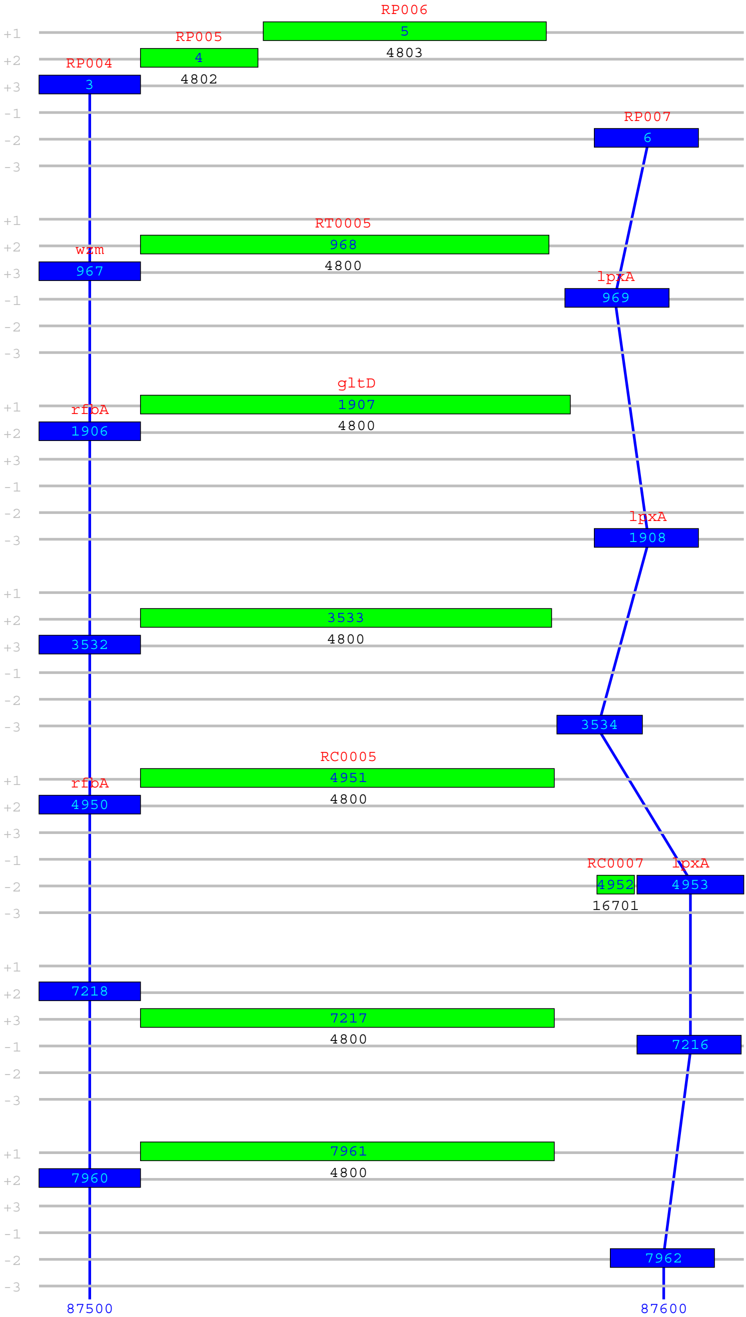

1 Rickettsia prowazekii str. Madrid E, complete genome
2 Rickettsia typhi str. wilmington, complete genome
3 Rickettsia felis URRWXCal2, complete genome
4 Rickettsia akari str. Hartford chromosome, whole genome shotgun sequence
5 Rickettsia conorii str. Malish 7, complete genome
6 Rickettsia sibirica 246 rsib_agncrt, whole genome shotgun sequence
7 Rickettsia rickettsii chromosome, whole genome shotgun sequence

Reg_id: 331

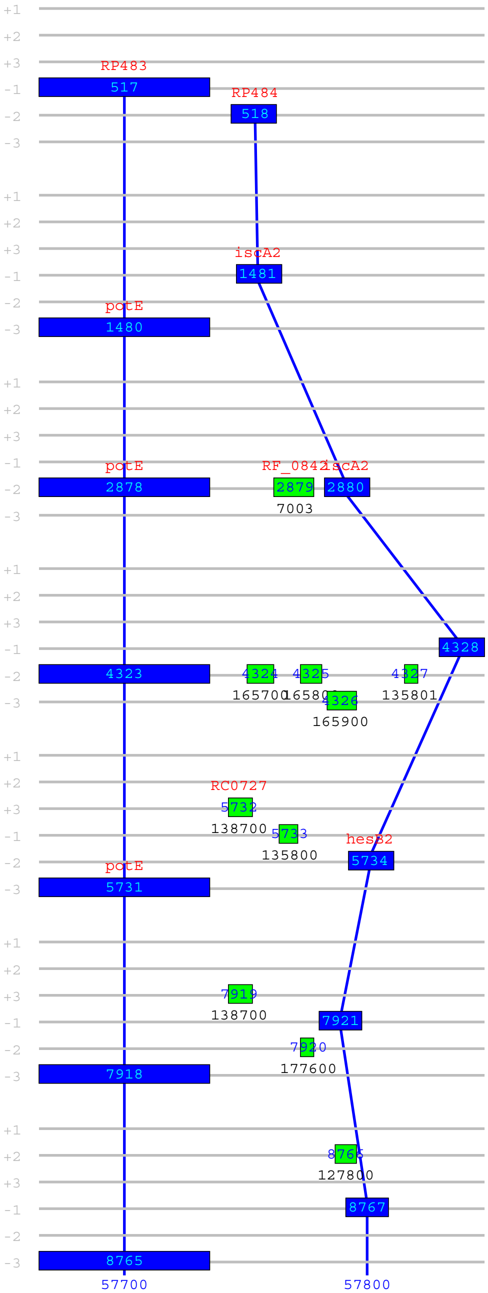

1 Rickettsia prowazekii str. Madrid E, complete genome
2 Rickettsia typhi str. wilmington, complete genome
3 Rickettsia felis URRWXCal2, complete genome
4 Rickettsia akari str. Hartford chromosome, whole genome shotgun sequence
5 Rickettsia conorii str. Malish 7, complete genome
6 Rickettsia sibirica 246 rsib_agncrt, whole genome shotgun sequence
7 Rickettsia rickettsii chromosome, whole genome shotgun sequence

Reg_id: 334

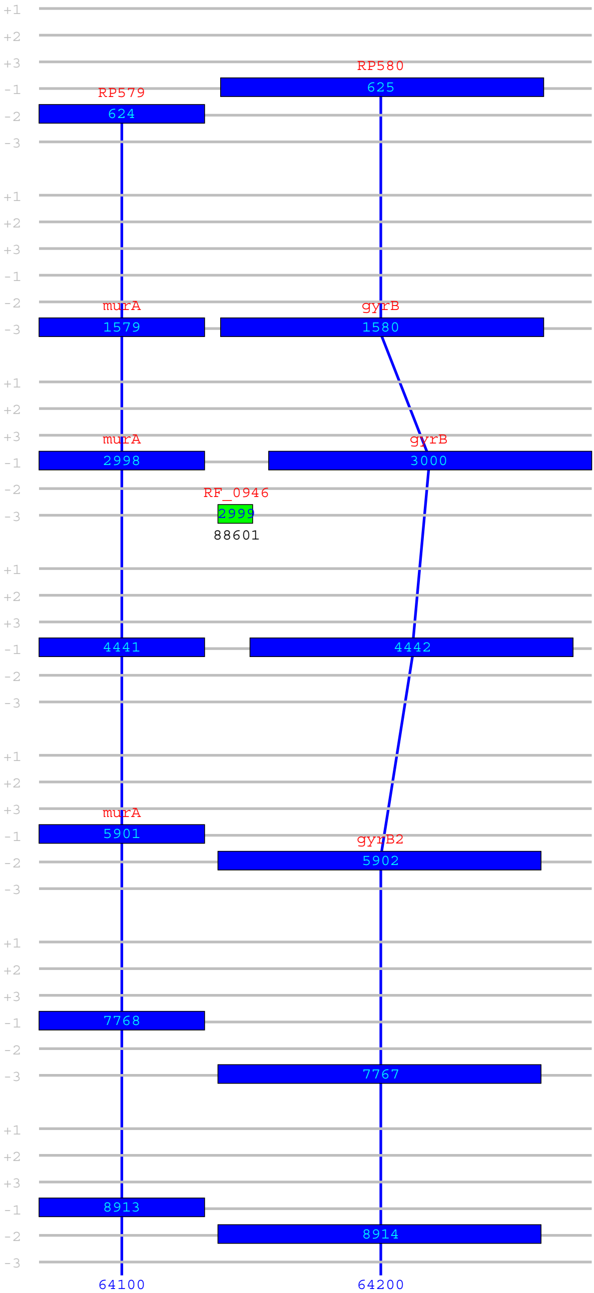

1 Rickettsia prowazekii str. Madrid E, complete genome
2 Rickettsia typhi str. wilmington, complete genome
3 Rickettsia felis URRWXCal2, complete genome
4 Rickettsia akari str. Hartford chromosome, whole genome shotgun sequence
5 Rickettsia conorii str. Malish 7, complete genome
6 Rickettsia sibirica 246 rsib_agncrt, whole genome shotgun sequence
7 Rickettsia rickettsii chromosome, whole genome shotgun sequence

Reg_id: 337

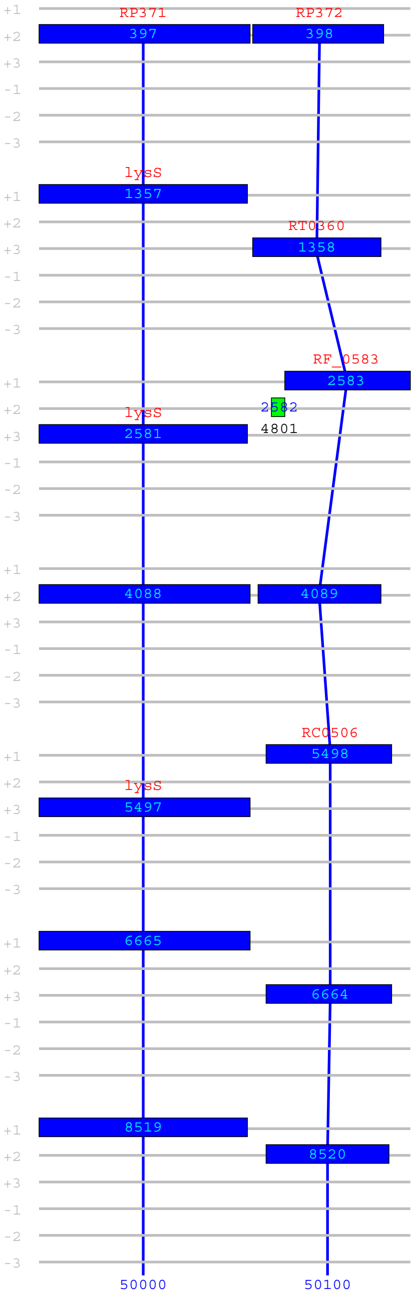

1 Rickettsia prowazekii str. Madrid E, complete genome
2 Rickettsia typhi str. wilmington, complete genome
3 Rickettsia felis URRWXCal2, complete genome
4 Rickettsia akari str. Hartford chromosome, whole genome shotgun sequence
5 Rickettsia conorii str. Malish 7, complete genome
6 Rickettsia sibirica 246 rsib_agncrt, whole genome shotgun sequence
7 Rickettsia rickettsii chromosome, whole genome shotgun sequence

Reg_id: 338

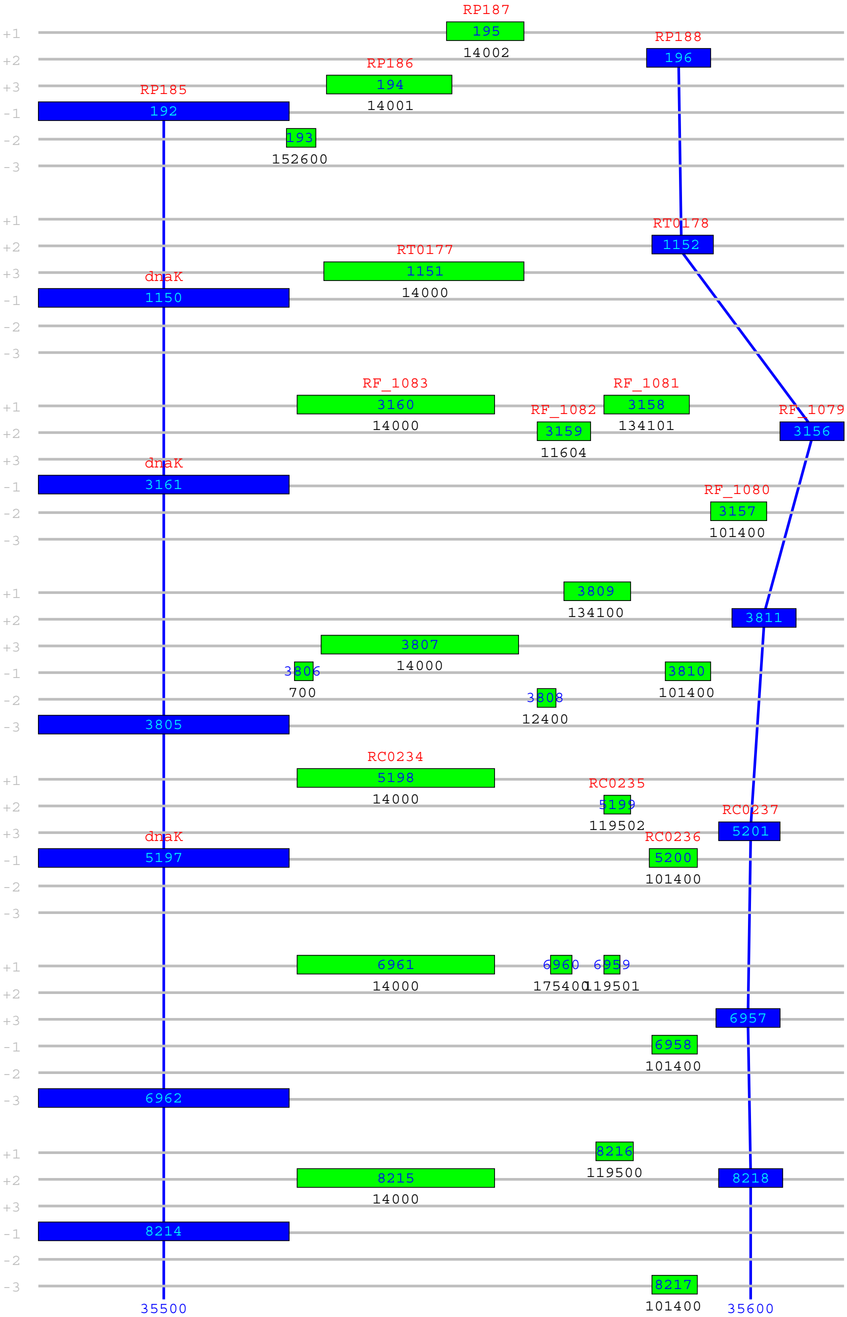

1 Rickettsia prowazekii str. Madrid E, complete genome
2 Rickettsia typhi str. wilmington, complete genome
3 Rickettsia felis URRWXCal2, complete genome
4 Rickettsia akari str. Hartford chromosome, whole genome shotgun sequence
5 Rickettsia conorii str. Malish 7, complete genome
6 Rickettsia sibirica 246 rsib_agncrt, whole genome shotgun sequence
7 Rickettsia rickettsii chromosome, whole genome shotgun sequence

Reg_id: 339

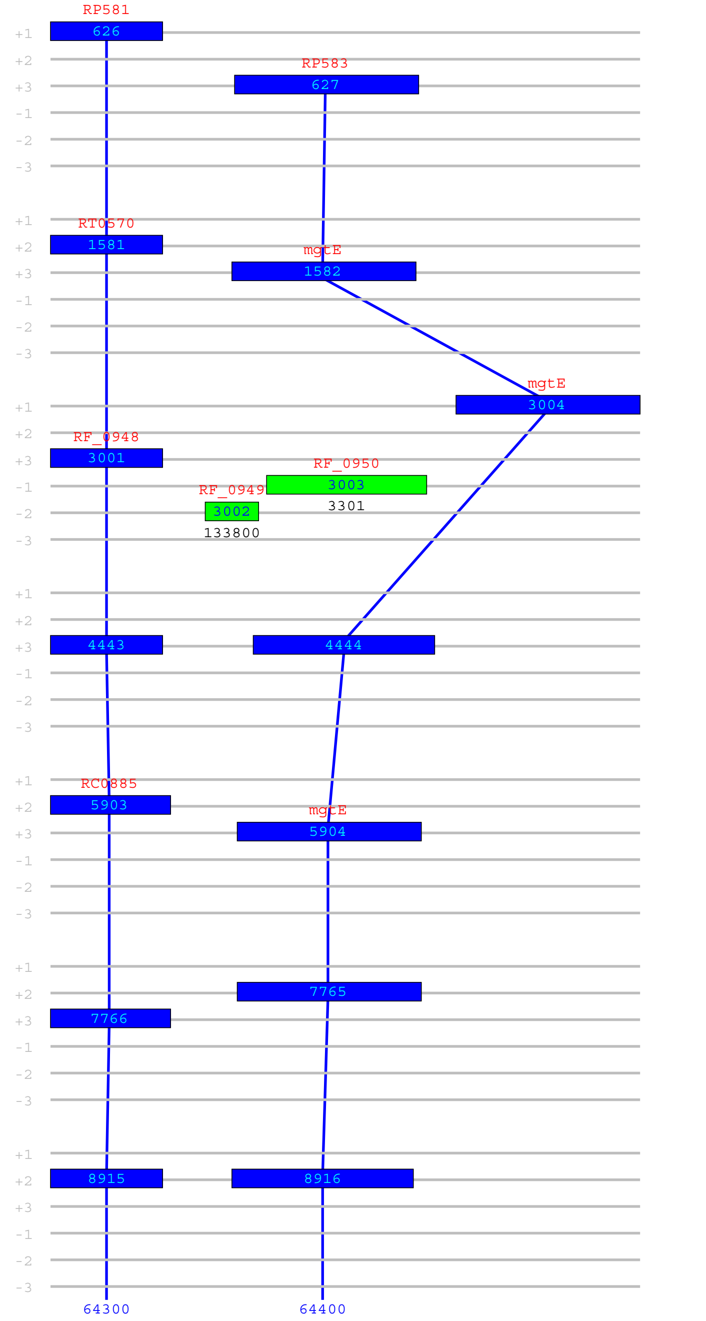

1 Rickettsia prowazekii str. Madrid E, complete genome
2 Rickettsia typhi str. wilmington, complete genome
3 Rickettsia felis URRWXCal2, complete genome
4 Rickettsia akari str. Hartford chromosome, whole genome shotgun sequence
5 Rickettsia conorii str. Malish 7, complete genome
6 Rickettsia sibirica 246 rsib_agncrt, whole genome shotgun sequence
7 Rickettsia rickettsii chromosome, whole genome shotgun sequence

Reg_id: 340

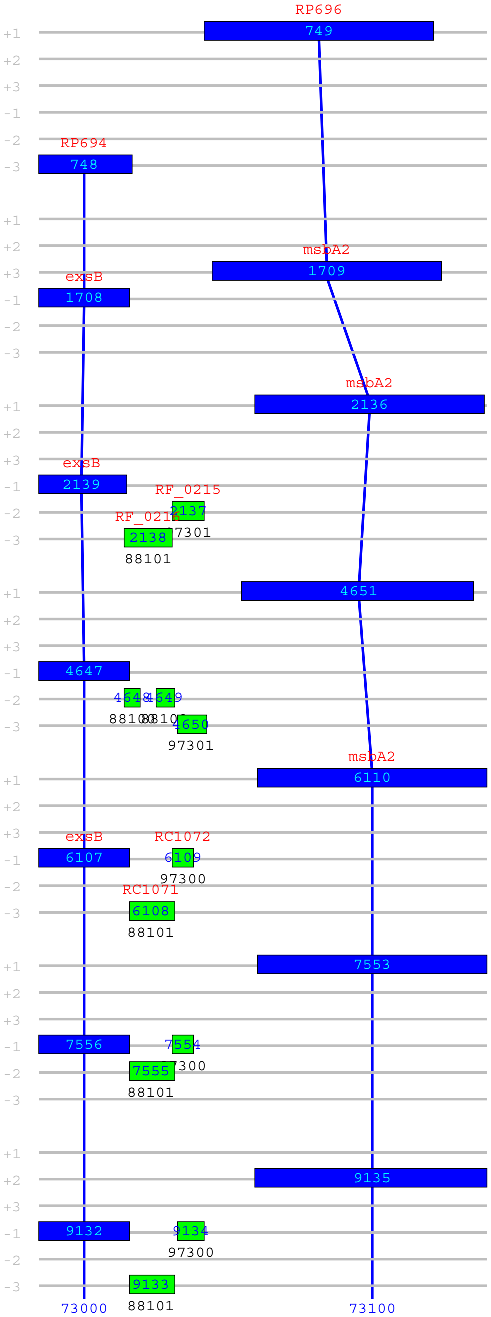

1 Rickettsia prowazekii str. Madrid E, complete genome
2 Rickettsia typhi str. wilmington, complete genome
3 Rickettsia felis URRWXCal2, complete genome
4 Rickettsia akari str. Hartford chromosome, whole genome shotgun sequence
5 Rickettsia conorii str. Malish 7, complete genome
6 Rickettsia sibirica 246 rsib_agncrt, whole genome shotgun sequence
7 Rickettsia rickettsii chromosome, whole genome shotgun sequence

Reg_id: 341

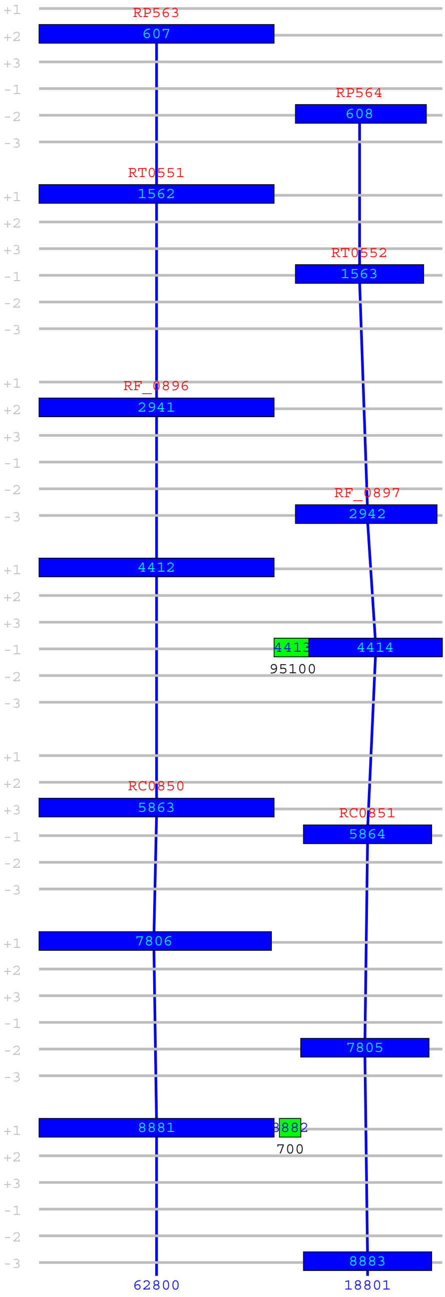

1 Rickettsia prowazekii str. Madrid E, complete genome
2 Rickettsia typhi str. wilmington, complete genome
3 Rickettsia felis URRWXCal2, complete genome
4 Rickettsia akari str. Hartford chromosome, whole genome shotgun sequence
5 Rickettsia conorii str. Malish 7, complete genome
6 Rickettsia sibirica 246 rsib_agncrt, whole genome shotgun sequence
7 Rickettsia rickettsii chromosome, whole genome shotgun sequence

Reg_id: 343

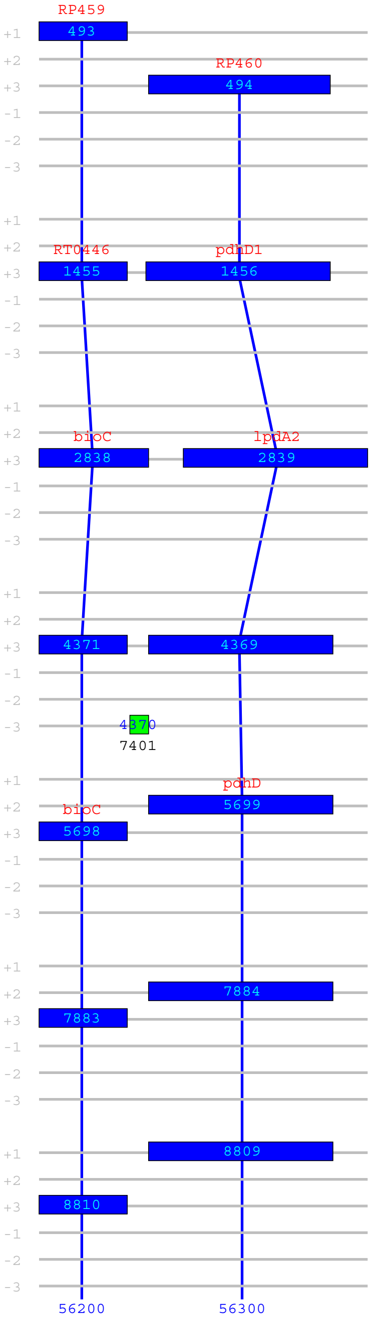

1 Rickettsia prowazekii str. Madrid E, complete genome
2 Rickettsia typhi str. wilmington, complete genome
3 Rickettsia felis URRWXCal2, complete genome
4 Rickettsia akari str. Hartford chromosome, whole genome shotgun sequence
5 Rickettsia conorii str. Malish 7, complete genome
6 Rickettsia sibirica 246 rsib_agncrt, whole genome shotgun sequence
7 Rickettsia rickettsii chromosome, whole genome shotgun sequence

Reg_id: 346

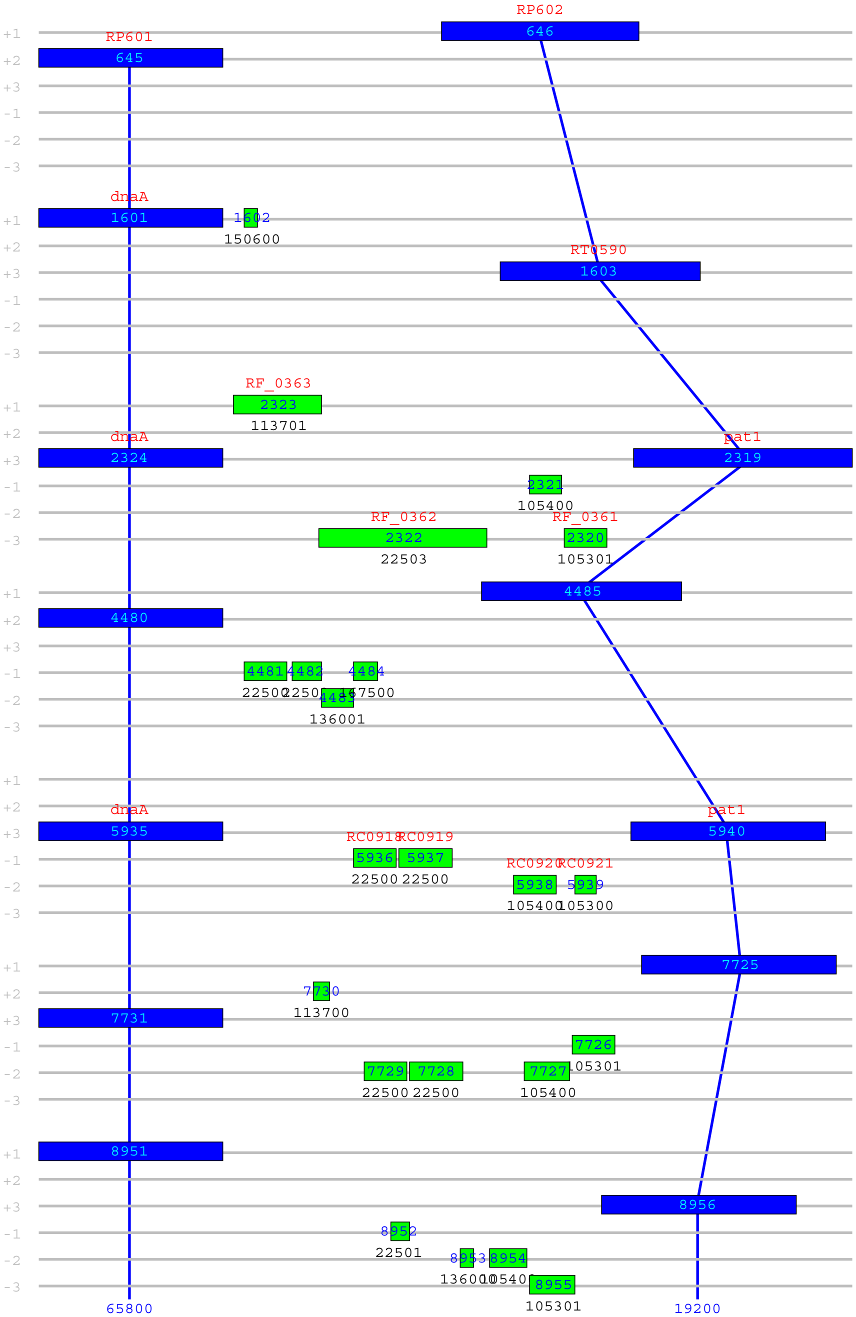

1 Rickettsia prowazekii str. Madrid E, complete genome
2 Rickettsia typhi str. wilmington, complete genome
3 Rickettsia felis URRWXCal2, complete genome
4 Rickettsia akari str. Hartford chromosome, whole genome shotgun sequence
5 Rickettsia conorii str. Malish 7, complete genome
6 Rickettsia sibirica 246 rsib_agncrt, whole genome shotgun sequence
7 Rickettsia rickettsii chromosome, whole genome shotgun sequence

Reg_id: 351

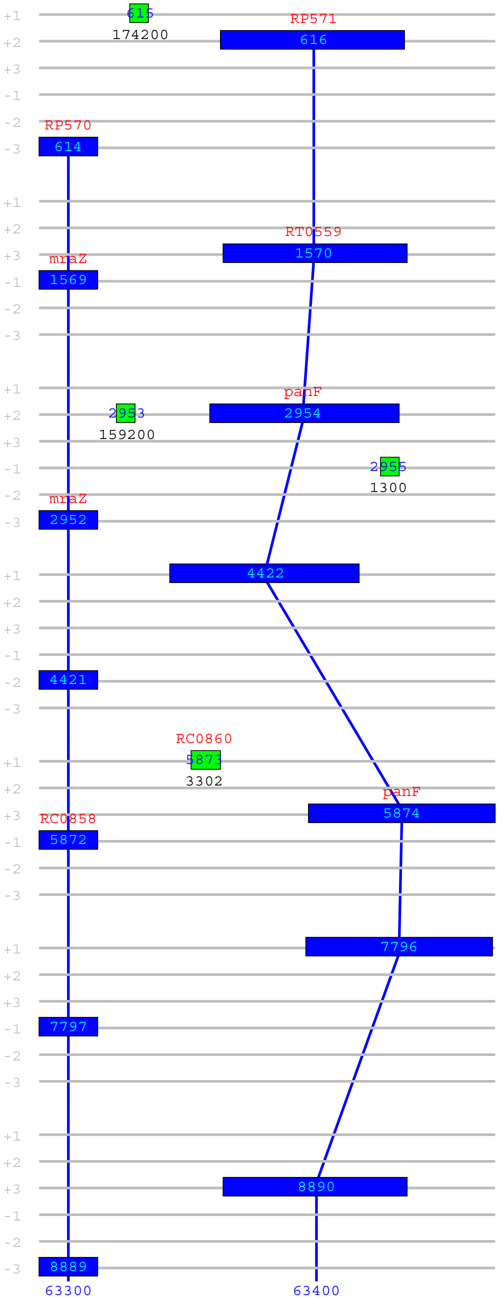

1 Rickettsia prowazekii str. Madrid E, complete genome
2 Rickettsia typhi str. wilmington, complete genome
3 Rickettsia felis URRWXCal2, complete genome
4 Rickettsia akari str. Hartford chromosome, whole genome shotgun sequence
5 Rickettsia conorii str. Malish 7, complete genome
6 Rickettsia sibirica 246 rsib_agncrt, whole genome shotgun sequence
7 Rickettsia rickettsii chromosome, whole genome shotgun sequence

Reg_id: 357

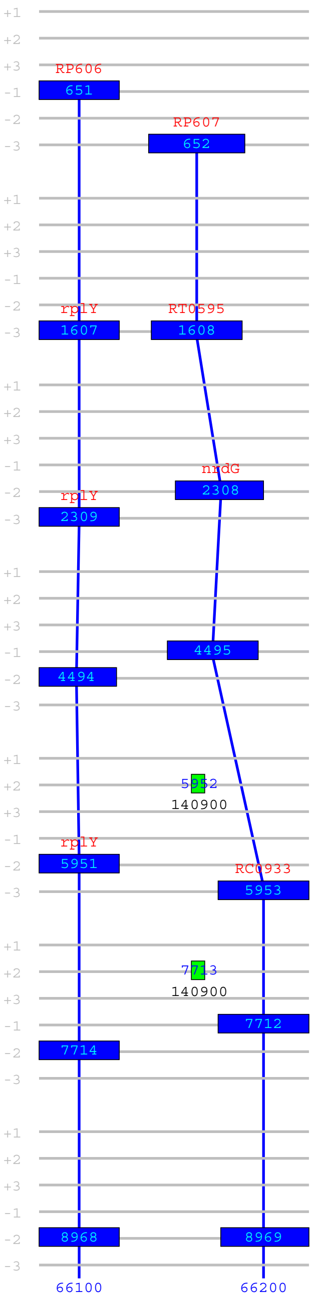

1 Rickettsia prowazekii str. Madrid E, complete genome
2 Rickettsia typhi str. wilmington, complete genome
3 Rickettsia felis URRWXCal2, complete genome
4 Rickettsia akari str. Hartford chromosome, whole genome shotgun sequence
5 Rickettsia conorii str. Malish 7, complete genome
6 Rickettsia sibirica 246 rsib_agncrt, whole genome shotgun sequence
7 Rickettsia rickettsii chromosome, whole genome shotgun sequence

Reg_id: 358

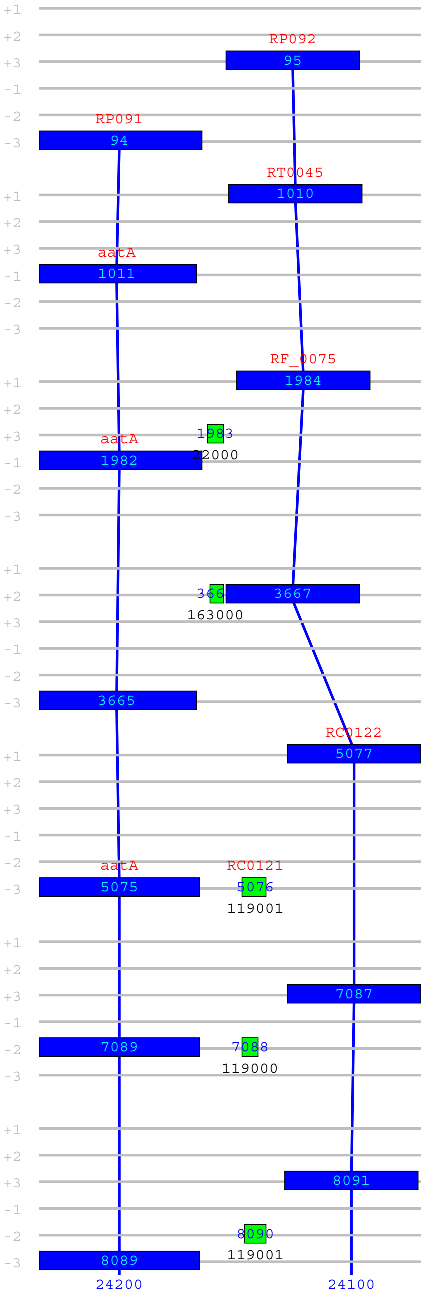

1 Rickettsia prowazekii str. Madrid E, complete genome
2 Rickettsia typhi str. wilmington, complete genome
3 Rickettsia felis URRWXCal2, complete genome
4 Rickettsia akari str. Hartford chromosome, whole genome shotgun sequence
5 Rickettsia conorii str. Malish 7, complete genome
6 Rickettsia sibirica 246 rsib_agncrt, whole genome shotgun sequence
7 Rickettsia rickettsii chromosome, whole genome shotgun sequence

Reg_id: 361

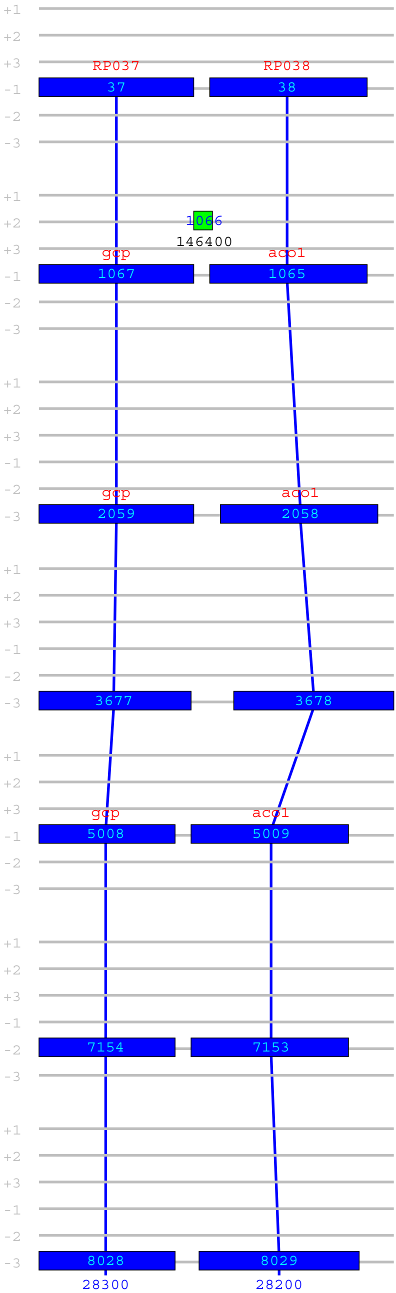

1 Rickettsia prowazekii str. Madrid E, complete genome
2 Rickettsia typhi str. wilmington, complete genome
3 Rickettsia felis URRWXCal2, complete genome
4 Rickettsia akari str. Hartford chromosome, whole genome shotgun sequence
5 Rickettsia conorii str. Malish 7, complete genome
6 Rickettsia sibirica 246 rsib_agncrt, whole genome shotgun sequence
7 Rickettsia rickettsii chromosome, whole genome shotgun sequence

Reg_id: 365

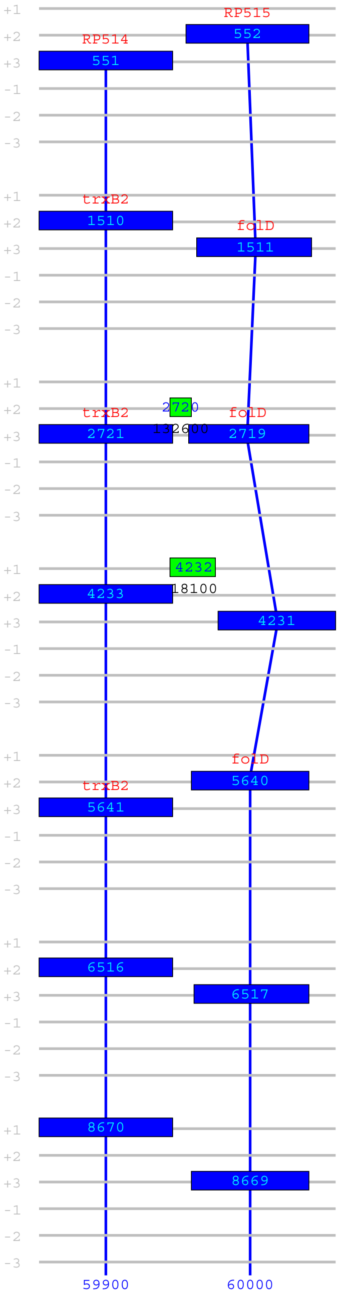

1 Rickettsia prowazekii str. Madrid E, complete genome
2 Rickettsia typhi str. wilmington, complete genome
3 Rickettsia felis URRWXCal2, complete genome
4 Rickettsia akari str. Hartford chromosome, whole genome shotgun sequence
5 Rickettsia conorii str. Malish 7, complete genome
6 Rickettsia sibirica 246 rsib_agncrt, whole genome shotgun sequence
7 Rickettsia rickettsii chromosome, whole genome shotgun sequence

Reg_id: 366

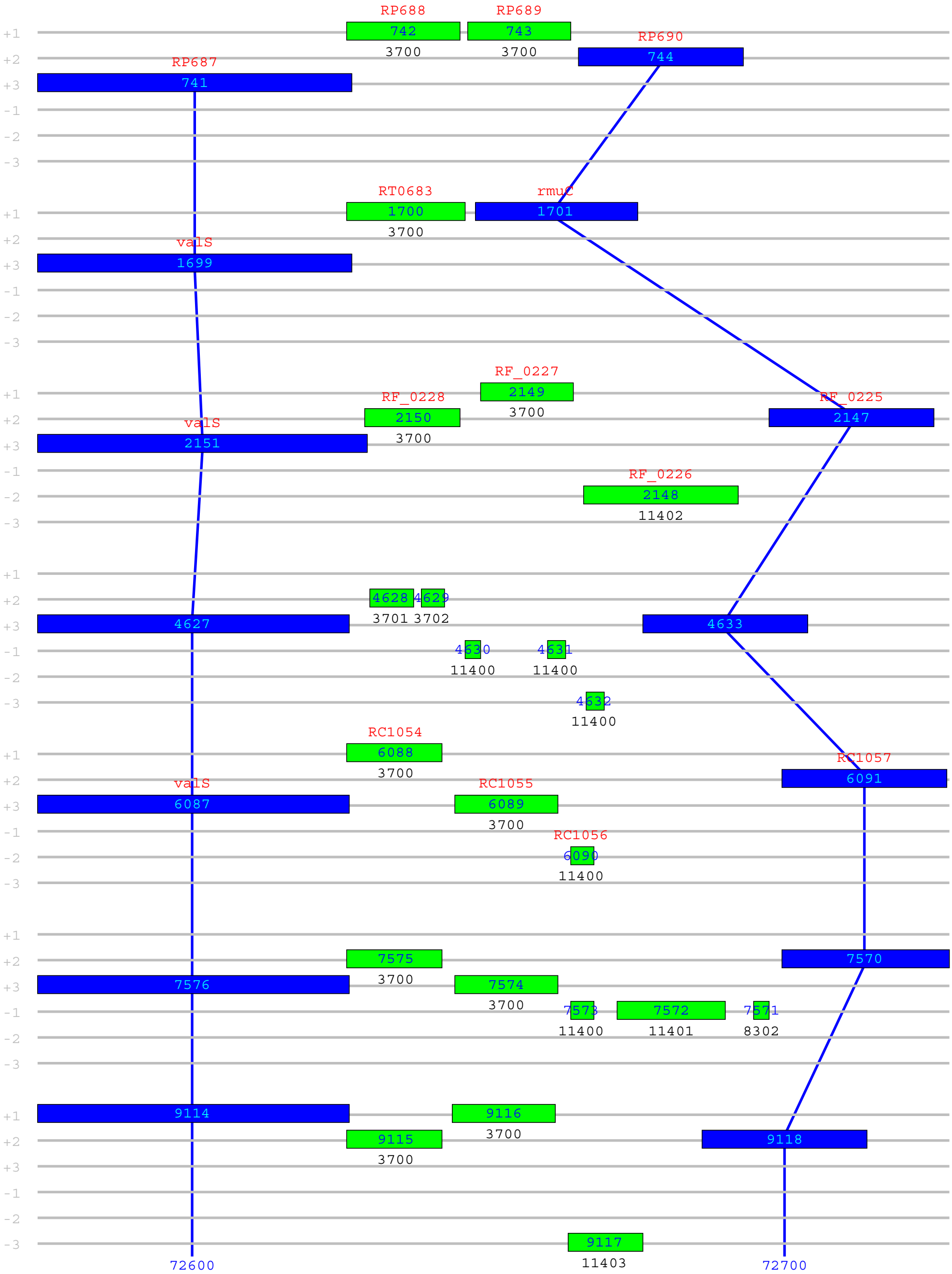

1 Rickettsia prowazekii str. Madrid E, complete genome
2 Rickettsia typhi str. wilmington, complete genome
3 Rickettsia felis URRWXCal2, complete genome
4 Rickettsia akari str. Hartford chromosome, whole genome shotgun sequence
5 Rickettsia conorii str. Malish 7, complete genome
6 Rickettsia sibirica 246 rsib_agncrt, whole genome shotgun sequence
7 Rickettsia rickettsii chromosome, whole genome shotgun sequence

Reg_id: 368

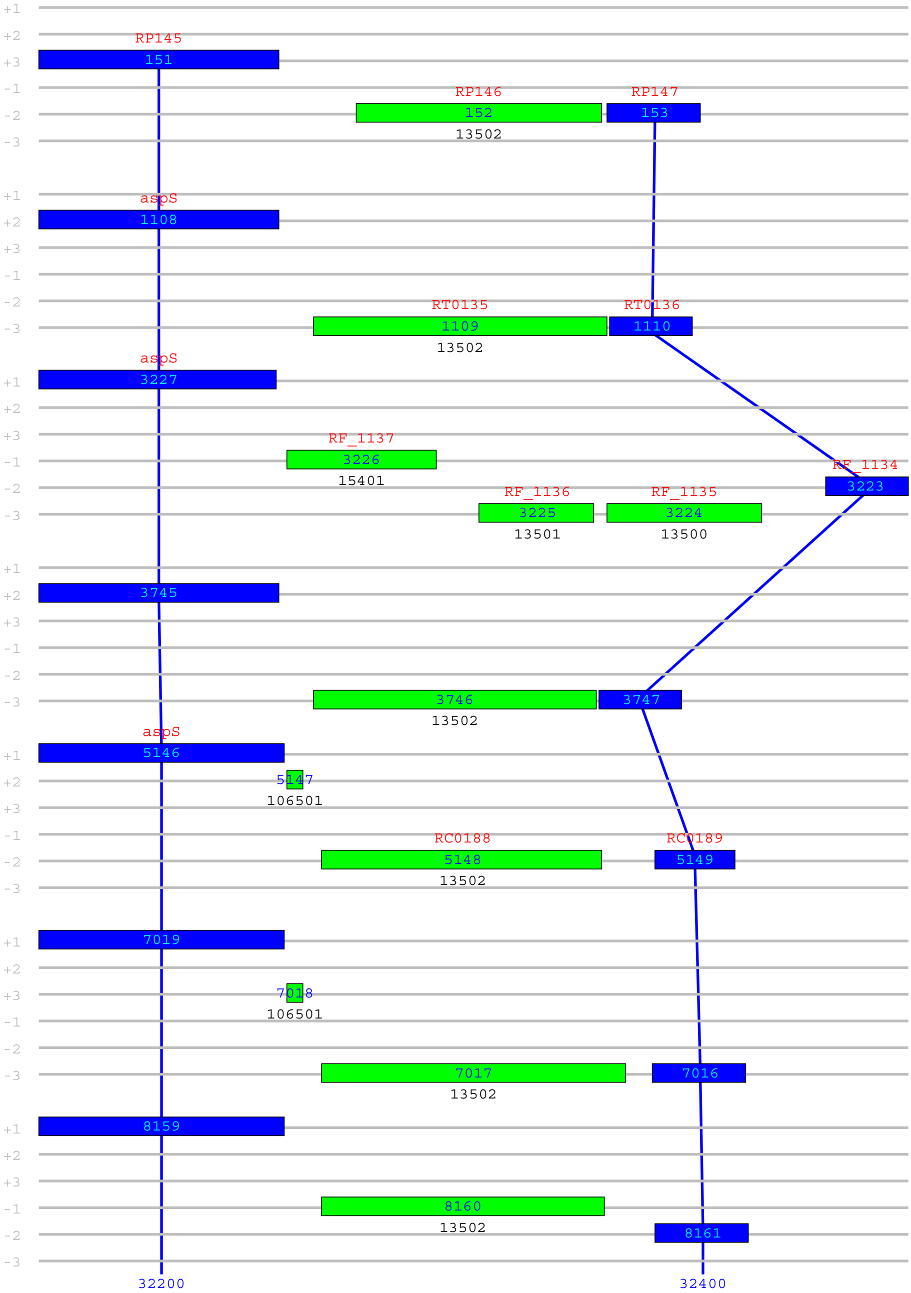

1 Rickettsia prowazekii str. Madrid E, complete genome
2 Rickettsia typhi str. wilmington, complete genome
3 Rickettsia felis URRWXCal2, complete genome
4 Rickettsia akari str. Hartford chromosome, whole genome shotgun sequence
5 Rickettsia conorii str. Malish 7, complete genome
6 Rickettsia sibirica 246 rsib_agncrt, whole genome shotgun sequence
7 Rickettsia rickettsii chromosome, whole genome shotgun sequence

Reg_id: 376

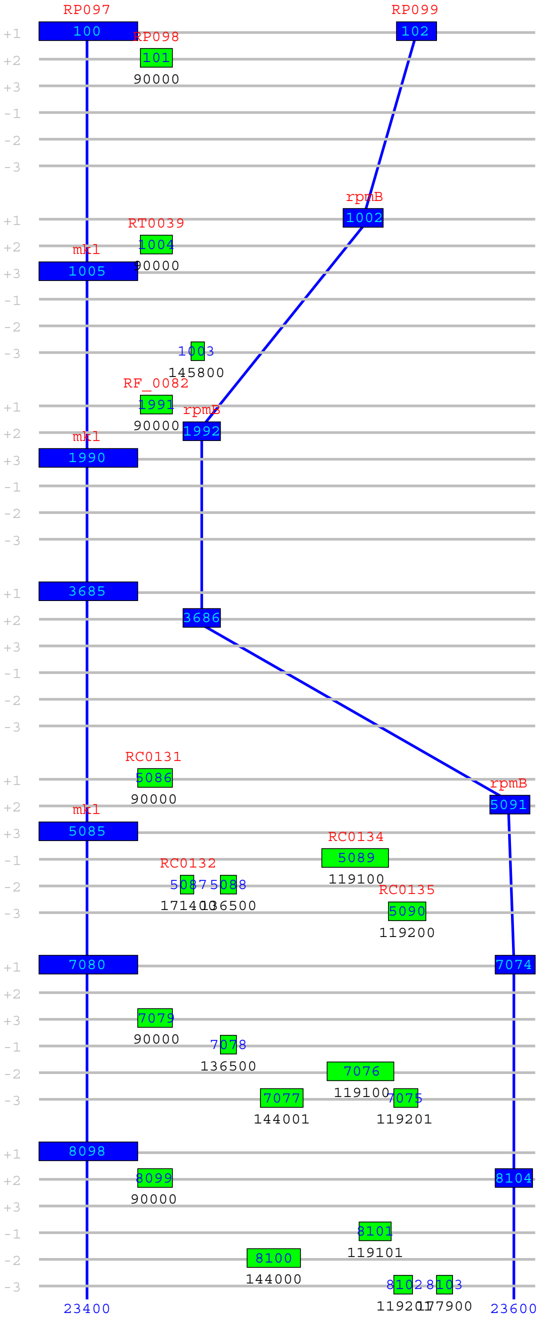

1 Rickettsia prowazekii str. Madrid E, complete genome
2 Rickettsia typhi str. wilmington, complete genome
3 Rickettsia felis URRWXCal2, complete genome
4 Rickettsia akari str. Hartford chromosome, whole genome shotgun sequence
5 Rickettsia conorii str. Malish 7, complete genome
6 Rickettsia sibirica 246 rsib_agncrt, whole genome shotgun sequence
7 Rickettsia rickettsii chromosome, whole genome shotgun sequence

Reg_id: 377

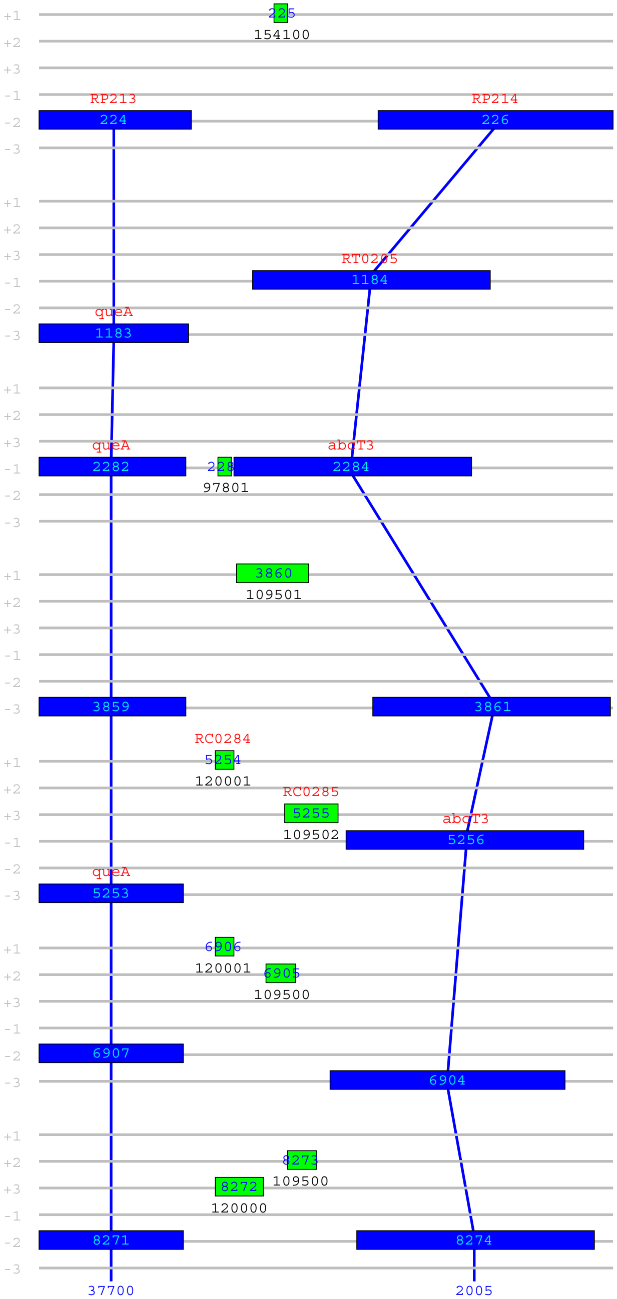

1 Rickettsia prowazekii str. Madrid E, complete genome
2 Rickettsia typhi str. wilmington, complete genome
3 Rickettsia felis URRWXCal2, complete genome
4 Rickettsia akari str. Hartford chromosome, whole genome shotgun sequence
5 Rickettsia conorii str. Malish 7, complete genome
6 Rickettsia sibirica 246 rsib_agncrt, whole genome shotgun sequence
7 Rickettsia rickettsii chromosome, whole genome shotgun sequence

Reg_id: 378

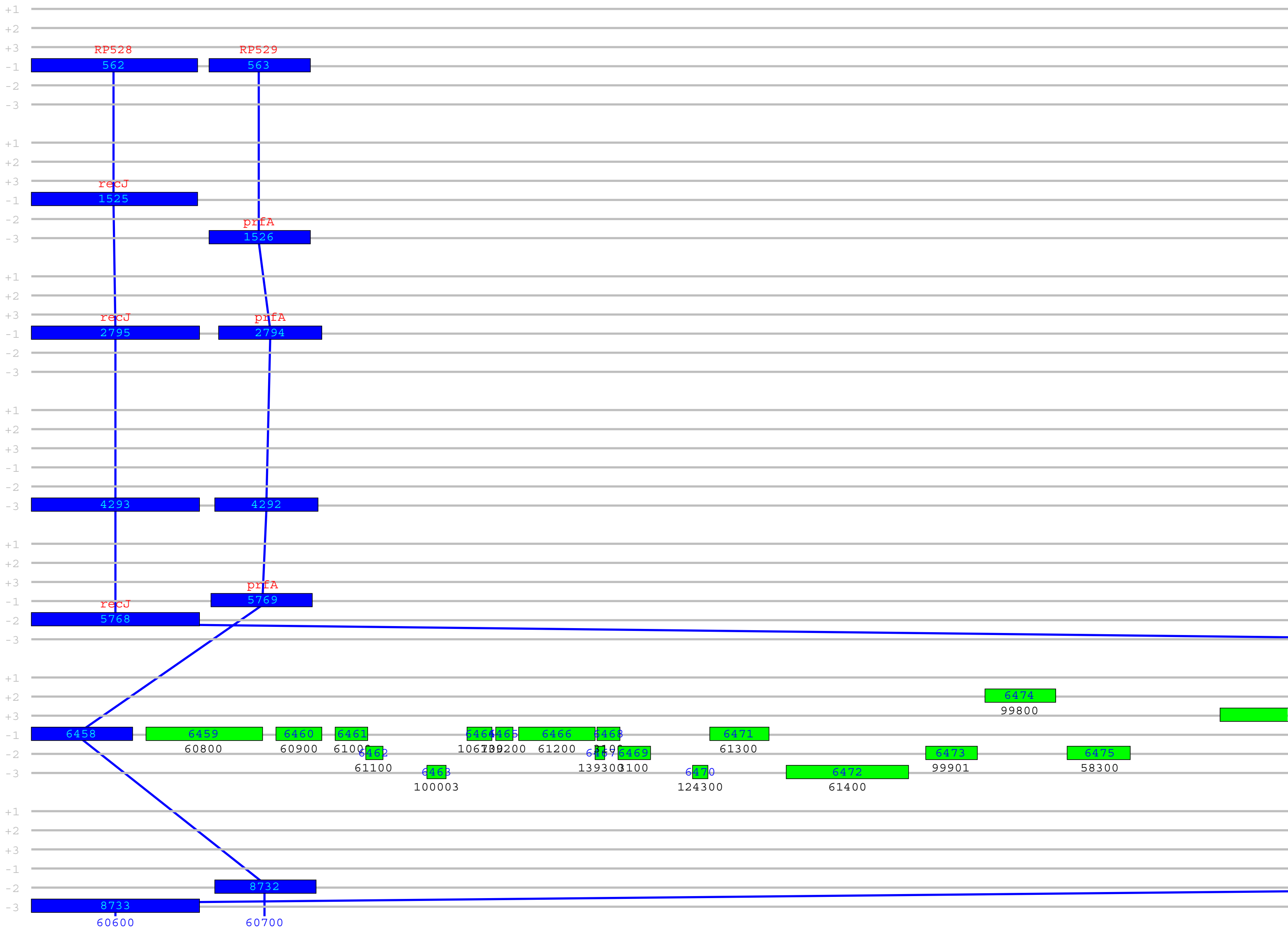

1 Rickettsia prowazekii str. Madrid E, complete genome
2 Rickettsia typhi str. wilmington, complete genome
3 Rickettsia felis URRWXCal2, complete genome
4 Rickettsia akari str. Hartford chromosome, whole genome shotgun sequence
5 Rickettsia conorii str. Malish 7, complete genome
6 Rickettsia sibirica 246 rsib_agncrt, whole genome shotgun sequence
7 Rickettsia rickettsii chromosome, whole genome shotgun sequence

Reg_id: 382

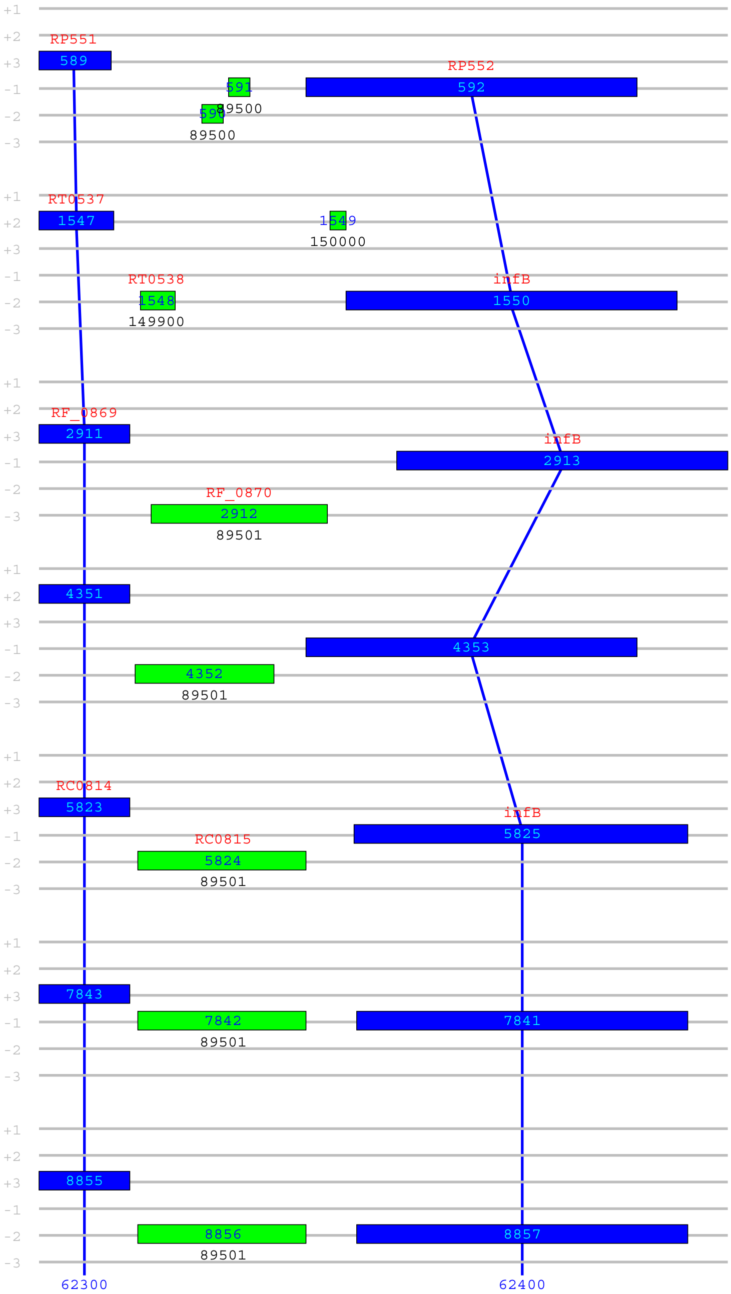

1 Rickettsia prowazekii str. Madrid E, complete genome
2 Rickettsia typhi str. wilmington, complete genome
3 Rickettsia felis URRWXCal2, complete genome
4 Rickettsia akari str. Hartford chromosome, whole genome shotgun sequence
5 Rickettsia conorii str. Malish 7, complete genome
6 Rickettsia sibirica 246 rsib_agncrt, whole genome shotgun sequence
7 Rickettsia rickettsii chromosome, whole genome shotgun sequence

Reg_id: 385

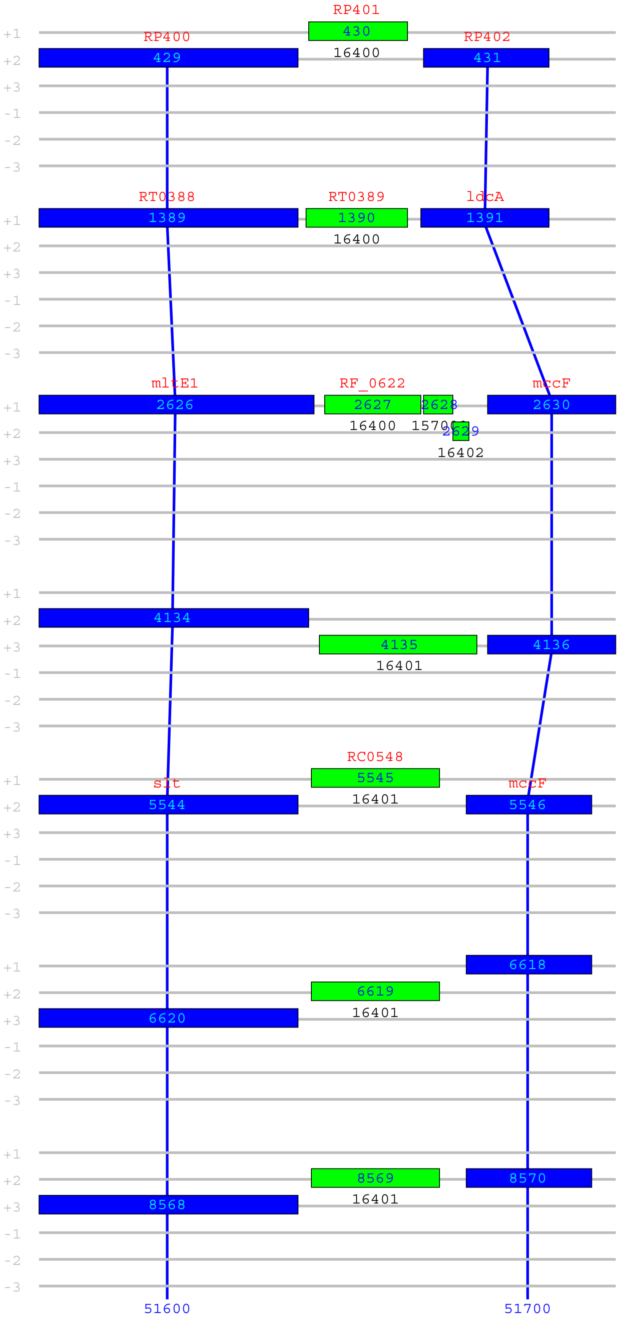

1 Rickettsia prowazekii str. Madrid E, complete genome
2 Rickettsia typhi str. wilmington, complete genome
3 Rickettsia felis URRWXCal2, complete genome
4 Rickettsia akari str. Hartford chromosome, whole genome shotgun sequence
5 Rickettsia conorii str. Malish 7, complete genome
6 Rickettsia sibirica 246 rsib_agncrt, whole genome shotgun sequence
7 Rickettsia rickettsii chromosome, whole genome shotgun sequence

Reg_id: 386

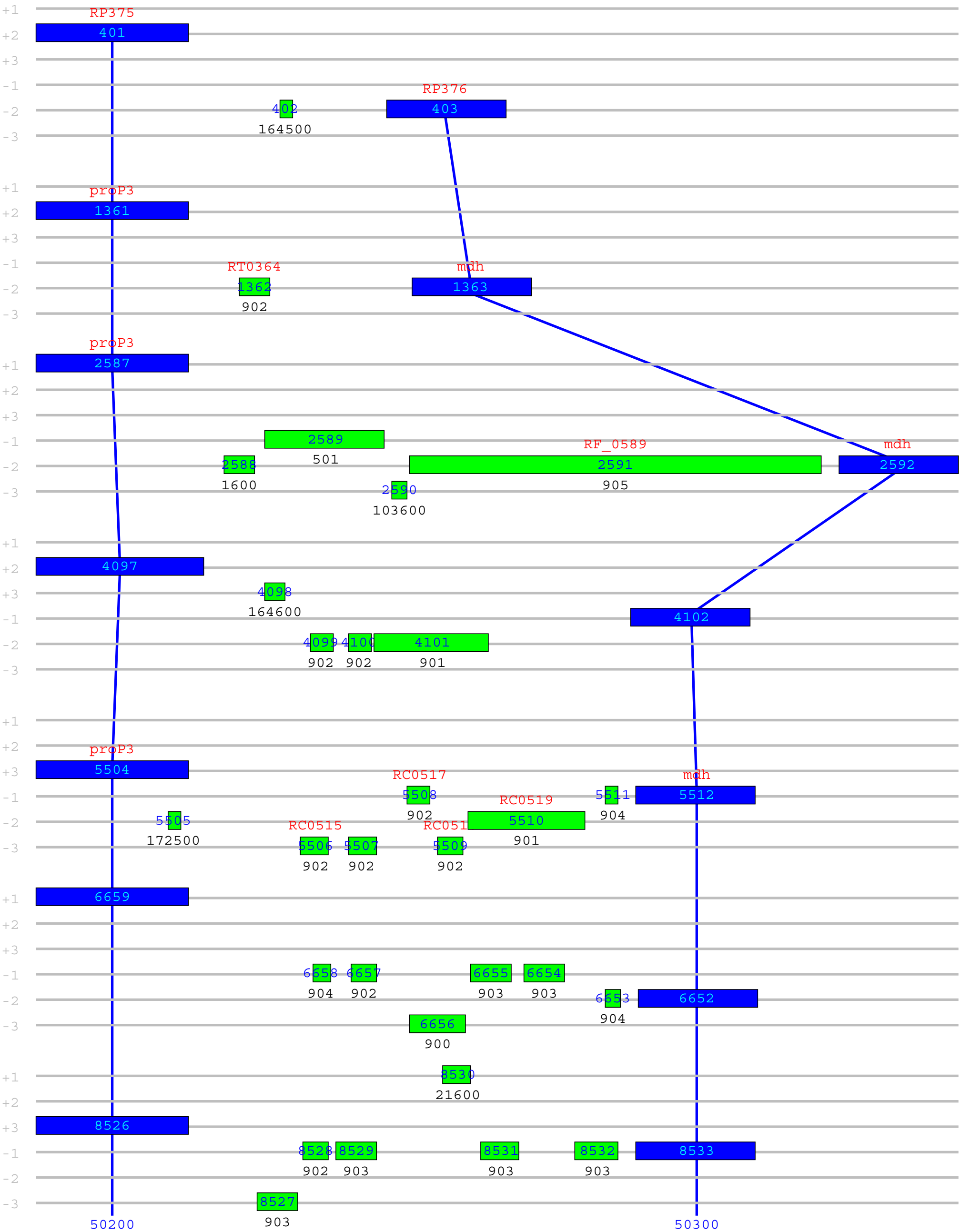

1 Rickettsia prowazekii str. Madrid E, complete genome
2 Rickettsia typhi str. wilmington, complete genome
3 Rickettsia felis URRWXCal2, complete genome
4 Rickettsia akari str. Hartford chromosome, whole genome shotgun sequence
5 Rickettsia conorii str. Malish 7, complete genome
6 Rickettsia sibirica 246 rsib_agncrt, whole genome shotgun sequence
7 Rickettsia rickettsii chromosome, whole genome shotgun sequence

Reg_id: 387

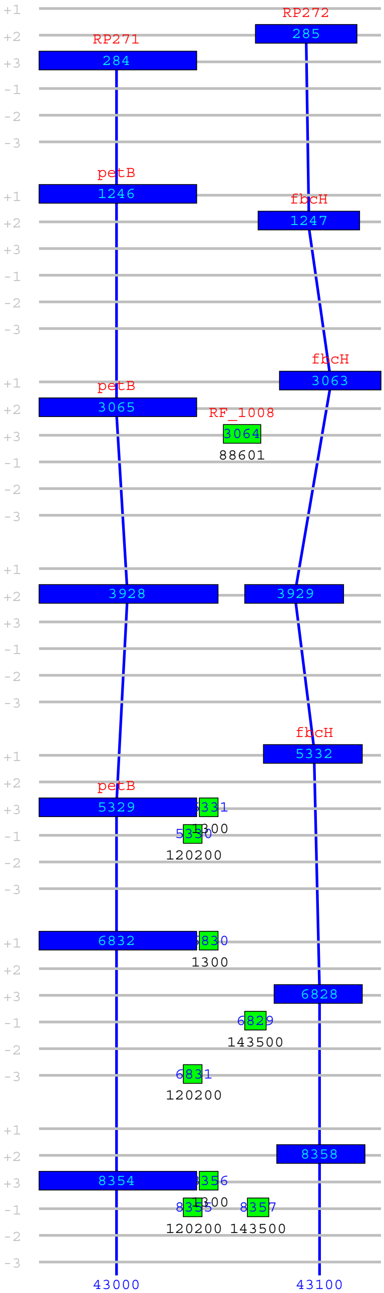

1 Rickettsia prowazekii str. Madrid E, complete genome
2 Rickettsia typhi str. wilmington, complete genome
3 Rickettsia felis URRWXCal2, complete genome
4 Rickettsia akari str. Hartford chromosome, whole genome shotgun sequence
5 Rickettsia conorii str. Malish 7, complete genome
6 Rickettsia sibirica 246 rsib_agncrt, whole genome shotgun sequence
7 Rickettsia rickettsii chromosome, whole genome shotgun sequence

Reg_id: 388

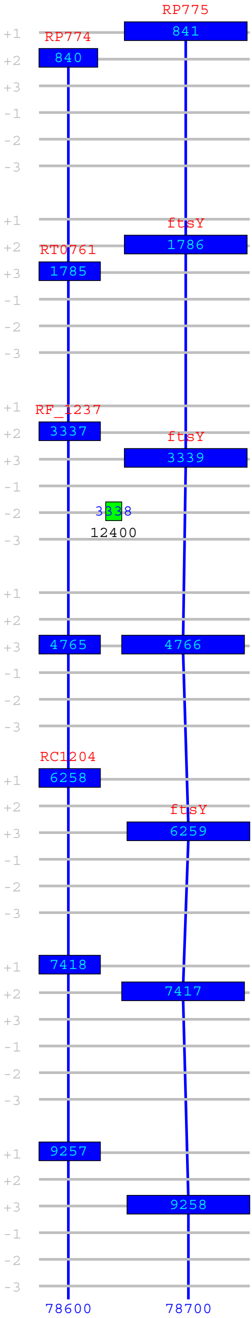

1 Rickettsia prowazekii str. Madrid E, complete genome
2 Rickettsia typhi str. wilmington, complete genome
3 Rickettsia felis URRWXCal2, complete genome
4 Rickettsia akari str. Hartford chromosome, whole genome shotgun sequence
5 Rickettsia conorii str. Malish 7, complete genome
6 Rickettsia sibirica 246 rsib_agncrt, whole genome shotgun sequence
7 Rickettsia rickettsii chromosome, whole genome shotgun sequence

Reg_id: 391

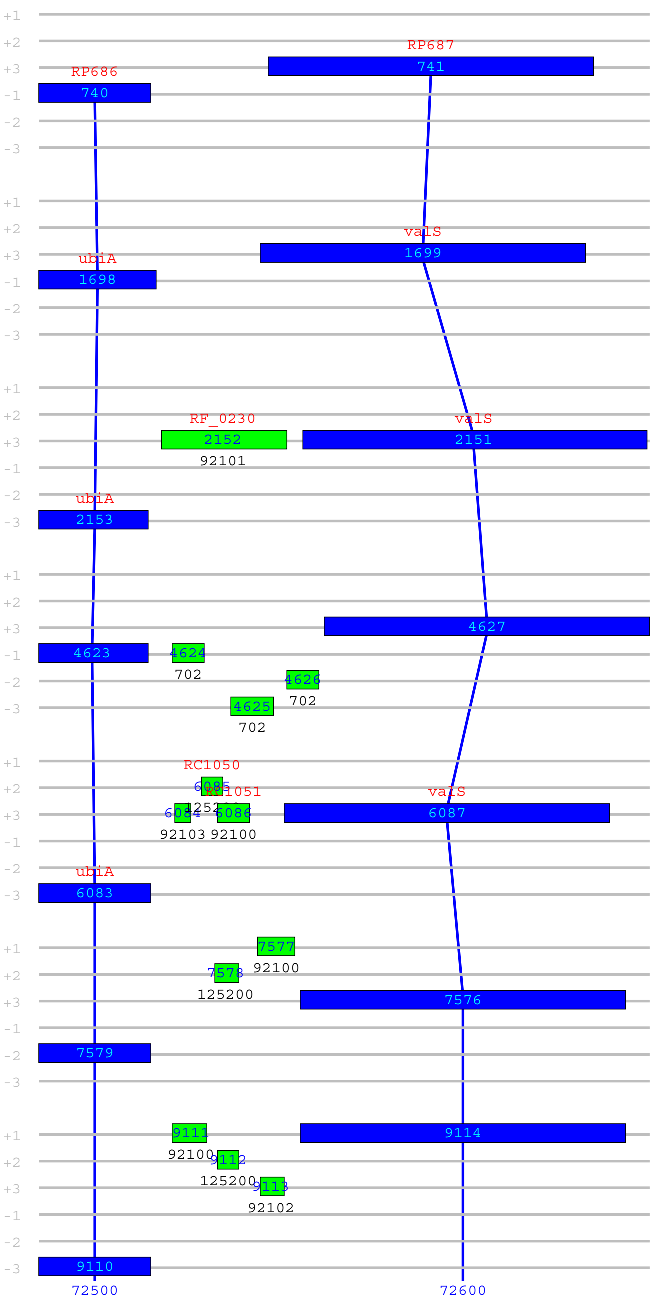

1 Rickettsia prowazekii str. Madrid E, complete genome
2 Rickettsia typhi str. wilmington, complete genome
3 Rickettsia felis URRWXCal2, complete genome
4 Rickettsia akari str. Hartford chromosome, whole genome shotgun sequence
5 Rickettsia conorii str. Malish 7, complete genome
6 Rickettsia sibirica 246 rsib_agncrt, whole genome shotgun sequence
7 Rickettsia rickettsii chromosome, whole genome shotgun sequence

Reg_id: 394

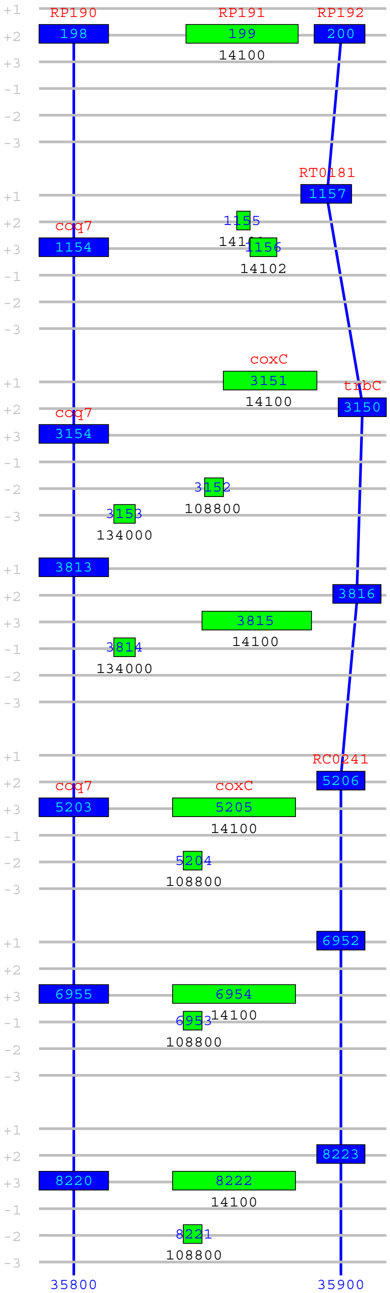

1 Rickettsia prowazekii str. Madrid E, complete genome
2 Rickettsia typhi str. wilmington, complete genome
3 Rickettsia felis URRWXCal2, complete genome
4 Rickettsia akari str. Hartford chromosome, whole genome shotgun sequence
5 Rickettsia conorii str. Malish 7, complete genome
6 Rickettsia sibirica 246 rsib_agncrt, whole genome shotgun sequence
7 Rickettsia rickettsii chromosome, whole genome shotgun sequence

Reg_id: 395

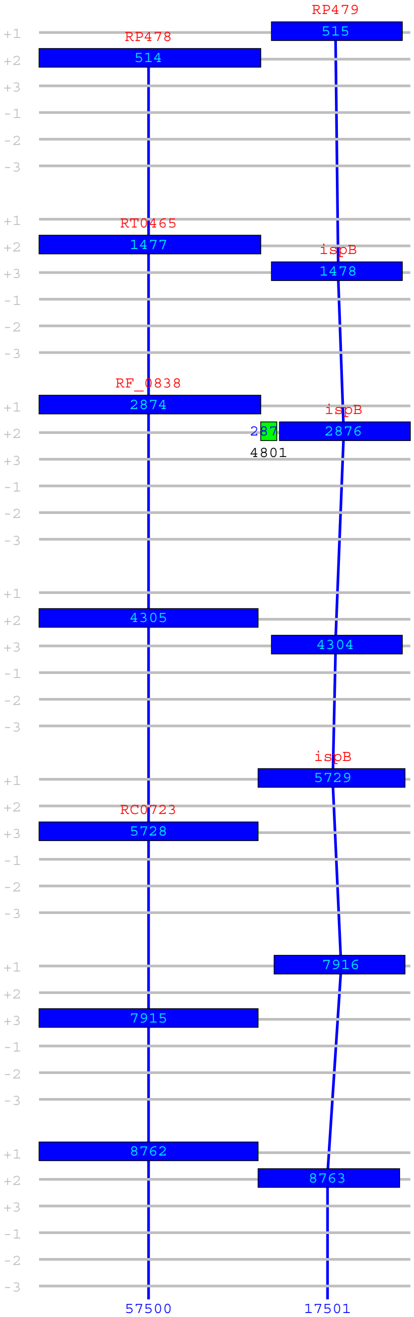

1 Rickettsia prowazekii str. Madrid E, complete genome
2 Rickettsia typhi str. wilmington, complete genome
3 Rickettsia felis URRWXCal2, complete genome
4 Rickettsia akari str. Hartford chromosome, whole genome shotgun sequence
5 Rickettsia conorii str. Malish 7, complete genome
6 Rickettsia sibirica 246 rsib_agncrt, whole genome shotgun sequence
7 Rickettsia rickettsii chromosome, whole genome shotgun sequence

Reg_id: 396

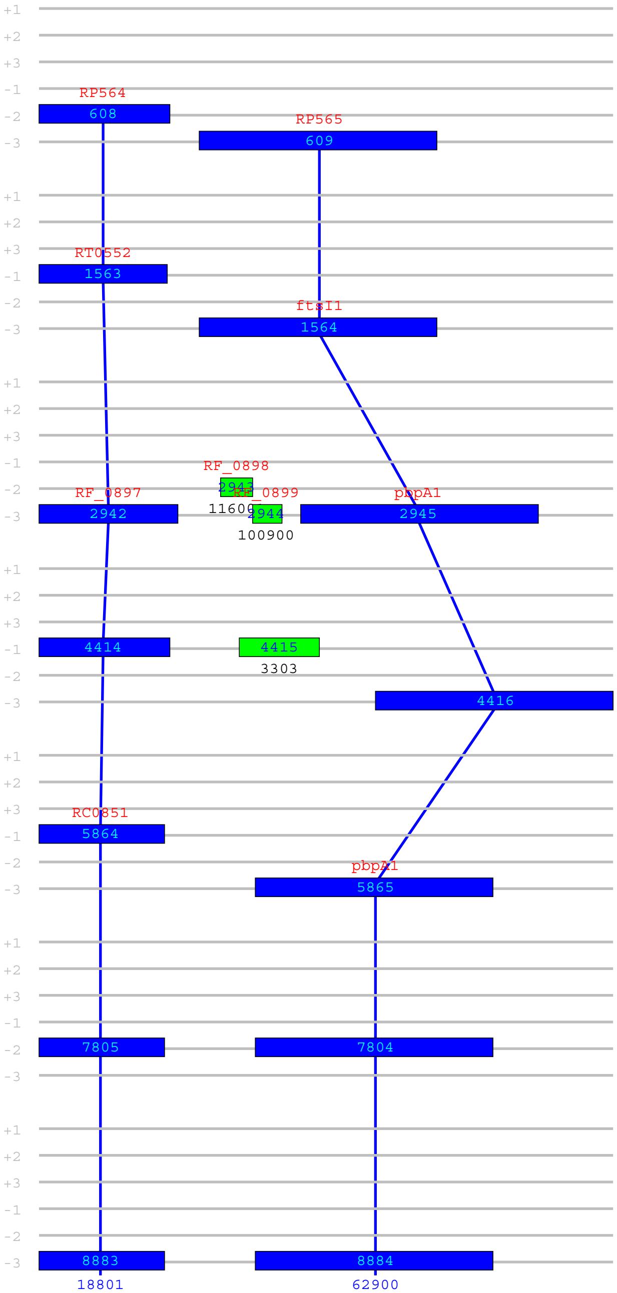

1 Rickettsia prowazekii str. Madrid E, complete genome
2 Rickettsia typhi str. wilmington, complete genome
3 Rickettsia felis URRWXCal2, complete genome
4 Rickettsia akari str. Hartford chromosome, whole genome shotgun sequence
5 Rickettsia conorii str. Malish 7, complete genome
6 Rickettsia sibirica 246 rsib_agncrt, whole genome shotgun sequence
7 Rickettsia rickettsii chromosome, whole genome shotgun sequence

Reg_id: 397

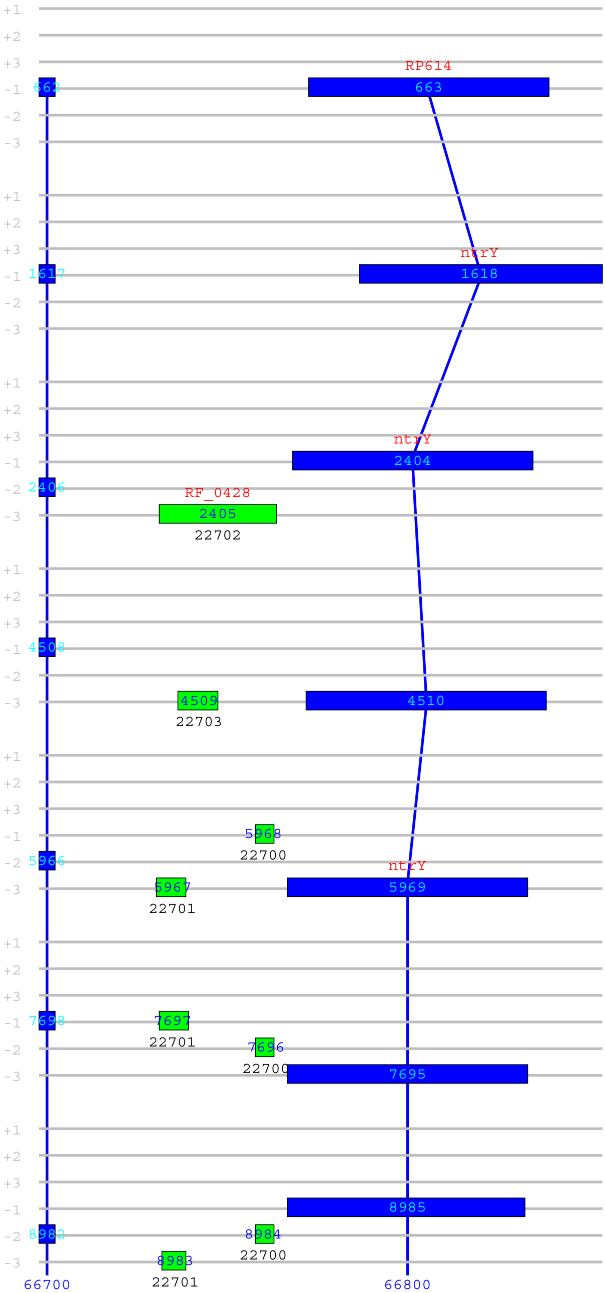

1 Rickettsia prowazekii str. Madrid E, complete genome
2 Rickettsia typhi str. wilmington, complete genome
3 Rickettsia felis URRWXCal2, complete genome
4 Rickettsia akari str. Hartford chromosome, whole genome shotgun sequence
5 Rickettsia conorii str. Malish 7, complete genome
6 Rickettsia sibirica 246 rsib_agncrt, whole genome shotgun sequence
7 Rickettsia rickettsii chromosome, whole genome shotgun sequence

Reg_id: 398

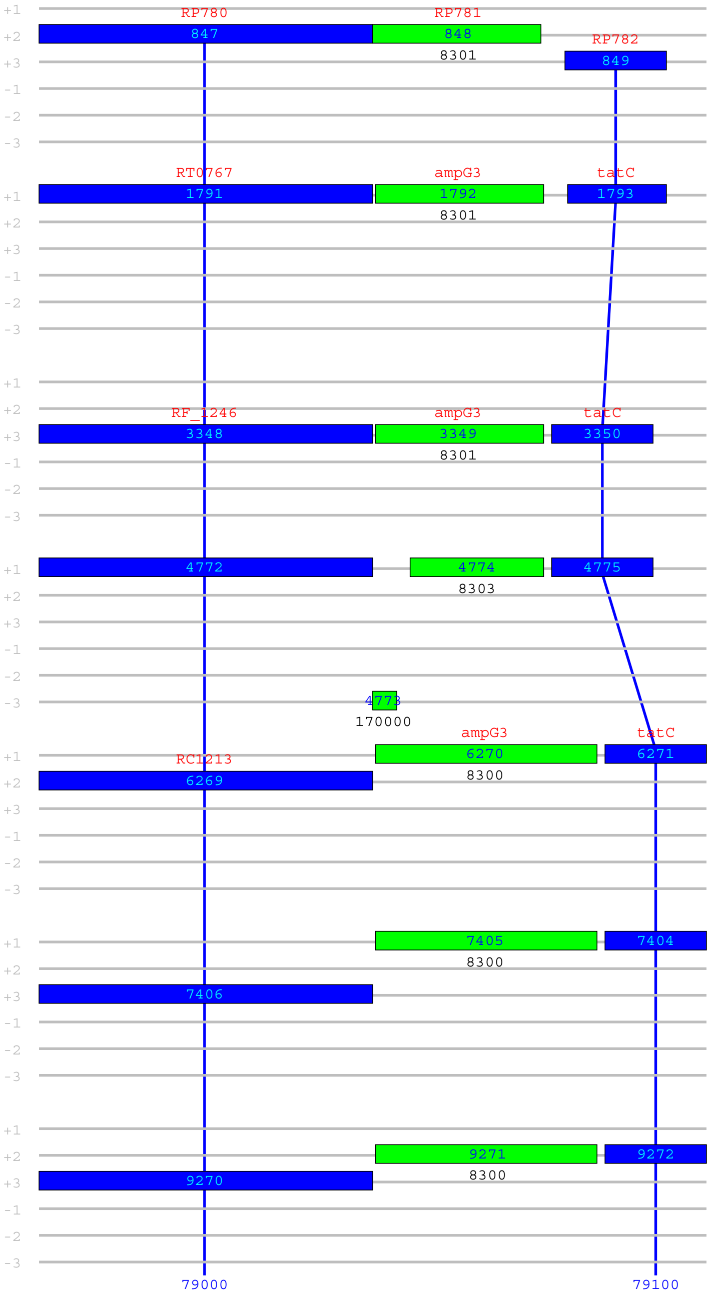

1 Rickettsia prowazekii str. Madrid E, complete genome
2 Rickettsia typhi str. wilmington, complete genome
3 Rickettsia felis URRWXCal2, complete genome
4 Rickettsia akari str. Hartford chromosome, whole genome shotgun sequence
5 Rickettsia conorii str. Malish 7, complete genome
6 Rickettsia sibirica 246 rsib_agncrt, whole genome shotgun sequence
7 Rickettsia rickettsii chromosome, whole genome shotgun sequence

Reg_id: 400

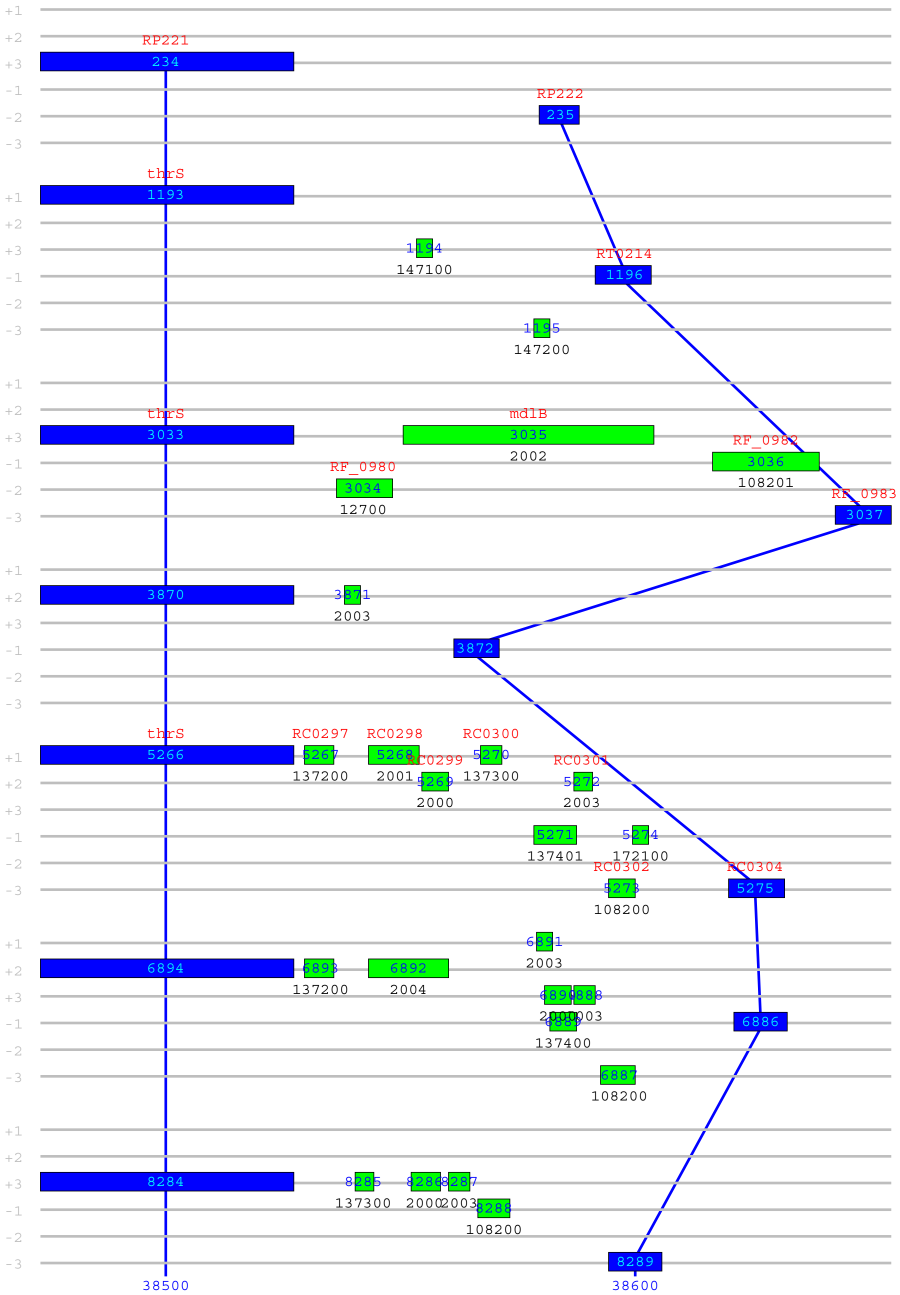

1 Rickettsia prowazekii str. Madrid E, complete genome
2 Rickettsia typhi str. wilmington, complete genome
3 Rickettsia felis URRWXCal2, complete genome
4 Rickettsia akari str. Hartford chromosome, whole genome shotgun sequence
5 Rickettsia conorii str. Malish 7, complete genome
6 Rickettsia sibirica 246 rsib_agncrt, whole genome shotgun sequence
7 Rickettsia rickettsii chromosome, whole genome shotgun sequence

Reg_id: 401

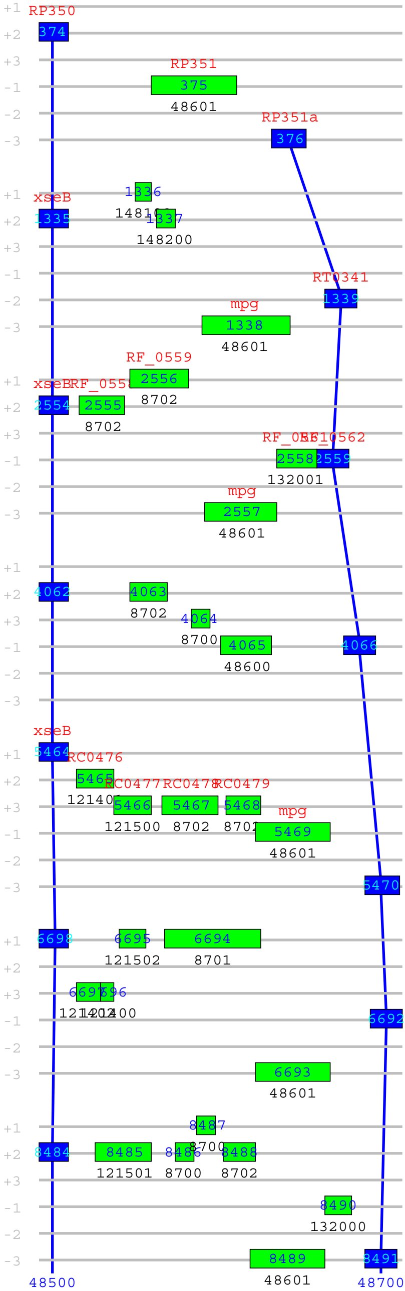

1 Rickettsia prowazekii str. Madrid E, complete genome
2 Rickettsia typhi str. wilmington, complete genome
3 Rickettsia felis URRWXCal2, complete genome
4 Rickettsia akari str. Hartford chromosome, whole genome shotgun sequence
5 Rickettsia conorii str. Malish 7, complete genome
6 Rickettsia sibirica 246 rsib_agncrt, whole genome shotgun sequence
7 Rickettsia rickettsii chromosome, whole genome shotgun sequence

Reg_id: 403

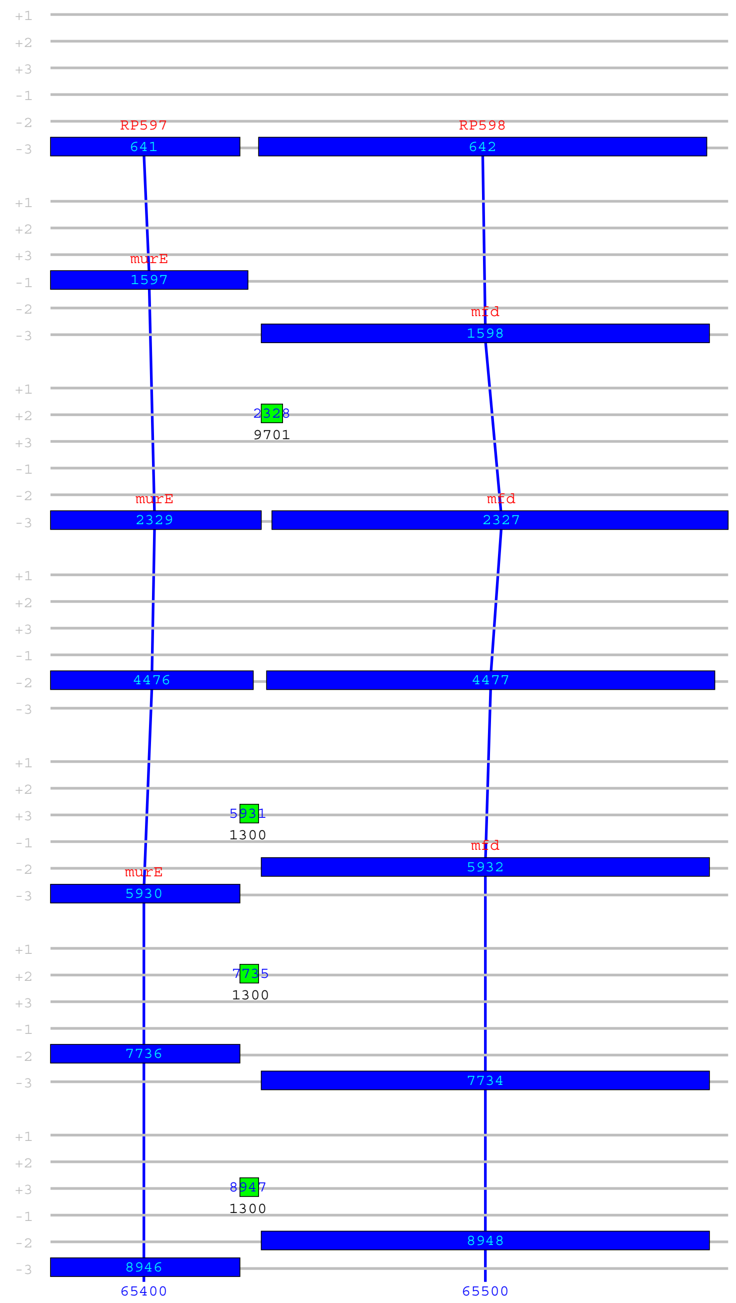

1 Rickettsia prowazekii str. Madrid E, complete genome
2 Rickettsia typhi str. wilmington, complete genome
3 Rickettsia felis URRWXCal2, complete genome
4 Rickettsia akari str. Hartford chromosome, whole genome shotgun sequence
5 Rickettsia conorii str. Malish 7, complete genome
6 Rickettsia sibirica 246 rsib_agncrt, whole genome shotgun sequence
7 Rickettsia rickettsii chromosome, whole genome shotgun sequence

Reg_id: 404

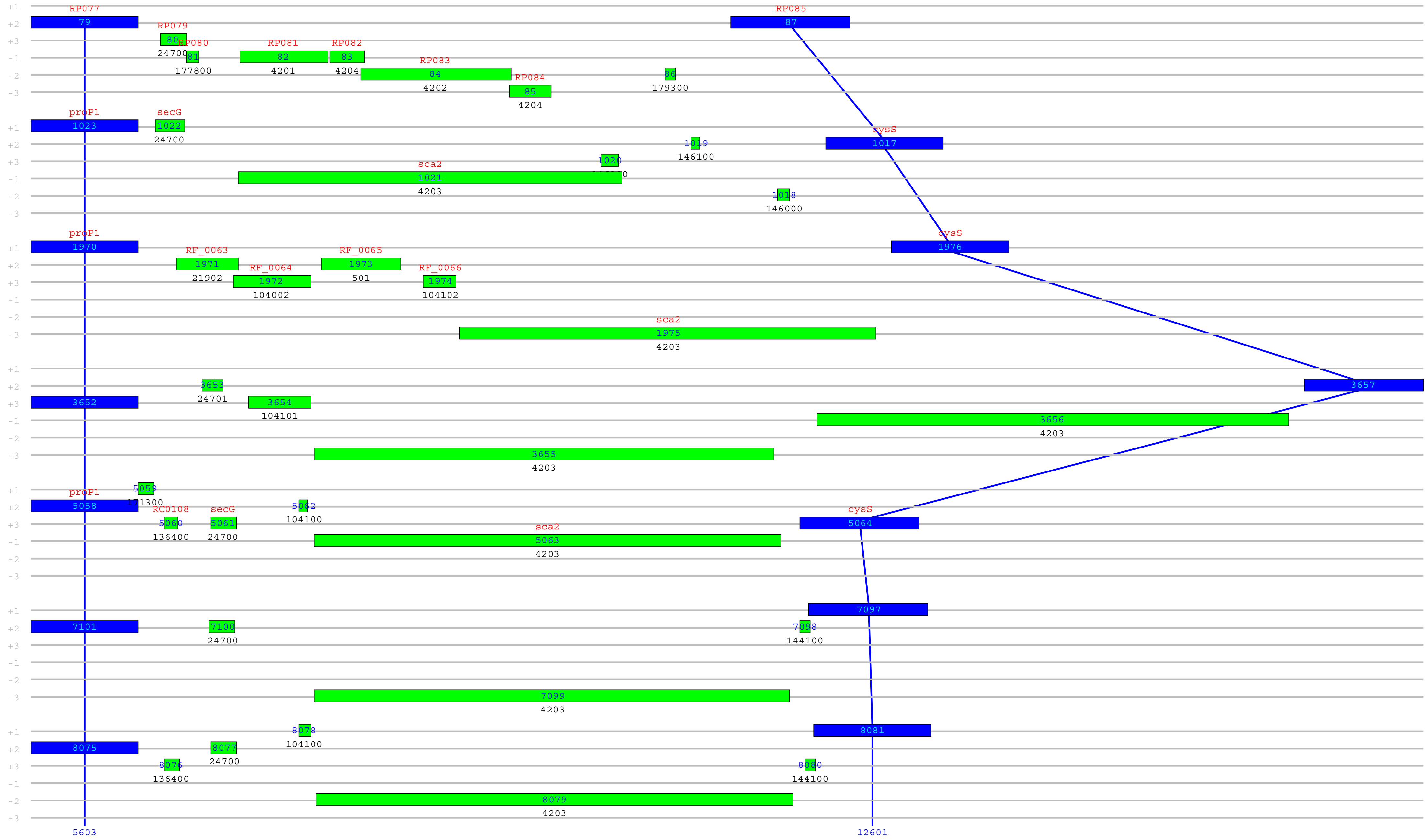

1 Rickettsia prowazekii str. Madrid E, complete genome
2 Rickettsia typhi str. wilmington, complete genome
3 Rickettsia felis URRWXCal2, complete genome
4 Rickettsia akari str. Hartford chromosome, whole genome shotgun sequence
5 Rickettsia conorii str. Malish 7, complete genome
6 Rickettsia sibirica 246 rsib_agncrt, whole genome shotgun sequence
7 Rickettsia rickettsii chromosome, whole genome shotgun sequence

Reg_id: 412

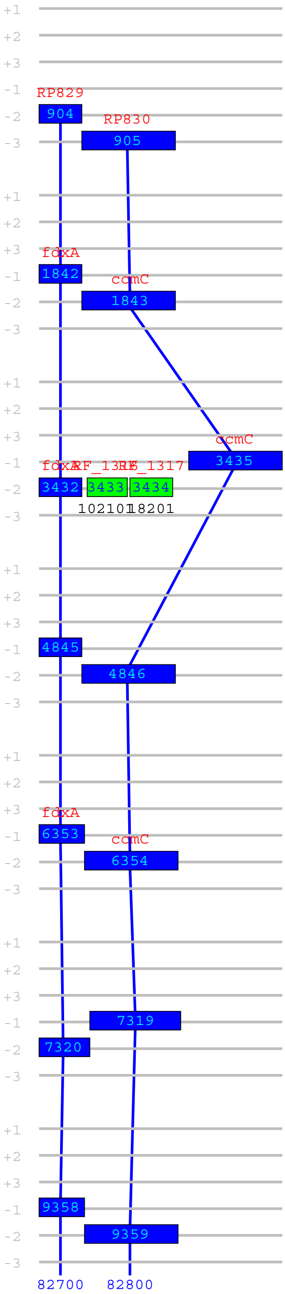

1 Rickettsia prowazekii str. Madrid E, complete genome
2 Rickettsia typhi str. wilmington, complete genome
3 Rickettsia felis URRWXCal2, complete genome
4 Rickettsia akari str. Hartford chromosome, whole genome shotgun sequence
5 Rickettsia conorii str. Malish 7, complete genome
6 Rickettsia sibirica 246 rsib_agncrt, whole genome shotgun sequence
7 Rickettsia rickettsii chromosome, whole genome shotgun sequence

Reg_id: 415

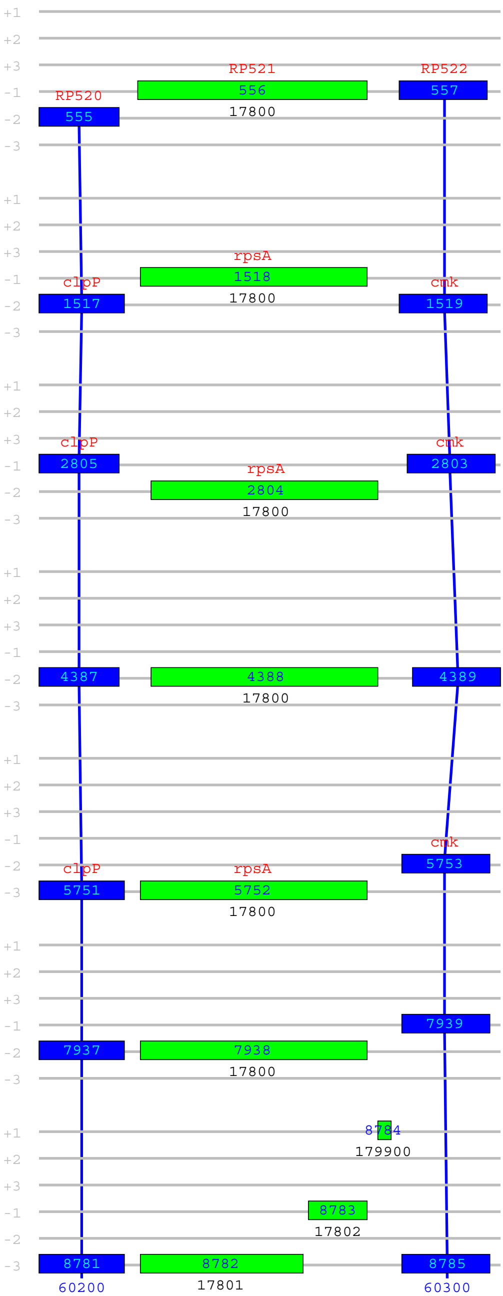

1 Rickettsia prowazekii str. Madrid E, complete genome
2 Rickettsia typhi str. wilmington, complete genome
3 Rickettsia felis URRWXCal2, complete genome
4 Rickettsia akari str. Hartford chromosome, whole genome shotgun sequence
5 Rickettsia conorii str. Malish 7, complete genome
6 Rickettsia sibirica 246 rsib_agncrt, whole genome shotgun sequence
7 Rickettsia rickettsii chromosome, whole genome shotgun sequence

Reg_id: 416

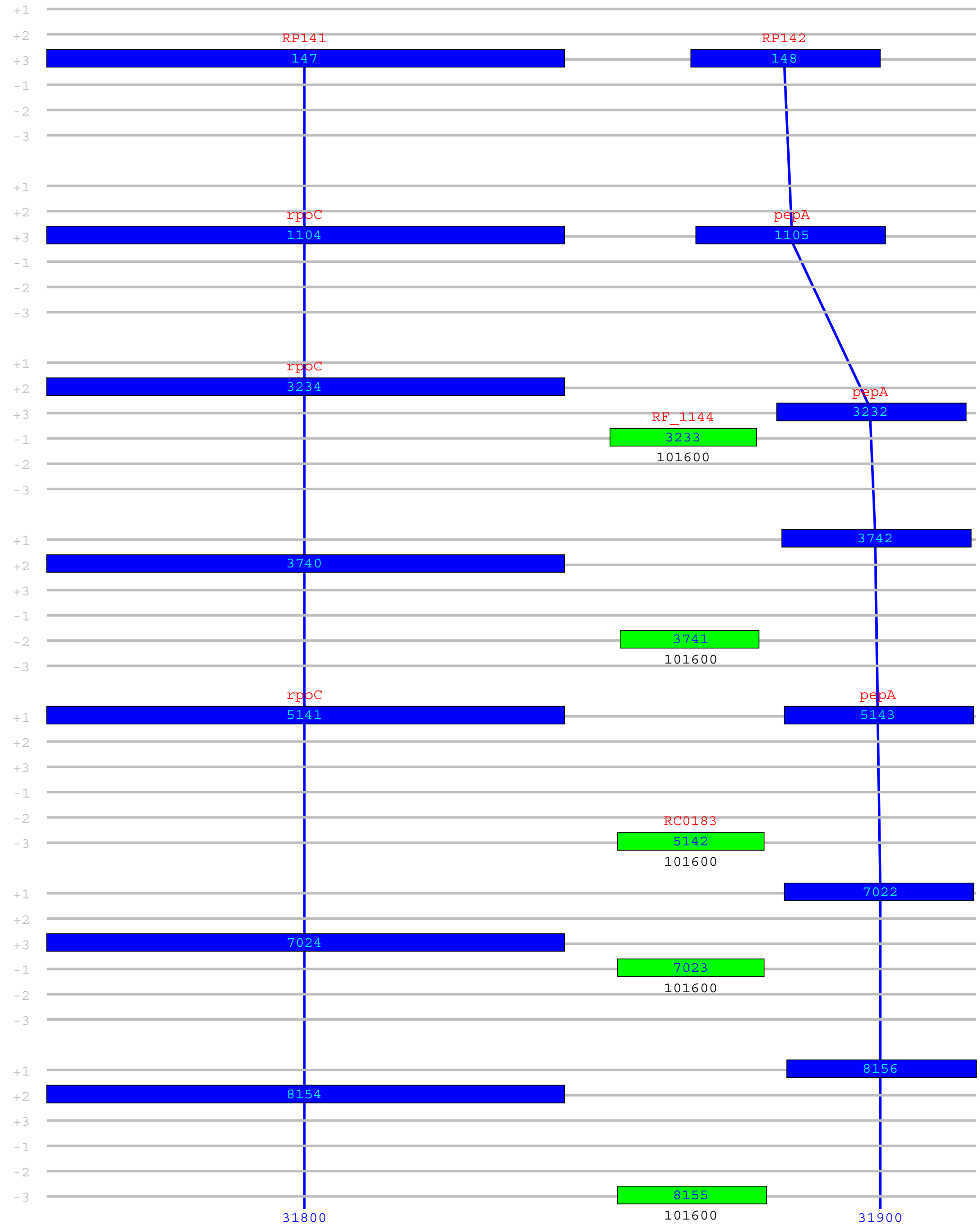

1 Rickettsia prowazekii str. Madrid E, complete genome
2 Rickettsia typhi str. wilmington, complete genome
3 Rickettsia felis URRWXCal2, complete genome
4 Rickettsia akari str. Hartford chromosome, whole genome shotgun sequence
5 Rickettsia conorii str. Malish 7, complete genome
6 Rickettsia sibirica 246 rsib_agncrt, whole genome shotgun sequence
7 Rickettsia rickettsii chromosome, whole genome shotgun sequence

Reg_id: 418

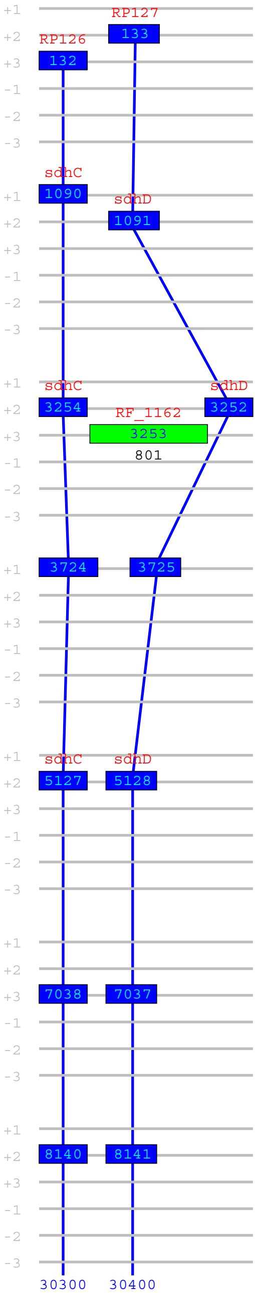

1 Rickettsia prowazekii str. Madrid E, complete genome
2 Rickettsia typhi str. wilmington, complete genome
3 Rickettsia felis URRWXCal2, complete genome
4 Rickettsia akari str. Hartford chromosome, whole genome shotgun sequence
5 Rickettsia conorii str. Malish 7, complete genome
6 Rickettsia sibirica 246 rsib_agncrt, whole genome shotgun sequence
7 Rickettsia rickettsii chromosome, whole genome shotgun sequence

Reg_id: 420

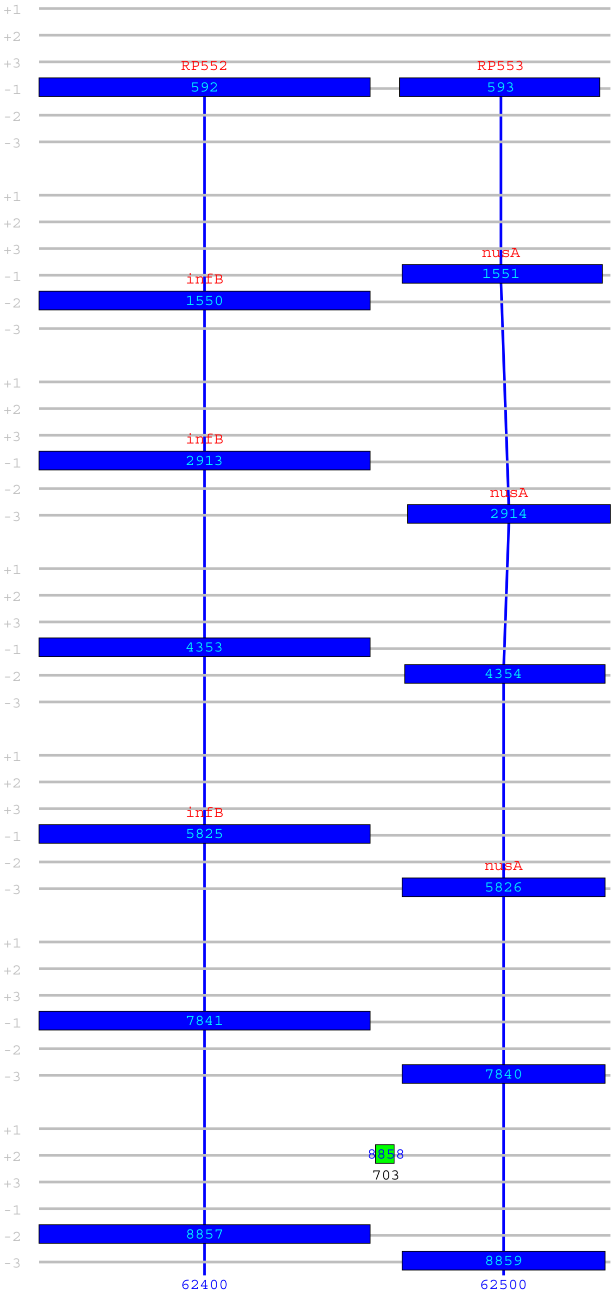

1 Rickettsia prowazekii str. Madrid E, complete genome
2 Rickettsia typhi str. wilmington, complete genome
3 Rickettsia felis URRWXCal2, complete genome
4 Rickettsia akari str. Hartford chromosome, whole genome shotgun sequence
5 Rickettsia conorii str. Malish 7, complete genome
6 Rickettsia sibirica 246 rsib_agncrt, whole genome shotgun sequence
7 Rickettsia rickettsii chromosome, whole genome shotgun sequence

Reg_id: 421

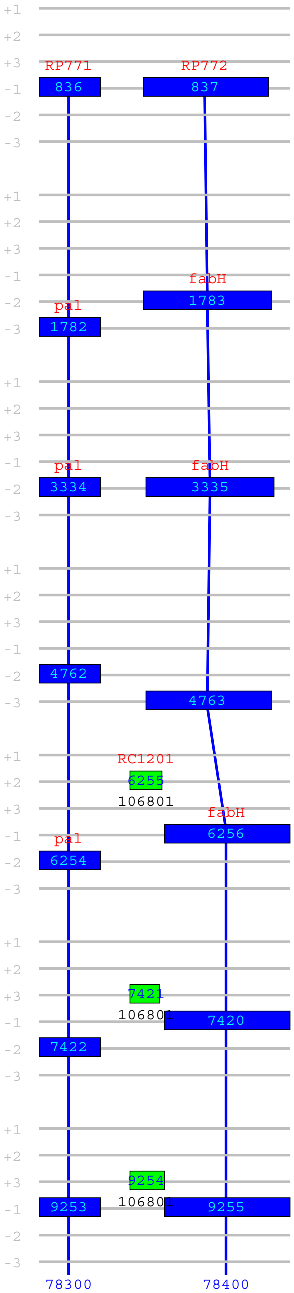

1 Rickettsia prowazekii str. Madrid E, complete genome
2 Rickettsia typhi str. wilmington, complete genome
3 Rickettsia felis URRWXCal2, complete genome
4 Rickettsia akari str. Hartford chromosome, whole genome shotgun sequence
5 Rickettsia conorii str. Malish 7, complete genome
6 Rickettsia sibirica 246 rsib_agncrt, whole genome shotgun sequence
7 Rickettsia rickettsii chromosome, whole genome shotgun sequence

Reg_id: 422

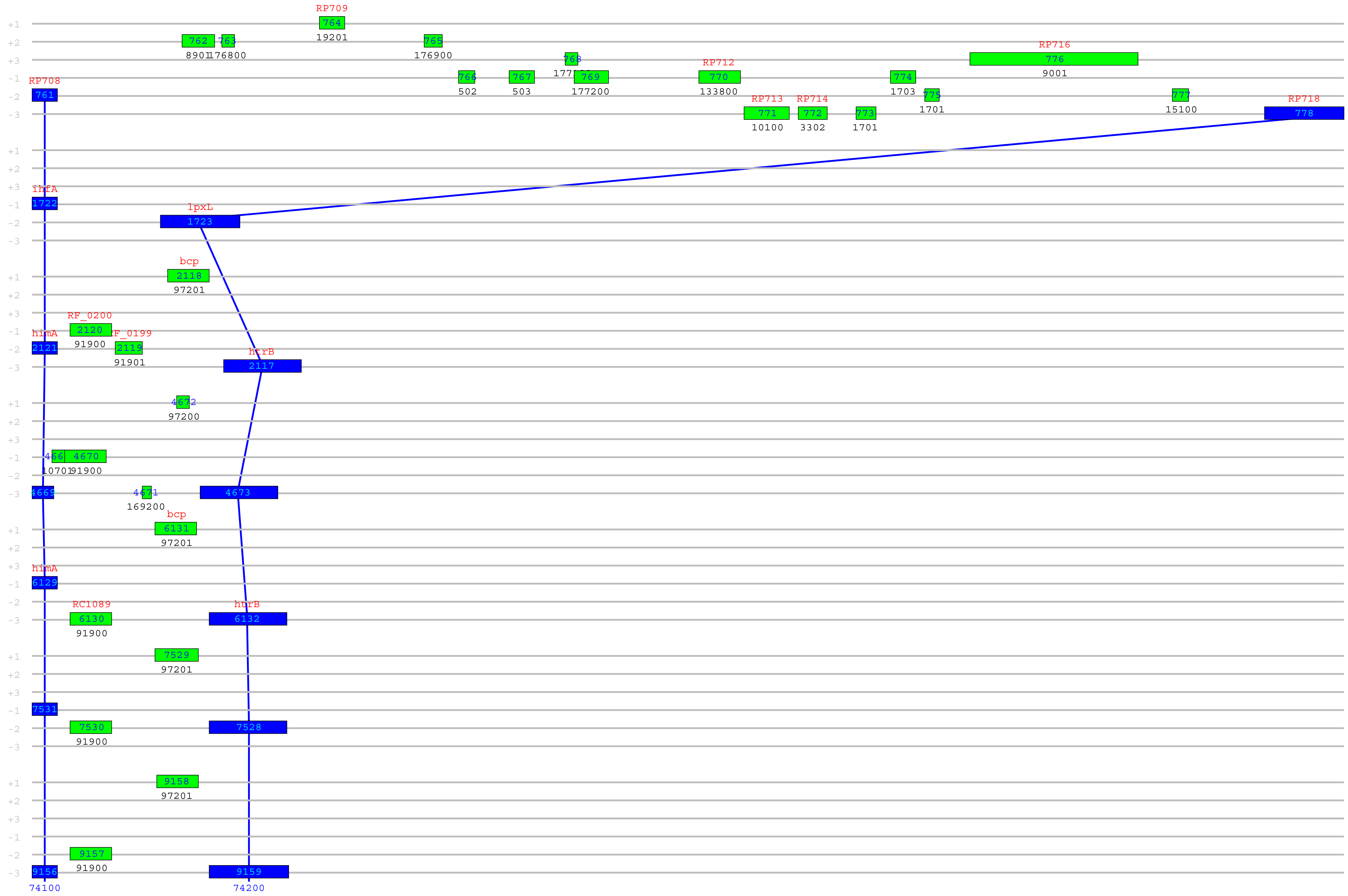

1 Rickettsia prowazekii str. Madrid E, complete genome
2 Rickettsia typhi str. wilmington, complete genome
3 Rickettsia felis URRWXCal2, complete genome
4 Rickettsia akari str. Hartford chromosome, whole genome shotgun sequence
5 Rickettsia conorii str. Malish 7, complete genome
6 Rickettsia sibirica 246 rsib_agncrt, whole genome shotgun sequence
7 Rickettsia rickettsii chromosome, whole genome shotgun sequence

Reg_id: 426

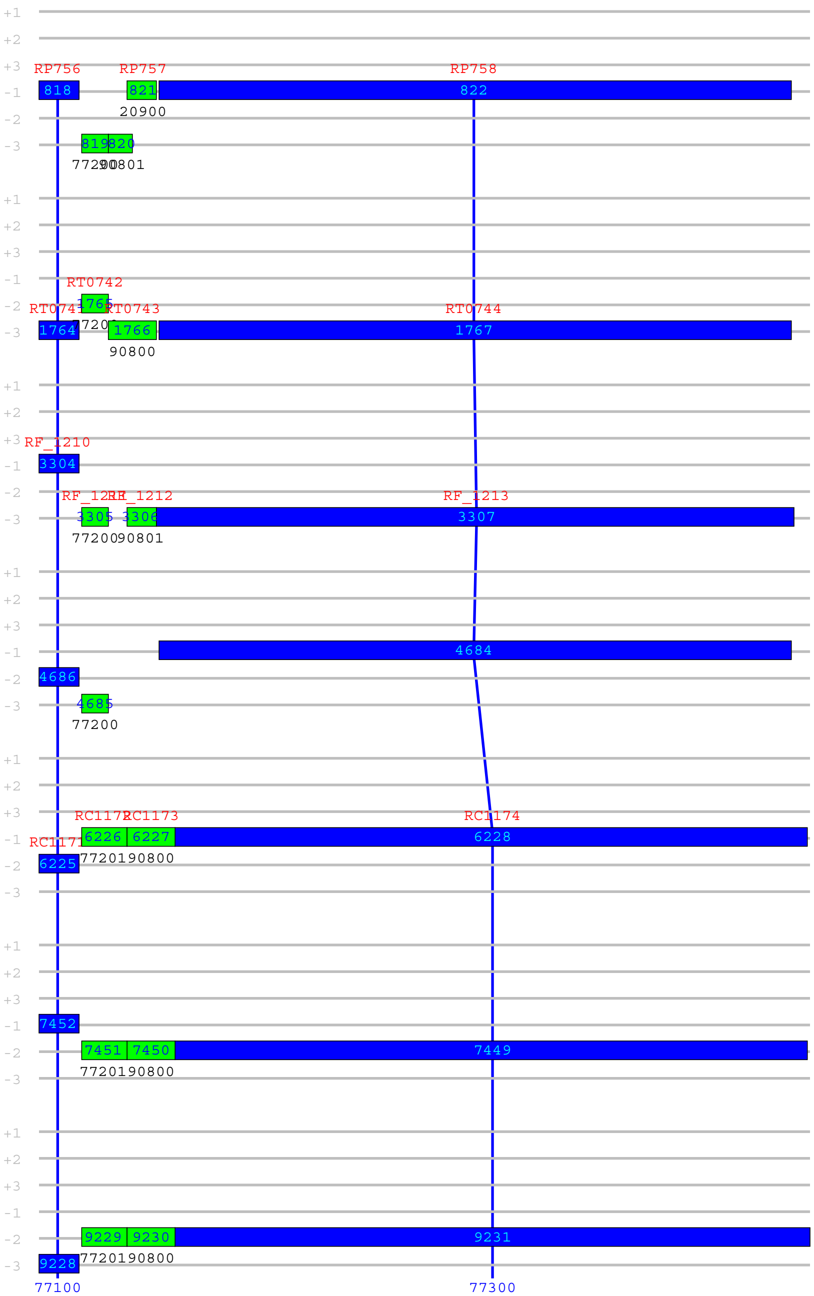

1 Rickettsia prowazekii str. Madrid E, complete genome
2 Rickettsia typhi str. wilmington, complete genome
3 Rickettsia felis URRWXCal2, complete genome
4 Rickettsia akari str. Hartford chromosome, whole genome shotgun sequence
5 Rickettsia conorii str. Malish 7, complete genome
6 Rickettsia sibirica 246 rsib_agncrt, whole genome shotgun sequence
7 Rickettsia rickettsii chromosome, whole genome shotgun sequence

Reg_id: 427

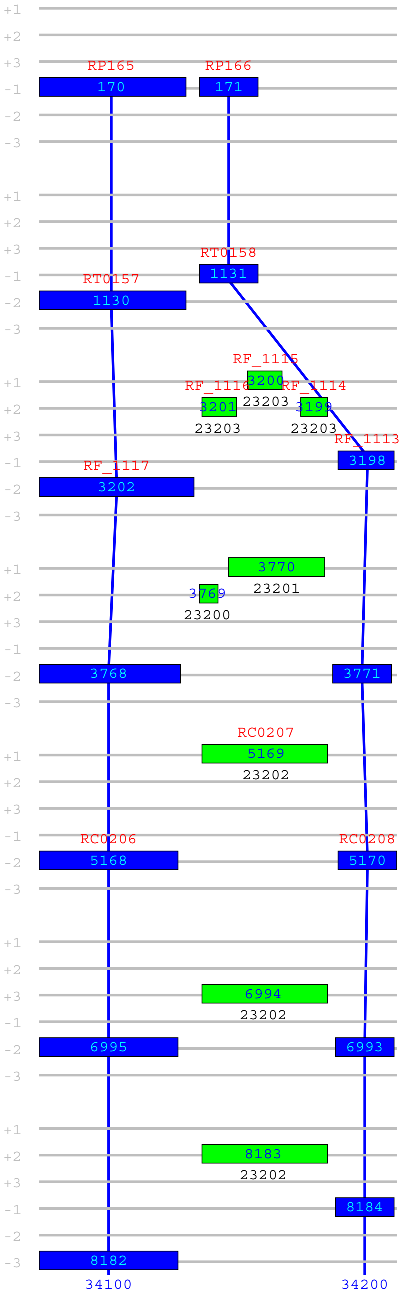

1 Rickettsia prowazekii str. Madrid E, complete genome
2 Rickettsia typhi str. wilmington, complete genome
3 Rickettsia felis URRWXCal2, complete genome
4 Rickettsia akari str. Hartford chromosome, whole genome shotgun sequence
5 Rickettsia conorii str. Malish 7, complete genome
6 Rickettsia sibirica 246 rsib_agncrt, whole genome shotgun sequence
7 Rickettsia rickettsii chromosome, whole genome shotgun sequence

Reg_id: 431

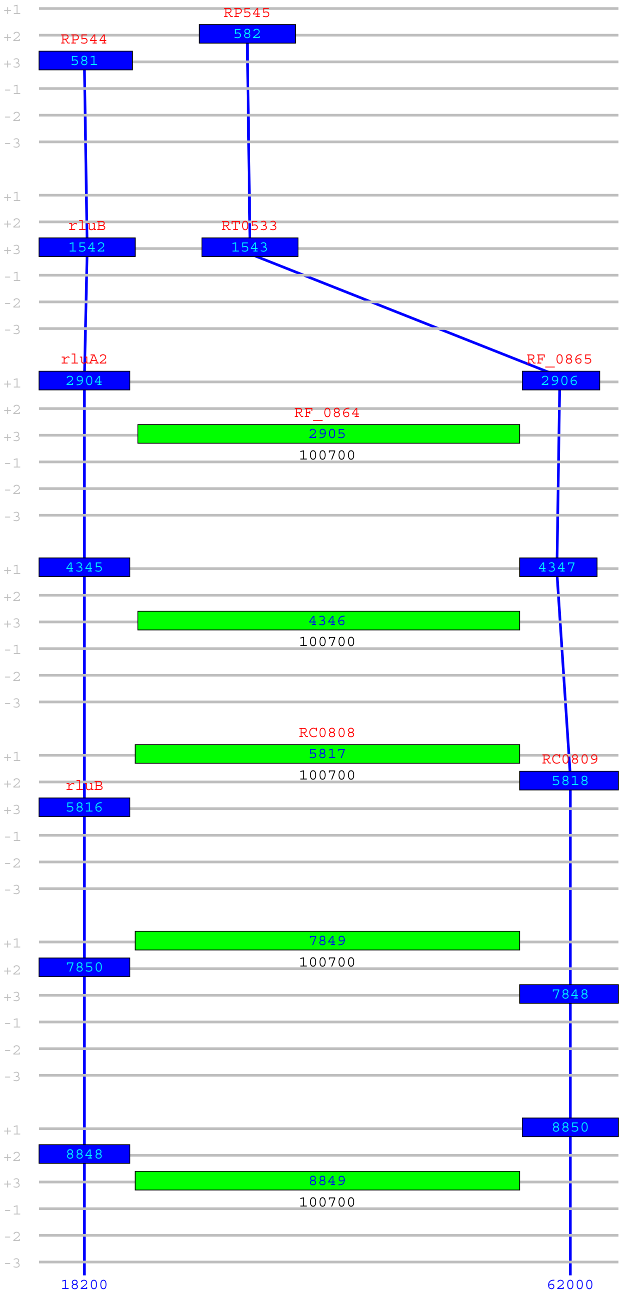

1 Rickettsia prowazekii str. Madrid E, complete genome
2 Rickettsia typhi str. wilmington, complete genome
3 Rickettsia felis URRWXCal2, complete genome
4 Rickettsia akari str. Hartford chromosome, whole genome shotgun sequence
5 Rickettsia conorii str. Malish 7, complete genome
6 Rickettsia sibirica 246 rsib_agncrt, whole genome shotgun sequence
7 Rickettsia rickettsii chromosome, whole genome shotgun sequence

Reg_id: 433

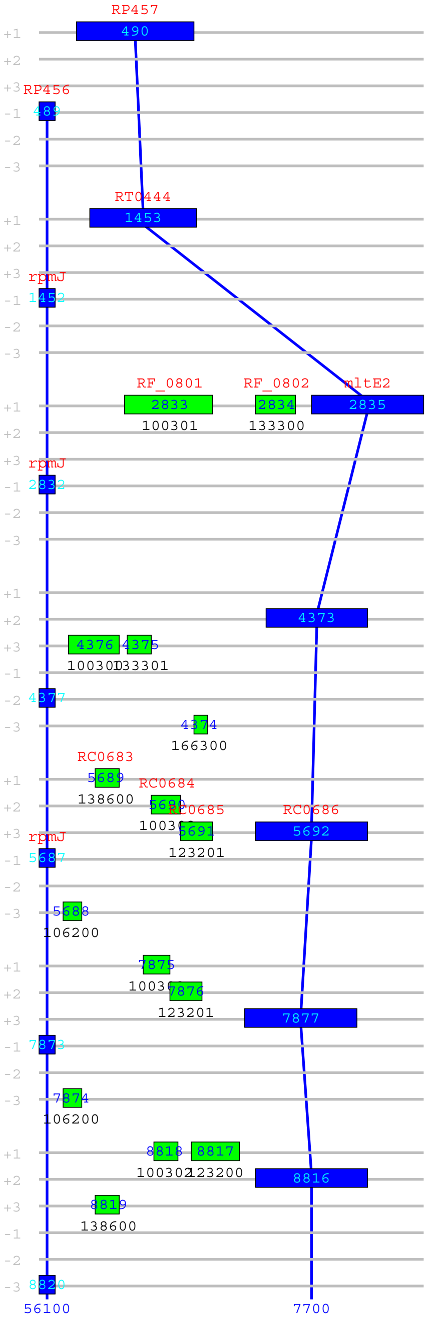

1 Rickettsia prowazekii str. Madrid E, complete genome
2 Rickettsia typhi str. wilmington, complete genome
3 Rickettsia felis URRWXCal2, complete genome
4 Rickettsia akari str. Hartford chromosome, whole genome shotgun sequence
5 Rickettsia conorii str. Malish 7, complete genome
6 Rickettsia sibirica 246 rsib_agncrt, whole genome shotgun sequence
7 Rickettsia rickettsii chromosome, whole genome shotgun sequence

Reg_id: 434

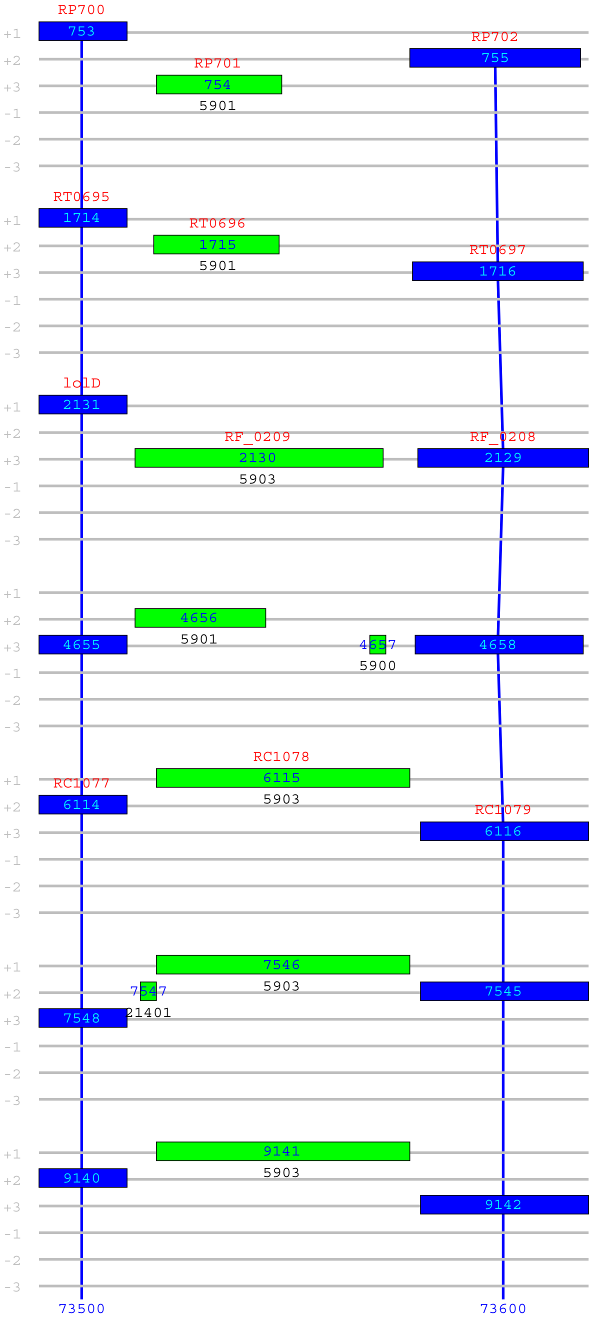

1 Rickettsia prowazekii str. Madrid E, complete genome
2 Rickettsia typhi str. wilmington, complete genome
3 Rickettsia felis URRWXCal2, complete genome
4 Rickettsia akari str. Hartford chromosome, whole genome shotgun sequence
5 Rickettsia conorii str. Malish 7, complete genome
6 Rickettsia sibirica 246 rsib_agncrt, whole genome shotgun sequence
7 Rickettsia rickettsii chromosome, whole genome shotgun sequence

Reg_id: 435

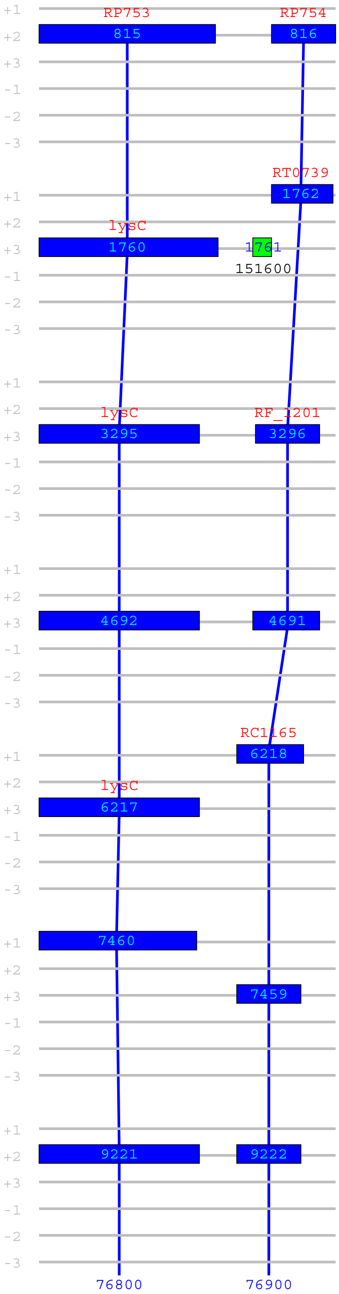

1 Rickettsia prowazekii str. Madrid E, complete genome
2 Rickettsia typhi str. wilmington, complete genome
3 Rickettsia felis URRWXCal2, complete genome
4 Rickettsia akari str. Hartford chromosome, whole genome shotgun sequence
5 Rickettsia conorii str. Malish 7, complete genome
6 Rickettsia sibirica 246 rsib_agncrt, whole genome shotgun sequence
7 Rickettsia rickettsii chromosome, whole genome shotgun sequence

Reg_id: 436

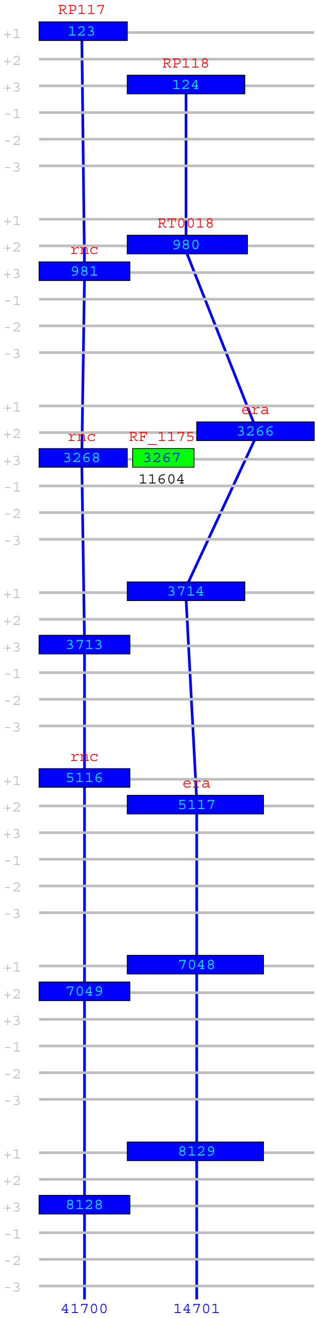

1 Rickettsia prowazekii str. Madrid E, complete genome
2 Rickettsia typhi str. wilmington, complete genome
3 Rickettsia felis URRWXCal2, complete genome
4 Rickettsia akari str. Hartford chromosome, whole genome shotgun sequence
5 Rickettsia conorii str. Malish 7, complete genome
6 Rickettsia sibirica 246 rsib_agncrt, whole genome shotgun sequence
7 Rickettsia rickettsii chromosome, whole genome shotgun sequence

Reg_id: 437

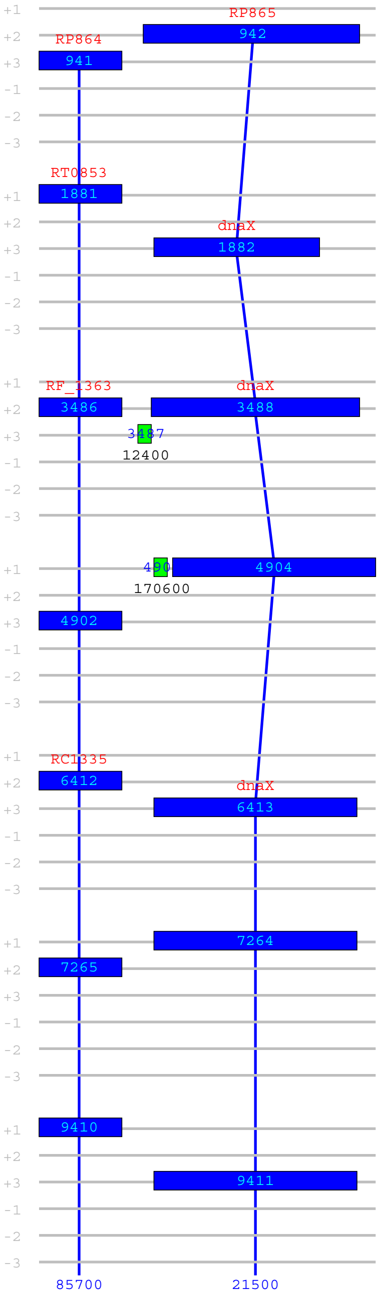

1 Rickettsia prowazekii str. Madrid E, complete genome
2 Rickettsia typhi str. wilmington, complete genome
3 Rickettsia felis URRWXCal2, complete genome
4 Rickettsia akari str. Hartford chromosome, whole genome shotgun sequence
5 Rickettsia conorii str. Malish 7, complete genome
6 Rickettsia sibirica 246 rsib_agncrt, whole genome shotgun sequence
7 Rickettsia rickettsii chromosome, whole genome shotgun sequence

Reg_id: 439

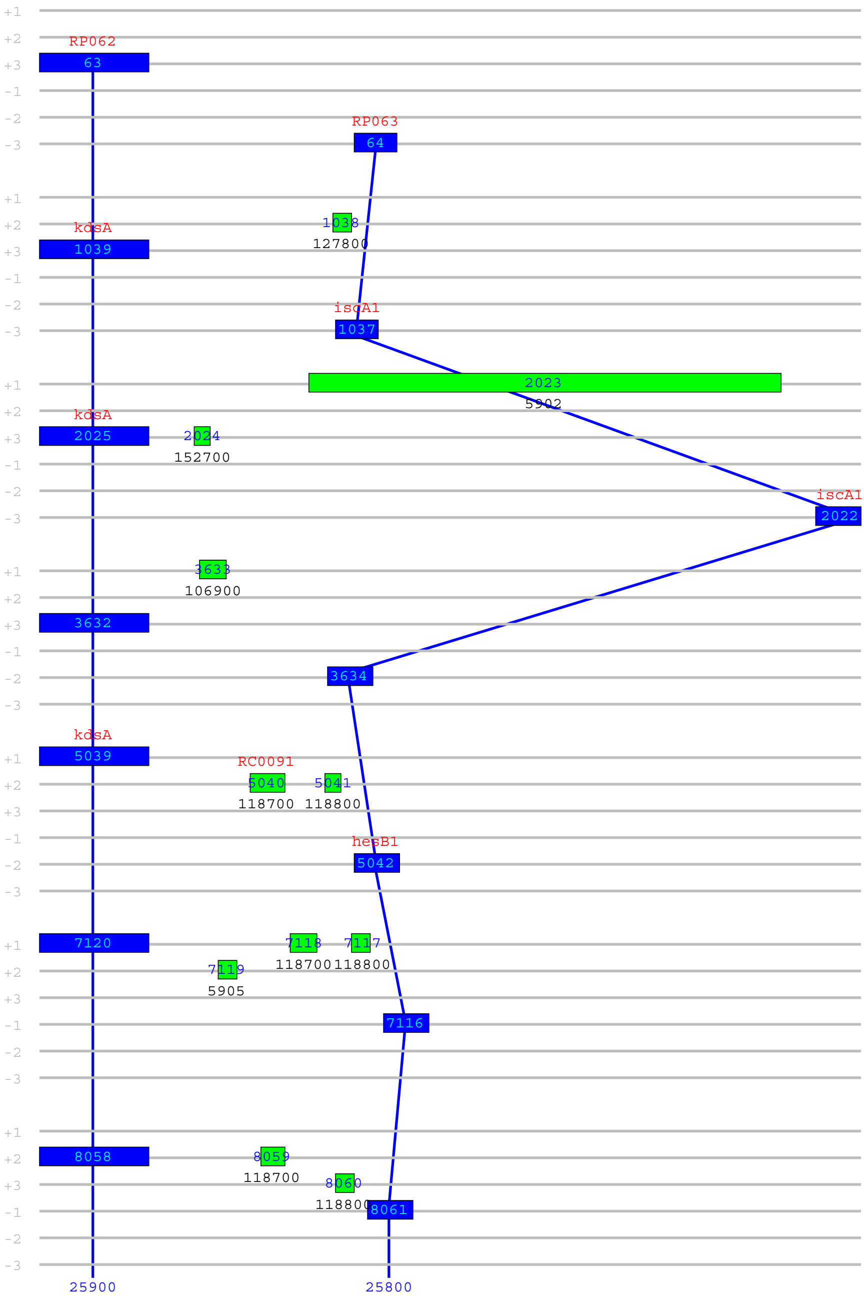

1 Rickettsia prowazekii str. Madrid E, complete genome
2 Rickettsia typhi str. wilmington, complete genome
3 Rickettsia felis URRWXCal2, complete genome
4 Rickettsia akari str. Hartford chromosome, whole genome shotgun sequence
5 Rickettsia conorii str. Malish 7, complete genome
6 Rickettsia sibirica 246 rsib_agncrt, whole genome shotgun sequence
7 Rickettsia rickettsii chromosome, whole genome shotgun sequence

Reg_id: 440

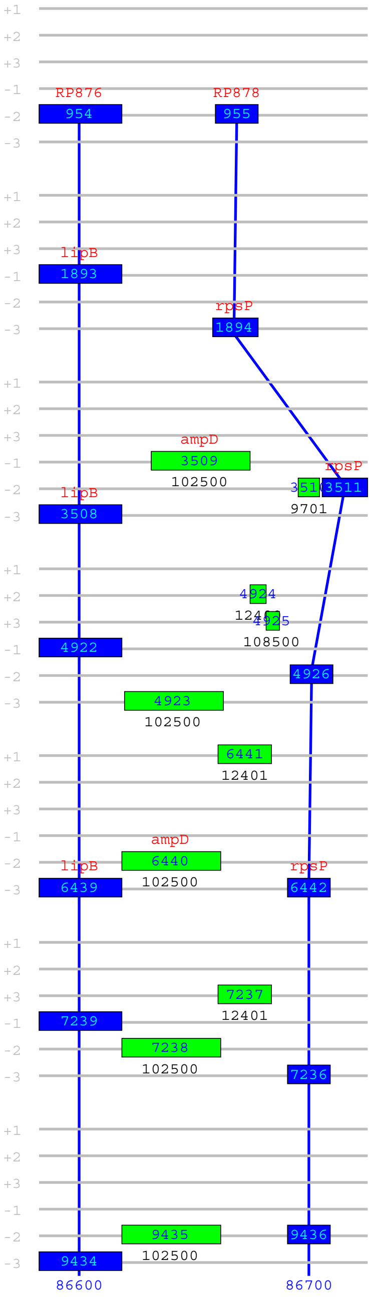

1 Rickettsia prowazekii str. Madrid E, complete genome
2 Rickettsia typhi str. wilmington, complete genome
3 Rickettsia felis URRWXCal2, complete genome
4 Rickettsia akari str. Hartford chromosome, whole genome shotgun sequence
5 Rickettsia conorii str. Malish 7, complete genome
6 Rickettsia sibirica 246 rsib_agncrt, whole genome shotgun sequence
7 Rickettsia rickettsii chromosome, whole genome shotgun sequence

Reg_id: 442

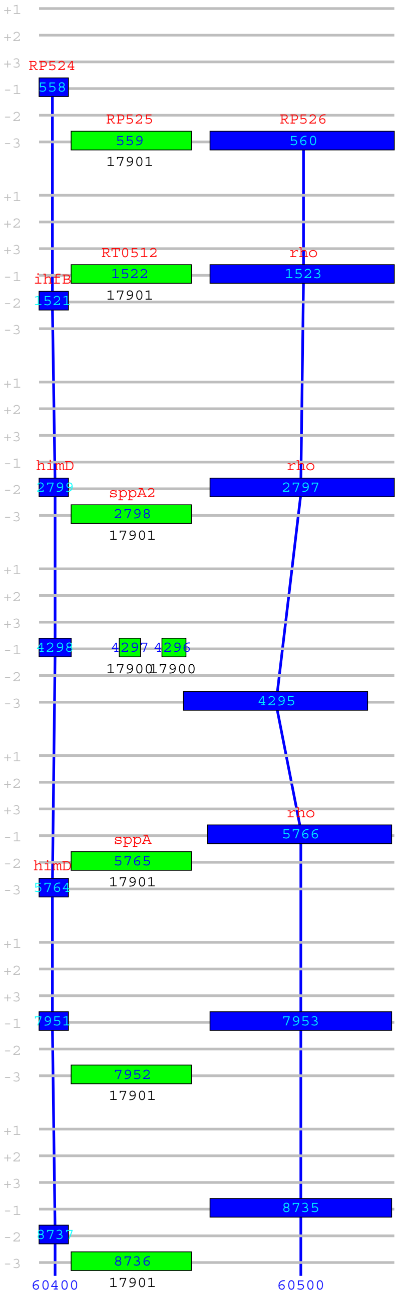

1 Rickettsia prowazekii str. Madrid E, complete genome
2 Rickettsia typhi str. wilmington, complete genome
3 Rickettsia felis URRWXCal2, complete genome
4 Rickettsia akari str. Hartford chromosome, whole genome shotgun sequence
5 Rickettsia conorii str. Malish 7, complete genome
6 Rickettsia sibirica 246 rsib_agncrt, whole genome shotgun sequence
7 Rickettsia rickettsii chromosome, whole genome shotgun sequence

Reg_id: 443

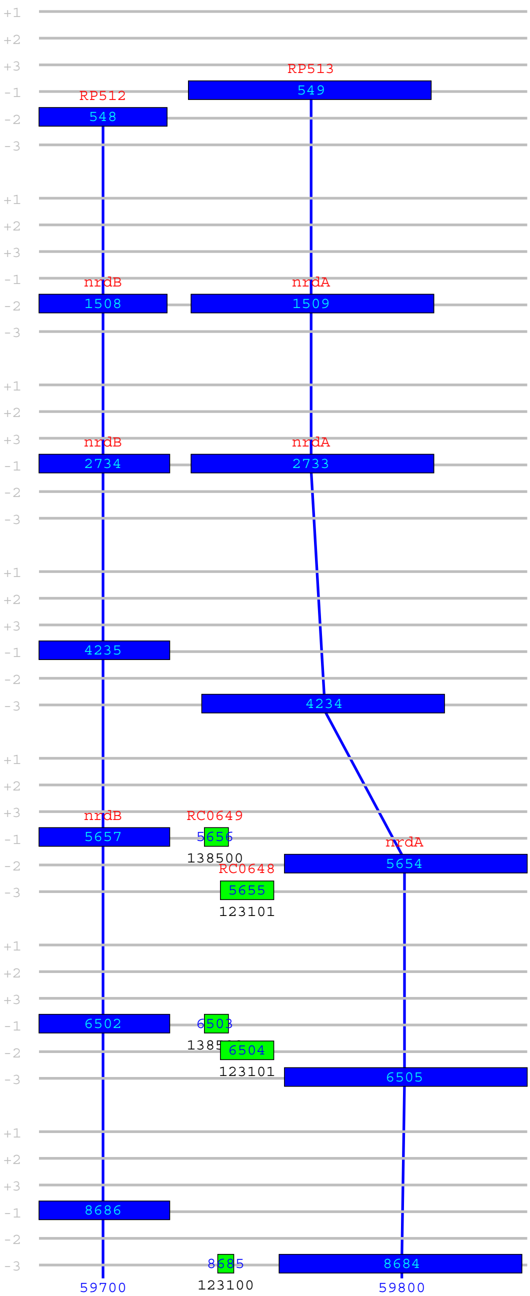

1 Rickettsia prowazekii str. Madrid E, complete genome
2 Rickettsia typhi str. wilmington, complete genome
3 Rickettsia felis URRWXCal2, complete genome
4 Rickettsia akari str. Hartford chromosome, whole genome shotgun sequence
5 Rickettsia conorii str. Malish 7, complete genome
6 Rickettsia sibirica 246 rsib_agncrt, whole genome shotgun sequence
7 Rickettsia rickettsii chromosome, whole genome shotgun sequence

Reg_id: 447

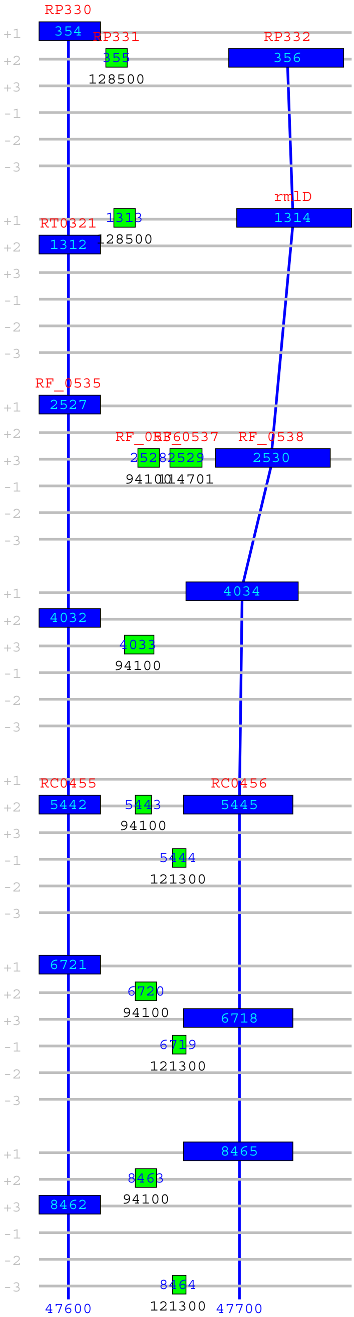

1 Rickettsia prowazekii str. Madrid E, complete genome
2 Rickettsia typhi str. wilmington, complete genome
3 Rickettsia felis URRWXCal2, complete genome
4 Rickettsia akari str. Hartford chromosome, whole genome shotgun sequence
5 Rickettsia conorii str. Malish 7, complete genome
6 Rickettsia sibirica 246 rsib_agncrt, whole genome shotgun sequence
7 Rickettsia rickettsii chromosome, whole genome shotgun sequence

Reg_id: 448

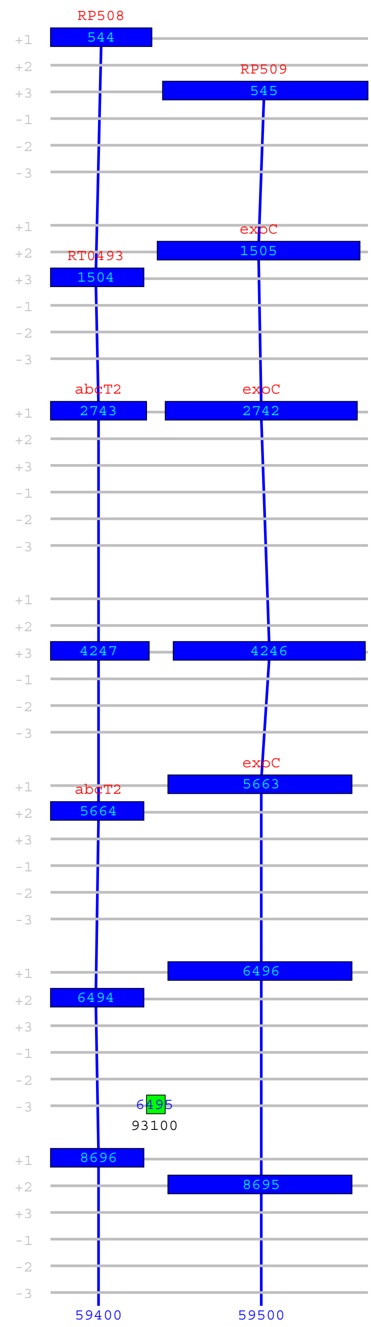

1 Rickettsia prowazekii str. Madrid E, complete genome
2 Rickettsia typhi str. wilmington, complete genome
3 Rickettsia felis URRWXCal2, complete genome
4 Rickettsia akari str. Hartford chromosome, whole genome shotgun sequence
5 Rickettsia conorii str. Malish 7, complete genome
6 Rickettsia sibirica 246 rsib_agncrt, whole genome shotgun sequence
7 Rickettsia rickettsii chromosome, whole genome shotgun sequence

Reg_id: 451

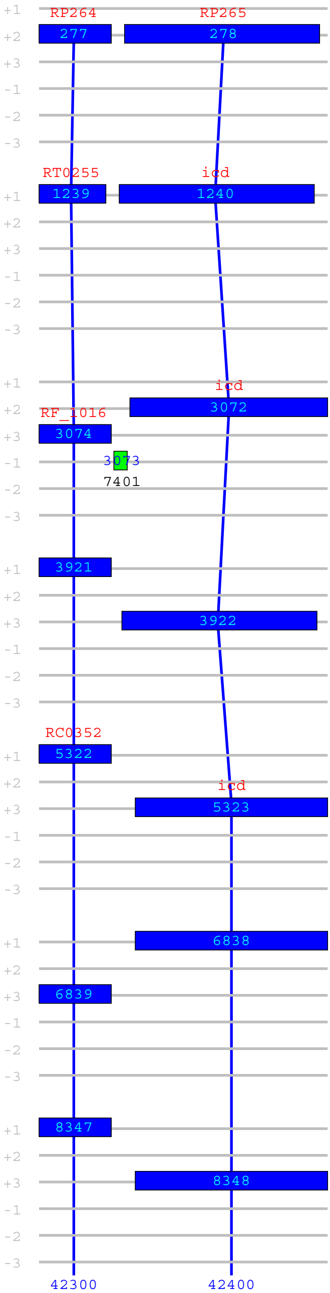

1 Rickettsia prowazekii str. Madrid E, complete genome
2 Rickettsia typhi str. wilmington, complete genome
3 Rickettsia felis URRWXCal2, complete genome
4 Rickettsia akari str. Hartford chromosome, whole genome shotgun sequence
5 Rickettsia conorii str. Malish 7, complete genome
6 Rickettsia sibirica 246 rsib_agncrt, whole genome shotgun sequence
7 Rickettsia rickettsii chromosome, whole genome shotgun sequence

Reg_id: 452

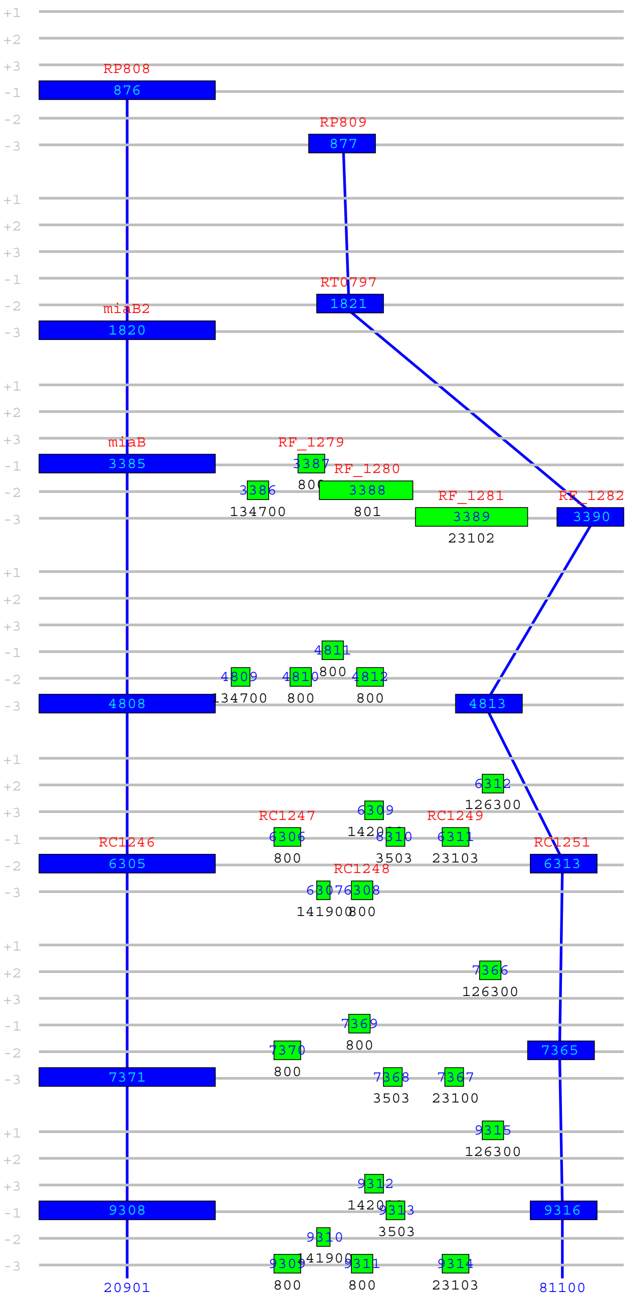

1 Rickettsia prowazekii str. Madrid E, complete genome
2 Rickettsia typhi str. wilmington, complete genome
3 Rickettsia felis URRWXCal2, complete genome
4 Rickettsia akari str. Hartford chromosome, whole genome shotgun sequence
5 Rickettsia conorii str. Malish 7, complete genome
6 Rickettsia sibirica 246 rsib_agncrt, whole genome shotgun sequence
7 Rickettsia rickettsii chromosome, whole genome shotgun sequence

Reg_id: 453

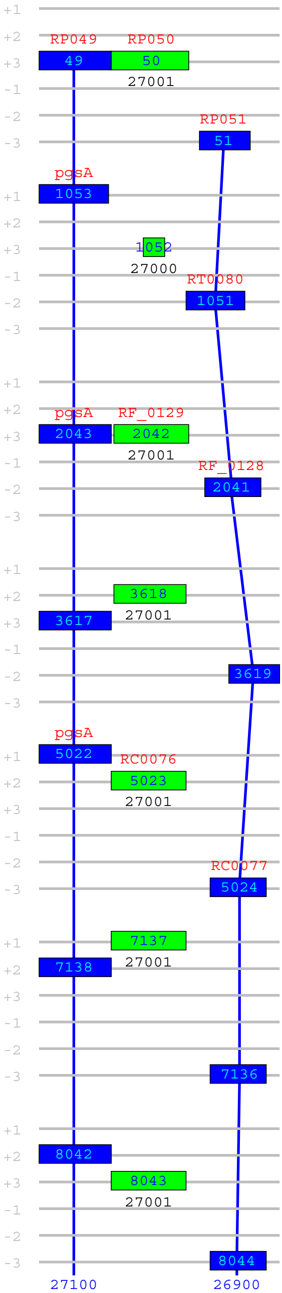

1 Rickettsia prowazekii str. Madrid E, complete genome
2 Rickettsia typhi str. wilmington, complete genome
3 Rickettsia felis URRWXCal2, complete genome
4 Rickettsia akari str. Hartford chromosome, whole genome shotgun sequence
5 Rickettsia conorii str. Malish 7, complete genome
6 Rickettsia sibirica 246 rsib_agncrt, whole genome shotgun sequence
7 Rickettsia rickettsii chromosome, whole genome shotgun sequence

Reg_id: 454

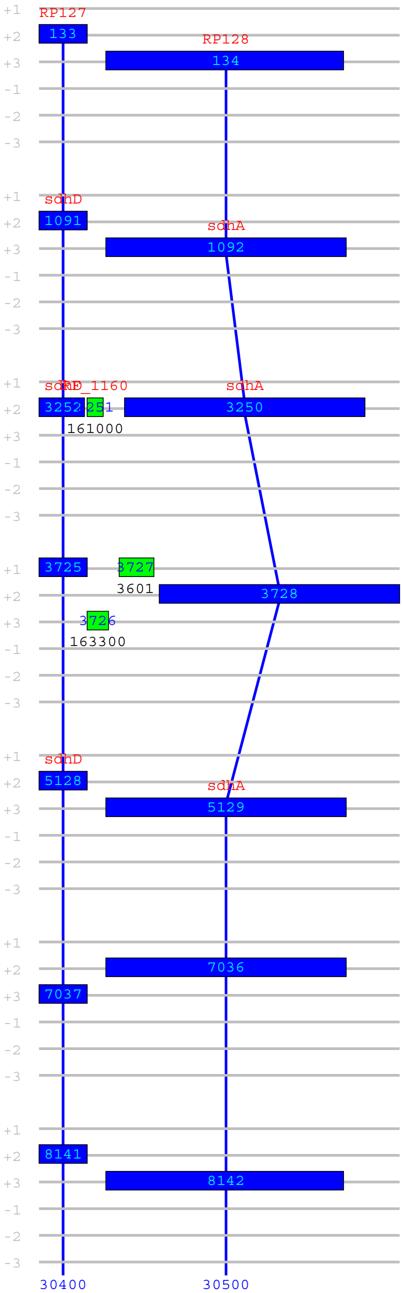

1 Rickettsia prowazekii str. Madrid E, complete genome
2 Rickettsia typhi str. wilmington, complete genome
3 Rickettsia felis URRWXCal2, complete genome
4 Rickettsia akari str. Hartford chromosome, whole genome shotgun sequence
5 Rickettsia conorii str. Malish 7, complete genome
6 Rickettsia sibirica 246 rsib_agncrt, whole genome shotgun sequence
7 Rickettsia rickettsii chromosome, whole genome shotgun sequence

Reg_id: 457

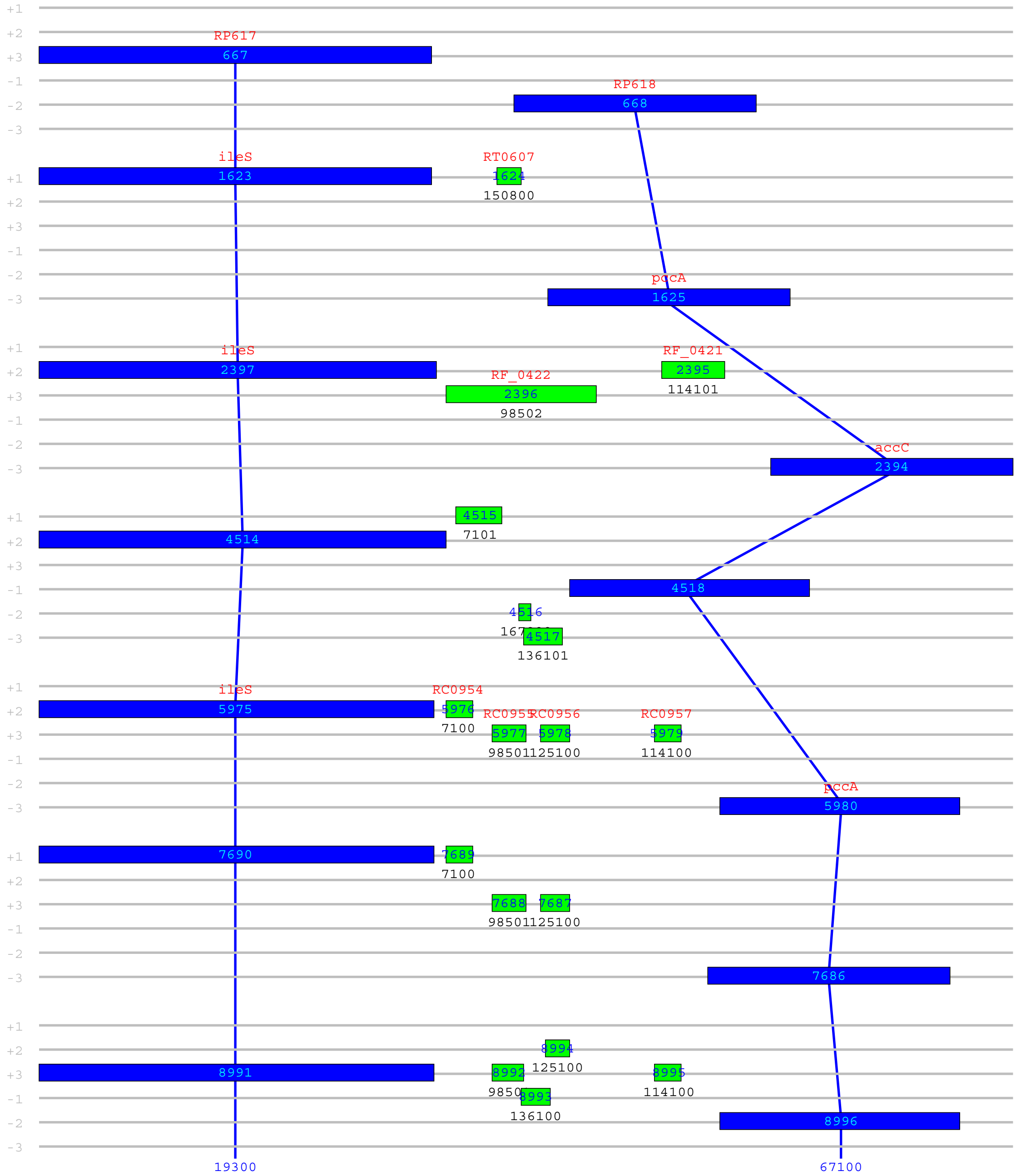

1 Rickettsia prowazekii str. Madrid E, complete genome
2 Rickettsia typhi str. wilmington, complete genome
3 Rickettsia felis URRWXCal2, complete genome
4 Rickettsia akari str. Hartford chromosome, whole genome shotgun sequence
5 Rickettsia conorii str. Malish 7, complete genome
6 Rickettsia sibirica 246 rsib_agncrt, whole genome shotgun sequence
7 Rickettsia rickettsii chromosome, whole genome shotgun sequence

Reg_id: 460

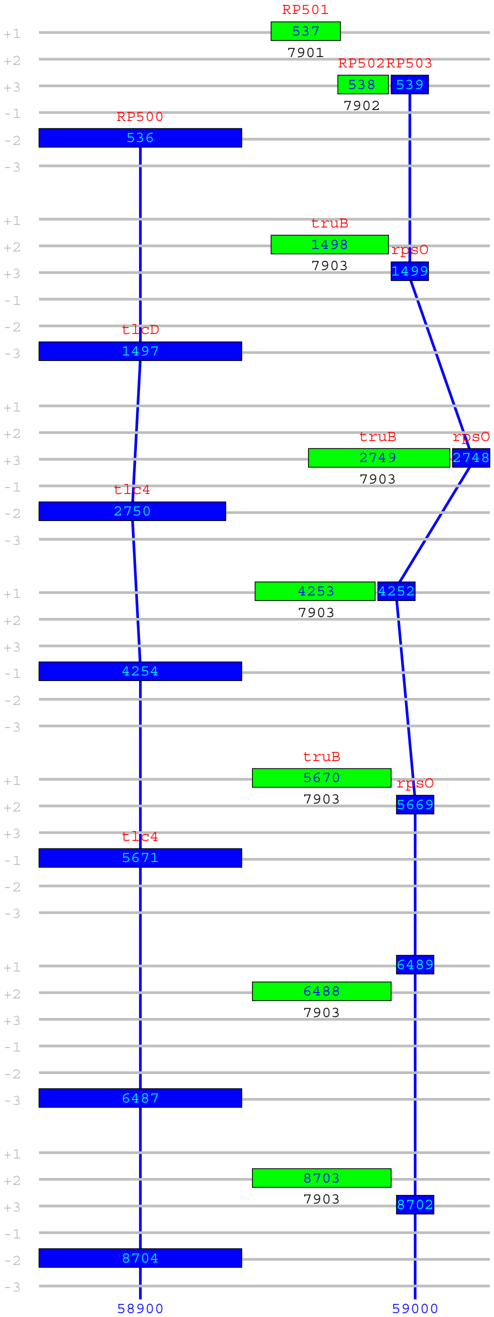

1 Rickettsia prowazekii str. Madrid E, complete genome
2 Rickettsia typhi str. wilmington, complete genome
3 Rickettsia felis URRWXCal2, complete genome
4 Rickettsia akari str. Hartford chromosome, whole genome shotgun sequence
5 Rickettsia conorii str. Malish 7, complete genome
6 Rickettsia sibirica 246 rsib_agncrt, whole genome shotgun sequence
7 Rickettsia rickettsii chromosome, whole genome shotgun sequence

Reg_id: 461

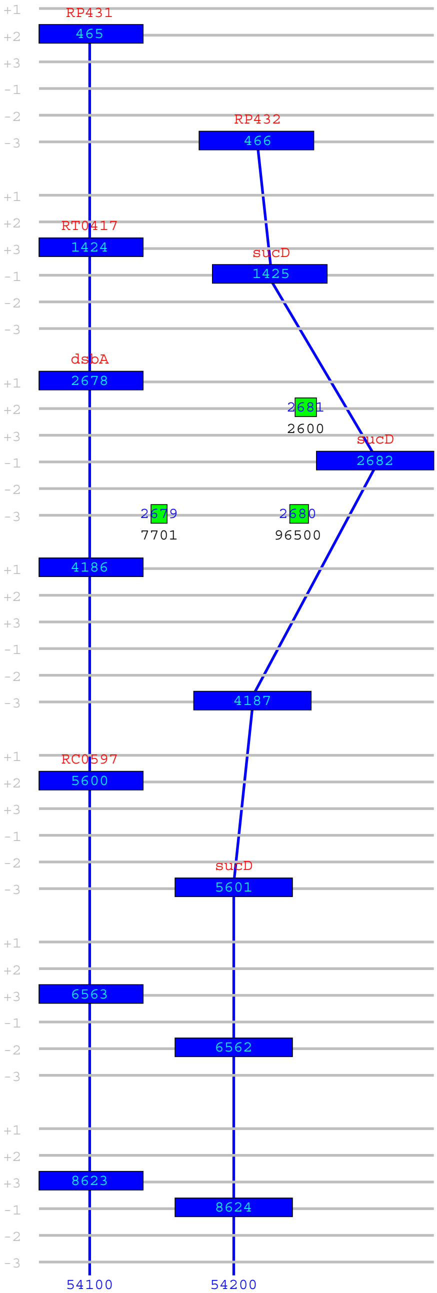

1 Rickettsia prowazekii str. Madrid E, complete genome
2 Rickettsia typhi str. wilmington, complete genome
3 Rickettsia felis URRWXCal2, complete genome
4 Rickettsia akari str. Hartford chromosome, whole genome shotgun sequence
5 Rickettsia conorii str. Malish 7, complete genome
6 Rickettsia sibirica 246 rsib_agncrt, whole genome shotgun sequence
7 Rickettsia rickettsii chromosome, whole genome shotgun sequence

Reg_id: 464

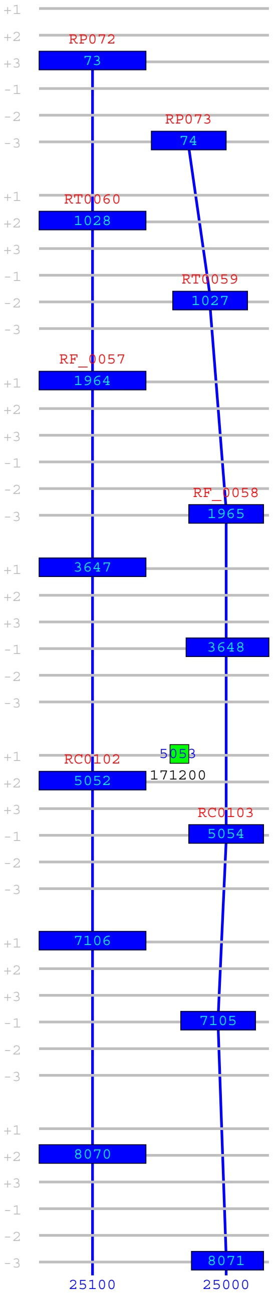

1 Rickettsia prowazekii str. Madrid E, complete genome
2 Rickettsia typhi str. wilmington, complete genome
3 Rickettsia felis URRWXCal2, complete genome
4 Rickettsia akari str. Hartford chromosome, whole genome shotgun sequence
5 Rickettsia conorii str. Malish 7, complete genome
6 Rickettsia sibirica 246 rsib_agncrt, whole genome shotgun sequence
7 Rickettsia rickettsii chromosome, whole genome shotgun sequence

Reg_id: 465

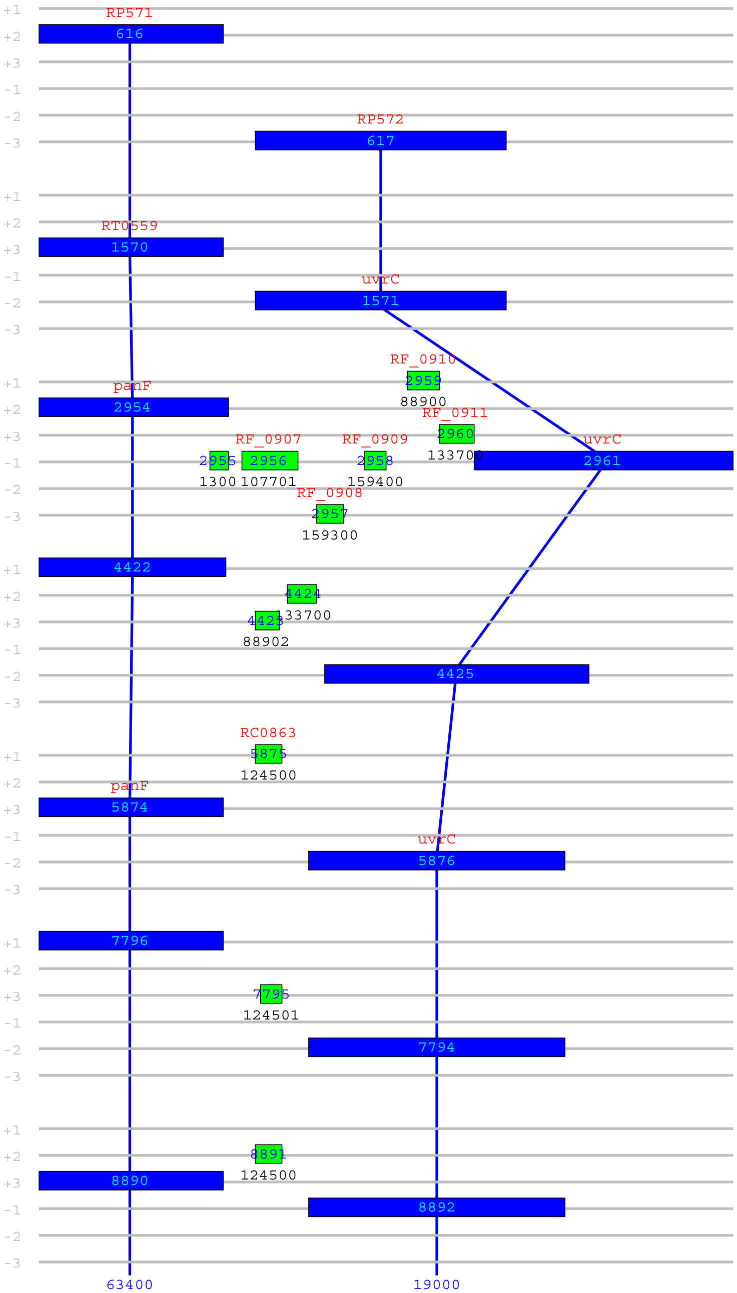

1 Rickettsia prowazekii str. Madrid E, complete genome
2 Rickettsia typhi str. wilmington, complete genome
3 Rickettsia felis URRWXCal2, complete genome
4 Rickettsia akari str. Hartford chromosome, whole genome shotgun sequence
5 Rickettsia conorii str. Malish 7, complete genome
6 Rickettsia sibirica 246 rsib_agncrt, whole genome shotgun sequence
7 Rickettsia rickettsii chromosome, whole genome shotgun sequence

Reg_id: 472

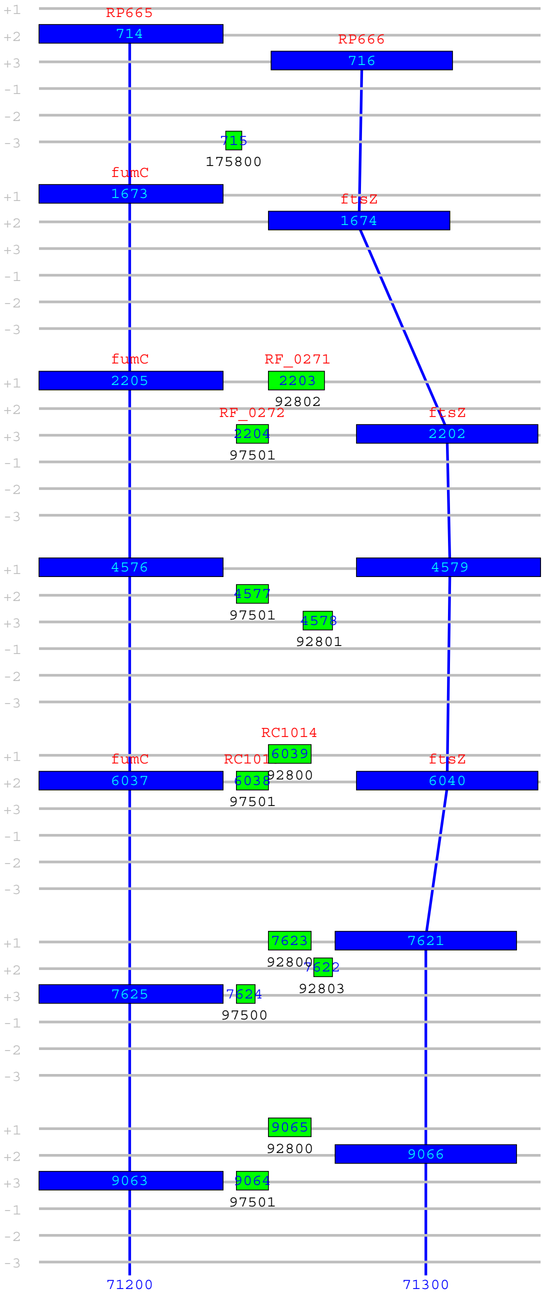

1 Rickettsia prowazekii str. Madrid E, complete genome
2 Rickettsia typhi str. wilmington, complete genome
3 Rickettsia felis URRWXCal2, complete genome
4 Rickettsia akari str. Hartford chromosome, whole genome shotgun sequence
5 Rickettsia conorii str. Malish 7, complete genome
6 Rickettsia sibirica 246 rsib_agncrt, whole genome shotgun sequence
7 Rickettsia rickettsii chromosome, whole genome shotgun sequence

Reg_id: 477

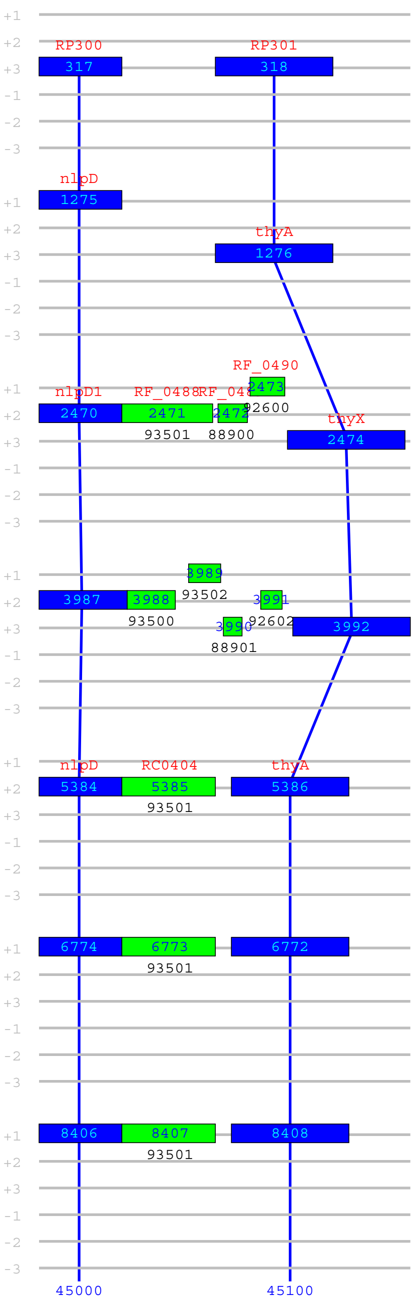

1 Rickettsia prowazekii str. Madrid E, complete genome
2 Rickettsia typhi str. wilmington, complete genome
3 Rickettsia felis URRWXCal2, complete genome
4 Rickettsia akari str. Hartford chromosome, whole genome shotgun sequence
5 Rickettsia conorii str. Malish 7, complete genome
6 Rickettsia sibirica 246 rsib_agncrt, whole genome shotgun sequence
7 Rickettsia rickettsii chromosome, whole genome shotgun sequence

Reg_id: 478

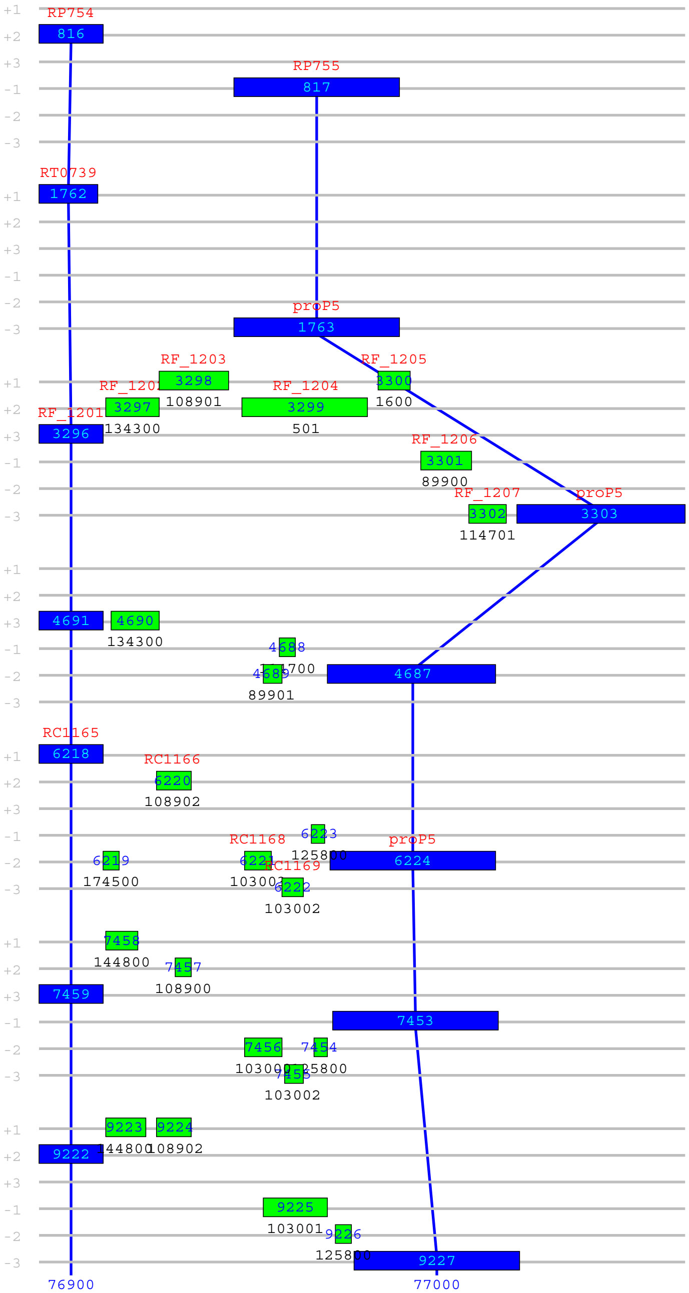

1 Rickettsia prowazekii str. Madrid E, complete genome
2 Rickettsia typhi str. wilmington, complete genome
3 Rickettsia felis URRWXCal2, complete genome
4 Rickettsia akari str. Hartford chromosome, whole genome shotgun sequence
5 Rickettsia conorii str. Malish 7, complete genome
6 Rickettsia sibirica 246 rsib_agncrt, whole genome shotgun sequence
7 Rickettsia rickettsii chromosome, whole genome shotgun sequence

Reg_id: 479

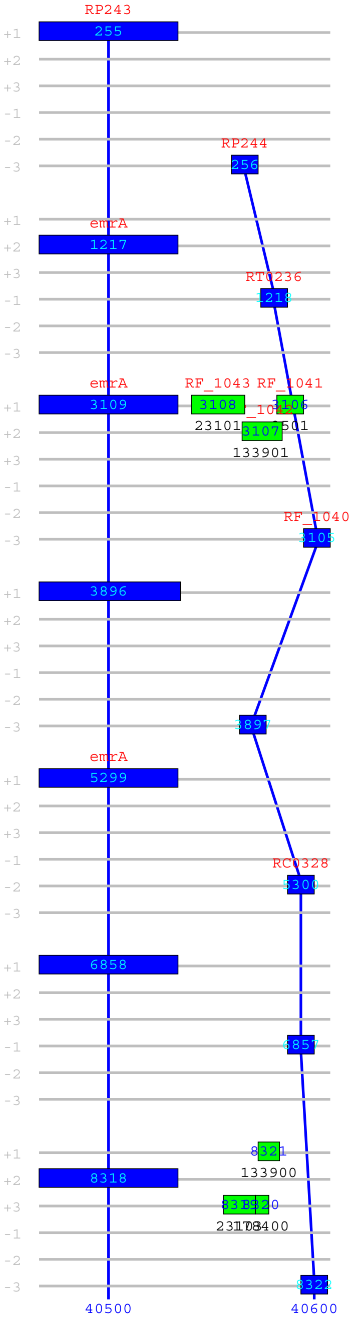

1 Rickettsia prowazekii str. Madrid E, complete genome
2 Rickettsia typhi str. wilmington, complete genome
3 Rickettsia felis URRWXCal2, complete genome
4 Rickettsia akari str. Hartford chromosome, whole genome shotgun sequence
5 Rickettsia conorii str. Malish 7, complete genome
6 Rickettsia sibirica 246 rsib_agncrt, whole genome shotgun sequence
7 Rickettsia rickettsii chromosome, whole genome shotgun sequence

Reg_id: 481

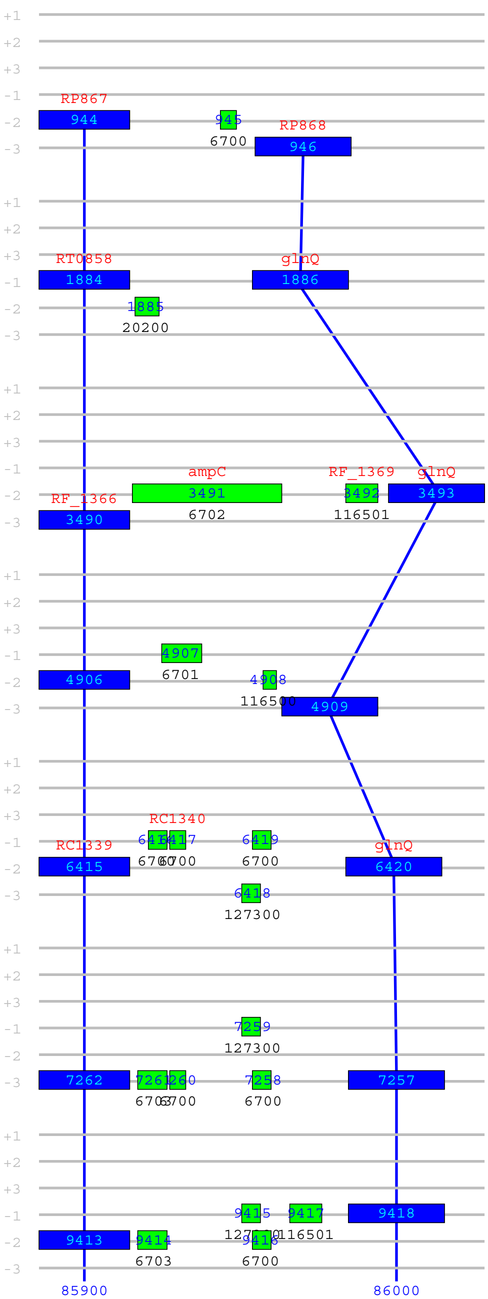

1 Rickettsia prowazekii str. Madrid E, complete genome
2 Rickettsia typhi str. wilmington, complete genome
3 Rickettsia felis URRWXCal2, complete genome
4 Rickettsia akari str. Hartford chromosome, whole genome shotgun sequence
5 Rickettsia conorii str. Malish 7, complete genome
6 Rickettsia sibirica 246 rsib_agncrt, whole genome shotgun sequence
7 Rickettsia rickettsii chromosome, whole genome shotgun sequence

Reg_id: 482

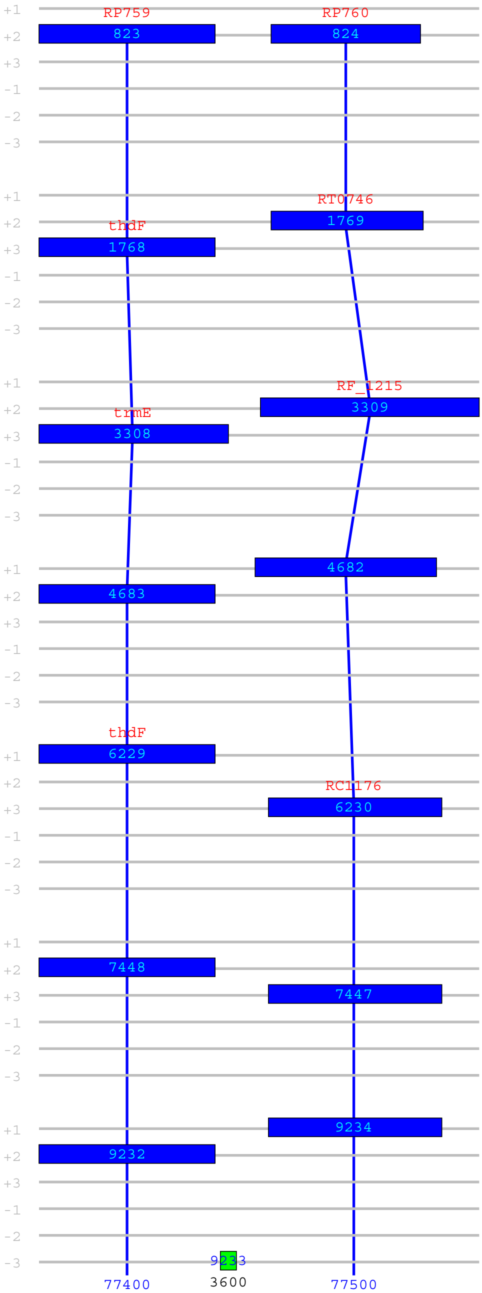

1 Rickettsia prowazekii str. Madrid E, complete genome
2 Rickettsia typhi str. wilmington, complete genome
3 Rickettsia felis URRWXCal2, complete genome
4 Rickettsia akari str. Hartford chromosome, whole genome shotgun sequence
5 Rickettsia conorii str. Malish 7, complete genome
6 Rickettsia sibirica 246 rsib_agncrt, whole genome shotgun sequence
7 Rickettsia rickettsii chromosome, whole genome shotgun sequence

Reg_id: 483

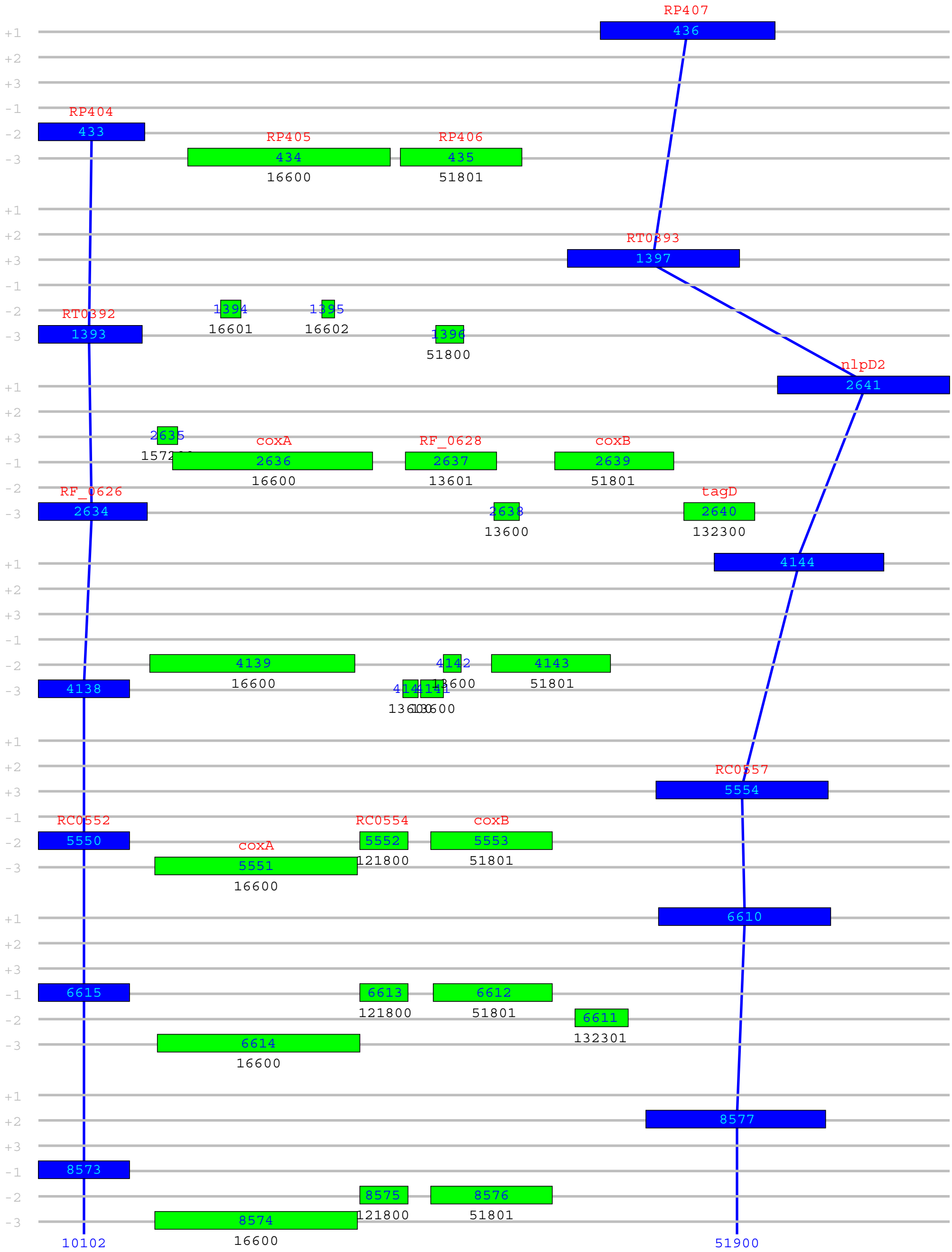

1 Rickettsia prowazekii str. Madrid E, complete genome
2 Rickettsia typhi str. wilmington, complete genome
3 Rickettsia felis URRWXCal2, complete genome
4 Rickettsia akari str. Hartford chromosome, whole genome shotgun sequence
5 Rickettsia conorii str. Malish 7, complete genome
6 Rickettsia sibirica 246 rsib_agncrt, whole genome shotgun sequence
7 Rickettsia rickettsii chromosome, whole genome shotgun sequence

Reg_id: 484

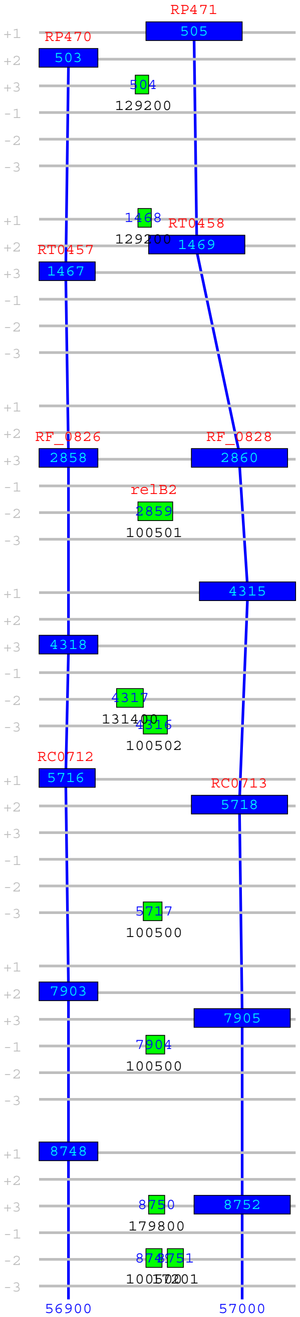

1 Rickettsia prowazekii str. Madrid E, complete genome
2 Rickettsia typhi str. wilmington, complete genome
3 Rickettsia felis URRWXCal2, complete genome
4 Rickettsia akari str. Hartford chromosome, whole genome shotgun sequence
5 Rickettsia conorii str. Malish 7, complete genome
6 Rickettsia sibirica 246 rsib_agncrt, whole genome shotgun sequence
7 Rickettsia rickettsii chromosome, whole genome shotgun sequence

Reg_id: 485

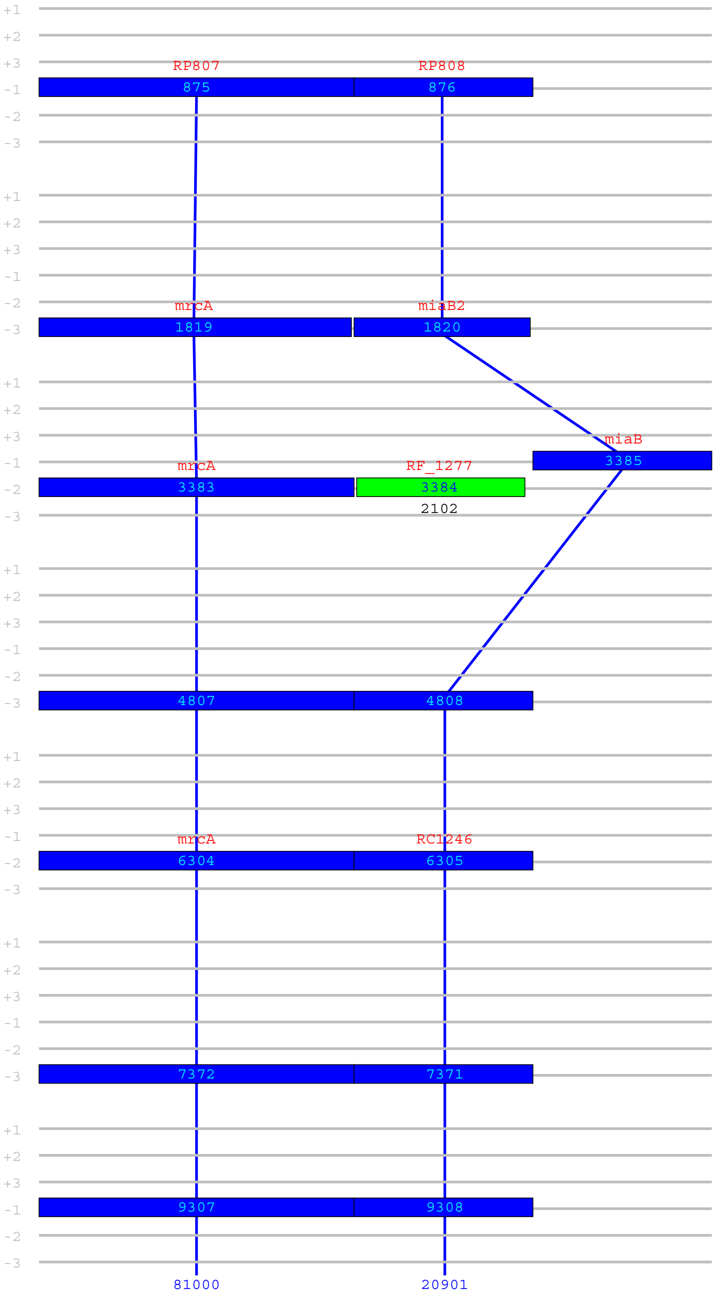

1 Rickettsia prowazekii str. Madrid E, complete genome
2 Rickettsia typhi str. wilmington, complete genome
3 Rickettsia felis URRWXCal2, complete genome
4 Rickettsia akari str. Hartford chromosome, whole genome shotgun sequence
5 Rickettsia conorii str. Malish 7, complete genome
6 Rickettsia sibirica 246 rsib_agncrt, whole genome shotgun sequence
7 Rickettsia rickettsii chromosome, whole genome shotgun sequence

Reg_id: 489

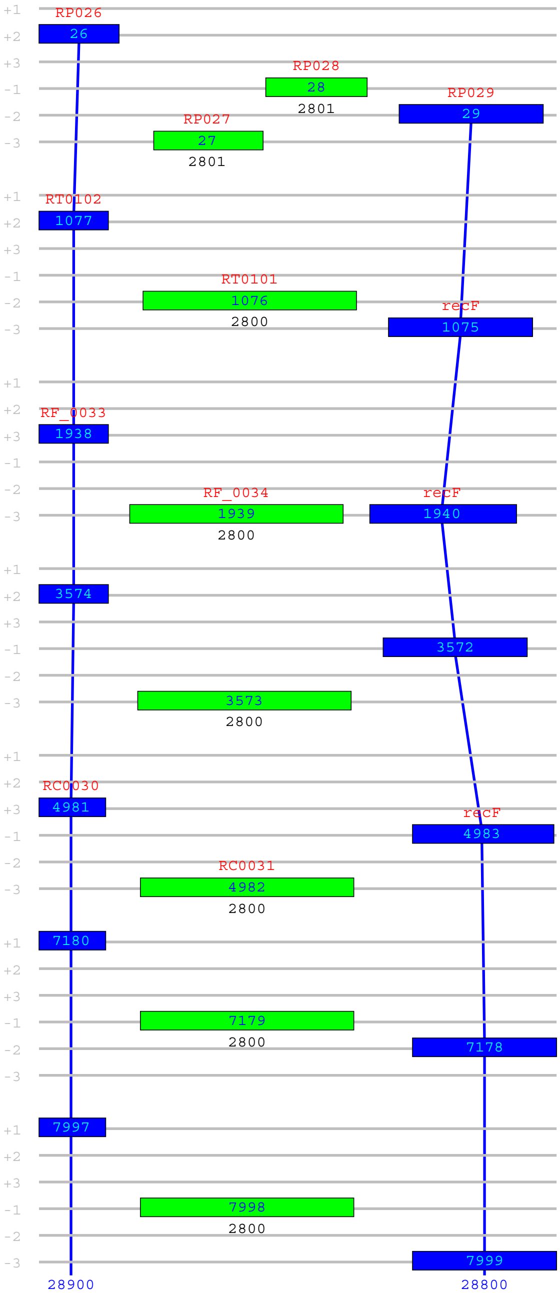

1 Rickettsia prowazekii str. Madrid E, complete genome
2 Rickettsia typhi str. wilmington, complete genome
3 Rickettsia felis URRWXCal2, complete genome
4 Rickettsia akari str. Hartford chromosome, whole genome shotgun sequence
5 Rickettsia conorii str. Malish 7, complete genome
6 Rickettsia sibirica 246 rsib_agncrt, whole genome shotgun sequence
7 Rickettsia rickettsii chromosome, whole genome shotgun sequence

Reg_id: 490

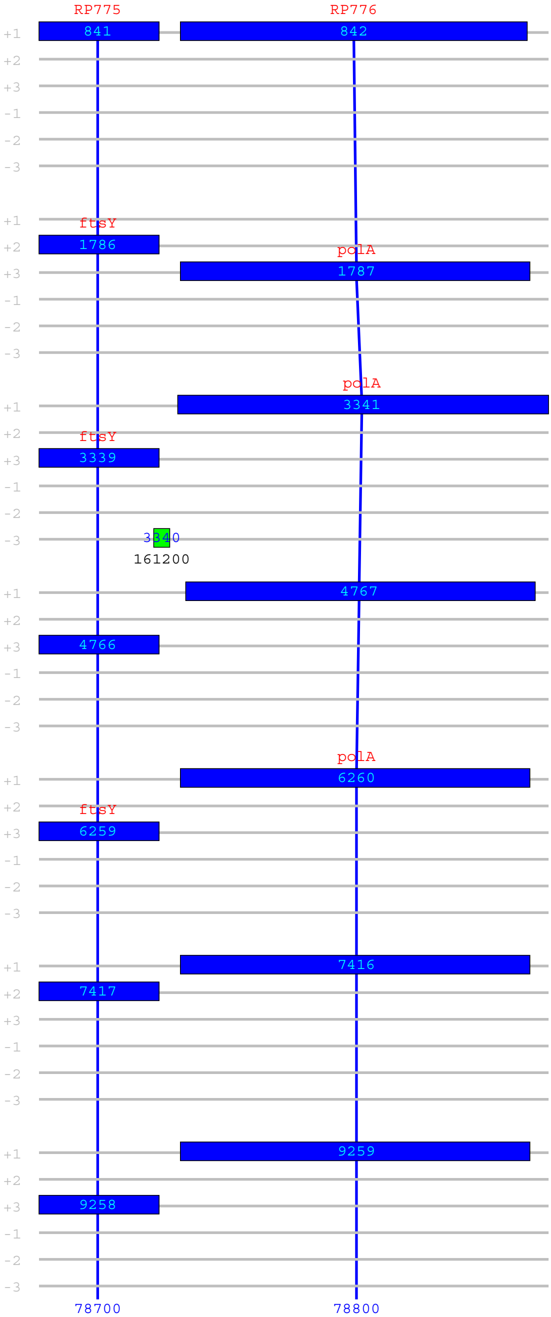

1 Rickettsia prowazekii str. Madrid E, complete genome
2 Rickettsia typhi str. wilmington, complete genome
3 Rickettsia felis URRWXCal2, complete genome
4 Rickettsia akari str. Hartford chromosome, whole genome shotgun sequence
5 Rickettsia conorii str. Malish 7, complete genome
6 Rickettsia sibirica 246 rsib_agncrt, whole genome shotgun sequence
7 Rickettsia rickettsii chromosome, whole genome shotgun sequence

Reg_id: 494

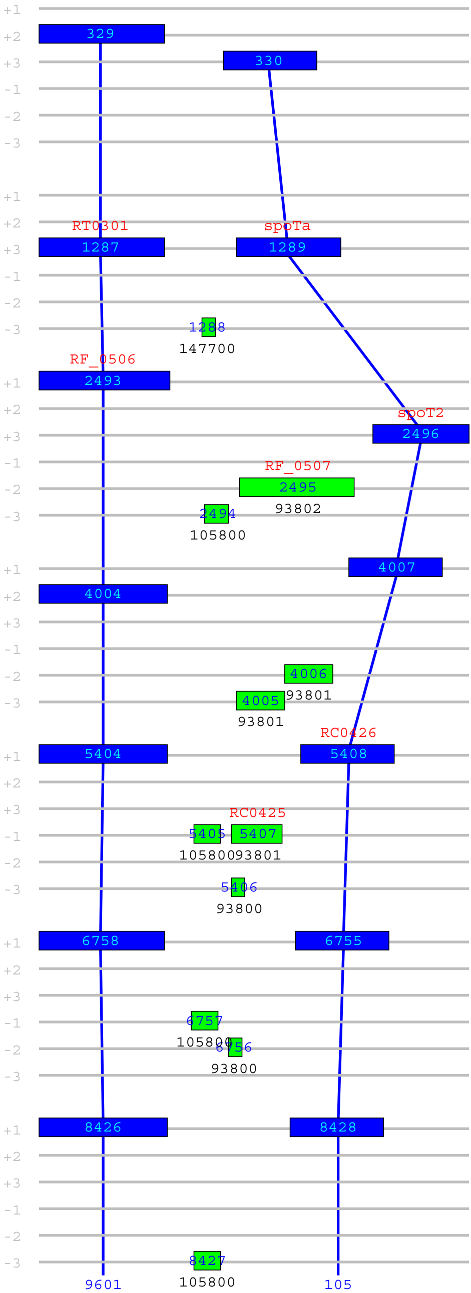

1 Rickettsia prowazekii str. Madrid E, complete genome
2 Rickettsia typhi str. wilmington, complete genome
3 Rickettsia felis URRWXCal2, complete genome
4 Rickettsia akari str. Hartford chromosome, whole genome shotgun sequence
5 Rickettsia conorii str. Malish 7, complete genome
6 Rickettsia sibirica 246 rsib_agncrt, whole genome shotgun sequence
7 Rickettsia rickettsii chromosome, whole genome shotgun sequence

Reg_id: 497

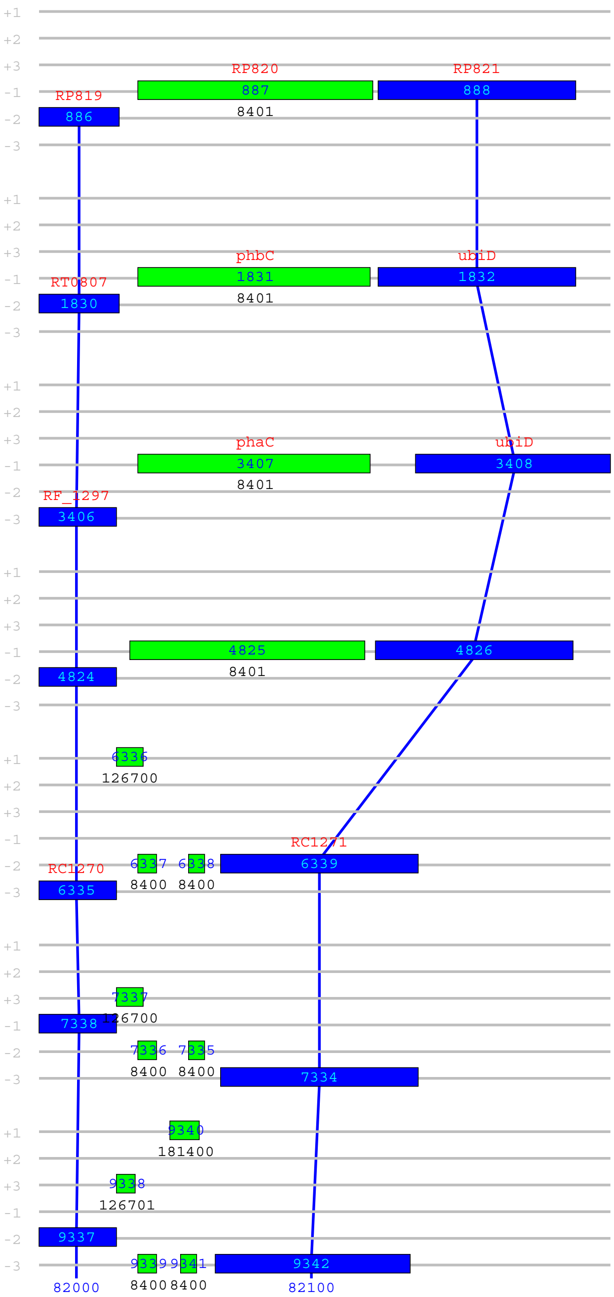

1 Rickettsia prowazekii str. Madrid E, complete genome
2 Rickettsia typhi str. wilmington, complete genome
3 Rickettsia felis URRWXCal2, complete genome
4 Rickettsia akari str. Hartford chromosome, whole genome shotgun sequence
5 Rickettsia conorii str. Malish 7, complete genome
6 Rickettsia sibirica 246 rsib_agncrt, whole genome shotgun sequence
7 Rickettsia rickettsii chromosome, whole genome shotgun sequence

Reg_id: 499

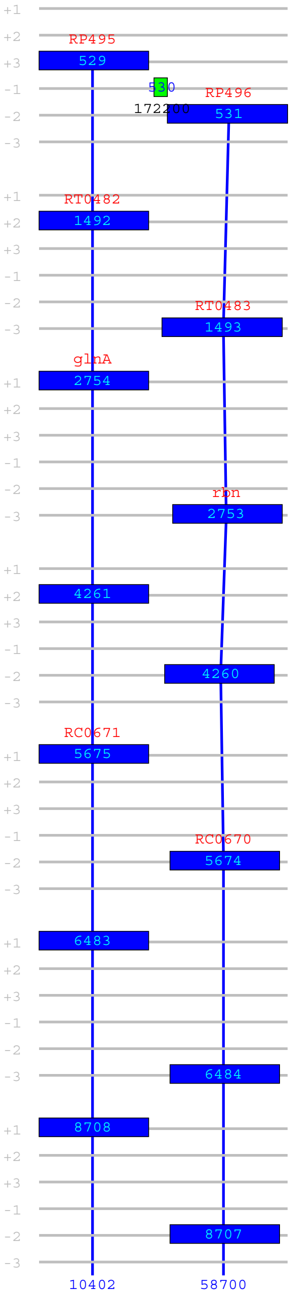

1 Rickettsia prowazekii str. Madrid E, complete genome
2 Rickettsia typhi str. wilmington, complete genome
3 Rickettsia felis URRWXCal2, complete genome
4 Rickettsia akari str. Hartford chromosome, whole genome shotgun sequence
5 Rickettsia conorii str. Malish 7, complete genome
6 Rickettsia sibirica 246 rsib_agncrt, whole genome shotgun sequence
7 Rickettsia rickettsii chromosome, whole genome shotgun sequence

Reg_id: 501

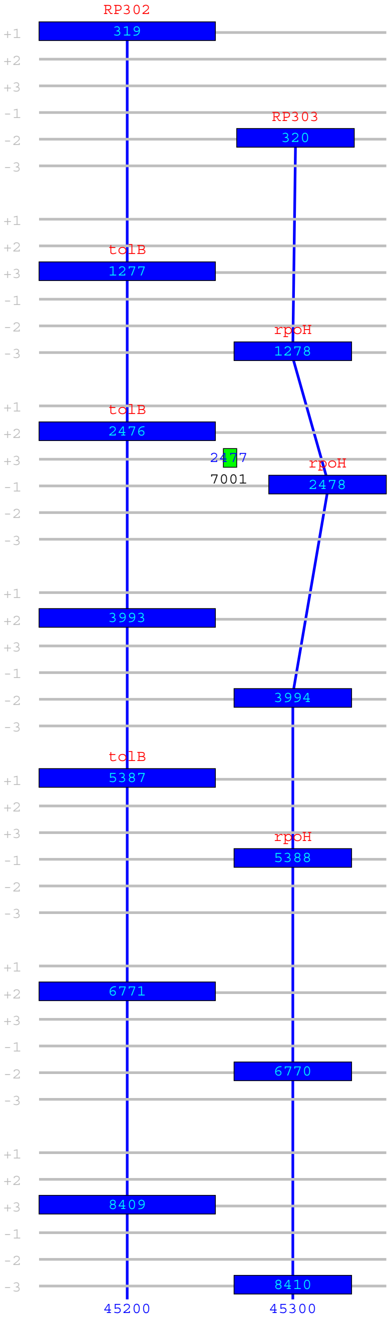

1 Rickettsia prowazekii str. Madrid E, complete genome
2 Rickettsia typhi str. wilmington, complete genome
3 Rickettsia felis URRWXCal2, complete genome
4 Rickettsia akari str. Hartford chromosome, whole genome shotgun sequence
5 Rickettsia conorii str. Malish 7, complete genome
6 Rickettsia sibirica 246 rsib_agncrt, whole genome shotgun sequence
7 Rickettsia rickettsii chromosome, whole genome shotgun sequence

Reg_id: 509

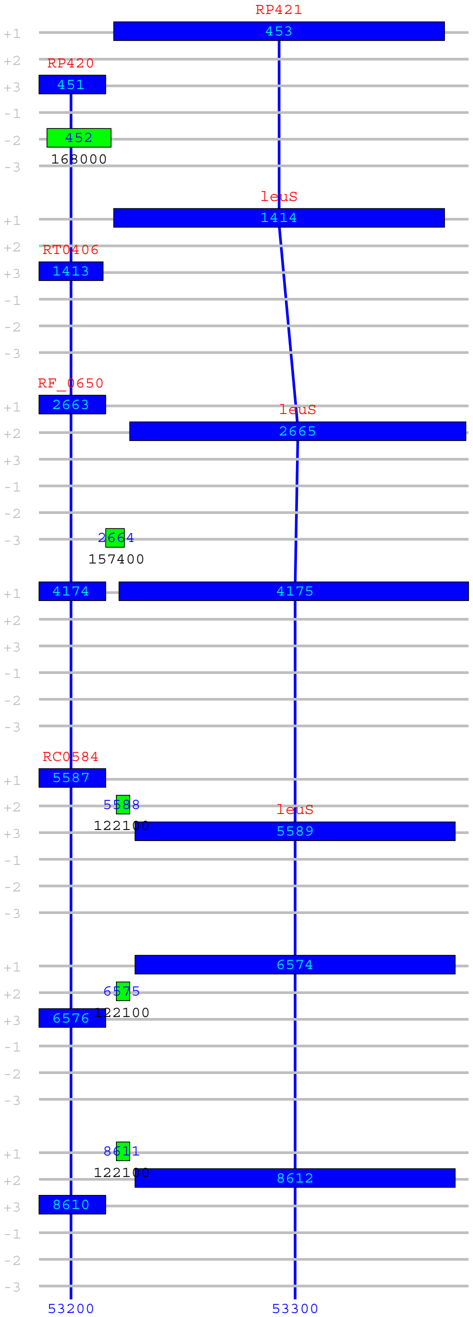

1 Rickettsia prowazekii str. Madrid E, complete genome
2 Rickettsia typhi str. wilmington, complete genome
3 Rickettsia felis URRWXCal2, complete genome
4 Rickettsia akari str. Hartford chromosome, whole genome shotgun sequence
5 Rickettsia conorii str. Malish 7, complete genome
6 Rickettsia sibirica 246 rsib_agncrt, whole genome shotgun sequence
7 Rickettsia rickettsii chromosome, whole genome shotgun sequence

Reg_id: 510

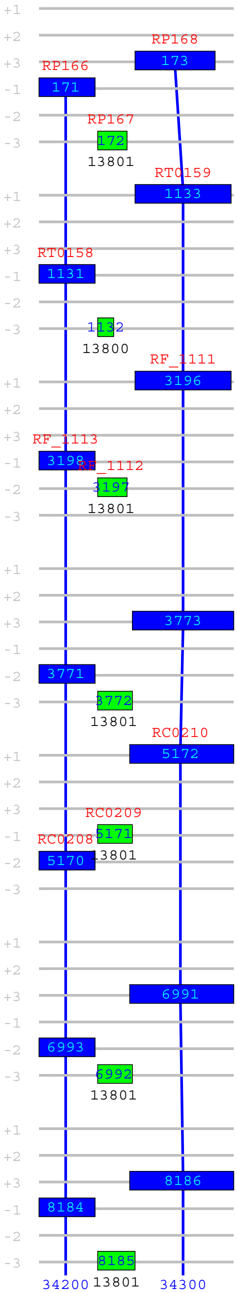

1 Rickettsia prowazekii str. Madrid E, complete genome
2 Rickettsia typhi str. wilmington, complete genome
3 Rickettsia felis URRWXCal2, complete genome
4 Rickettsia akari str. Hartford chromosome, whole genome shotgun sequence
5 Rickettsia conorii str. Malish 7, complete genome
6 Rickettsia sibirica 246 rsib_agncrt, whole genome shotgun sequence
7 Rickettsia rickettsii chromosome, whole genome shotgun sequence

Reg_id: 512

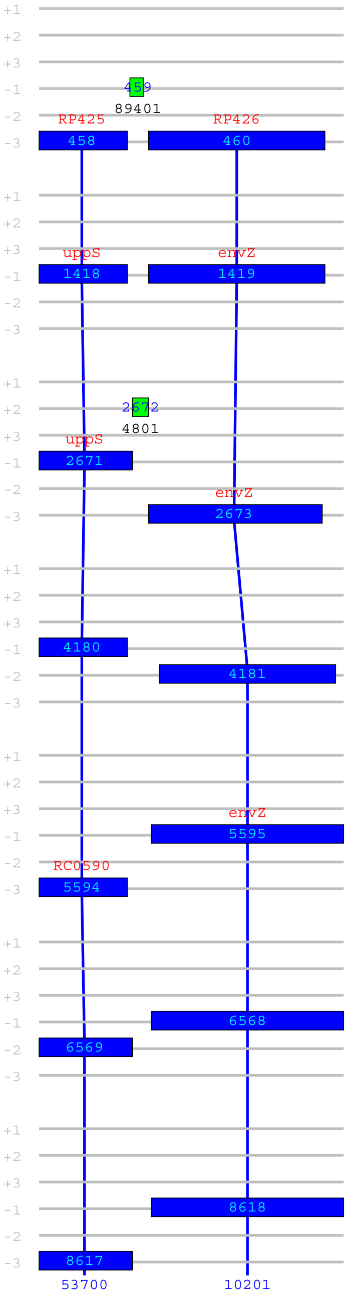

1 Rickettsia prowazekii str. Madrid E, complete genome
2 Rickettsia typhi str. wilmington, complete genome
3 Rickettsia felis URRWXCal2, complete genome
4 Rickettsia akari str. Hartford chromosome, whole genome shotgun sequence
5 Rickettsia conorii str. Malish 7, complete genome
6 Rickettsia sibirica 246 rsib_agncrt, whole genome shotgun sequence
7 Rickettsia rickettsii chromosome, whole genome shotgun sequence

Reg_id: 513

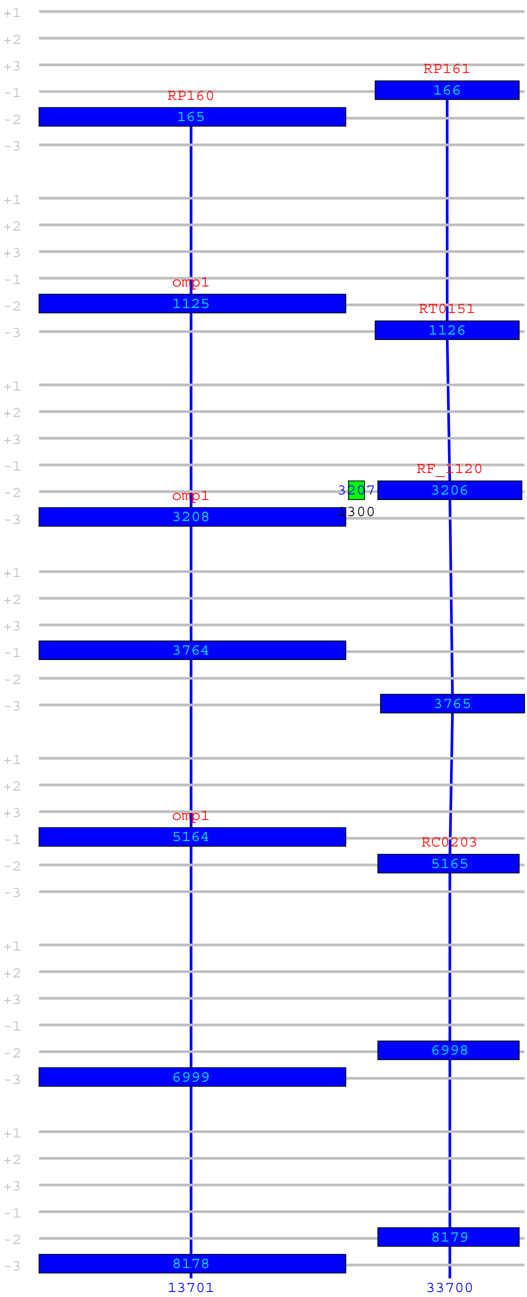

1 Rickettsia prowazekii str. Madrid E, complete genome
2 Rickettsia typhi str. wilmington, complete genome
3 Rickettsia felis URRWXCal2, complete genome
4 Rickettsia akari str. Hartford chromosome, whole genome shotgun sequence
5 Rickettsia conorii str. Malish 7, complete genome
6 Rickettsia sibirica 246 rsib_agncrt, whole genome shotgun sequence
7 Rickettsia rickettsii chromosome, whole genome shotgun sequence

Reg_id: 514

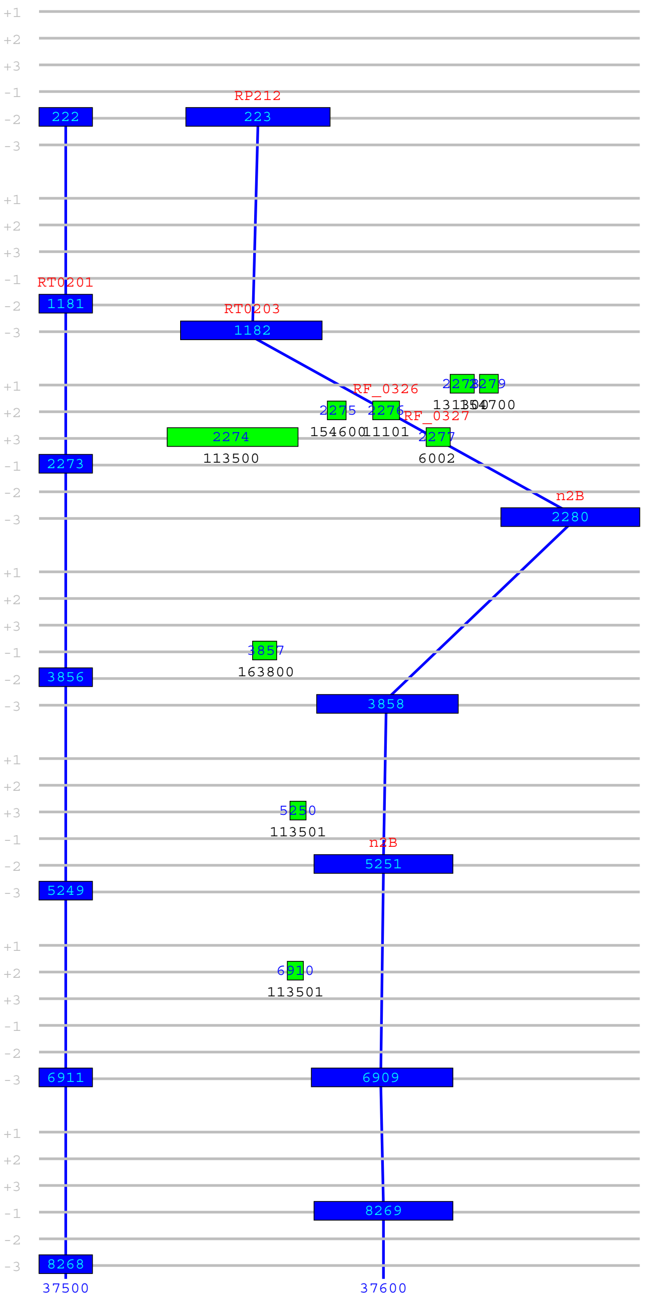

1 Rickettsia prowazekii str. Madrid E, complete genome
2 Rickettsia typhi str. wilmington, complete genome
3 Rickettsia felis URRWXCal2, complete genome
4 Rickettsia akari str. Hartford chromosome, whole genome shotgun sequence
5 Rickettsia conorii str. Malish 7, complete genome
6 Rickettsia sibirica 246 rsib_agncrt, whole genome shotgun sequence
7 Rickettsia rickettsii chromosome, whole genome shotgun sequence

Reg_id: 517

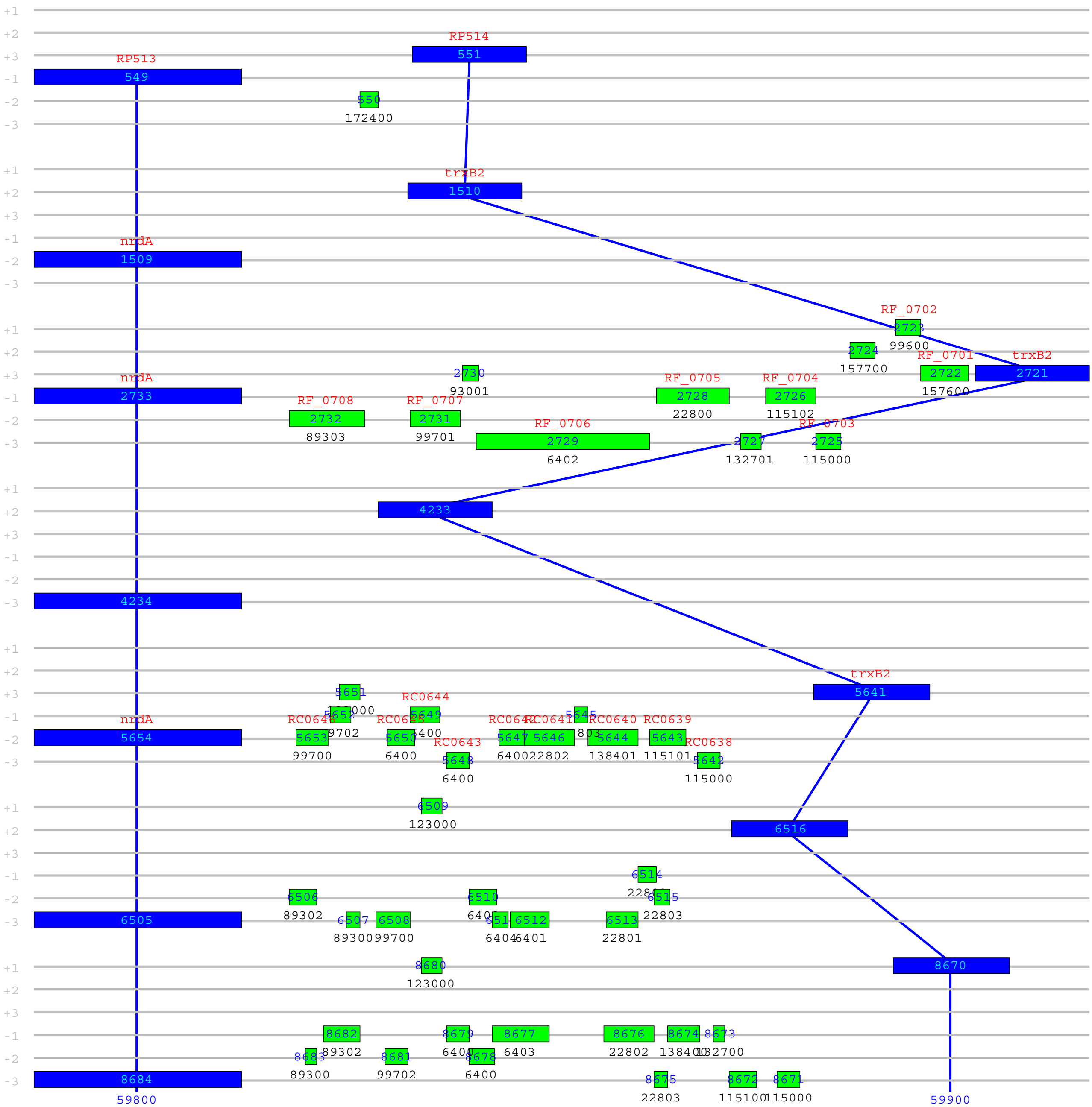

1 Rickettsia prowazekii str. Madrid E, complete genome
2 Rickettsia typhi str. wilmington, complete genome
3 Rickettsia felis URRWXCal2, complete genome
4 Rickettsia akari str. Hartford chromosome, whole genome shotgun sequence
5 Rickettsia conorii str. Malish 7, complete genome
6 Rickettsia sibirica 246 rsib_agncrt, whole genome shotgun sequence
7 Rickettsia rickettsii chromosome, whole genome shotgun sequence

Reg_id: 518

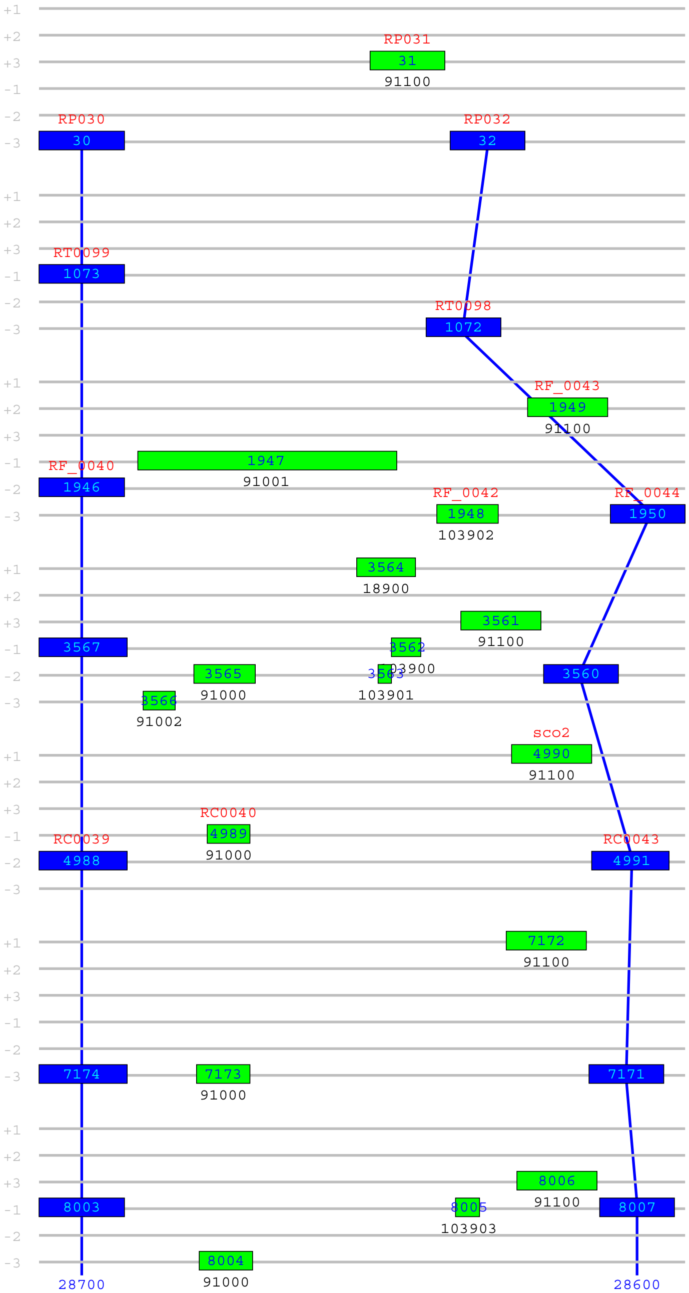

1 Rickettsia prowazekii str. Madrid E, complete genome
2 Rickettsia typhi str. wilmington, complete genome
3 Rickettsia felis URRWXCal2, complete genome
4 Rickettsia akari str. Hartford chromosome, whole genome shotgun sequence
5 Rickettsia conorii str. Malish 7, complete genome
6 Rickettsia sibirica 246 rsib_agncrt, whole genome shotgun sequence
7 Rickettsia rickettsii chromosome, whole genome shotgun sequence

Reg_id: 519

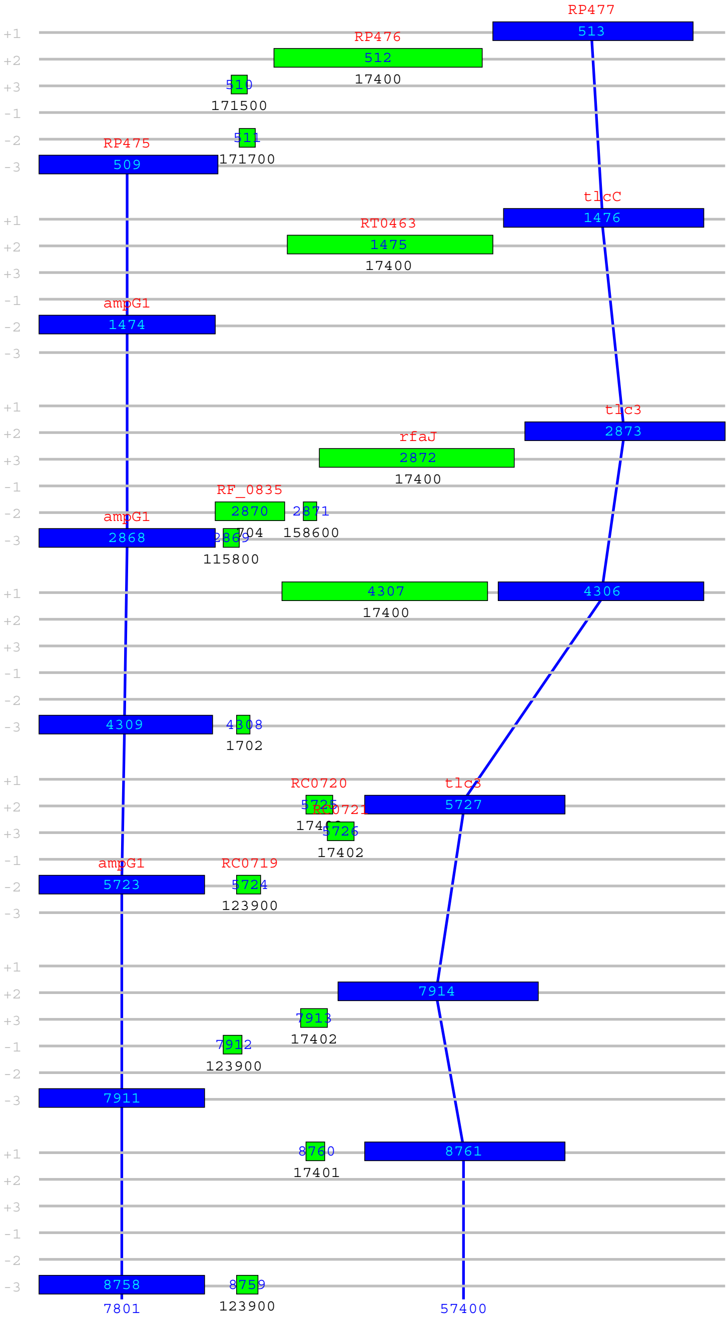

1 Rickettsia prowazekii str. Madrid E, complete genome
2 Rickettsia typhi str. wilmington, complete genome
3 Rickettsia felis URRWXCal2, complete genome
4 Rickettsia akari str. Hartford chromosome, whole genome shotgun sequence
5 Rickettsia conorii str. Malish 7, complete genome
6 Rickettsia sibirica 246 rsib_agncrt, whole genome shotgun sequence
7 Rickettsia rickettsii chromosome, whole genome shotgun sequence

Reg_id: 520

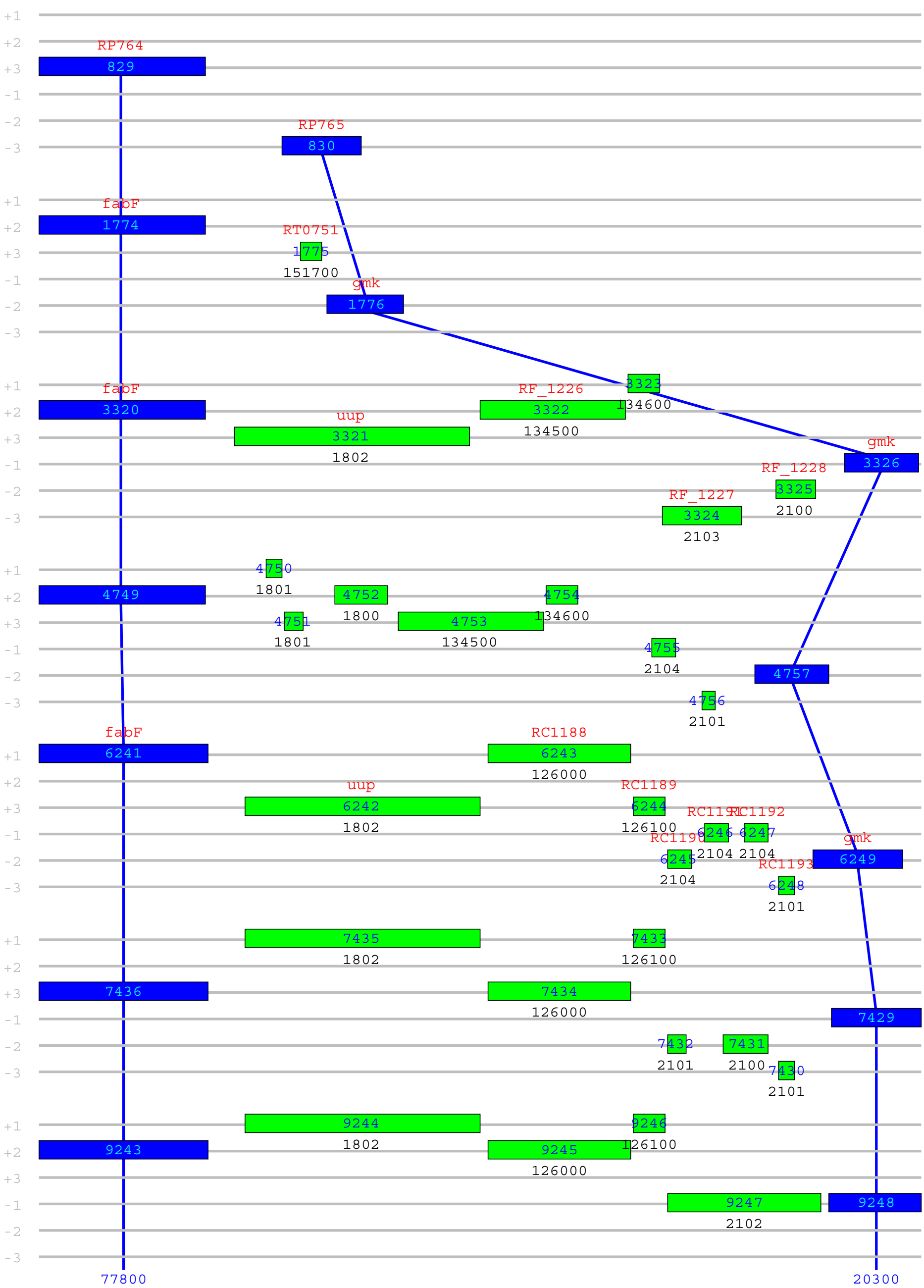

1 Rickettsia prowazekii str. Madrid E, complete genome
2 Rickettsia typhi str. wilmington, complete genome
3 Rickettsia felis URRWXCal2, complete genome
4 Rickettsia akari str. Hartford chromosome, whole genome shotgun sequence
5 Rickettsia conorii str. Malish 7, complete genome
6 Rickettsia sibirica 246 rsib_agncrt, whole genome shotgun sequence
7 Rickettsia rickettsii chromosome, whole genome shotgun sequence

Reg_id: 523

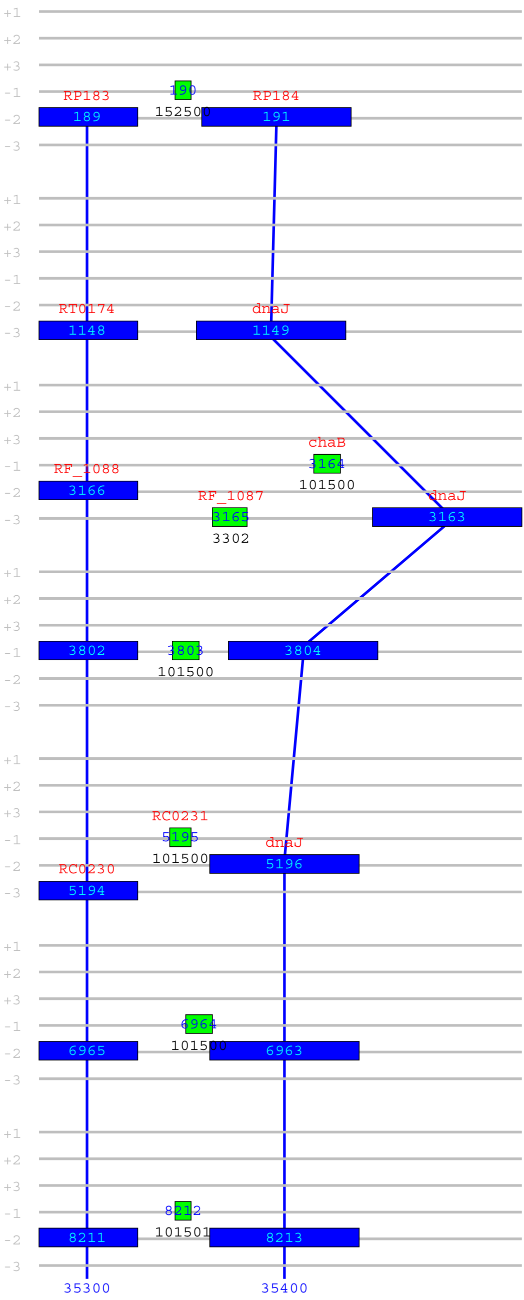

1 Rickettsia prowazekii str. Madrid E, complete genome
2 Rickettsia typhi str. wilmington, complete genome
3 Rickettsia felis URRWXCal2, complete genome
4 Rickettsia akari str. Hartford chromosome, whole genome shotgun sequence
5 Rickettsia conorii str. Malish 7, complete genome
6 Rickettsia sibirica 246 rsib_agncrt, whole genome shotgun sequence
7 Rickettsia rickettsii chromosome, whole genome shotgun sequence

Reg_id: 525

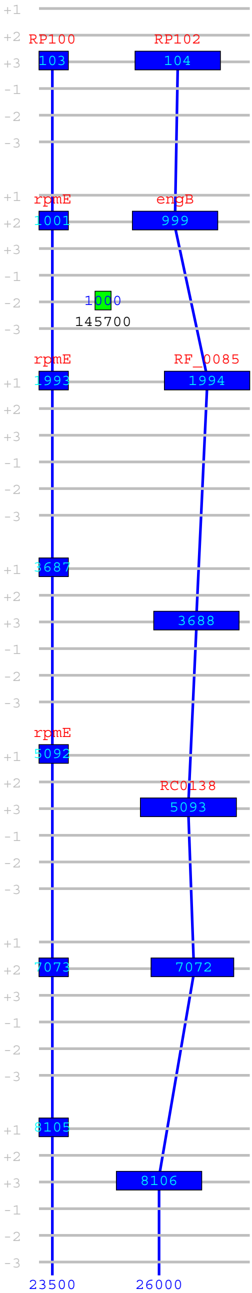

1 Rickettsia prowazekii str. Madrid E, complete genome
2 Rickettsia typhi str. wilmington, complete genome
3 Rickettsia felis URRWXCal2, complete genome
4 Rickettsia akari str. Hartford chromosome, whole genome shotgun sequence
5 Rickettsia conorii str. Malish 7, complete genome
6 Rickettsia sibirica 246 rsib_agncrt, whole genome shotgun sequence
7 Rickettsia rickettsii chromosome, whole genome shotgun sequence

Reg_id: 530

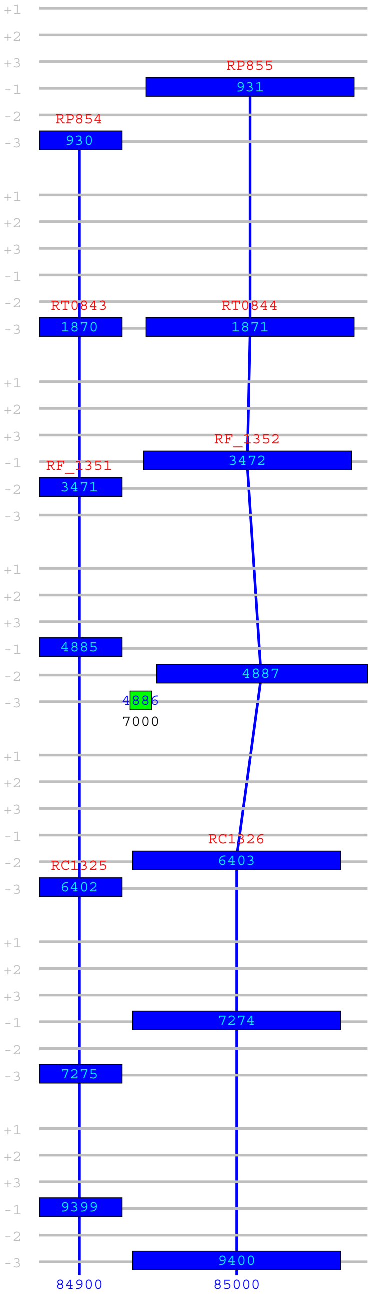

1 Rickettsia prowazekii str. Madrid E, complete genome
2 Rickettsia typhi str. wilmington, complete genome
3 Rickettsia felis URRWXCal2, complete genome
4 Rickettsia akari str. Hartford chromosome, whole genome shotgun sequence
5 Rickettsia conorii str. Malish 7, complete genome
6 Rickettsia sibirica 246 rsib_agncrt, whole genome shotgun sequence
7 Rickettsia rickettsii chromosome, whole genome shotgun sequence

Reg_id: 534

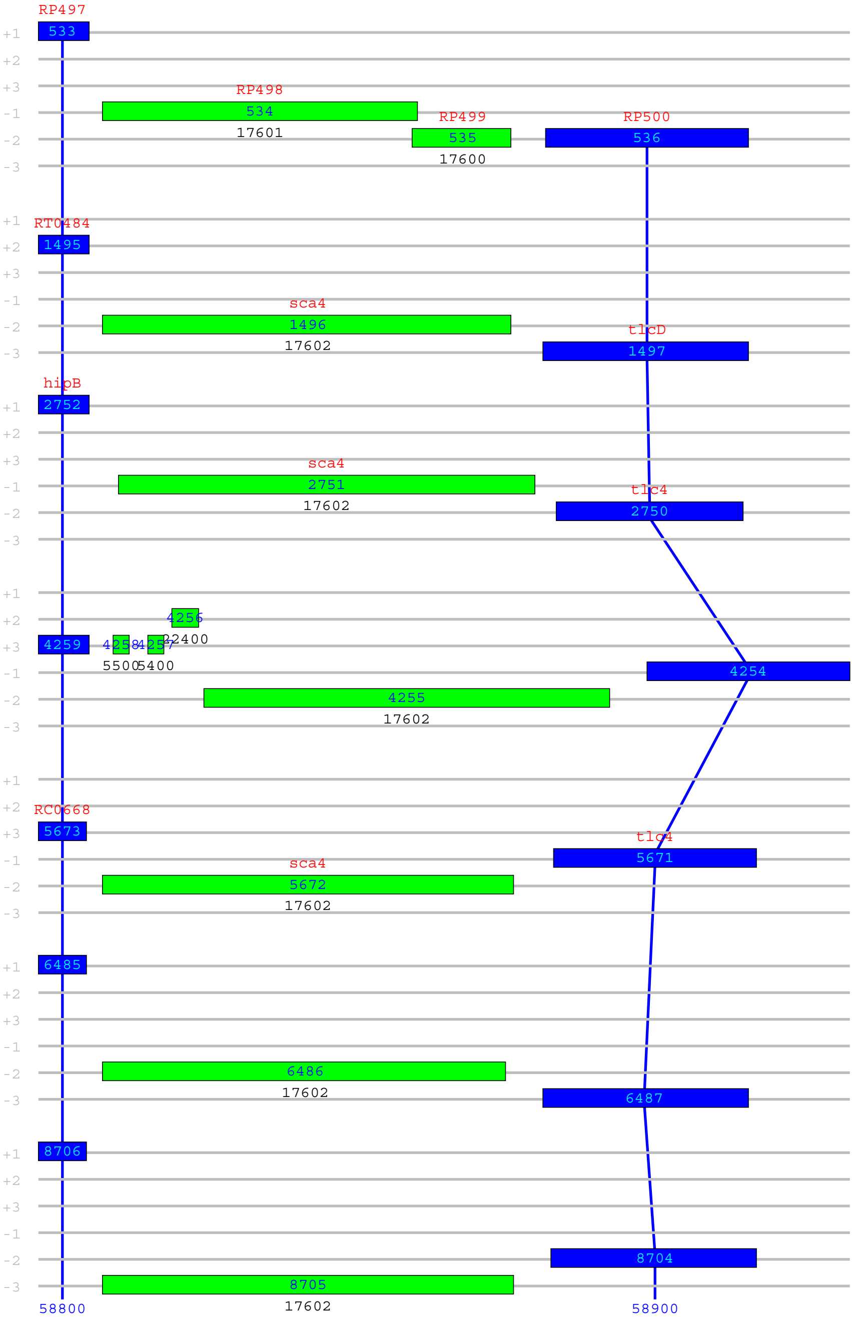

1 Rickettsia prowazekii str. Madrid E, complete genome
2 Rickettsia typhi str. wilmington, complete genome
3 Rickettsia felis URRWXCal2, complete genome
4 Rickettsia akari str. Hartford chromosome, whole genome shotgun sequence
5 Rickettsia conorii str. Malish 7, complete genome
6 Rickettsia sibirica 246 rsib_agncrt, whole genome shotgun sequence
7 Rickettsia rickettsii chromosome, whole genome shotgun sequence

Reg_id: 539

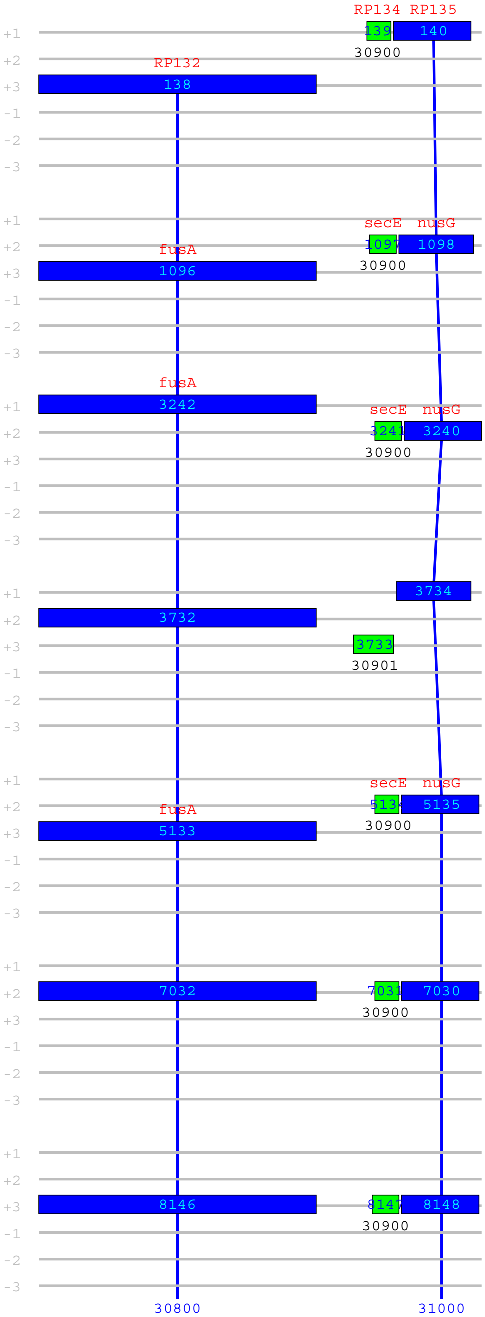

1 Rickettsia prowazekii str. Madrid E, complete genome
2 Rickettsia typhi str. wilmington, complete genome
3 Rickettsia felis URRWXCal2, complete genome
4 Rickettsia akari str. Hartford chromosome, whole genome shotgun sequence
5 Rickettsia conorii str. Malish 7, complete genome
6 Rickettsia sibirica 246 rsib_agncrt, whole genome shotgun sequence
7 Rickettsia rickettsii chromosome, whole genome shotgun sequence

Reg_id: 540

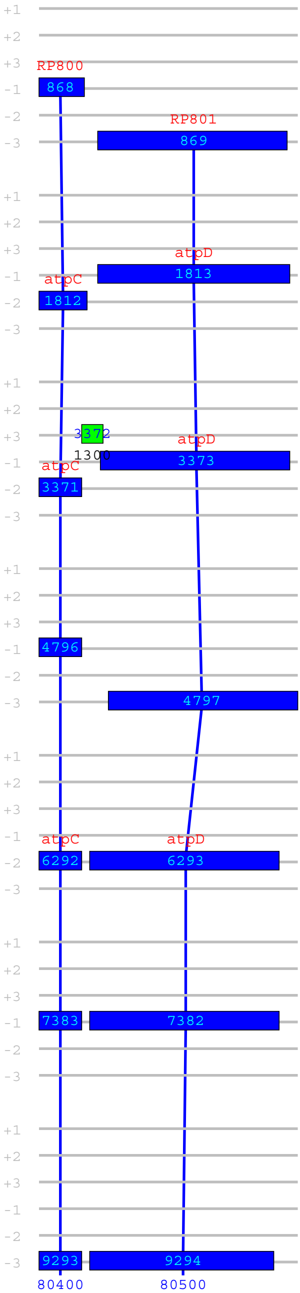

1 Rickettsia prowazekii str. Madrid E, complete genome
2 Rickettsia typhi str. wilmington, complete genome
3 Rickettsia felis URRWXCal2, complete genome
4 Rickettsia akari str. Hartford chromosome, whole genome shotgun sequence
5 Rickettsia conorii str. Malish 7, complete genome
6 Rickettsia sibirica 246 rsib_agncrt, whole genome shotgun sequence
7 Rickettsia rickettsii chromosome, whole genome shotgun sequence

Reg_id: 542

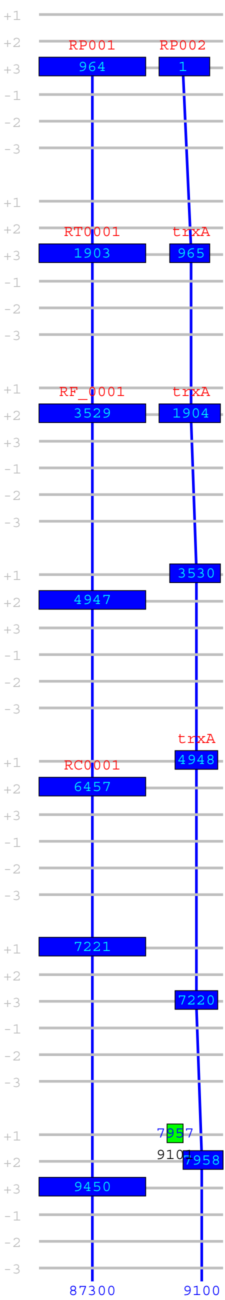

1 Rickettsia prowazekii str. Madrid E, complete genome
2 Rickettsia typhi str. wilmington, complete genome
3 Rickettsia felis URRWXCal2, complete genome
4 Rickettsia akari str. Hartford chromosome, whole genome shotgun sequence
5 Rickettsia conorii str. Malish 7, complete genome
6 Rickettsia sibirica 246 rsib_agncrt, whole genome shotgun sequence
7 Rickettsia rickettsii chromosome, whole genome shotgun sequence

Reg_id: 544

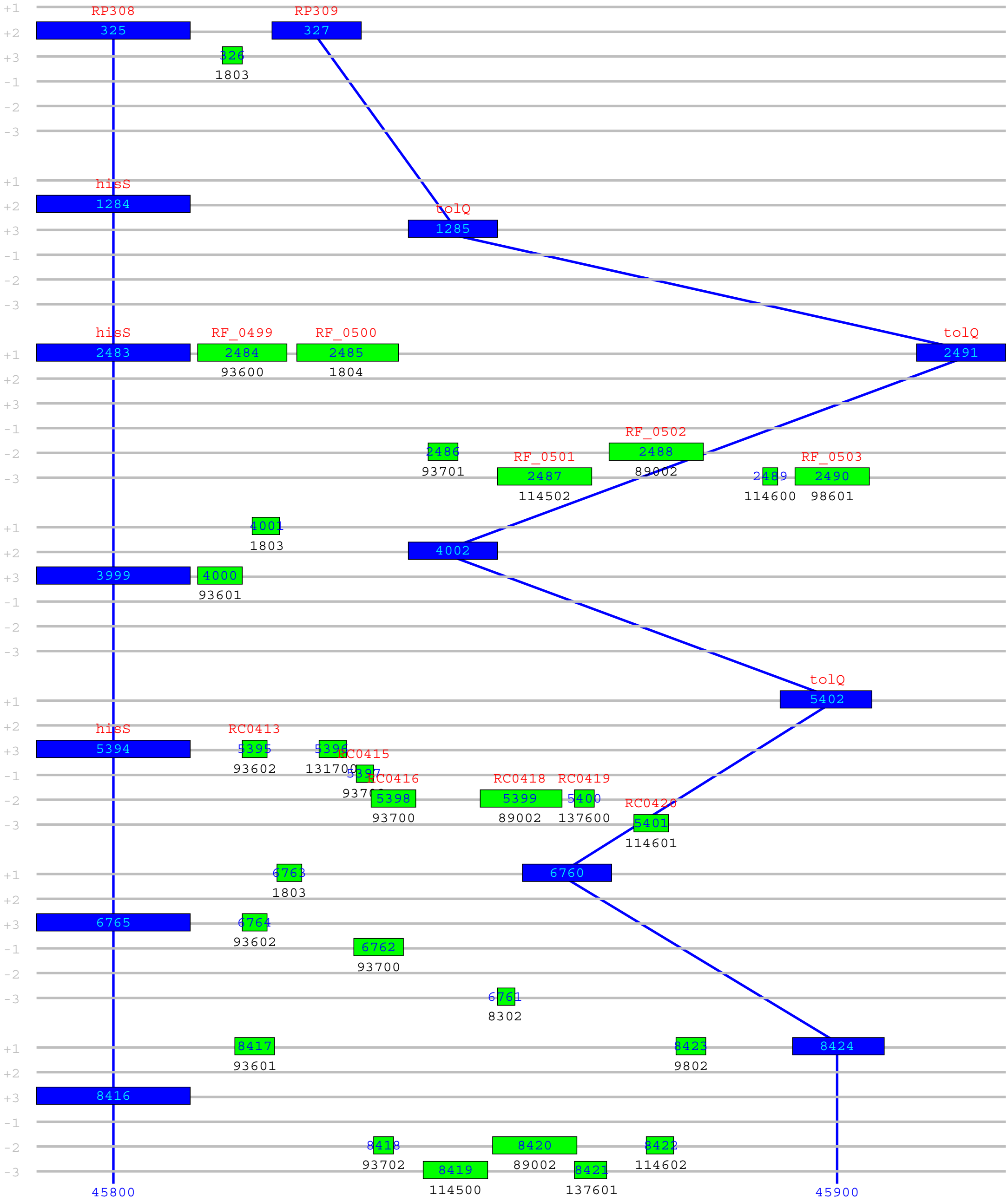

1 Rickettsia prowazekii str. Madrid E, complete genome
2 Rickettsia typhi str. wilmington, complete genome
3 Rickettsia felis URRWXCal2, complete genome
4 Rickettsia akari str. Hartford chromosome, whole genome shotgun sequence
5 Rickettsia conorii str. Malish 7, complete genome
6 Rickettsia sibirica 246 rsib_agncrt, whole genome shotgun sequence
7 Rickettsia rickettsii chromosome, whole genome shotgun sequence

Reg_id: 546

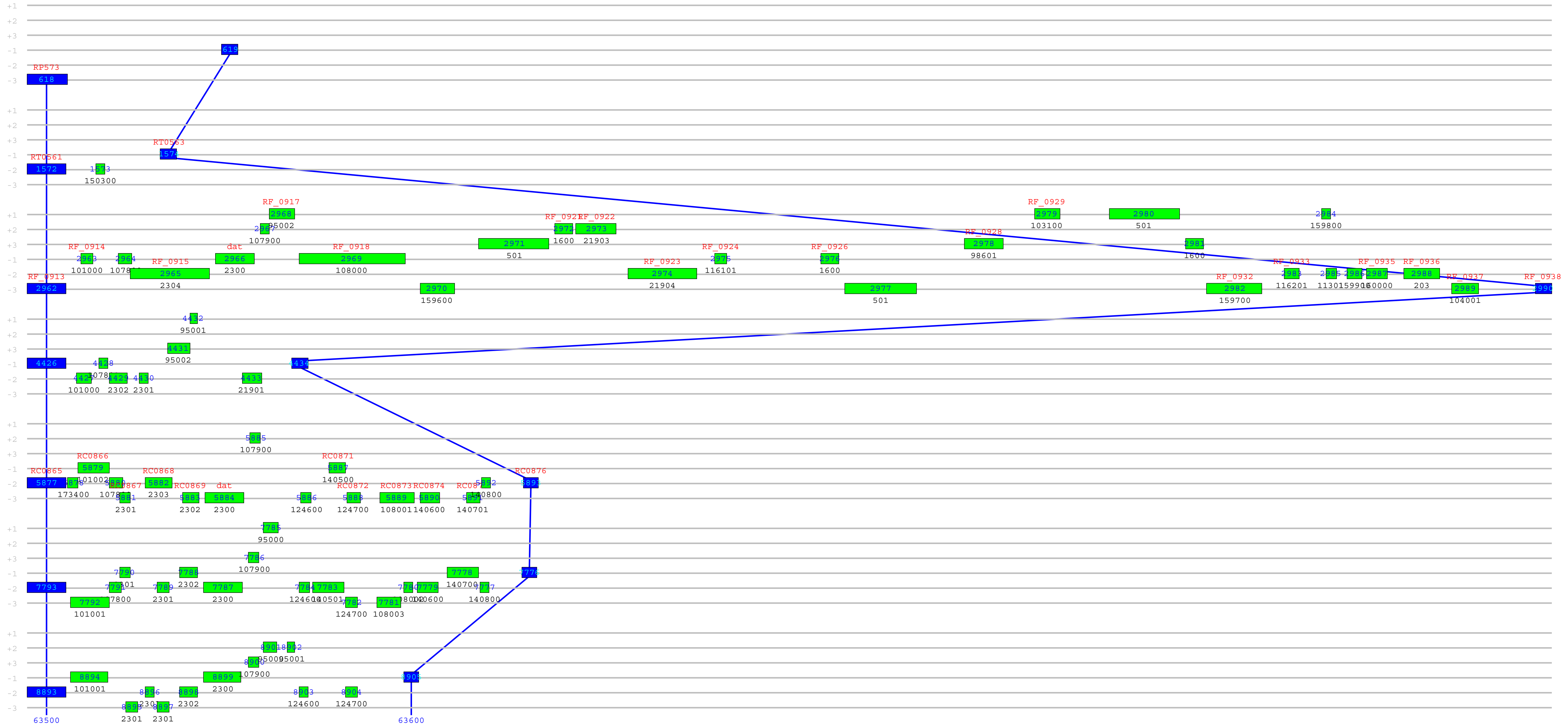

1 Rickettsia prowazekii str. Madrid E, complete genome
2 Rickettsia typhi str. wilmington, complete genome
3 Rickettsia felis URRWXCal2, complete genome
4 Rickettsia akari str. Hartford chromosome, whole genome shotgun sequence
5 Rickettsia conorii str. Malish 7, complete genome
6 Rickettsia sibirica 246 rsib_agncrt, whole genome shotgun sequence
7 Rickettsia rickettsii chromosome, whole genome shotgun sequence

Reg_id: 547

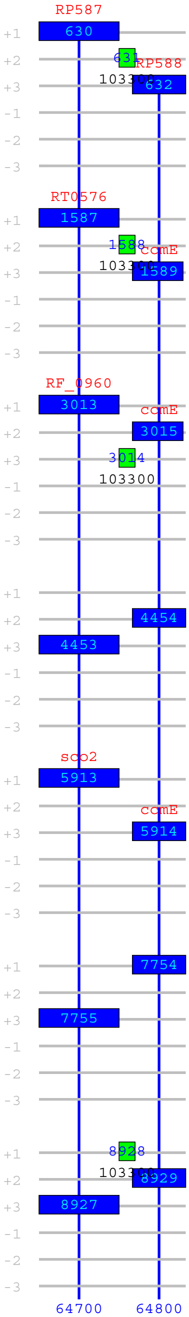

1 Rickettsia prowazekii str. Madrid E, complete genome
2 Rickettsia typhi str. wilmington, complete genome
3 Rickettsia felis URRWXCal2, complete genome
4 Rickettsia akari str. Hartford chromosome, whole genome shotgun sequence
5 Rickettsia conorii str. Malish 7, complete genome
6 Rickettsia sibirica 246 rsib_agncrt, whole genome shotgun sequence
7 Rickettsia rickettsii chromosome, whole genome shotgun sequence

Reg_id: 550

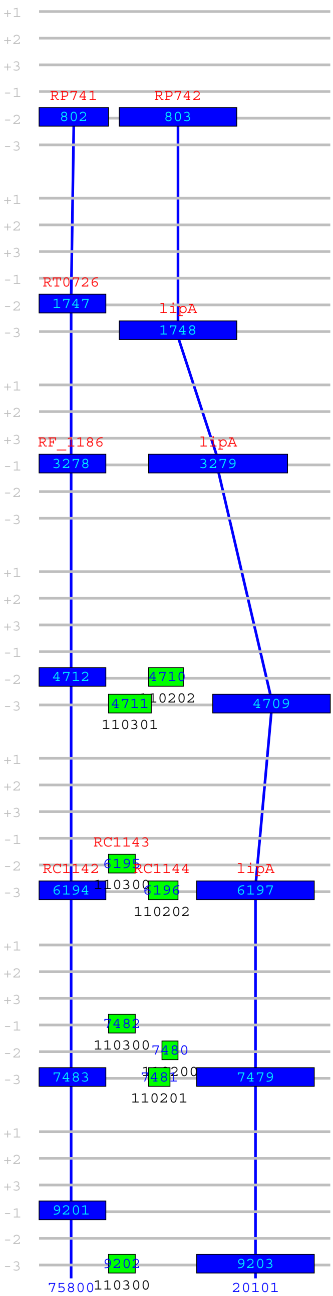

1 Rickettsia prowazekii str. Madrid E, complete genome
2 Rickettsia typhi str. wilmington, complete genome
3 Rickettsia felis URRWXCal2, complete genome
4 Rickettsia akari str. Hartford chromosome, whole genome shotgun sequence
5 Rickettsia conorii str. Malish 7, complete genome
6 Rickettsia sibirica 246 rsib_agncrt, whole genome shotgun sequence
7 Rickettsia rickettsii chromosome, whole genome shotgun sequence

Reg_id: 551

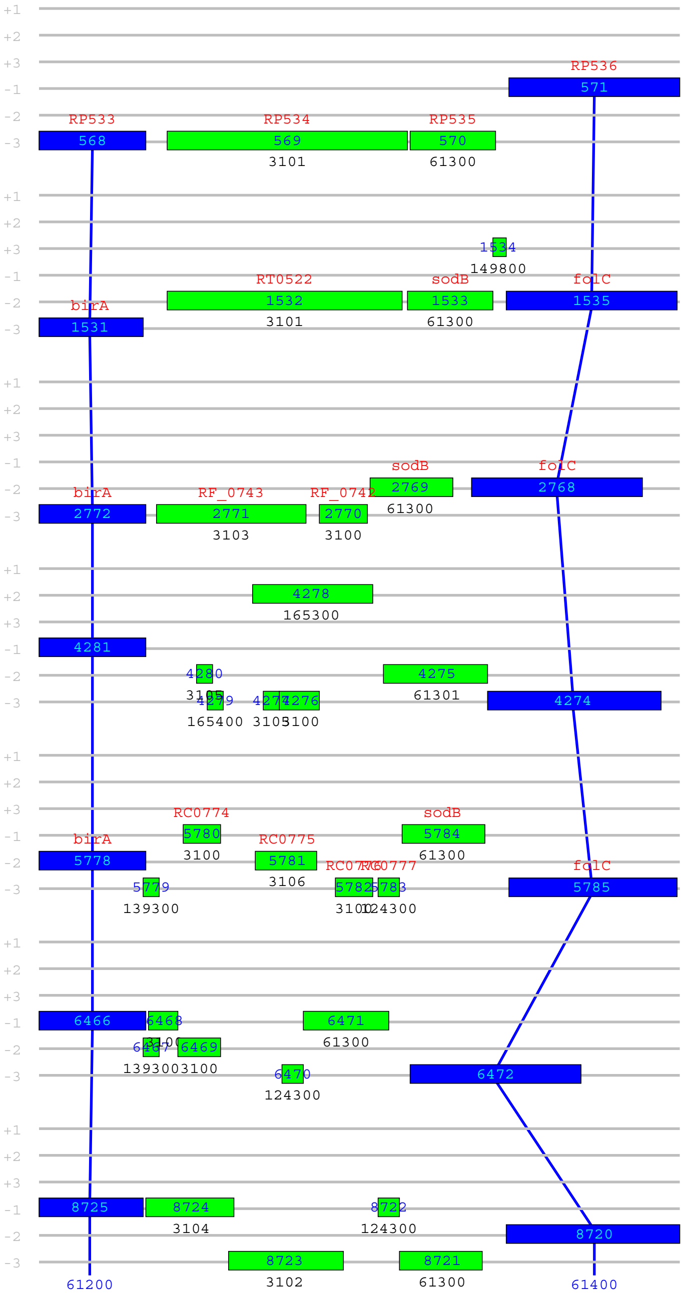

1 Rickettsia prowazekii str. Madrid E, complete genome
2 Rickettsia typhi str. wilmington, complete genome
3 Rickettsia felis URRWXCal2, complete genome
4 Rickettsia akari str. Hartford chromosome, whole genome shotgun sequence
5 Rickettsia conorii str. Malish 7, complete genome
6 Rickettsia sibirica 246 rsib_agncrt, whole genome shotgun sequence
7 Rickettsia rickettsii chromosome, whole genome shotgun sequence

Reg_id: 552

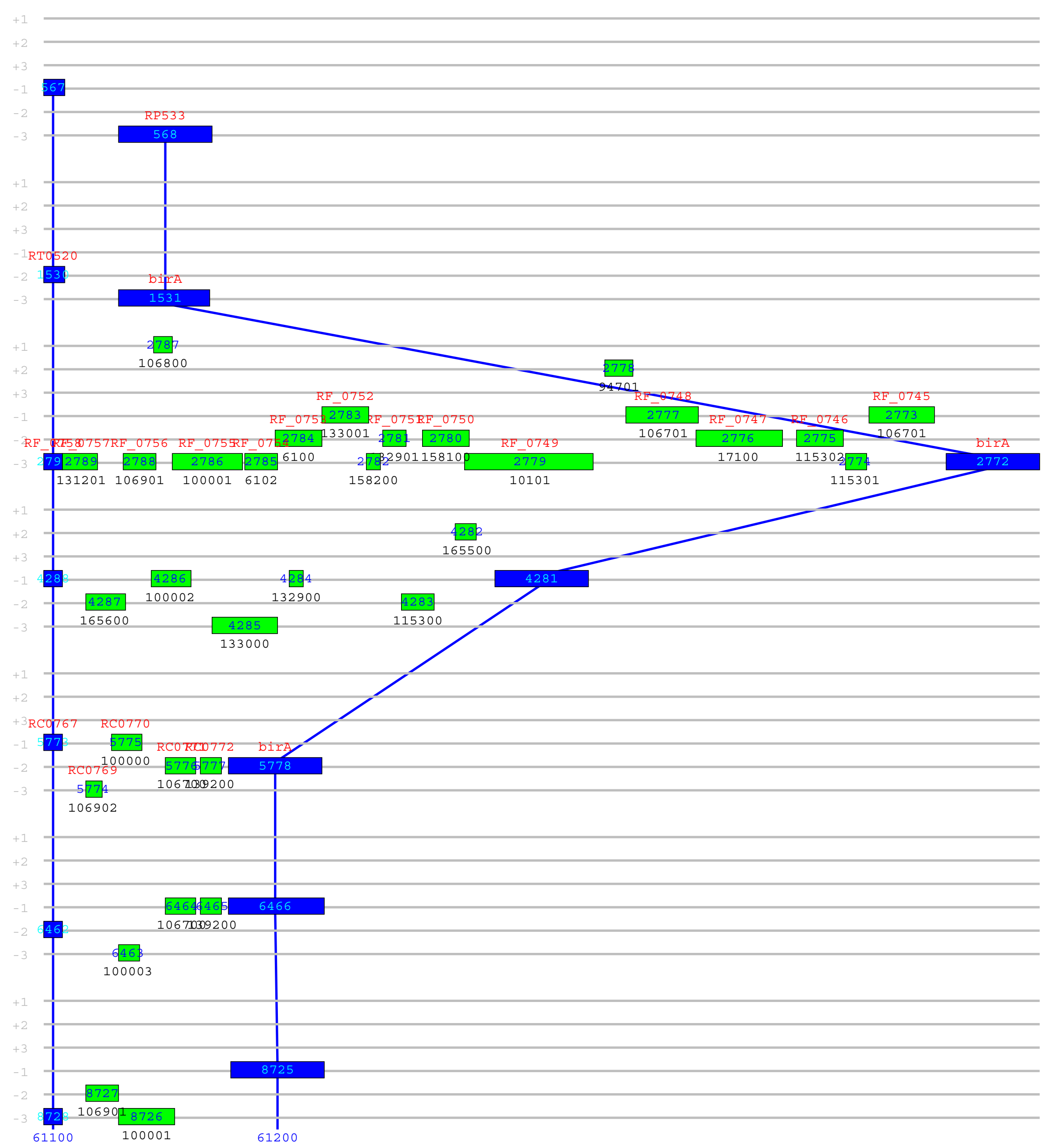

1 Rickettsia prowazekii str. Madrid E, complete genome
2 Rickettsia typhi str. wilmington, complete genome
3 Rickettsia felis URRWXCal2, complete genome
4 Rickettsia akari str. Hartford chromosome, whole genome shotgun sequence
5 Rickettsia conorii str. Malish 7, complete genome
6 Rickettsia sibirica 246 rsib_agncrt, whole genome shotgun sequence
7 Rickettsia rickettsii chromosome, whole genome shotgun sequence

Reg_id: 555

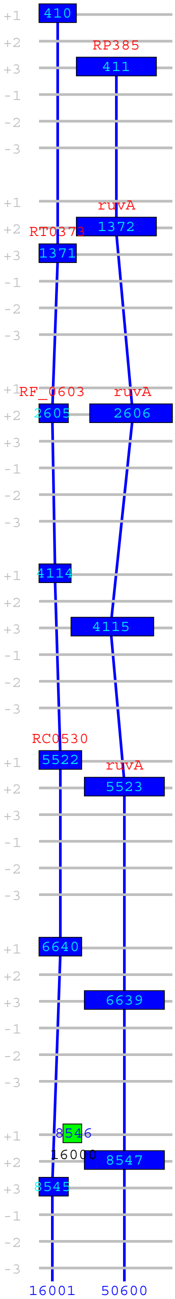

1 Rickettsia prowazekii str. Madrid E, complete genome
2 Rickettsia typhi str. wilmington, complete genome
3 Rickettsia felis URRWXCal2, complete genome
4 Rickettsia akari str. Hartford chromosome, whole genome shotgun sequence
5 Rickettsia conorii str. Malish 7, complete genome
6 Rickettsia sibirica 246 rsib_agncrt, whole genome shotgun sequence
7 Rickettsia rickettsii chromosome, whole genome shotgun sequence

Reg_id: 560

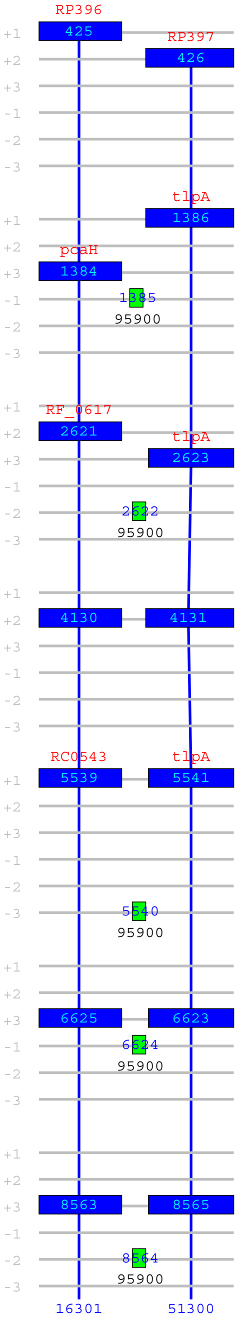

1 Rickettsia prowazekii str. Madrid E, complete genome
2 Rickettsia typhi str. wilmington, complete genome
3 Rickettsia felis URRWXCal2, complete genome
4 Rickettsia akari str. Hartford chromosome, whole genome shotgun sequence
5 Rickettsia conorii str. Malish 7, complete genome
6 Rickettsia sibirica 246 rsib_agncrt, whole genome shotgun sequence
7 Rickettsia rickettsii chromosome, whole genome shotgun sequence

Reg_id: 562

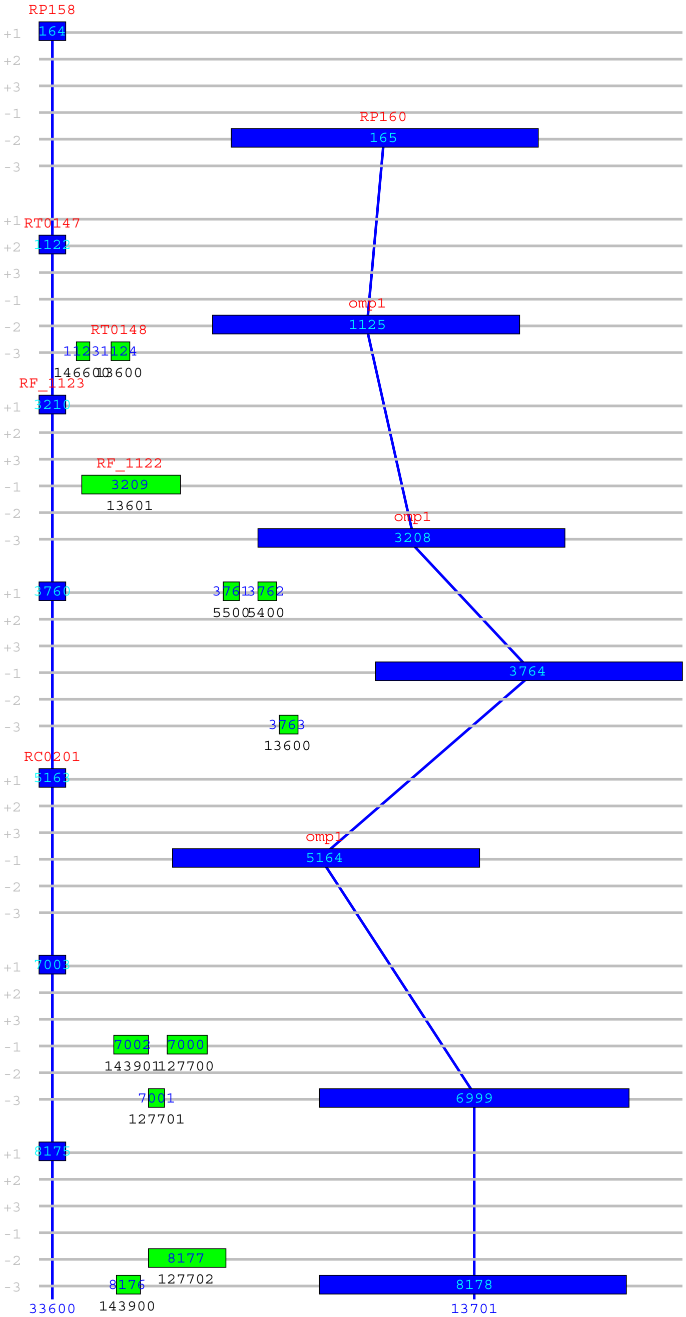

1 Rickettsia prowazekii str. Madrid E, complete genome
2 Rickettsia typhi str. wilmington, complete genome
3 Rickettsia felis URRWXCal2, complete genome
4 Rickettsia akari str. Hartford chromosome, whole genome shotgun sequence
5 Rickettsia conorii str. Malish 7, complete genome
6 Rickettsia sibirica 246 rsib_agncrt, whole genome shotgun sequence
7 Rickettsia rickettsii chromosome, whole genome shotgun sequence

Reg_id: 564

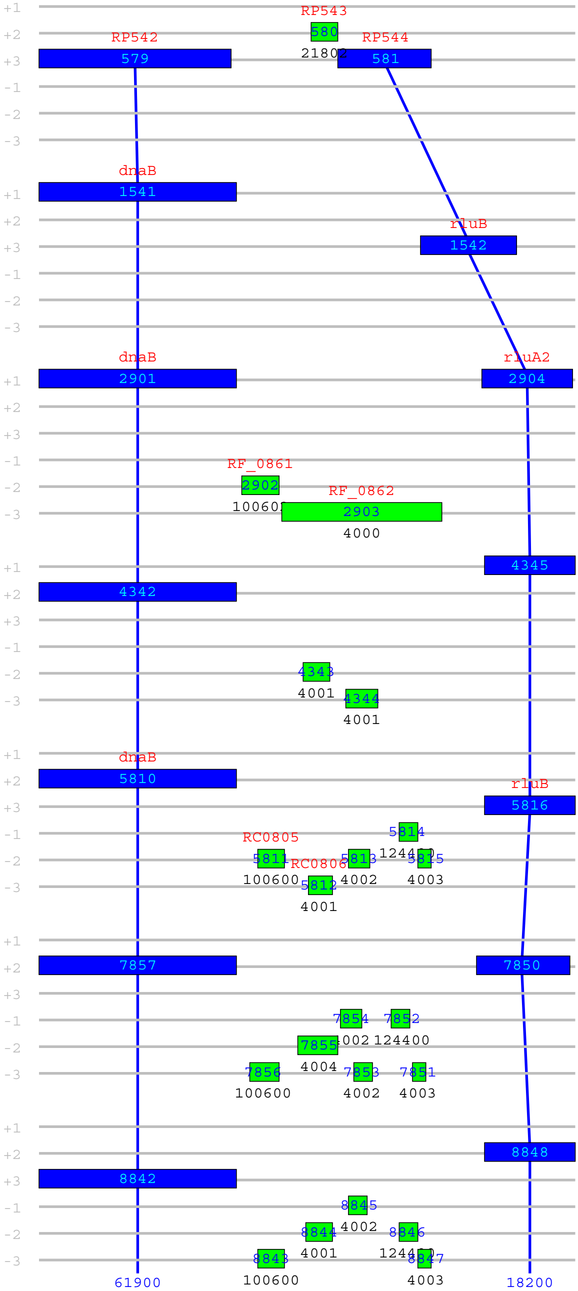

1 Rickettsia prowazekii str. Madrid E, complete genome
2 Rickettsia typhi str. wilmington, complete genome
3 Rickettsia felis URRWXCal2, complete genome
4 Rickettsia akari str. Hartford chromosome, whole genome shotgun sequence
5 Rickettsia conorii str. Malish 7, complete genome
6 Rickettsia sibirica 246 rsib_agncrt, whole genome shotgun sequence
7 Rickettsia rickettsii chromosome, whole genome shotgun sequence

Reg_id: 565

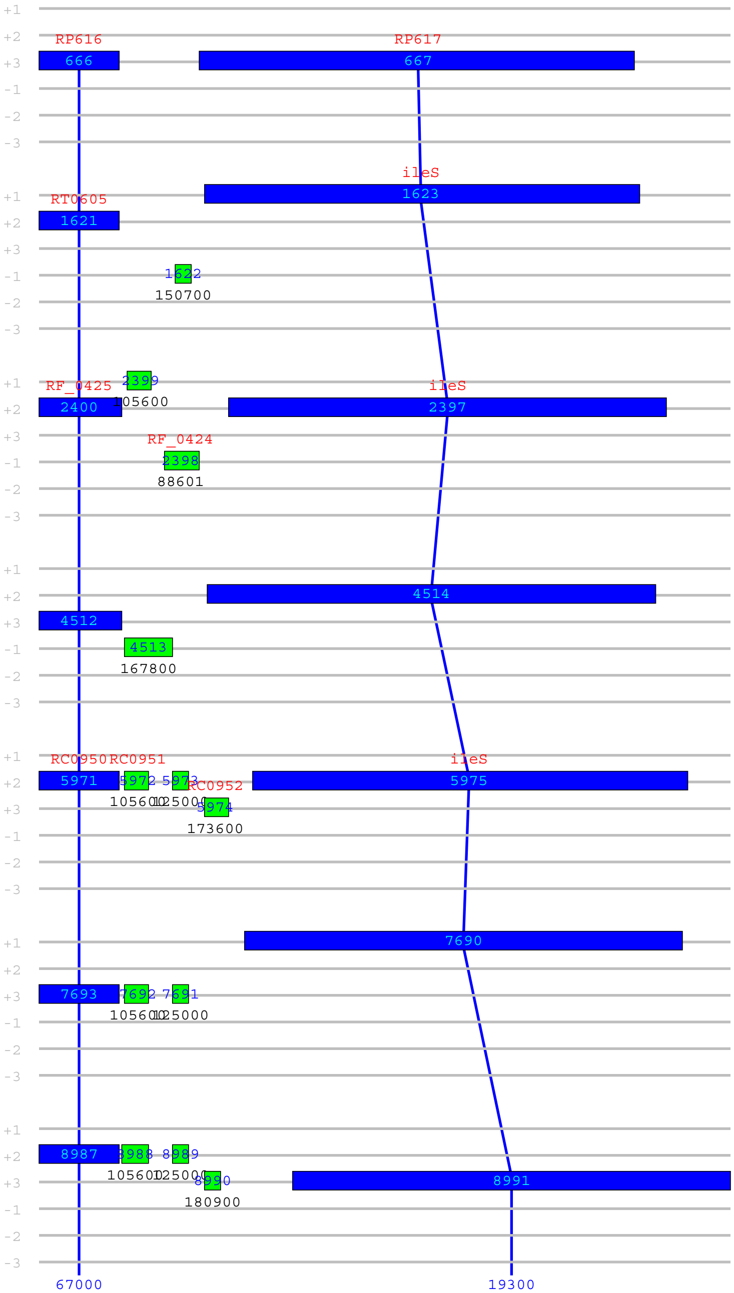

1 Rickettsia prowazekii str. Madrid E, complete genome
2 Rickettsia typhi str. wilmington, complete genome
3 Rickettsia felis URRWXCal2, complete genome
4 Rickettsia akari str. Hartford chromosome, whole genome shotgun sequence
5 Rickettsia conorii str. Malish 7, complete genome
6 Rickettsia sibirica 246 rsib_agncrt, whole genome shotgun sequence
7 Rickettsia rickettsii chromosome, whole genome shotgun sequence

Reg_id: 570

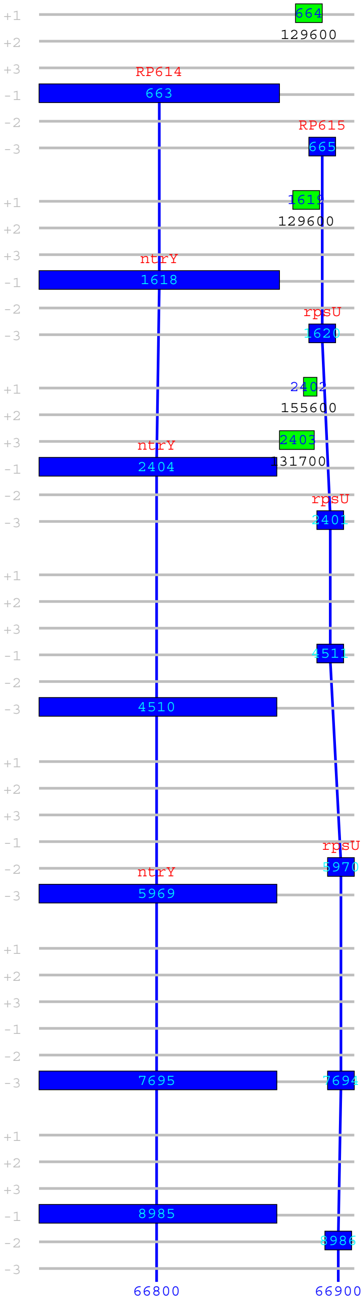

1 Rickettsia prowazekii str. Madrid E, complete genome
2 Rickettsia typhi str. wilmington, complete genome
3 Rickettsia felis URRWXCal2, complete genome
4 Rickettsia akari str. Hartford chromosome, whole genome shotgun sequence
5 Rickettsia conorii str. Malish 7, complete genome
6 Rickettsia sibirica 246 rsib_agncrt, whole genome shotgun sequence
7 Rickettsia rickettsii chromosome, whole genome shotgun sequence

Reg_id: 571

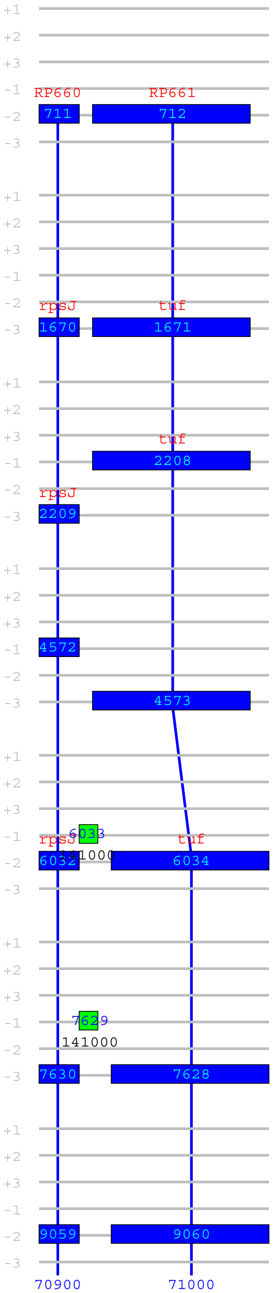

1 Rickettsia prowazekii str. Madrid E, complete genome
2 Rickettsia typhi str. wilmington, complete genome
3 Rickettsia felis URRWXCal2, complete genome
4 Rickettsia akari str. Hartford chromosome, whole genome shotgun sequence
5 Rickettsia conorii str. Malish 7, complete genome
6 Rickettsia sibirica 246 rsib_agncrt, whole genome shotgun sequence
7 Rickettsia rickettsii chromosome, whole genome shotgun sequence

Reg_id: 574

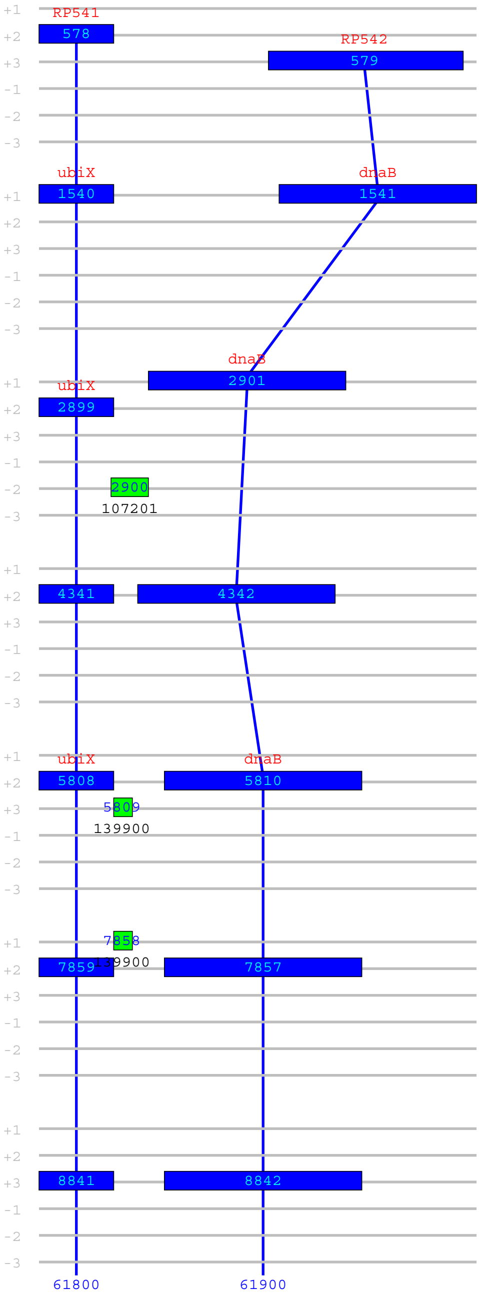

1 Rickettsia prowazekii str. Madrid E, complete genome
2 Rickettsia typhi str. wilmington, complete genome
3 Rickettsia felis URRWXCal2, complete genome
4 Rickettsia akari str. Hartford chromosome, whole genome shotgun sequence
5 Rickettsia conorii str. Malish 7, complete genome
6 Rickettsia sibirica 246 rsib_agncrt, whole genome shotgun sequence
7 Rickettsia rickettsii chromosome, whole genome shotgun sequence

Reg_id: 576

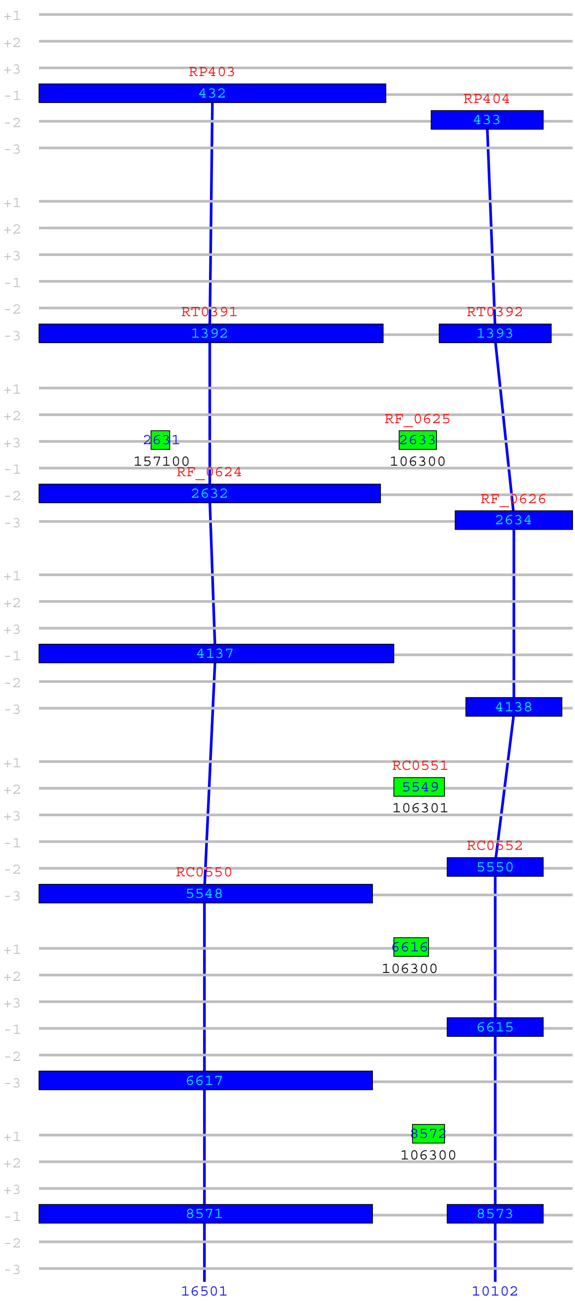

1 Rickettsia prowazekii str. Madrid E, complete genome
2 Rickettsia typhi str. wilmington, complete genome
3 Rickettsia felis URRWXCal2, complete genome
4 Rickettsia akari str. Hartford chromosome, whole genome shotgun sequence
5 Rickettsia conorii str. Malish 7, complete genome
6 Rickettsia sibirica 246 rsib_agncrt, whole genome shotgun sequence
7 Rickettsia rickettsii chromosome, whole genome shotgun sequence

Reg_id: 577

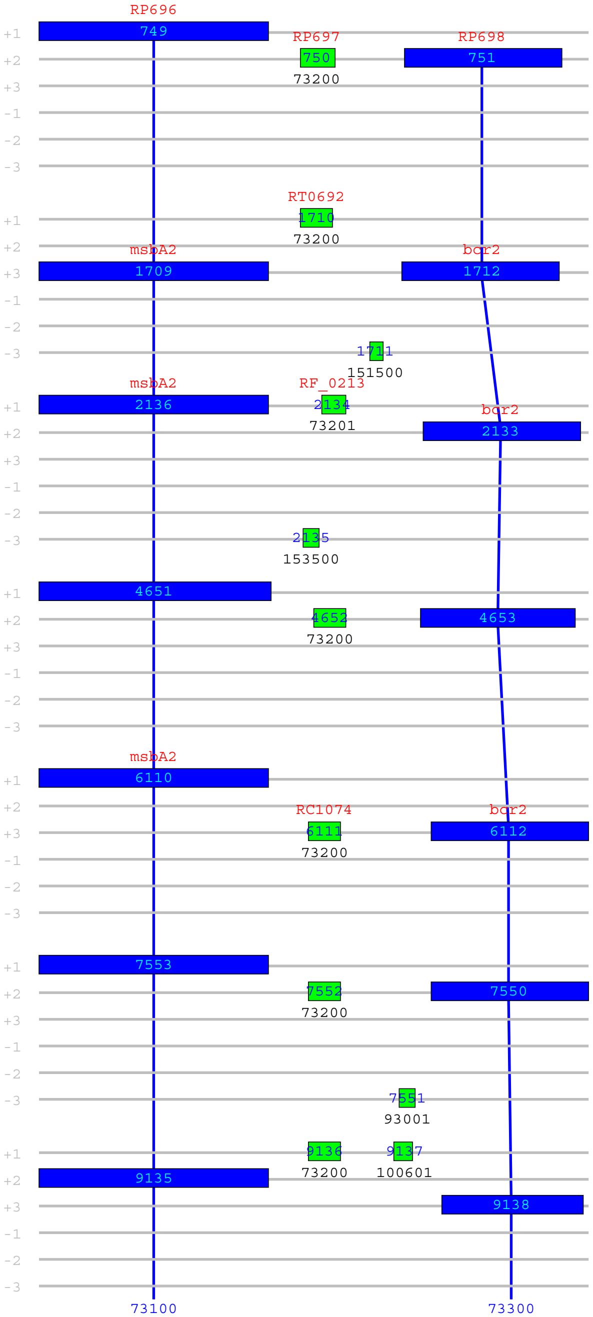

1 Rickettsia prowazekii str. Madrid E, complete genome
2 Rickettsia typhi str. wilmington, complete genome
3 Rickettsia felis URRWXCal2, complete genome
4 Rickettsia akari str. Hartford chromosome, whole genome shotgun sequence
5 Rickettsia conorii str. Malish 7, complete genome
6 Rickettsia sibirica 246 rsib_agncrt, whole genome shotgun sequence
7 Rickettsia rickettsii chromosome, whole genome shotgun sequence

Reg_id: 581

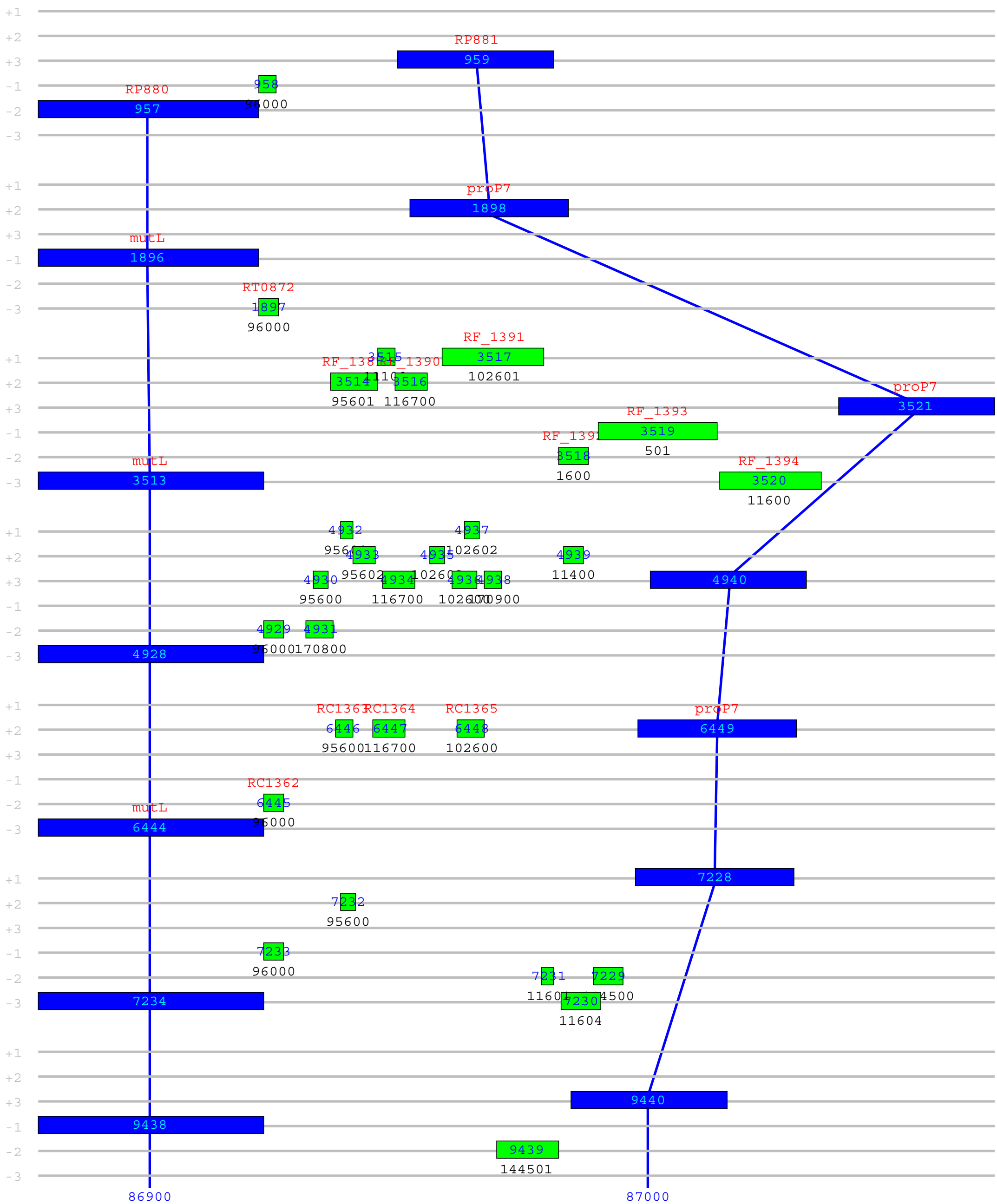

1 Rickettsia prowazekii str. Madrid E, complete genome
2 Rickettsia typhi str. wilmington, complete genome
3 Rickettsia felis URRWXCal2, complete genome
4 Rickettsia akari str. Hartford chromosome, whole genome shotgun sequence
5 Rickettsia conorii str. Malish 7, complete genome
6 Rickettsia sibirica 246 rsib_agncrt, whole genome shotgun sequence
7 Rickettsia rickettsii chromosome, whole genome shotgun sequence

Reg_id: 584

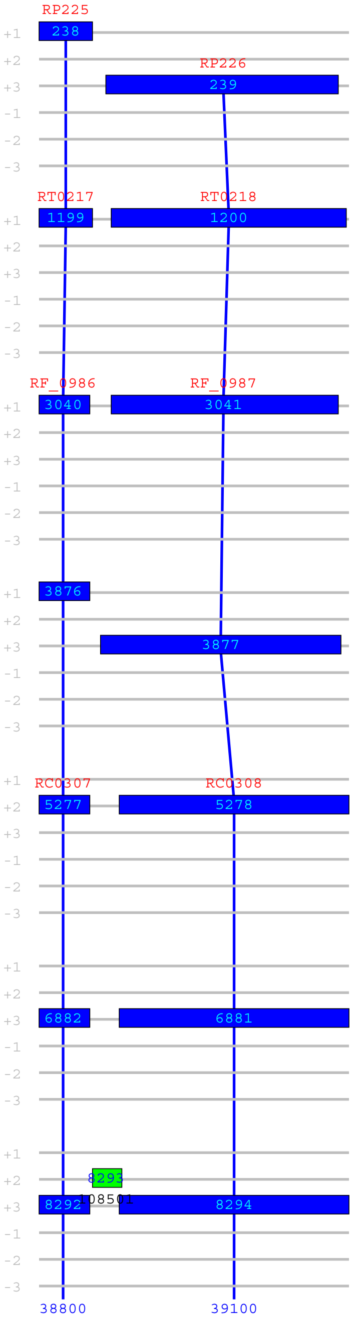

1 Rickettsia prowazekii str. Madrid E, complete genome
2 Rickettsia typhi str. wilmington, complete genome
3 Rickettsia felis URRWXCal2, complete genome
4 Rickettsia akari str. Hartford chromosome, whole genome shotgun sequence
5 Rickettsia conorii str. Malish 7, complete genome
6 Rickettsia sibirica 246 rsib_agncrt, whole genome shotgun sequence
7 Rickettsia rickettsii chromosome, whole genome shotgun sequence

Reg_id: 586

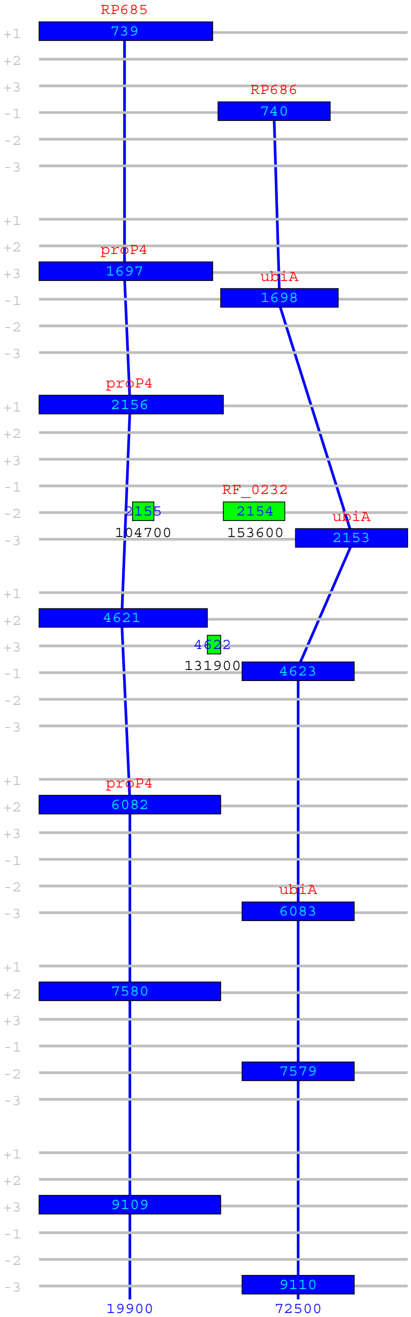

1 Rickettsia prowazekii str. Madrid E, complete genome
2 Rickettsia typhi str. wilmington, complete genome
3 Rickettsia felis URRWXCal2, complete genome
4 Rickettsia akari str. Hartford chromosome, whole genome shotgun sequence
5 Rickettsia conorii str. Malish 7, complete genome
6 Rickettsia sibirica 246 rsib_agncrt, whole genome shotgun sequence
7 Rickettsia rickettsii chromosome, whole genome shotgun sequence

Reg_id: 588

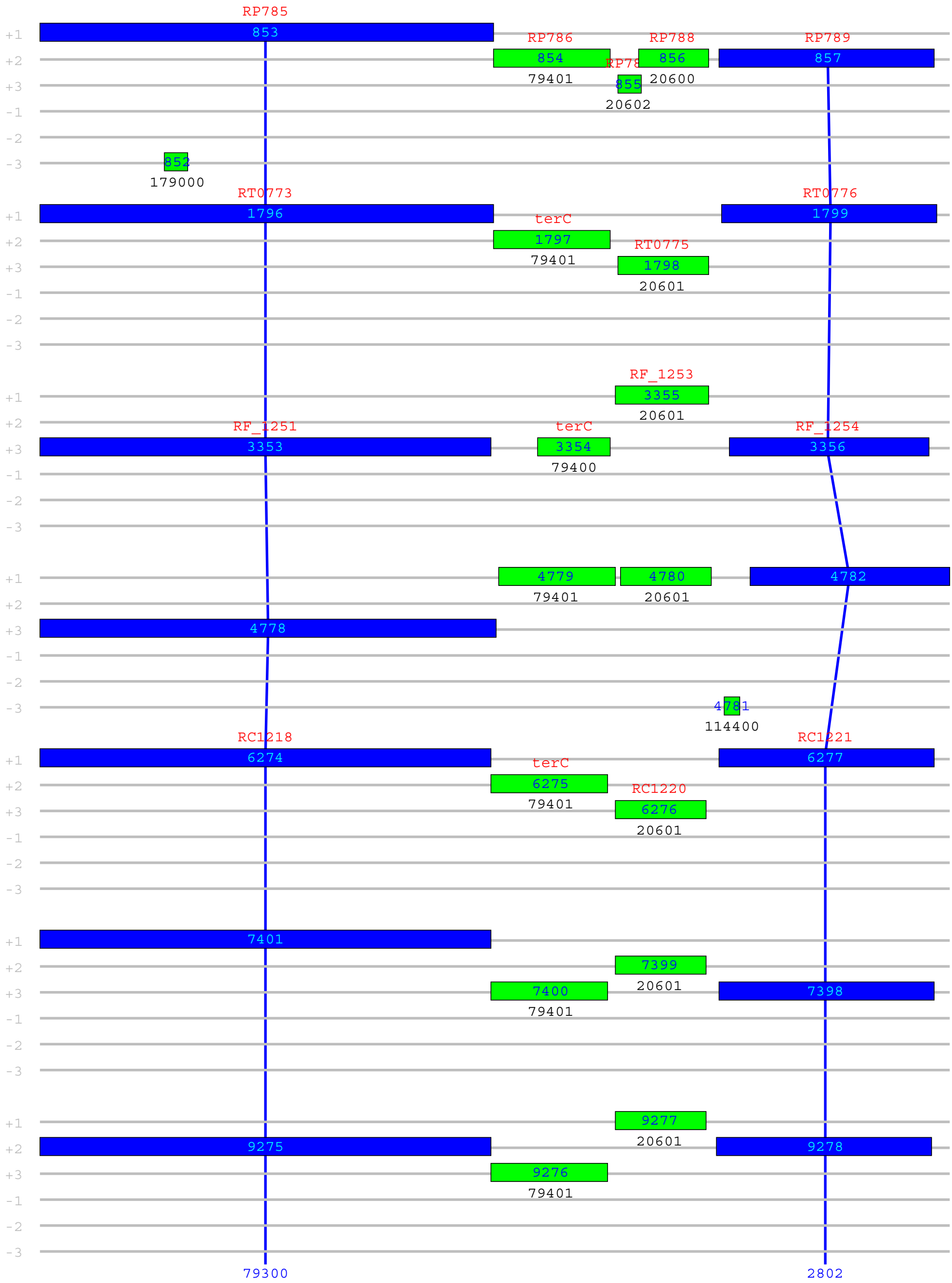

1 Rickettsia prowazekii str. Madrid E, complete genome
2 Rickettsia typhi str. wilmington, complete genome
3 Rickettsia felis URRWXCal2, complete genome
4 Rickettsia akari str. Hartford chromosome, whole genome shotgun sequence
5 Rickettsia conorii str. Malish 7, complete genome
6 Rickettsia sibirica 246 rsib_agncrt, whole genome shotgun sequence
7 Rickettsia rickettsii chromosome, whole genome shotgun sequence

Reg_id: 589

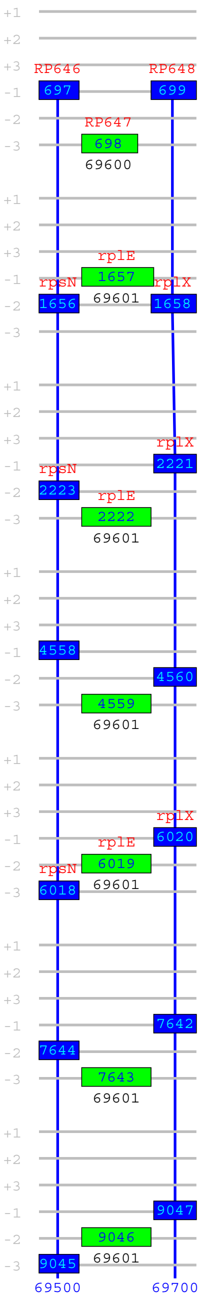

1 Rickettsia prowazekii str. Madrid E, complete genome
2 Rickettsia typhi str. wilmington, complete genome
3 Rickettsia felis URRWXCal2, complete genome
4 Rickettsia akari str. Hartford chromosome, whole genome shotgun sequence
5 Rickettsia conorii str. Malish 7, complete genome
6 Rickettsia sibirica 246 rsib_agncrt, whole genome shotgun sequence
7 Rickettsia rickettsii chromosome, whole genome shotgun sequence

Reg_id: 590

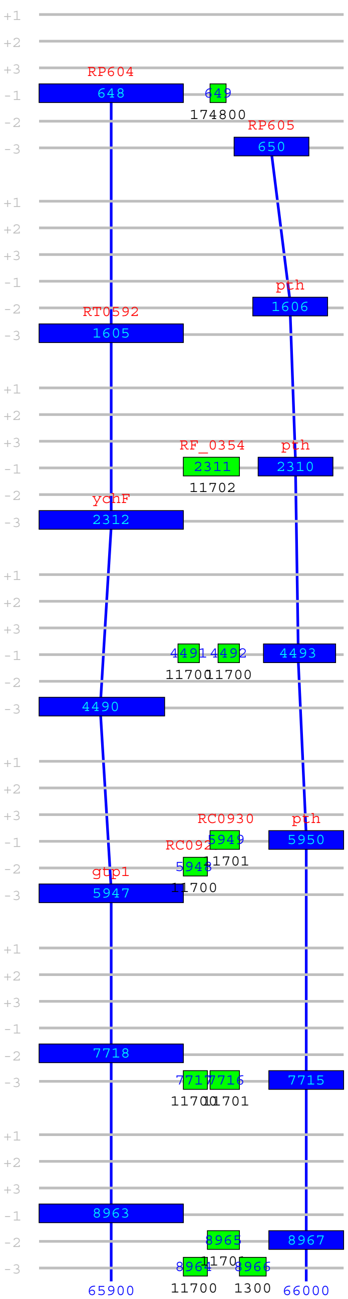

1 Rickettsia prowazekii str. Madrid E, complete genome
2 Rickettsia typhi str. wilmington, complete genome
3 Rickettsia felis URRWXCal2, complete genome
4 Rickettsia akari str. Hartford chromosome, whole genome shotgun sequence
5 Rickettsia conorii str. Malish 7, complete genome
6 Rickettsia sibirica 246 rsib_agncrt, whole genome shotgun sequence
7 Rickettsia rickettsii chromosome, whole genome shotgun sequence

Reg_id: 595

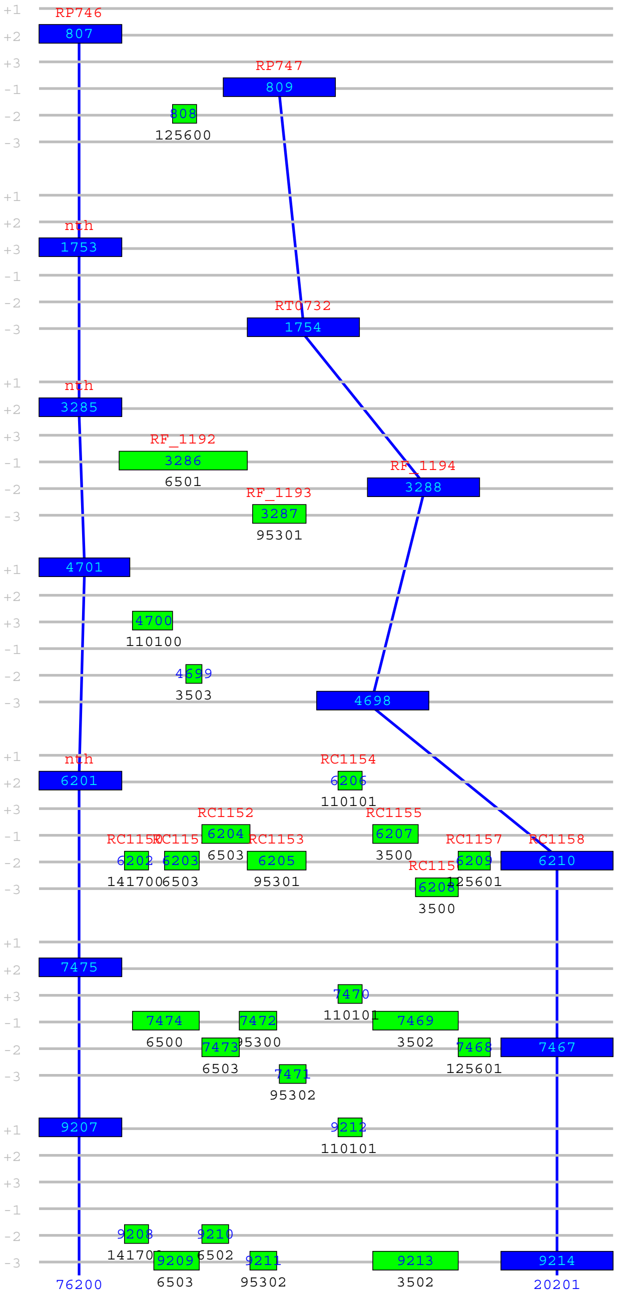

1 Rickettsia prowazekii str. Madrid E, complete genome
2 Rickettsia typhi str. wilmington, complete genome
3 Rickettsia felis URRWXCal2, complete genome
4 Rickettsia akari str. Hartford chromosome, whole genome shotgun sequence
5 Rickettsia conorii str. Malish 7, complete genome
6 Rickettsia sibirica 246 rsib_agncrt, whole genome shotgun sequence
7 Rickettsia rickettsii chromosome, whole genome shotgun sequence

Reg_id: 598

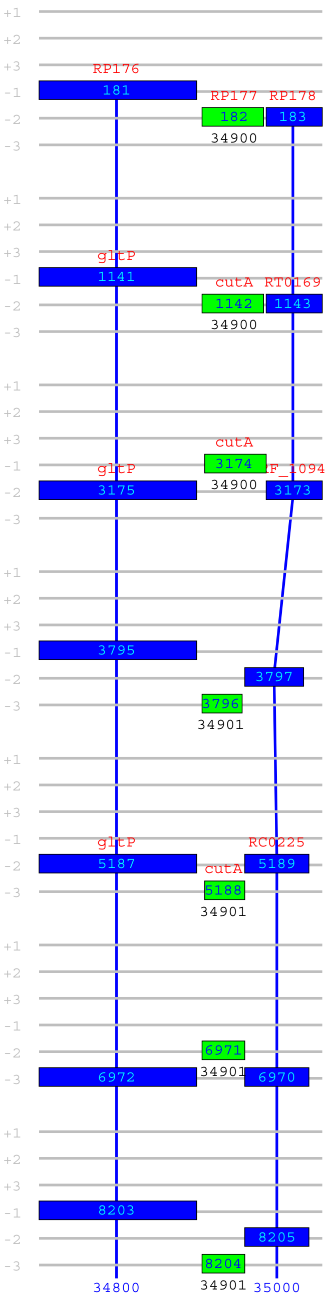

1 Rickettsia prowazekii str. Madrid E, complete genome
2 Rickettsia typhi str. wilmington, complete genome
3 Rickettsia felis URRWXCal2, complete genome
4 Rickettsia akari str. Hartford chromosome, whole genome shotgun sequence
5 Rickettsia conorii str. Malish 7, complete genome
6 Rickettsia sibirica 246 rsib_agncrt, whole genome shotgun sequence
7 Rickettsia rickettsii chromosome, whole genome shotgun sequence

Reg_id: 599

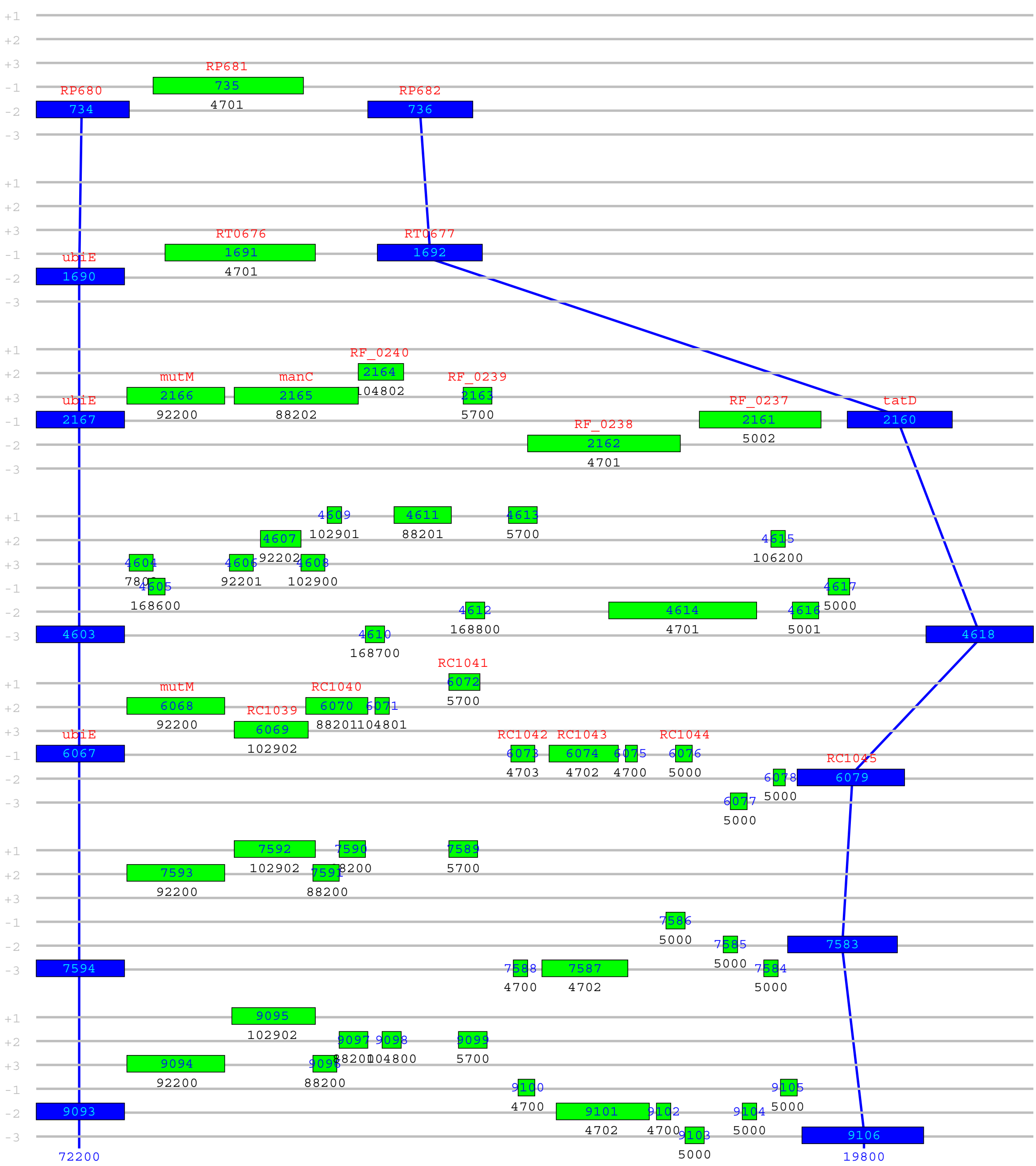

1 Rickettsia prowazekii str. Madrid E, complete genome
2 Rickettsia typhi str. wilmington, complete genome
3 Rickettsia felis URRWXCal2, complete genome
4 Rickettsia akari str. Hartford chromosome, whole genome shotgun sequence
5 Rickettsia conorii str. Malish 7, complete genome
6 Rickettsia sibirica 246 rsib_agncrt, whole genome shotgun sequence
7 Rickettsia rickettsii chromosome, whole genome shotgun sequence

Reg_id: 601

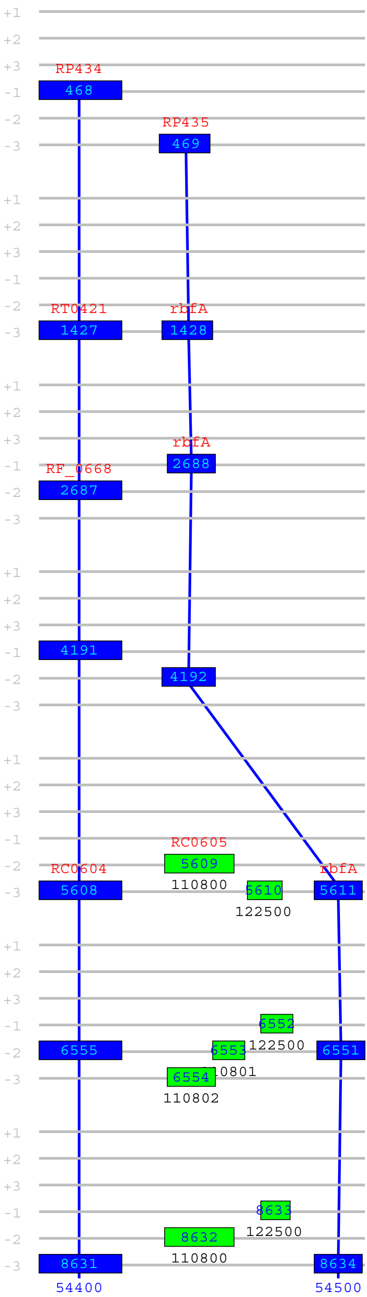

1 Rickettsia prowazekii str. Madrid E, complete genome
2 Rickettsia typhi str. wilmington, complete genome
3 Rickettsia felis URRWXCal2, complete genome
4 Rickettsia akari str. Hartford chromosome, whole genome shotgun sequence
5 Rickettsia conorii str. Malish 7, complete genome
6 Rickettsia sibirica 246 rsib_agncrt, whole genome shotgun sequence
7 Rickettsia rickettsii chromosome, whole genome shotgun sequence

Reg_id: 603

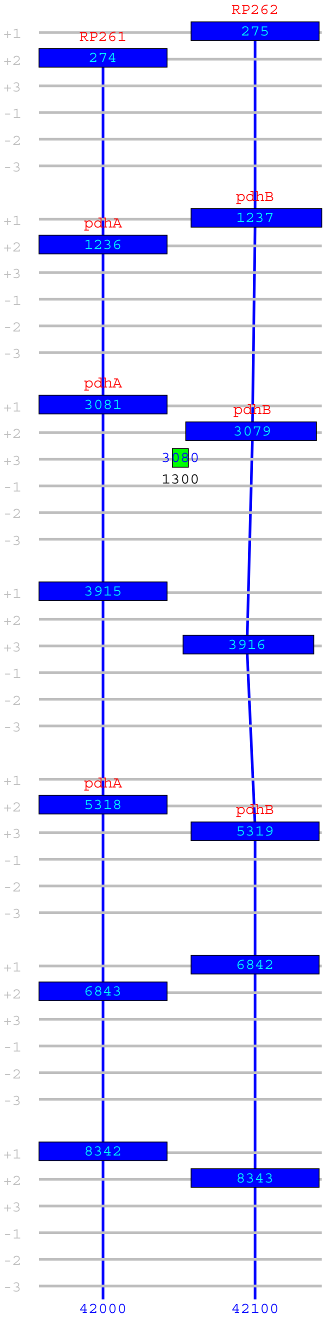

1 Rickettsia prowazekii str. Madrid E, complete genome
2 Rickettsia typhi str. wilmington, complete genome
3 Rickettsia felis URRWXCal2, complete genome
4 Rickettsia akari str. Hartford chromosome, whole genome shotgun sequence
5 Rickettsia conorii str. Malish 7, complete genome
6 Rickettsia sibirica 246 rsib_agncrt, whole genome shotgun sequence
7 Rickettsia rickettsii chromosome, whole genome shotgun sequence

Reg_id: 604

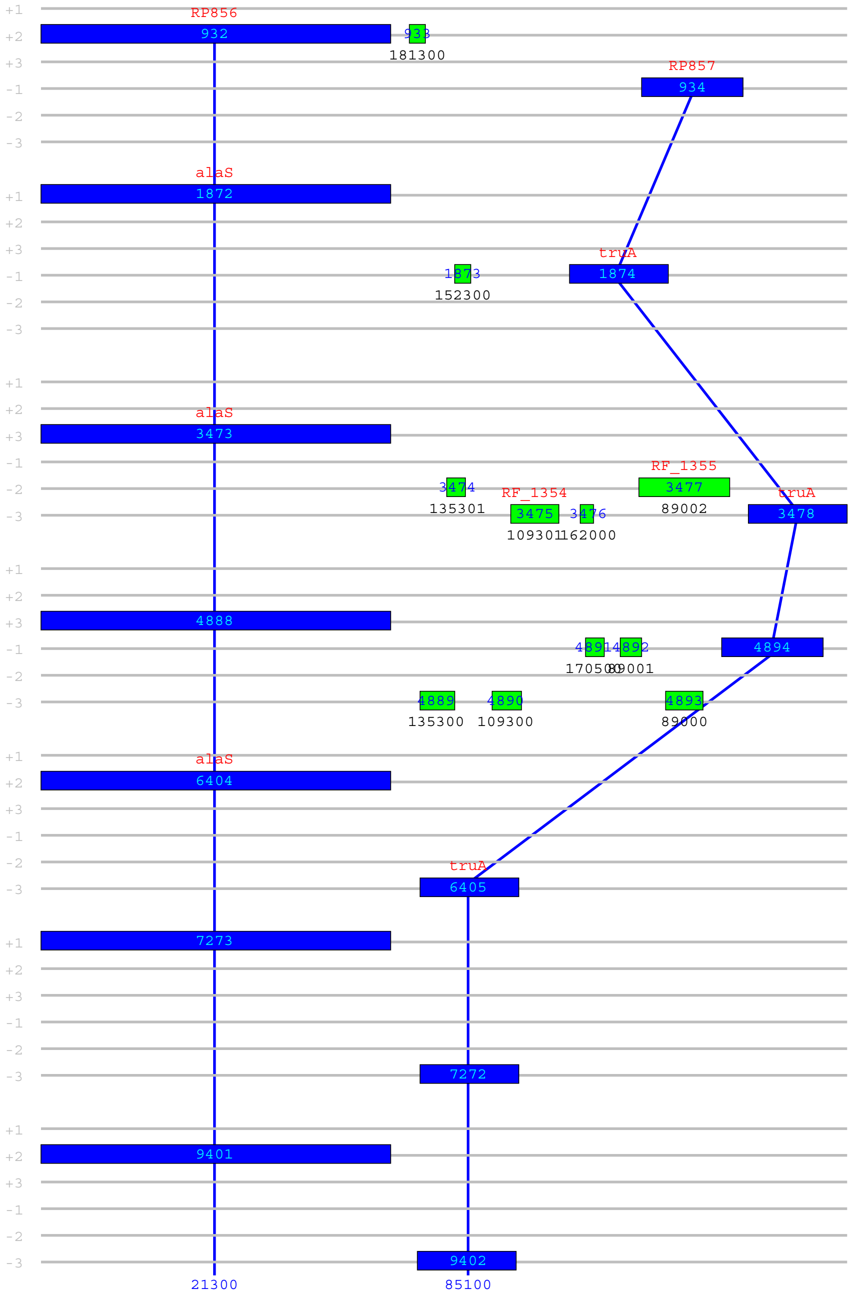

1 Rickettsia prowazekii str. Madrid E, complete genome
2 Rickettsia typhi str. wilmington, complete genome
3 Rickettsia felis URRWXCal2, complete genome
4 Rickettsia akari str. Hartford chromosome, whole genome shotgun sequence
5 Rickettsia conorii str. Malish 7, complete genome
6 Rickettsia sibirica 246 rsib_agncrt, whole genome shotgun sequence
7 Rickettsia rickettsii chromosome, whole genome shotgun sequence

Reg_id: 605

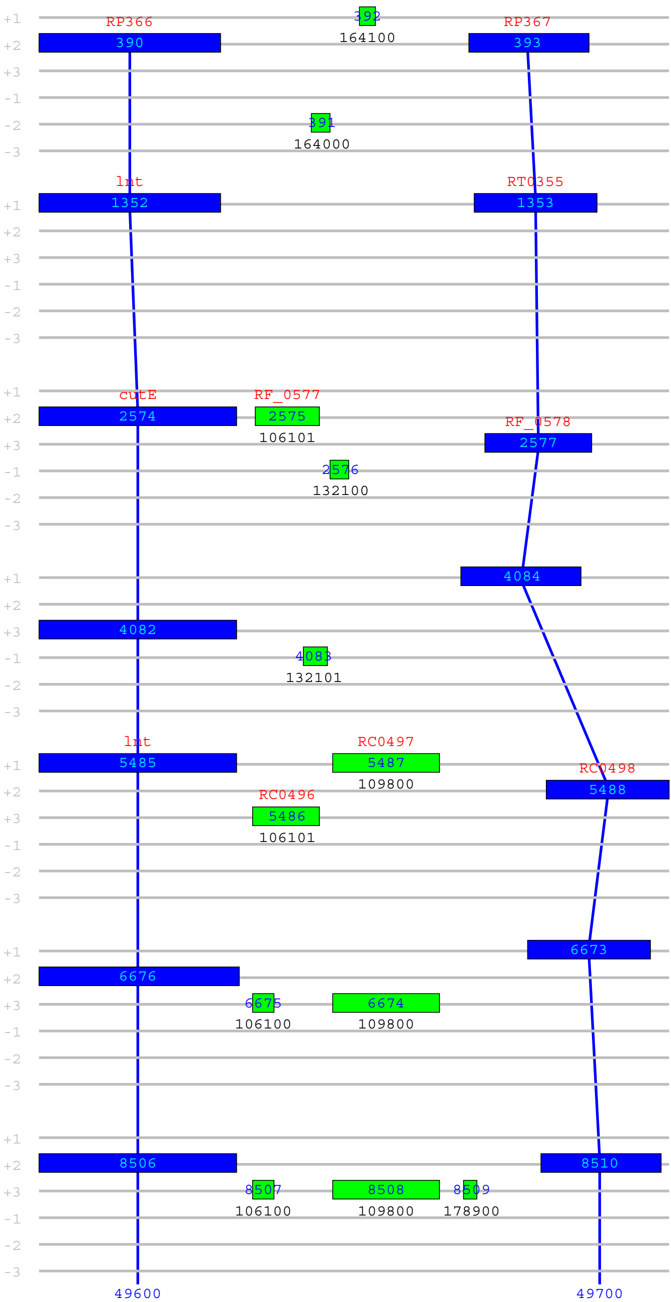

1 Rickettsia prowazekii str. Madrid E, complete genome
2 Rickettsia typhi str. wilmington, complete genome
3 Rickettsia felis URRWXCal2, complete genome
4 Rickettsia akari str. Hartford chromosome, whole genome shotgun sequence
5 Rickettsia conorii str. Malish 7, complete genome
6 Rickettsia sibirica 246 rsib_agncrt, whole genome shotgun sequence
7 Rickettsia rickettsii chromosome, whole genome shotgun sequence

Reg_id: 606

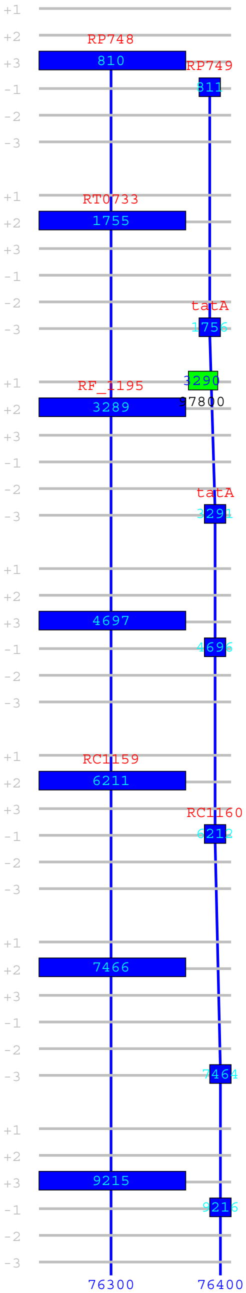

1 Rickettsia prowazekii str. Madrid E, complete genome
2 Rickettsia typhi str. wilmington, complete genome
3 Rickettsia felis URRWXCal2, complete genome
4 Rickettsia akari str. Hartford chromosome, whole genome shotgun sequence
5 Rickettsia conorii str. Malish 7, complete genome
6 Rickettsia sibirica 246 rsib_agncrt, whole genome shotgun sequence
7 Rickettsia rickettsii chromosome, whole genome shotgun sequence

Reg_id: 609

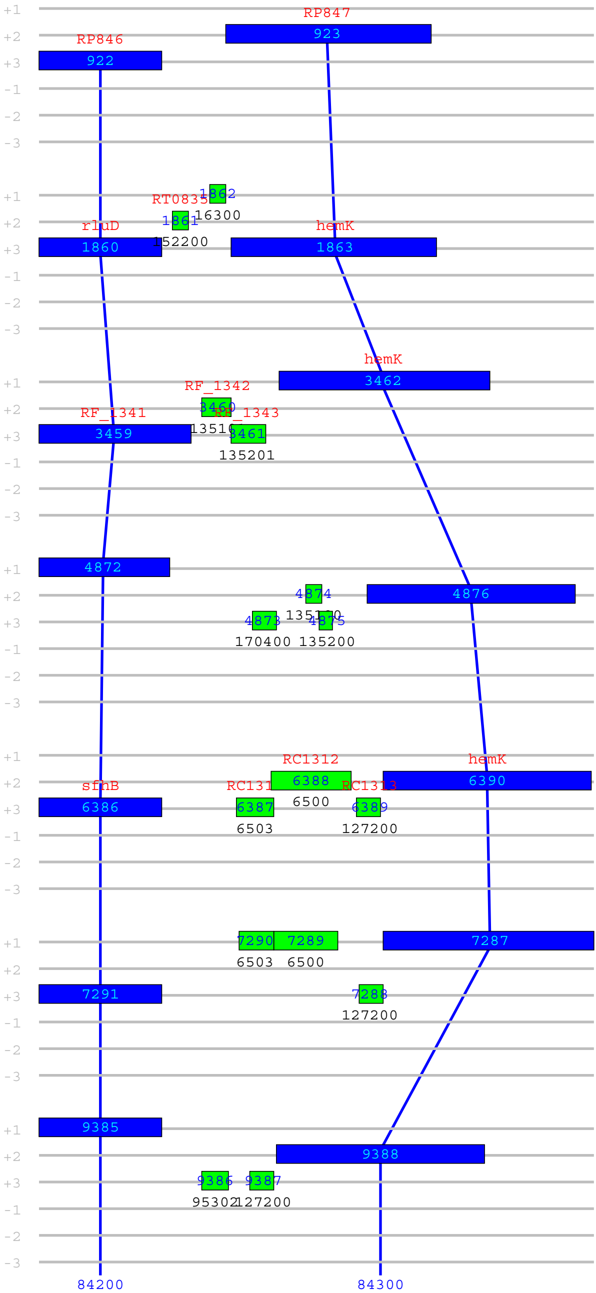

1 Rickettsia prowazekii str. Madrid E, complete genome
2 Rickettsia typhi str. wilmington, complete genome
3 Rickettsia felis URRWXCal2, complete genome
4 Rickettsia akari str. Hartford chromosome, whole genome shotgun sequence
5 Rickettsia conorii str. Malish 7, complete genome
6 Rickettsia sibirica 246 rsib_agncrt, whole genome shotgun sequence
7 Rickettsia rickettsii chromosome, whole genome shotgun sequence

Reg_id: 610

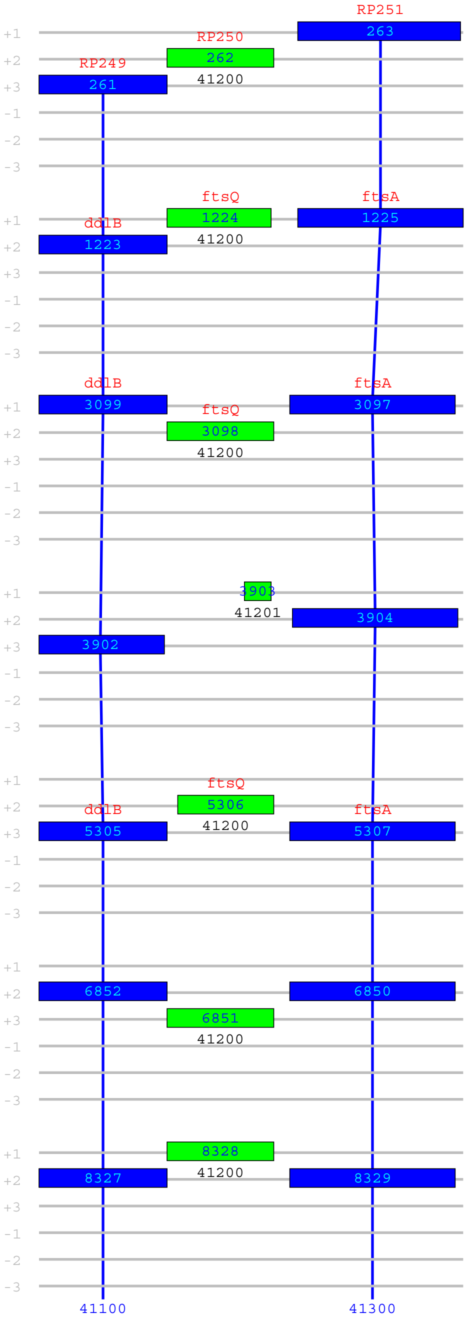

1 Rickettsia prowazekii str. Madrid E, complete genome
2 Rickettsia typhi str. wilmington, complete genome
3 Rickettsia felis URRWXCal2, complete genome
4 Rickettsia akari str. Hartford chromosome, whole genome shotgun sequence
5 Rickettsia conorii str. Malish 7, complete genome
6 Rickettsia sibirica 246 rsib_agncrt, whole genome shotgun sequence
7 Rickettsia rickettsii chromosome, whole genome shotgun sequence

Reg_id: 612

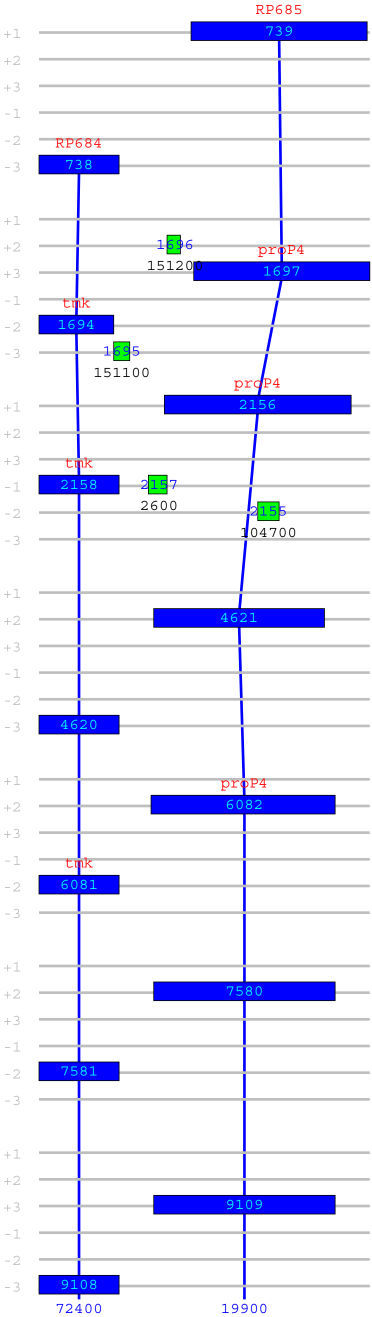

1 Rickettsia prowazekii str. Madrid E, complete genome
2 Rickettsia typhi str. wilmington, complete genome
3 Rickettsia felis URRWXCal2, complete genome
4 Rickettsia akari str. Hartford chromosome, whole genome shotgun sequence
5 Rickettsia conorii str. Malish 7, complete genome
6 Rickettsia sibirica 246 rsib_agncrt, whole genome shotgun sequence
7 Rickettsia rickettsii chromosome, whole genome shotgun sequence

Reg_id: 614

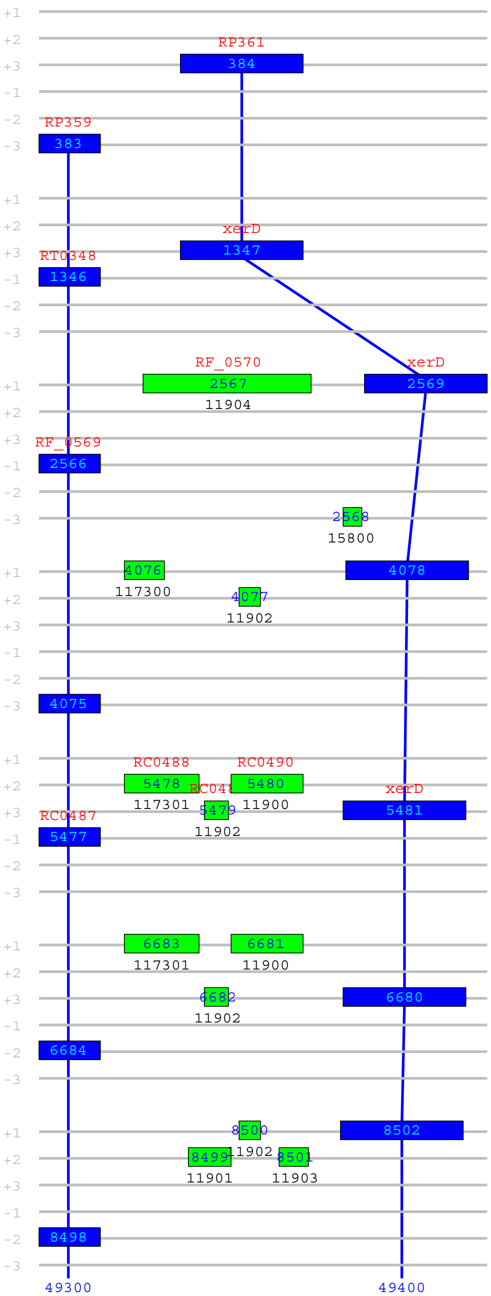

1 Rickettsia prowazekii str. Madrid E, complete genome
2 Rickettsia typhi str. wilmington, complete genome
3 Rickettsia felis URRWXCal2, complete genome
4 Rickettsia akari str. Hartford chromosome, whole genome shotgun sequence
5 Rickettsia conorii str. Malish 7, complete genome
6 Rickettsia sibirica 246 rsib_agncrt, whole genome shotgun sequence
7 Rickettsia rickettsii chromosome, whole genome shotgun sequence

Reg_id: 618

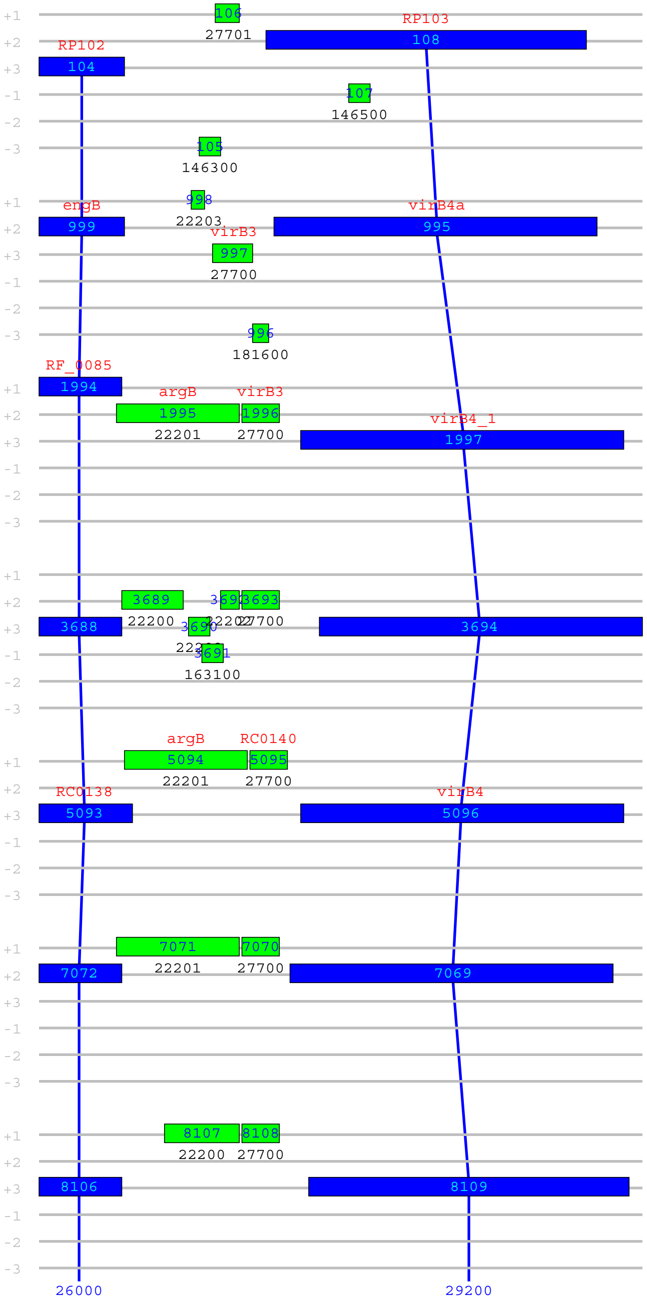

1 Rickettsia prowazekii str. Madrid E, complete genome
2 Rickettsia typhi str. wilmington, complete genome
3 Rickettsia felis URRWXCal2, complete genome
4 Rickettsia akari str. Hartford chromosome, whole genome shotgun sequence
5 Rickettsia conorii str. Malish 7, complete genome
6 Rickettsia sibirica 246 rsib_agncrt, whole genome shotgun sequence
7 Rickettsia rickettsii chromosome, whole genome shotgun sequence

Reg_id: 623

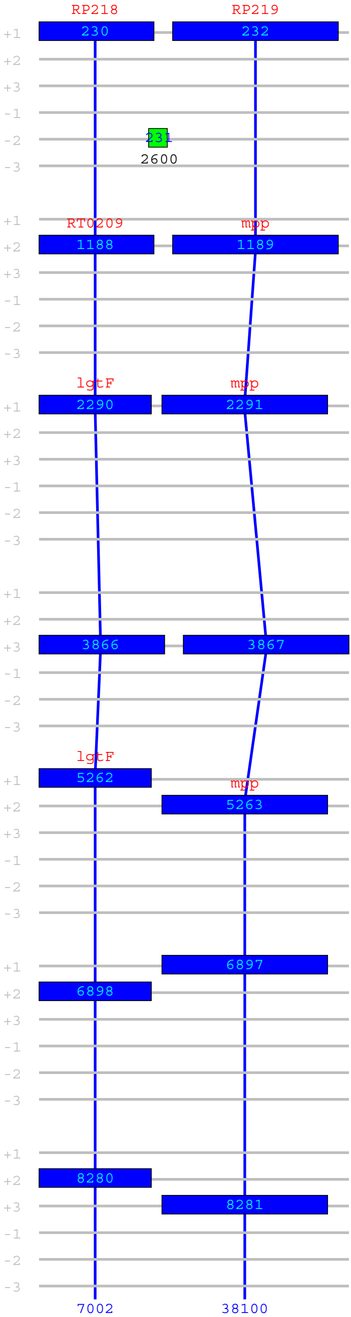

1 Rickettsia prowazekii str. Madrid E, complete genome
2 Rickettsia typhi str. wilmington, complete genome
3 Rickettsia felis URRWXCal2, complete genome
4 Rickettsia akari str. Hartford chromosome, whole genome shotgun sequence
5 Rickettsia conorii str. Malish 7, complete genome
6 Rickettsia sibirica 246 rsib_agncrt, whole genome shotgun sequence
7 Rickettsia rickettsii chromosome, whole genome shotgun sequence

Reg_id: 626

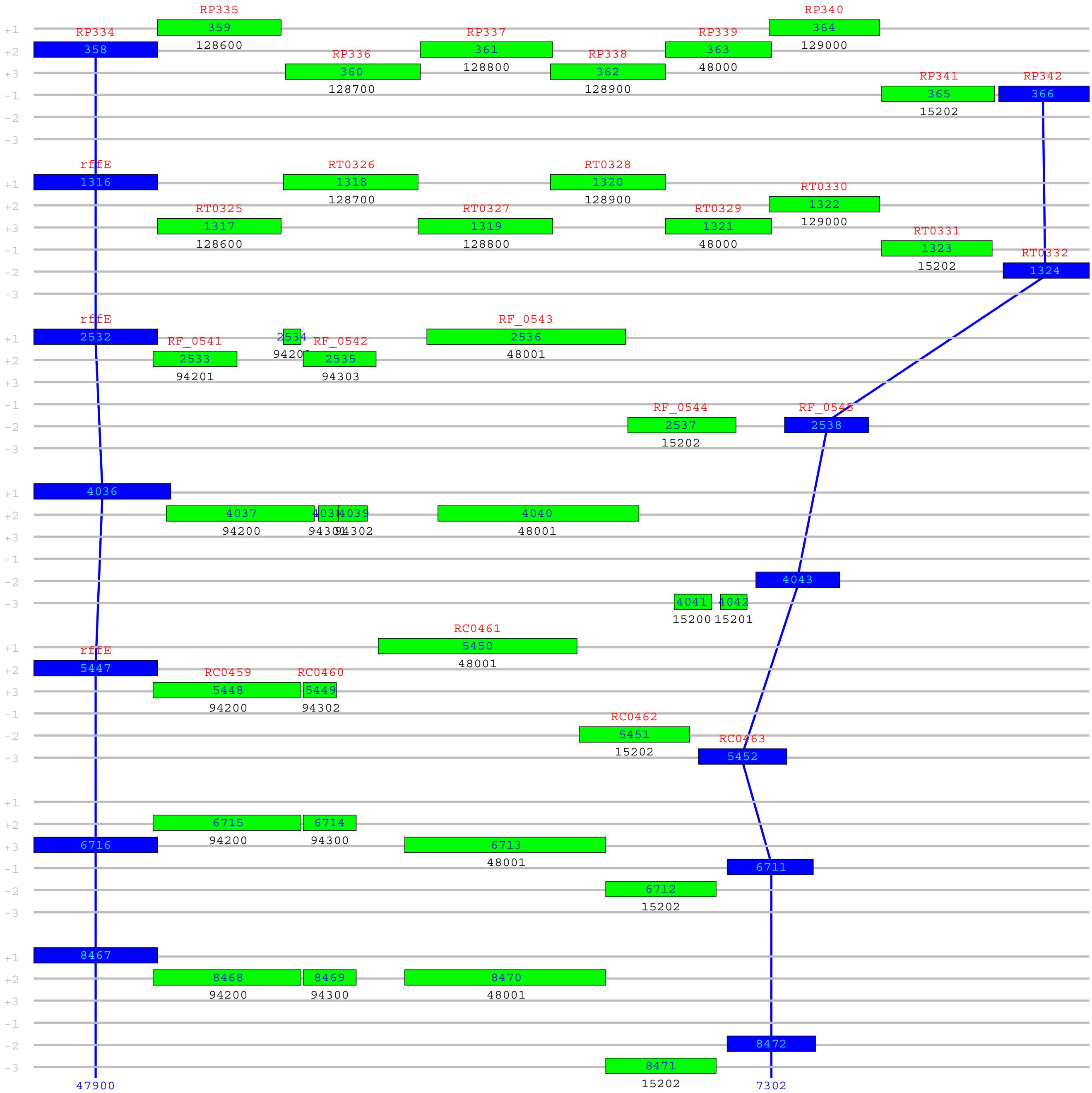

1 Rickettsia prowazekii str. Madrid E, complete genome
2 Rickettsia typhi str. wilmington, complete genome
3 Rickettsia felis URRWXCal2, complete genome
4 Rickettsia akari str. Hartford chromosome, whole genome shotgun sequence
5 Rickettsia conorii str. Malish 7, complete genome
6 Rickettsia sibirica 246 rsib_agncrt, whole genome shotgun sequence
7 Rickettsia rickettsii chromosome, whole genome shotgun sequence

Reg_id: 627

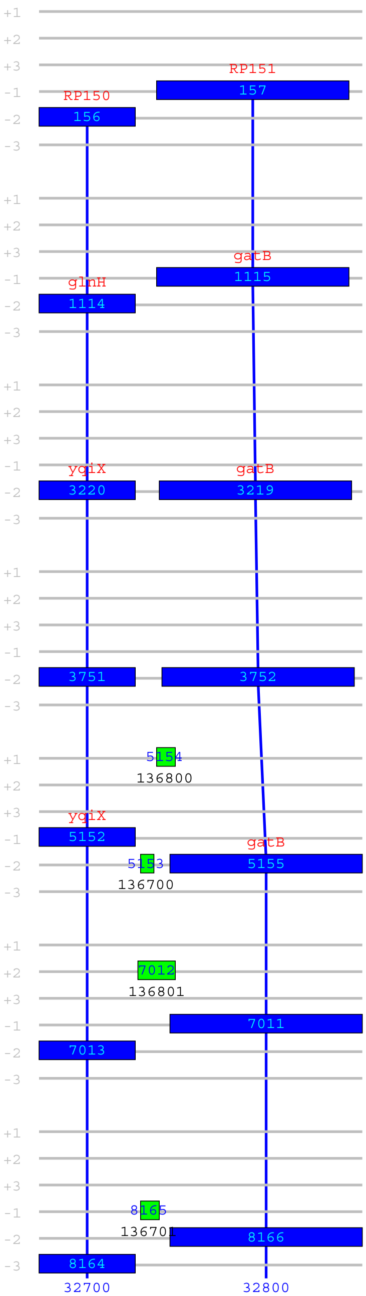

1 Rickettsia prowazekii str. Madrid E, complete genome
2 Rickettsia typhi str. wilmington, complete genome
3 Rickettsia felis URRWXCal2, complete genome
4 Rickettsia akari str. Hartford chromosome, whole genome shotgun sequence
5 Rickettsia conorii str. Malish 7, complete genome
6 Rickettsia sibirica 246 rsib_agncrt, whole genome shotgun sequence
7 Rickettsia rickettsii chromosome, whole genome shotgun sequence

Reg_id: 631

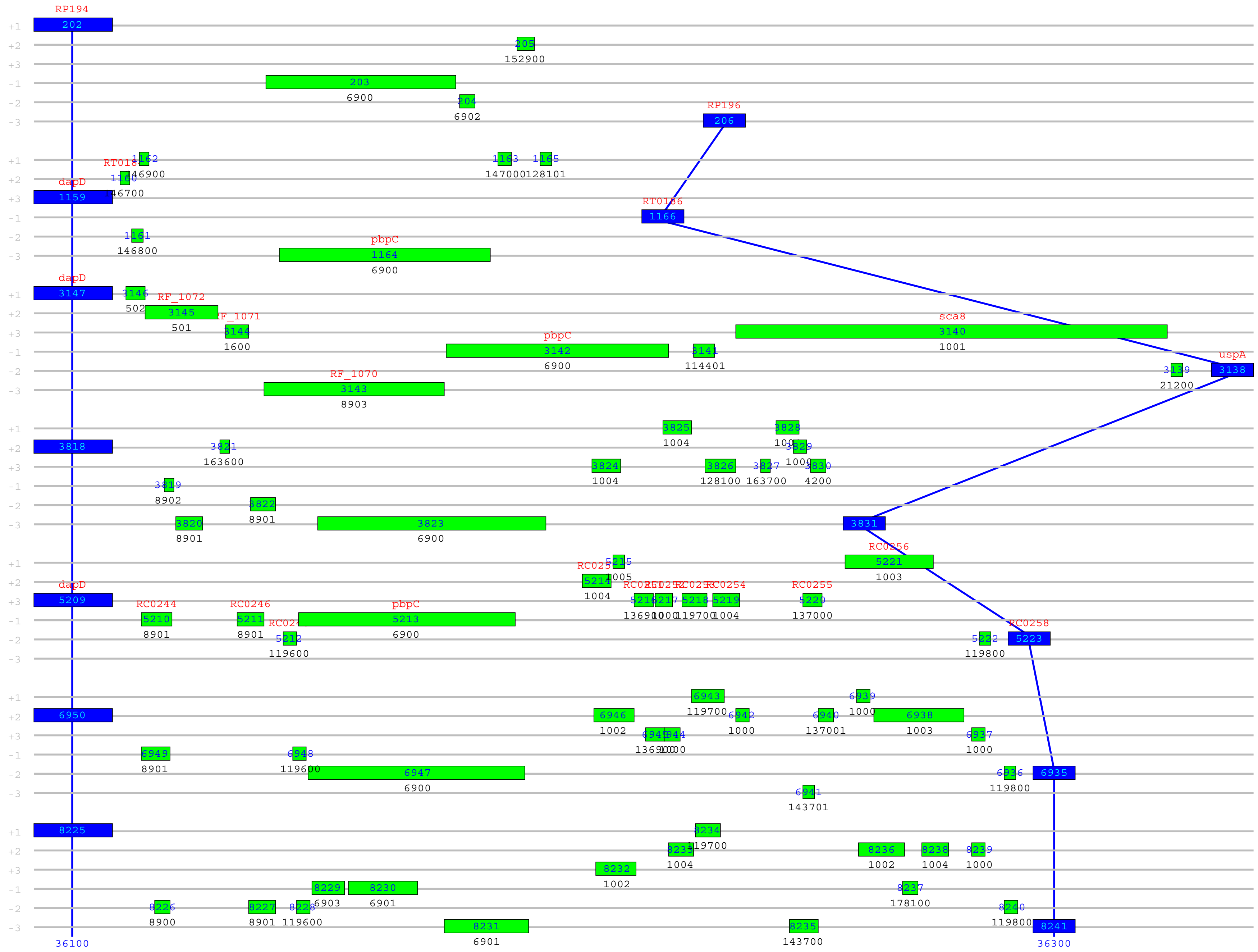

1 Rickettsia prowazekii str. Madrid E, complete genome
2 Rickettsia typhi str. wilmington, complete genome
3 Rickettsia felis URRWXCal2, complete genome
4 Rickettsia akari str. Hartford chromosome, whole genome shotgun sequence
5 Rickettsia conorii str. Malish 7, complete genome
6 Rickettsia sibirica 246 rsib_agncrt, whole genome shotgun sequence
7 Rickettsia rickettsii chromosome, whole genome shotgun sequence

Reg_id: 634

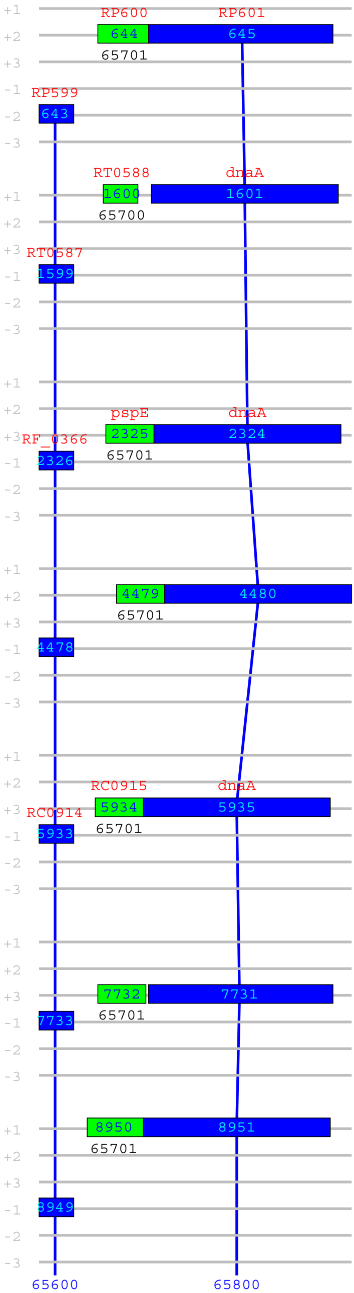

1 Rickettsia prowazekii str. Madrid E, complete genome
2 Rickettsia typhi str. wilmington, complete genome
3 Rickettsia felis URRWXCal2, complete genome
4 Rickettsia akari str. Hartford chromosome, whole genome shotgun sequence
5 Rickettsia conorii str. Malish 7, complete genome
6 Rickettsia sibirica 246 rsib_agncrt, whole genome shotgun sequence
7 Rickettsia rickettsii chromosome, whole genome shotgun sequence

Reg_id: 636

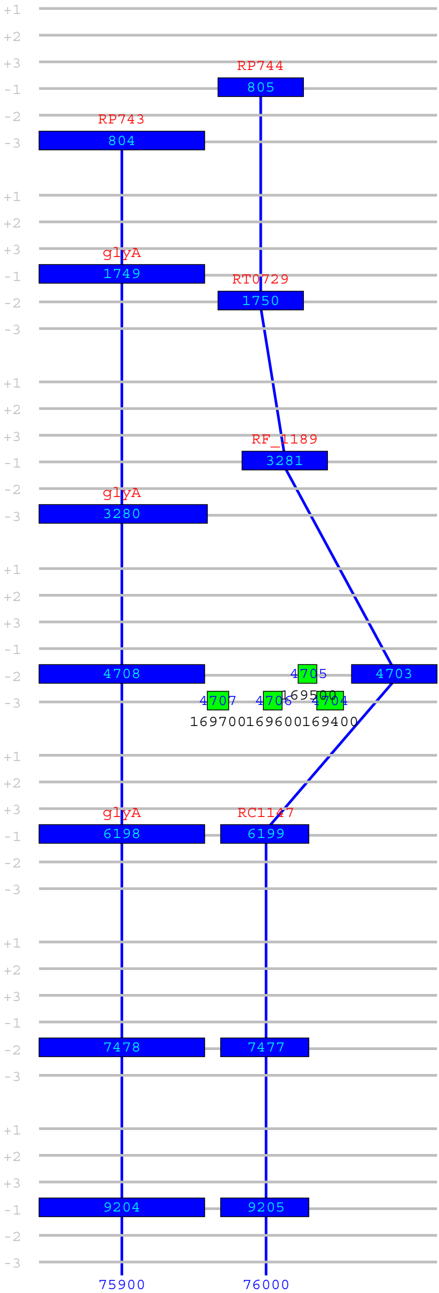

1 Rickettsia prowazekii str. Madrid E, complete genome
2 Rickettsia typhi str. wilmington, complete genome
3 Rickettsia felis URRWXCal2, complete genome
4 Rickettsia akari str. Hartford chromosome, whole genome shotgun sequence
5 Rickettsia conorii str. Malish 7, complete genome
6 Rickettsia sibirica 246 rsib_agncrt, whole genome shotgun sequence
7 Rickettsia rickettsii chromosome, whole genome shotgun sequence

Reg_id: 637

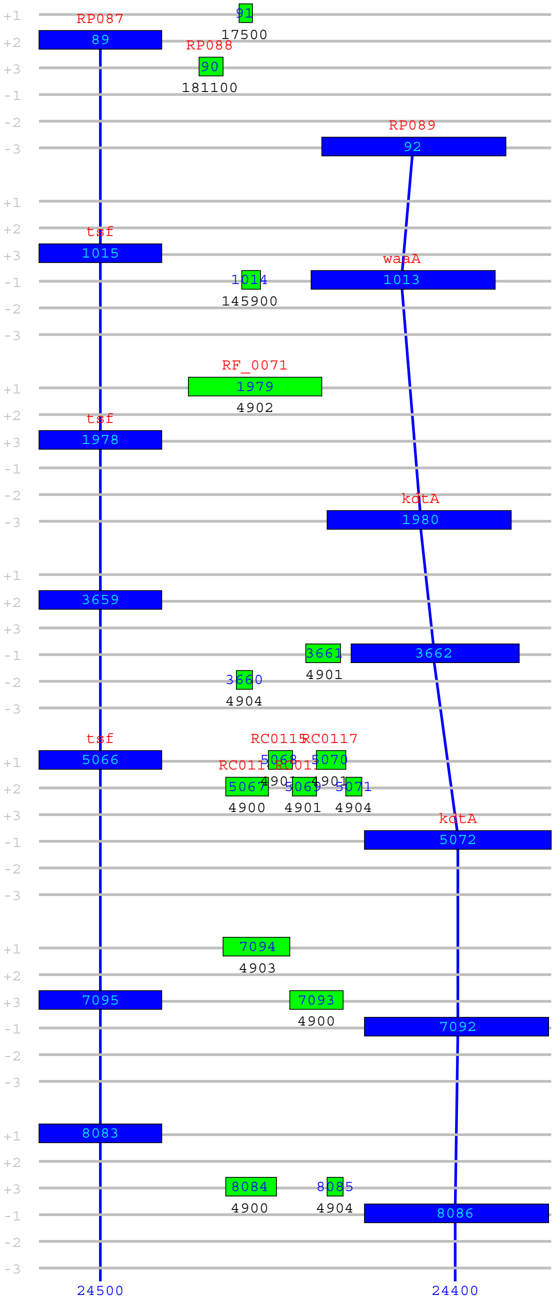

1 Rickettsia prowazekii str. Madrid E, complete genome
2 Rickettsia typhi str. wilmington, complete genome
3 Rickettsia felis URRWXCal2, complete genome
4 Rickettsia akari str. Hartford chromosome, whole genome shotgun sequence
5 Rickettsia conorii str. Malish 7, complete genome
6 Rickettsia sibirica 246 rsib_agncrt, whole genome shotgun sequence
7 Rickettsia rickettsii chromosome, whole genome shotgun sequence

Reg_id: 639

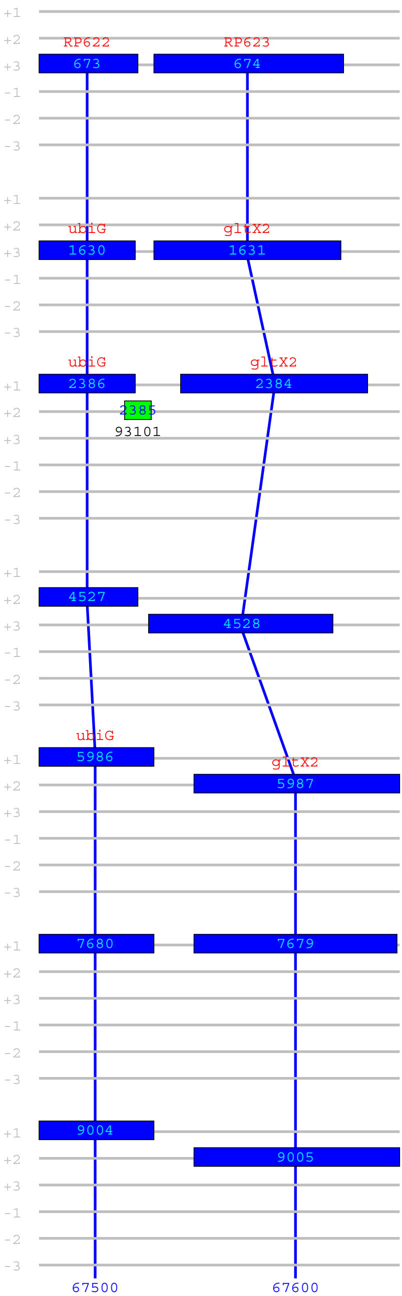

1 Rickettsia prowazekii str. Madrid E, complete genome
2 Rickettsia typhi str. wilmington, complete genome
3 Rickettsia felis URRWXCal2, complete genome
4 Rickettsia akari str. Hartford chromosome, whole genome shotgun sequence
5 Rickettsia conorii str. Malish 7, complete genome
6 Rickettsia sibirica 246 rsib_agncrt, whole genome shotgun sequence
7 Rickettsia rickettsii chromosome, whole genome shotgun sequence

Reg_id: 640

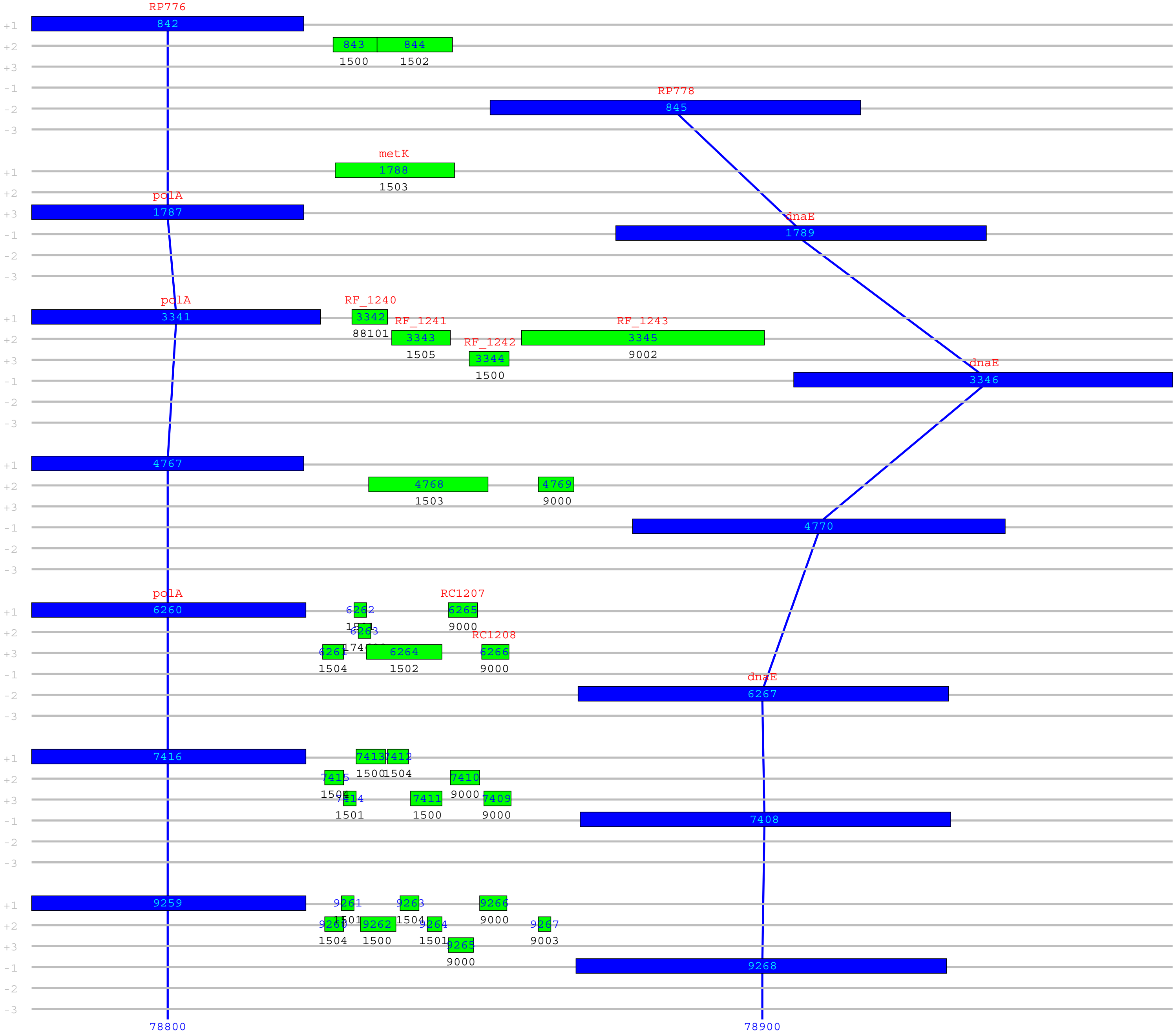

1 Rickettsia prowazekii str. Madrid E, complete genome
2 Rickettsia typhi str. wilmington, complete genome
3 Rickettsia felis URRWXCal2, complete genome
4 Rickettsia akari str. Hartford chromosome, whole genome shotgun sequence
5 Rickettsia conorii str. Malish 7, complete genome
6 Rickettsia sibirica 246 rsib_agncrt, whole genome shotgun sequence
7 Rickettsia rickettsii chromosome, whole genome shotgun sequence

Reg_id: 641

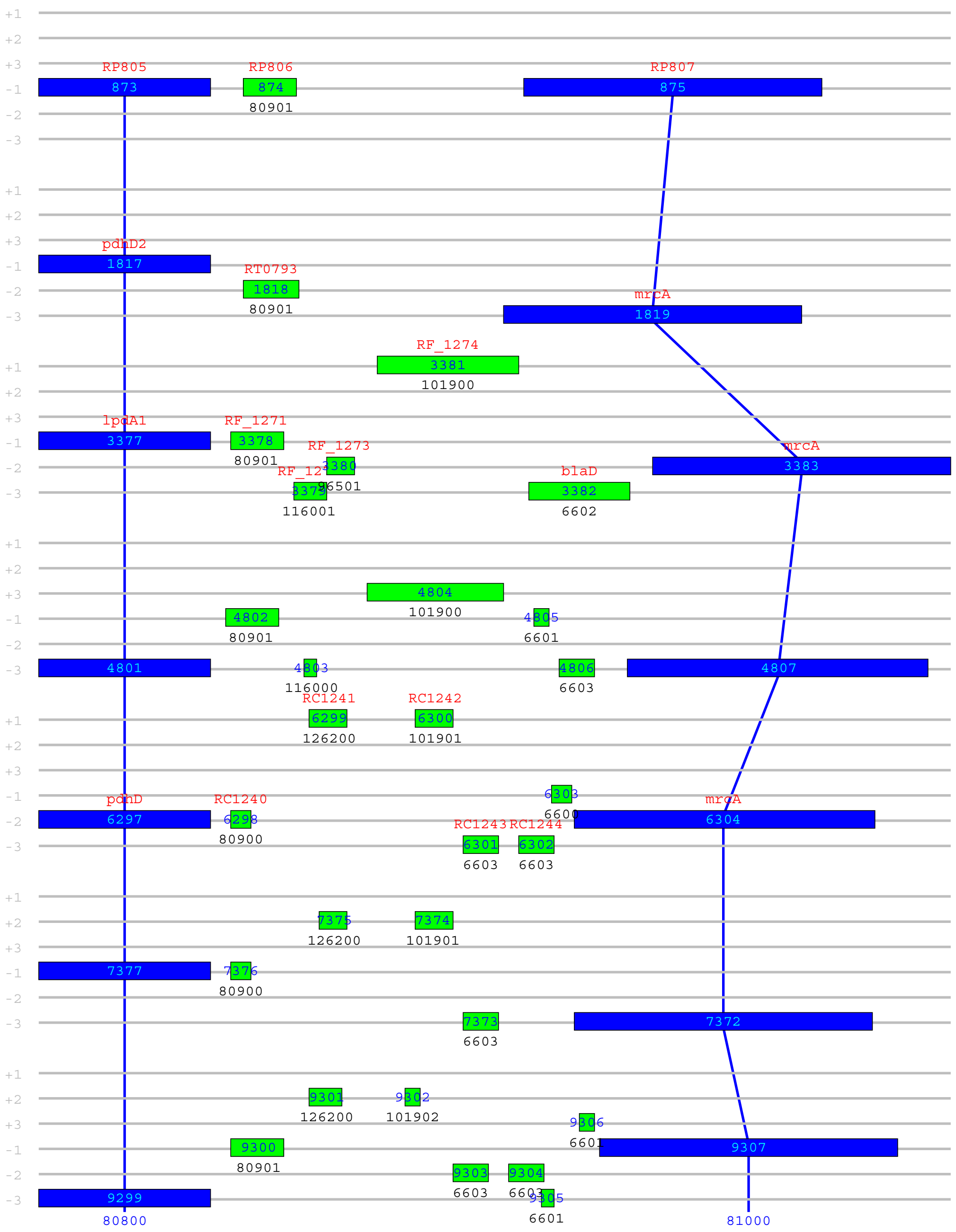

1 Rickettsia prowazekii str. Madrid E, complete genome
2 Rickettsia typhi str. wilmington, complete genome
3 Rickettsia felis URRWXCal2, complete genome
4 Rickettsia akari str. Hartford chromosome, whole genome shotgun sequence
5 Rickettsia conorii str. Malish 7, complete genome
6 Rickettsia sibirica 246 rsib_agncrt, whole genome shotgun sequence
7 Rickettsia rickettsii chromosome, whole genome shotgun sequence

Reg_id: 642

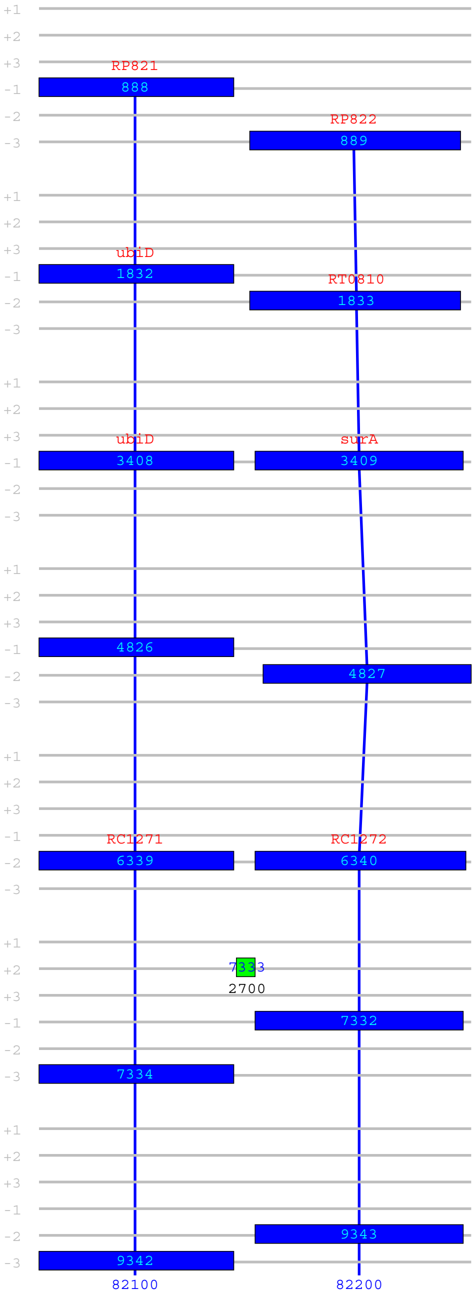

1 Rickettsia prowazekii str. Madrid E, complete genome
2 Rickettsia typhi str. wilmington, complete genome
3 Rickettsia felis URRWXCal2, complete genome
4 Rickettsia akari str. Hartford chromosome, whole genome shotgun sequence
5 Rickettsia conorii str. Malish 7, complete genome
6 Rickettsia sibirica 246 rsib_agncrt, whole genome shotgun sequence
7 Rickettsia rickettsii chromosome, whole genome shotgun sequence

Reg_id: 643

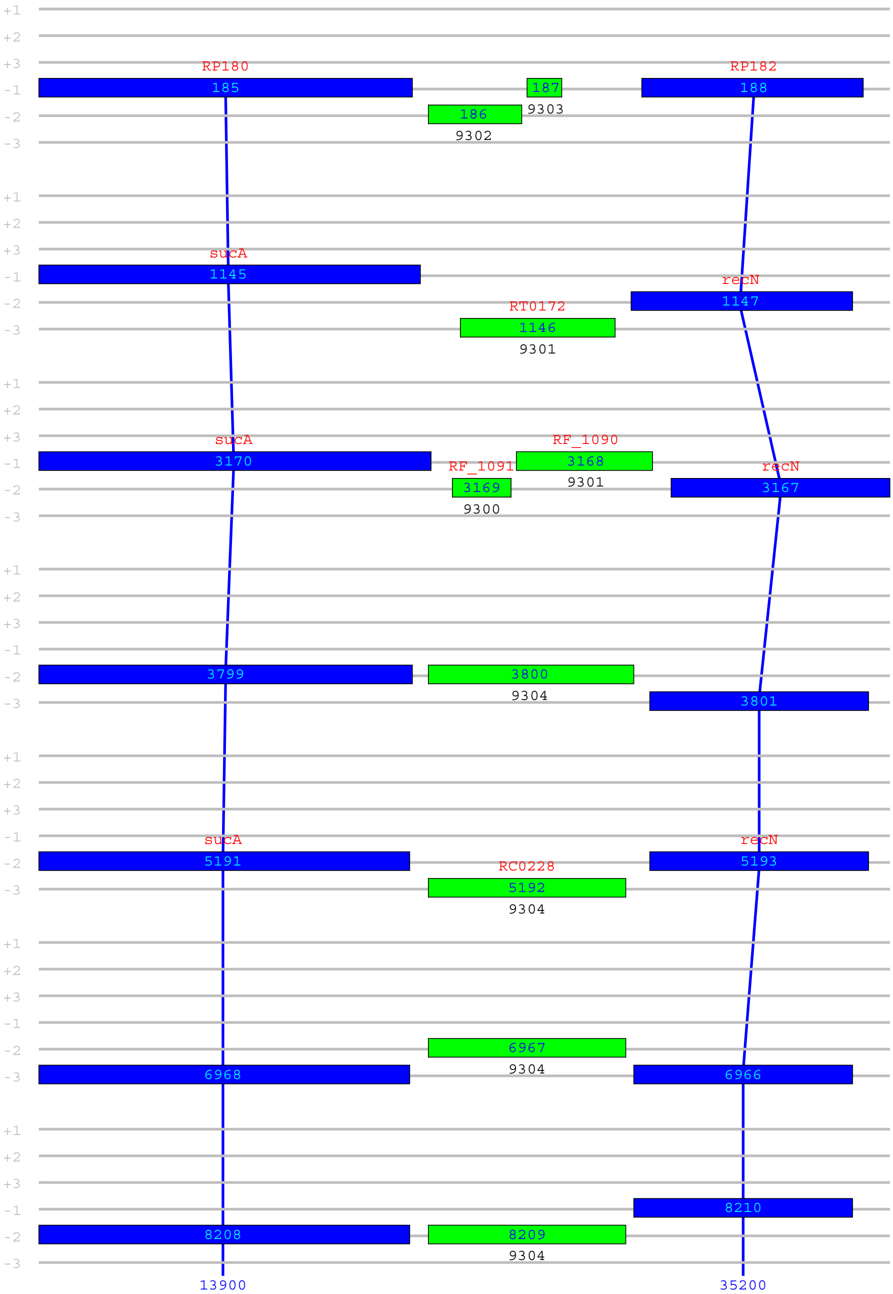

1 Rickettsia prowazekii str. Madrid E, complete genome
2 Rickettsia typhi str. wilmington, complete genome
3 Rickettsia felis URRWXCal2, complete genome
4 Rickettsia akari str. Hartford chromosome, whole genome shotgun sequence
5 Rickettsia conorii str. Malish 7, complete genome
6 Rickettsia sibirica 246 rsib_agncrt, whole genome shotgun sequence
7 Rickettsia rickettsii chromosome, whole genome shotgun sequence

Reg_id: 644

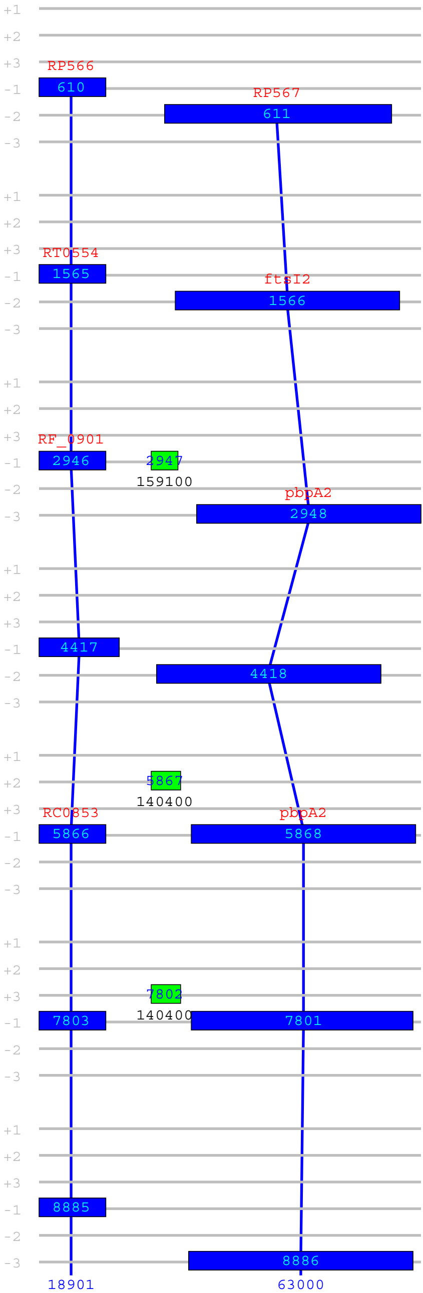

1 Rickettsia prowazekii str. Madrid E, complete genome
2 Rickettsia typhi str. wilmington, complete genome
3 Rickettsia felis URRWXCal2, complete genome
4 Rickettsia akari str. Hartford chromosome, whole genome shotgun sequence
5 Rickettsia conorii str. Malish 7, complete genome
6 Rickettsia sibirica 246 rsib_agncrt, whole genome shotgun sequence
7 Rickettsia rickettsii chromosome, whole genome shotgun sequence

Reg_id: 646

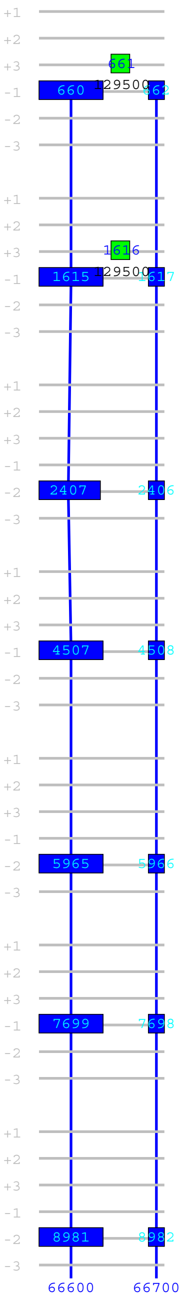

1 Rickettsia prowazekii str. Madrid E, complete genome
2 Rickettsia typhi str. wilmington, complete genome
3 Rickettsia felis URRWXCal2, complete genome
4 Rickettsia akari str. Hartford chromosome, whole genome shotgun sequence
5 Rickettsia conorii str. Malish 7, complete genome
6 Rickettsia sibirica 246 rsib_agncrt, whole genome shotgun sequence
7 Rickettsia rickettsii chromosome, whole genome shotgun sequence

Reg_id: 647

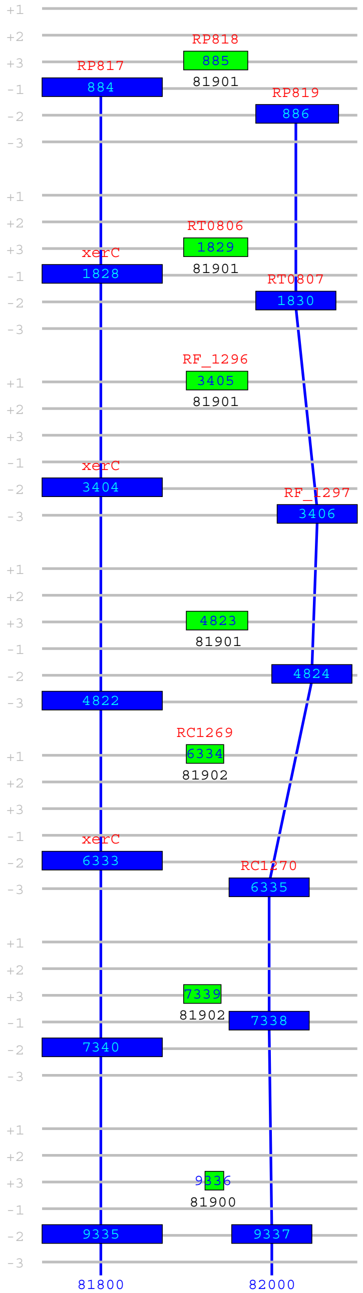

1 Rickettsia prowazekii str. Madrid E, complete genome
2 Rickettsia typhi str. wilmington, complete genome
3 Rickettsia felis URRWXCal2, complete genome
4 Rickettsia akari str. Hartford chromosome, whole genome shotgun sequence
5 Rickettsia conorii str. Malish 7, complete genome
6 Rickettsia sibirica 246 rsib_agncrt, whole genome shotgun sequence
7 Rickettsia rickettsii chromosome, whole genome shotgun sequence

Reg_id: 648

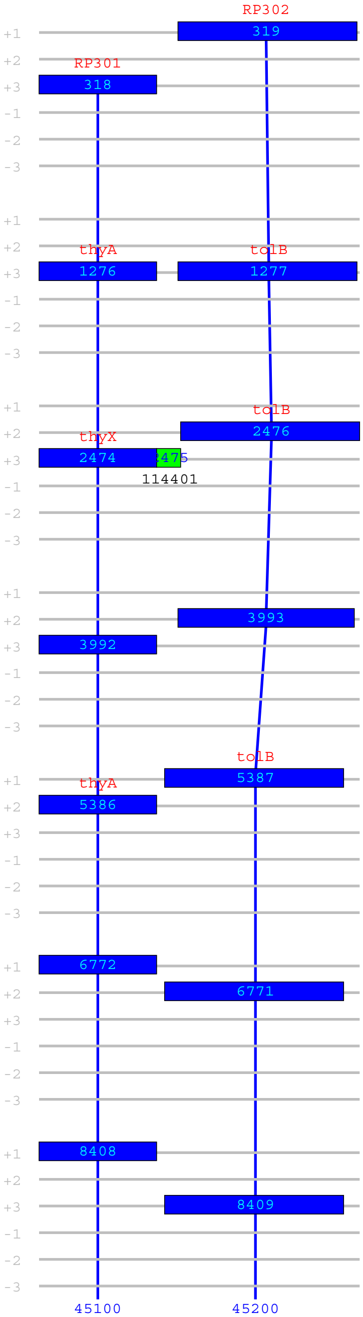

1 Rickettsia prowazekii str. Madrid E, complete genome
2 Rickettsia typhi str. wilmington, complete genome
3 Rickettsia felis URRWXCal2, complete genome
4 Rickettsia akari str. Hartford chromosome, whole genome shotgun sequence
5 Rickettsia conorii str. Malish 7, complete genome
6 Rickettsia sibirica 246 rsib_agncrt, whole genome shotgun sequence
7 Rickettsia rickettsii chromosome, whole genome shotgun sequence

Reg_id: 653

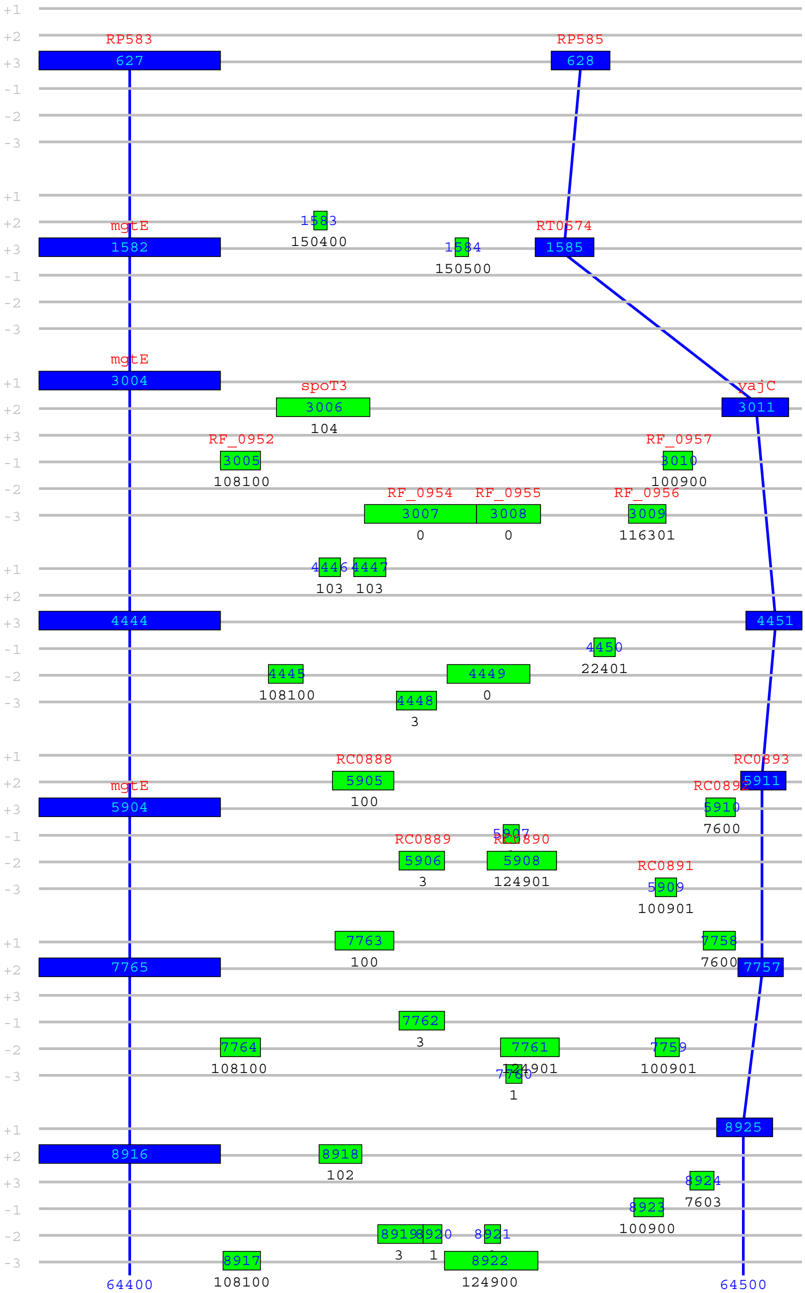

1 Rickettsia prowazekii str. Madrid E, complete genome
2 Rickettsia typhi str. wilmington, complete genome
3 Rickettsia felis URRWXCal2, complete genome
4 Rickettsia akari str. Hartford chromosome, whole genome shotgun sequence
5 Rickettsia conorii str. Malish 7, complete genome
6 Rickettsia sibirica 246 rsib_agncrt, whole genome shotgun sequence
7 Rickettsia rickettsii chromosome, whole genome shotgun sequence

Reg_id: 655

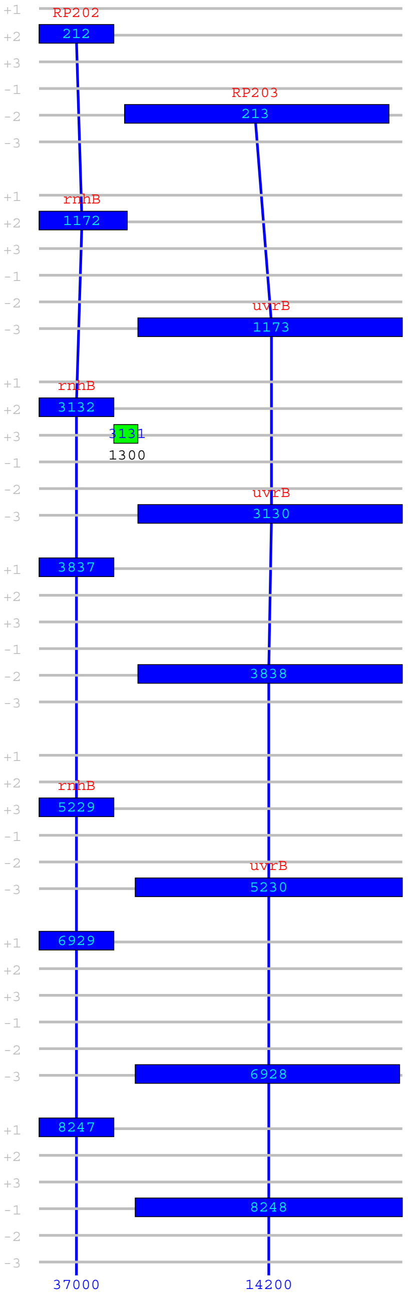

1 Rickettsia prowazekii str. Madrid E, complete genome
2 Rickettsia typhi str. wilmington, complete genome
3 Rickettsia felis URRWXCal2, complete genome
4 Rickettsia akari str. Hartford chromosome, whole genome shotgun sequence
5 Rickettsia conorii str. Malish 7, complete genome
6 Rickettsia sibirica 246 rsib_agncrt, whole genome shotgun sequence
7 Rickettsia rickettsii chromosome, whole genome shotgun sequence

Reg_id: 657

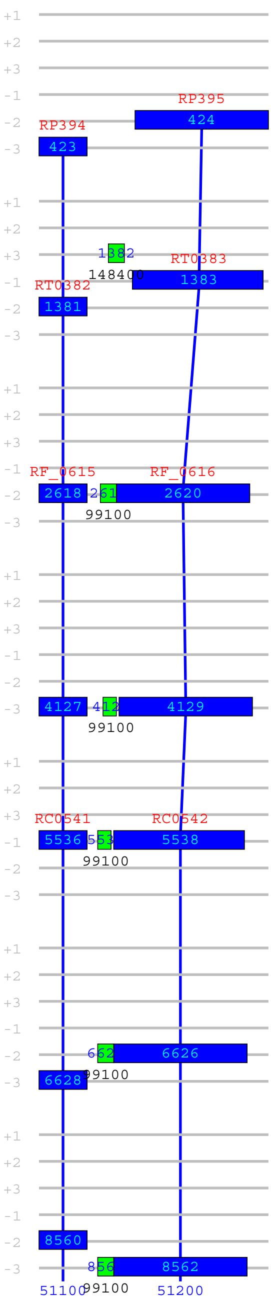

Supplement: Additional data file 1 — Presented is the visualization of all variable segments containing ORFs in the seven Rickettsia genomes using GenComp. [file gb-2008-9-2-r42-S1.pdf]
