# Supplementary material for: Visualization of pseudogenes in intracellular bacteria reveals the different tracks to gene destruction
Source: Genome Biol. 2008 Feb 26;9(2):R42. doi: 10.1186/gb-2008-9-2-r42 (PMC2374718; doi:10.1186/gb-2008-9-2-r42)
Supplement: Additional data file 2 — Presented is the distribution of individual strain-variable ORFs into species sets, phylogroups, and functional categories. Also shown is the most similar sequence outside the Rickettsiales. [file gb-2008-9-2-r42-S2.pdf]

reg_id	cluster_head	chr_id_list	Class	R_order	O_order	W_order	R_protdist	O_protdist	W_protdis	top_hit_group	top_hit_dist	top_hit_code	top_gene	top_genus	top_desc	second_group	second_genus
640	15	['1', '2', '3', '4', '5', '6',	None	-	-	-	-	-	-	Gammaproteobacteri	0,410476	H	-	Thiomicrospi	Methionine adenosyltransferase	-	-
626	480	['1', '2', '3', '4', '5', '6',	R	1	-	-	0,269062	-	-	Alphaproteobacteria	0,269062	M	-	Rickettsia	Glycosyltransferase	Deltaproteobacteria	Geobacter
152	68	['1', '2', '3', '4', '5', '6',	R	1	-	-	0,073905	-	-	Alphaproteobacteria	0,073905	C	-	Rickettsia	Phosphate acetyltransferase Pta	Betaproteobacteria	Dechloromonas
73	6	['1', '2', '3', '4', '5', '6',	R	1	-	-	0,543469	-	-	Alphaproteobacteria	0,543469	-	-	Rickettsia	hypothetical protein	-	-
641	809	['1', '2', '3', '4', '5', '6',	R	1	-	-	0,216468	-	-	Alphaproteobacteria	0,216468	P	-	Rickettsia	Strees induced DNA-binding Dps family proteir	Gammaproteobacteria	Legionella
113	131	['1', '2', '3', '4', '5', '6',	R	1	-	-	0,052257	-	-	Alphaproteobacteria	0,052257	IQR	phbB	Rickettsia	Acetoacetyl-CoA reductase	Gammaproteobacteria	Colwellia
134	14	['1', '2', '3', '4', '5', '6',	R	1	-	-	0,563912	-	-	Alphaproteobacteria	0,563912	-	-	Rickettsia	hypothetical protein	-	-
647	819	['1', '2', '3', '4', '5', '6',	R	1	-	-	0,234048	-	-	Alphaproteobacteria	0,234048	-	-	Rickettsia	Phasin family protein	-	-
404	42	['1', '2', '3', '4', '5', '6',	R	1	-	-	1,233456	-	-	Alphaproteobacteria	1,233456	-	sca2	Rickettsia	Cell surface antigen Sca2	Bacilli	Streptococcus
588	794	['1', '2', '3', '4', '5', '6',	R	1	-	-	0,159003	-	-	Alphaproteobacteria	0,159003	P	terC	Rickettsia	Tellurium resistance protein TerC	Alphaproteobacteria	Agrobacterium
497	84	['1', '2', '3', '4', '5', '6',	R	1	-	-	0,119101	-	-	Alphaproteobacteria	0,119101	I	-	Rickettsia	Poly(3-hydroxyalkanoate) synthetase	Gammaproteobacteria	Legionella
588	206	['1', '2', '3', '4', '5', '6',	R	1	-	-	0,226816	-	-	Alphaproteobacteria	0,226816	-	-	Rickettsia	hypothetical protein	-	-
338	140	['1', '2', '3', '4', '5', '6',	R	1	-	-	0,193703	-	-	Alphaproteobacteria	0,193703	O	-	Rickettsia	Heat shock protease	Deltaproteobacteria	Syntrophus
116	177	['1', '2', '3', '4', '5', '6',	R	1	-	-	0,192964	-	-	Alphaproteobacteria	0,192964	S	-	Rickettsia	hypothetical protein	-	-
152	43	['1', '2', '3', '4', '5', '6',	R	1	-	-	0,170826	-	-	Alphaproteobacteria	0,170826	C	ackA	Rickettsia	Acetate kinase	Betaproteobacteria	Burkholderia
234	11	['1', '2', '3', '4', '5', '6',	R	1	-	-	0,157254	-	-	Alphaproteobacteria	0,157254	V	acrF	Rickettsia	Hydrophobe/amphiphile efflux-1 (HAE1) famil	Deltaproteobacteria	Desulfotalea
322	103	['1', '2', '3', '4', '5', '6',	R	1	-	-	1,119039	-	-	Alphaproteobacteria	1,119039	-	-	Rickettsia	hypothetical protein	-	-
204	200	['1', '2', '3', '4', '5', '6',	R	1	-	-	0,340883	-	-	Alphaproteobacteria	0,340883	I	phbC	Rickettsia	Poly-beta-hydroxybutyrate polymerase	Euryarchaeota	Haloarcula
551	31	['1', '2', '3', '4', '5', '6',	R	1	-	-	0,43275	-	-	Alphaproteobacteria	0,43275	R	rssA	Rickettsia	Putative esterase	Bacteroidetes/Chlorobi g	Porphyromonas
289	155	['1', '2', '3', '4', '5', '6',	R	1	-	-	0,950442	-	-	Alphaproteobacteria	0,950442	-	-	Rickettsia	hypothetical protein	-	-
539	309	['1', '2', '3', '4', '5', '6',	R	1	-	-	0,221604	-	-	Alphaproteobacteria	0,221604	U	secE	Rickettsia	Preprotein translocase SecE subunit	-	-
599	47	['1', '2', '3', '4', '5', '6',	R	1	-	-	0,182057	-	-	Alphaproteobacteria	0,182057	I	-	Rickettsia	Poly-beta-hydroxyalkanoate depolymerase	Alphaproteobacteria	Magnetospirillum
489	28	['1', '2', '3', '4', '5', '6',	R	1	-	-	0,951488	-	-	Alphaproteobacteria	0,951488	QR	-	Rickettsia	Putative methyltransferase	Alphaproteobacteria	Bradyrhizobium
71	718	['1', '2', '3', '4', '5', '6',	R	1	-	-	0,35386	-	-	Alphaproteobacteria	0,35386	M	ostA	Rickettsia	Organic solvent tolerance protein-like protein	Alphaproteobacteria	Magnetospirillum
289	99	['1', '2', '3', '4', '5', '6',	R	1	-	-	0,704326	-	-	Alphaproteobacteria	0,704326	-	-	Rickettsia	hypothetical protein	-	-
626	152	['1', '2', '3', '4', '5', '6',	R	1	-	-	0,283262	-	-	Alphaproteobacteria	0,283262	M	-	Rickettsia	Efflux transporter, RND family, MFP subunit	Gammaproteobacteria	Coxiella
631	69	['1', '2', '3', '4', '5', '6',	R	1	-	-	0,302436	-	-	Alphaproteobacteria	0,302436	M	pbpC	Rickettsia	Bifunctional penicillin-binding protein 1C	Gammaproteobacteria	Legionella
366	37	['1', '2', '3', '4', '5', '6',	R	1	-	-	0,422736	-	-	Alphaproteobacteria	0,422736	M	-	Rickettsia	LPS biosynthesis protein	Actinobacteria	Bifidobacterium
519	174	['1', '2', '3', '4', '5', '6',	R	1	-	-	0,472881	-	-	Alphaproteobacteria	0,472881	M	rfaJ	Rickettsia	Lipopolysaccharide 1,2-glucosyltransferase Rf	Gammaproteobacteria	Escherichia
303	414	['1', '2', '3', '4', '5', '6',	R	1	-	-	0,283093	-	-	Alphaproteobacteria	0,283093	UO	-	Rickettsia	Membrane protein	-	-
634	657	['1', '2', '3', '4', '5', '6',	R	1	-	-	0,441028	-	-	Alphaproteobacteria	0,441028	P	pspE	Rickettsia	Rhodanese-related sulfurtransferase	Actinobacteria	Mycobacterium
102	94	['1', '2', '3', '4', '5', '6',	RO	1	2	-	0,225911	1,501419	-	Alphaproteobacteria	0,225911	V	pbpE	Rickettsia	Penicillin-binding protein 4*	Bacilli	Bacillus
460	79	['1', '2', '3', '4', '5', '6',	RO	1	2	-	0,248836	0,882791	-	Alphaproteobacteria	0,248836	J	truB	Rickettsia	tRNA pseudouridine synthase B	Alphaproteobacteria	Ehrlichia
534	176	['1', '2', '3', '4', '5', '6',	RO	1	2	-	0,838136	2,458572	-	Alphaproteobacteria	0,838136	-	sca4	Rickettsia	Cell surface antigen Sca4	-	-
598	349	['1', '2', '3', '4', '5', '6',	RO	1	2	-	0,213078	0,960595	-	Alphaproteobacteria	0,213078	P	cutA	Rickettsia	Periplasmic divalent cation tolerance protein	Crenarchaeota	Sulfolobus
87	184	['1', '2', '3', '4', '5', '6',	RO	1	10	-	0,274016	1,259782	-	Alphaproteobacteria	0,274016	S	-	Rickettsia	hypothetical protein	-	-
5	4	['1', '2', '3', '4', '5', '6',	RO	33	38	-	2,543228	2,599967	-	Alphaproteobacteria	1,44751	E	-	Zymomonas	prolyl oligopeptidase family protein	-	-
434	59	['1', '2', '3', '4', '5', '6',	RO	1	2	-	0,705641	2,225706	-	Alphaproteobacteria	0,705641	-	-	Rickettsia	hypothetical protein	-	-
234	344	['1', '2', '3', '4', '5', '6',	RO	1	2	-	0,267996	0,705977	-	Alphaproteobacteria	0,267996	-	hupA	Rickettsia	DNA-binding protein HU	Gammaproteobacteria	Vibrio
234	345	['1', '2', '3', '4', '5', '6',	RO	1	2	-	0,219944	1,366569	-	Alphaproteobacteria	0,219944	L	holB	Rickettsia	DNA polymerase III delta subunit	-	-
368	135	['1', '2', '3', '4', '5', '6',	RO	2	1	-	0,978952	0,941406	-	Alphaproteobacteria	0,941406	-	-	Orientia tsut	-	-	-
181	863	['1', '2', '3', '4', '5', '6',	RO	1	2	-	0,189996	1,539793	-	Alphaproteobacteria	0,189996	-	holC	Rickettsia	DNA polymerase III chi subunit HolC	-	-
129	195	['1', '2', '3', '4', '5', '6',	RO	1	2	-	0,12221	0,526603	-	Alphaproteobacteria	0,12221	J	rph	Rickettsia	Ribonuclease PH	Betaproteobacteria	Chromobacterium
398	83	['1', '2', '3', '4', '5', '6',	RO	1	11	-	0,256699	1,656705	-	Alphaproteobacteria	0,256699	-	ampG3	Rickettsia	AmpG	-	-
442	179	['1', '2', '3', '4', '5', '6',	RO	1	2	-	0,19677	0,827146	-	Alphaproteobacteria	0,19677	UO	sppA2	Rickettsia	Signal peptide peptidase SppA, 36K type	Alphaproteobacteria	Ehrlichia
160	330	['1', '2', '3', '4', '5', '6',	RO	1	6	-	0,233653	1,188383	-	Alphaproteobacteria	0,233653	J	gatC	Rickettsia	Glutamyl-tRNA(Gln) amidotransferase subunit	-	-
139	133	['1', '2', '3', '4', '5', '6',	ROW	1	2	20	0,156085	0,683255	1,0229	Alphaproteobacteria	0,156085	R	-	Rickettsia	Putative sulfurtransferase	-	-
252	492	['1', '2', '3', '4', '5', '6',	ROW	1	2	13	0,083387	0,417	0,67581	Alphaproteobacteria	0,083387	C	nuoA	Rickettsia	NADH dehydrogenase I chain A	-	-
618	277	['1', '2', '3', '4', '5', '6',	ROW	1	3	2	0,065046	0,872903	0,80557	Alphaproteobacteria	0,065046	U	virB3	Rickettsia	VirB3	-	-
394	141	['1', '2', '3', '4', '5', '6',	ROW	1	2	5	0,170939	0,734029	0,87443	Alphaproteobacteria	0,170939	C	coxC	Rickettsia	Cytochrome c oxidase subunit III	-	-
551	613	['1', '2', '3', '4', '5', '6',	ROW	1	9	44	0,257241	0,758604	0,95248	Alphaproteobacteria	0,257241	P	sodB	Rickettsia	Superoxide dismutase	-	-
136	194	['1', '2', '3', '4', '5', '6',	ROW	1	2	4	0,206389	1,255057	1,45208	Alphaproteobacteria	0,206389	P	znuB	Rickettsia	Zinc/manganese ABC transporter permease pr	-	-
302	107	['1', '2', '3', '4', '5', '6',	ROW	1	2	4	0,229153	0,79495	1,02813	Alphaproteobacteria	0,229153	R	ubiB	Rickettsia	2-polyprenylphenol 6-hydroxylase	-	-
314	832	['1', '2', '3', '4', '5', '6',	ROW	1	2	35	0,133914	0,85981	1,28331	Alphaproteobacteria	0,133914	P	znuC	Rickettsia	Zinc ABC transporter ATP-binding protein	-	-
301	110	['1', '2', '3', '4', '5', '6',	ROW	1	2	12	0,253329	1,32219	1,87456	Alphaproteobacteria	0,253329	S	surf1	Rickettsia	Surfeit locus protein 1	-	-
60	435	['1', '2', '3', '4', '5', '6',	ROW	1	2	4	0,253811	1,351517	1,41621	Alphaproteobacteria	0,253811	PC	nuoL2	Rickettsia	NADH dehydrogenase I chain L	-	-
100	564	['1', '2', '3', '4', '5', '6',	ROW	1	4	5	0,292292	1,763197	1,76416	Alphaproteobacteria	0,292292	-	-	Rickettsia	5-Formyltetrahydrofolate cyclo-ligase	-	-
143	32	['1', '2', '3', '4', '5', '6',	ROW	1	2	9	0,107452	1,210841	1,33369	Alphaproteobacteria	0,107452	-	proP6	Rickettsia	Proline/betaine transporter	-	-
483	518	['1', '2', '3', '4', '5', '6',	ROW	1	2	9	0,126549	0,590224	0,91787	Alphaproteobacteria	0,126549	C	coxB	Rickettsia	Cytochrome c oxidase polypeptide II	-	-
267	76	['1', '2', '3', '4', '5', '6',	ROW	1	5	3	0,282442	1,071299	1,02449	Alphaproteobacteria	0,282442	M	rlpA	Rickettsia	Rare lipoprotein A precursor	-	-
171	663	['1', '2', '3', '4', '5', '6',	ROW	1	2	6	0,046094	0,403119	0,50548	Alphaproteobacteria	0,046094	J	rpmI	Rickettsia	50S ribosomal protein L35	-	-
415	178	['1', '2', '3', '4', '5', '6',	ROW	1	2	83	0,127342	0,643664	1,13304	Alphaproteobacteria	0,127342	J	rpsA	Rickettsia	30S ribosomal protein S1	-	-
610	412	['1', '2', '3', '4', '5', '6',	ROW	1	2	3	0,220984	1,023293	1,83973	Alphaproteobacteria	0,220984	M	ftsQ	Rickettsia	Cell division protein ftsQ	Alphaproteobacteria	Sphingopyxis
251	854	['1', '2', '3', '4', '5', '6',	ROW	1	8	17	0,189097	0,558803	0,61379	Alphaproteobacteria	0,189097	K	greA	Rickettsia	Transcription elongation factor GreA	-	-
643	93	['1', '2', '3', '4', '5', '6',	ROW	1	2	41	0,204208	0,741894	1,8123	Alphaproteobacteria	0,204208	E	-	Rickettsia	Thermostable carboxypeptidase	-	-
401	486	['1', '2', '3', '4', '5', '6',	ROW	1	2	3	0,275698	0,605357	0,7587	Alphaproteobacteria	0,275698	-	mpg	Rickettsia	DNA-3-methyladenine glycosidase	Alphaproteobacteria	Ehrlichia
14	436	['1', '2', '3', '4', '5', '6',	ROW	1	2	4	0,116553	0,688023	0,95058	Alphaproteobacteria	0,116553	P	mnhC	Rickettsia	Multisubunit Na+/H+ antiporter, MnhC subunit	-	-
483	166	['1', '2', '3', '4', '5', '6',	ROW	1	2	4	0,087978	0,272533	0,43792	Alphaproteobacteria	0,087978	C	coxA	Rickettsia	Cytochrome c oxidase polypeptide I	-	-
304	183	['1', '2', '3', '4', '5', '6',	ROW	1	2	3	0,205293	0,957642	1,37824	Alphaproteobacteria	0,205293	L	recO	Rickettsia	DNA repair protein RecO	Alphaproteobacteria	Rhodobacter
589	696	['1', '2', '3', '4', '5', '6',	RW	1	-	19	0,087361	-	0,57145	Alphaproteobacteria	0,087361	J	rplE	Rickettsia	50S ribosomal protein L5	-	-
267	162	['1', '2', '3', '4', '5', '6',	RW	1	-	2	0,16536	-	1,09685	Alphaproteobacteria	0,16536	R	osmY	Rickettsia	Putative periplasmic or secreted lipoprotein	Alphaproteobacteria	Ehrlichia

205	29	['1', '2', '3', '4', '5', '6',	RW	1	-	15	0,2224	-	2,03956	Alphaproteobacteria	0,2224	-	bcr1	Rickettsia	MFS-type bicyclomycin resistance protein	-	-
328	48	['1', '2', '3', '4', '5', '6',	RW	1	-	2	0,262384	-	0,91326	Alphaproteobacteria	0,262384	HCRE	gltD	Rickettsia	NADPH-dependent glutamate synthase beta cl	Alphaproteobacteria	Ehrlichia
6	157	['1', '2', '3', '4', '5', '6',	RW	1	-	4	0,231297	-	0,71116	Alphaproteobacteria	0,231297	F	pyrG	Rickettsia	CTP synthase	-	-
209	196	['1', '2', '3', '4', '5', '6',	RW	1	-	2	0,147271	-	0,7803	Alphaproteobacteria	0,147271	KJL	rhlE	Rickettsia	ATP-dependent RNA helicase RhlE	Alphaproteobacteria	Candidatus Pelagibacter
204	82	['1', '2', '3', '4', '5', '6']	R	1	-	-	0,116071	-	-	Alphaproteobacteria	0,116071	I	paaJ	Rickettsia	Acetyl-CoA acetyltransferase	Gammaproteobacteria	Hahella
102	418	['1', '2', '3', '4', '5', '7']	R	1	-	-	0,265737	-	-	Alphaproteobacteria	0,265737	-	-	Rickettsia	hypothetical protein	-	-
102	904	['1', '2', '3', '4', '5', '7']	ROW	1	2	10	0,181395	1,082241	1,5197	Alphaproteobacteria	0,181395	J	rluA1	Rickettsia	Ribosomal large subunit pseudouridine synthas	-	-
102	902	['1', '2', '3', '4', '5', '7']	ROW	1	2	13	0,19903	0,833291	1,14201	Alphaproteobacteria	0,19903	J	rne	Rickettsia	Ribonuclease E	-	-
102	903	['1', '2', '3', '4', '5', '7']	ROW	1	2	3	0,255227	0,996948	1,06336	Alphaproteobacteria	0,255227	O	coxW	Rickettsia	Cytochrome c oxidase assembly protein	Alphaproteobacteria	Anaplasma
246	75	['1', '2', '3', '4', '5']	R	1	-	-	0,575975	-	-	Alphaproteobacteria	0,575975	R	sec7	Rickettsia	Sec7 domain containing protein	Gammaproteobacteria	Legionella
258	143	['1', '2', '3', '4', '6']	R	1	-	-	0,440218	-	-	Alphaproteobacteria	0,440218	-	-	Rickettsia	hypothetical protein	Mollicutes	Mycoplasma
376	900	['1', '2', '3', '5', '6', '7']	R	1	-	-	0,285923	-	-	Alphaproteobacteria	0,285923	-	-	Rickettsia	hypothetical protein	-	-
215	465	['1', '2', '3', '5', '6', '7']	RO	1	2	-	0,402835	1,293091	-	Alphaproteobacteria	0,402835	-	-	Rickettsia	hypothetical protein	-	-
234	958	['1', '2', '3', '6', '7']	R	1	-	-	0,126874	-	-	Alphaproteobacteria	0,126874	S	-	Rickettsia	hypothetical protein	-	-
33	1116	['1', '2', '3']	R	1	-	-	0,670442	-	-	Alphaproteobacteria	0,670442	-	-	Rickettsia	hypothetical protein	-	-
263	57	['1', '2', '3']	R	1	-	-	0,398331	-	-	Alphaproteobacteria	0,398331	-	-	Rickettsia	hypothetical protein	-	-
13	1115	['1', '2', '3']	RW	2	-	1	1,369791	-	1,27362	Alphaproteobacteria	1,273622	R	-	Wolbachia	hypothetical protein	-	-
577	732	['1', '2', '4', '5', '6', '7']	R	1	-	-	0,685393	-	-	Alphaproteobacteria	0,685393	-	-	Rickettsia	hypothetical protein	-	-
280	905	['1', '2', '4', '5', '6', '7']	R	1	-	-	0,296153	-	-	Alphaproteobacteria	0,296153	R	-	Rickettsia	ABC transporter ATP-binding protein	Bacilli	Enterococcus
581	960	['1', '2', '4', '5', '6']	R	1	-	-	0,226286	-	-	Alphaproteobacteria	0,226286	S	-	Rickettsia	hypothetical protein	-	-
177	1	['1', '2', '4']	RO	1	2	-	0,657351	1,002835	-	Alphaproteobacteria	0,657351	KT	spoT21	Rickettsia	Guanosine polyphosphate pyrophosphohydrola	Clostridia	Clostridium
626	1287	['1', '2']	None	-	-	-	-	-	-	Gammaproteobacteri	2,118001	M	wcaC	Shigella	putative glycosyl transferase	-	-
626	1289	['1', '2']	None	-	-	-	-	-	-	Bacilli	1,793594	S	-	Bacillus	hypothetical protein	-	-
626	1290	['1', '2']	None	-	-	-	-	-	-	Alphaproteobacteria	1,303694	M	-	Rhodopseudc	glycosyl transferase, group 1	-	-
626	1286	['1', '2']	R	1	-	-	0,457884	-	-	Alphaproteobacteria	0,457884	-	-	Rickettsia	hypothetical protein	Deltaproteobacteria	Geobacter
453	270	['1', '3', '4', '5', '6', '7']	R	1	-	-	0,161073	-	-	Alphaproteobacteria	0,161073	-	-	Rickettsia	hypothetical protein	Betaproteobacteria	Nitrosomonas
199	949	['1', '3', '4', '5', '6', '7']	RO	1	26	-	0,150769	1,056609	-	Alphaproteobacteria	0,150769	K	iscR	Rickettsia	Iron-sulfur cluster assembly transcription factc	-	-
244	158	['1', '3', '4', '5', '6', '7']	ROW	1	6	3	0,43785	1,87039	1,59137	Alphaproteobacteria	0,43785	-	-	Rickettsia	hypothetical protein	-	-
518	911	['1', '3', '4', '5', '6', '7']	ROW	1	2	17	0,383039	1,727531	2,00923	Alphaproteobacteria	0,383039	R	-	Rickettsia	Sco2 protein precursor	-	-
108	454	['1', '3', '4', '5', '6', '7']	ROW	1	2	5	0,106747	0,554845	0,97323	Alphaproteobacteria	0,106747	O	cox11	Rickettsia	Cytochrome c oxidase assembly protein cox11	-	-
46	153	['1', '3', '4', '5', '6', '7']	ROW	1	2	4	0,282079	0,830188	0,87505	Alphaproteobacteria	0,282079	O	cyoB	Rickettsia	Protoheme IX farnesyltransferase	-	-
510	138	['1', '3', '5', '6', '7']	R	1	-	-	0,07657	-	-	Alphaproteobacteria	0,07657	S	-	Rickettsia	hypothetical protein	-	-
404	247	['1', '4', '5', '6', '7']	R	1	-	-	0,201426	-	-	Alphaproteobacteria	0,201426	-	secG	Rickettsia	Protein-export membrane protein secG	-	-
422	5	['1']															
422	1772	['1']	None	-	-	-	-	-	-	Betaproteobacteria	0,581282	L	-	Cupriavidus	Resolvase-like protein	-	-
422	101	['1']	None	-	-	-	-	-	-	Gammaproteobacteri	1,342796	R	-	Legionella	hypothetical protein	-	-
426	209	['1']	R	1	-	-	0,569215	-	-	Alphaproteobacteria	0,569215	S	-	Rickettsia	hypothetical protein	-	-
422	1338	['1']	R	1	-	-	1,055681	-	-	Alphaproteobacteria	1,055681	R	-	Rickettsia	Ankyrin repeat	-	-
422	90	['1']	RO	1	2	-	0,727038	1,602045	-	Alphaproteobacteria	0,727038	-	-	Rickettsia	Ankyrin repeat	-	-
51	161	['1']	RO	1	17	-	0,213848	0,763811	-	Alphaproteobacteria	0,213848	V	msbA1	Rickettsia	Multidrug resistance protein	-	-
422	89	['1']	RO	2	1	-	1,231006	1,198695	-	Alphaproteobacteria	1,198695	-	-	Orientia tsut	-	-	-
422	17	['1']	RO	1	2	-	0,739867	1,533799	-	Alphaproteobacteria	0,739867	-	-	Rickettsia	Transposase and inactivated derivative	-	-
422	33	['1']	W	-	-	1	-	-	1,30394	Alphaproteobacteria	1,303935	R	-	Wolbachia	ankyrin repeat domain protein	-	-
162	108	['2', '3', '4', '5', '6', '7']	RO	1	12	-	0,252026	1,385878	-	Alphaproteobacteria	0,252026	KT	spoT11	Rickettsia	Guanosine polyphosphate pyrophosphohydrola	-	-
426	908	['2', '3', '5', '6', '7']	R	1	-	-	0,335731	-	-	Alphaproteobacteria	0,335731	S	-	Rickettsia	hypothetical protein	-	-
397	227	['3', '4', '5', '6', '7']	None	-	-	-	-	-	-	Bacilli	0,728009	R	-	Bacillus	aminoglycoside phosphotransferase	-	-
440	1025	['3', '4', '5', '6', '7']	OW	-	37	64	-	1,50895	1,91341	Gammaproteobacteri	0,805021	V	ampD	Coxiella	N-acetylmuramoyl-L-alanine amidase	-	-
65	967	['3', '4', '5', '6', '7']	R	1	-	-	0,328968	-	-	Alphaproteobacteria	0,328968	QR	-	Rickettsia	Tellurite resistance protein-related protein	Gammaproteobacteria	Vibrio
65	966	['3', '4', '5', '6', '7']	R	61	-	-	1,955077	-	-	Gammaproteobacteri	1,427098	-	-	Legionella	polypeptide deformylase	-	-
401	87	['3', '4', '5', '6', '7']	R	1	-	-	0,280902	-	-	Alphaproteobacteria	0,280902	-	-	Rickettsia	hypothetical protein	-	-
56	976	['3', '4', '5', '6', '7']	R	1	-	-	0,675718	-	-	Alphaproteobacteria	0,675718	-	-	Rickettsia	hypothetical protein	-	-
130	884	['3', '4', '5', '6', '7']	R	1	-	-	0,751219	-	-	Alphaproteobacteria	0,751219	-	rickA	Rickettsia	Actin polymerization protein RickA	Mollicutes	Mycoplasma
130	885	['3', '4', '5', '6', '7']	R	1	-	-	0,135224	-	-	Alphaproteobacteria	0,135224	I	scoB	Rickettsia	Succinyl-CoA:3-ketoacid-coenzyme A transfer	Alphaproteobacteria	Rhodopseudomonas
546	23	['3', '4', '5', '6', '7']	R	2	-	-	0,408341	-	-	Gammaproteobacteri	0,402981	LF	-	Legionella	hypothetical protein	-	-
46	945	['3', '4', '5', '6', '7']	R	1	-	-	0,200974	-	-	Alphaproteobacteria	0,200974	-	-	Rickettsia	hypothetical protein	Deinococcus-Thermus	Deinococcus
626	942	['3', '4', '5', '6', '7']	R	1	-	-	0,63976	-	-	Alphaproteobacteria	0,63976	M	-	Rickettsia	hypothetical protein	-	-
452	8	['3', '4', '5', '6', '7']	R	1	-	-	0,280682	-	-	Alphaproteobacteria	0,280682	S	-	Rickettsia	Nucleotidyltransferase	Bacteroidetes/Chlorobi g	Bacteroides
249	24	['3', '4', '5', '6', '7']	R	41	-	-	3,922317	-	-	Alphaproteobacteria	2,26504	-	-	Mesorhizobiu	hypothetical glycine-rich protein	-	-
472	975	['3', '4', '5', '6', '7']	R	1	-	-	0,20116	-	-	Alphaproteobacteria	0,20116	D	-	Rickettsia	Antitoxin of toxin-antitoxin (TA) system Phd	Cyanobacteria	Gloeobacter
590	117	['3', '4', '5', '6', '7']	R	1	-	-	0,314754	-	-	Alphaproteobacteria	0,314754	-	-	Rickettsia	Acetyltransferase	Gammaproteobacteria	Legionella
520	21	['3', '4', '5', '6', '7']	R	1	-	-	0,767065	-	-	Alphaproteobacteria	0,767065	R	-	Rickettsia	Putative AAA+ superfamily ATPase	Bacteroidetes/Chlorobi g	Pelodictyon
477	935	['3', '4', '5', '6', '7']	R	1	-	-	0,243418	-	-	Alphaproteobacteria	0,243418	R	-	Rickettsia	Putative metal-dependent hydrolase	Clostridia	Clostridium
113	39	['3', '4', '5', '6', '7']	R	1	-	-	0,563589	-	-	Alphaproteobacteria	0,563589	-	-	Rickettsia	hypothetical protein	Alphaproteobacteria	Neorickettsia
546	1010	['3', '4', '5', '6', '7']	R	1	-	-	0,391411	-	-	Alphaproteobacteria	0,391411	L	-	Rickettsia	Putative DNA alkylation repair enzyme	Gammaproteobacteria	Legionella
193	897	['3', '4', '5', '6', '7']	R	1	-	-	0,260003	-	-	Alphaproteobacteria	0,260003	-	-	Rickettsia	Cephalosporin hydroxylase	-	-
130	118	['3', '4', '5', '6', '7']	R	1	-	-	0,166307	-	-	Alphaproteobacteria	0,166307	R	-	Rickettsia	Tryptophan repressor binding protein	Betaproteobacteria	Burkholderia
13	45	['3', '4', '5', '6', '7']	R	1	-	-	0,360408	-	-	Alphaproteobacteria	0,360408	-	-	Rickettsia	hypothetical protein	-	-
653	0	['3', '4', '5', '6', '7']	R	1	-	-	0,603261	-	-	Alphaproteobacteria	0,603261	-	proP9_2	Rickettsia	Proline/betaine transporter	-	-
518	910	['3', '4', '5', '6', '7']	R	1	-	-	1,000611	-	-	Alphaproteobacteria	1,000611	-	-	Rickettsia	hypothetical protein	-	-
149	88	['3', '4', '5', '6', '7']	R	1	-	-	0,233526	-	-	Alphaproteobacteria	0,233526	R	lysE	Rickettsia	Lysine efflux permease	Bacilli	Staphylococcus
422	919	['3', '4', '5', '6', '7']	R	1	-	-	0,475601	-	-	Alphaproteobacteria	0,475601	-	-	Rickettsia	hypothetical protein	-	-
366	114	['3', '4', '5', '6', '7']	R	1	-	-	0,209666	-	-	Alphaproteobacteria	0,209666	R	-	Rickettsia	Putative AAA+ superfamily ATPase	Gammaproteobacteria	Coxiella

212	892	['3', '4', '5', '6', '7']	R	1	-	-	0,161645	-	-	Alphaproteobacteria	0,161645	C	gabD	Rickettsia	Succinate semialdehyde dehydrogenase	Gammaproteobacteria	Vibrio
314	1022	['3', '4', '5', '6', '7']	R	1	-	-	0,175252	-	-	Alphaproteobacteria	0,175252	R	-	Rickettsia	Phosphatidylethanolamine-binding protein PEB	Gammaproteobacteria	Legionella
127	1024	['3', '4', '5', '6', '7']	R	1	-	-	0,108703	-	-	Alphaproteobacteria	0,108703	V	-	Rickettsia	Type I restriction-modification system methylt	-	-
127	125	['3', '4', '5', '6', '7']	R	1	-	-	0,386479	-	-	Alphaproteobacteria	0,386479	-	-	Rickettsia	Na+/H+ antiporter NhaA	Gammaproteobacteria	Escherichia
564	40	['3', '4', '5', '6', '7']	R	1	-	-	0,268119	-	-	Alphaproteobacteria	0,268119	E	-	Rickettsia	Amino acid permeases	Gammaproteobacteria	Photobacterium
130	226	['3', '4', '5', '6', '7']	R	1	-	-	0,068172	-	-	Alphaproteobacteria	0,068172	I	-	Rickettsia	Succinyl-CoA:3-ketoacid-coenzyme A transfera	Alphaproteobacteria	Caulobacter
4	987	['3', '4', '5', '6', '7']	R	1	-	-	0,501123	-	-	Alphaproteobacteria	0,501123	-	-	Rickettsia	hypothetical protein	-	-
302	21	['3', '4', '5', '6', '7']	R	1	-	-	0,171498	-	-	Alphaproteobacteria	0,171498	R	-	Rickettsia	Putative AAA+ superfamily ATPase	Bacteroidetes/Chlorobi g	Pelodictyon
186	901	['3', '4', '5', '6', '7']	R	1	-	-	0,564076	-	-	Alphaproteobacteria	0,564076	-	-	Rickettsia	hypothetical protein	-	-
12	12	['3', '4', '5', '6', '7']	R	1	-	-	0,436888	-	-	Alphaproteobacteria	0,436888	-	-	Rickettsia	hypothetical protein	-	-
157	993	['3', '4', '5', '6', '7']	R	1	-	-	0,112561	-	-	Alphaproteobacteria	0,112561	E	-	Rickettsia	Acylamino-acid-releasing enzyme	Gammaproteobacteria	Xanthomonas
106	918	['3', '4', '5', '6', '7']	R	1	-	-	0,224232	-	-	Alphaproteobacteria	0,224232	R	comJ	Rickettsia	ComJ	Alphaproteobacteria	Gluconobacter
416	1016	['3', '4', '5', '6', '7']	R	1	-	-	1,31725	-	-	Alphaproteobacteria	1,31725	-	-	Rickettsia	hypothetical protein	-	-
385	164	['3', '4', '5', '6', '7']	R	1	-	-	1,43967	-	-	Alphaproteobacteria	1,43967	-	-	Rickettsia	hypothetical protein	-	-
224	53	['3', '4', '5', '6', '7']	R	1	-	-	0,23192	-	-	Alphaproteobacteria	0,23192	M	-	Rickettsia	Membrane-fusion protein component of the RN	Deltaproteobacteria	Geobacter
177	983	['3', '4', '5', '6', '7']	R	1	-	-	0,312116	-	-	Alphaproteobacteria	0,312116	-	-	Rickettsia	hypothetical protein	-	-
287	974	['3', '4', '5', '6', '7']	R	1	-	-	0,127515	-	-	Alphaproteobacteria	0,127515	S	-	Rickettsia	hypothetical protein	-	-
544	936	['3', '4', '5', '6', '7']	R	1	-	-	0,098334	-	-	Alphaproteobacteria	0,098334	R	-	Rickettsia	ABC transporter ATP-binding protein	Chlamydiae/Verrucomicr	Candidatus Protochlamydia
340	881	['3', '4', '5', '6', '7']	R	1	-	-	0,145539	-	-	Alphaproteobacteria	0,145539	S	-	Rickettsia	HicB-like protein	Gammaproteobacteria	Nitrosococcus
302	85	['3', '4', '5', '6', '7']	R	1	-	-	0,334404	-	-	Alphaproteobacteria	0,334404	-	-	Rickettsia	hypothetical protein	-	-
134	920	['3', '4', '5', '6', '7']	R	1	-	-	0,122947	-	-	Alphaproteobacteria	0,122947	S	-	Rickettsia	Putative integral membrane protein	Gammaproteobacteria	Saccharophagus
224	121	['3', '4', '5', '6', '7']	R	1	-	-	0,073081	-	-	Alphaproteobacteria	0,073081	V	-	Rickettsia	RND family efflux transporter	Deltaproteobacteria	Geobacter
224	120	['3', '4', '5', '6', '7']	R	1	-	-	0,096288	-	-	Alphaproteobacteria	0,096288	V	-	Rickettsia	RND family efflux transporter	Deltaproteobacteria	Desulfovibrio
244	989	['3', '4', '5', '6', '7']	R	1	-	-	0,300544	-	-	Alphaproteobacteria	0,300544	F	mutT	Rickettsia	ADP-ribose pyrophosphatase MutT	Gammaproteobacteria	Idiomarina
224	220	['3', '4', '5', '6', '7']	R	1	-	-	0,363357	-	-	Alphaproteobacteria	0,363357	UM	-	Rickettsia	RND efflux system, outer membrane protein	Betaproteobacteria	Chromobacterium
224	11	['3', '4', '5', '6', '7']	R	1	-	-	0,446057	-	-	Alphaproteobacteria	0,446057	V	-	Rickettsia	RND family efflux transporter	Deltaproteobacteria	Geobacter
29	927	['3', '4', '5', '6', '7']	R	1	-	-	0,355093	-	-	Alphaproteobacteria	0,355093	-	ecoT	Rickettsia	Ecotin precursor	Gammaproteobacteria	Pseudomonas
641	66	['3', '4', '5', '6', '7']	R	1	-	-	0,153418	-	-	Alphaproteobacteria	0,153418	-	blaD	Rickettsia	Class D beta-lactamase	Gammaproteobacteria	Legionella
520	18	['3', '4', '5', '6', '7']	RO	1	2	-	0,148722	0,676845	-	Alphaproteobacteria	0,148722	R	uup	Rickettsia	ABC transporter ATP-binding protein Uup	Alphaproteobacteria	Magnetospirillum
130	86	['3', '4', '5', '6', '7']	RO	1	2	-	0,238535	1,763521	-	Alphaproteobacteria	0,238535	-	-	Rickettsia	hypothetical protein	-	-
137	123	['3', '4', '5', '6', '7']	RO	1	2	-	0,104173	0,702936	-	Alphaproteobacteria	0,104173	-	-	Rickettsia	hypothetical protein	Betaproteobacteria	Ralstonia
31	58	['3', '4', '5', '6', '7']	RO	1	2	-	0,299879	1,580005	-	Alphaproteobacteria	0,299879	ET	-	Rickettsia	hypothetical protein	Gammaproteobacteria	Hahella
653	1	['3', '4', '5', '6', '7']	RO	1	2	-	0,400015	1,078511	-	Alphaproteobacteria	0,400015	KT	spoT21	Rickettsia	Guanosine polyphosphate pyrophosphohydrola	Gammaproteobacteria	Vibrio
51	990	['3', '4', '5', '6', '7']	RO	1	56	-	0,172019	1,621445	-	Alphaproteobacteria	0,172019	-	ampG4	Rickettsia	AmpG	-	-
308	63	['3', '4', '5', '6', '7']	ROW	1	2	5	0,187389	0,550793	0,94564	Alphaproteobacteria	0,187389	S	-	Rickettsia	Phage portal protein	-	-
431	1007	['3', '4', '5', '6', '7']	ROW	15	3	8	2,856171	2,091997	2,46955	Cyanobacteria	2,067174	S	-	Synechococc	pentapeptide repeat family protein	-	-
614	119	['3', '4', '5', '6', '7']	ROW	1	2	4	0,142706	0,714132	0,9942	Alphaproteobacteria	0,142706	SR	-	Rickettsia	Putative phage terminase protein	-	-
248	954	['3', '4', '5', '6', '7']	ROW	1	2	24	0,157852	0,695416	1,18091	Alphaproteobacteria	0,157852	L	radC	Rickettsia	DNA repair protein RadC	-	-
308	888	['3', '4', '5', '6', '7']	ROW	1	2	3	0,283203	0,874975	1,08588	Alphaproteobacteria	0,283203	-	-	Rickettsia	hypothetical protein	Alphaproteobacteria	Ehrlichia
618	222	['3', '4', '5', '6', '7']	ROW	1	2	3	0,305018	1,242261	1,56393	Alphaproteobacteria	0,305018	E	argB	Rickettsia	Acetylglutamate kinase	Thermotogae	Thermotoga
552	1000	['3', '4', '5', '6', '7']	ROW	1	2	3	0,196195	1,065366	1,33997	Alphaproteobacteria	0,196195	-	-	Rickettsia	hypothetical protein	Alphaproteobacteria	Ehrlichia
129	52	['3', '4', '5', '6', '7']	ROW	1	2	3	0,24123	1,134895	1,4615	Alphaproteobacteria	0,24123	M	-	Rickettsia	Glycosyltransferase	Actinobacteria	Corynebacterium
18	72	['3', '4', '5', '6', '7']	ROW	1	2	9	0,182285	0,748823	1,07463	Alphaproteobacteria	0,182285	UL	-	Rickettsia	Putative DNA processing protein DprA	-	-
422	972	['3', '4', '5', '6', '7']	ROW	1	14	2	0,141435	0,907112	0,75125	Alphaproteobacteria	0,141435	O	bcp	Rickettsia	Bacterioferritin comigratory protein	-	-
299	60	['3', '4', '5', '6', '7']	RW	1	-	2	0,292637	-	0,87349	Alphaproteobacteria	0,292637	H	folKP	Rickettsia	Folate synthesis bifunctional protein	Chlamydiae/Verrucomicr	Chlamydophila
599	922	['3', '4', '5', '6', '7']	RW	1	-	3	0,278424	-	1,03972	Alphaproteobacteria	0,278424	L	mutM	Rickettsia	Formamidopyrimidine-DNA glycosidase	-	-
599	882	['3', '4', '5', '6', '7']	RW	1	-	2	0,387278	-	1,22116	Alphaproteobacteria	0,387278	M	-	Rickettsia	Mannose-1-phosphate guanylyltransferase	Gammaproteobacteria	Acinetobacter
106	744	['3', '4', '5', '6', '7']	RW	1	-	5	0,275895	-	1,82219	Alphaproteobacteria	0,275895	M	-	Rickettsia	Periplasmic protein	-	-
134	223	['3', '4', '5', '6', '7']	RW	1	-	28	0,204014	-	2,03751	Alphaproteobacteria	0,204014	MG	-	Rickettsia	Putative oxidoreductase protein	-	-
205	8	['3', '4', '5', '6']	R	1	-	-	0,313991	-	-	Alphaproteobacteria	0,313991	S	-	Rickettsia	Nucleotidyltransferase	Bacteroidetes/Chlorobi g	Bacteroides
105	955	['3', '4', '5', '6']	R	1	-	-	0,515591	-	-	Alphaproteobacteria	0,515591	-	-	Rickettsia	hypothetical protein	-	-
581	956	['3', '4', '5', '6']	R	1	-	-	0,252451	-	-	Alphaproteobacteria	0,252451	IQR	-	Rickettsia	Oxidoreductase	Betaproteobacteria	Polaromonas
346	225	['3', '4', '5', '6']	R	1	-	-	2,797661	-	-	Alphaproteobacteria	2,797661	-	-	Rickettsia	hypothetical protein	-	-
546	1078	['3', '4', '5', '6']	R	1	-	-	0,194561	-	-	Alphaproteobacteria	0,194561	L	-	Rickettsia	Putative DNA alkylation repair enzyme	-	-
523	1015	['3', '4', '5', '6']	R	1	-	-	0,108033	-	-	Alphaproteobacteria	0,108033	R	chaB	Rickettsia	Cation transport regulator ChaB	Gammaproteobacteria	Sodalis
581	1167	['3', '4', '5']	R	1	-	-	0,066687	-	-	Alphaproteobacteria	0,066687	IQR	-	Rickettsia	Oxidoreductase	Alphaproteobacteria	Agrobacterium
204	1129	['3', '4', '5']	R	1	-	-	0,551421	-	-	Alphaproteobacteria	0,551421	L	-	Rickettsia	Superfamily I DNA and RNA helicases	-	-
204	1045	['3', '4', '5']	R	1	-	-	0,398537	-	-	Alphaproteobacteria	0,398537	-	-	Rickettsia	hypothetical protein	-	-
151	1044	['3', '4', '6', '7']	R	1	-	-	0,175158	-	-	Alphaproteobacteria	0,175158	L	-	Rickettsia	Site-specific DNA methylase	Bacilli	Streptococcus
544	18	['3', '4', '6']	R	1	-	-	0,141961	-	-	Alphaproteobacteria	0,141961	R	-	Rickettsia	ABC transporter ATP-binding protein	Gammaproteobacteria	Pseudomonas
127	1166	['3', '4', '7']	R	1	-	-	0,181374	-	-	Alphaproteobacteria	0,181374	V	-	Rickettsia	Type I restriction-modification system methylt	-	-
447	941	['3', '4']	None	-	-	-	-	-	-	Gammaproteobacteri	0,582917	R	-	Legionella	hypothetical protein	-	-
340	973	['3', '4']	None	-	-	-	-	-	-	Gammaproteobacteri	0,925316	-	-	Nitrosococcu	hypothetical protein	-	-
251	1354	['3', '4']	None	-	-	-	-	-	-	Mollicutes	1,04019	-	-	Mycoplasma	hypothetical protein	-	-
604	1093	['3', '4']	R	1	-	-	0,859998	-	-	Alphaproteobacteria	0,859998	-	-	Rickettsia	hypothetical protein	-	-
631	89	['3', '4']	R	1	-	-	0,879405	-	-	Alphaproteobacteria	0,879405	-	-	Rickettsia	hypothetical protein	-	-
235	1348	['3', '4']	R	1	-	-	0,129298	-	-	Alphaproteobacteria	0,129298	K	-	Rickettsia	Phage-related transcriptional regulator	Gammaproteobacteria	Nitrosococcus
465	889	['3', '4']	R	2	-	-	0,966205	-	-	Betaproteobacteria	0,907877	D	-	Azoarcus	putative antitoxin of toxin-antitoxin stability sy	-	-
494	938	['3', '4']	R	1	-	-	0,398424	-	-	Alphaproteobacteria	0,398424	-	-	Rickettsia	hypothetical protein	Gammaproteobacteria	Colwellia
310	961	['3', '4']	R	1	-	-	0,06852	-	-	Alphaproteobacteria	0,06852	-	-	Rickettsia	Antitoxin of toxin-antitoxin (TA) system StbD	-	-
520	1345	['3', '4']	R	1	-	-	0,897034	-	-	Alphaproteobacteria	0,897034	-	-	Rickettsia	hypothetical protein	-	-
478	899	['3', '4']	R	1	-	-	0,228154	-	-	Alphaproteobacteria	0,228154	S	-	Rickettsia	Putative nucleic-acid-binding protein	Cyanobacteria	Anabaena

609	1351	['3', '4']	R	1	-	-	0,131332	-	-	Alphaproteobacteria	0,131332	-	-	Rickettsia	hypothetical protein	-	-
308	2	['3', '4']	R	21	-	-	3,429921	-	-	Cyanobacteria	1,792236	-	-	Nostoc	hypothetical protein	-	-
212	1149	['3', '4']	R	1	-	-	0,606697	-	-	Alphaproteobacteria	0,606697	-	-	Rickettsia	hypothetical protein	-	-
552	1329	['3', '4']	R	1	-	-	0,298278	-	-	Alphaproteobacteria	0,298278	-	-	Rickettsia	hypothetical protein	-	-
552	1330	['3', '4']	R	1	-	-	0,185473	-	-	Alphaproteobacteria	0,185473	-	-	Rickettsia	hypothetical protein	-	-
204	112	['3', '4']	R	1	-	-	0,157719	-	-	Alphaproteobacteria	0,157719	S	-	Rickettsia	hypothetical protein	Bacteroidetes/Chlorobi g	Chlorobium
193	1342	['3', '4']	R	1	-	-	0,187809	-	-	Alphaproteobacteria	0,187809	V	-	Rickettsia	Cephalosporin hydroxylase	Alphaproteobacteria	Mesorhizobium
483	136	['3', '4']	R	1	-	-	0,366652	-	-	Alphaproteobacteria	0,366652	-	-	Rickettsia	hypothetical protein	-	-
478	1343	['3', '4']	R	1	-	-	0,335221	-	-	Alphaproteobacteria	0,335221	R	-	Rickettsia	Putative Zn-dependent hydrolase	Alphaproteobacteria	Rhizobium
212	970	['3', '4']	R	1	-	-	0,65342	-	-	Alphaproteobacteria	0,65342	-	-	Rickettsia	hypothetical protein	-	-
599	50	['3', '4']	R	1	-	-	0,378129	-	-	Alphaproteobacteria	0,378129	S	-	Rickettsia	hypothetical protein	Betaproteobacteria	Ralstonia
79	0	['3', '4']	RO	1	15	-	0,649951	1,991991	-	Alphaproteobacteria	0,649951	-	proP10	Rickettsia	Proline/betaine transporter	-	-
404	1041	['3', '4']	RO	1	3	-	0,612374	0,710461	-	Alphaproteobacteria	0,612374	S	-	Rickettsia	Putative integral membrane protein	-	-
5	17	['3', '4']	RO	1	2	-	0,297994	1,253252	-	Alphaproteobacteria	0,297994	-	-	Rickettsia	Transposase and inactivated derivative	-	-
177	108	['3', '4']	RO	1	18	-	0,589013	1,453899	-	Alphaproteobacteria	0,589013	KT	spoT12	Rickettsia	Guanosine polyphosphate pyrophosphohydrola	-	-
50	1143	['3', '4']	RW	18	-	1	2,2766	-	0,4174	Alphaproteobacteria	0,417396	S	-	Wolbachia	Fic family protein	-	-
299	1122	['3', '4']	W	-	-	1	-	-	0,54866	Alphaproteobacteria	0,548664	H	-	Wolbachia	coenzyme PQQ synthesis protein C, putative	Alphaproteobacteria	Neorickettsia
46	944	['3', '5', '6', '7']	None	-	-	-	-	-	-	Actinobacteria	1,160917	L	-	Streptomyce	lyase	-	-
256	979	['3', '5', '6', '7']	None	-	-	-	-	-	-	Gammaproteobacteri	0,46117	L	-	Pseudomona	DNA-damage-inducible protein	-	-
517	228	['3', '5', '6', '7']	R	1	-	-	0,340014	-	-	Alphaproteobacteria	0,340014	UN	fimD	Rickettsia	P pilus assembly, fimbrial Usher protein	Betaproteobacteria	Ralstonia
95	1083	['3', '5', '6', '7']	R	1	-	-	0,217994	-	-	Alphaproteobacteria	0,217994	-	-	Rickettsia	hypothetical protein	-	-
87	1074	['3', '5', '6', '7']	R	1	-	-	0,141915	-	-	Alphaproteobacteria	0,141915	K	-	Rickettsia	Putative transcriptional regulator	Gammaproteobacteria	Legionella
65	916	['3', '5', '6', '7']	R	1	-	-	0,343602	-	-	Alphaproteobacteria	0,343602	G	-	Rickettsia	hypothetical protein	-	-
517	64	['3', '5', '6', '7']	R	1	-	-	0,283325	-	-	Alphaproteobacteria	0,283325	UN	fimD	Rickettsia	P pilus assembly, fimbrial Usher protein	Betaproteobacteria	Ralstonia
129	982	['3', '5', '6', '7']	R	1	-	-	0,23771	-	-	Alphaproteobacteria	0,23771	-	-	Rickettsia	hypothetical protein	-	-
517	997	['3', '5', '6', '7']	R	1	-	-	0,39305	-	-	Alphaproteobacteria	0,39305	S	-	Rickettsia	hypothetical protein	Betaproteobacteria	Burkholderia
197	41	['3', '5', '6', '7']	R	1	-	-	0,147248	-	-	Alphaproteobacteria	0,147248	-	-	Rickettsia	Prophage antirepressor	Betaproteobacteria	Rhodoferax
631	10	['3', '5', '6', '7']	R	1	-	-	1,655466	-	-	Alphaproteobacteria	1,655466	-	sca2	Rickettsia	Cell surface antigen Sca2	-	-
157	115	['3', '5', '6', '7']	R	1	-	-	0,252257	-	-	Alphaproteobacteria	0,252257	C	-	Rickettsia	Cytochrome b561 family protein	-	-
127	1023	['3', '5', '6', '7']	R	1	-	-	0,211629	-	-	Alphaproteobacteria	0,211629	V	-	Rickettsia	Type I restriction-modification system methylt	Alphaproteobacteria	Gluconobacter
13	1059	['3', '5', '6', '7']	R	1	-	-	0,233683	-	-	Alphaproteobacteria	0,233683	-	-	Rickettsia	hypothetical protein	Cyanobacteria	Gloeobacter
13	988	['3', '5', '6', '7']	R	1	-	-	0,369973	-	-	Alphaproteobacteria	0,369973	R	-	Rickettsia	hypothetical protein	Cyanobacteria	Gloeobacter
60	1057	['3', '5', '6', '7']	R	1	-	-	0,339875	-	-	Alphaproteobacteria	0,339875	-	vapB1	Rickettsia	Antitoxin of toxin-antitoxin (TA) system VapB	-	-
564	1006	['3', '5', '6', '7']	R	1	-	-	0,316375	-	-	Alphaproteobacteria	0,316375	-	-	Rickettsia	hypothetical protein	-	-
60	934	['3', '5', '6', '7']	R	1	-	-	0,253263	-	-	Alphaproteobacteria	0,253263	R	vapC1	Rickettsia	Toxin of toxin-antitoxin (TA) system VapC	-	-
400	1082	['3', '5', '6', '7']	R	1	-	-	0,322744	-	-	Alphaproteobacteria	0,322744	-	-	Rickettsia	hypothetical protein	-	-
235	3	['3', '5', '6', '7']	RO	1	8	-	0,590997	2,549899	-	Alphaproteobacteria	0,590997	S	-	Rickettsia	Cell surface antigen Sca13	-	-
391	921	['3', '5', '6', '7']	RO	1	2	-	0,302947	0,444064	-	Alphaproteobacteria	0,302947	S	-	Rickettsia	Transposase and inactivated derivative	Gammaproteobacteria	Escherichia
400	20	['3', '5', '6', '7']	ROW	1	17	6	0,195927	1,612065	1,4754	Alphaproteobacteria	0,195927	V	mdlB	Rickettsia	ABC-type multidrug transport system, ATPase	-	-
118	939	['3', '5', '6', '7']	ROW	1	2	18	0,126498	0,552444	0,83955	Alphaproteobacteria	0,126498	O	-	Rickettsia	Mg chelatase-related protein	-	-
299	218	['3', '5', '6', '7']	RW	1	-	2	0,307197	-	1,05942	Alphaproteobacteria	0,307197	H	folA	Rickettsia	Dihydrofolate reductase	Chlamydiae/Verrucomicr	Chlamydophila
95	7	['3', '5', '6', '7']	RW	5	-	8	0,908683	-	0,94484	Cyanobacteria	0,788975	S	-	Anabaena	Protein of unknown function DUF1016	-	-
136	984	['3', '5', '6']	None	-	-	-	-	-	-	Alphaproteobacteria	1,321404	K	-	Magnetospiri	Regulator of competence-specific gene	-	-
514	1135	['3', '5', '6']	R	1	-	-	0,226125	-	-	Alphaproteobacteria	0,226125	G	-	Rickettsia	Capsular polysaccharide biosynthesis protein	Cyanobacteria	Synechococcus
346	1054	['3', '5', '6']	R	1	-	-	0,981465	-	-	Alphaproteobacteria	0,981465	-	-	Rickettsia	hypothetical protein	-	-
87	229	['3', '5', '6']	R	1	-	-	1,183305	-	-	Alphaproteobacteria	1,183305	C	-	Rickettsia	hypothetical protein	-	-
546	1080	['3', '5', '6']	R	1	-	-	0,4037	-	-	Alphaproteobacteria	0,4037	V	-	Rickettsia	Type I site-specific restriction-modification sys	Bacilli	Bacillus
544	890	['3', '5', '7']	R	1	-	-	1,686048	-	-	Alphaproteobacteria	1,686048	-	-	Rickettsia	hypothetical protein	-	-
478	1089	['3', '5', '7']	R	1	-	-	0,211501	-	-	Alphaproteobacteria	0,211501	R	-	Rickettsia	Putative Zn-dependent hydrolase	Gammaproteobacteria	Colwellia
204	22	['3', '5', '7']	R	1	-	-	0,547969	-	-	Alphaproteobacteria	0,547969	L	-	Rickettsia	Superfamily I DNA and RNA helicases	Gammaproteobacteria	Photobacterium
113	969	['3', '5']	R	1	-	-	1,470791	-	-	Alphaproteobacteria	1,470791	-	-	Rickettsia	hypothetical protein	-	-
110	880	['3', '5']	R	1	-	-	0,983855	-	-	Alphaproteobacteria	0,983855	-	-	Rickettsia	hypothetical protein	-	-
517	1151	['3', '5']	R	1	-	-	0,901567	-	-	Alphaproteobacteria	0,901567	UN	famC	Rickettsia	P pilus assembly protein, chaperone PapD	Alphaproteobacteria	Agrobacterium
544	937	['3', '5']	R	1	-	-	1,048241	-	-	Alphaproteobacteria	1,048241	-	-	Rickettsia	hypothetical protein	-	-
581	1026	['3', '5']	R	1	-	-	0,545425	-	-	Alphaproteobacteria	0,545425	-	-	Rickettsia	hypothetical protein	-	-
605	1061	['3', '5']	RO	1	2	-	0,400939	1,212152	-	Alphaproteobacteria	0,400939	-	-	Rickettsia	hypothetical protein	Alphaproteobacteria	Magnetospirillum
79	836	['3', '6', '7']	R	1	-	-	0,85703	-	-	Alphaproteobacteria	0,85703	-	-	Rickettsia	MFS-type transporter	-	-
483	1323	['3', '6']	R	1	-	-	0,248373	-	-	Alphaproteobacteria	0,248373	IM	tagD	Rickettsia	Glycerol-3-phosphate cytidyltransferase TagD	Euryarchaeota	Pyrococcus
637	49	['3', '6']	R	1	-	-	1,231236	-	-	Alphaproteobacteria	1,231236	-	-	Rickettsia	hypothetical protein	-	-
93	1127	['3', '6']	R	1	-	-	0,591081	-	-	Alphaproteobacteria	0,591081	S	-	Rickettsia	hypothetical protein	Gammaproteobacteria	Salmonella
581	116	['3', '6']	RO	6	5	-	1,460959	1,444765	-	Euryarchaeota	1,169046	R	-	Methanosarc	TPR repeat	-	-
517	893	['3', '7']	None	-	-	-	-	-	-	Gammaproteobacteri	1,399921	S	-	Pseudomona	chitin-binding protein	-	-
481	1165	['3', '7']	None	-	-	-	-	-	-	Betaproteobacteria	0,738295	D	-	Nitrosomona	hypothetical protein	-	-
552	1069	['3', '7']	R	1	-	-	0,425439	-	-	Alphaproteobacteria	0,425439	-	-	Rickettsia	hypothetical protein	-	-
544	1145	['3', '7']	R	1	-	-	1,234922	-	-	Alphaproteobacteria	1,234922	-	-	Rickettsia	hypothetical protein	-	-
164	740	['3']															
152	1301	['3']															
324	1011	['3']															
152	1302	['3']	None	-	-	-	-	-	-	Spirochaetes	0,934434	R	-	Treponema	vapC protein, putative	-	-
478	16	['3']	None	-	-	-	-	-	-	Gammaproteobacteri	0,608197	L	-	Psychrobact	transposase, mutator type	-	-
519	7	['3']	None	-	-	-	-	-	-	Betaproteobacteria	1,310369	S	-	Burkholderia	protein of unknown function DUF1016	-	-
368	154	['3']	None	-	-	-	-	-	-	Cyanobacteria	0,672341	M	-	Gloeobacter	hypothetical protein	-	-

481	67	['3']	None	-	-	-	-	-	-	Gammaproteobacteri:	1,685646	V	-	Legionella	hypothetical protein	-	-
386	16	['3']	None	-	-	-	-	-	-	Gammaproteobacteri:	0,626258	L	-	Psychrobacte	transposase, mutator type	-	-
472	928	['3']	None	-	-	-	-	-	-	Alphaproteobacteria	1,414841	R	-	Rhodopseudo	PilT protein	-	-
546	16	['3']	None	-	-	-	-	-	-	Gammaproteobacteri:	0,620344	L	-	Psychrobacte	transposase, mutator type	-	-
653	1163	['3']	None	-	-	-	-	-	-	Deinococcus-Thermus	0,980495	JD	-	Thermus	toxin-like protein	-	-
581	16	['3']	None	-	-	-	-	-	-	Gammaproteobacteri:	0,598458	L	-	Psychrobacte	transposase, mutator type	-	-
401	1320	['3']	None	-	-	-	-	-	-	Bacilli	0,907341	-	-	Streptococcu	hypothetical protein	-	-
13	201	['3']	None	-	-	-	-	-	-	Deltaproteobacteria	0,436078	H	bioB	Lawsonia	biotin synthase	-	-
263	926	['3']	None	-	-	-	-	-	-	Fibrobacteres/Acidob	0,620495	R	-	unclassified /	metallophosphoesterase	-	-
126	16	['3']	None	-	-	-	-	-	-	Gammaproteobacteri:	0,608197	L	-	Psychrobacte	transposase, mutator type	-	-
204	970	['3']	None	-	-	-	-	-	-	Alphaproteobacteria	1,495783	G	-	Nitrobacter	PfkB	-	-
177	1555	['3']	None	-	-	-	-	-	-	Betaproteobacteria	0,873945	-	-	Burkholderia	Putative exochitinase, ChiA-like	-	-
250	1314	['3']	None	-	-	-	-	-	-	Deltaproteobacteria	0,677497	S	-	Syntrophus	DNA damage inducible protein	-	-
250	979	['3']	None	-	-	-	-	-	-	Deltaproteobacteria	1,095651	L	-	Syntrophus	DNA -damage-inducible protein J	-	-
631	16	['3']	None	-	-	-	-	-	-	Gammaproteobacteri:	0,626258	L	-	Psychrobacte	transposase, mutator type	-	-
134	1046	['3']	None	-	-	-	-	-	-	Betaproteobacteria	1,974375	-	-	Polaromonas	hypothetical protein	-	-
280	33	['3']	OW	-	7	1	-	2,263848	1,99854	Alphaproteobacteria	1,998541	R	-	Wolbachia	ankyrin repeat domain protein	-	-
339	1338	['3']	R	1	-	-	0,497062	-	-	Alphaproteobacteria	0,497062	R	-	Rickettsia	Ankyrin repeat	-	-
546	1596	['3']	R	1	-	-	0,391634	-	-	Alphaproteobacteria	0,391634	V	-	Rickettsia	Type I site-specific restriction-modification sys	Epsilonproteobacteria	Thiomicrospira
454	1610	['3']	R	1	-	-	0,203755	-	-	Alphaproteobacteria	0,203755	K	-	Rickettsia	hypothetical protein	-	-
457	985	['3']	R	1	-	-	0,621722	-	-	Alphaproteobacteria	0,621722	-	-	Rickettsia	Ankyrin repeat	-	-
322	994	['3']	R	1	-	-	1,746639	-	-	Alphaproteobacteria	1,746639	-	-	Rickettsia	hypothetical protein	-	-
151	1530	['3']	R	1	-	-	0,172611	-	-	Alphaproteobacteria	0,172611	L	-	Rickettsia	Site-specific DNA methylase	-	-
204	917	['3']	R	1	-	-	1,710966	-	-	Alphaproteobacteria	1,710966	-	-	Rickettsia	hypothetical protein	-	-
235	190	['3']	R	1	-	-	0,445094	-	-	Alphaproteobacteria	0,445094	-	-	Rickettsia	hypothetical protein	-	-
436	116	['3']	R	1	-	-	1,032986	-	-	Alphaproteobacteria	1,032986	-	-	Rickettsia	Tetratricopeptide repeat-containing protein	Euryarchaeota	Methanosarcina
518	1039	['3']	R	1	-	-	1,031118	-	-	Alphaproteobacteria	1,031118	-	-	Rickettsia	hypothetical protein	-	-
404	1040	['3']	R	1	-	-	1,36071	-	-	Alphaproteobacteria	1,36071	-	-	Rickettsia	hypothetical protein	-	-
386	9	['3']	R	1	-	-	1,042063	-	-	Alphaproteobacteria	1,042063	-	-	Rickettsia	Variable membrane protein-like protein	Mollicutes	Mycoplasma
263	113	['3']	R	1	-	-	1,48174	-	-	Alphaproteobacteria	1,48174	-	-	Rickettsia	hypothetical protein	-	-
546	219	['3']	R	1	-	-	0,635346	-	-	Alphaproteobacteria	0,635346	R	-	Rickettsia	Ankyrin repeat	Euryarchaeota	Methanosarcina
465	1594	['3']	R	1	-	-	0,271676	-	-	Alphaproteobacteria	0,271676	S	-	Rickettsia	Fic family protein	-	-
465	1593	['3']	R	1	-	-	0,203175	-	-	Alphaproteobacteria	0,203175	S	-	Rickettsia	Fic family protein	Alphaproteobacteria	Gluconobacter
433	1003	['3']	R	1	-	-	2,528947	-	-	Alphaproteobacteria	2,528947	-	-	Rickettsia	hypothetical protein	-	-
404	5	['3']	R	1	-	-	0,400651	-	-	Alphaproteobacteria	0,400651	L	-	Rickettsia	Transposase and inactivated derivative	Alphaproteobacteria	Rhodospirillum
640	881	['3']	R	1	-	-	0,165881	-	-	Alphaproteobacteria	0,165881	S	-	Rickettsia	HicB-like protein	Spirochaetes	Treponema
199	1071	['3']	R	1	-	-	0,778508	-	-	Alphaproteobacteria	0,778508	-	-	Rickettsia	hypothetical protein	-	-
129	1554	['3']	R	1	-	-	0,478555	-	-	Alphaproteobacteria	0,478555	-	-	Rickettsia	hypothetical protein	-	-
231	971	['3']	R	1	-	-	0,48414	-	-	Alphaproteobacteria	0,48414	-	-	Rickettsia	hypothetical protein	-	-
517	996	['3']	R	1	-	-	0,20492	-	-	Alphaproteobacteria	0,20492	-	-	Rickettsia	Antitoxin of toxin-antitoxin (TA) system Phd	-	-
303	106	['3']	R	1	-	-	1,269756	-	-	Alphaproteobacteria	1,269756	-	-	Rickettsia	hypothetical protein	-	-
478	1147	['3']	R	1	-	-	0,289676	-	-	Alphaproteobacteria	0,289676	-	-	Rickettsia	Transcriptional regulator	-	-
517	1576	['3']	R	1	-	-	0,110753	-	-	Alphaproteobacteria	0,110753	S	-	Rickettsia	Toxin of toxin-antitoxin (TA) system	Cyanobacteria	Nostoc
235	2	['3']	R	1	-	-	0,941089	-	-	Alphaproteobacteria	0,941089	-	-	Rickettsia	Cell surface antigen Sca9	-	-
184	1084	['3']	R	1	-	-	1,316398	-	-	Alphaproteobacteria	1,316398	R	-	Rickettsia	hypothetical protein	-	-
465	1077	['3']	R	1	-	-	0,087241	-	-	Alphaproteobacteria	0,087241	S	-	Rickettsia	Fic family protein	Alphaproteobacteria	Agrobacterium
544	986	['3']	R	1	-	-	1,611114	-	-	Alphaproteobacteria	1,611114	-	-	Rickettsia	hypothetical protein	-	-
224	891	['3']	R	1	-	-	1,105399	-	-	Alphaproteobacteria	1,105399	-	-	Rickettsia	hypothetical protein	-	-
546	1040	['3']	R	1	-	-	0,647439	-	-	Alphaproteobacteria	0,647439	-	-	Rickettsia	hypothetical protein	-	-
79	896	['3']	R	1	-	-	1,140126	-	-	Alphaproteobacteria	1,140126	-	-	Rickettsia	Leucine-rich repeats (LRRs), ribonuclease inhib	Gammaproteobacteria	Legionella
562	136	['3']	R	1	-	-	0,602457	-	-	Alphaproteobacteria	0,602457	-	-	Rickettsia	hypothetical protein	-	-
244	1567	['3']	R	1	-	-	0,32309	-	-	Alphaproteobacteria	0,32309	-	-	Rickettsia	Alkylated DNA repair protein	Alphaproteobacteria	Agrobacterium
137	148	['3']	R	1	-	-	0,140467	-	-	Alphaproteobacteria	0,140467	O	hspC1	Rickettsia	Small heat shock protein	-	-
546	986	['3']	R	1	-	-	1,314016	-	-	Alphaproteobacteria	1,314016	-	-	Rickettsia	hypothetical protein	-	-
125	1322	['3']	R	1	-	-	0,872854	-	-	Alphaproteobacteria	0,872854	-	-	Rickettsia	hypothetical protein	-	-
79	1040	['3']	R	1	-	-	0,821252	-	-	Alphaproteobacteria	0,821252	-	-	Rickettsia	Leucine-rich repeats (LRRs), ribonuclease inhib	-	-
485	21	['3']	R	1	-	-	0,123205	-	-	Alphaproteobacteria	0,123205	R	-	Rickettsia	Putative AAA+ superfamily ATPase	Alphaproteobacteria	Rhizobium
65	1126	['3']	R	1	-	-	0,290078	-	-	Alphaproteobacteria	0,290078	G	-	Rickettsia	hypothetical protein	-	-
552	1067	['3']	R	1	-	-	0,544011	-	-	Alphaproteobacteria	0,544011	-	-	Rickettsia	hypothetical protein	-	-
552	171	['3']	R	1	-	-	0,738109	-	-	Alphaproteobacteria	0,738109	-	-	Rickettsia	hypothetical protein	-	-
447	1147	['3']	R	1	-	-	0,190004	-	-	Alphaproteobacteria	0,190004	-	-	Rickettsia	Transcriptional regulator	-	-
479	35	['3']	R	1	-	-	0,515354	-	-	Alphaproteobacteria	0,515354	-	-	Rickettsia	hypothetical protein	-	-
609	1352	['3']	R	1	-	-	0,081195	-	-	Alphaproteobacteria	0,081195	T	-	Rickettsia	Growth inhibitor	-	-
552	61	['3']	R	1	-	-	0,379802	-	-	Alphaproteobacteria	0,379802	-	-	Rickettsia	Nucleotidyltransferase substrate binding prote	Gammaproteobacteria	Coxiella
552	1312	['3']	R	1	-	-	0,219737	-	-	Alphaproteobacteria	0,219737	-	-	Rickettsia	hypothetical protein	-	-
205	1309	['3']	R	1	-	-	0,768267	-	-	Alphaproteobacteria	0,768267	L	-	Rickettsia	Cassette chromosome recombinase B	Alphaproteobacteria	Nitrobacter
412	182	['3']	R	1	-	-	0,440054	-	-	Alphaproteobacteria	0,440054	S	-	Rickettsia	hypothetical protein	Alphaproteobacteria	Bartonella
310	1038	['3']	R	1	-	-	0,198454	-	-	Alphaproteobacteria	0,198454	JD	-	Rickettsia	Cytotoxic translational repressor of toxin-antit	-	-
604	890	['3']	R	1	-	-	1,533837	-	-	Alphaproteobacteria	1,533837	-	-	Rickettsia	hypothetical protein	-	-
331	70	['3']	R	1	-	-	0,141262	-	-	Alphaproteobacteria	0,141262	-	-	Rickettsia	hypothetical protein	-	-
324	8	['3']	R	1	-	-	0,216164	-	-	Alphaproteobacteria	0,216164	S	-	Rickettsia	Nucleotidyltransferase	Bacteroidetes/Chlorobi g	Bacteroides
581	111	['3']	R	1	-	-	0,141565	-	-	Alphaproteobacteria	0,141565	IQR	-	Rickettsia	Oxidoreductase	Alphaproteobacteria	Sinorhizobium

168	61	['3']	R	1	-	-	0,649625	-	-	Alphaproteobacteria	0,649625	-	-	Rickettsia	Nucleotidyltransferase	Clostridia	Thermoanaerobacter
479	1339	['3']	R	1	-	-	1,300167	-	-	Alphaproteobacteria	1,300167	-	-	Rickettsia	hypothetical protein	-	-
65	1528	['3']	R	1	-	-	0,615172	-	-	Alphaproteobacteria	0,615172	G	-	Rickettsia	hypothetical protein	-	-
418	8	['3']	R	1	-	-	0,275917	-	-	Alphaproteobacteria	0,275917	S	-	Rickettsia	Nucleotidyltransferase	Bacteroidetes/Chlorobi g	Bacteroides
263	1309	['3']	R	1	-	-	0,698115	-	-	Alphaproteobacteria	0,698115	L	-	Rickettsia	Cassette chromosome recombinase B	-	-
249	2	['3']	R	1	-	-	1,241468	-	-	Alphaproteobacteria	1,241468	S	-	Rickettsia	Cell surface antigen Sca13	-	-
308	59	['3']	R	1	-	-	0,359406	-	-	Alphaproteobacteria	0,359406	-	-	Rickettsia	hypothetical protein	-	-
599	1048	['3']	R	1	-	-	0,793864	-	-	Alphaproteobacteria	0,793864	-	-	Rickettsia	hypothetical protein	-	-
586	1536	['3']	R	1	-	-	1,094311	-	-	Alphaproteobacteria	1,094311	-	-	Rickettsia	hypothetical protein	-	-
412	1021	['3']	R	1	-	-	0,153357	-	-	Alphaproteobacteria	0,153357	K	-	Rickettsia	Putative transcriptional regulator	Chlamydiae/Verrucomicr	Candidatus Protochlamydia
477	926	['3']	R	2	-	-	0,876784	-	-	Betaproteobacteria	0,78856	S	-	Nitrosomona	hypothetical protein	-	-
404	219	['3']	R	1	-	-	1,570862	-	-	Alphaproteobacteria	1,570862	R	-	Rickettsia	Ankyrin repeat	-	-
235	1163	['3']	R	1	-	-	0,232611	-	-	Alphaproteobacteria	0,232611	JD	-	Rickettsia	Cytotoxic translational repressor of toxin-antit	-	-
346	1137	['3']	R	1	-	-	0,927831	-	-	Alphaproteobacteria	0,927831	-	-	Rickettsia	hypothetical protein	-	-
263	116	['3']	RO	11	8	-	1,781832	1,583048	-	Euryarchaeota	1,499519	R	-	Methanococc	Tetratricopeptide protein	-	-
65	1043	['3']	RO	24	21	-	1,231528	1,119363	-	Cyanobacteria	0,891767	L	-	Anabaena	DNA adenine methylase	-	-
640	90	['3']	RO	1	2	-	1,737575	2,063836	-	Alphaproteobacteria	1,737575	-	-	Rickettsia	hypothetical protein	-	-
439	59	['3']	RO	1	2	-	1,101299	2,987619	-	Alphaproteobacteria	1,101299	-	-	Rickettsia	hypothetical protein	-	-
55	17	['3']	RO	1	2	-	0,5493	1,29624	-	Alphaproteobacteria	0,5493	-	-	Rickettsia	Transposase and inactivated derivative	Gammaproteobacteria	Salmonella
452	231	['3']	ROW	1	3	2	0,925798	1,667205	1,51392	Alphaproteobacteria	0,925798	-	-	Rickettsia	hypothetical protein	-	-
552	1581	['3']	ROW	1	2	4	0,125782	0,615668	1,01227	Alphaproteobacteria	0,125782	R	-	Rickettsia	Phage prohead protease and phage major cap:	-	-
479	231	['3']	ROW	1	2	3	0,998607	1,417913	1,42774	Alphaproteobacteria	0,998607	-	-	Rickettsia	hypothetical protein	-	-
552	101	['3']	ROW	1	2	5	0,2284	1,090372	1,7961	Alphaproteobacteria	0,2284	R	-	Rickettsia	Phage prohead protease and phage major cap:	-	-
339	33	['3']	ROW	1	9	6	0,507285	2,645766	2,43356	Alphaproteobacteria	0,507285	R	-	Rickettsia	Ankyrin repeat	-	-
386	5	['3']	RW	1	-	17	0,304274	-	1,0189	Alphaproteobacteria	0,304274	L	-	Rickettsia	Transposase and inactivated derivative	-	-
565	886	['3']	RW	1	-	6	0,145	-	0,76094	Alphaproteobacteria	0,145	L	-	Rickettsia	Excinuclease ABC subunit C	-	-
106	1534	['3']	RW	1	-	13	0,539507	-	1,09456	Alphaproteobacteria	0,539507	R	-	Rickettsia	Putative virulence protein	-	-
546	5	['3']	RW	1	-	17	0,304274	-	1,0189	Alphaproteobacteria	0,304274	L	-	Rickettsia	Transposase and inactivated derivative	-	-
249	33	['3']	RW	1	-	2	0,806192	-	1,99063	Alphaproteobacteria	0,806192	R	-	Rickettsia	Ankyrin repeat	Cyanobacteria	Anabaena
387	886	['3']	RW	3	-	10	0,675966	-	0,86048	Alphaproteobacteria	0,546534	L	-	Nitrobacter	Excinuclease ABC, C subunit-like	-	-
631	5	['3']	RW	1	-	20	0,317119	-	0,91681	Alphaproteobacteria	0,317119	L	-	Rickettsia	Transposase and inactivated derivative	-	-
126	5	['3']	RW	1	-	17	0,304274	-	1,0189	Alphaproteobacteria	0,304274	L	-	Rickettsia	Transposase and inactivated derivative	-	-
581	5	['3']	RW	1	-	17	0,304274	-	1,0189	Alphaproteobacteria	0,304274	L	-	Rickettsia	Transposase and inactivated derivative	-	-
478	5	['3']	RW	1	-	17	0,304274	-	1,0189	Alphaproteobacteria	0,304274	L	-	Rickettsia	Transposase and inactivated derivative	-	-
334	886	['3']	RW	1	-	5	0,167944	-	0,7935	Alphaproteobacteria	0,167944	L	-	Rickettsia	Excinuclease ABC subunit C	-	-
193	898	['3']	W	-	-	1	-	-	2,12496	Alphaproteobacteria	2,124961	R	-	Wolbachia	ankyrin repeat domain protein	Cyanobacteria	Anabaena
36	1349	['3']	W	-	-	20	-	-	2,26657	Bacilli	0,924038	S	-	Geobacillus	huntingtin interacting protein E-like protein	-	-
641	1160	['3']	W	-	-	1	-	-	0,99656	Alphaproteobacteria	0,996558	JD	-	Wolbachia	hypothetical protein	Deltaproteobacteria	Pelobacter
50	1562	['3']	W	-	-	1	-	-	0,75209	Alphaproteobacteria	0,752086	S	-	Wolbachia	Fic family protein	-	-
191	1027	['4', '5', '6', '7']	OW	-	2	1	-	0,832887	0,83213	Alphaproteobacteria	0,83213	-	-	Wolbachia	hypothetical protein	-	-
595	1101	['4', '5', '6', '7']	R	1	-	-	0,231354	-	-	Alphaproteobacteria	0,231354	S	-	Rickettsia	hypothetical protein	-	-
550	1103	['4', '5', '6', '7']	R	1	-	-	0,469859	-	-	Alphaproteobacteria	0,469859	T	-	Rickettsia	PemK-like growth inhibitor	Gammaproteobacteria	Xylella
246	1096	['4', '5', '6', '7']	R	1	-	-	0,216647	-	-	Alphaproteobacteria	0,216647	-	-	Rickettsia	Transcriptional regulator	-	-
248	1105	['4', '5', '6', '7']	RO	1	2	-	0,466843	0,904887	-	Alphaproteobacteria	0,466843	L	radC	Rickettsia	DNA repair protein RadC	Alphaproteobacteria	Silicibacter
614	1173	['4', '5', '6']	ROW	1	2	4	0,141375	0,550381	0,79914	Alphaproteobacteria	0,141375	SR	-	Rickettsia	Putative phage terminase protein	-	-
550	1102	['4', '5']	R	1	-	-	0,377504	-	-	Alphaproteobacteria	0,377504	-	-	Rickettsia	hypothetical protein	Gammaproteobacteria	Xylella
636	1696	['4']															
182	886	['4']															
636	1695	['4']	None	-	-	-	-	-	-	Gammaproteobacteri:	0,312445	L	-	Xylella	DNA modification methylase	-	-
365	181	['4']	R	1	-	-	0,93954	-	-	Alphaproteobacteria	0,93954	C	nuoN1	Rickettsia	NADH:ubiquinone oxidoreductase subunit 2 (cl	-	-
609	1704	['4']	R	1	-	-	0,279348	-	-	Alphaproteobacteria	0,279348	L	-	Rickettsia	Transposase and inactivated derivative	-	-
308	1643	['4']	R	1	-	-	0,338573	-	-	Alphaproteobacteria	0,338573	-	-	Rickettsia	hypothetical protein	-	-
552	1656	['4']	R	1	-	-	0,632334	-	-	Alphaproteobacteria	0,632334	-	-	Rickettsia	hypothetical protein	-	-
454	36	['4']	R	1	-	-	0,434343	-	-	Alphaproteobacteria	0,434343	R	-	Rickettsia	Toxin of toxin-antitoxin (TA) system ParE	-	-
308	17	['4']	R	1	-	-	0,666197	-	-	Alphaproteobacteria	0,666197	-	-	Rickettsia	Transposase and inactivated derivative	-	-
127	1355	['4']	R	1	-	-	0,533647	-	-	Alphaproteobacteria	0,533647	V	-	Rickettsia	Type I restriction-modification system methylt	Deltaproteobacteria	Desulfovibrio
565	1678	['4']	R	1	-	-	0,402348	-	-	Alphaproteobacteria	0,402348	R	-	Rickettsia	hypothetical protein	-	-
196	35	['4']	R	1	-	-	0,521764	-	-	Alphaproteobacteria	0,521764	-	-	Rickettsia	hypothetical protein	-	-
268	98	['4']	R	1	-	-	0,323057	-	-	Alphaproteobacteria	0,323057	-	-	Rickettsia	hypothetical protein	-	-
396	33	['4']	R	1	-	-	0,951843	-	-	Alphaproteobacteria	0,951843	-	-	Rickettsia	Ankyrin repeat	-	-
391	7	['4']	RW	1	-	2	0,288592	-	0,47612	Alphaproteobacteria	0,288592	S	-	Rickettsia	hypothetical protein	Betaproteobacteria	Cupriavidus
166	1043	['5', '6', '7']	None	-	-	-	-	-	-	Clostridia	1,253582	L	-	Clostridium	putative adenine-specific DNA methyltransfera	-	-
240	980	['5', '6', '7']	None	-	-	-	-	-	-	Gammaproteobacteri:	1,00037	K	-	Nitrosococcu	transcriptional regulator, XRE family	-	-
605	1098	['5', '6', '7']	None	-	-	-	-	-	-	Alphaproteobacteria	1,791663	V	-	Rhizobium	probable N-acetylmuramoyl-L-alanine amidase	-	-
118	1211	['5', '6', '7']	None	-	-	-	-	-	-	Bacteroidetes/Chloro	1,640271	-	-	Chlorobium	hypothetical protein	-	-
166	1233	['5', '6', '7']	None	-	-	-	-	-	-	Alphaproteobacteria	1,460348	L	-	Rhodopseudc	ISSpo8, transposase	-	-
191	1097	['5', '6', '7']	OW	-	2	1	-	0,427375	0,39313	Alphaproteobacteria	0,393127	-	-	Wolbachia	hypothetical protein	-	-
426	772	['5', '6', '7']	R	1	-	-	0,81125	-	-	Alphaproteobacteria	0,81125	M	-	Rickettsia	Putative nucleoside-diphosphate-sugar epimer	-	-
245	926	['5', '6', '7']	R	1	-	-	0,223882	-	-	Alphaproteobacteria	0,223882	S	-	Rickettsia	Putative toxin of toxin-antitoxin (TA) system	Betaproteobacteria	Nitrosomonas
520	1260	['5', '6', '7']	R	1	-	-	1,7938	-	-	Alphaproteobacteria	1,7938	-	-	Rickettsia	hypothetical protein	-	-
157	1223	['5', '6', '7']	R	1	-	-	0,236711	-	-	Alphaproteobacteria	0,236711	R	-	Rickettsia	Putative nucleic-acid-binding protein	-	-
157	1224	['5', '6', '7']	R	1	-	-	0,095386	-	-	Alphaproteobacteria	0,095386	K	-	Rickettsia	Transcriptional regulator	-	-

443	1231	['5', '6', '7']	R	1	-	-	0,20878	-	-	Alphaproteobacteria	0,20878	-	-	Rickettsia	hypothetical protein	-	-
262	1092	['5', '6', '7']	R	1	-	-	0,189356	-	-	Alphaproteobacteria	0,189356	-	-	Rickettsia	Putative antitoxin of toxin-antitoxin (TA) syst	-	-
134	1253	['5', '6', '7']	R	1	-	-	0,216828	-	-	Alphaproteobacteria	0,216828	-	-	Rickettsia	Alpha-(1,3)-fucosyltransferase	-	-
653	1249	['5', '6', '7']	R	1	-	-	0,901091	-	-	Alphaproteobacteria	0,901091	-	proP9_2	Rickettsia	Proline/betaine transporter	-	-
391	1252	['5', '6', '7']	R	1	-	-	0,493183	-	-	Alphaproteobacteria	0,493183	S	-	Rickettsia	Transposase and inactivated derivative	-	-
199	1109	['5', '6', '7']	R	1	-	-	0,862664	-	-	Alphaproteobacteria	0,862664	-	-	Rickettsia	hypothetical protein	-	-
245	889	['5', '6', '7']	R	1	-	-	0,240132	-	-	Alphaproteobacteria	0,240132	D	-	Rickettsia	Antitoxin of toxin-antitoxin (TA) system StbD	Betaproteobacteria	Azoarcus
246	899	['5', '6', '7']	R	1	-	-	0,228633	-	-	Alphaproteobacteria	0,228633	S	-	Rickettsia	hypothetical protein	Fibrobacteres/Acidobact	unclassified Acidobacteriac
231	986	['5', '6', '7']	R	1	-	-	1,180335	-	-	Alphaproteobacteria	1,180335	-	-	Rickettsia	hypothetical protein	-	-
599	57	['5', '6', '7']	R	1	-	-	0,299343	-	-	Alphaproteobacteria	0,299343	-	-	Rickettsia	hypothetical protein	-	-
262	1106	['5', '6', '7']	R	1	-	-	0,287381	-	-	Alphaproteobacteria	0,287381	R	vapC3	Rickettsia	Toxin of toxin-antitoxin (TA) system VapC	Betaproteobacteria	Nitrosomonas
118	1208	['5', '6', '7']	ROW	1	2	22	0,245044	0,748201	1,19076	Alphaproteobacteria	0,245044	O	-	Rickettsia	Mg chelatase-related protein	-	-
599	1029	['5', '6', '7']	RW	1	-	4	0,295422	-	1,24614	Alphaproteobacteria	0,295422	M	-	Rickettsia	Mannose-1-phosphate guanylyltransferase	-	-
595	35	['5', '6', '7']	RW	1	-	5	0,325906	-	1,75157	Alphaproteobacteria	0,325906	-	-	Rickettsia	hypothetical protein	-	-
310	1077	['5', '6', '7']	RW	1	-	23	0,22903	-	2,53579	Alphaproteobacteria	0,22903	S	-	Rickettsia	Cell filamentation protein Fic	-	-
314	1268	['5', '6']															
609	1272	['5', '6']															
338	1014	['5', '6']															
127	1274	['5', '6']	None	-	-	-	-	-	-	Epsilonproteobacteri	1,112057	V	-	Helicobacter	type I restriction/modification enzyme	-	-
33	1100	['5', '6']	R	1	-	-	0,713152	-	-	Alphaproteobacteria	0,713152	-	-	Rickettsia	hypothetical protein	-	-
595	1256	['5', '6']	R	1	-	-	0,182558	-	-	Alphaproteobacteria	0,182558	-	-	Rickettsia	hypothetical protein	-	-
134	1254	['5', '6']	R	1	-	-	0,584989	-	-	Alphaproteobacteria	0,584989	M	-	Rickettsia	Glycosyltransferase	-	-
113	1184	['5', '6']	R	1	-	-	1,18263	-	-	Alphaproteobacteria	1,18263	-	-	Rickettsia	hypothetical protein	-	-
443	1385	['5', '6']	R	1	-	-	0,532642	-	-	Alphaproteobacteria	0,532642	-	-	Rickettsia	hypothetical protein	-	-
127	1431	['5', '6']	R	1	-	-	0,147161	-	-	Alphaproteobacteria	0,147161	V	-	Rickettsia	Type I restriction-modification system methylt	-	-
546	1247	['5', '6']	R	1	-	-	0,493369	-	-	Alphaproteobacteria	0,493369	V	-	Rickettsia	Type I site-specific restriction-modification sys	-	-
519	1239	['5', '6']	R	1	-	-	0,409229	-	-	Alphaproteobacteria	0,409229	-	-	Rickettsia	hypothetical protein	-	-
134	1120	['5', '6']	R	1	-	-	0,380865	-	-	Alphaproteobacteria	0,380865	-	-	Rickettsia	Alpha-(1,3)-fucosyltransferase	-	-
653	76	['5', '7']															
401	1215	['5', '7']	None	-	-	-	-	-	-	Gammaproteobacteri	0,622825	S	-	Coxiella	hypothetical protein	-	-
256	1429	['5', '7']	None	-	-	-	-	-	-	Betaproteobacteria	0,284552	S	-	Cupriavidus	Plasmid stabilization system	-	-
517	1384	['5', '7']	R	1	-	-	0,629564	-	-	Alphaproteobacteria	0,629564	UN	fimD	Rickettsia	P pilus assembly, fimbrial Usher protein	-	-
224	1219	['5', '7']	R	1	-	-	0,628874	-	-	Alphaproteobacteria	0,628874	V	-	Rickettsia	RND family efflux transporter	Deltaproteobacteria	Geobacter
400	1373	['5', '7']	R	1	-	-	0,511651	-	-	Alphaproteobacteria	0,511651	V	mdlB	Rickettsia	ABC-type multidrug transport system, ATPase	-	-
401	1214	['5']	None	-	-	-	-	-	-	Cyanobacteria	0,853976	-	-	Synechococc	hypothetical protein	-	-
79	1162	['5']	R	2	-	-	0,738828	-	-	Gammaproteobacteri	0,718101	R	-	Sodalis	phage lysozyme lysis protein	-	-
204	1743	['5']	R	1	-	-	0,951088	-	-	Alphaproteobacteria	0,951088	L	-	Rickettsia	Superfamily I DNA and RNA helicases	-	-
328	167	['5']	R	1	-	-	0,465193	-	-	Alphaproteobacteria	0,465193	-	-	Rickettsia	hypothetical protein	-	-
601	1225	['6', '7']															
36	1035	['6', '7']	None	-	-	-	-	-	-	Alphaproteobacteria	1,602602	I	-	Magnetospiri	Predicted acyltransferase	-	-
483	1218	['6', '7']	None	-	-	-	-	-	-	Spirochaetes	1,016052	R	vatB	Leptospira	acetyltransferase	-	-
33	1099	['6', '7']	R	1	-	-	0,624347	-	-	Alphaproteobacteria	0,624347	-	-	Rickettsia	hypothetical protein	-	-
240	1055	['6']	None	-	-	-	-	-	-	Betaproteobacteria	1,297191	-	relE	Azoarcus	RelE-like Cytotoxic translational repressor of t	-	-
546	1405	['6']	R	1	-	-	0,883494	-	-	Alphaproteobacteria	0,883494	V	-	Rickettsia	Type I site-specific restriction-modification sys	-	-
34	886	['6']	R	1	-	-	0,358279	-	-	Alphaproteobacteria	0,358279	L	-	Rickettsia	Excinuclease ABC subunit C	-	-
191	35	['7']															
314	1269	['7']															
276	145	['7']	R	1	-	-	0,223475	-	-	Alphaproteobacteria	0,223475	T	-	Rickettsia	Regulatory components of sensory transducti	-	-
33	1226	['7']	R	1	-	-	0,700588	-	-	Alphaproteobacteria	0,700588	-	-	Rickettsia	hypothetical protein	-	-
433	1232	['7']	R	1	-	-	1,32592	-	-	Alphaproteobacteria	1,32592	-	-	Rickettsia	hypothetical protein	-	-
581	1445	['7']	RO	1	9	-	0,970986	1,98899	-	Alphaproteobacteria	0,970986	-	-	Rickettsia	Tetratricopeptide repeat-containing protein	-	-
